# Efficient weighted univariate clustering maps outstanding dysregulated genomic zones in human cancers

**DOI:** 10.1093/bioinformatics/btaa613

**Published:** 2020-07-03

**Authors:** Mingzhou Song, Hua Zhong

**Affiliations:** Department of Computer Science; Molecular Biology Graduate Program, New Mexico State University, Las Cruces, NM 88003, USA; Department of Computer Science

## Abstract

**Motivation:**

Chromosomal patterning of gene expression in cancer can arise from aneuploidy, genome disorganization or abnormal DNA methylation. To map such patterns, we introduce a weighted univariate clustering algorithm to guarantee linear runtime, optimality and reproducibility.

**Results:**

We present the chromosome clustering method, establish its optimality and runtime and evaluate its performance. It uses dynamic programming enhanced with an algorithm to reduce search-space in-place to decrease runtime overhead. Using the method, we delineated outstanding genomic zones in 17 human cancer types. We identified strong continuity in dysregulation polarity—dominance by either up- or downregulated genes in a zone—along chromosomes in all cancer types. Significantly polarized dysregulation zones specific to cancer types are found, offering potential diagnostic biomarkers. Unreported previously, a total of 109 loci with conserved dysregulation polarity across cancer types give insights into pan-cancer mechanisms. Efficient chromosomal clustering opens a window to characterize molecular patterns in cancer genome and beyond.

**Availability and implementation:**

Weighted univariate clustering algorithms are implemented within the R package ‘Ckmeans.1d.dp’ (4.0.0 or above), freely available at https://cran.r-project.org/package=Ckmeans.1d.dp.

**Supplementary information:**

Supplementary data are available at *Bioinformatics* online.

## 1 Introduction

Cancer has been characterized by abnormal gene activity, such as loss of function of tumor-suppressor genes (Liotta *et al.*, 1991; Woo *et al.*, 2017). However, single-gene characterization does not convey cancer transcriptome patterns associated with genomic regions, possibly resulting from aneuploidy (Turkheimer *et al.*, 2006), DNA methylation heterogeneity (Wang *et al.*, 2015b) or genome disorganization (Achinger-Kawecka and Clark, 2017; Kaiser and Semple, 2017; Taberlay *et al.*, 2016). Driven by allelic imbalance, entire chromosomal arms of 3p (-) and 22q (+) are differentially expressed in head and neck squamous cell carcinoma (Masayesva *et al.*, 2004). Levesque and Raj (2013) observed that a set of genes on a translocated chromosome 19 is transcribed much more than the same set of genes on the intact copy of the chromosome in human cervical cancer cells. Schwarzer *et al.* (2017) found that megabase-sized active/inactive compartments and submegabase topologically associating domains (TADs) spatially insulate gene expression into zones along chromosomes. Boundaries of TAD can be disrupted in tumor cells (Flavahan *et al.*, 2016). Such chromosomal expression patterning motivated us to characterize cancer transcriptome by examining gene dysregulation in genomic zones along chromosomes.

Early approaches to finding chromosomal expression patterns operate locally without global optimality. Vogel *et al.* (2005) clustered genes in heart tissues along chromosomes such that all genes in a genomic interval must be differentially expressed, stressing local over global expression patterns. MACAT (Toedling *et al.*, 2005), LAP (Callegaro *et al.*, 2006) and SODEGIR (Bicciato *et al.*, 2009) smooth gene expression along chromosomes using kernels, incompatible with sharp chromatin boundaries due to compartmentalization or TAD insulation. Chromosomal expression was studied using cytogenetic bands (Wang *et al.*, 2015a), which are predefined thus not adaptive to data. ClusterScan requires user-specified parameters, a maximum feature distance or a window size, inflexible to varying resolutions of chromosomal events (Volpe *et al.*, 2018). More recently, chromosomal segmentation has been applied to find expression-driven genomic zones. Optimizing the constancy of gene activity, segmentation is less sensitive to gene proximity than clustering. Chromosomal transcription patterns in *Drosophila* (Rubin and Green, 2013) were studied by fitting a Gaussian mixture model to capture segmental gene expression. Later chromosomal segmentation approaches used dynamic programming to achieve global optimality. SegCorr (Delatola *et al.*, 2017) uses dynamic programming of runtime $O(kn^{2})$, where *k* is the number of segments and *n* is the number of genes. Rendersome (Nilsson *et al.*, 2008) minimized total variation within segments using dynamic programming also at a quadratic runtime. The runtime of segmentation is a roadblock to analyzing a large number of genomic elements, such as transcription start sites when both coding and noncoding genes are considered.

To overcome such issues, we present a chromosomal clustering method using a fast, optimal and reproducible weighted univariate clustering (WUC) algorithm. Based on the linear-time SMAWK algorithm for dynamic programming speedup (Aggarwal *et al.*, 1987), WUC innovates with an in-place procedure to substantially reduce the runtime overhead, becoming practical on large sample sizes. We also describe a statistical routine to estimate the number of clusters using the Gaussian mixture model. We illustrate the improved empirical runtime, guaranteed optimality and reproducibility of WUC in comparison with other clustering methods.

By chromosomal clustering, we mapped polarized genomic zones on transcriptomes of matched tumor-normal pairs from 17 human cancer types. A zone is polarized in regulation if either up- or downregulated genes within the zone dominate in proportion. In parallel, on somatic copy number data with both tumor and matched normal sample pairs from the same 17 cancer types, we found that zone polarization in somatic copy number alteration (SCNA) agrees well with known cancer genome instability. We observed a weak positive correlation in zone polarization between regulation and SCNA: zone polarity in SCNA is often not transcribed to zone polarity in regulation in tumors versus matched normal. Top polarized dysregulation zones are highly specific to cancer types. The polarized zones are enriched for known genetic and epigenetic events associated with cancer. Most importantly, a total of 109 loci distributed on 21 chromosomes are found to be conserved in dysregulation polarity across 14 (>80%) or more cancer types. These conserved loci are largely independent of SCNA, constituting a unique pan-cancer transcriptomic characteristic.

Applicable to genomic features of any organism with genome sequence scaffolds, the chromosome clustering method now can solve problems of large sample sizes on long chromosomes. It can be used in studying spatial properties of genome, transcriptome and epigenome, opening a window to characterize patterns of molecular activity along chromosomes in genomes.

## 2 Materials and methods

### 2.1 Optimal, fast and reproducible solution to weighted univariate clustering

We state the WUC problem and give its dynamic programming solutions. Given an array of *n* sorted numbers $x_{0}\leq x_{1}\leq \cdots \leq x_{n-1}$ with non-negative weights *y*_0_, *y*_1_, $\ldots ,\;y_{n-1}$, we define a *k*-clustering *C*(*k*, *n*) as *k* non-overlapping intervals to cover 0,…,n−1:

(1) $$C(k,n)=\{\underset{\text{cluster}\;0}{\underset{︸}{\left[j_{0}+1,j_{1}\right]}},\underset{\text{cluster}\;1}{\underset{︸}{\left[j_{1}+1,j_{2}\right]}},\cdots ,\underset{\text{cluster}\;k-1}{\underset{︸}{\left[j_{k-1}+1,j_{k}\right]}}\}$$

where $j_{0}=-1<j_{1}<\cdots <j_{k}=n-1$. $j_{k-1}+1$ and *j_k_* are the lower and upper decision boundaries of cluster *k* – 1. Let μ(j,i) be the weighted mean function defined from *x_j_* to *x_i_*:

(2) $$\mu (j,i)=\frac{1}{Y_{i}-Y_{j-1}}\sum_{l=j}^{i}x_{l}y_{l}\;(j\leq i)$$

where $Y_{i}=\sum_{l=0}^{i}y_{l}$ for i≥0 and $Y_{-1}=0$. We define the sum of squared distances function *s*(*j*, *i*) from each point in $\left[x_{j},x_{i}\right]$ to the mean μ(j,i) as

(3) $$s(j,i)=\{\begin{array}{cc} \sum_{l=j}^{i}y_{l}{\left[x_{l}-\mu (j,i)\right]}^{2} & j\leq i\\ \infty & j>i \end{array}$$

For a clustering *C*(*k*, *n*), the total sum of squared distances is

(4) $$\mathrm{SSQ}(C(k,n))=\sum_{q=0}^{k-1}s(j_{q}+1,j_{q+1})=\sum_{i=0}^{n-1}y_{i}x_{i}^{2}-\sum_{q=0}^{k-1}\left(Y_{j_{q+1}}-Y_{j_{q}}\right)\mu^{2}(j_{q}+1,j_{q+1})$$

The WUC problem is to find a clustering $C^{*}(k,n)$ to minimize SSQ(C(k,n)).

Bellman (1973) gave the first dynamic programming solution. Let *S* be a *k *×* n* matrix:

(5) $$S[q,i]=\underset{C}{\min}\{\mathrm{SSQ}(C(q+1,i+1))\}\;q=0,\ldots ,k-1;\;i=0,\ldots ,n-1$$

which is the minimum *SSQ* when *x*_0_ to *x_i_* are grouped by an optimal clustering $C^{*}(q+1,i+1)$. Let J[q,i] in another *k *×* n* matrix *J* be the smallest index to the points in cluster *q* of $C^{*}(q+1,i+1)$. The following defines recurrence equations for dynamic programming:

(6) $$S[q,i]=\underset{q\;\leq \;j\;\leq \;i}{\min}\;S[q-1,j-1]+s(j,i)\;0<q\leq i$$

 

(7) $$J[q,i]=\max\left\{j\;|\underset{q\;\leq \;j\;\leq \;i}{\text{argmin}}\;S[q-1,j-1]+s(j,i)\right\}\;0<q\leq i$$

For multiple optimal solutions, we assign the maximum of all optimal indices to J[q,i]. Matrices *S* and *J* are initialized by S[0,i]=s(0,i),J[0,i]=0  (0≤i≤n−1). To prevent empty clusters, we set S[q,i]=∞ and J[q,i] undefined when *i *<* q*. As solving Eqs. (6) and (7) directly leads to cubic $O(kn^{3})$ runtime, previous work sped up the dynamic programming to quadratic $O(kn^{2})$ and log-linear O(k n lg n) time, as reviewed in Supplementary Note N5.

We present a linear *O*(*kn*) solution to WUC based on a *total monotone* property—even if we constrain the cluster boundaries to fall on a subset of the data points, the constrained optimal boundaries will not decrease if additional points greater than the current points in the subproblem are inserted. This property enables one to fill out each row of the dynamic programming matrix *S* in linear time by calling the SMAWK algorithm.

We first show WUC satisfies a concave quadrangle inequality (Theorem 1). Then we formulate subproblems of WUC as row-minima search in totally monotonic matrices (Lemma 2, Theorem 3). We further improve SMAWK by an in-place algorithm to perform search-space reduction in an array, instead of deleting matrix columns. We prove the algorithm always terminates (Theorem 4), is correct (Theorem 5), and runs in linear time to *n*.

Theorem 1 *For any four increasing indices* $0\leq i_{1}\leq i_{2}\leq i_{3}\leq i_{4}\leq n-1$  *to sequence* $x_{0},\ldots ,x_{n-1}$  *already sorted in ascending order, s(j, i) satisfies the concave quadrangle inequality* $s(i_{2},i_{3})+s(i_{1},i_{4})\geq s(i_{2},i_{4})+s(i_{1},i_{3})$*. (Proof given in Supplementary Note N5)*

Transforming the WUC problem to *k* – 1 subproblems of matrix search, we define an *n *×* n* clustering matrix *A*(*q*) (q=1,…,k−1) via its element at row *i* and column *j*:

(8) $$A{\left(q\right)}_{i,\;j}=\{\begin{array}{cc} S[q-1,j-1]+s(j,i) & 1\leq q\leq j\leq i<n\\ +\infty & 0\leq j<q\;\text{or}\;i<j<n \end{array}$$

Definition 1 *The optimal index function* $j_{q}(i)$  *maps i in given cluster q to the largest index that achieves the minimum* S[q,i]  *by* $j_{q}(i)=J[q,i]$.

As our derivation next is for a fixed *q*, we thus drop *q* to simplify *A*(*q*) to *A* and $j_{q}(i)$ to *j*(*i*)—the largest index to the minimum element in row *i* of *A*.

Definition 2 *Matrix A is monotonic if and only if* $j(i_{1})\leq j(i_{2})$  *is true for any* $i_{1}<i_{2}$.

Definition 3 *Matrix A is totally monotonic if and only if every submatrix of A is monotonic.*

Lemma 2 **(2 × 2 matrix monotonicity)**. *Let* A′  *be a 2 × 2 submatrix of A defined by*
 

(9) $$A\prime =\left[\begin{array}{cc} A_{i_{1},\;j_{1}} & A_{i_{1},\;j_{2}}\\ A_{i_{2},\;j_{1}} & A_{i_{2},\;j_{2}} \end{array}\right]$$

*where* $i_{1}<i_{2}$  *and* $j_{1}<j_{2}$*. Let* j′(i′)∈[0,1]  *be the largest column index of the minimum element in row* i′∈[0,1]  *in* A′*. Then we have* j′(0)≤j′(1)*. (Proof given in Supplementary Note N5)*

Theorem 3 *Clustering matrix A is totally monotonic. (Proof given in Supplementary Note N5)*

The linear-time solution to a totally monotonic matrix search subproblem relies on the search-space reduction algorithm Reduce(A) (Aggarwal *et al.*, 1987). For a totally monotonic *N *×* M* matrix *A* (Theorem 3), it trims down columns in *A* to no more than *N* in linear time O(N+M), while still preserving the optimal solutions. If M≤N, no column reduction is performed. We adapt the original Reduce algorithm to Reduce-Min with three changes. Reduce-Min preserves row minima instead of maxima; all indices are 0-based instead of 1-based; and multiple optimal solutions are broken by taking the larger column index, to be consistent with previous versions of the ‘Ckmeans.1d.dp’ package.

Definition 4 *An entry* $A_{i,\;j\prime }$  *is infeasible if* j′≠j(i). *Column* j′  *of A is infeasible if* $A_{i,\;j\prime }$  *is infeasible for every i. Among multiple optimal solutions, the one with the largest index is feasible and the others infeasible.*

As maintaining a copy of matrix *A* would require at least Ω(NM) time, the algorithm must compute only needed entries in *A* in constant time without storing the entire matrix *A*. For WUC, this is possible by maintaining only indices of feasible columns in *A* using a stack (http://www.ics.uci.edu/~eppstein/PADS/SMAWK.py) or a preallocated linked list of input size (Luessi *et al.*, 2009). Still, these implementations require either dynamic memory allocation or pointer maintenance within the while-loop, thus carrying considerable runtime overhead. To reduce this overhead, we accomplish column reduction in an array of length *M* in place as given in the Reduce-Min-In-Place algorithm.

Reduce-Min(*A*: N×M total monotonic matrix)

1.  p=0

2.  **while** *A* has more than *N* columns

3.    **if**  $A_{p,\;p}<A_{p,\;p+1}$ and *p* < N−1

4.      p=p+1

5.    **elseif**  $A_{p,\;p}<A_{p,\;p+1}$ and p≡ N−1

6.      delete column N of *A*

7.    **elseif**  $A_{p,\;p}\geq A_{p,\;p+1}$

8.      delete column *p* of *A*

9.      **if**  p>0

10.        p=p−1

11.  **return** *A*

Reduce-Min-In-Place(*cols*, *N*, *A*)

1. *M* = length(*cols*)

2. **if**  M≤N  **return** *cols*

3. l=−1 **//** cols[0..l]: l+1 column indices examined and feasible so far

4. r=0  **//** cols[r..M−1]: M−r column indices to be examined

5. **while**  (l+1)+(M−r)>N

6.    p= l+1

7.    j= cols[r]

8.    $j_{+}=\;\mathrm{cols}[r+1]$

9.    **if**  $A_{p,\;j}<A_{p,\;j_{+}}$ and p<N−1

        **//** $A_{p,\;j}\equiv \;A(p,j),\;A_{p,\;j_{+}}\equiv \;A(p,j_{+})$

10.      l=l+1

11.      cols[l]= j

12.      r=r+1

13.      **elseif**  $A_{p,\;j}<A_{p,\;j_{+}}$ and p≡ N−1

        **//** Column $j_{+}$ of *A* is infeasible

14.      cols[r+1]=j

15.       r=r+1

16.      **elseif**  $A_{p,j}\geq A_{p,\;j_{+}}$  **//** Column *j* of *A* is infeasible

17.      **if**  p>0

18.        cols[r]= cols[l]

19.        l=l−1

20.      **else**

21.        r=r+1

22.  cols[l+1..N−1]= cols[r..M−1]

23.  **return**  cols[0..N−1]

The algorithm moves feasible column indices toward the beginning of the column index array. Inside the while-loop, the column index array is used in place without dynamic memory deallocation of infeasible column indices. It realizes the original Reduce algorithm using the simplest data structure with minimal runtime and memory overhead as compared to previous solutions.

Theorem 4 Reduce-Min-In-Place *always terminates. (Proof given in Supplementary Note N5)*

Theorem 5 Reduce-Min-In-Place *correctly removes only infeasible candidate columns from the input matrix. The output matrix has no more columns than rows. Additionally, the output matrix is still totally monotonic. (Proof given in Supplementary Note N5)*

The SMAWK algorithm reduces columns from *A* first, recursively solves a submatrix containing the odd rows, and then calculates solutions to the even rows. We present the Fill-Row-SMAWK algorithm following the same strategy without explicitly maintaining matrix *A*, to calculate an entire row in matrix *S*. Fill-Row-SMAWK is called by Algorithm Weighted-Univariate-Clustering-(WUC)-Linear to compute the entire dynamic programming matrix (Supplementary Note N5).

Reduce-Min-In-Place runs in O(N+M), the same with the Reduce algorithm, as manipulating the array adds only a constant factor. Thus, the runtime of Fill-Row-SMAWK on input matrix of size N×M is T(N,M)=T(N/2,N/2)+O(N+M)=O(N+M). As N≤n and M≤n, the runtime for the entire row of *n* elements in *S* is T(n)=O(n). Therefore, the total runtime of WUC on *n* input points is *O*(*kn*) as *x* is sorted. Although additional space in *O*(*n*) is needed to store running sums and candidate indices during the recursion, the space complexity remains *O*(*kn*) when backtrack must be conducted.

When the number of clusters is given by a range $\left[k_{\min},k_{\max}\right]$, the runtime of each algorithm replaces *k* by $k_{\max}$, as solving for $k_{\max}$ would automatically generate results for smaller *k* values. To choose an optimal number of clusters from the range for *k*, we use the Bayesian information criterion that promotes likelihood based on a Gaussian mixture model and penalizes the number of components in the model. This is an important option for chromosomal clustering where the number of clusters is typically unknown. We describe a self-contained theoretical framework with full details of all relevant algorithms in Supplementary Note N5.

### 2.2 Mapping genomic zones and their polarity in human cancers

#### 2.2.1 Human cancer transcriptome profiles

We selected 17 cancer types from National Cancer Institute (NCI) Genomic Data Common (GDC) (Grossman *et al.*, 2016), requiring each cancer type having at least eight pairs of tumor and matched normal RNA-seq samples. We downloaded the HTSeq count files via the R package ‘TCGAbiolinks’ 2.10.2 (Colaprico *et al.*, 2016). The counts are the total number of raw reads sequenced from full mRNA transcripts and aligned to each gene. One library of a colon adenocarcinoma (COAD) patient was removed as an outlier, due to its much lower sequencing depth (fewer than 8 million read counts) than other COAD patients. We used 707 tumor and 683 matched normal tissue transcriptomes from these 17 cancer types; other tumor transcriptomic data without matched normal tissues were not included in our study. Human reference genome GRCh38.p12 and GENCODE human genome annotation v29 were used in our analysis.

Raw read counts mapped to each gene were normalized within and across samples to reduce sequencing biases, using the upper quantile methods for within-lane and also between-lane correction, global-scaling and full-quantile normalization from the R package ‘EDASeq’ (Risso *et al.*, 2011) as integrated into the TCGAanalyze_Normalization function in the ‘TCGAbiolinks’ package. The GC content of a gene was calculated as the percentage of GC on all exons of the gene; biases due to GC content were removed by normalization. Normalized numbers of reads per gene were further linearly scaled by the total number of reads in each sample to counts per million (CPM).

Read counts not normalized by gene size reflect transcription density in a genomic neighborhood. This will ensure that gene clusters along a chromosome to be equally spread when the same number of transcripts is expressed by genes of different lengths; otherwise, shorter genes will be in compact clusters inconsistent with the biology that all genes are expressed at the same abundance. In zone polarity calling, the ratio of a gene in cancer versus in normal is used which is insensitive to gene size.

Occasionally, a patient can have more than one tumor and one normal profiles. In genomic zone mapping, all profiles for a patient were used to delineate cluster boundaries weighed by ensemble expression levels of genes. In zone polarity determination, however, only one matched pair for each unique patient was used for statistical testing.

#### 2.2.2 Mapping cancer genomic zones by chromosomal clustering

For each cancer type, we pooled its tumor and matched normal transcriptomes to compute genomic zones using WUC. Pooling makes zone mapping sensitive to transcription activities in both tumors and normal tissues. We clustered transcription start sites as the position of each gene weighed by its expression level for each of chromosomes 1–22, X and Y. Specifically, the position of a gene on the forward strand of human reference genome is its start coordinate; otherwise, the position is the end coordinate for a gene on the reverse strand. For each chromosome, the resolution *r* of genomic zone was set to 1 Mb to match the typical size of a TAD. This effect of resolution *r* is to impose an upper limit on the total number of clusters; the actual width of a cluster is automatically determined which can be either less than or greater than the resolution. Let *L* be the length in base pair of a chromosome. Let *G* be the number of annotated genes to be clustered on that chromosome. The maximum number of clusters along a chromosome was set to $k_{\max}=\max\{20,\min\{G/5,\left\lceil L/r\right\rceil \}\}$, where $k_{\max}$ is at least 20, the average number of genes in each cluster is at least 5, and the minimum average cluster width is *r*. As long as $k_{\max}$ is large enough, the clustering result will be insensitive to the actual value of $k_{\max}$, because an optimal number of clusters is automatically chosen between two and $k_{\max}$ using BIC during clustering. Clustering in a genomic neighborhood is fine-grained where either cancer or normal tissue shows high transcription activity; otherwise, clustering is coarse-grained such as around the centromere of each chromosome.

The WUC algorithms are implemented in the ‘Ckmeans.1d.dp’ package version ≥ 4.0.0. The input includes starting genomic coordinates of genes, expression levels as weights and the range of *k* as defined above. The output is an optimal clustering. Genomic zones are further declared by the adaptive histogram R function ahist() also available in the same package. Instead of always setting zone boundaries at midpoints between two consecutive clusters, ahist() puts boundaries between clusters such that a long stretch of chromosomal region without any gene such as centromere has its own empty zone. Specifically, the upper and lower zone boundaries of a cluster can only extend from its left- and right-most locations by an amount up to the maximum distance between a pair of consecutive points within the cluster. If the midpoint with the left neighboring cluster is between the lower and upper zone boundaries, the lower zone boundary will shrink to the midpoint; the upper zone boundary is similarly adjusted. Therefore, the number of zones may be greater than the number of clusters, as new empty zones may have been created. The specifics of determining zone boundaries have no impact on the determination of zone polarity to be described next. Its main utility is to provide a more accurate visualization of those genomic regions with no detectable activities along chromosomes.

#### 2.2.3 Calculating the polarity of a genomic zone

We determine the polarity of a zone by the disproportion between positive and negative activity of genes within the zone. For transcriptome data, gene activity is measured by expression level; for SCNA data, gene activity is measured by copy number. We define a gene to be positive/negative in a matched tumor-normal pair by requiring a minimum of 5% increase/decrease in activity level:

(10) $$\text{Gene}\;\text{polarity}=\{\begin{array}{cc} +, & \;\text{log}\;\frac{\text{cancer}+1}{\text{normal}+1}>\text{log}\;1.05\\ -, & \;\text{log}\;\frac{\text{cancer}+1}{\text{normal}+1}<-\text{log}\;1.05\\ \text{none}, & \text{otherwise} \end{array}$$

This criterion is a condition on effect size. The statistical significance of a zone will be determined by the collective behavior of genes within the zone. The polarity of a zone is positive/negative if there are more genes with positive/negative matched pairs; otherwise, the zone has no polarity:

(11) $$\text{Zone}\;\text{polarity}=\{\begin{array}{cc} +, & \#\text{positivegenes}>\#\text{negativegenes}\\ -, & \#\text{positivegenes}<\#\text{negativegenes}\\ \text{none}, & \text{otherwise} \end{array}$$

We determine the statistical significance of a zone by Pearson’s chi-squared test on the contingency table:

**Table:** 

**# positive pairs in zone**	**# positive pairs outside zone**
# negative pairs in zone	# negative pairs outside zone

Resulting *P*-values of the test on all zones of a given cancer type were corrected by Benjamini–Hochberg adjustment. If the adjusted *P*-value of a zone is no more than 0.05, we call the zone outstanding.

#### 2.2.4 Human cancer somatic copy number alternation profiles and SCNA polarization maps

Somatic copy number variation data of patients for the same 17 cancer types were downloaded from NCI GDC via ‘TCGAbiolinks’ 2.10.2. The data include 633 tumor and 633 matched normal copy number variation profiles in the format of copy number segments. Each unique patient has one copy number profile from cancer and one form matched normal tissue. We mapped SCNA data to the same zones in the genomic zone maps obtained from the transcriptome data. Specifically, within each genomic zone, we obtain its copy number defined by the average copy number weighted by segment length in a sample. The polarity of a zone is further determined using the same method in Eq. (11) but applied on the zone copy number profiles.

## 3 Results

### 3.1 Fast, optimal and reproducible weighted univariate clustering

#### 3.1.1 Overview of the weighted univariate clustering algorithm

We present the WUC algorithm at the core of the chromosomal clustering method. The input to WUC includes a sorted array of *n* real numbers, *n* non-negative weights and a range of integers up to *k* to select an optimal number of clusters. WUC transforms the clustering problem to *k* matrix search subproblems solvable by the SMAWK algorithm (Aggarwal *et al.*, 1987). We improve the SMAWK algorithm by an in-place procedure for search space reduction using an array, instead of a matrix or a linked list (Hershberger and Suri, 1997). We prove that WUC is correct and always terminates in *O*(*kn*) time. WUC not only achieves theoretical optimality on the weighted within-cluster sum of squared distances, but also greatly outperforms mainstream heuristic clustering methods on both internal and external cluster quality measures.

#### 3.1.2 Optimal weighted univariate clustering produces best quality clusters

The WUC algorithm provably minimizes the weighted within-cluster sum of squared distances (SSQ), a widely used objective function for cluster analysis (see Section 2). Now, we empirically evaluate its performance using two established cluster quality measures: the average silhouette width (ASW) (Rousseeuw, 1987) and adjusted Rand index (ARI) (Hubert and Arabie, 1985), neither equivalent to SSQ nor biased over the number of clusters *k*. For both measures, a higher value indicates a better cluster quality. ASW, an internal measure for cluster validation, evaluates the relative distance between a point and other points within the same cluster against those in the nearest cluster. Sharply different from silhouette, ARI is an external measure not based on distance. Instead, it compares a clustering against the ground-truth clustering by their agreement in contrast to chance. We compare both measures on our clustering results with mainstream heuristic methods, including model-based clustering, heuristic *k*-means and hierarchical clustering. Specifically, we used R package ‘mclust’ (Scrucca *et al.*, 2016) for finite Gaussian mixture model (GMM)-based clustering, four heuristic *k*-means options—‘Hartigan-Wong’ (Hartigan and Wong, 1979), ‘Lloyd’ (Lloyd, 1982), ‘Forgy’ (Forgy, 1965) and ‘MacQueen’ (MacQueen, 1967)—by R function kmeans (nstart = 1), and three hierarchical clustering linkage options—single, average-UPGMA and complete—by R function hclust in package ‘stats’ (R Core Team, 2016). To make our performance evaluation general, we used three datasets from diverse domains: the optical density of protein DNase from R package ‘datasets’ (*n *=* *176), a simulated GMM of five components (*n *=* *251) and locations of dinucleotide CC on the plus strand of the 16 569 bp human mitochondrial (MT) genome (*n *=* *1771). The datasets are unweighted, as only WUC supports unequal weights. The ARI is evaluated only on GMM data with fixed *k *=* *5 in three sample sizes, as the other two datasets do not have ground-truth clusterings.

**Fig. 1.**
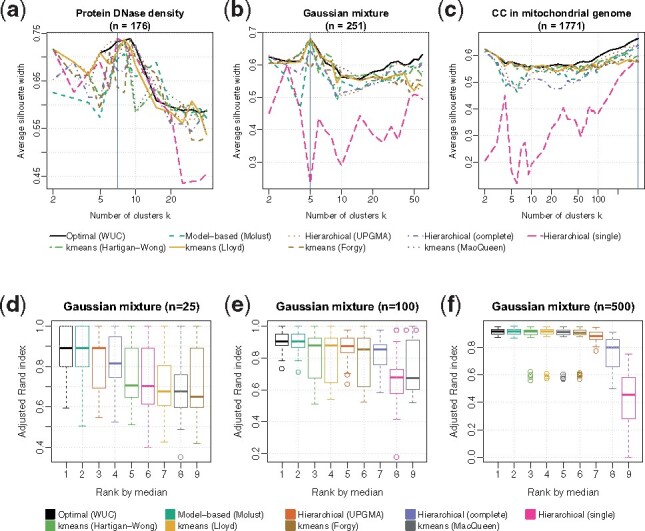
Optimal univariate clustering leads in silhouette cluster quality over heuristic clustering. Heuristic methods include model-based, heuristic *k*-means and hierarchical clustering in eight configurations. Cluster quality is measured by average silhouette width (ASW) and adjusted Rand index (ARI)—both the higher the better, as a function of number of clusters *k*. (**a**) Optical density of protein DNase (*n* = 176). (**b**) Simulated data (*n* = 251) from a five-component Gaussian mixture model. (**c**) Locations (*n* = 1771) of CC dinucleotide on the human mitochondrial genome. The blue vertical lines in (a–c) mark the maximum ASW in each plot. (**d–f**) ranked ARI of each method from the same Gaussian mixture model with (b), in different sample sizes of (d) 25, (e) 100 and (f) 500, each replicated 51 times to produce the box plots

WUC leads in both ASW and ARI among all methods (Fig. 1). In all datasets, WUC achieved the second highest, highest and highest ASW values on each dataset; the advantage becomes evident when either the sample size or the number of clusters is large. On the GMM data of three sample sizes, WUC attained the highest median ARI values in all cases. The empirical evidence here from diverse data and important cluster quality measures suggests that the WUC algorithm has the potential to replace mainstream heuristic clustering methods in the univariate case.

#### 3.1.3 Reproducibility, runtime and scalability

The reproducibility of WUC and heuristic *k*-means is compared on clustering CpG sites for the MT genome in Supplementary Note N1. WUC returned identical results in all four runs, while heuristic *k*-means clusters visibly deviated from the optimal solution in different ways. Despite the MT genome containing only 435 CpG sites—a small dataset, differences among the four heuristic runs are evident. Randomization in heuristic clustering to improve global optimality sacrificed reproducibility, while the deterministic WUC algorithm guarantees to reproduce.

On real datasets, WUC is scalable to solve large problems. We compare WUC with the Hartigan–Wong algorithm, the default option of kmeans(). It uses a greedy strategy to repeatedly update the cluster assignment of each point. The large dataset used is CpG sites along 25 human chromosomes (1–22, X, Y and MT). A CpG site is a genomic coordinate on a chromosome where a cytosine (C) is followed immediately by a phosphate (p) and a guanine (G). Their clusters are called CpG islands. On human reference genome version GRCh38, we performed four runs on each chromosome at *k *=* *20: two runs with one restart and two runs with 20 restarts for heuristic *k*-means. From results in Supplementary Note N1, heuristic *k*-means can finish faster than WUC but with high relative errors greater than 100%; when nstart was set to 20, relative errors were greatly reduced but it is ten times slower than WUC on an input of 2 500 000 points.

Next, we compare the runtime of the methods as functions of sample size *n* and number of clusters *k*. We include the quadratic (Wang and Song, 2011), log-linear and linear time solutions for WUC, all implemented in package ‘Ckmeans.1d.dp’ and the Hartigan–Wong’s *k*-means algorithm. Supplementary Figure N1.7 in Supplementary Note N1 compares the runtime of the methods as a function of *n* averaged over multiple runs at fixed *k *=* *5. At n<300, the log-linear solution is fastest due to its low overhead. At 500<n<3000, all three methods used comparable time. As *n* increases beyond 5000, the linear solution ran stably faster than log-linear or heuristic solutions.

### 3.2 Dysregulated genomic zones in human cancers

To study how cancer may have reshaped gene expression landscapes along chromosomes, we mapped genomic zones by clustering genes weighed by expression levels for 17 major human cancer types. The input data include 1390 RNA-seq transcriptome profiles on matched tumor-normal pairs for 17 cancer types downloaded from National Cancer Institute (NCI) Genomic Data Common (GDC) (Grossman *et al.*, 2016). Table 1 lists the 17 cancer types, their abbreviations, numbers of zones and numbers of statistically significantly polarized zones for each cancer type. We call a zone positively/negatively polarized if the number of upregulated genes is greater/fewer than the number of downregulated genes inside the zone; otherwise, the zone has no regulation polarity. Among the 3615–3984 zones for each cancer type, the number of significantly positively polarized zones ranges from 206 (5%) to 1126 (31%) and the number of significantly negatively polarized zones varies from 237 (6%) to 1218 (34%). The statistical significance of disproportion is determined by Pearson’s chi-squared test. The total numbers of outstanding genomic zones in every cancer type are higher than those expected by chance by permutation tests (Supplementary Note N2.1). Genomic zones and associated statistics for all 17 cancer types are given in Supplementary Table S1. We examined the robustness of zone boundaries along chromosome 12, of an average chromosomal length, on BRCA, COAD and ESCA of maximum, median and minimum sample sizes, respectively. By bootstrapping, numbers of zones varied only slightly by one to four and zone boundaries are about 88, 87 and 70% identical to those estimated using original samples (Supplementary Note N2.2). This suggests that zone boundaries are robust to the sample sizes used in this study.

**Table 1.** Statistics of regulation zones and their polarization in 17 human cancer types

Cancer study (project ID)	Sample size	#Zones	Outstanding	Positive+	Negative-
Bladder Urothelial Carcinoma (BLCA)	19×2	3752	1229	544	685
Breast Invasive Carcinoma (BRCA)	112×2	3615	2344	1126	1218
Cholangiocarcinoma (CHOL)	9×2	3984	1203	600	603
Colon Adenocarcinoma (COAD)	40×2	3733	1929	968	961
Esophageal Carcinoma (ESCA)	8×2	3708	443	206	237
Head and Neck Squamous Cell Carcinoma (HNSC)	43×2	3618	1666	852	814
Kidney Chromophobe (KICH)	23×2	3804	1994	947	1047
Kidney Renal Clear Cell Carcinoma (KIRC)	72×2	3699	2293	1098	1195
Kidney Renal Papillary Cell Carcinoma (KIRP)	31×2	3744	1763	801	962
Liver Hepatocellular Carcinoma (LIHC)	50×2	3656	1803	854	949
Lung Adenocarcinoma (LUAD)	57×2	3683	2076	1017	1059
Lung Squamous Cell Carcinoma (LUSC)	49×2	3730	2181	1064	1117
Prostate Adenocarcinoma (PRAD)	52×2	3663	1915	866	1049
Rectum Adenocarcinoma (READ)	9×2	3905	901	422	479
Stomach Adenocarcinoma (STAD)	27×2	3660	1228	585	643
Thyroid Carcinoma (THCA)	58×2	3681	2034	968	1066
Uterine Corpus Endometrial Carcinoma (UCEC)	23×2	3731	1490	709	781

*Note*: Numbers of significantly positively (+) and negatively (-) regulated zones are based on *P*-values (≤0.05) corrected for multiple testing by the Benjamini–Hochberg method.

Of the same 17 cancer types, 1266 copy number alteration profiles of matched tumor-normal samples were also used to map polarization in SCNA over zone boundaries derived above for each cancer type. Positive and negative polarities in SCNA within a zone are defined by dominance in the proportion of amplified/deleted copies of a genomic zone.

Zone boundaries are obtained by univariate clustering weighted by pooled tumor and normal samples to capture gene neighborhoods in both cancer and normal tissues. Zone polarity calling used numbers of differentially expressed genes, not sums of gene expression, in a zone. It contrasts proportions of up- and downregulated genes within versus outside a zone, robust to outliers of highly differentially expressed genes in the zone. Significantly polarized zones represent outstanding dysregulation or SCNA patterns not expected by chance. Human cancer genome polarization maps of both dysregulation and SCNA are shown by chromosome (Supplementary Fig. S1) and by cancer type (Supplementary Fig. S2).

By comparing outstanding genomic zones of each human cancer type and known regulatory signals in public repositories including ENCODE, we find that cancer-related genetic and epigenetic events are enriched in the outstanding zones (Supplementary Note N2.3). For example, Pol2 binding sites of breast cancer cell line MCF-7 are enriched in positively polarized zones of BRCA. The H3K4me3 epigenetic modification is enriched in outstanding genomic zones of all cancer types. Such evidence provides support to the biological relevance of the outstanding zones.

#### 3.2.1 Conserved dysregulation loci across cancer types

**Fig. 2.**
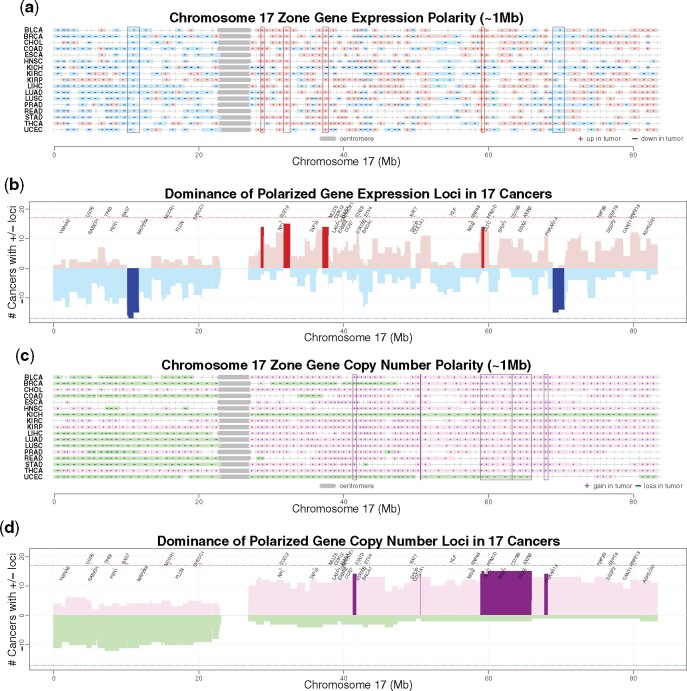
Maps of significant polarization in regulation and somatic copy number alteration (SCNA) along chromosome 17 across cancer types. (**a**) 17p is dominated by negative (-, blue) polarity in regulation, but 17q is more dominated by positive polarity (+, red) in regulation. Six loci surrounded by boxes are conserved in zone regulation polarity across cancer types. (**b**) Numbers of cancer types either positively (red) or negatively (blue) polarized in regulation along chromosomal loci. Loci are defined by intersections among zones. Dark red/blue bars indicate 14 (80%) or more of cancer types being identically polarized at a locus. Known cancer genes are marked along chromosomes. (**c**) Statistically significant zone polarization in SCNA along the chromosome across cancer types, corresponding to amplification (+, purple) and deletion (-, green), respectively. (**d**) Numbers of cancer types either positively (purple) or negatively (green) polarized in SCNA at loci along the chromosome. Dark purple/green bars indicate over 80% of cancer types are positively/negatively polarized in SCNA at a locus

The human cancer dysregulation maps suggest that regulation polarity is highly conserved at many genomic loci across cancer types. Figure 2 and Supplementary Figure S1 show polarization maps of the 17 cancer types by chromosome. Curated cancer genes and loci from COSMIC Cancer Gene Census (CGC) v87 (Futreal *et al.*, 2004) are marked along chromosomes. As zone boundaries vary by cancer type, we define loci as zone intersections across cancer types. Some loci maintain a conserved polarity across cancer types, presenting visible vertical patterning (Fig. 2a and Supplementary Figs S1.1a–S1.24a). Strong conservation of dysregulation polarity across cancer types is found at 109 loci over all chromosomes except 21, 22 and Y, as summarized in Supplementary Table S2. A locus is declared conserved if it shares the same regulation polarity across 14 or more (>80%) cancer types. Via a permutation test, the *P*-value of having more than the observed conserved loci is no more than 0.001 (Supplementary Note N2.4), suggesting these loci are statistically significant.

A total of 59 out of the 109 loci are downregulated among >80% cancer types. In Figure 2a, b, the chr17:68.87–70.47 Mb locus is negatively (-) polarized in regulation in 15 cancer types excluding ESCA or THCA, despite genomic amplification (+) at this locus in 13 cancer types (Fig. 2c and d). The locus at chr17:10.12–11.74 Mb is consistently downregulated (-) in all cancer types despite somatic copy number gain (+) in CHOL, KIRC, KIRP and THCA (Fig. 2a and c). Cancer fusion gene *GAS7* is located at this locus (Fig. 2b and d), which suppresses tumor cell migration in lung cancer (Tseng *et al.*, 2015). At locus chr2:166.05–167.46 Mb, regulation is negatively polarized (-) in all cancer types except insignificant in ESCA, but SCNA polarization at the same locus is positive, negative and insignificant in 10 (+), 3 (-) and 4 cancer types, respectively (Supplementary Fig. S1.2). The locus of chr15:95.26–97.03 Mb, toward the q-arm telomere of chr15, has significantly more genes downregulated than upregulated in all cancer types, while the SCNA polarity of this locus across cancers is mixed (4 amplified, 8 deleted and 5 insignificant) (Supplementary Fig. S1.15).

The other 50 loci are upregulated among >80% cancer types. One upregulated (+) and amplified (+) locus chr7:102.17–102.59 Mb (Supplementary Fig. S1.7) overlaps haploinsufficient gene *CUX1*, CUT-like homeobox 1, known to be both oncogenic and tumor suppressing (CGC v87). Although underexpressed CUX1 promotes tumor development, overexpression of CUX1 is associated with advanced cancers (Ramdzan and Nepveu, 2014). At locus chr14:18.24–20.57 Mb (Supplementary Fig. S1.14), next to the centromere at the beginning of 14q, genes are mostly upregulated (+) in all cancer types except BRCA, while SCNA polarity splits between being positive and negative at this locus. Locus chr17:31.69–32.58 Mb is also both upregulated (+) and amplified (+) (Fig. 2). This locus contains gene *SUZ12*, suppressor of zeste 12 homolog (*Drosophila*). It is found to be frequently overexpressed in colorectal cancer (Liu *et al.*, 2015), non-small cell lung cancer (Liu *et al.*, 2014), ovarian cancer (Li *et al.*, 2012) and tongue squamous cell carcinoma (Hu *et al.*, 2017).

Somatic alteration at genomic loci is less consistent than regulation polarity across cancer types. The SCNA polarity at a locus often oscillates between being positive and negative across cancer types. In contrast to loci of conserved regulation polarity being found on 21 chromosomes (Supplementary Table S2), conservation (>80% cancer types) in SCNA zone polarity is found at loci located on only nine chromosomes (1, 4–8, 12, 17 and 20) (Supplementary Fig. S1). SCNA loci with the same polarization sign among >80% cancer types are heavily populated on chromosome 1q (+) (Supplementary Fig. S1.1), 7 (+) (Supplementary Fig. S1.7), 8q (+) (Supplementary Fig. S1.8) and 20 (+) (Supplementary Fig. S1.20). This suggests that conservation of regulation polarization at these loci is a strong pan-cancer characteristic, largely independent of SCNA polarization.

#### 3.2.2 Regulation polarity along chromosomes within cancer type

Polarization in regulation zone displays continuity along chromosomes as visible horizontal patterning within each cancer type. Dysregulation and SCNA polarity maps are shown in Supplementary Figure S2 by cancer type. Zones with the same polarity can stretch from several million to tens of millions base pairs. In BRCA, chromosome arms 1q, 6p, 8q, 16p, 19q and 20q present the strongest continuity in positive (+) regulation polarity, while 6q, 8p, 16q and 17p are most continuous in negative (-) regulation polarity (Supplementary Fig. S2.2a). In HNSC (Supplementary Fig. S2.6), we observe continuous positive (+) polarization of zones along 3q, 7p, 20 and 22q, and negative (-) polarization of zones along 3p, 10, 17p, 19 and Y. We thus reproduced a previous finding of underexpression of 3p and overexpression of 22q in HNSC (Masayesva *et al.*, 2004). In COAD (Supplementary Fig. S2.4), positively (+) polarized zones dominate 7, 8q, 12q, 13q, 16q and 20; negatively (-) polarized zones dominate 1p, 4, 8p, 15q, 17p and 18q. In READ (Supplementary Fig. S2.14), positively (+) polarized zones are mostly found on 7, 8q, 12q, 17q and 20q; negatively (-) polarized zones on 1p, 4, 5q, 8p, 14q, 15q, 17p and 18q. This is consistent with previous findings on colorectal cancer having overexpressed (+) 7p, 8q, 13q, 20q and underexpressed (-) 1p, 4, 5q, 8p, 14q, 15q and 18 (Tsafrir *et al.*, 2006).

Horizontal continuity in zone polarity of SCNA along chromosomes is prominent. Somatic deletion (-) and amplification (+) often extend to a chromosomal arm or even an entire chromosome. In BRCA (Supplementary Fig. S2.2b), continuous DNA gain dominates 1q, 3q, 5p, 6p, 7, 8q, 10p, 12p, 16p, 19q, 20 and 21q; continuous DNA loss dominates 1p, 2q, 3p, 4, 6q, 8p, 9, 10q, 13q, 14q, 15q, 16q, 17p, 18q, 22q and Xq. Such a strong continuity suggests that SCNA occurs at a more global scale than modification in gene expression programs in cancer.

Although we observe a weak positive correlation in horizontal polarization continuity between dysregulation and SCNA, horizontal continuity is not always transcribed from SCNA to dysregulation. In BRCA (Supplementary Fig. S2.2a, b) only six out of 12 positive SCNA arms exhibited continuity in upregulation, and only 4 out of 16 negative SCNA arms exhibited continuity in downregulation.

Occasionally, horizontal zone polarization patterns in dysregulation and SCNA can phenomenally mismatch along chromosomes. In BRCA (Supplementary Fig. S2.2), 1p is consistently negatively (-) polarized in SCNA, but a long stretch of 1p between 25 and 50 Mb is positively (+) polarized in regulation. The region of chr7:80–90 Mb is positively (+) polarized in SCNA but negatively (-) polarized in regulation. These are examples where horizontal polarization continuity in regulation cannot be explained by the corresponding pattern in SCNA, suggesting horizontal continuity in regulation can be independent of SCNA.

#### 3.2.3 Top dysregulated zones are often specific to cancer type

Most significantly dysregulated zones are specific to cancer type. A top dysregulated zone may be either consistent with or opposite to the polarity of SCNA of the zone. Some such top zones are discussed for their cancer relevance in Supplementary Note N3. The top five dysregulated zones of each cancer type are visualized in Supplementary Figure S3. Complementary to the pan-cancer conservation of regulation polarity loci, unique patterns within top genomic zones can characterize a cancer type. These zones could be potential biomarkers for specific cancer types.

#### 3.2.4 Copy number alteration zone maps are highly consistent with known cancer aneuploidy

**Fig. 3.**
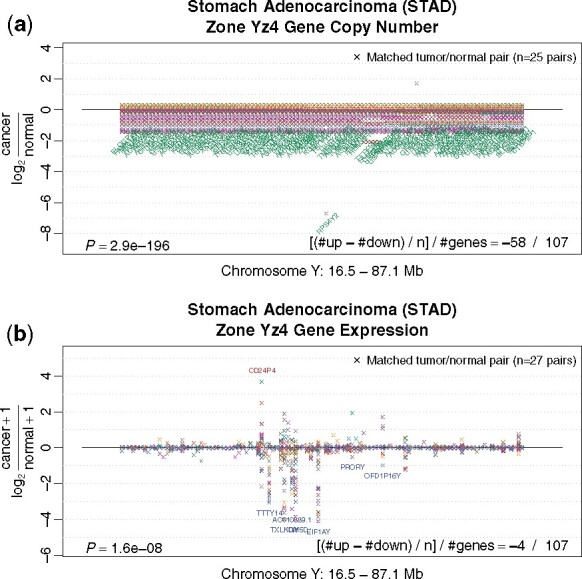
The top polarized zone in somatic copy number alteration in stomach adenocarcinoma (STAD). (**a**) Log ratios of SCNA of all genes in STAD tumor over matched normal stomach tissue in the most significantly polarized SCNA zone in STAD. (**b**) Log ratios of gene expression in STAD tumor over matched normal stomach tissue of the same zone. Genes are ordered by genomic coordinate but equal-space positioned

To evaluate the effectiveness of chromosomal clustering, we compare SCNA zone polarization with known cancer genome instability. SCNA polarization maps of all 17 cancer types are shown in Supplementary Figures S1 and S2. Most significantly polarized zones for each cancer type are displayed in Supplementary Note N4. With only one exception, they strongly agree with known SCNA in cancer, despite the fact that only up to 100 or so pairs of matched tumor normal tissues were used for each cancer type. For example, in stomach adenocarcinoma (STAD), the most polarized zone chrY:16.5–87.1 Mb (Fig. 3) covers almost the entire long arm of Yq, overlapping Yq11.22, Yq11.23 and Yq12. This zone is among the most heavily negatively polarized, consistent with previous findings that deletion on Y-chromosome is the most prominent cytogenetic band abnormality in gastric cancer (Ochi *et al.*, 1986). Except the top SCNA zones of ESCA identified with a small sample size (*n *=* *8 × 2), all top SCNA zones of other 16 cancer types coincide with frequent genome aberrations known for each respective cancer type (Supplementary Note N4). Therefore, these findings support the effectiveness of our methodology in detecting polarized genomic zones. It also suggests that a moderate number of matched tumor-normal pairs can reproduce genome instability findings from previous large-scale cancer genome studies mostly not using matched normal samples.

## 4 Discussion

Chromosomes contain domains of gene expression (Cohen *et al.*, 2000). Genes along the chromosome are clustered by expression and by function (Birnbaum *et al.*, 2003; Williams and Bowles, 2004), with as many as 20 genes in each cluster in plants (Williams and Bowles, 2004). Our study delineated conserved pan-cancer gene regulation loci and also cancer-specific gene regulation zones, identifying a chromatin impact not explainable by a single-gene regulatory mechanism. Several chromatin-level mechanisms offer possible causes of regulatory zoning. Enhancers (Quintero-Cadena and Sternberg, 2016) shared by genes in a zone could be utilized differentially in cancer and normal tissues, leading to polarization in zone regulation. Insulated genomic neighborhoods are needed for normal gene activation and repression (Hnisz *et al.*, 2016). An insulated neighborhood can be disrupted in cancer directly due to the loss of CTCF binding (Bradner *et al.*, 2017). In IDH mutant gliomas, this loss is a consequence of CTCF anchor site methylation (Flavahan *et al.*, 2016). The distribution of zone size is consistent with basic units for chromatin organization: insulated neighborhoods have a size between 25 and 940 kb (Hnisz *et al.*, 2016); TAD contains one or more insulated neighborhoods with a size of hundreds of kilobases (Dixon *et al.*, 2012); multi-TAD compartments A and B have a size of several megabases (Wang *et al.*, 2016).

Chromosomal clustering is different from segmentation. Segmentation looks for regions with events equal in magnitude; weighted clustering looks for regions with concentrated events. A segment can contain events scattered far apart but equal in magnitude; a compact cluster contains proximal events. Such differences are a consequence of their objective functions: segmentation penalizes the sum of squared differences in magnitude within a region; weighted clustering penalizes the weighted sum of squared distances between locations within a region. Indeed, spatial proximity between genomic elements is vital to gene regulation (Bonev and Cavalli, 2016).

Bellman (1973) first used dynamic programming to solve the problem of univariate clustering with general additive distance measures at a time complexity of $O(kn^{3})$. The R package ‘Ckmeans.1d.dp’ up to version 3.4.5 (Wang and Song, 2011) reduced the runtime to $O(kn^{2})$. The SMAWK algorithm enabled linear-time dynamic programming in histogram quantization (Wu and Rokne, 1989), scalar quantization (Wu and Zhang, 1993) and image thresholding (Luessi *et al.*, 2009). It is not popular most likely due to the runtime overhead of the original Reduce algorithm based on linked lists (Hershberger and Suri, 1997) or stacks (http://www.ics.uci.edu/~eppstein/PADS/SMAWK.py). Our search-space pruning algorithm Reduce-Min-In-Place addressed this issue, with implications on a broad range of problems where the SMAWK algorithm is applicable, far beyond weighted univariate clustering.

In summary, we have developed a chromosomal clustering method for delineating human cancer regulation zones along chromosomes. The method accelerates the characterization of activity over long sequences, such as genetic and epigenetic events in the order of hundreds of millions along chromosomes. This capacity to identify chromosomal patterns provides an avenue to reveal transcriptome organization anchored to the genome in the molecular biology of cancer and beyond.

## Funding

The work was partially supported by National Science Foundation [1661331 to M.S.], USDA [2016-51181-25408 to M.S.] and National Cancer Institute Partnership for the Advancement of Cancer Research NCI grants [U54 CA132383 (NMSU) and U54 CA132381 (Fred Hutch)].

*Conflict of Interest*: none declared.

## Supplementary Material

btaa613_Supplementary_Data

## References

[btaa613-B1] Achinger-Kawecka J. , Clark S.J. (2017) Disruption of the 3D cancer genome blueprint. Epigenomics, 9, 47–55. 27936932 10.2217/epi-2016-0111

[btaa613-B2] Aggarwal A. et al (1987) Geometric applications of a matrix-searching algorithm. Algorithmica, 2, 195–208.

[btaa613-B3] Bellman R. (1973) A note on cluster analysis and dynamic programming. Math. Biosci., 18, 311–312.

[btaa613-B4] Bicciato S. et al (2009) A computational procedure to identify significant overlap of differentially expressed and genomic imbalanced regions in cancer datasets. Nucleic Acids Res., 37, 5057–5070. 19542187 10.1093/nar/gkp520 PMC2731905

[btaa613-B5] Birnbaum K. et al (2003) A gene expression map of the Arabidopsis root. Science, 302, 1956–1960. 14671301 10.1126/science.1090022

[btaa613-B6] Bonev B. , Cavalli G. (2016) Organization and function of the 3D genome. Nat. Rev. Genet., 17, 661–678. 27739532 10.1038/nrg.2016.112

[btaa613-B7] Bradner J.E. et al (2017) Transcriptional addiction in cancer. Cell, 168, 629–643. 28187285 10.1016/j.cell.2016.12.013 PMC5308559

[btaa613-B8] Callegaro A. et al (2006) A locally adaptive statistical procedure (LAP) to identify differentially expressed chromosomal regions. Bioinformatics, 22, 2658–2666. 16951291 10.1093/bioinformatics/btl455

[btaa613-B9] Cohen B.A. et al (2000) A computational analysis of whole-genome expression data reveals chromosomal domains of gene expression. Nat. Genet., 26, 183–186. 11017073 10.1038/79896

[btaa613-B10] Colaprico A. et al (2016) TCGAbiolinks: an R/Bioconductor package for integrative analysis of TCGA data. Nucleic Acids Res., 44, e71. 26704973 10.1093/nar/gkv1507 PMC4856967

[btaa613-B11] Delatola E.I. et al (2017) SegCorr a statistical procedure for the detection of genomic regions of correlated expression. BMC Bioinformatics, 18, 333. 28697800 10.1186/s12859-017-1742-5 PMC5504623

[btaa613-B12] Dixon J.R. et al (2012) Topological domains in mammalian genomes identified by analysis of chromatin interactions. Nature, 485, 376–380. 22495300 10.1038/nature11082 PMC3356448

[btaa613-B13] Flavahan W.A. et al (2016) Insulator dysfunction and oncogene activation in IDH mutant gliomas. Nature, 529, 110–114. 26700815 10.1038/nature16490 PMC4831574

[btaa613-B14] Forgy E.W. (1965) Cluster analysis of multivariate data: efficiency versus interpretability of classifications. Biometrics, 21, 768–769.

[btaa613-B15] Futreal P.A. et al (2004) A census of human cancer genes. Nat. Rev. Cancer, 4, 177–183. 14993899 10.1038/nrc1299 PMC2665285

[btaa613-B16] Grossman R.L. et al (2016) Toward a shared vision for cancer genomic data. N. Engl. J. Med., 375, 1109–1112. 27653561 10.1056/NEJMp1607591 PMC6309165

[btaa613-B17] Hartigan J.A. , Wong M.A. (1979) Algorithm AS 136: a *k*-means clustering algorithm. J. R. Stat. Soc. Ser. C (Appl. Stat.*)*, 28, 100–108.

[btaa613-B18] Hershberger J. , Suri S. (1997) Matrix searching with the shortest-path metric. SIAM J. Comput., 26, 1612–1634.

[btaa613-B19] Hnisz D. et al (2016) Insulated neighborhoods: structural and functional units of mammalian gene control. Cell, 167, 1188–1200. 27863240 10.1016/j.cell.2016.10.024 PMC5125522

[btaa613-B20] Hu H. et al (2017) Overexpression of suppressor of zest 12 is associated with cervical node metastasis and unfavorable prognosis in tongue squamous cell carcinoma. Cancer Cell Int., 17, 26. 28228691 10.1186/s12935-017-0395-9 PMC5307854

[btaa613-B21] Hubert L. , Arabie P. (1985) Comparing partitions. J. Classif., 2, 193–218.

[btaa613-B22] Kaiser V.B. , Semple C.A. (2017) When TADs go bad: chromatin structure and nuclear organisation in human disease. F1000Research, 6, 314. 10.12688/f1000research.10792.1 PMC5373421 28408976

[btaa613-B23] Levesque M.J. , Raj A. (2013) Single-chromosome transcriptional profiling reveals chromosomal gene expression regulation. Nat. Methods, 10, 246–248. 23416756 10.1038/nmeth.2372 PMC4131260

[btaa613-B24] Li H. et al (2012) SUZ12 promotes human epithelial ovarian cancer by suppressing apoptosis via silencing HRK. Mol. Cancer Res., 10, 1462–1472. 22964433 10.1158/1541-7786.MCR-12-0335 PMC3501543

[btaa613-B25] Liotta L.A. et al (1991) Cancer metastasis and angiogenesis: an imbalance of positive and negative regulation. Cell, 64, 327–336. 1703045 10.1016/0092-8674(91)90642-c

[btaa613-B26] Liu C. et al (2014) SUZ12 is involved in progression of non-small cell lung cancer by promoting cell proliferation and metastasis. Tumour Biol., 35, 6073–6082. 24633887 10.1007/s13277-014-1804-5

[btaa613-B27] Liu Y.-L. et al (2015) Expression and clinicopathological significance of EED, SUZ12 and EZH2 mRNA in colorectal cancer. J. Cancer Res. Clin. Oncol., 141, 661–669. 25326896 10.1007/s00432-014-1854-5 PMC11823763

[btaa613-B28] Lloyd S. (1982) Least squares quantization in PCM. IEEE Trans. Inf. Theory, 28, 129–137.

[btaa613-B29] Luessi M. et al (2009) Framework for efficient optimal multilevel image thresholding. J. Electronic Imaging, 18, 013004.

[btaa613-B30] MacQueen J. (1967) Some methods for classification and analysis of multivariate observations. In *Proceedings of the Fifth Berkeley Symposium on Mathematical Statistics and Probability*, vol. 1. University of California Press, Berkeley, Calif, p. 281.

[btaa613-B31] Masayesva B.G. et al (2004) Gene expression alterations over large chromosomal regions in cancers include multiple genes unrelated to malignant progression. Proc. Natl. Acad. Sci. USA, 101, 8715–8720. 15155901 10.1073/pnas.0400027101 PMC423261

[btaa613-B32] Nilsson B. et al (2008) An improved method for detecting and delineating genomic regions with altered gene expression in cancer. Genome Biol., 9, R13. 18208590 10.1186/gb-2008-9-1-r13 PMC2395254

[btaa613-B33] Ochi H. et al (1986) Cytogenetic studies in primary gastric cancer. Cancer Genet. Cytogenet., 22, 295–307. 3731046 10.1016/0165-4608(86)90022-1

[btaa613-B34] Quintero-Cadena P. , Sternberg P.W. (2016) Enhancer sharing promotes neighborhoods of transcriptional regulation across eukaryotes. G3 Genes Genomes Genet., 6, 4167–4174. 10.1534/g3.116.036228 PMC5144984 27799341

[btaa613-B35] R Core Team. (2016) R: A Language and Environment for Statistical Computing. R Foundation for Statistical Computing, Vienna, Austria.

[btaa613-B36] Ramdzan Z.M. , Nepveu A. (2014) CUX1, a haploinsufficient tumour suppressor gene overexpressed in advanced cancers. Nat. Rev. Cancer, 14, 673–682. 25190083 10.1038/nrc3805

[btaa613-B37] Risso D. et al (2011) GC-content normalization for RNA-seq data. BMC Bioinformatics, 12, 480. 22177264 10.1186/1471-2105-12-480 PMC3315510

[btaa613-B38] Rousseeuw P.J. (1987) Silhouettes: a graphical aid to the interpretation and validation of cluster analysis. J. Comput. Appl. Math., 20, 53–65.

[btaa613-B39] Rubin A.F. , Green P. (2013) Expression-based segmentation of the Drosophila genome. BMC Genomics, 14, 812. 24256206 10.1186/1471-2164-14-812 PMC3909303

[btaa613-B40] Schwarzer W. et al (2017) Two independent modes of chromatin organization revealed by cohesin removal. Nature, 551, 51–56. 29094699 10.1038/nature24281 PMC5687303

[btaa613-B41] Scrucca L. et al (2016) mclust 5: clustering, classification and density estimation using Gaussian finite mixture models. R. J., 8, 289–233. 27818791 PMC5096736

[btaa613-B42] Taberlay P.C. et al (2016) Three-dimensional disorganization of the cancer genome occurs coincident with long-range genetic and epigenetic alterations. Genome Res., 26, 719–731. 27053337 10.1101/gr.201517.115 PMC4889976

[btaa613-B43] Toedling J. et al (2005) MACAT—microarray chromosome analysis tool. Bioinformatics, 21, 2112–2113. 15572464 10.1093/bioinformatics/bti183

[btaa613-B44] Tsafrir D. et al (2006) Relationship of gene expression and chromosomal abnormalities in colorectal cancer. Cancer Res., 66, 2129–2137. 16489013 10.1158/0008-5472.CAN-05-2569

[btaa613-B45] Tseng R.-C. et al (2015) Growth-arrest-specific 7C protein inhibits tumor metastasis via the N-WASP/FAK/F-actin and hnRNP U/beta-TrCP/beta-catenin pathways in lung cancer. Oncotarget, 6, 44207–44221. 26506240 10.18632/oncotarget.6229 PMC4792552

[btaa613-B46] Turkheimer F.E. et al (2006) Chromosomal patterns of gene expression from microarray data: methodology, validation and clinical relevance in gliomas. BMC Bioinformatics, 7, 526. 17140431 10.1186/1471-2105-7-526 PMC1698583

[btaa613-B47] Vogel J.H. et al (2005) Chromosomal clustering of a human transcriptome reveals regulatory background. BMC Bioinformatics, 6, 230. 16171528 10.1186/1471-2105-6-230 PMC1261156

[btaa613-B48] Volpe M. et al (2018) ClusterScan: simple and generalistic identification of genomic clusters. Bioinformatics, 34, 3921–3923. 29912285 10.1093/bioinformatics/bty486

[btaa613-B49] Wang H. , Song M. (2011) Ckmeans.1d.dp: optimal *k*-means clustering in one dimension by dynamic programming. R. J., 3, 29–33. 27942416 PMC5148156

[btaa613-B50] Wang H.-J. et al (2015a) Identification of aberrant chromosomal regions in human breast cancer using gene expression data and related gene information. Med. Sci. Monit., 21, 2557–2566. 26319982 10.12659/MSM.894887 PMC4557392

[btaa613-B51] Wang Q. et al (2015b) Heterogeneous dna methylation contributes to tumorigenesis through inducing the loss of coexpression connectivity in colorectal cancer. Genes Chromosomes Cancer, 54, 110–121. 25407423 10.1002/gcc.22224 PMC4785867

[btaa613-B52] Wang S. et al (2016) Spatial organization of chromatin domains and compartments in single chromosomes. Science, 353, 598–602. 27445307 10.1126/science.aaf8084 PMC4991974

[btaa613-B53] Williams E.J. , Bowles D.J. (2004) Coexpression of neighboring genes in the genome of *Arabidopsis thaliana*. Genome Res., 14, 1060–1067. 15173112 10.1101/gr.2131104 PMC419784

[btaa613-B54] Woo H.G. et al (2017) Integrative analysis of genomic and epigenomic regulation of the transcriptome in liver cancer. Nat. Commun., 8, 839. 29018224 10.1038/s41467-017-00991-w PMC5635060

[btaa613-B55] Wu X. , Rokne J. (1989) An *O*(*KN* lg *N*) algorithm for optimum *K*-level quantization on histograms of *N* points. In *Proceedings of the 17th Conference on ACM Annual Computer Science Conference*. ACM, Louisville, Kentucky, pp. 339–343.

[btaa613-B56] Wu X. , Zhang K. (1993) Quantizer monotonicities and globally optimal scalar quantizer design. IEEE Trans. Inf. Theory, 39, 1049–1053.

