# Supplementary material for: Efficient weighted univariate clustering maps outstanding dysregulated genomic zones in human cancers
Source: Bioinformatics. 2020 Jul 3;36(20):5027–36. doi: 10.1093/bioinformatics/btaa613 (PMC7755420; doi:10.1093/bioinformatics/btaa613)
Supplement: btaa613_Supplementary_Data [file btaa613_supplementary_data.zip › SuppFig-S1.pdf]

Supplementary Figure S1: Maps of genomic zone showing polarity in gene regulation and somatic copy number alteration in 17 cancer types (organized by chromosome)

List of Figures

S1.1 Chromosome 1 . . . . . S1-2
S1.2 Chromosome 2 . . . . . S1-3
S1.3 Chromosome 3 . . . . . S1-4
S1.4 Chromosome 4 . . . . . S1-5
S1.5 Chromosome 5 . . . . . S1-6
S1.6 Chromosome 6 . . . . . S1-7
S1.7 Chromosome 7 . . . . . S1-8
S1.8 Chromosome 8 . . . . . S1-9
S1.9 Chromosome 9 . . . . . S1-10
S1.10 Chromosome 10 . . . . . S1-11
S1.11 Chromosome 11 . . . . . S1-12
S1.12 Chromosome 12 . . . . . S1-13
S1.13 Chromosome 13 . . . . . S1-14
S1.14 Chromosome 14 . . . . . S1-15
S1.15 Chromosome 15 . . . . . S1-16
S1.16 Chromosome 16 . . . . . S1-17
S1.17 Chromosome 17 . . . . . S1-18
S1.18 Chromosome 18 . . . . . S1-19
S1.19 Chromosome 19 . . . . . S1-20
S1.20 Chromosome 20 . . . . . S1-21
S1.21 Chromosome 21 . . . . . S1-22
S1.22 Chromosome 22 . . . . . S1-23
S1.23 Chromosome X S1-24
S1.24 Chromosome Y S1-25

Legend used in maps of genomic zone

a, The shown chromosome is marked by genomic zone boundaries and polarization in regulation. Horizontal patterns are formed by consecutive zones of the same polarity along chromosomes. Vertical patterns of loci with the same polarity in at least 14 cancer types out of 17 are marked by rectangles. Red ‘+’ and blue ‘-’ mark statistically significant positive and negative zone polarization, corresponding to dominance of up- and down-regulated genes, respectively.

b, Numbers of cancer type that are either positively or negatively polarized in regulation in loci along the chromosome. Loci are defined by intersections among zones across cancer types. Red/blue bars represent numbers of cancer type positively/negatively polarized at a locus. Dark red/blue bars indicate 14 (80%) or more of 17 cancer types being identically polarized at a locus. Among a total of 717 known cancer genes and six cancer loci (IGK, IGL, IGH, TRA, TRB, TRD) from COSMIC Cancer Gene Census (CGC) version 87, those located on the shown chromosome are marked by name and location.

c, Purple ‘+’ and green ‘-’ mark statistically significant zone polarization in somatic copy number alteration (SCNA) along the chromosome across 17 cancer types, corresponding to amplification and deletion, respectively.

d, Numbers of cancer type that are either positively (purple) or negatively (green) polarized in SCNA in loci along the chromosome. Dark purple/green bars indicate over 80% of cancer types are positively/negatively polarized in SCNA at a locus. Known cancer genes from CGC v87 located on this chromosome are marked by name and location.

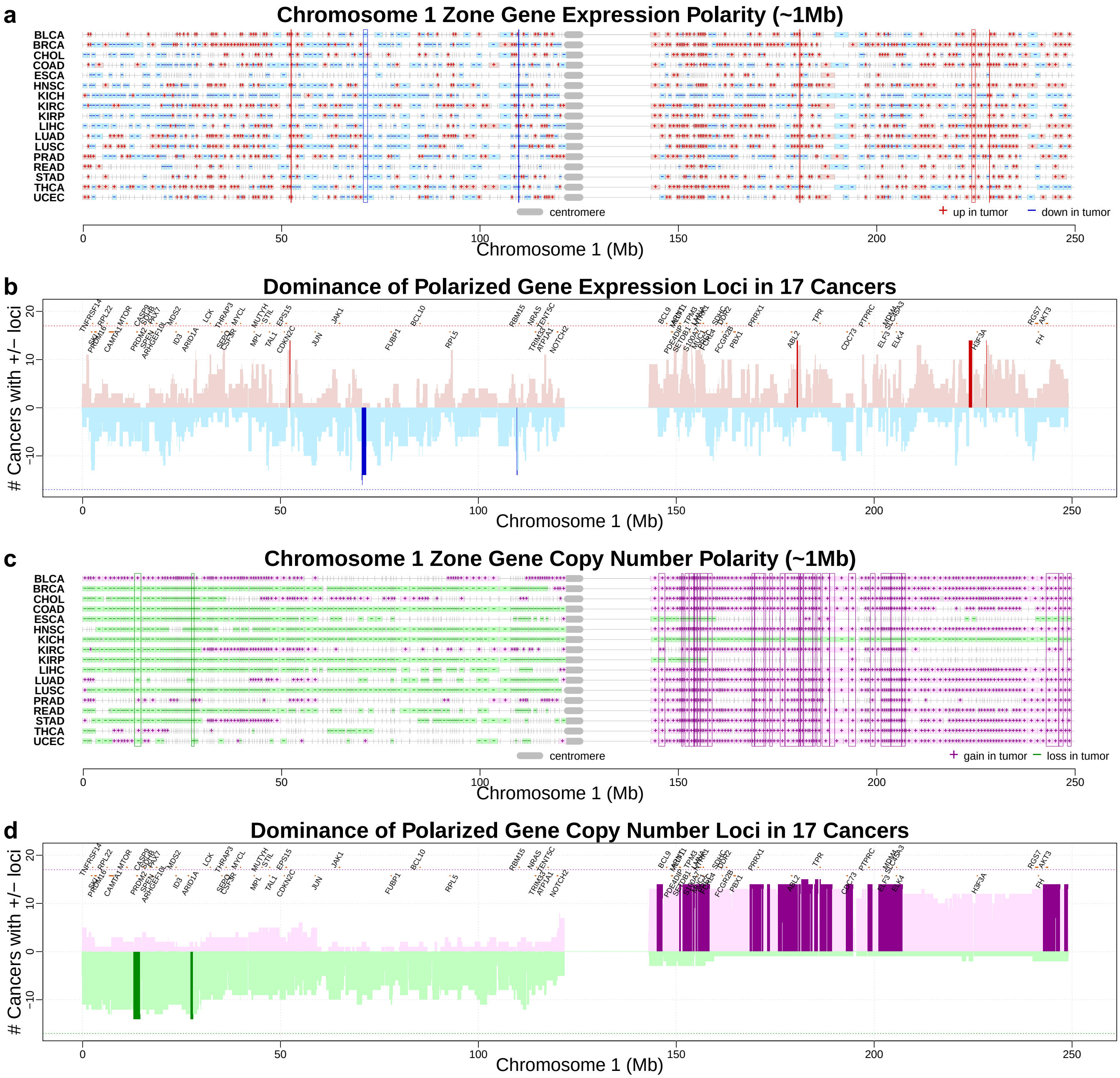

Supplementary Figure S1.1: Genomic zone maps along chromosome 1 in 17 cancer types versus their matched normal tissues. See the legend on page 1.

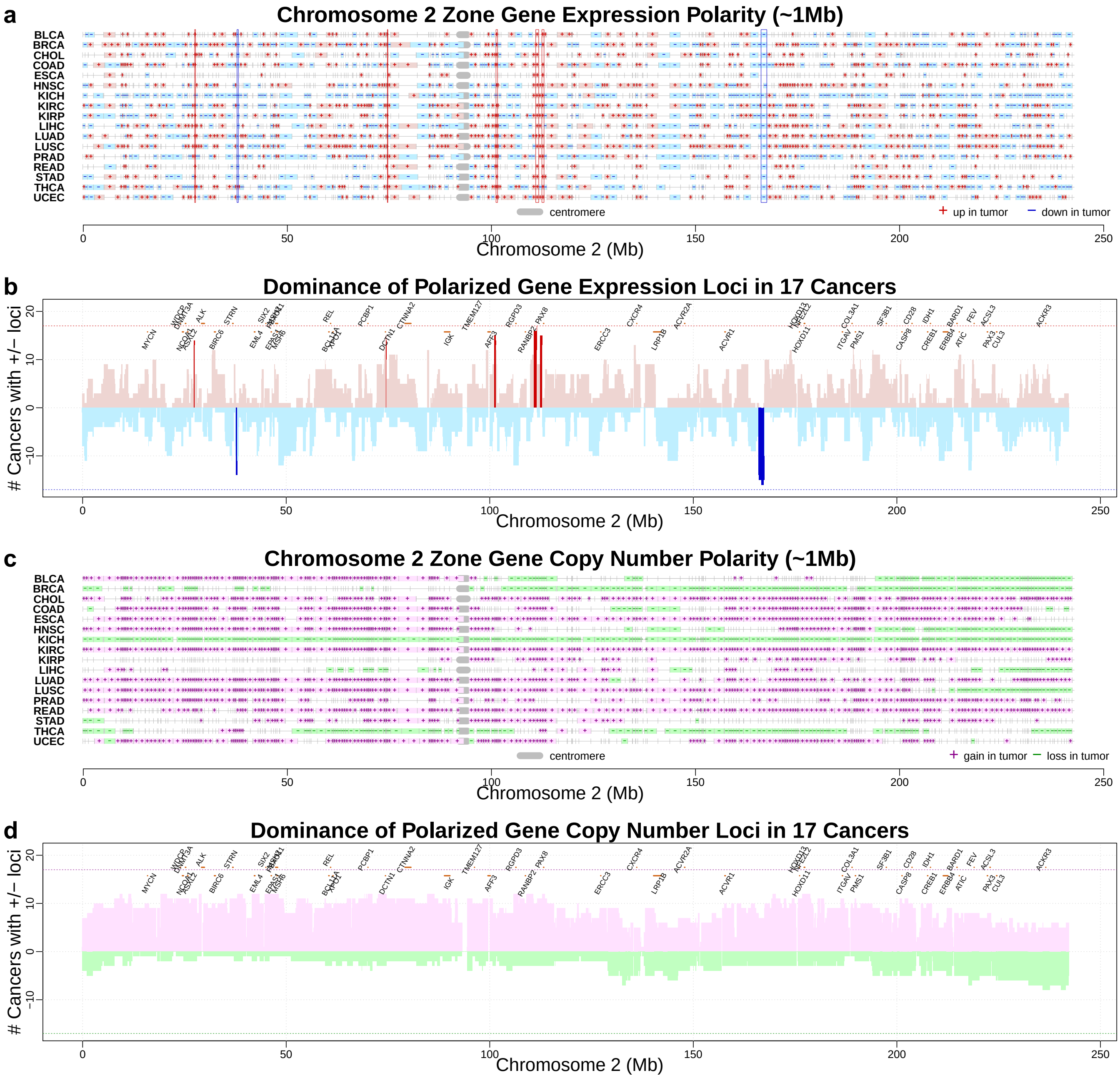

Supplementary Figure S1.2: Genomic zone maps along chromosome 2 in 17 cancer types versus their matched normal tissues. See the legend on page 1.

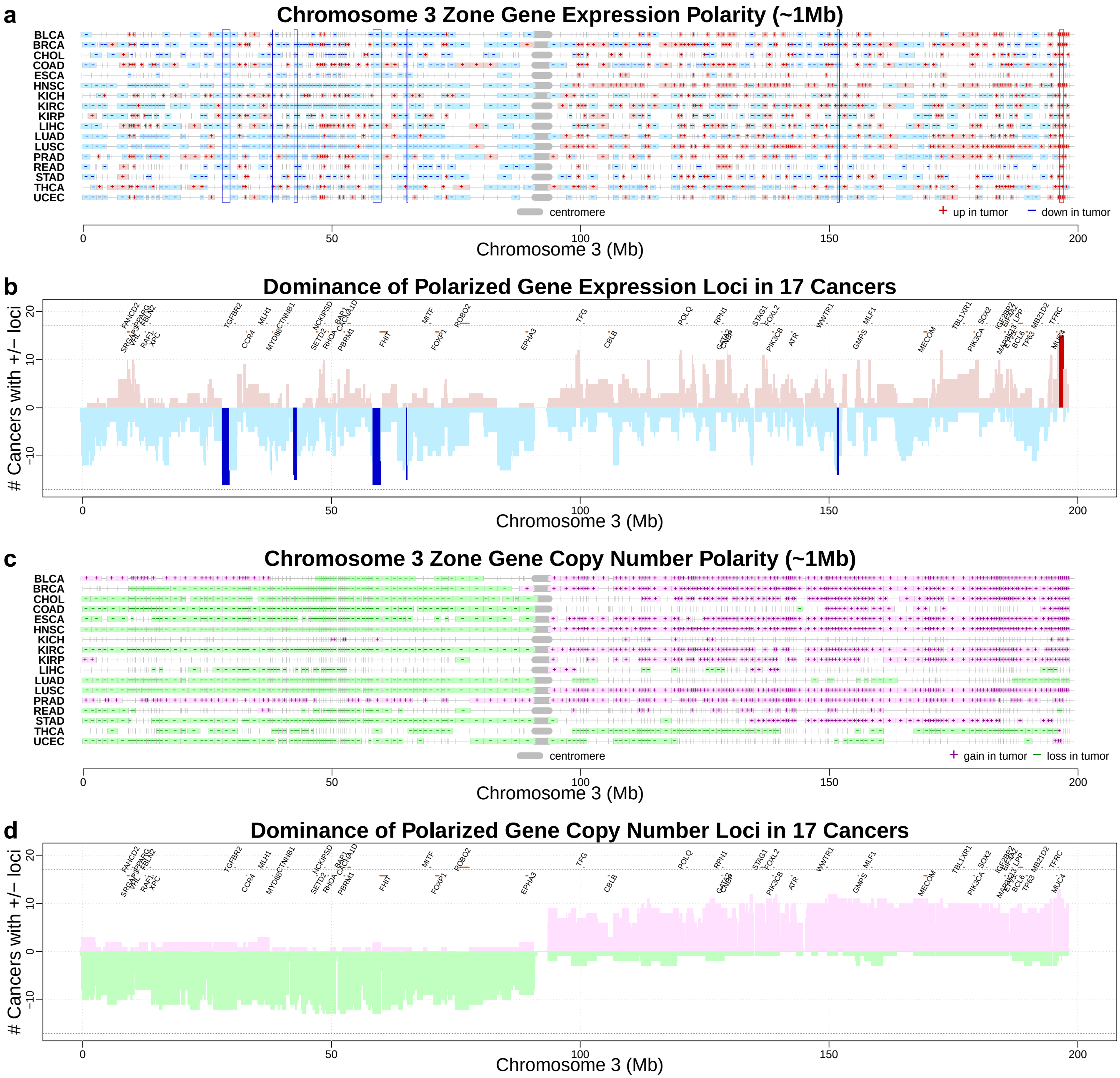

Supplementary Figure S1.3: Genomic zone maps along chromosome 3 in 17 cancer types versus their matched normal tissues. See the legend on page 1.

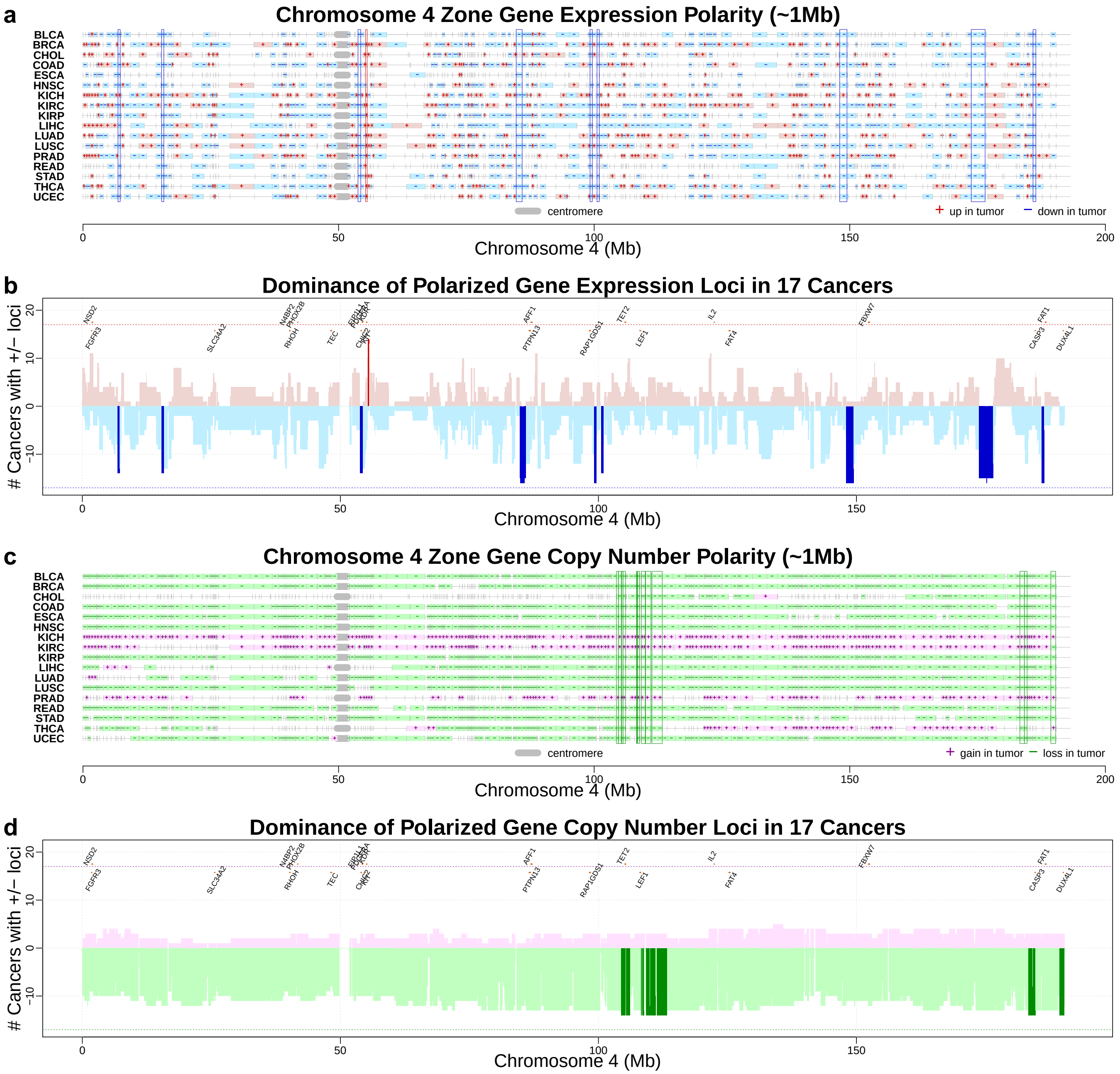

Supplementary Figure S1.4: Genomic zone maps along chromosome 4 in 17 cancer types versus their matched normal tissues. See the legend on page 1.

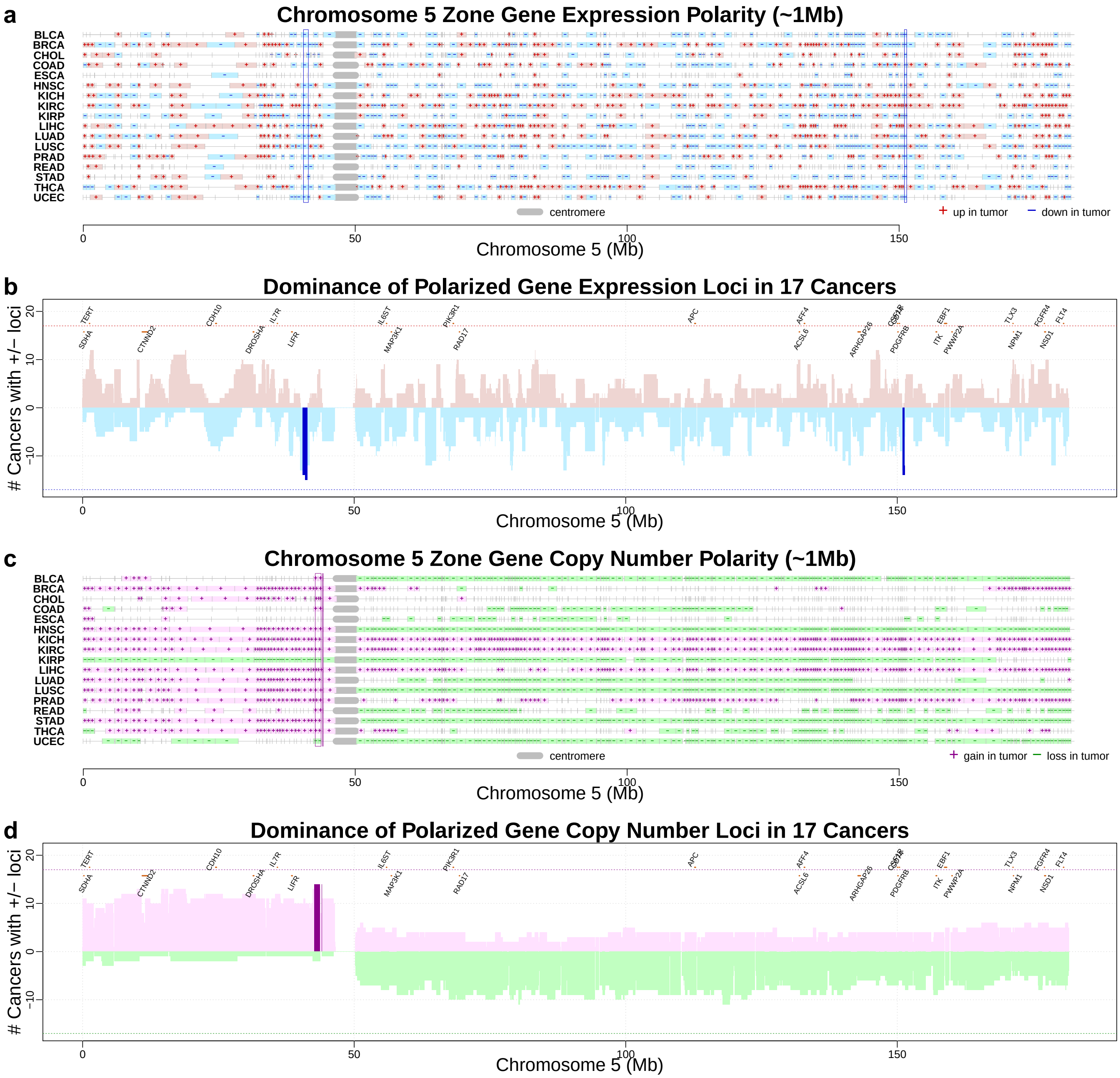

Supplementary Figure S1.5: Genomic zone maps along chromosome 5 in 17 cancer types versus their matched normal tissues. See the legend on page 1.

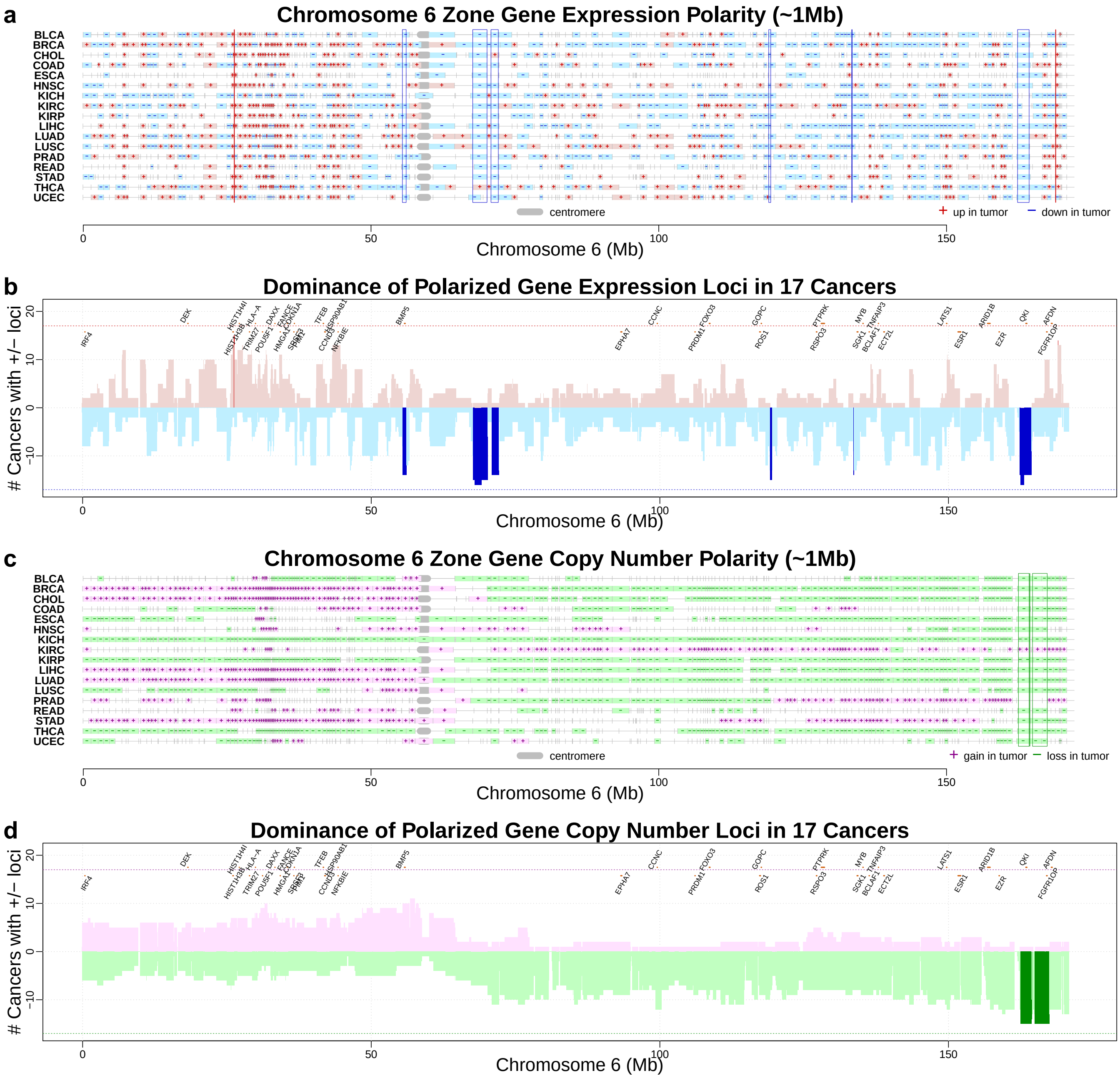

Supplementary Figure S1.6: Genomic zone maps along chromosome 6 in 17 cancer types versus their matched normal tissues. See the legend on page 1.

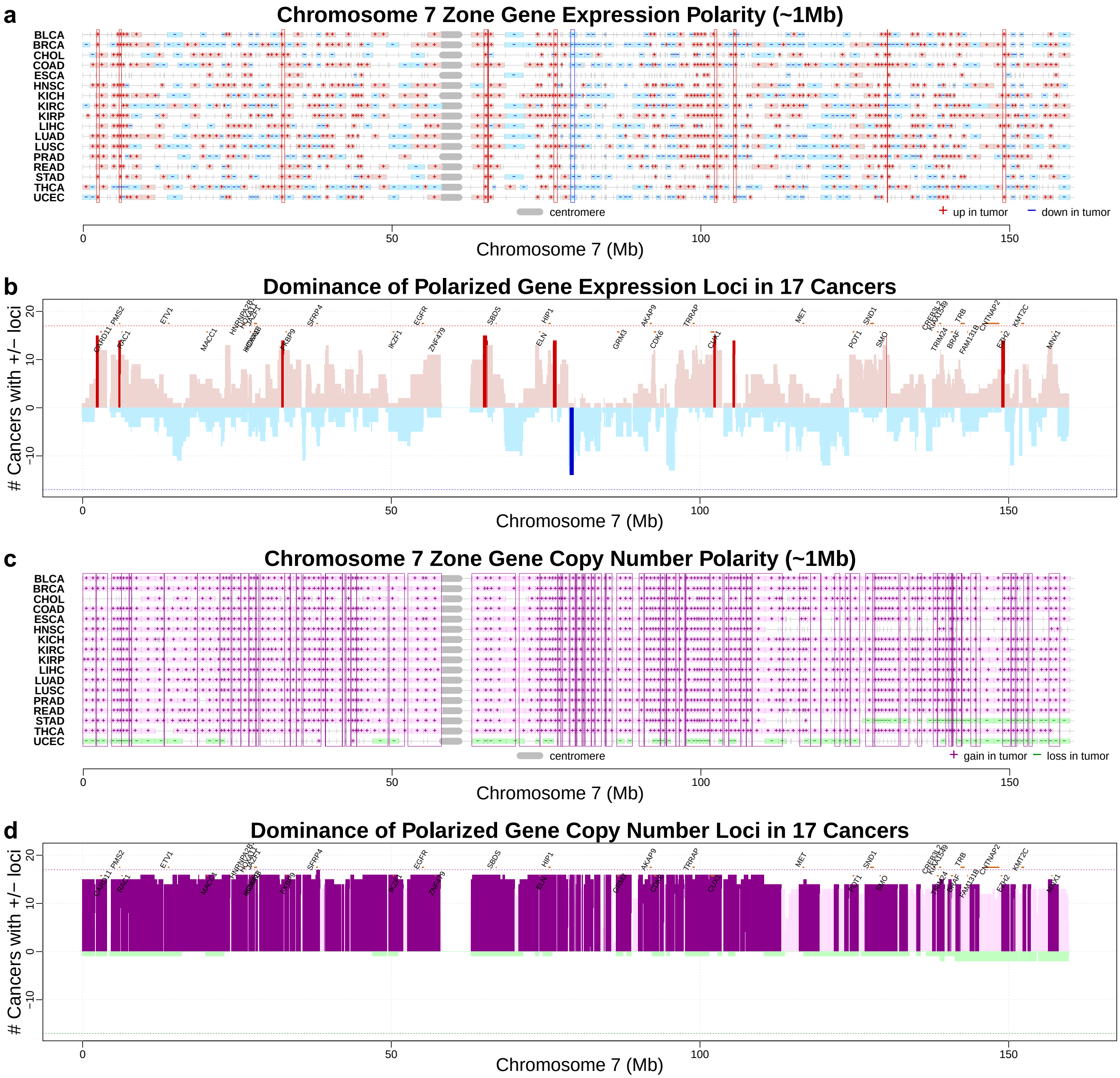

Supplementary Figure S1.7: Genomic zone maps along chromosome 7 in 17 cancer types versus their matched normal tissues. See the legend on page 1.

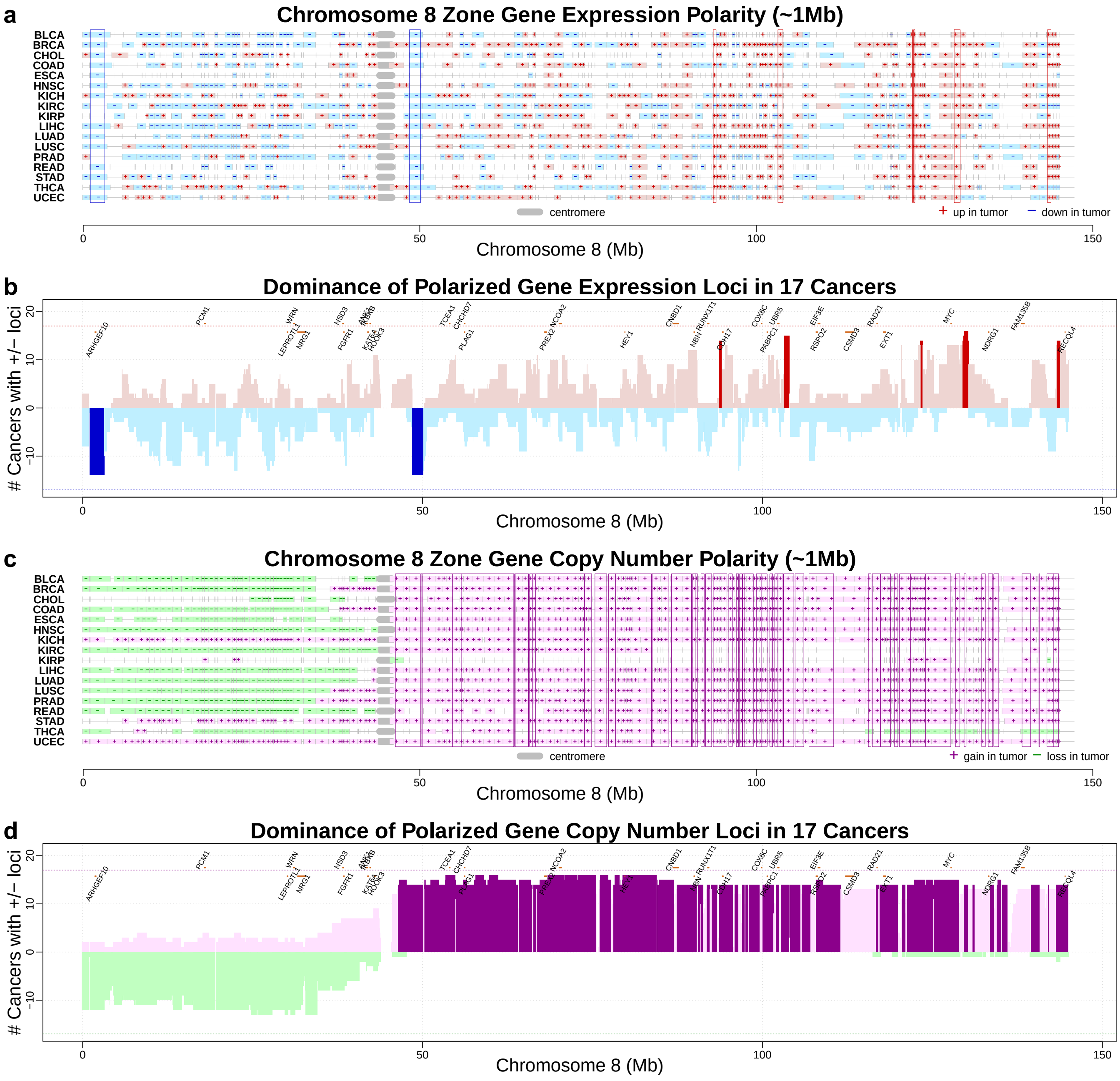

Supplementary Figure S1.8: Genomic zone maps along chromosome 8 in 17 cancer types versus their matched normal tissues. See the legend on page 1.

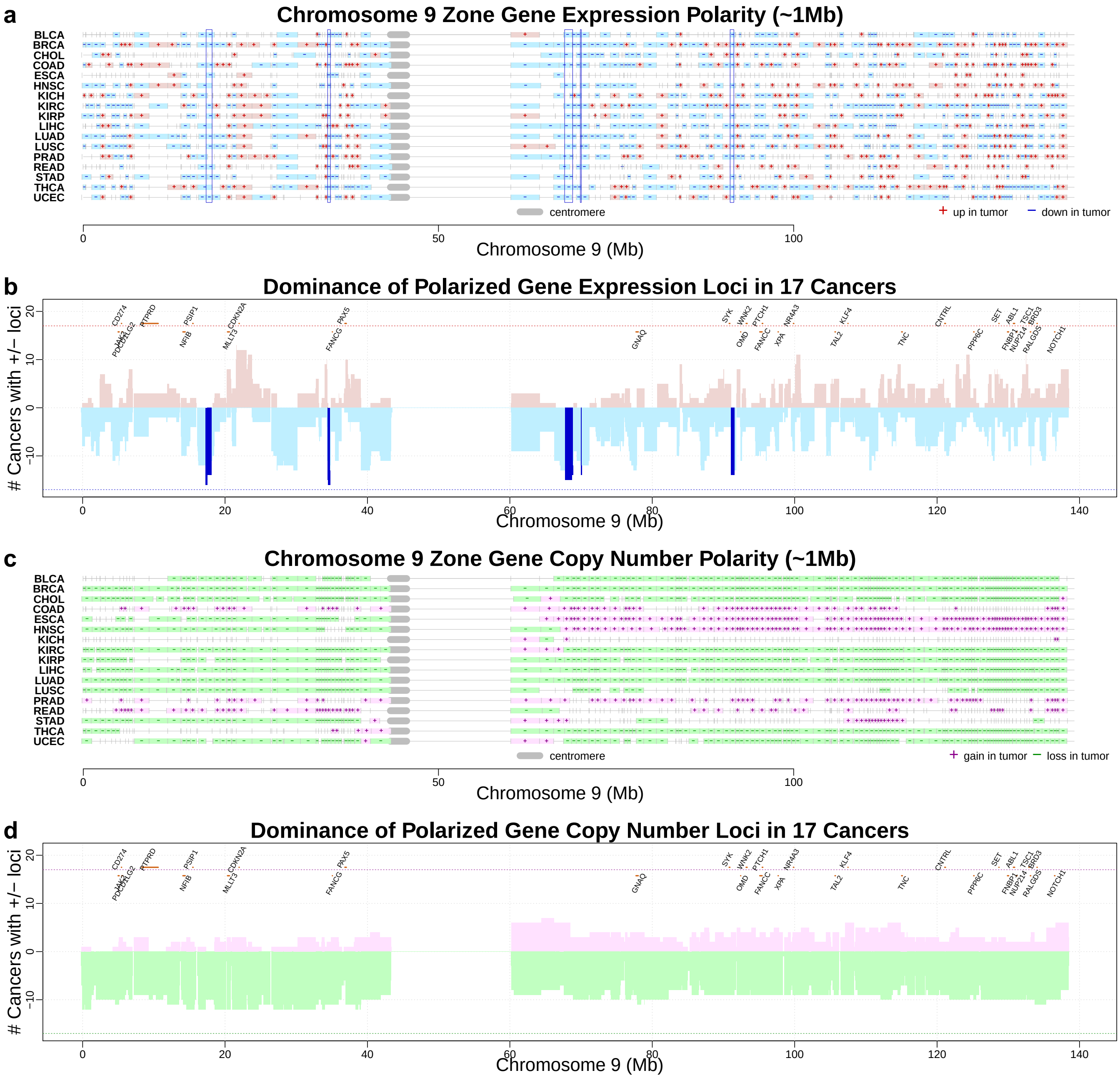

Supplementary Figure S1.9: Genomic zone maps along chromosome 9 in 17 cancer types versus their matched normal tissues. See the legend on page 1.

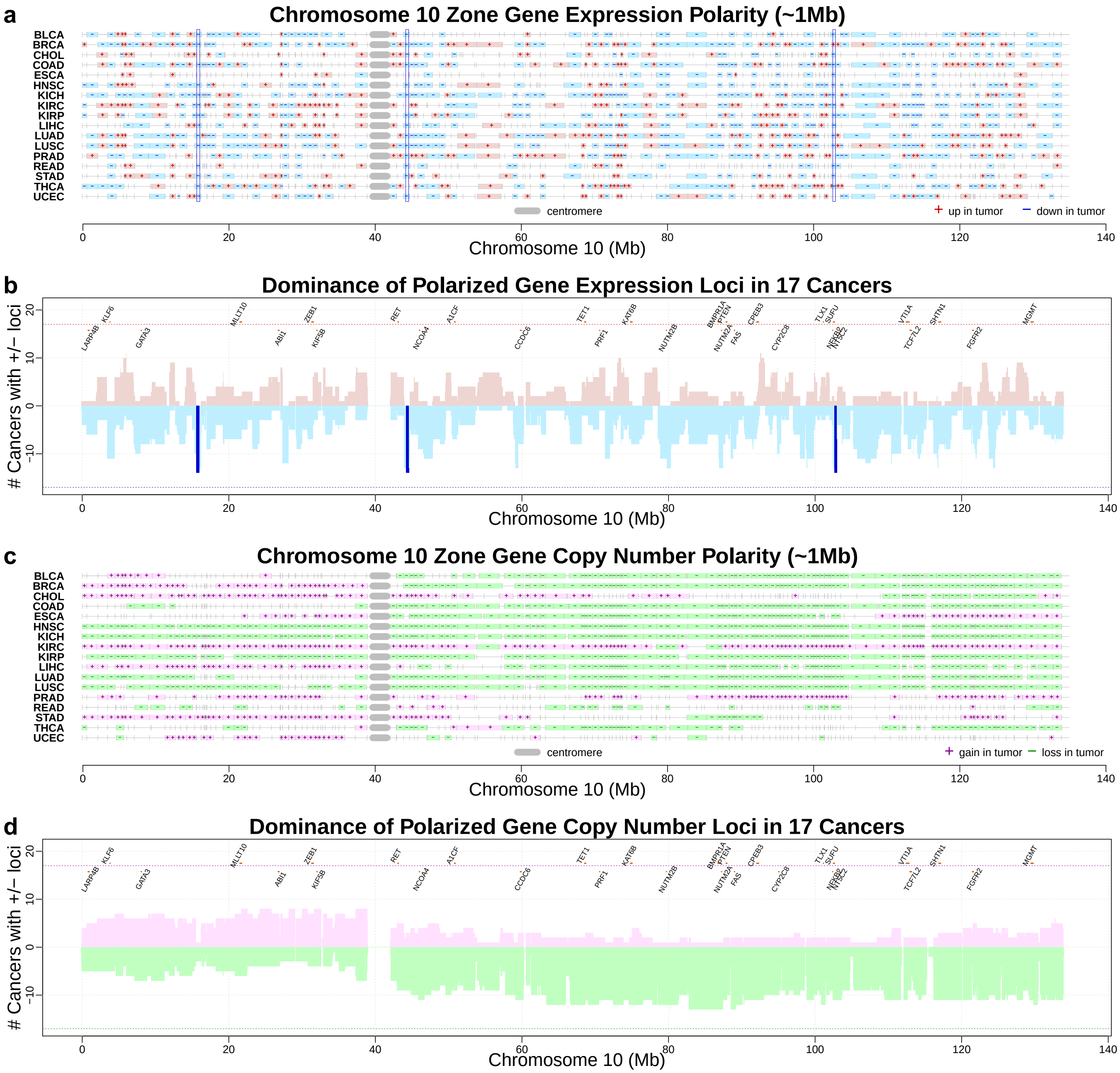

Supplementary Figure S1.10: Genomic zone maps along chromosome 10 in 17 cancer types versus their matched normal tissues. See the legend on page 1.

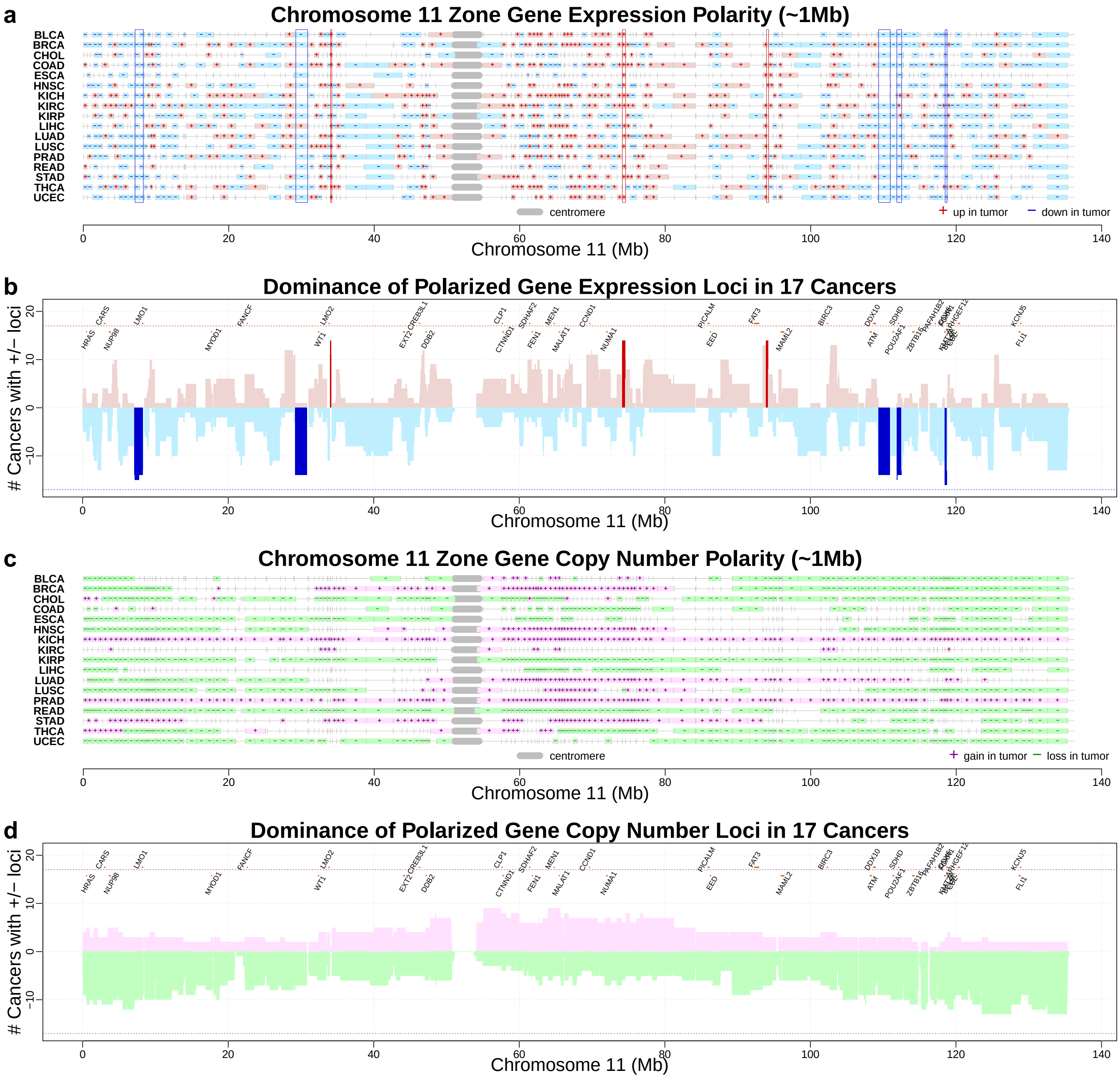

Supplementary Figure S1.11: Genomic zone maps along chromosome 11 in 17 cancer types versus their matched normal tissues. See the legend on page 1.

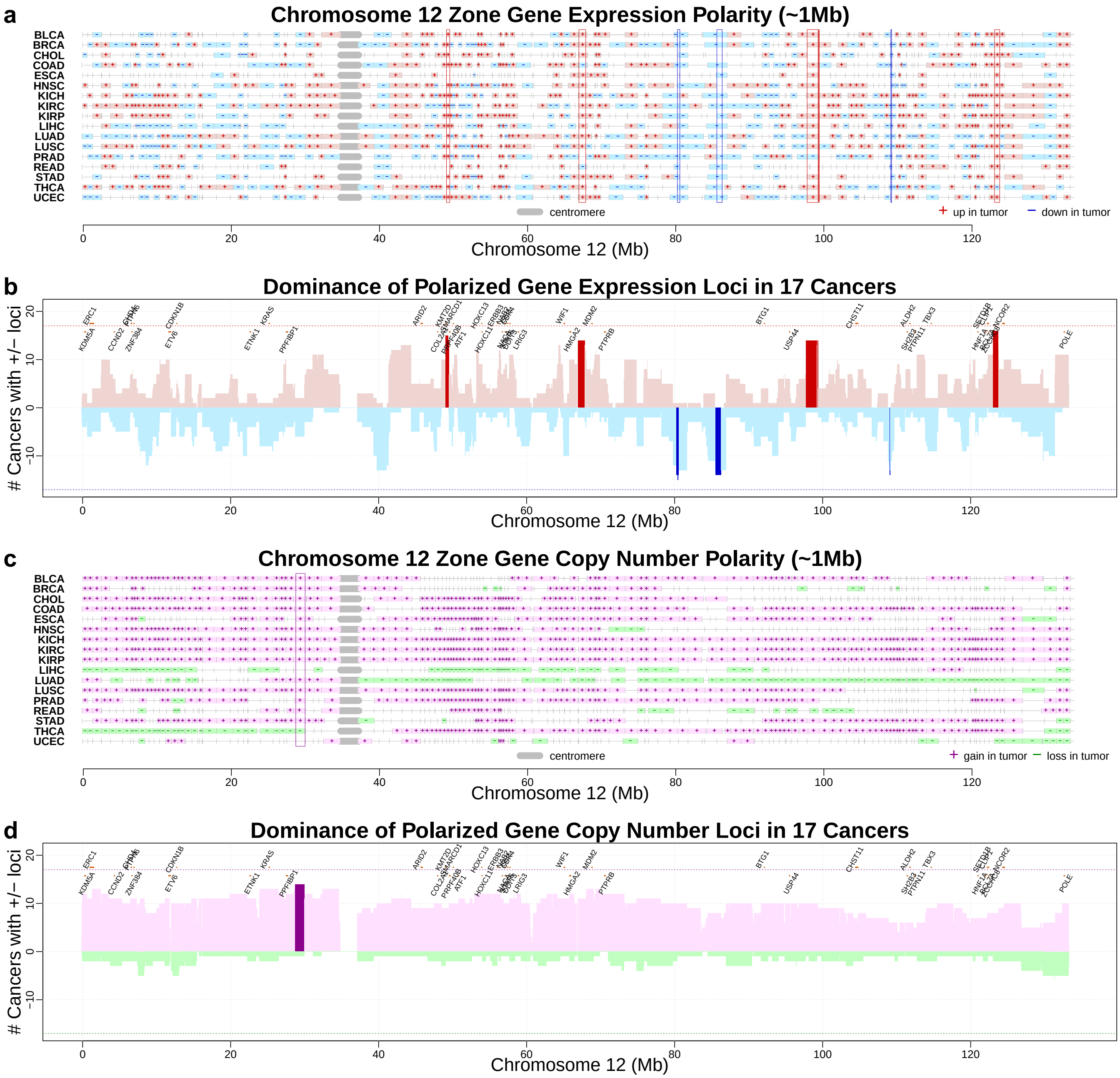

Supplementary Figure S1.12: Genomic zone maps along chromosome 12 in 17 cancer types versus their matched normal tissues. See the legend on page 1.

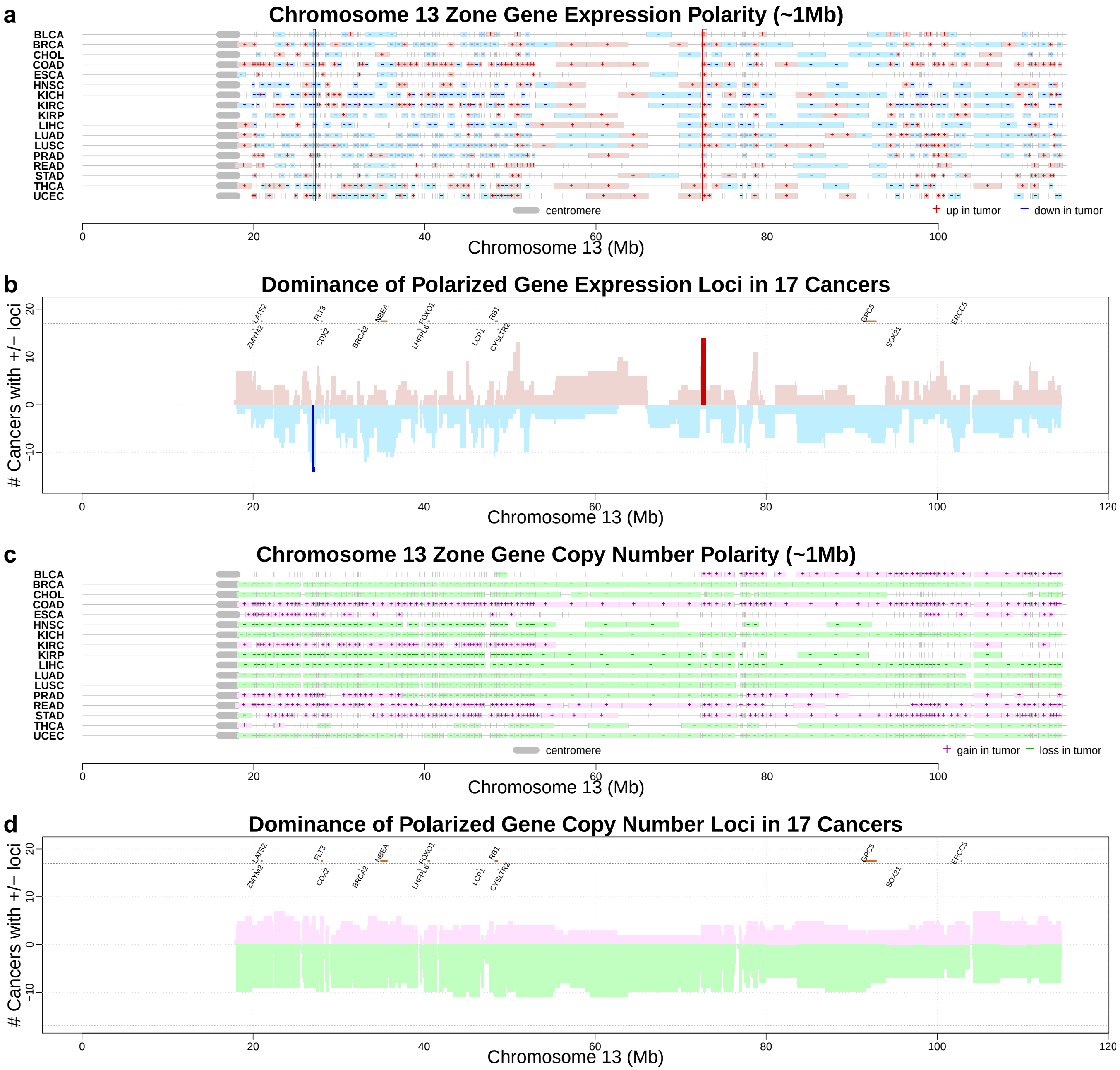

Supplementary Figure S1.13: Genomic zone maps along chromosome 13 in 17 cancer types versus their matched normal tissues. See the legend on page 1.

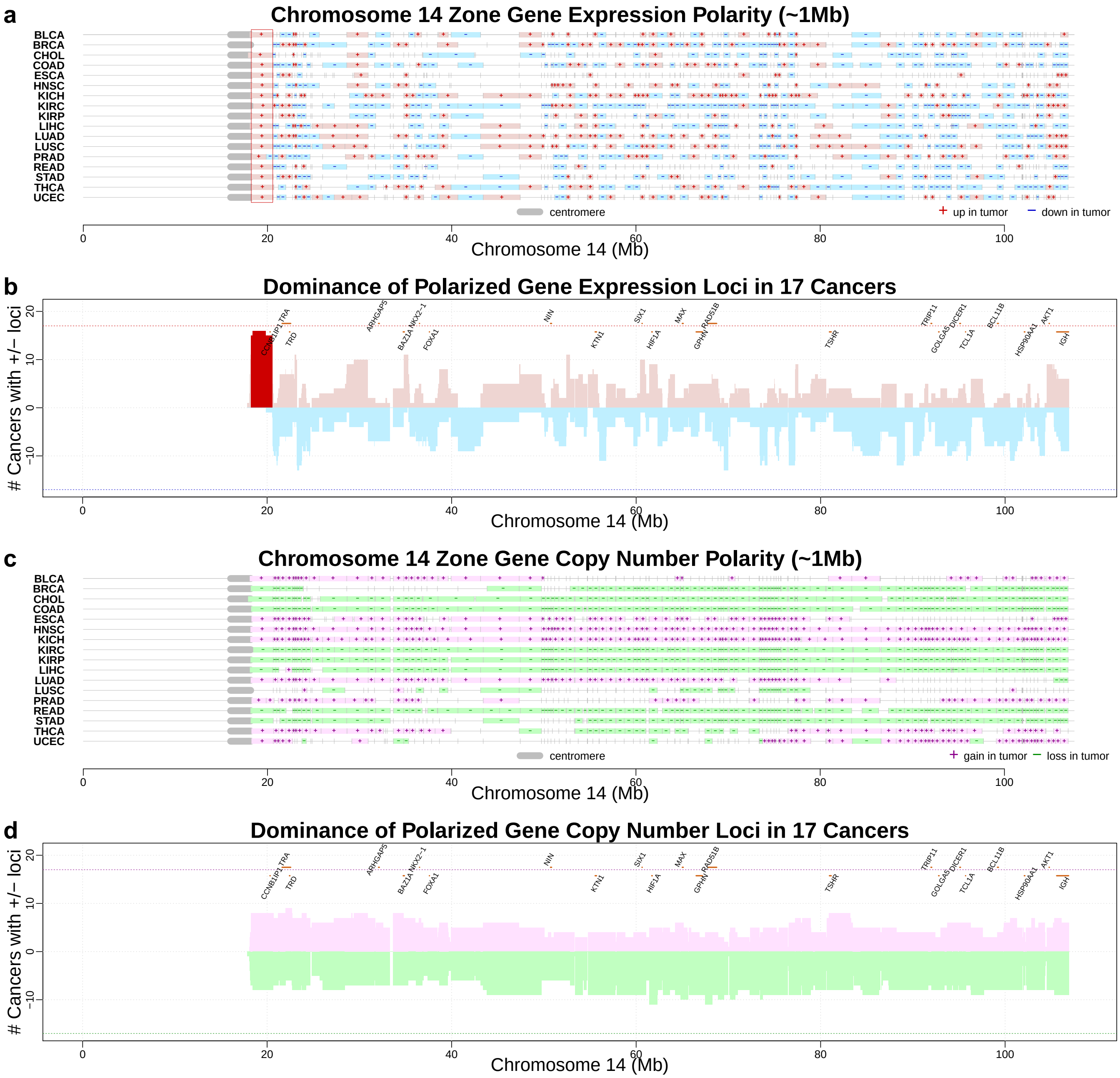

Supplementary Figure S1.14: Genomic zone maps along chromosome 14 in 17 cancer types versus their matched normal tissues. See the legend on page 1.

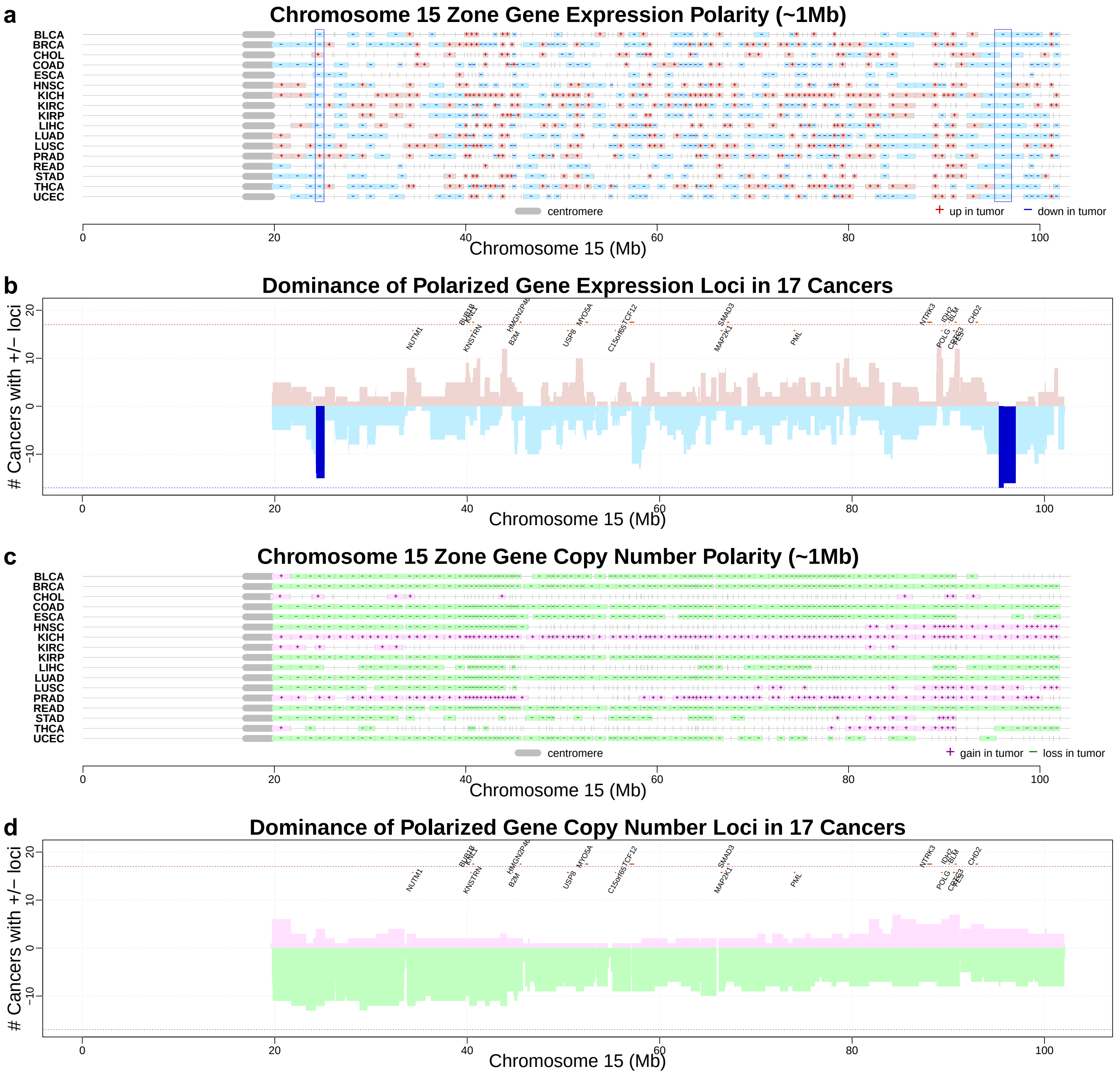

Supplementary Figure S1.15: Genomic zone maps along chromosome 15 in 17 cancer types versus their matched normal tissues. See the legend on page 1.

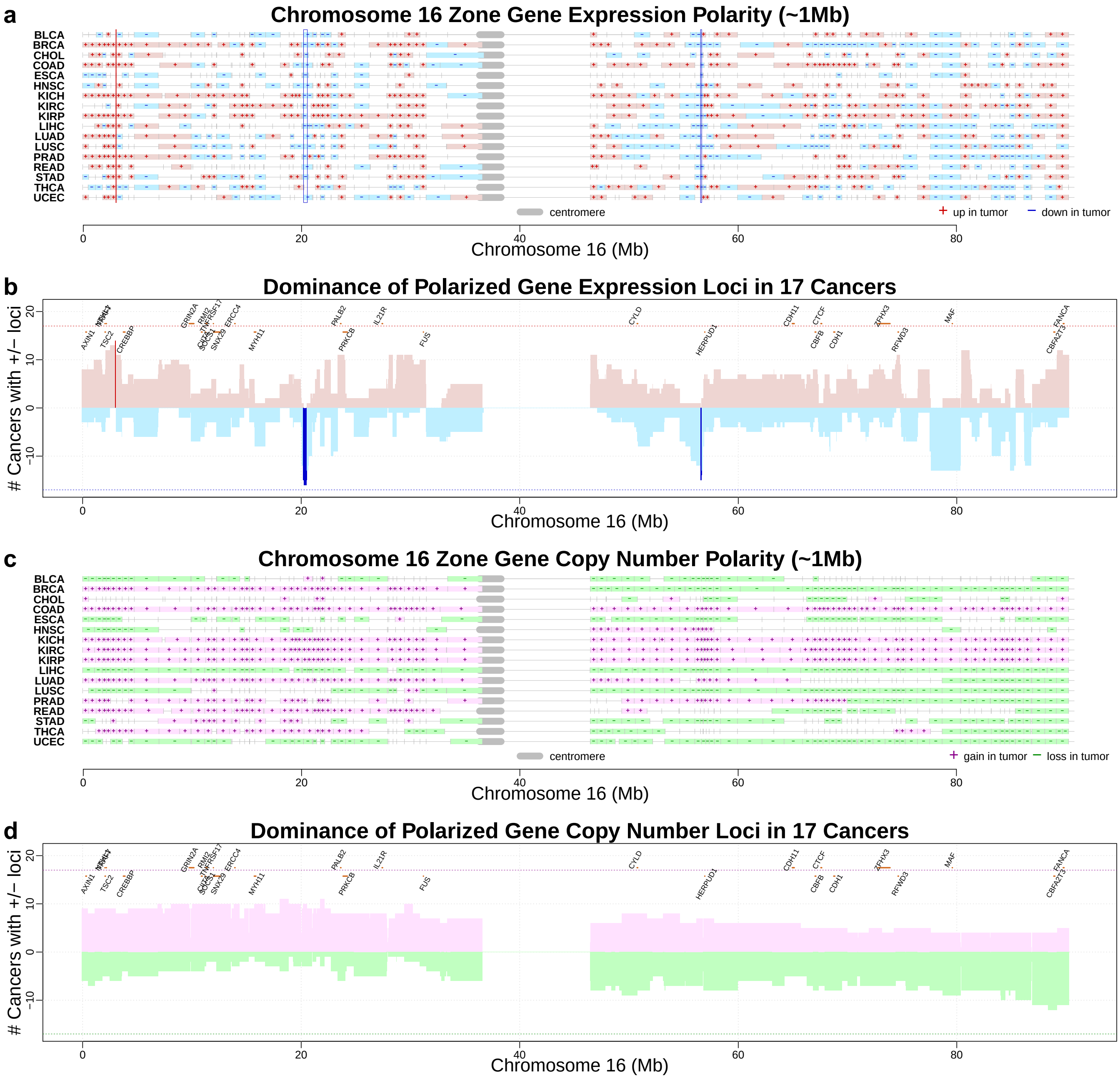

Supplementary Figure S1.16: Genomic zone maps along chromosome 16 in 17 cancer types versus their matched normal tissues. See the legend on page 1.

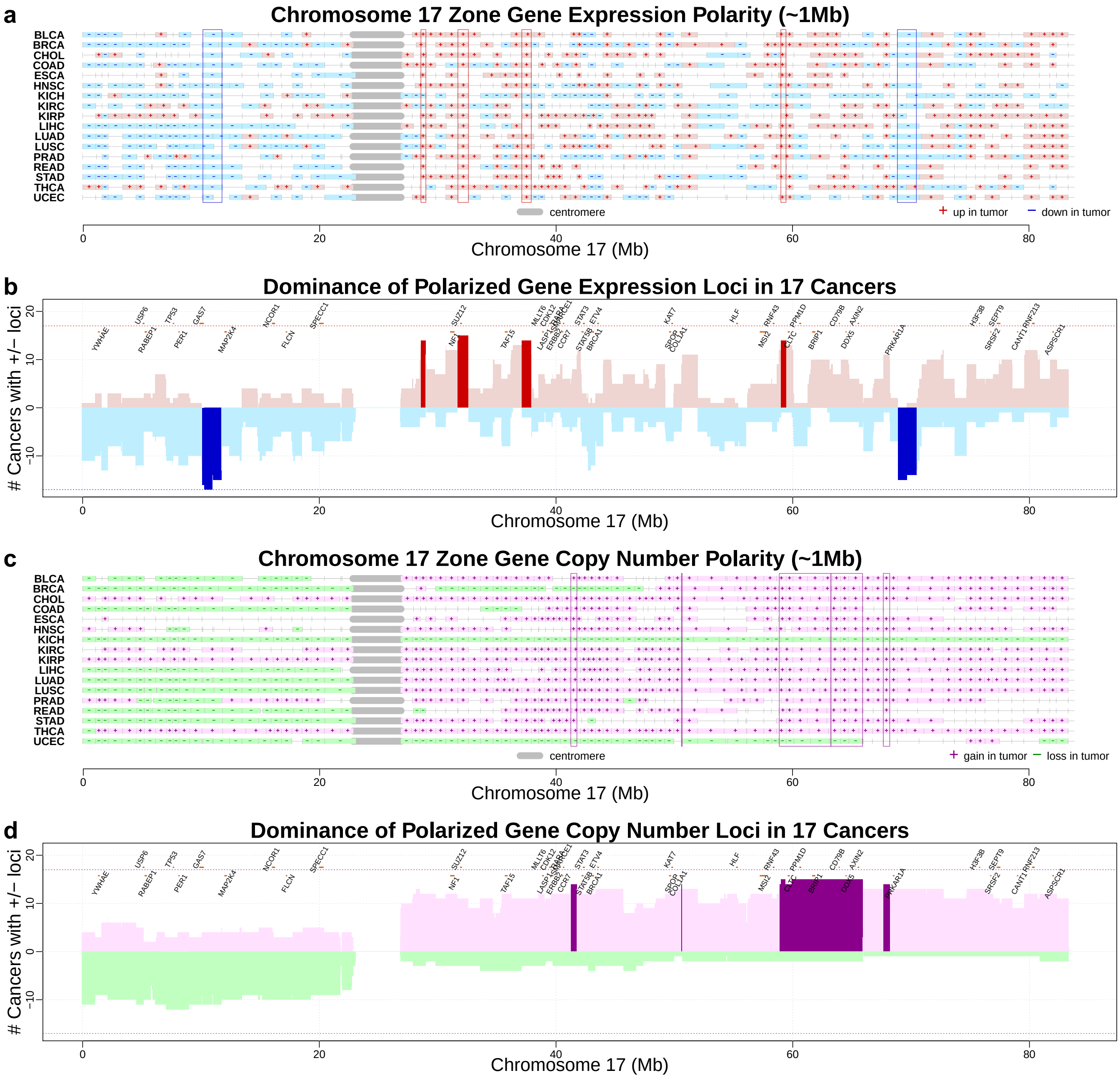

Supplementary Figure S1.17: Genomic zone maps along chromosome 17 in 17 cancer types versus their matched normal tissues. See the legend on page 1.

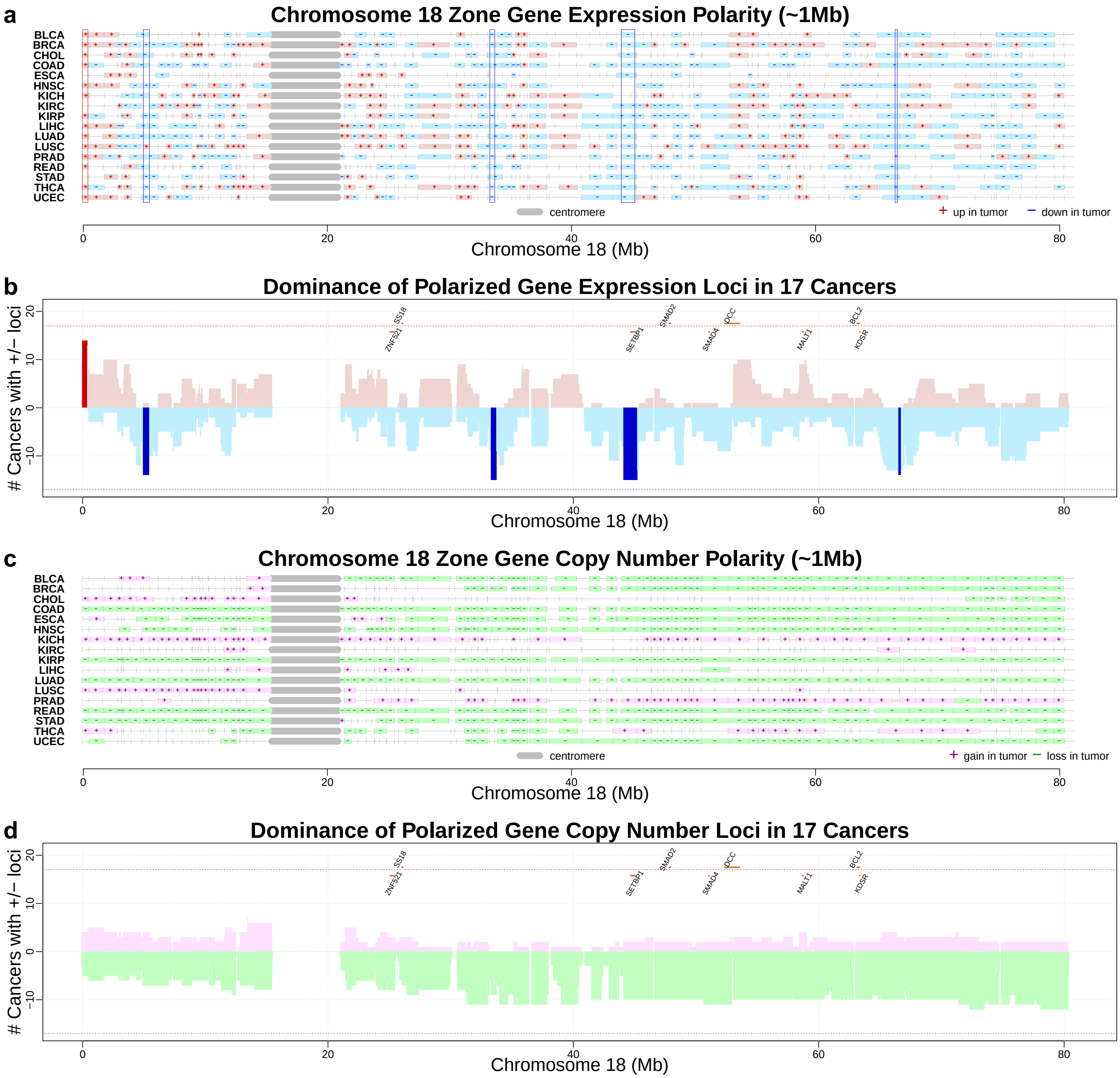

Supplementary Figure S1.18: Genomic zone maps along chromosome 18 in 18 cancer types versus their matched normal tissues. See the legend on page 1.

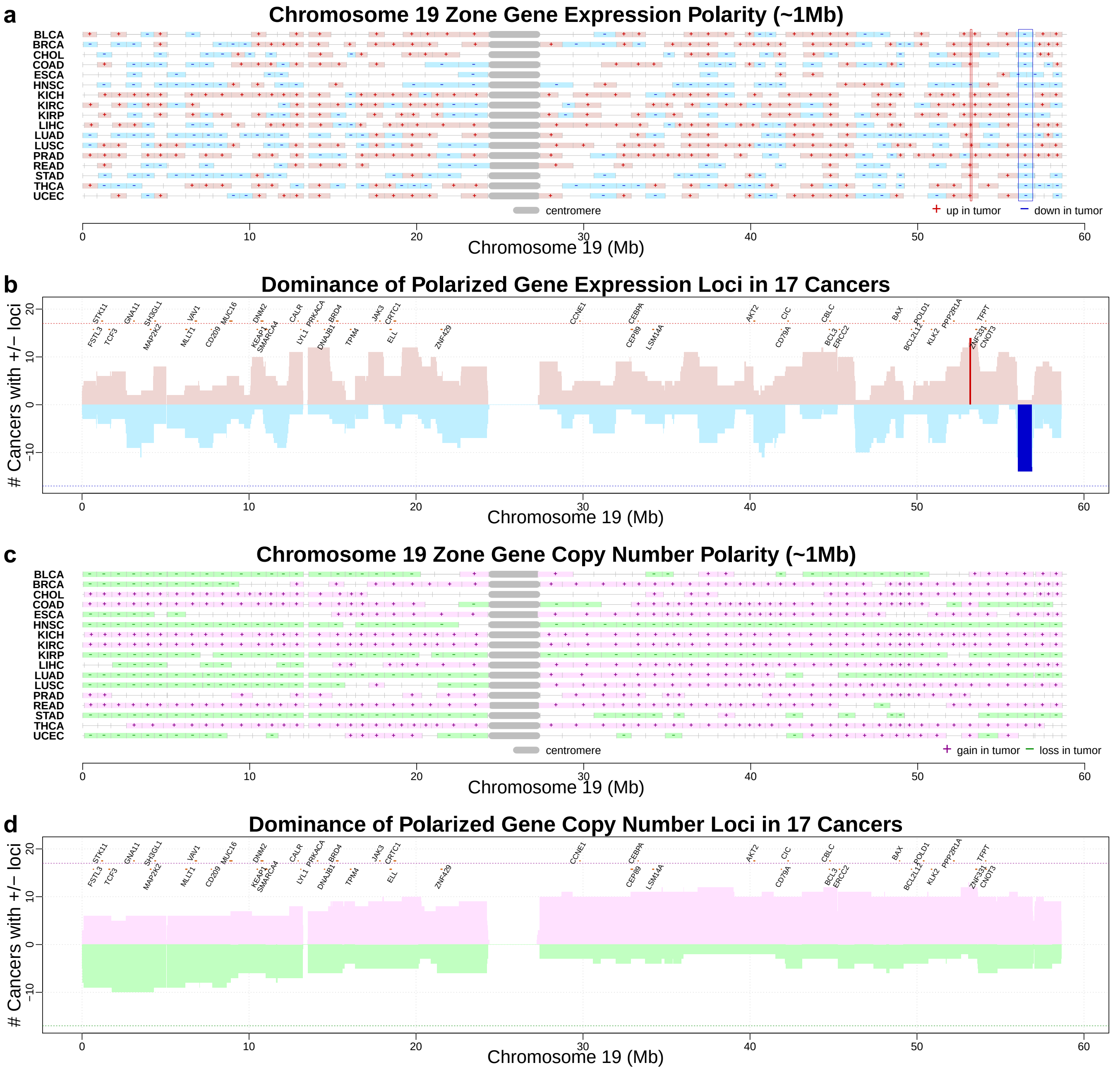

Supplementary Figure S1.19: Genomic zone maps along chromosome 19 in 17 cancer types versus their matched normal tissues. See the legend on page 1.

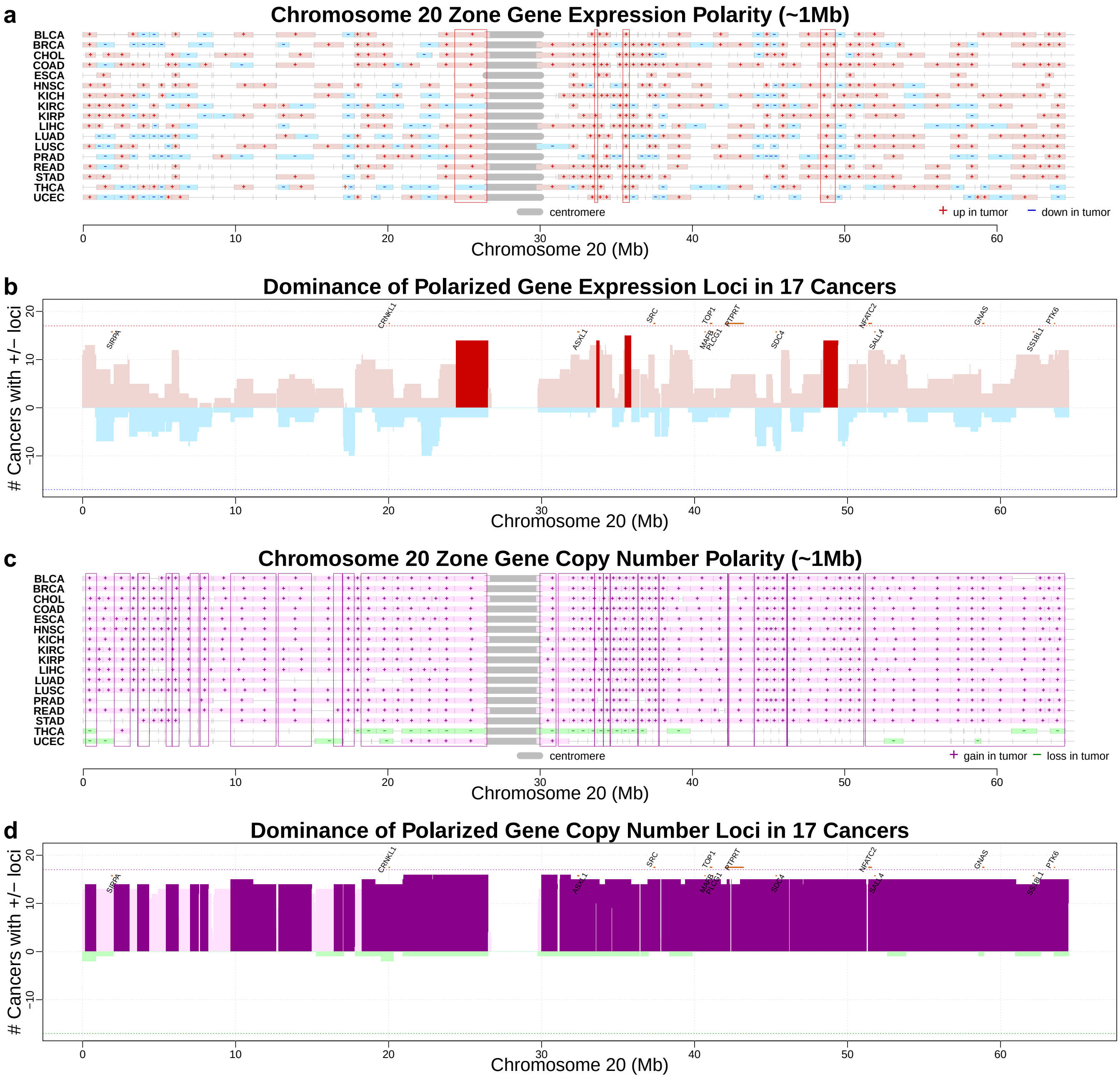

Supplementary Figure S1.20: Genomic zone maps along chromosome 20 in 17 cancer types versus their matched normal tissues. See the legend on page 1.

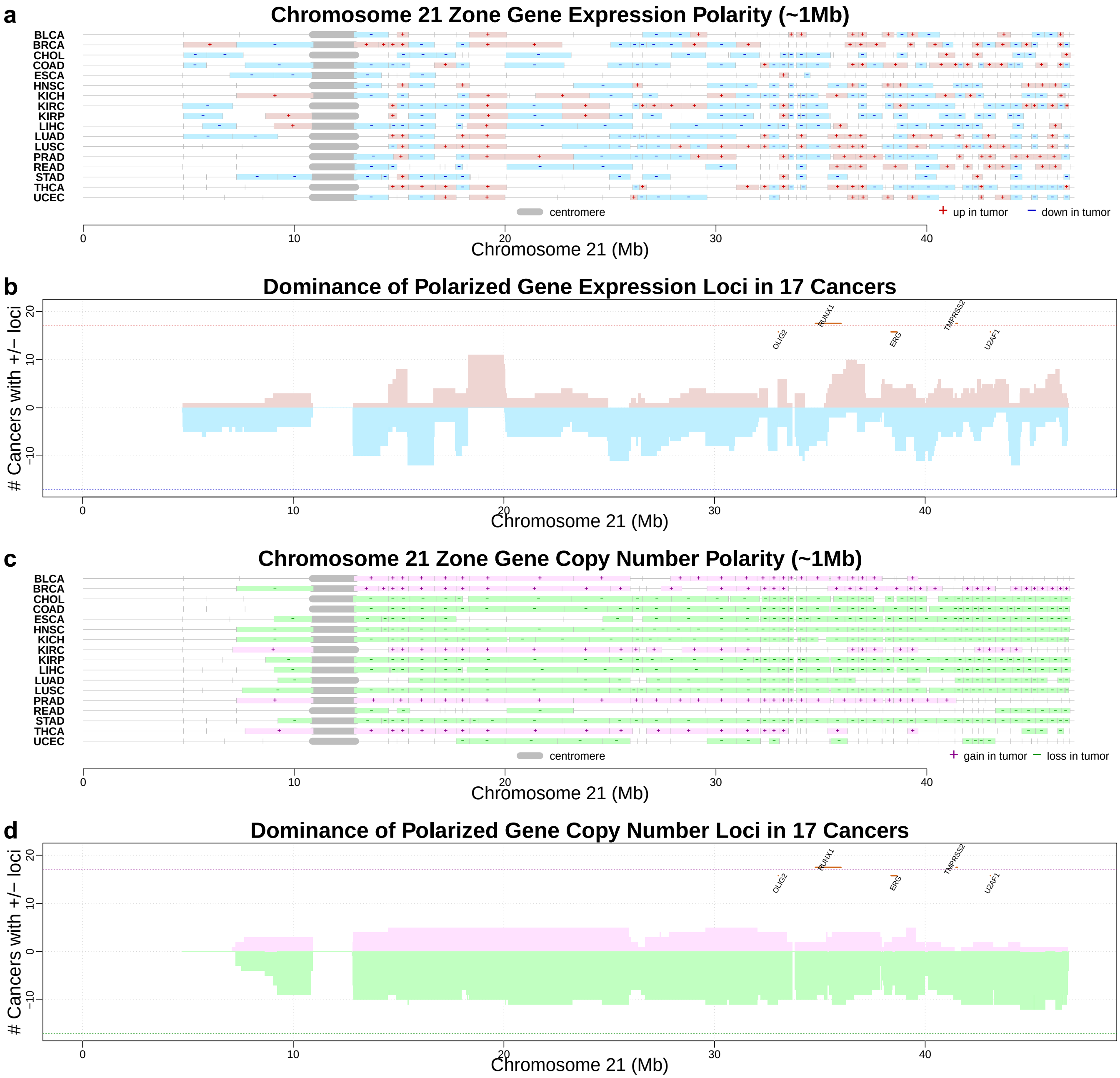

Supplementary Figure S1.21: Genomic zone maps along chromosome 21 in 17 cancer types versus their matched normal tissues. See the legend on page 1.

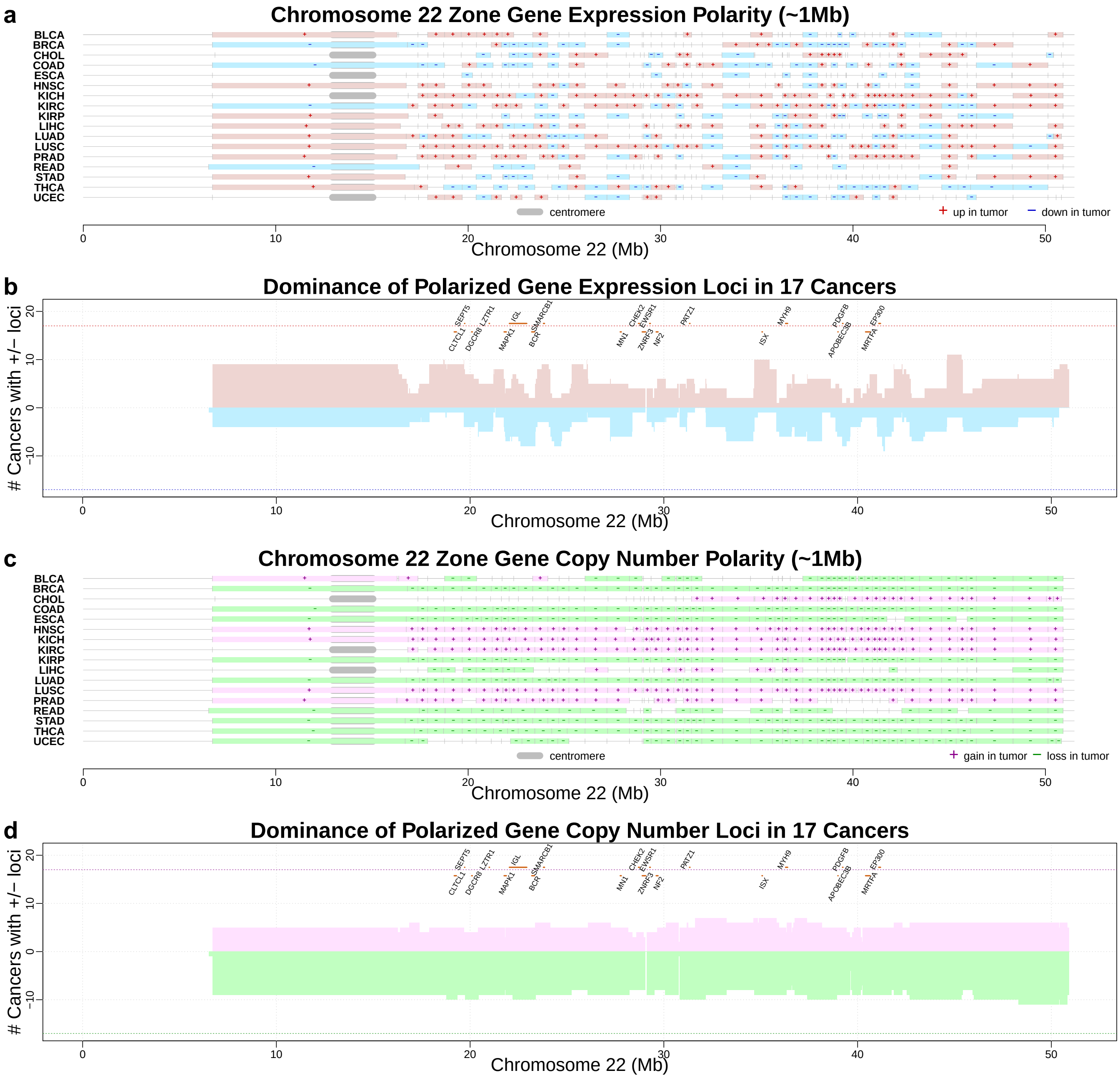

Supplementary Figure S1.22: Genomic zone maps along chromosome 22 in 17 cancer types versus their matched normal tissues. See the legend on page 1.

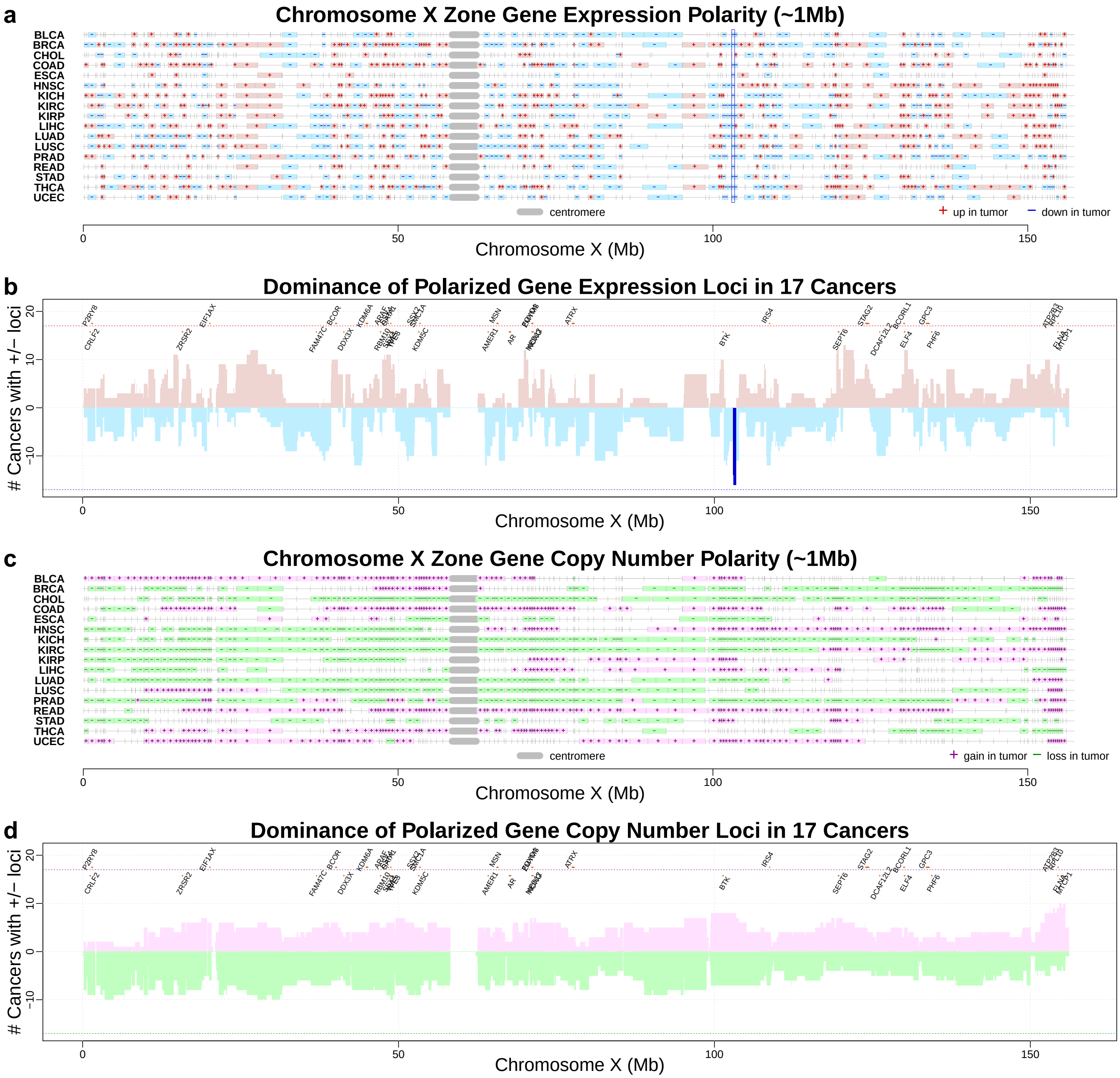

Supplementary Figure S1.23: Genomic zone maps along chromosome X in 17 cancer types versus their matched normal tissues. See the legend on page 1.

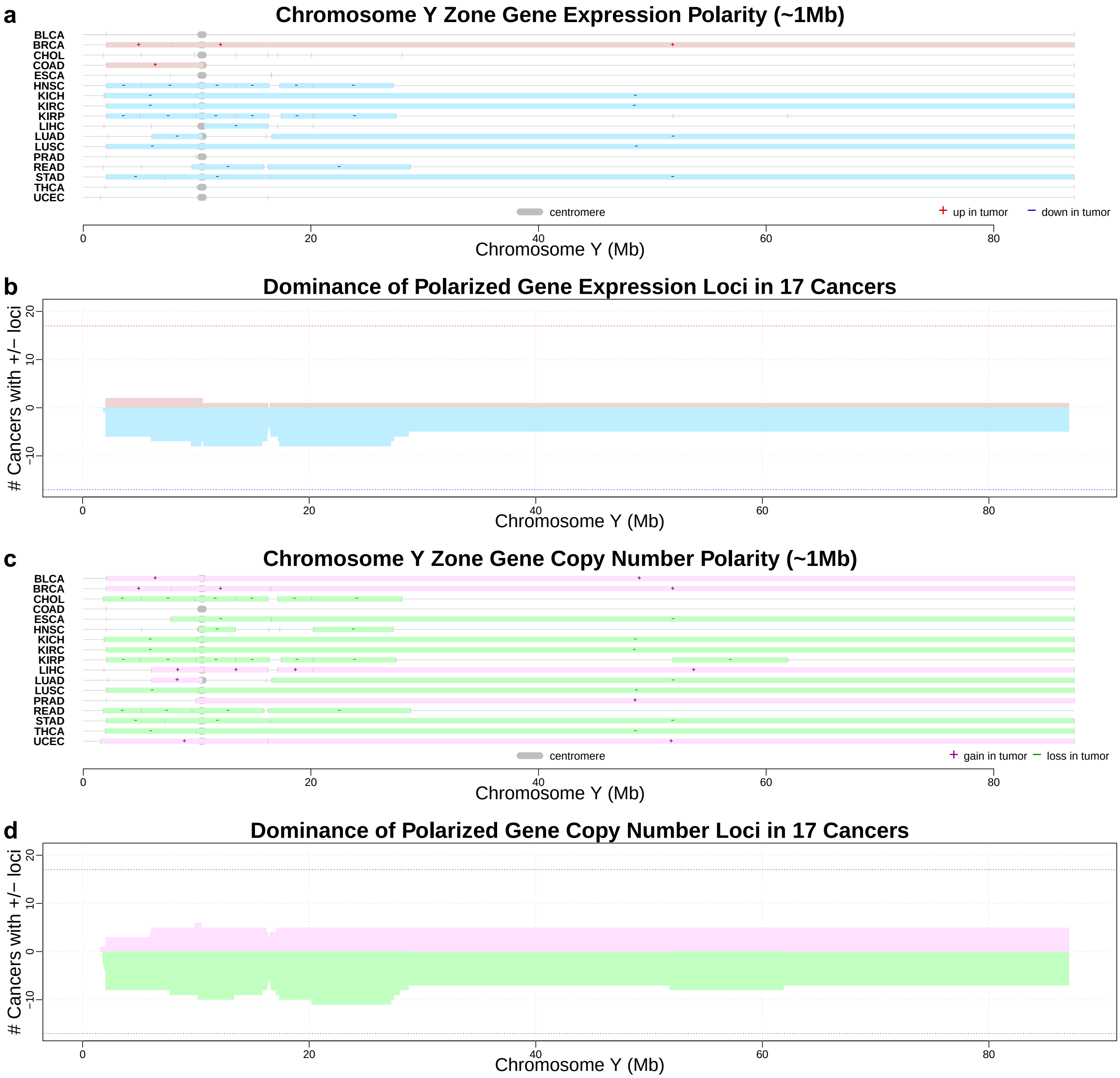

Supplementary Figure S1.24: Genomic zone maps along chromosome Y in 17 cancer types versus their matched normal tissues. See the legend on page 1.
