# Supplementary material for: Efficient weighted univariate clustering maps outstanding dysregulated genomic zones in human cancers
Source: Bioinformatics. 2020 Jul 3;36(20):5027–36. doi: 10.1093/bioinformatics/btaa613 (PMC7755420; doi:10.1093/bioinformatics/btaa613)
Supplement: btaa613_Supplementary_Data [file btaa613_supplementary_data.zip › SuppFig-S2.pdf]

Supplementary File S2: Maps of genomic zone showing polarity in gene regulation and somatic copy number alteration in 17 cancer types (organized by cancer type)

List of Figures

S2.1 BLCA . . . S2-2
S2.2 BRCA . . . S2-3
S2.3 CHOL . . . S2-4
S2.4 COAD . . . S2-5
S2.5 ESCA . . . S2-6
S2.6 HNSC . . . S2-7
S2.7 KICH . . . S2-8
S2.8 KIRC . . . S2-9
S2.9 KIRP . . . S2-10
S2.10 LIHC . . . S2-11
S2.11 LUAD . . . S2-12
S2.12 LUSC . . . S2-13
S2.13 PRAD . . . S2-14
S2.14 READ . . . S2-15
S2.15 STAD . . . S2-16
S2.16 THCA . . . S2-17
S2.17 UCEC . . . S2-18

Legend used by the maps of genomic zone

a, For the cancer type shown, chromosomes 1-22, X, and Y are marked by genomic zone boundaries and polarization in **regulation**. Horizontal patterns are formed by consecutive zones of the same polarity along chromosomes. Red '+' and blue '-' mark statistically significant positive and negative zone polarization, corresponding to dominance of up- and down-regulated genes, respectively.

b, For the same cancer type shown, chromosomes 1-22, X, and Y are marked by genomic zone boundaries and polarization in **somatic copy number alteration (SCNA)**. Horizontal patterns are formed by consecutive zones of the same polarity along chromosomes. Purple '+' and green '-' mark statistically significant zone polarization in SCNA along the chromosome across 17 cancer types, corresponding to amplification and deletion, respectively.

a

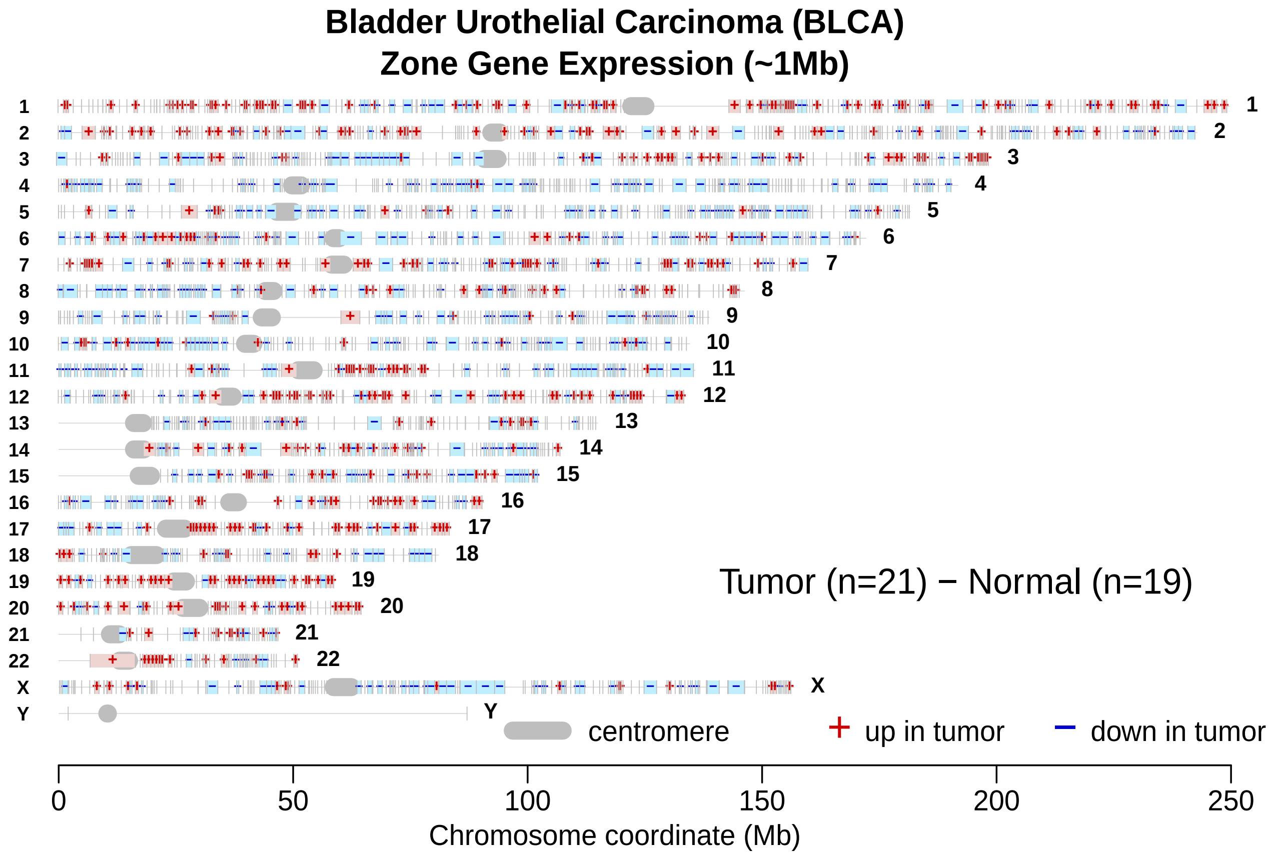

b

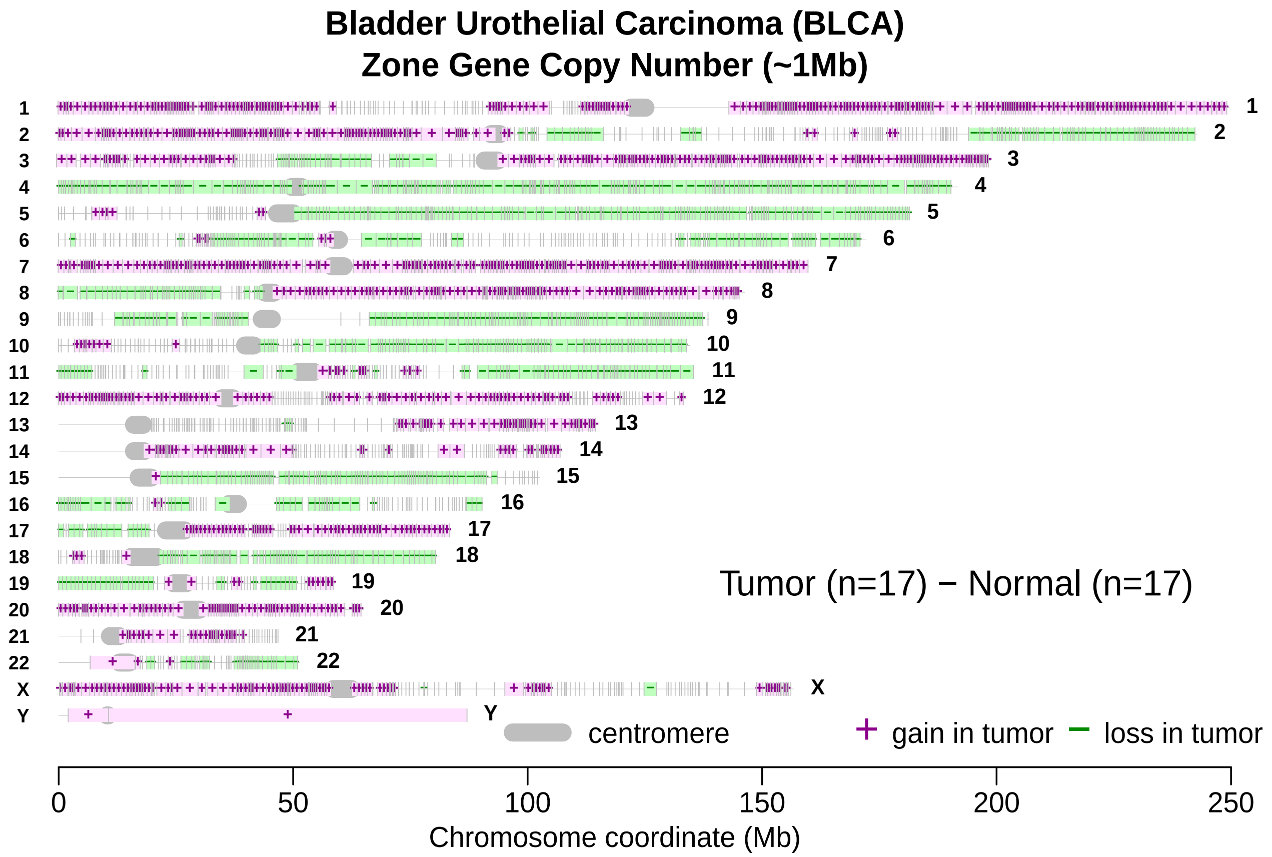

Supplementary Figure S2.1: Maps of genomic zone in BLCA. **a**, Polarization of zone regulation. **b**, Polarization of zone somatic copy number alteration. See the full legend on page 1.

a

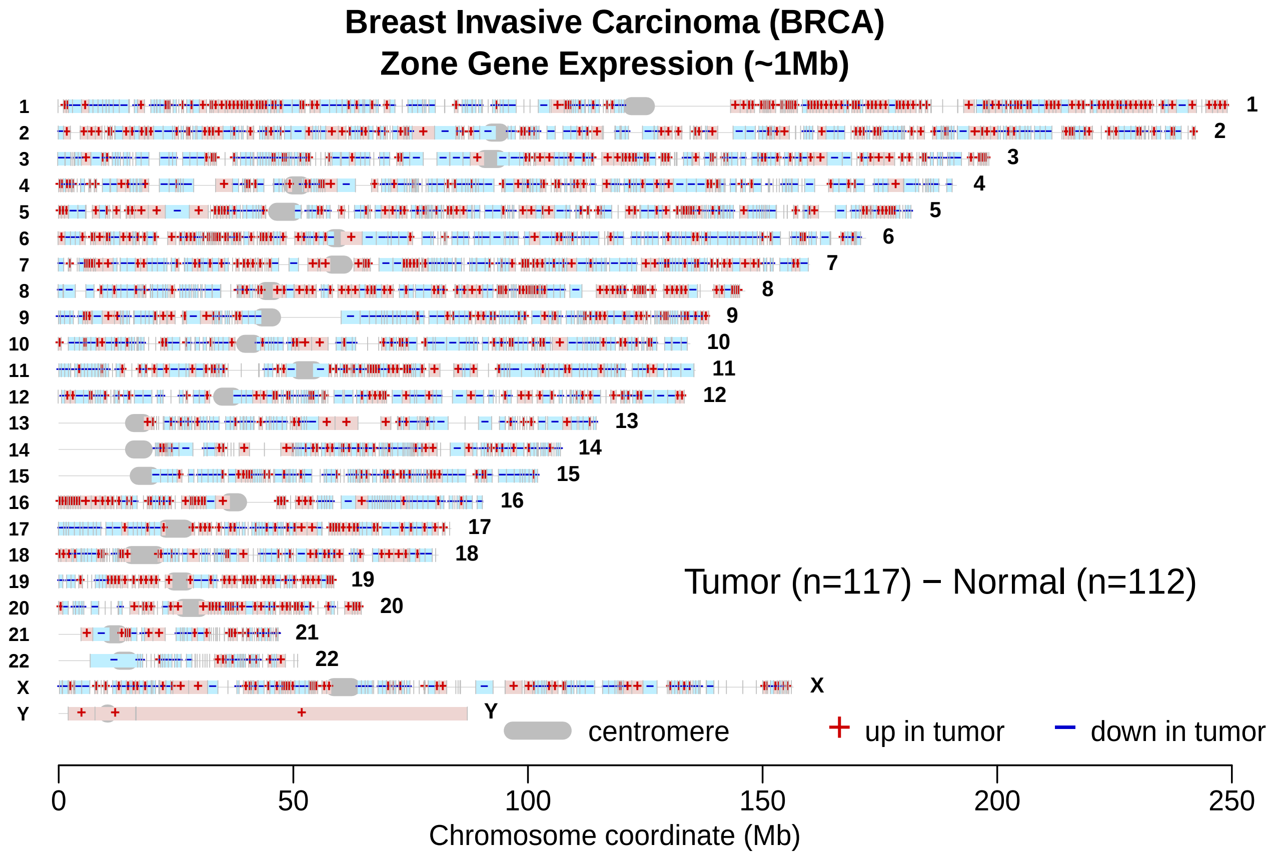

b

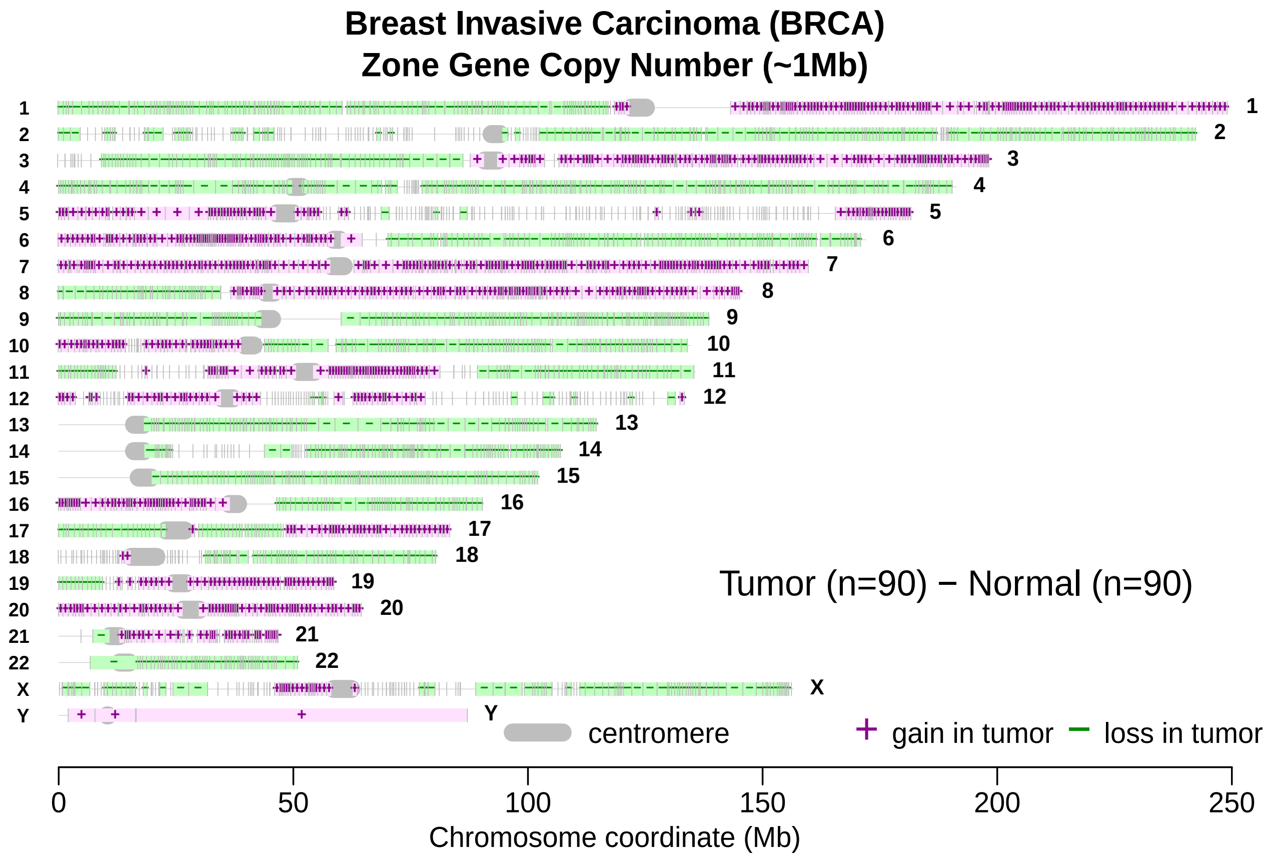

Supplementary Figure S2.2: Maps of genomic zone in BRCA. **a**, Polarization of zone regulation. **b**, Polarization of zone somatic copy number alteration. See the full legend on page 1.

a

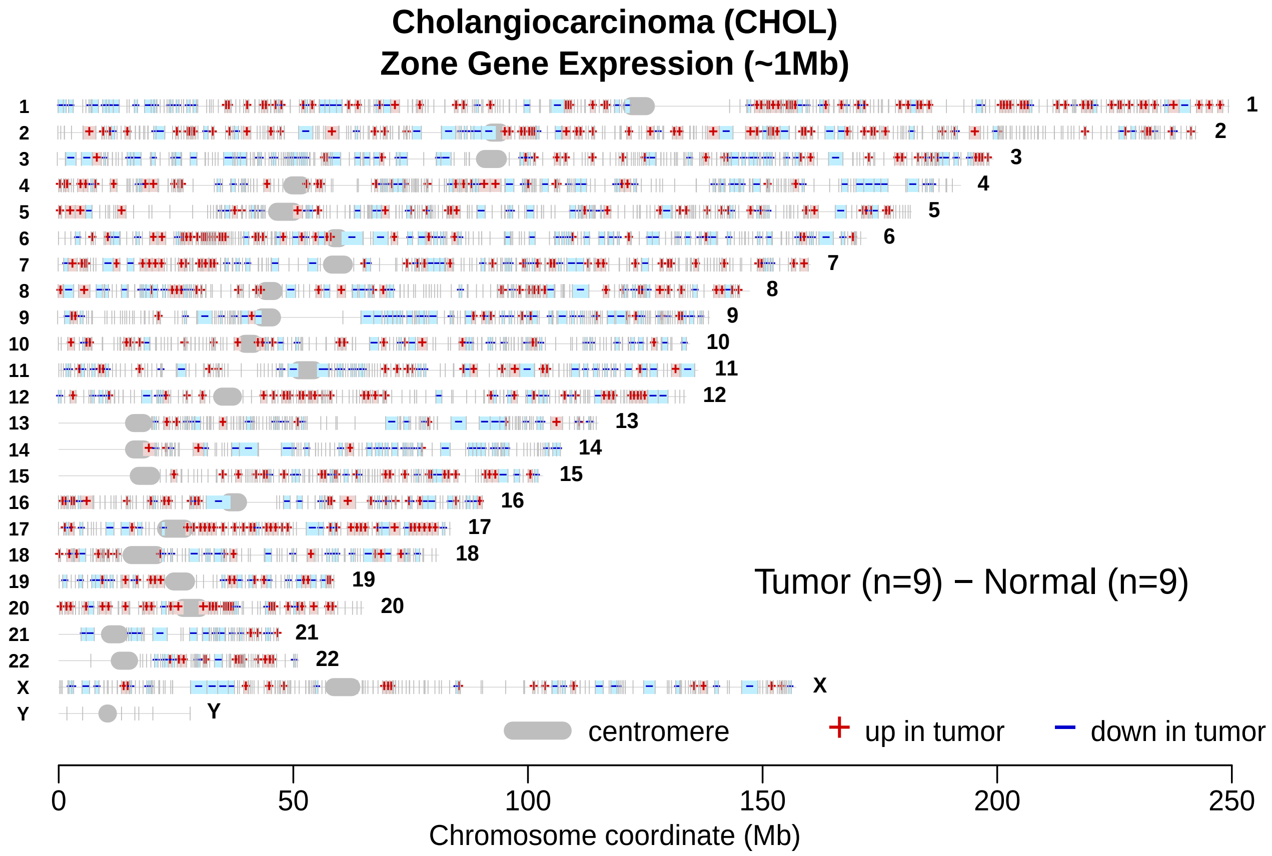

b

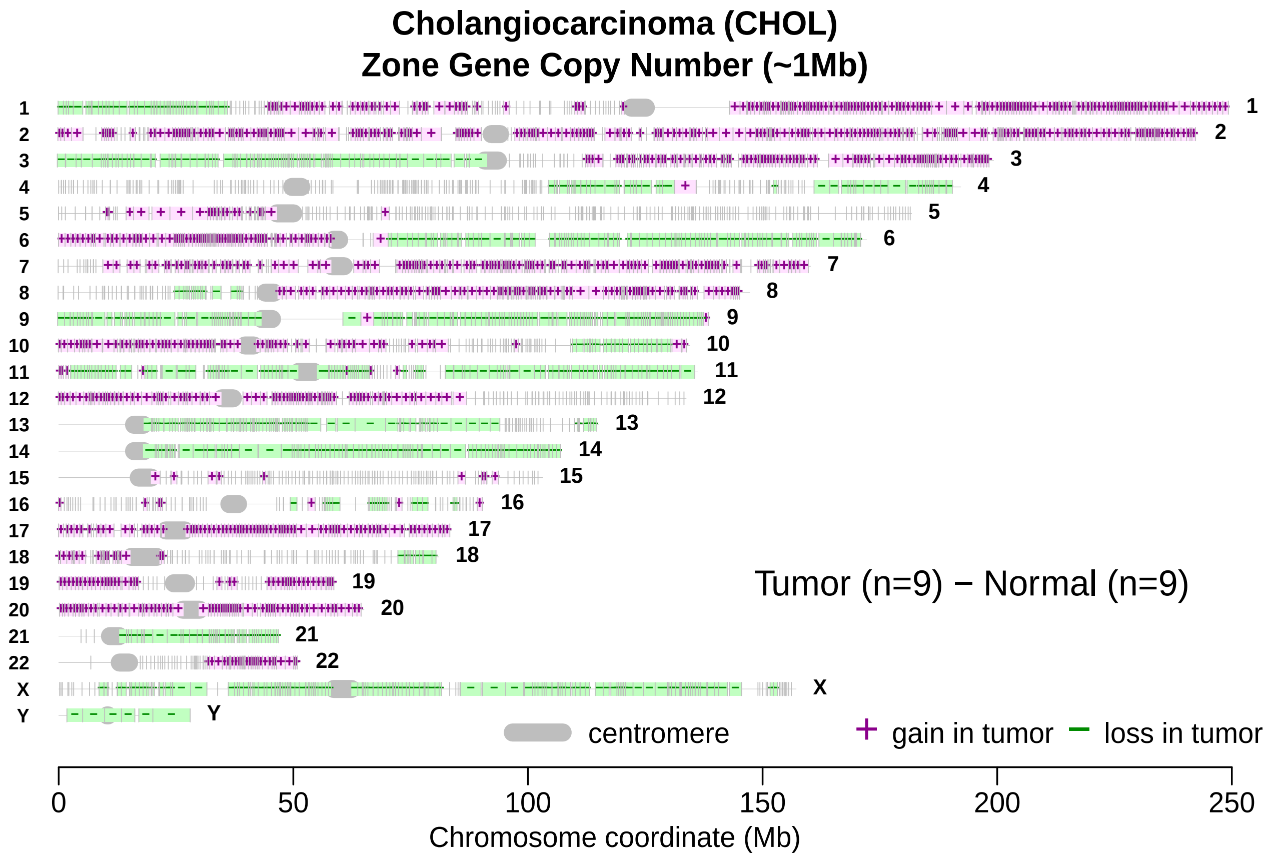

Supplementary Figure S2.3: Maps of genomic zone in CHOL. **a**, Polarization of zone regulation. **b**, Polarization of zone somatic copy number alteration. See the full legend on page 1.

a

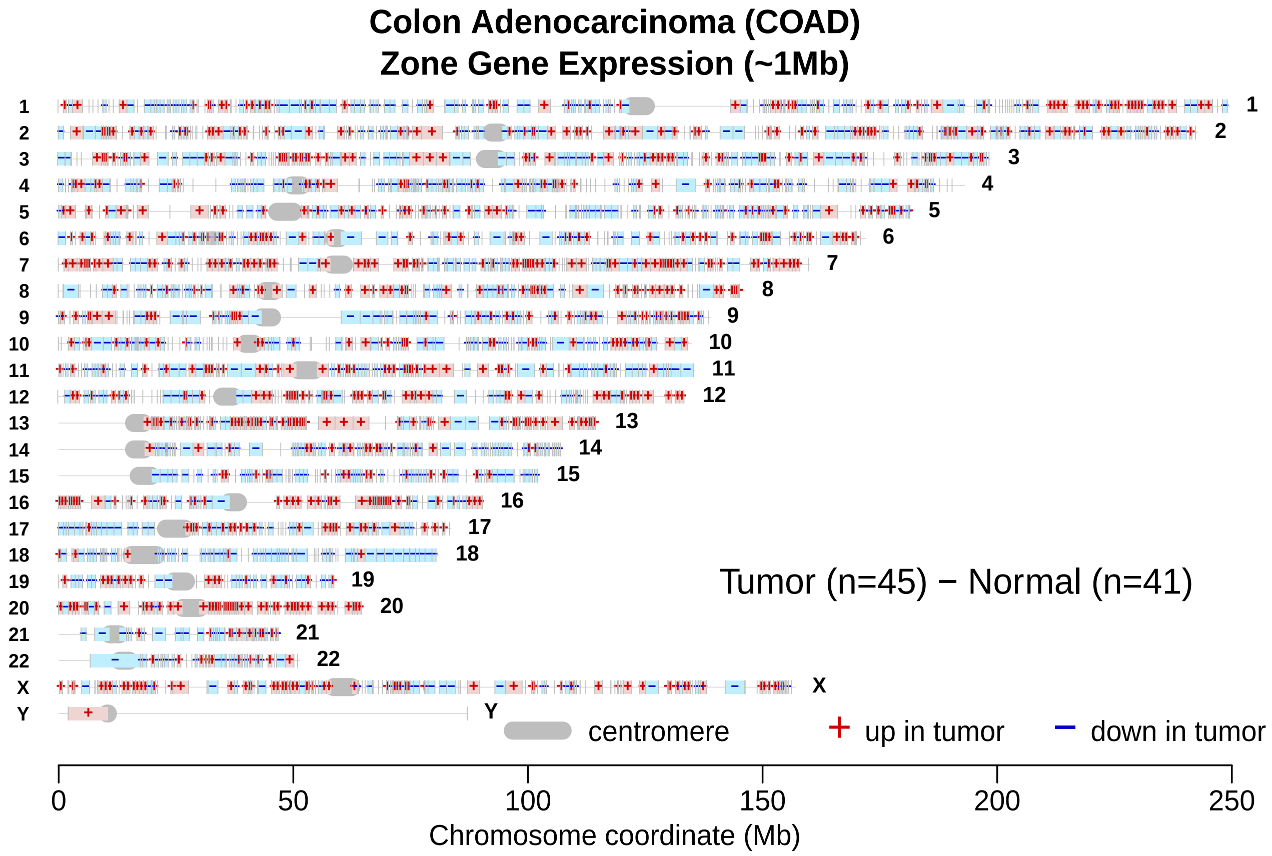

b

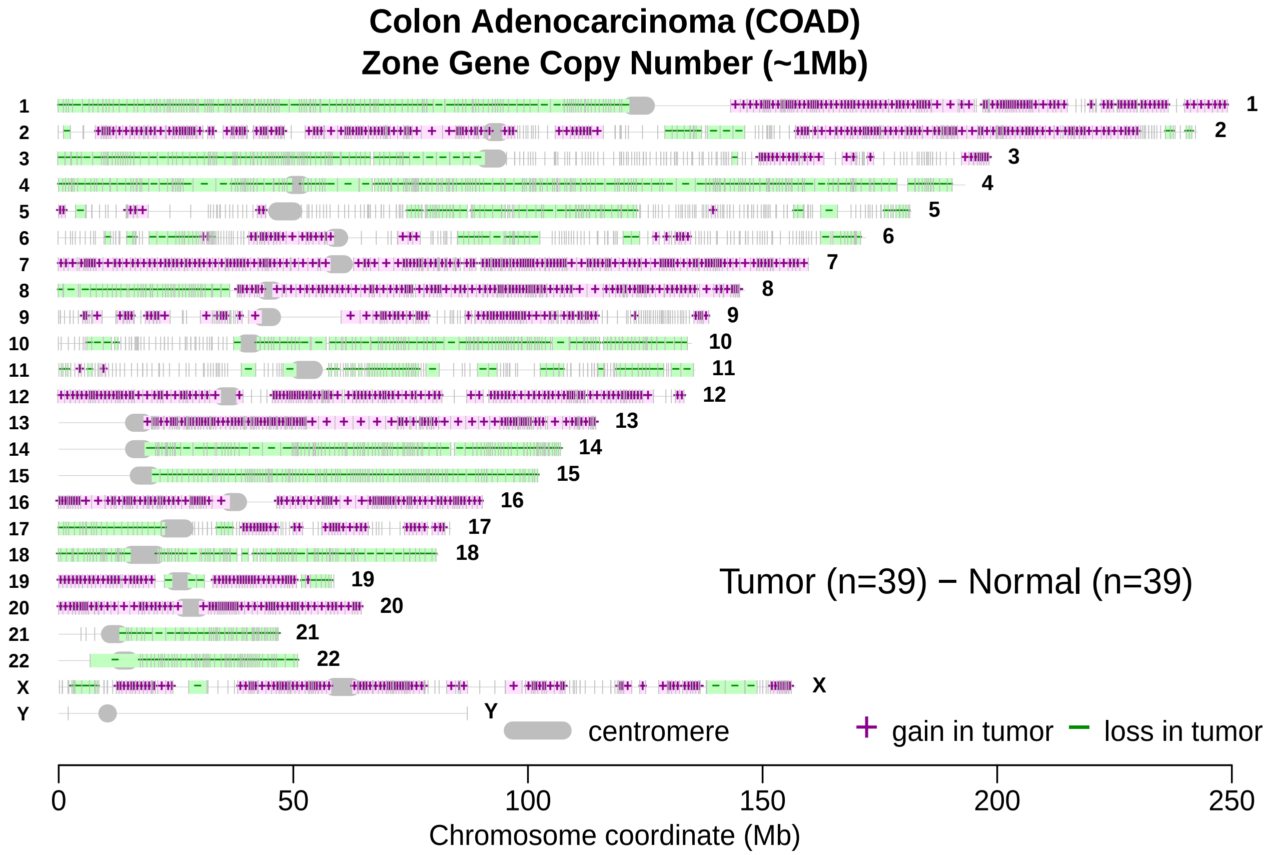

Supplementary Figure S2.4: Maps of genomic zone in COAD. **a**, Polarization of zone regulation. **b**, Polarization of zone somatic copy number alteration. See the full legend on page 1.

a

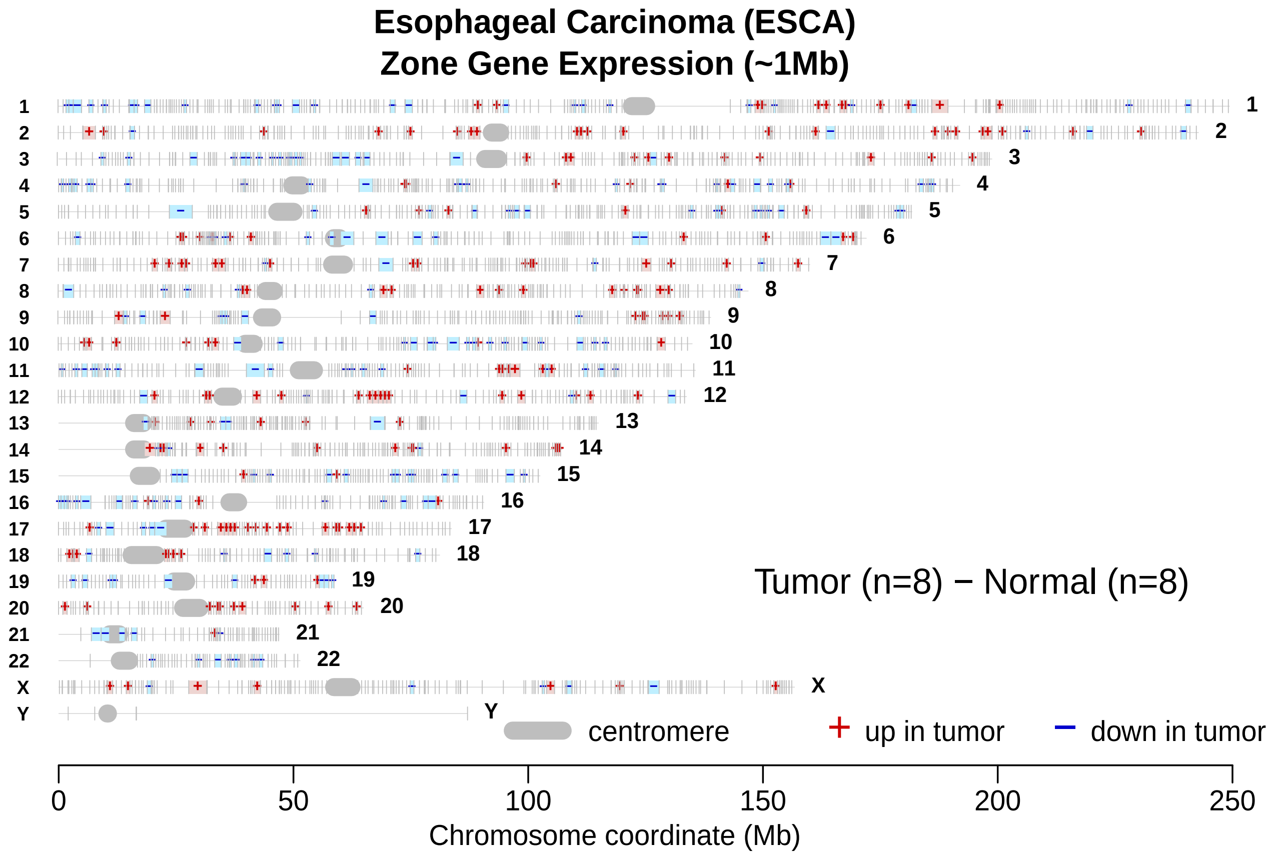

b

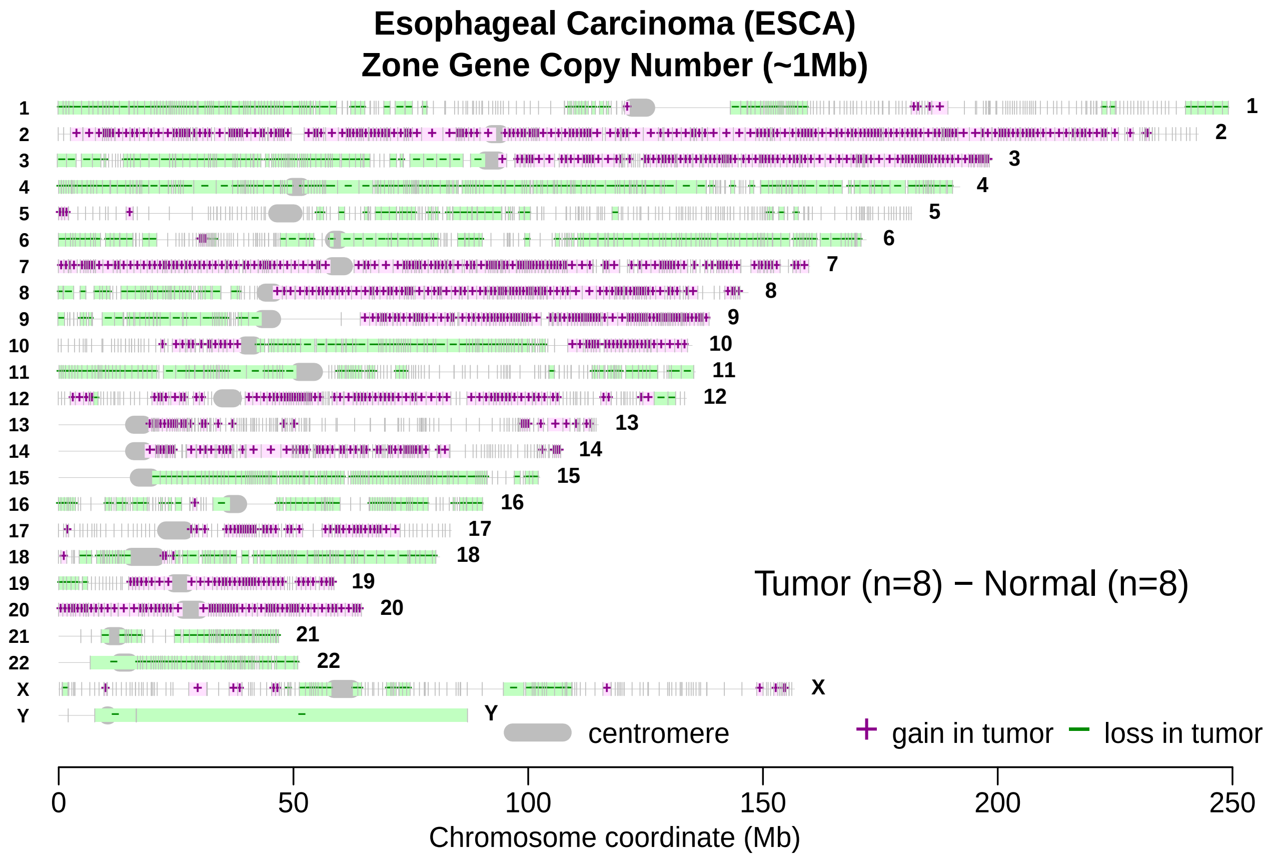

Supplementary Figure S2.5: Maps of genomic zone in ESCA. **a**, Polarization of zone regulation. **b**, Polarization of zone somatic copy number alteration. See the full legend on page 1.

a

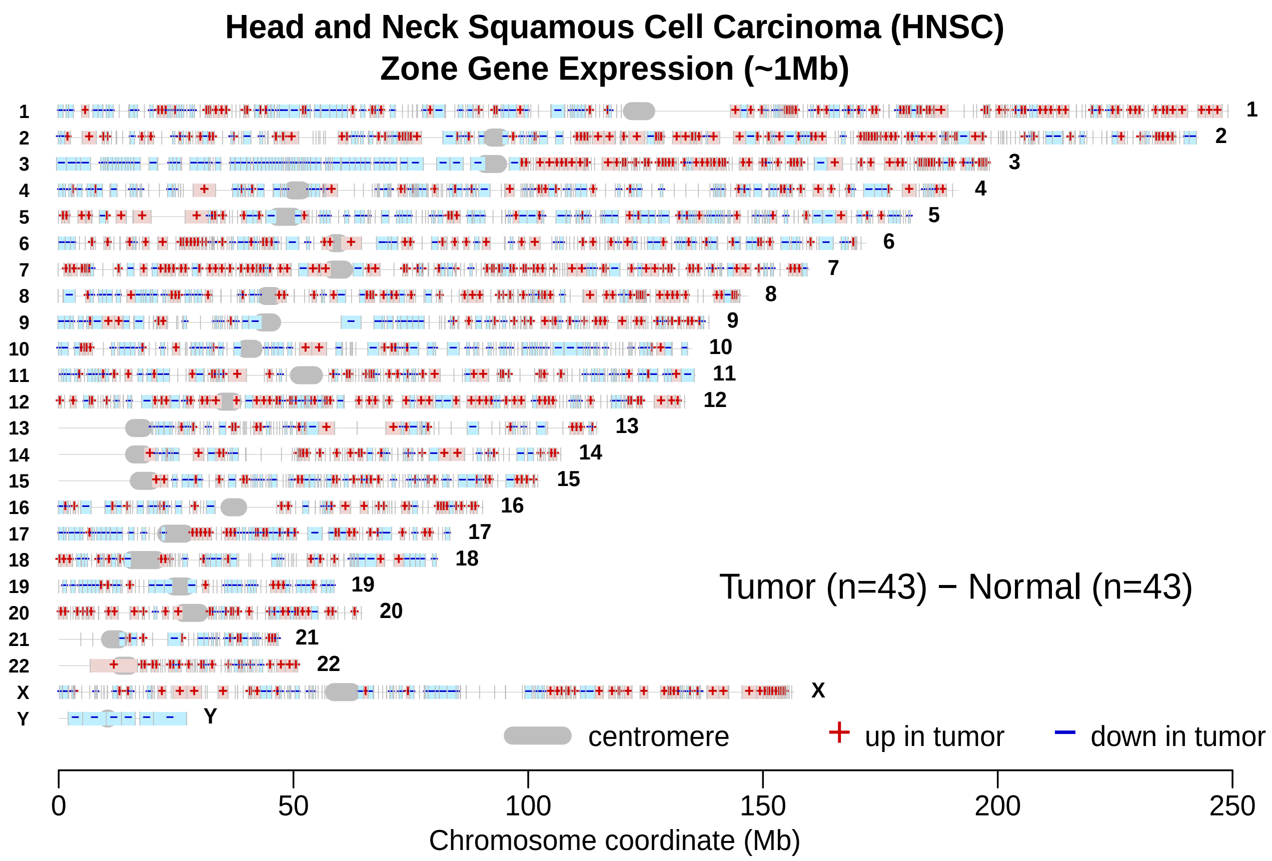

b

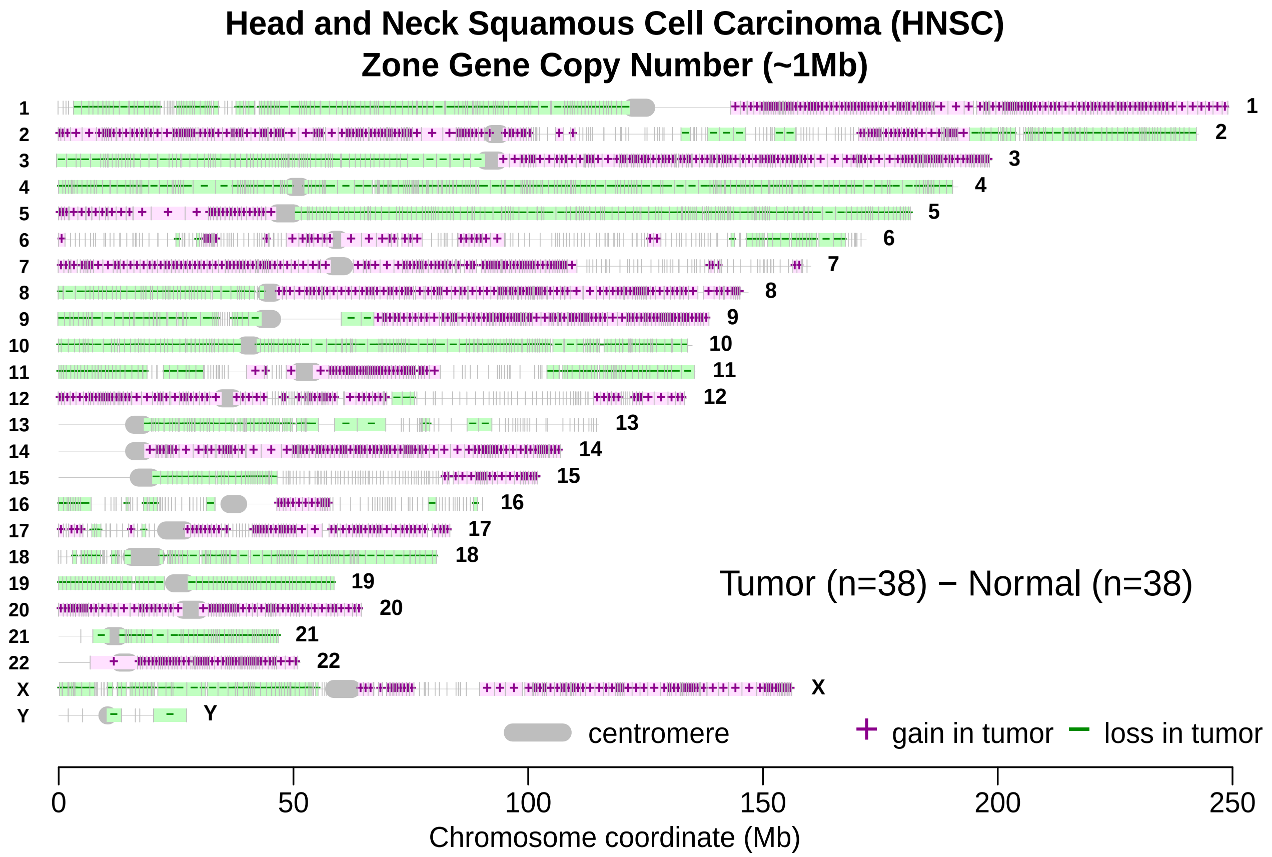

Supplementary Figure S2.6: Maps of genomic zone in HNSC. **a**, Polarization of zone regulation. **b**, Polarization of zone somatic copy number alteration. See the full legend on page 1.

a

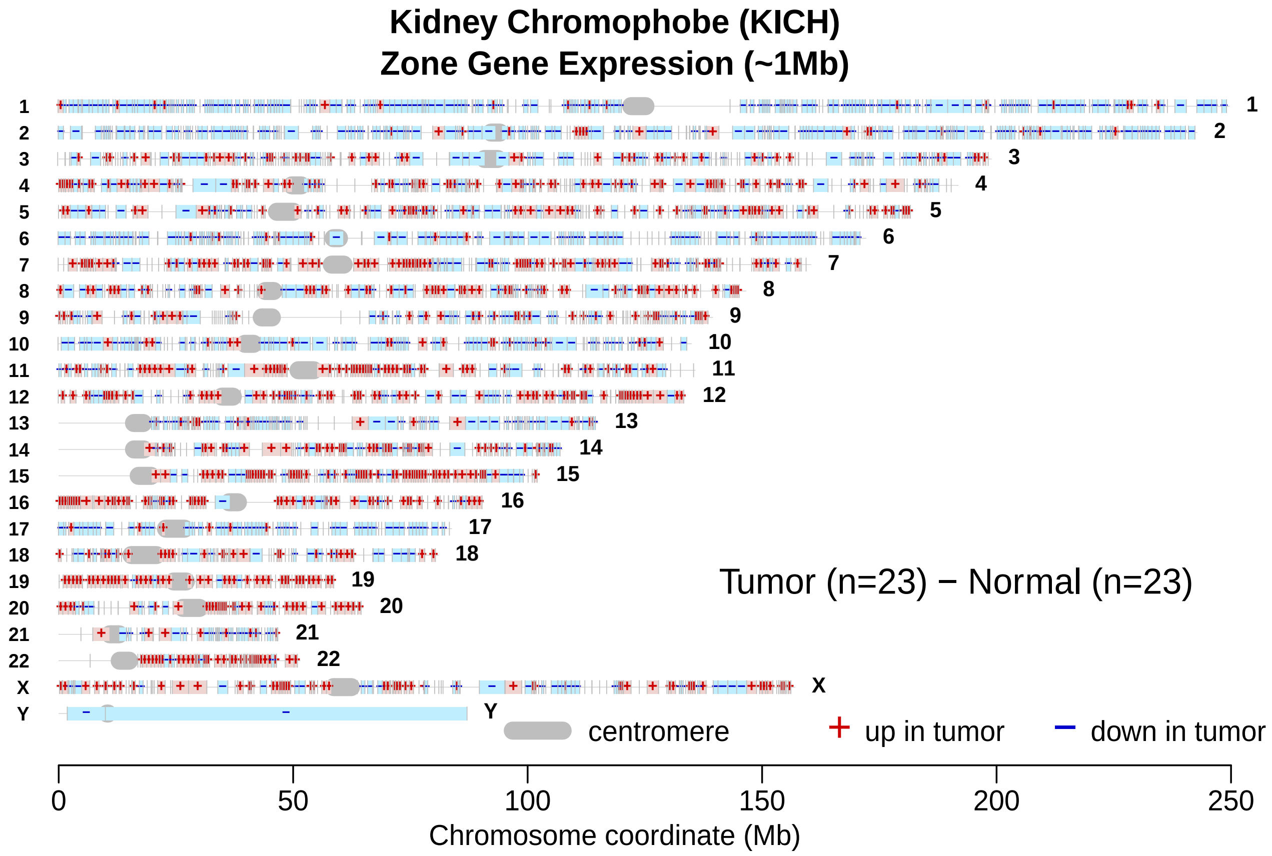

b

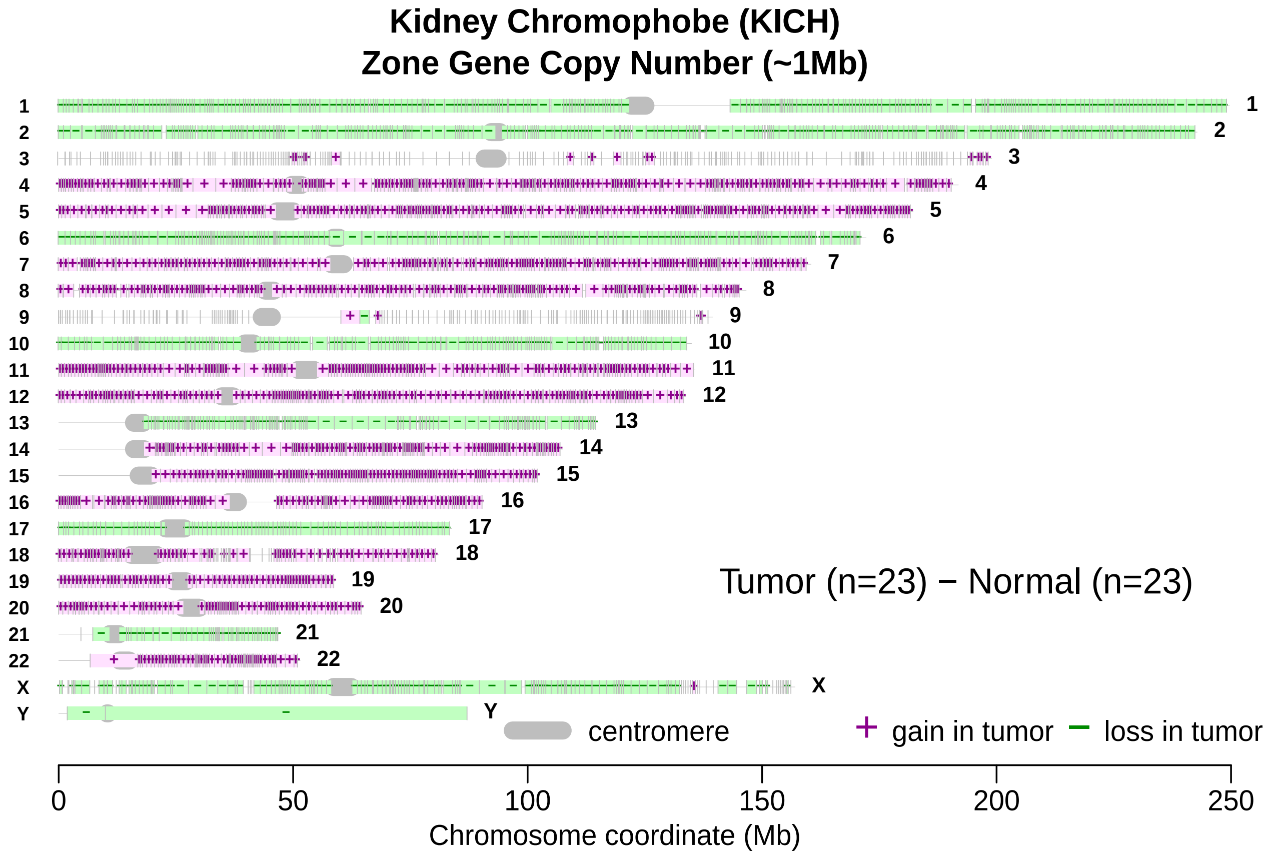

Supplementary Figure S2.7: Maps of genomic zone in KICH. **a**, Polarization of zone regulation. **b**, Polarization of zone somatic copy number alteration. See the full legend on page 1.

a

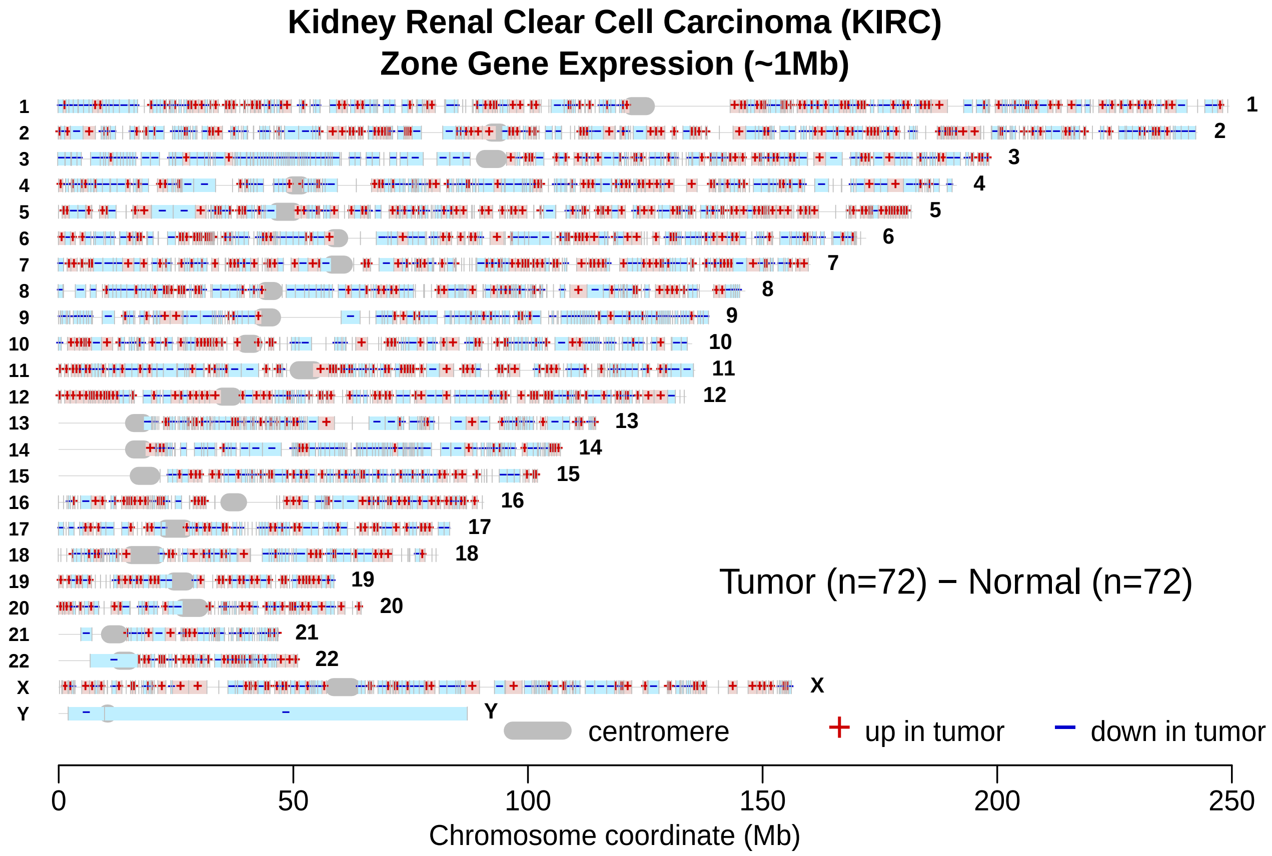

b

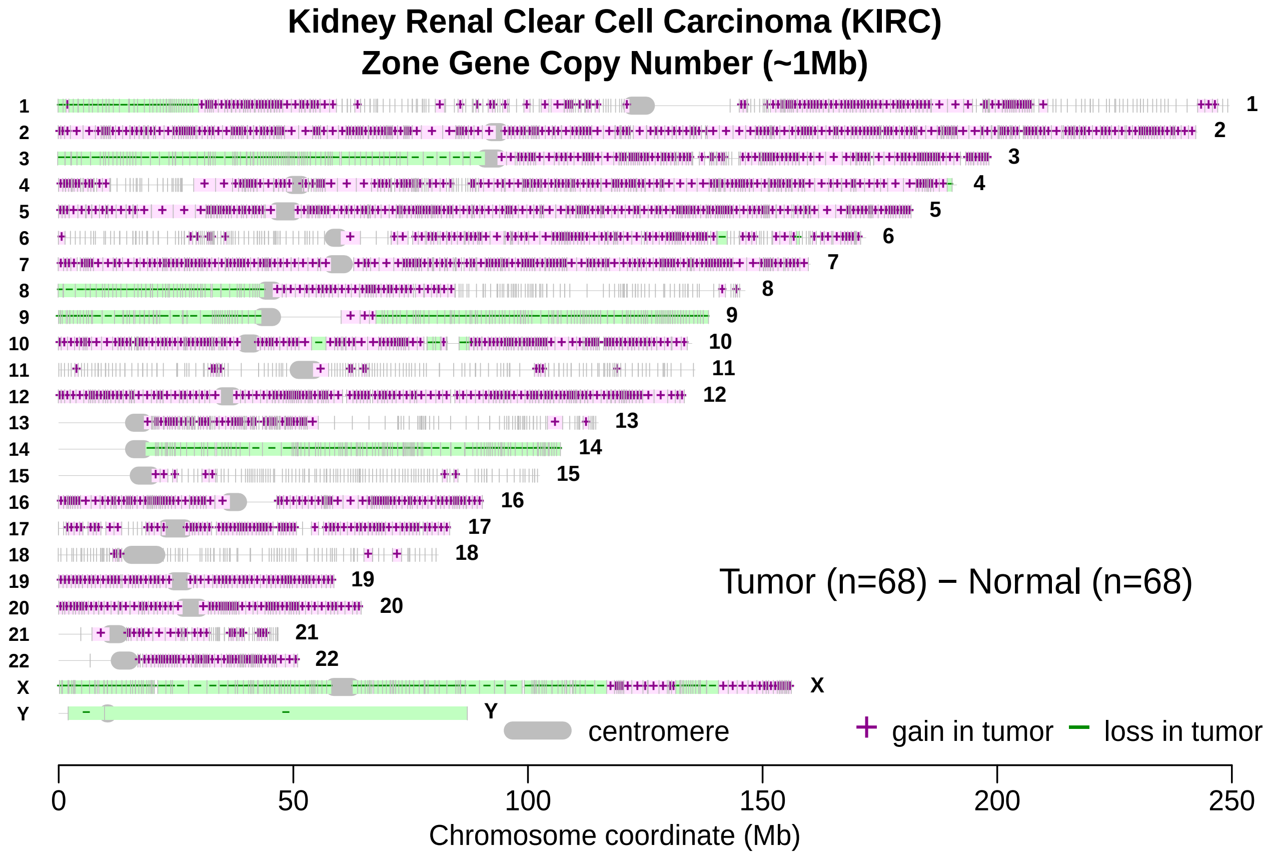

Supplementary Figure S2.8: Maps of genomic zone in KIRC. **a**, Polarization of zone regulation. **b**, Polarization of zone somatic copy number alteration. See the full legend on page 1.

a

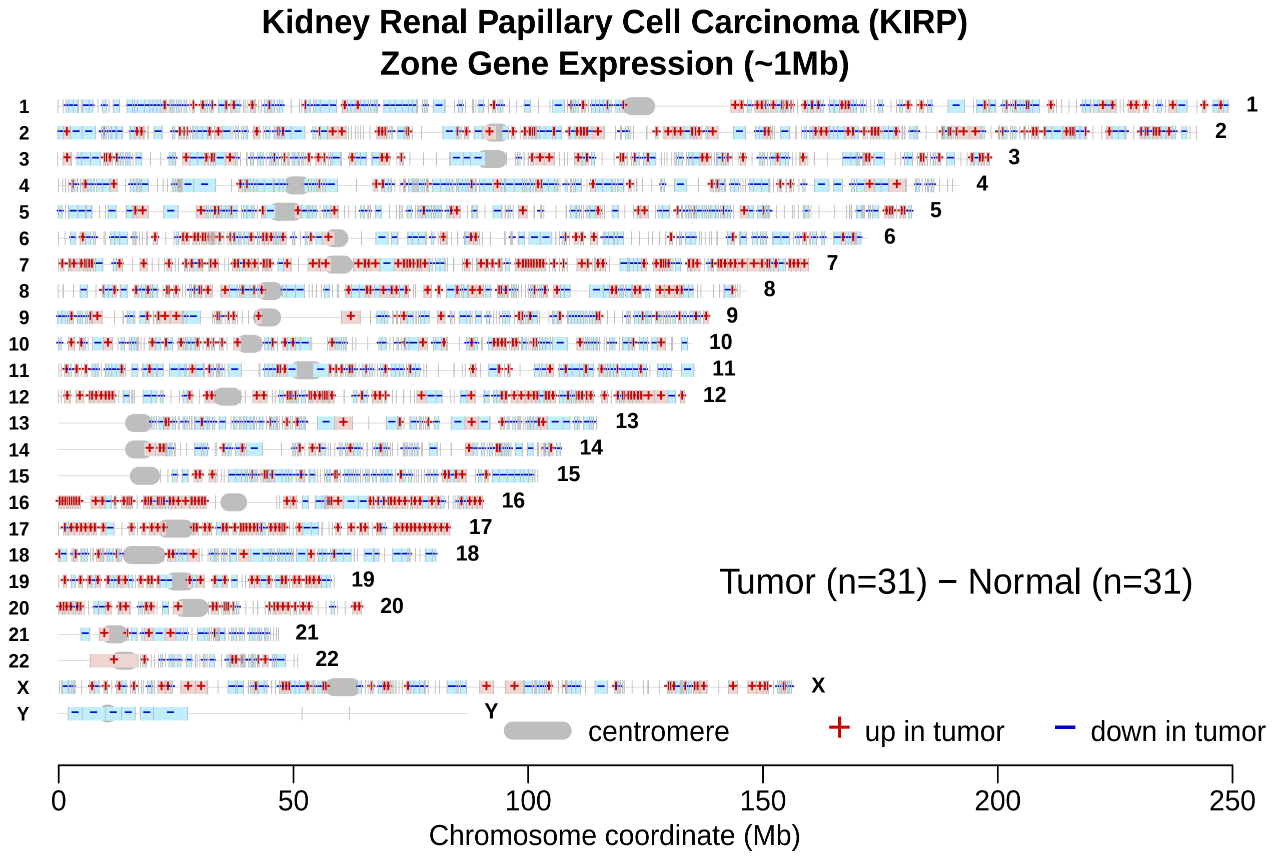

b

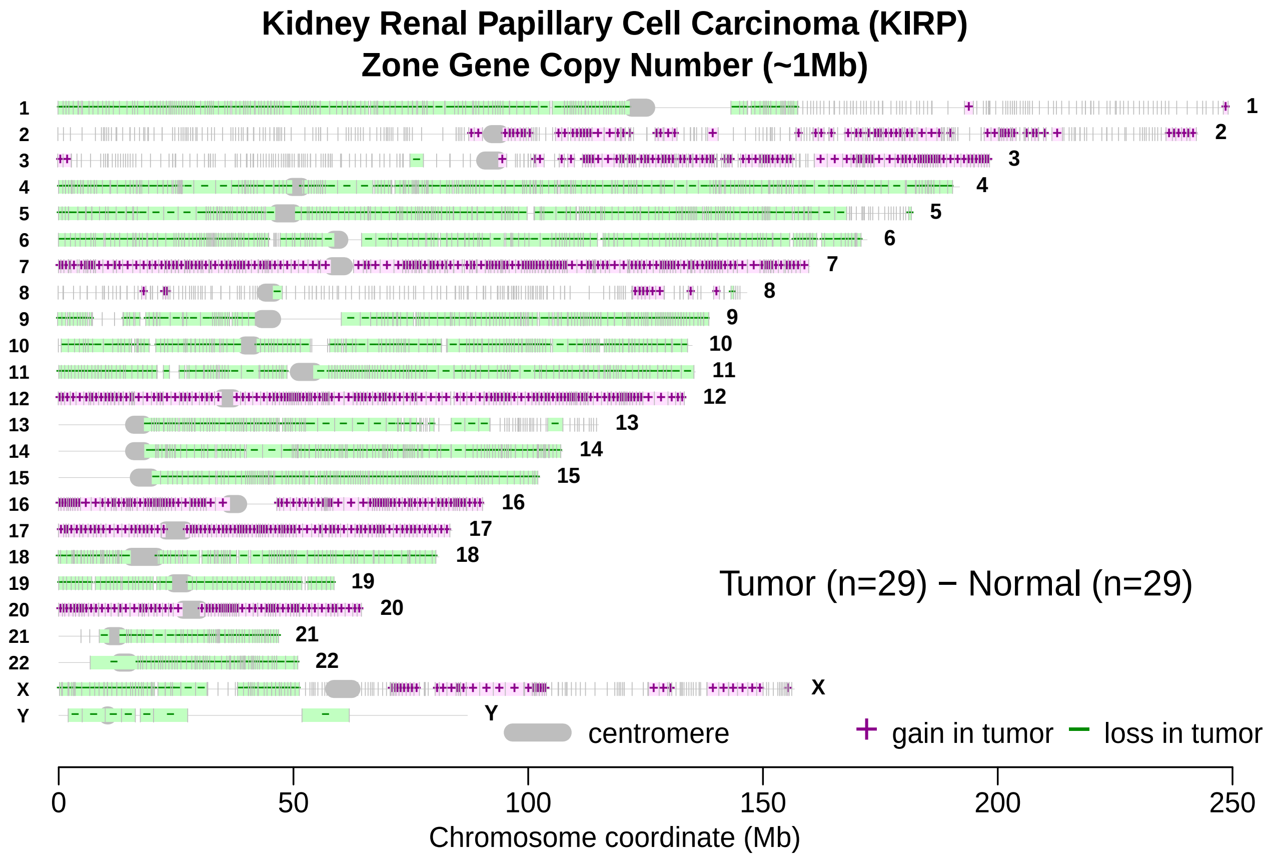

Supplementary Figure S2.9: Maps of genomic zone in KIRP. **a**, Polarization of zone regulation. **b**, Polarization of zone somatic copy number alteration. See the full legend on page 1.

a

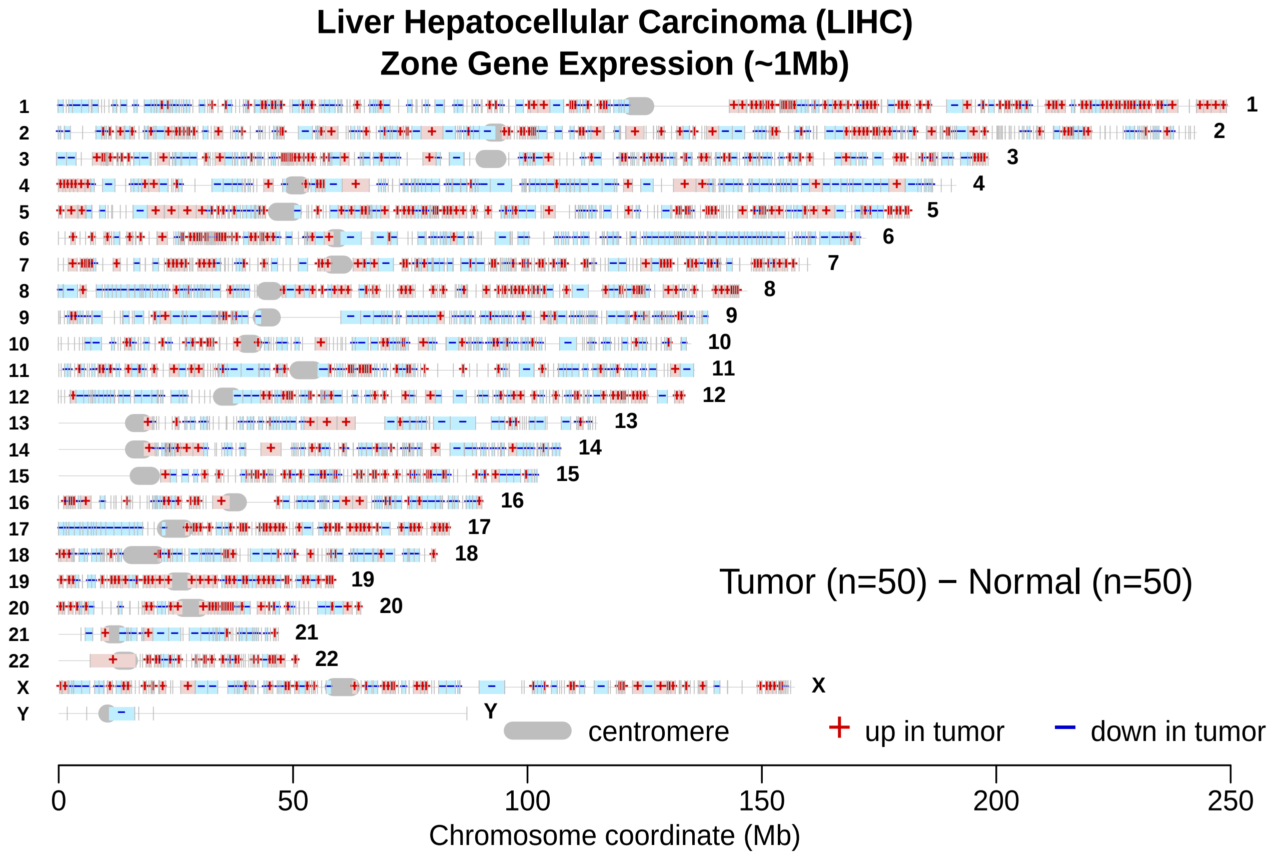

b

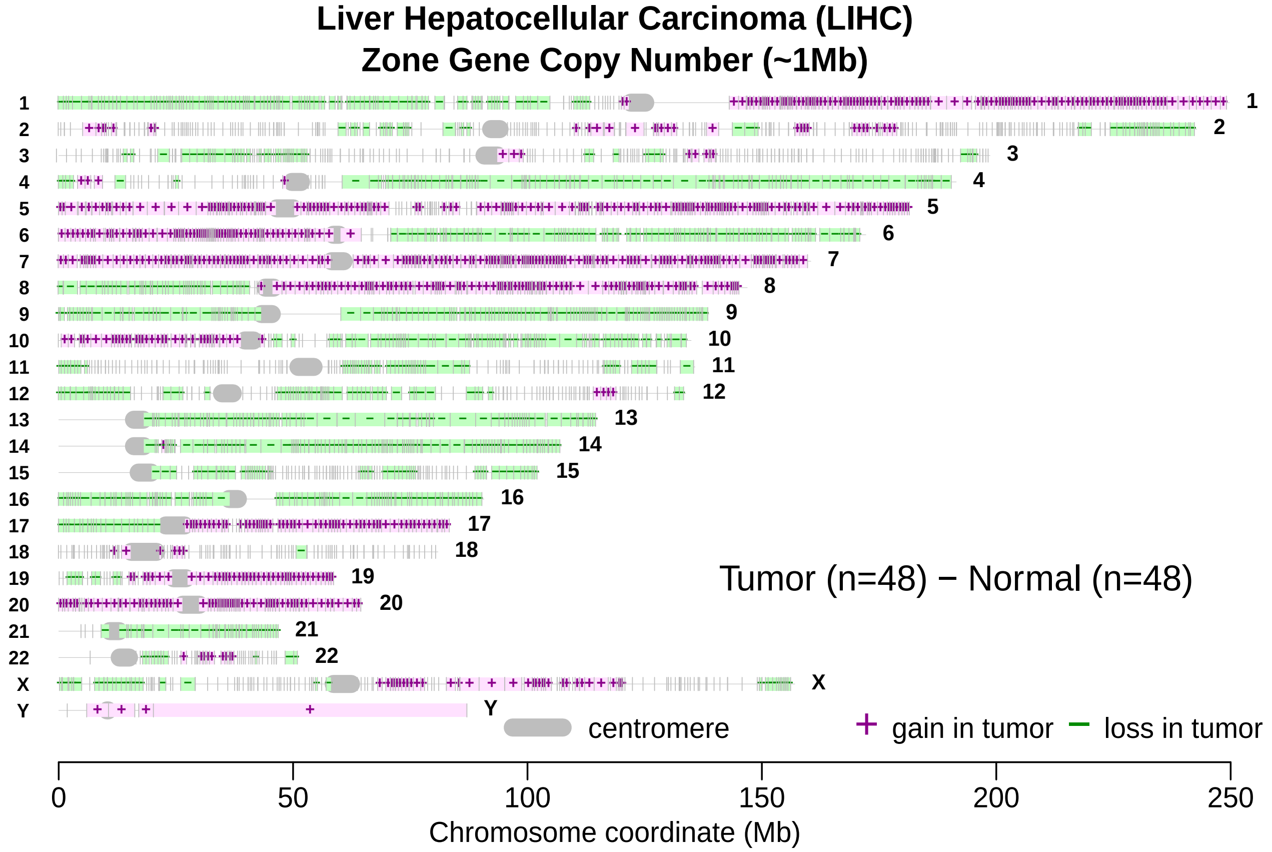

Supplementary Figure S2.10: Maps of genomic zone in LIHC. **a**, Polarization of zone regulation. **b**, Polarization of zone somatic copy number alteration. See the full legend on page 1.

a

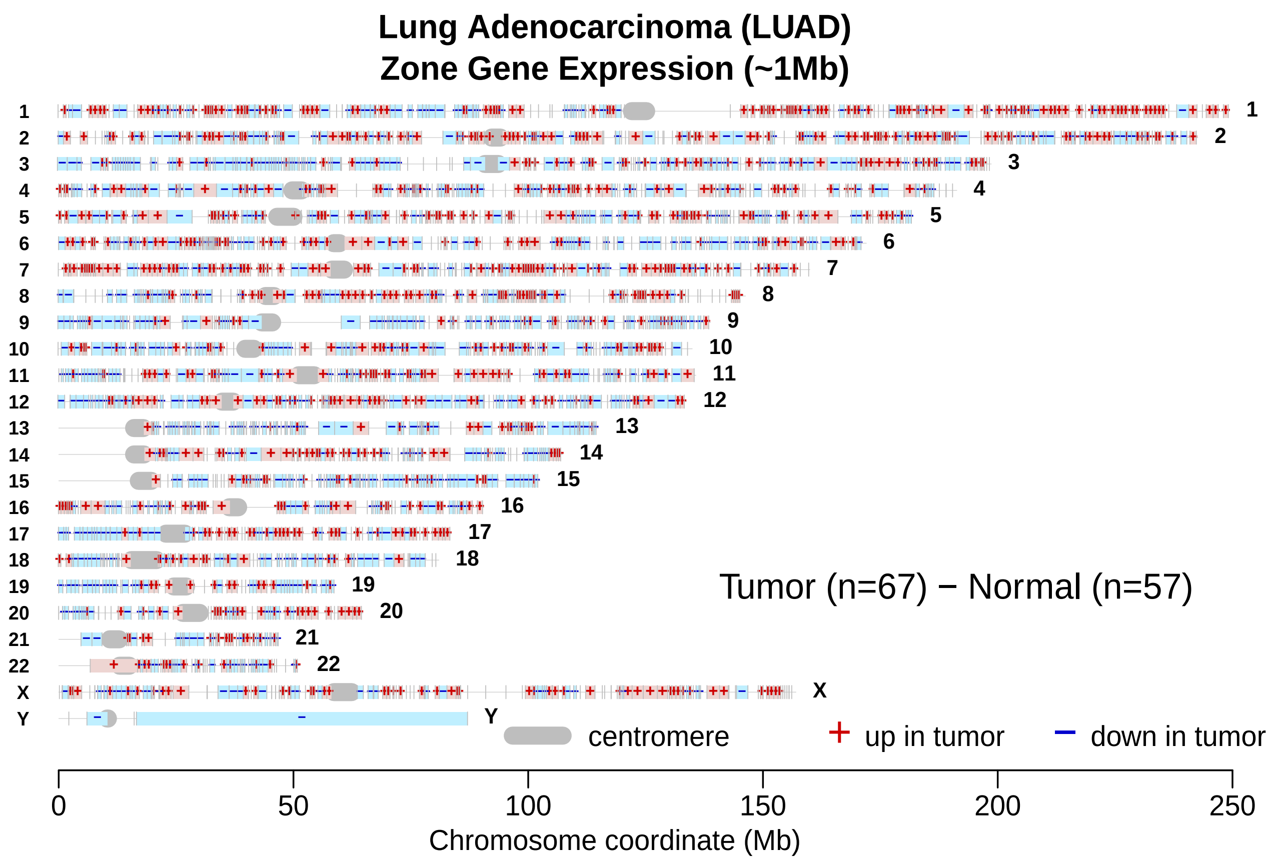

b

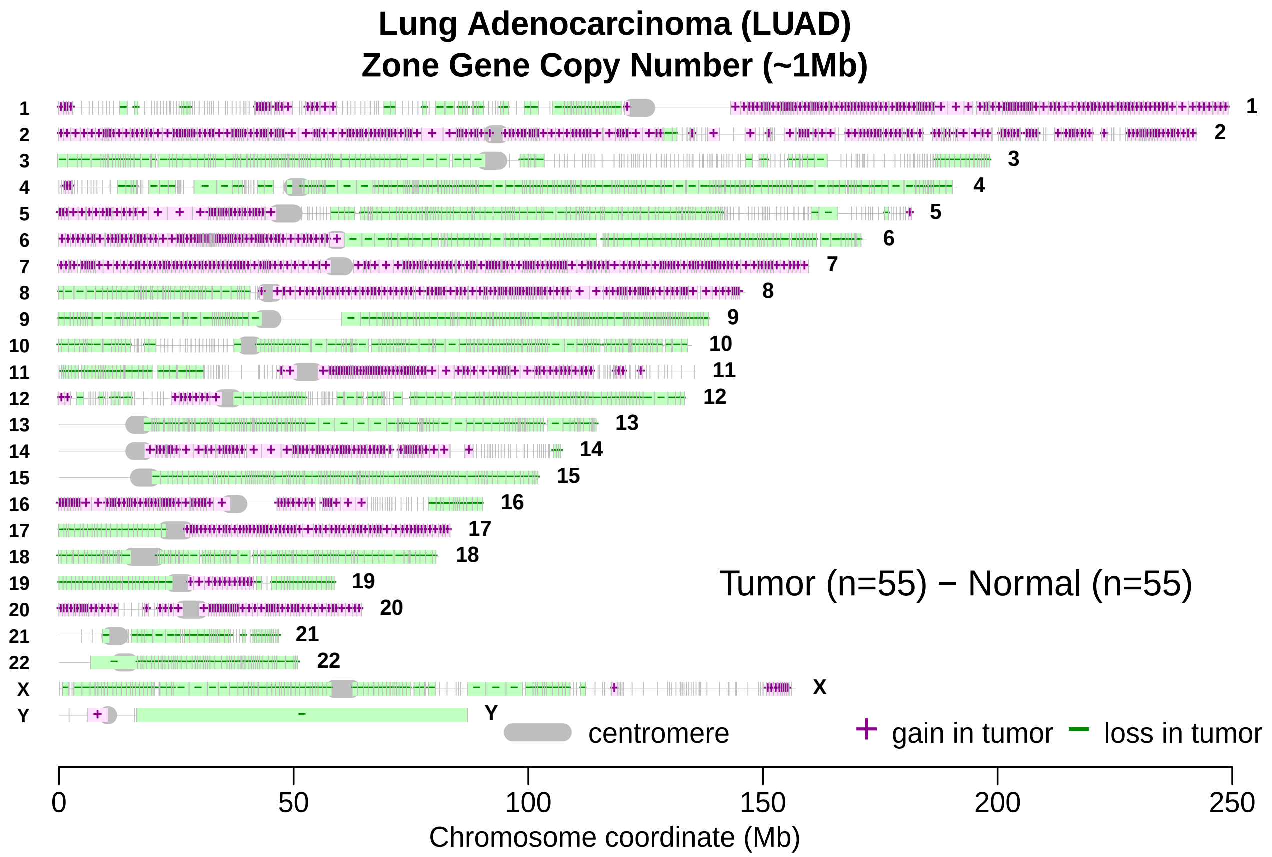

Supplementary Figure S2.11: Maps of genomic zone in LUAD. **a**, Polarization of zone regulation. **b**, Polarization of zone somatic copy number alteration. See the full legend on page 1.

a

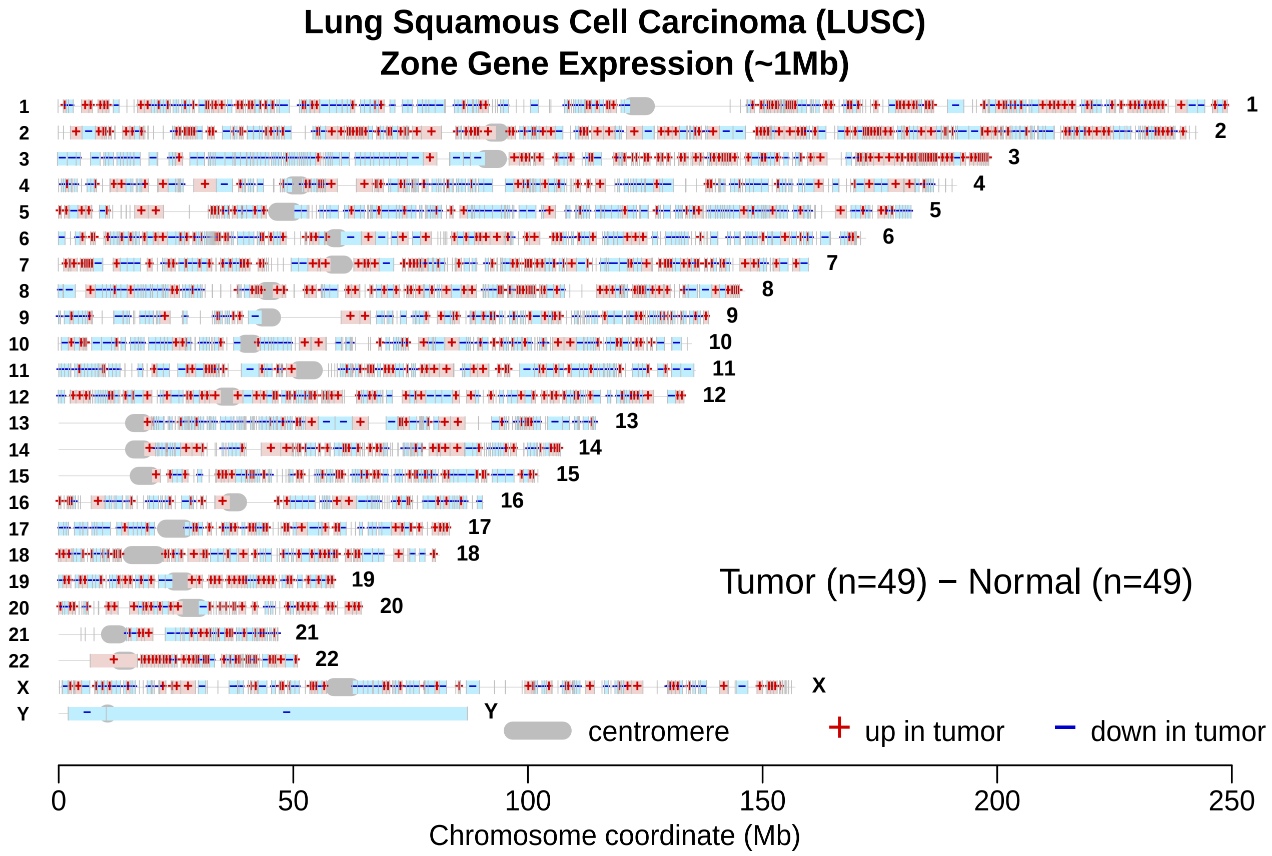

b

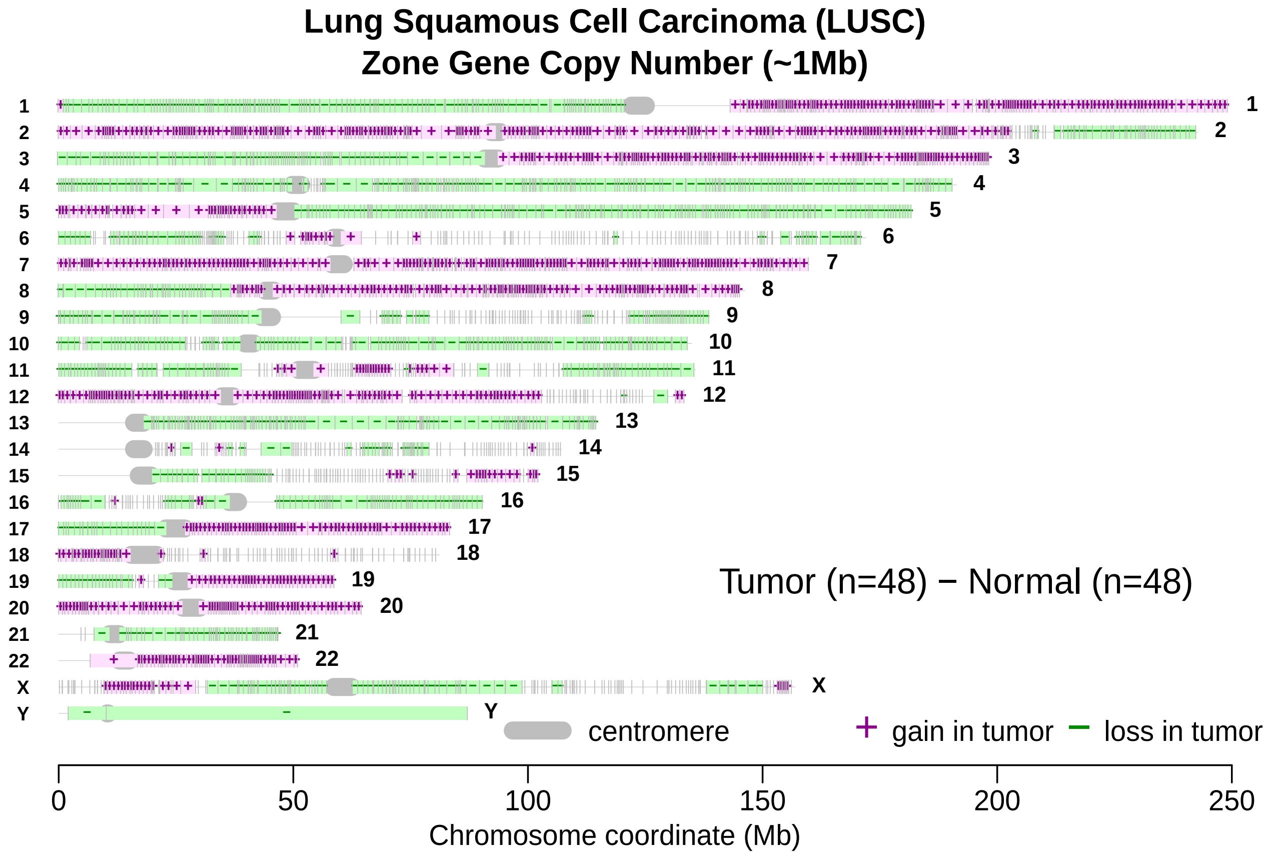

Supplementary Figure S2.12: Maps of genomic zone in LUSC. **a**, Polarization of zone regulation. **b**, Polarization of zone somatic copy number alteration. See the full legend on page 1.

a

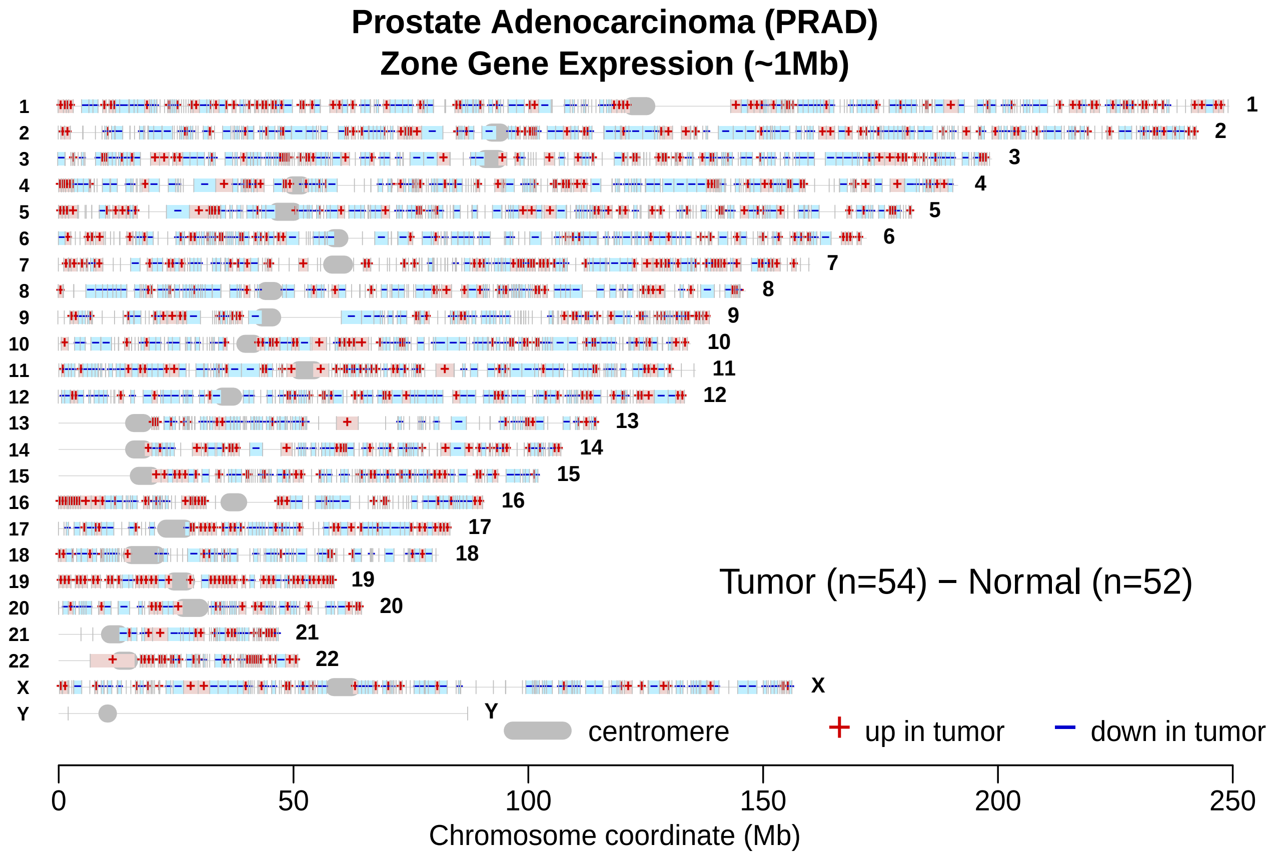

b

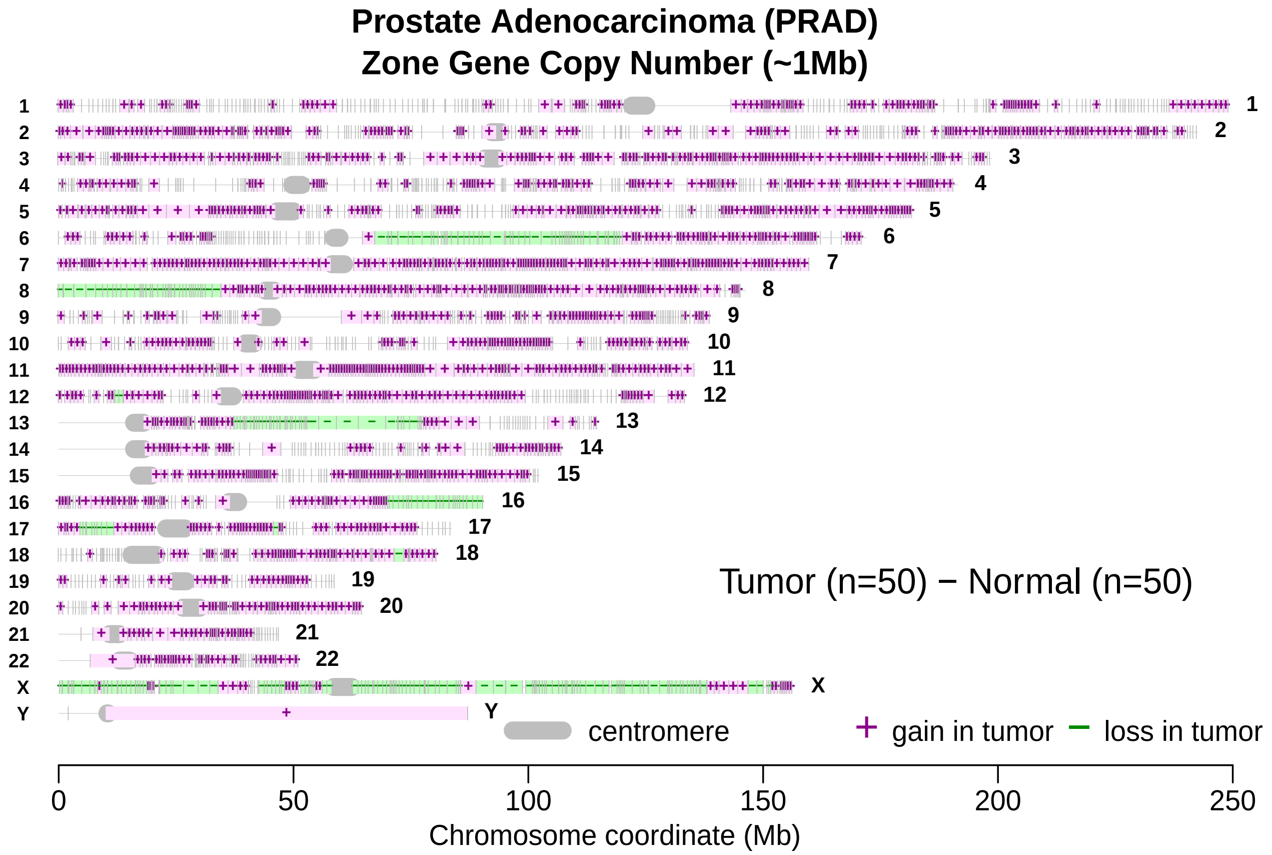

Supplementary Figure S2.13: Maps of genomic zone in PRAD. **a**, Polarization of zone regulation. **b**, Polarization of zone somatic copy number alteration. See the full legend on page 1.

a

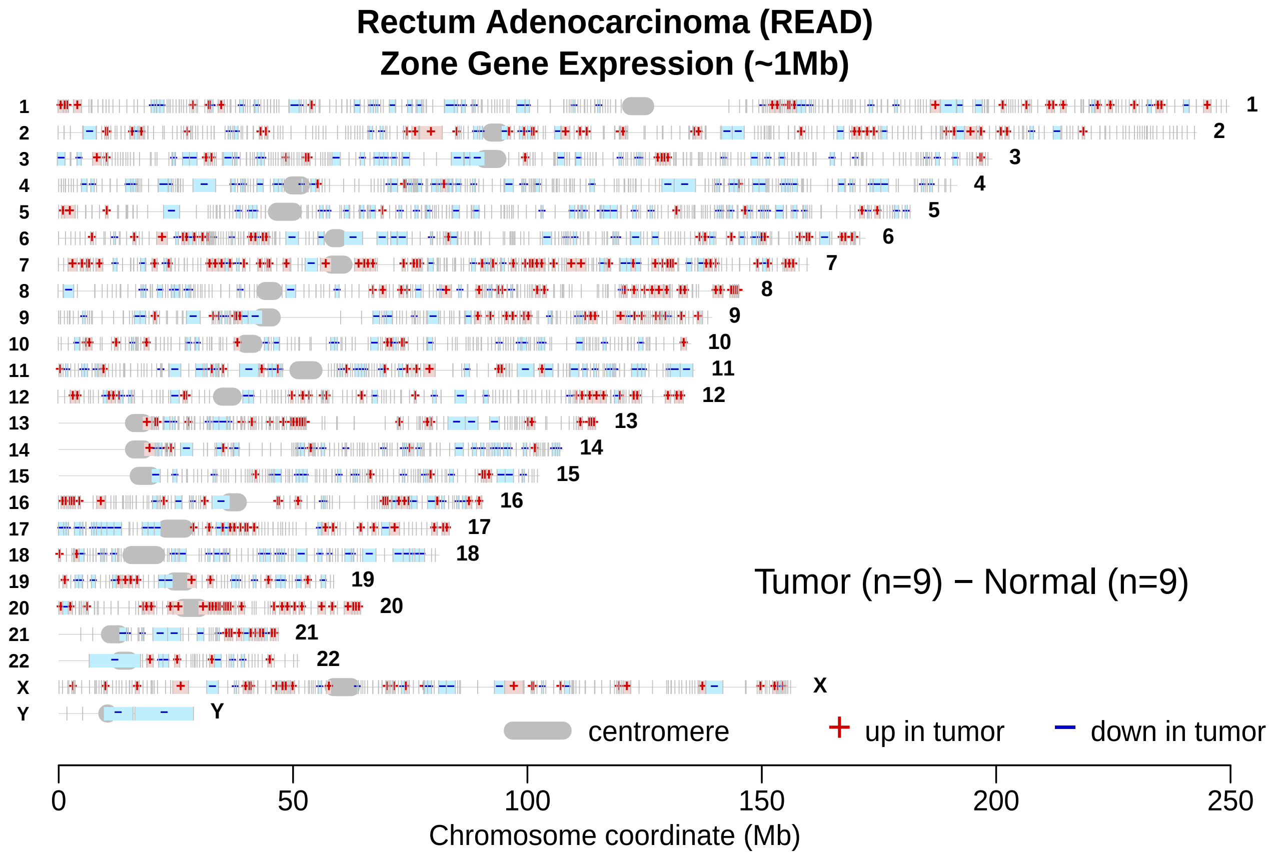

b

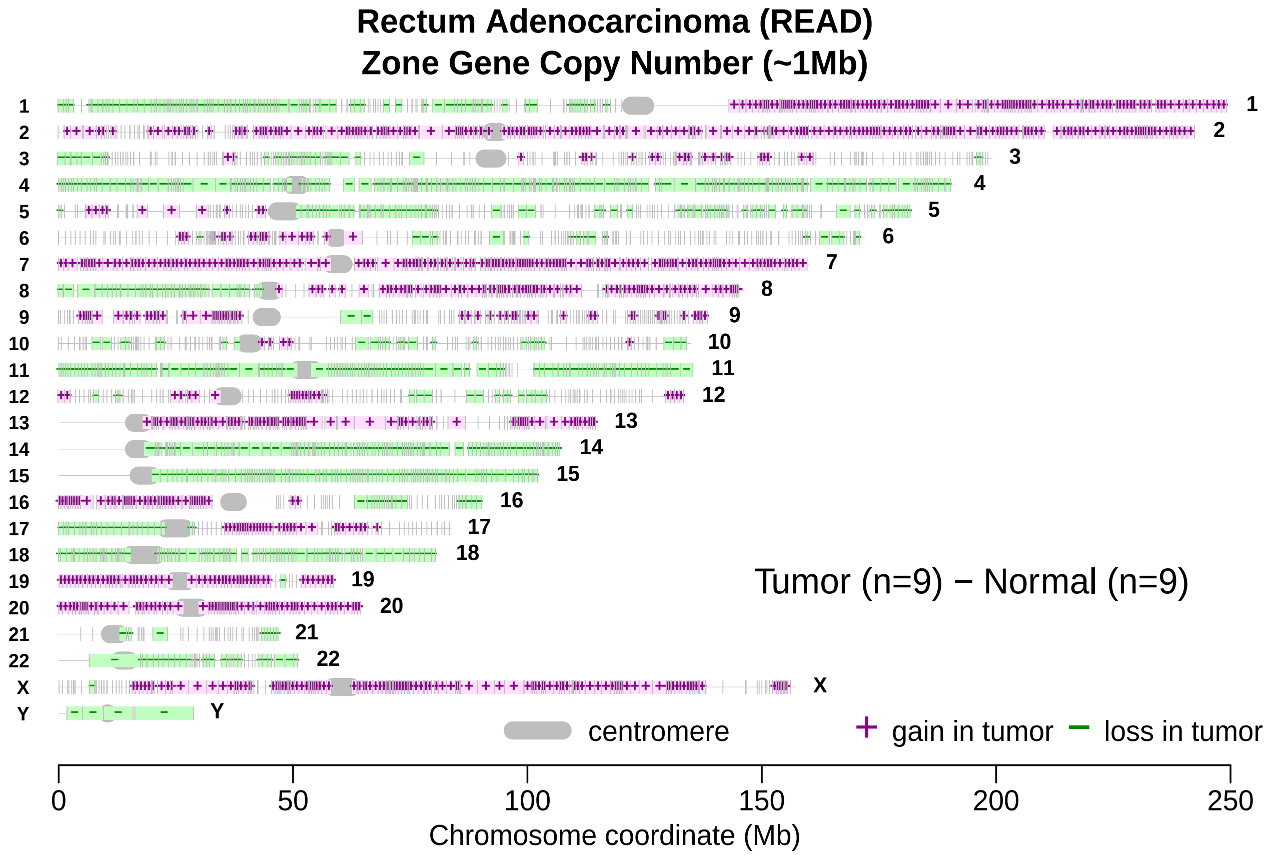

Supplementary Figure S2.14: Maps of genomic zone in READ. **a**, Polarization of zone regulation. **b**, Polarization of zone somatic copy number alteration. See the full legend on page 1.

a

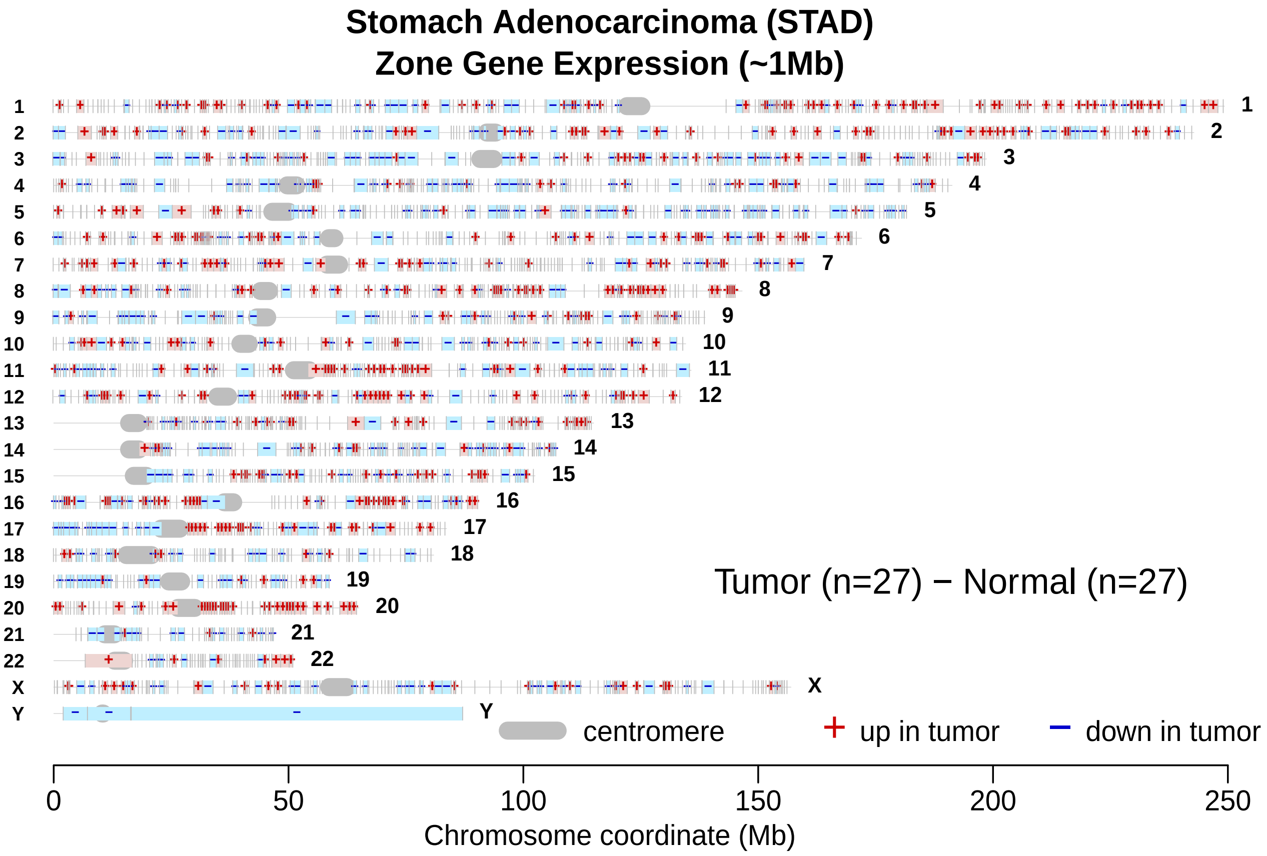

b

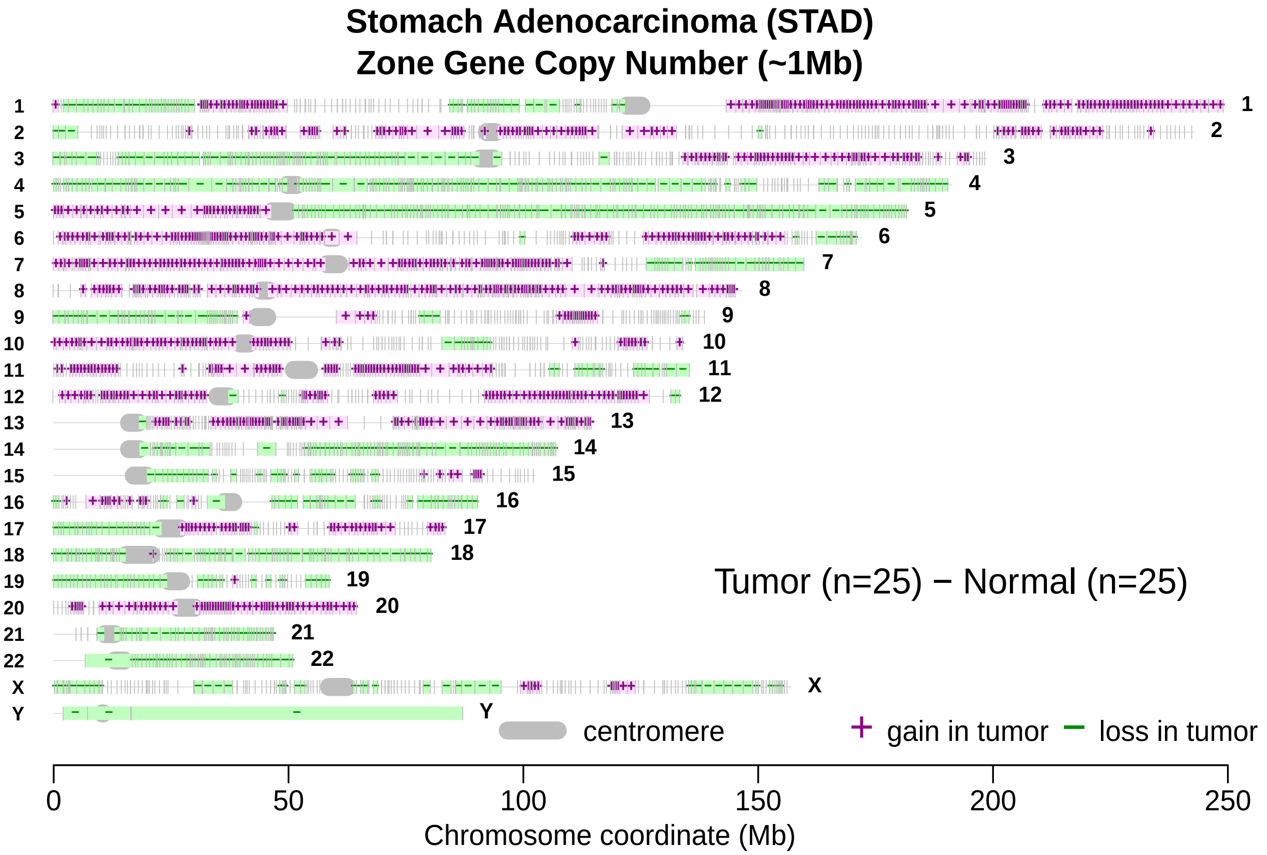

Supplementary Figure S2.15: Maps of genomic zone in STAD. **a**, Polarization of zone regulation. **b**, Polarization of zone somatic copy number alteration. See the full legend on page 1.

a

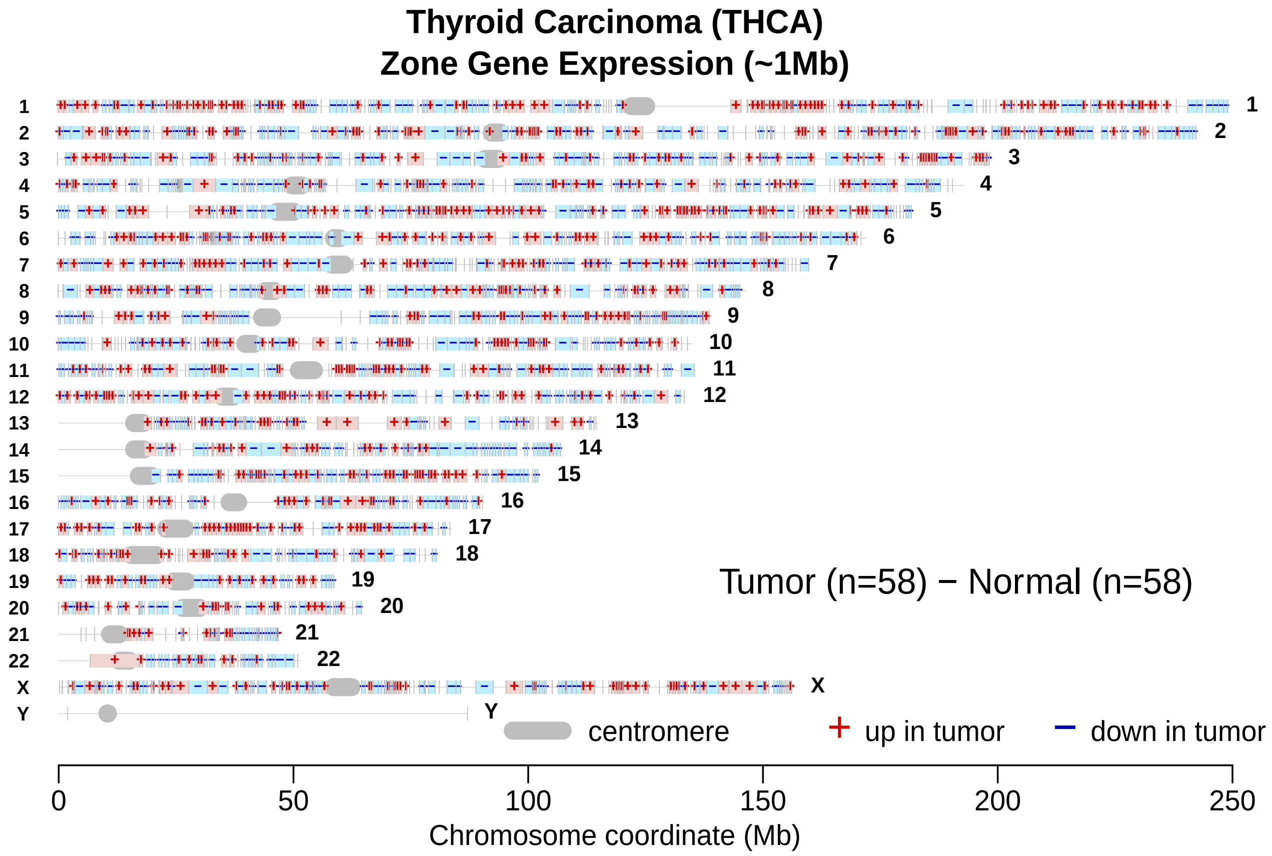

b

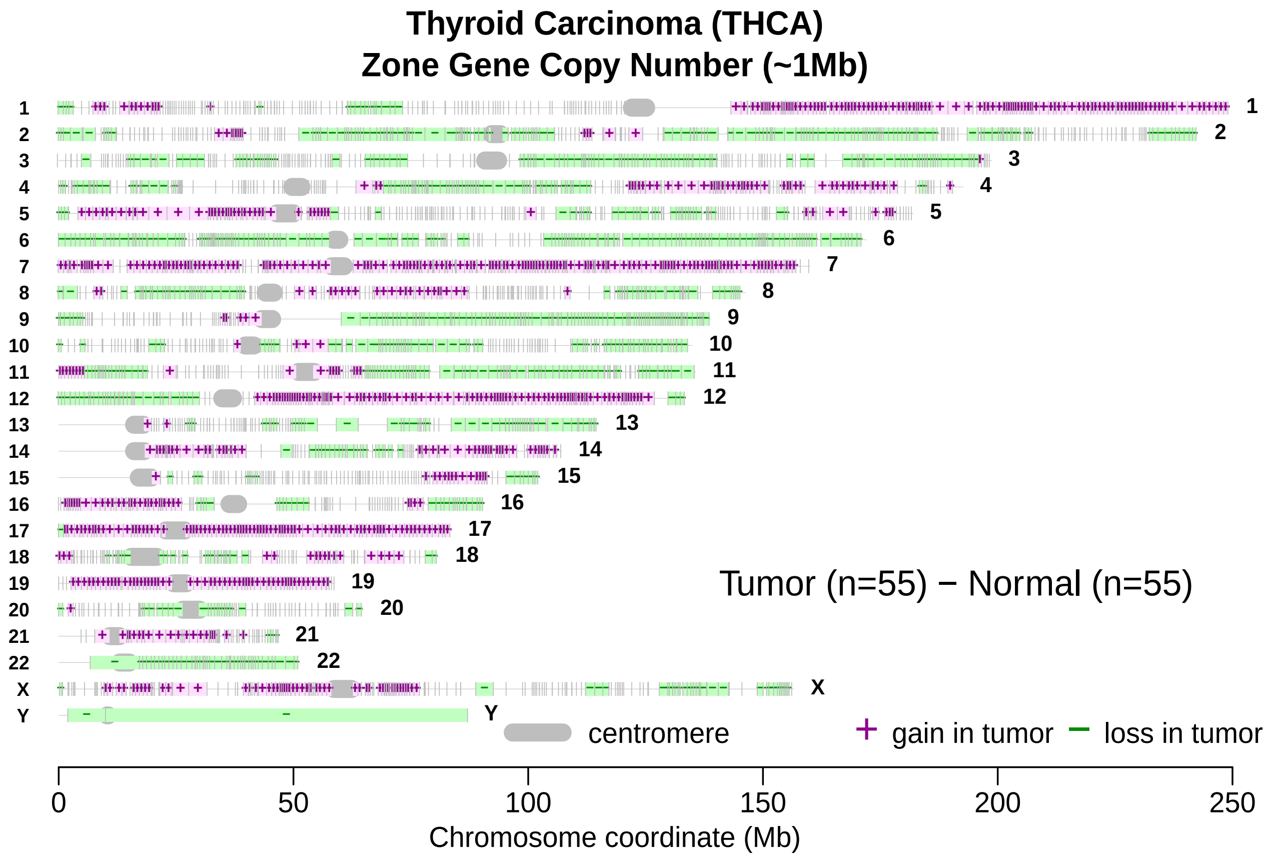

Supplementary Figure S2.16: Maps of genomic zone in THCA. **a**, Polarization of zone regulation. **b**, Polarization of zone somatic copy number alteration. See the full legend on page 1.

a

Uterine Corpus Endometrial Carcinoma (UCEC)
Zone Gene Expression (~1Mb)

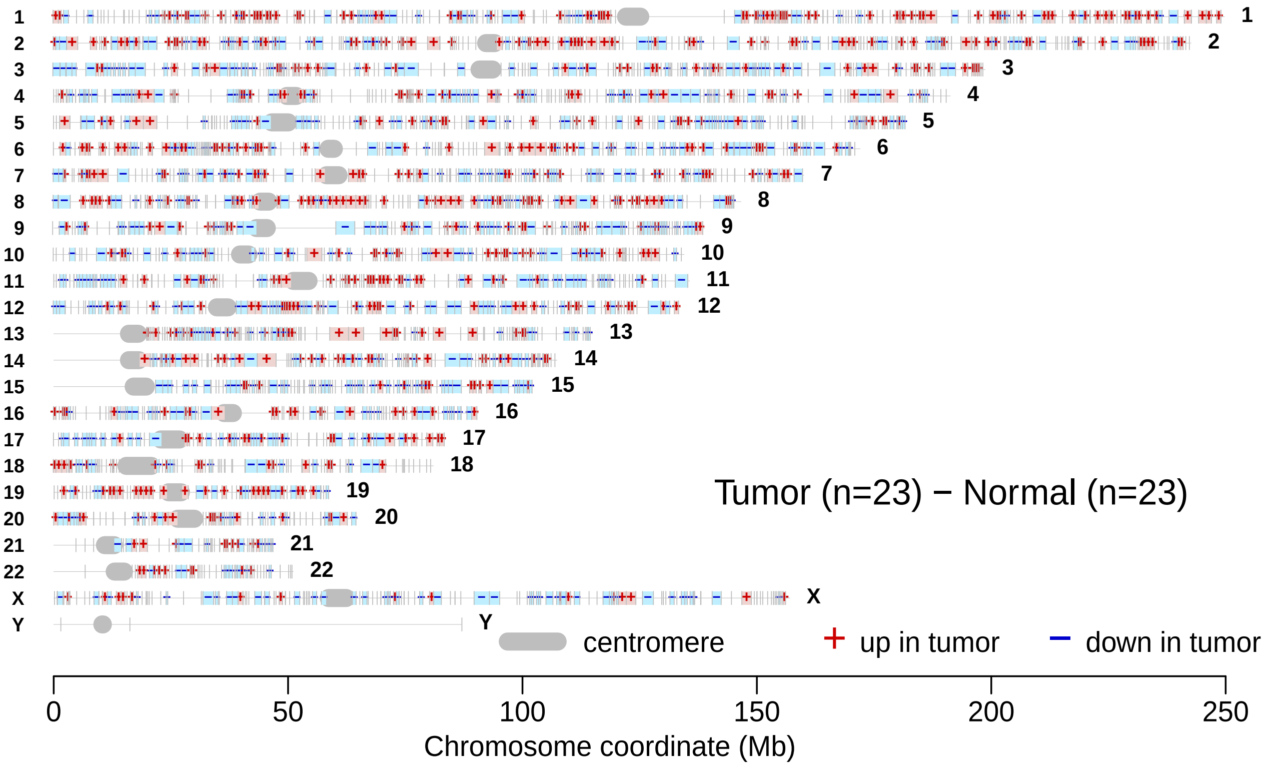

b

Uterine Corpus Endometrial Carcinoma (UCEC)
Zone Gene Copy Number (~1Mb)

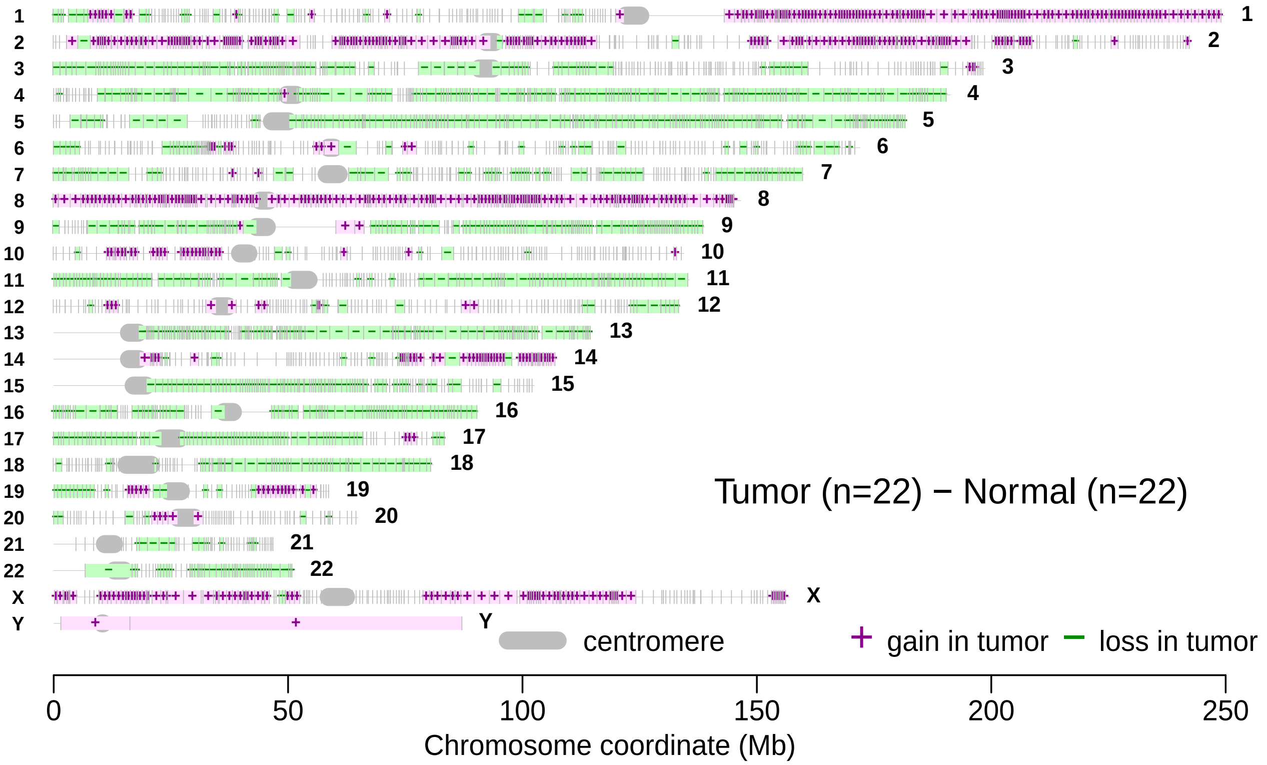

Supplementary Figure S2.17: Maps of genomic zone in UCEC. **a**, Polarization of zone regulation. **b**, Polarization of zone somatic copy number alteration. See the full legend on page 1.
