# Supplementary material for: Efficient weighted univariate clustering maps outstanding dysregulated genomic zones in human cancers
Source: Bioinformatics. 2020 Jul 3;36(20):5027–36. doi: 10.1093/bioinformatics/btaa613 (PMC7755420; doi:10.1093/bioinformatics/btaa613)
Supplement: btaa613_Supplementary_Data [file btaa613_supplementary_data.zip › SuppFig-S3.pdf]

Supplementary Figure S3: Top five most polarized regulation zones for each of 17 cancer types

List of Figures

S3.1 Most polarized zone in regulation in BLCA S3-4
S3.2 Most polarized zone in regulation in BRCA S3-5
S3.3 Most polarized zone in regulation in CHOL S3-6
S3.4 Most polarized zone in regulation in COAD S3-7
S3.5 Most polarized zone in regulation in ESCA S3-8
S3.6 Most polarized zone in regulation in HNSC S3-9
S3.7 Most polarized zone in regulation in KICH S3-10
S3.8 Most polarized zone in regulation in KIRC S3-11
S3.9 Most polarized zone in regulation in KIRP S3-12
S3.10 Most polarized zone in regulation in LIHC S3-13
S3.11 Most polarized zone in regulation in LUAD S3-14
S3.12 Most polarized zone in regulation in LUSC S3-15
S3.13 Most polarized zone in regulation in PRAD S3-16
S3.14 Most polarized zone in regulation in READ S3-17
S3.15 Most polarized zone in regulation in STAD S3-18
S3.16 Most polarized zone in regulation in THCA S3-19
S3.17 Most polarized zone in regulation in UCEC S3-20
S3.18 Second most polarized zone in regulation in BLCA S3-22
S3.19 Second most polarized zone in regulation in BRCA S3-23
S3.20 Second most polarized zone in regulation in CHOL S3-24
S3.21 Second most polarized zone in regulation in COAD S3-25
S3.22 Second most polarized zone in regulation in ESCA S3-26
S3.23 Second most polarized zone in regulation in HNSC S3-27
S3.24 Second most polarized zone in regulation in KICH S3-28
S3.25 Second most polarized zone in regulation in KIRC S3-29
S3.26 Second most polarized zone in regulation in KIRP S3-30
S3.27 Second most polarized zone in regulation in LIHC S3-31
S3.28 Second most polarized zone in regulation in LUAD S3-32
S3.29 Second most polarized zone in regulation in LUSC S3-33
S3.30 Second most polarized zone in regulation in PRAD S3-34
S3.31 Second most polarized zone in regulation in READ S3-35
S3.32 Second most polarized zone in regulation in STAD S3-36
S3.33 Second most polarized zone in regulation in THCA S3-37
S3.34 Second most polarized zone in regulation in UCEC S3-38
S3.35 Third most polarized zone in regulation in BLCA S3-40
S3.36 Third most polarized zone in regulation in BRCA S3-41
S3.37 Third most polarized zone in regulation in CHOL S3-42
S3.38 Third most polarized zone in regulation in COAD S3-43
S3.39 Third most polarized zone in regulation in ESCA S3-44
S3.40 Third most polarized zone in regulation in HNSC S3-45
S3.41 Third most polarized zone in regulation in KICH S3-46
S3.42 Third most polarized zone in regulation in KIRC S3-47

S3.43 Third most polarized zone in regulation in KIRP S3-48
S3.44 Third most polarized zone in regulation in LIHC S3-49
S3.45 Third most polarized zone in regulation in LUAD S3-50
S3.46 Third most polarized zone in regulation in LUSC S3-51
S3.47 Third most polarized zone in regulation in PRAD S3-52
S3.48 Third most polarized zone in regulation in READ S3-53
S3.49 Third most polarized zone in regulation in STAD S3-54
S3.50 Third most polarized zone in regulation in THCA S3-55
S3.51 Third most polarized zone in regulation in UCEC S3-56
S3.52 Fourth most polarized zone in regulation in BLCA S3-58
S3.53 Fourth most polarized zone in regulation in BRCA S3-59
S3.54 Fourth most polarized zone in regulation in CHOL S3-60
S3.55 Fourth most polarized zone in regulation in COAD S3-61
S3.56 Fourth most polarized zone in regulation in ESCA S3-62
S3.57 Fourth most polarized zone in regulation in HNSC S3-63
S3.58 Fourth most polarized zone in regulation in KICH S3-64
S3.59 Fourth most polarized zone in regulation in KIRC S3-65
S3.60 Fourth most polarized zone in regulation in KIRP S3-66
S3.61 Fourth most polarized zone in regulation in LIHC S3-67
S3.62 Fourth most polarized zone in regulation in LUAD S3-68
S3.63 Fourth most polarized zone in regulation in LUSC S3-69
S3.64 Fourth most polarized zone in regulation in PRAD S3-70
S3.65 Fourth most polarized zone in regulation in READ S3-71
S3.66 Fourth most polarized zone in regulation in STAD S3-72
S3.67 Fourth most polarized zone in regulation in THCA S3-73
S3.68 Fourth most polarized zone in regulation in UCEC S3-74
S3.69 Fifth most polarized zone in regulation in BLCA S3-76
S3.70 Fifth most polarized zone in regulation in BRCA S3-77
S3.71 Fifth most polarized zone in regulation in CHOL S3-78
S3.72 Fifth most polarized zone in regulation in COAD S3-79
S3.73 Fifth most polarized zone in regulation in ESCA S3-80
S3.74 Fifth most polarized zone in regulation in HNSC S3-81
S3.75 Fifth most polarized zone in regulation in KICH S3-82
S3.76 Fifth most polarized zone in regulation in KIRC S3-83
S3.77 Fifth most polarized zone in regulation in KIRP S3-84
S3.78 Fifth most polarized zone in regulation in LIHC S3-85
S3.79 Fifth most polarized zone in regulation in LUAD S3-86
S3.80 Fifth most polarized zone in regulation in LUSC S3-87
S3.81 Fifth most polarized zone in regulation in PRAD S3-88
S3.82 Fifth most polarized zone in regulation in READ S3-89
S3.83 Fifth most polarized zone in regulation in STAD S3-90
S3.84 Fifth most polarized zone in regulation in THCA S3-91
S3.85 Fifth most polarized zone in regulation in UCEC S3-92

Legend used in zone visualization

Each ‘x’ marks one patient with a pair of matched tumor-normal samples. Colors of the ‘x’ marks indicate different patients. The horizontal location of ‘x’ represents a unique gene.

Genes within a zone are ordered by their start genomic coordinates. They are equally spaced in the visualization within the zone, not linearly proportional to their genomic distance.

Known cancer genes from COSMIC Cancer Gene Census (CGC) version 87 and Network of Cancer Genes 6 are marked along the chromosomes. Those highly differentially regulated are marked in bold font. Six cancer loci from CGC—*IGH@*, *IGK@*, *IGL@*, *TRA@*, *TRB@*, and *TRD@*—are not marked, as they are not genes and thus no expression data is available.

a, The name of a gene is marked at its relative position only if its expression changed substantially between tumor and matched normal tissues. The color of a gene name text is red/blue for up-/down-regulation in cancer versus normal.

b, The name of a gene is marked at its relative position only if its copy number changed substantially between tumor and matched normal tissues. The color of a gene name text is purple/green for amplification/deletion in somatic copy number in cancer versus normal.

a

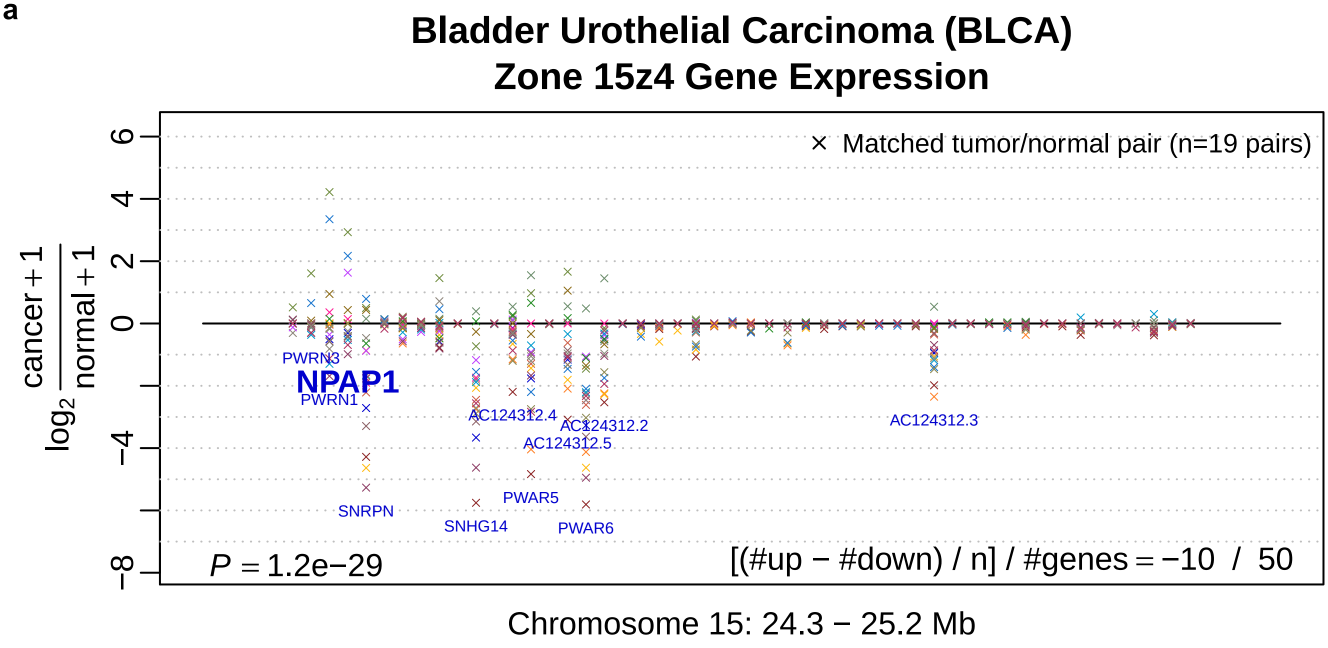

b

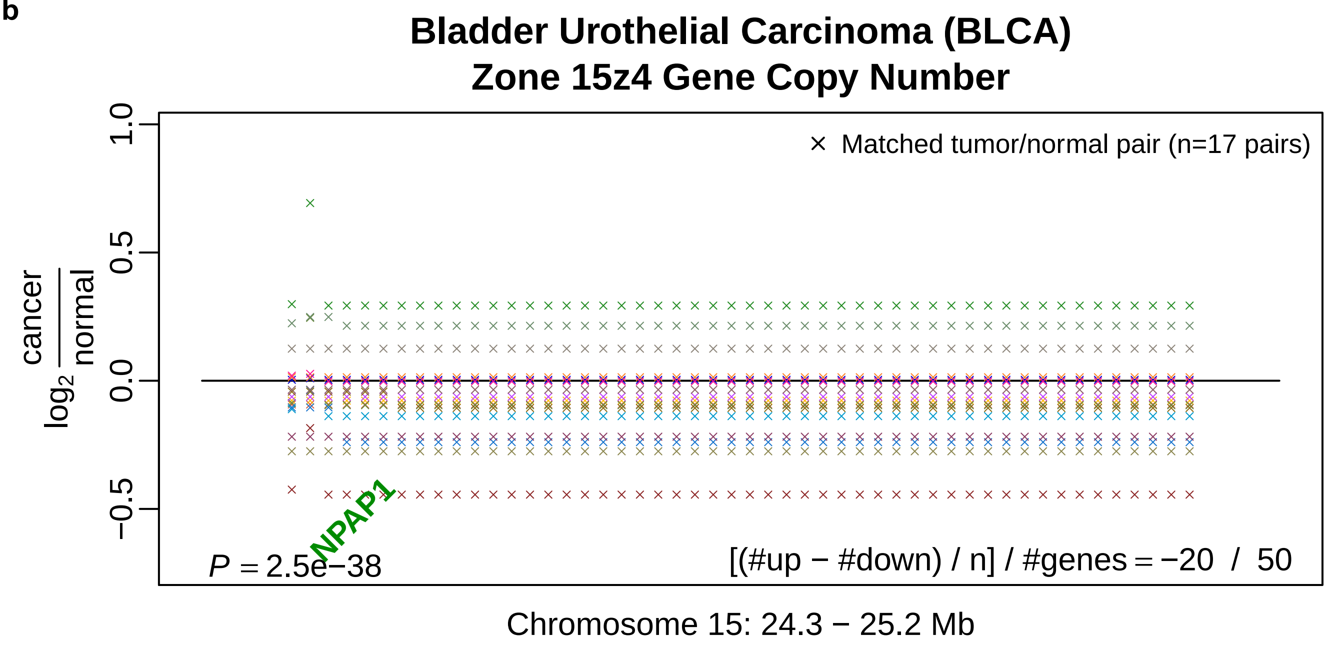

Supplementary Figure S3.1: The most statistically significant polarized regulation zone in BLCA. **a**, The gene expression log ratio of cancer to normal for each gene within the zone in each patient. **b**, The somatic copy number log ratio of cancer to normal for each gene within the zone in each patient. See the full legend on page 3.

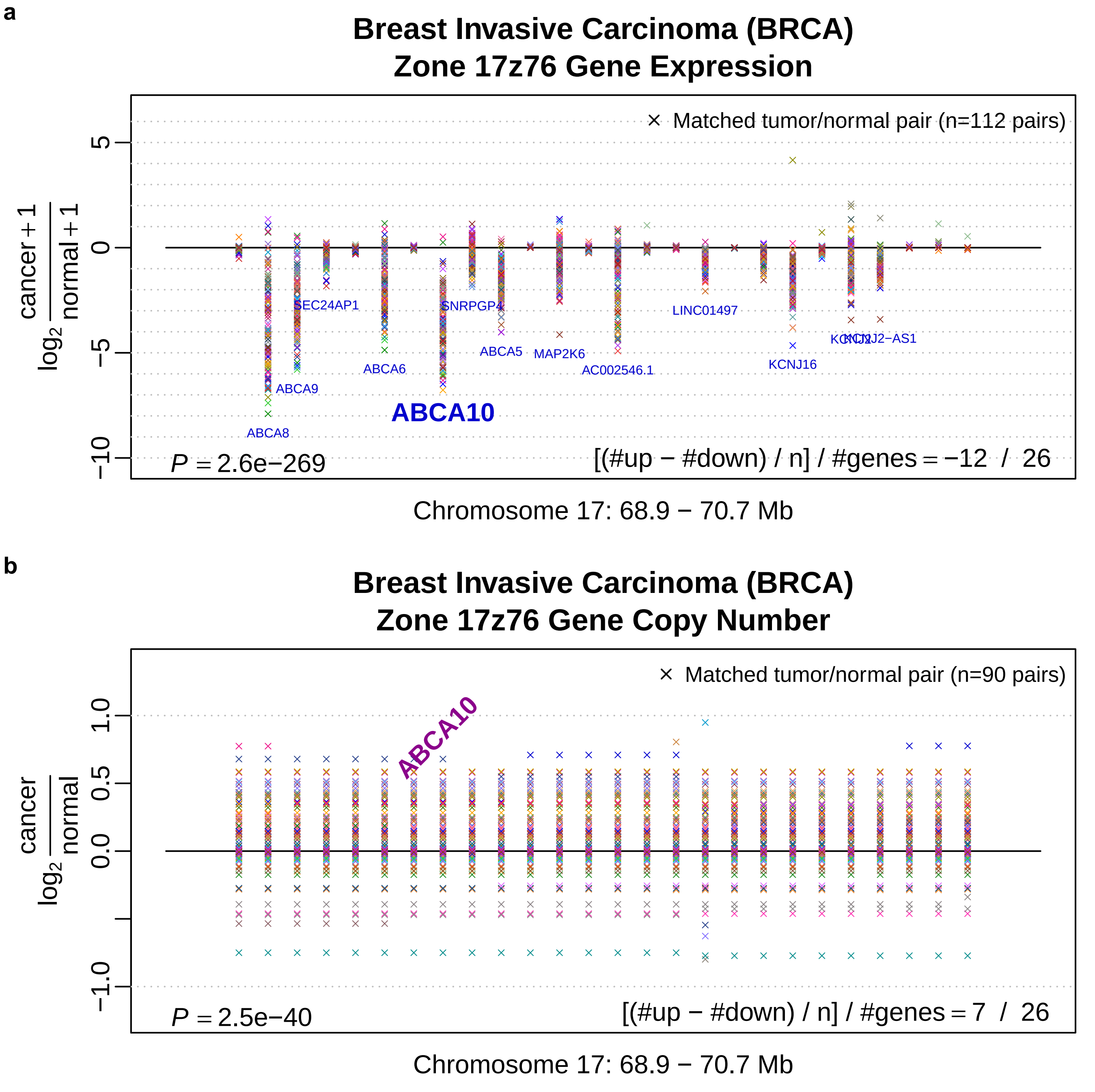

Supplementary Figure S3.2: The most statistically significant polarized regulation zone in BRCA. **a**, The gene expression log ratio of cancer to normal for each gene within the zone in each patient. **b**, The somatic copy number log ratio of cancer to normal for each gene within the zone in each patient. See the full legend on page 3.

a

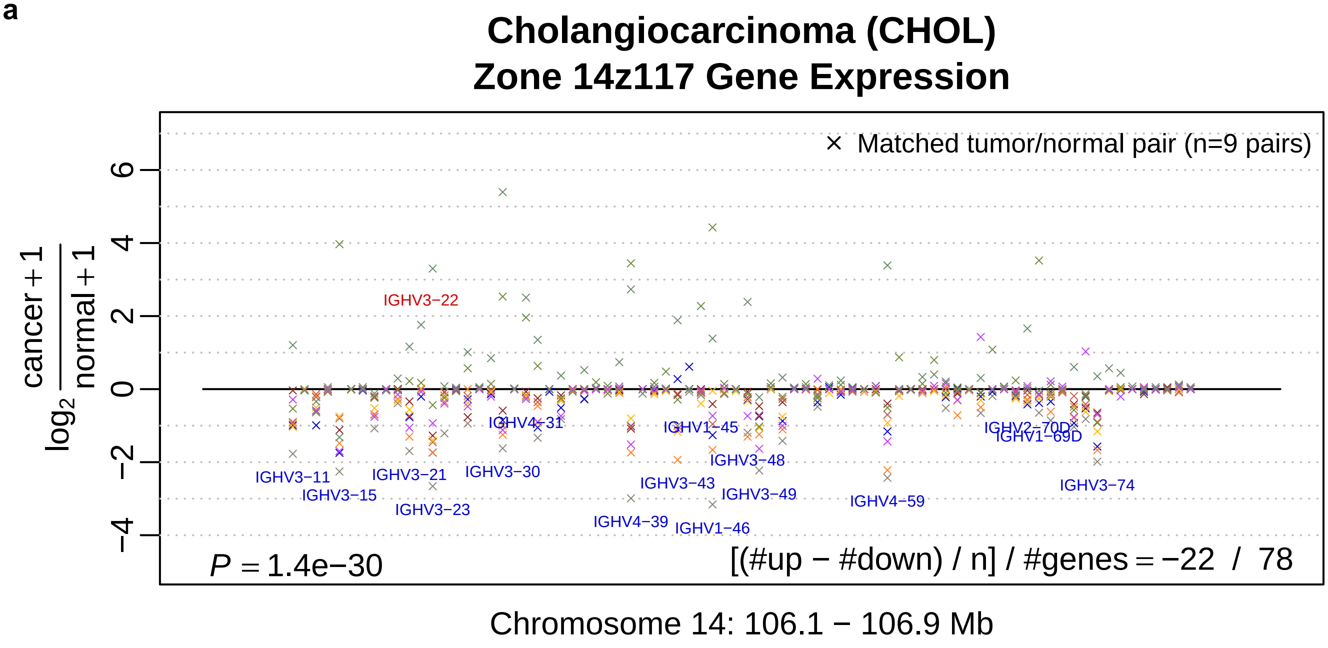

b

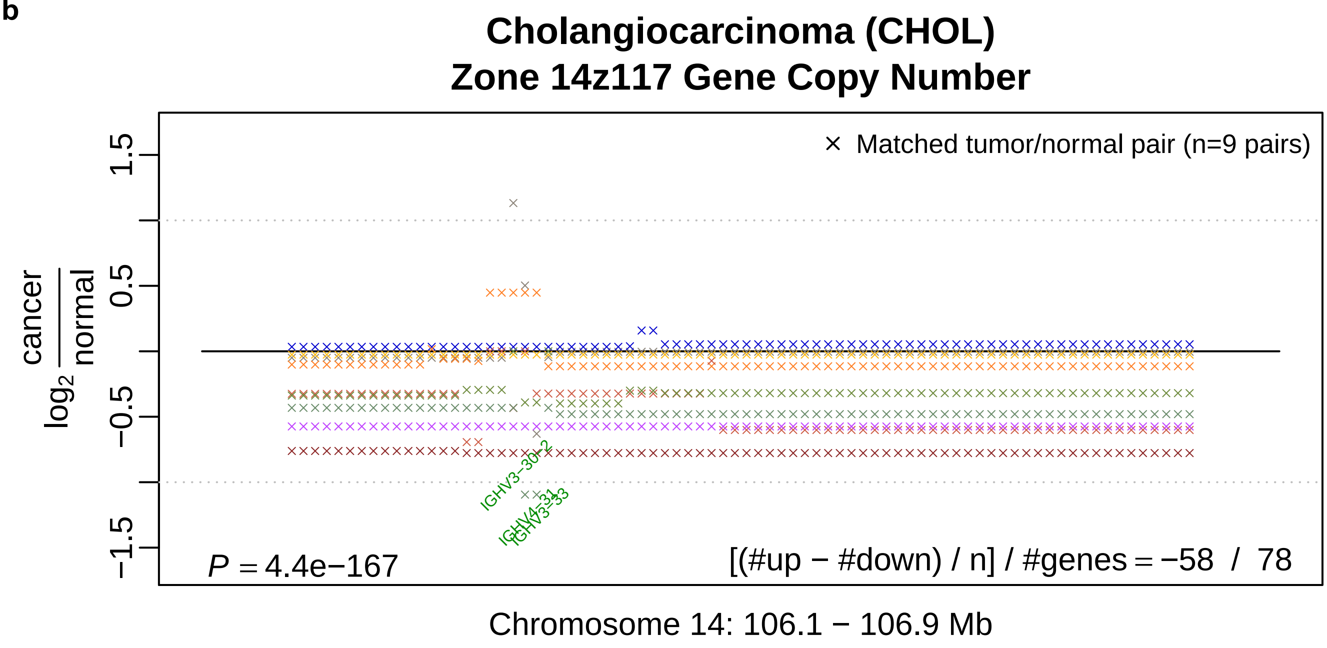

Supplementary Figure S3.3: The most statistically significant polarized regulation zone in CHOL. **a**, The gene expression log ratio of cancer to normal for each gene within the zone in each patient. **b**, The somatic copy number log ratio of cancer to normal for each gene within the zone in each patient. See the full legend on page 3.

a

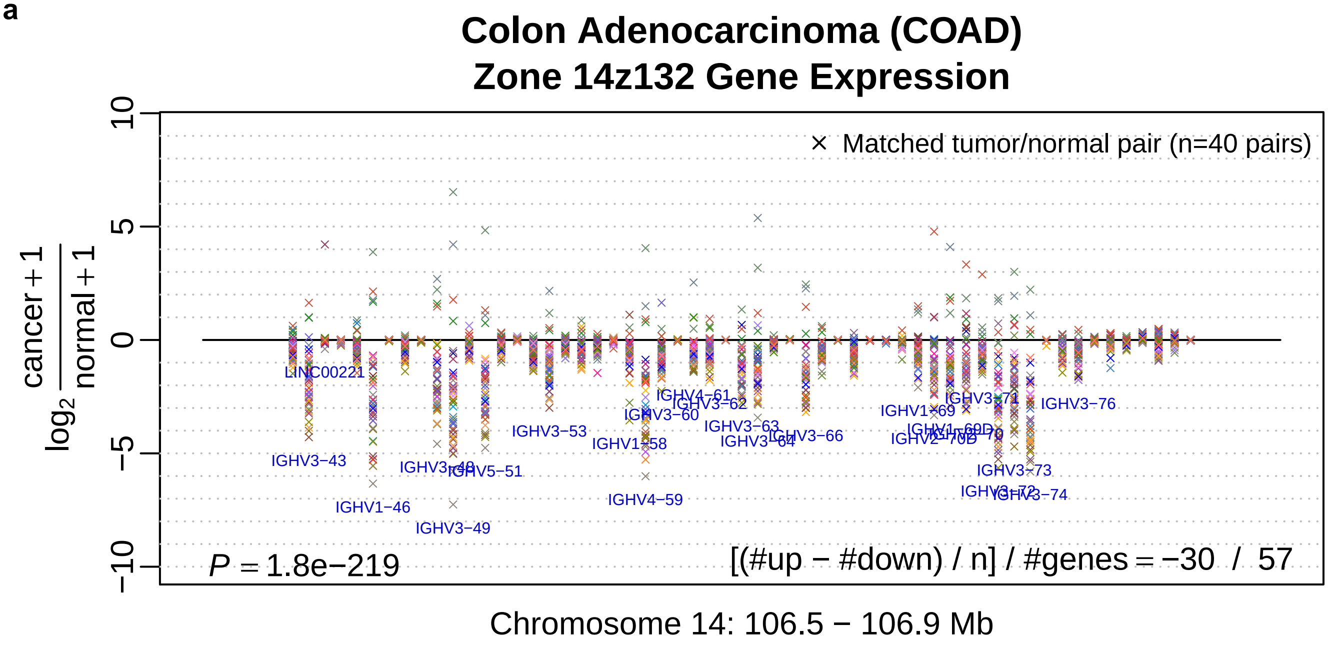

b

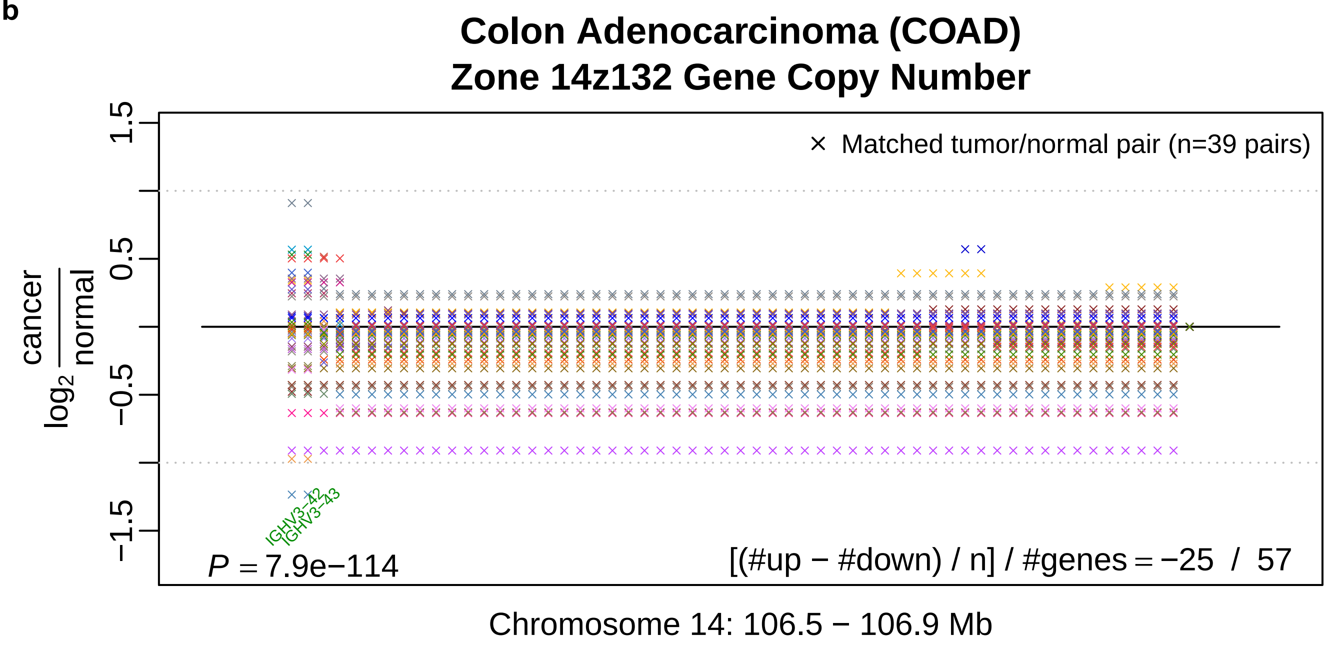

Supplementary Figure S3.4: The most statistically significant polarized regulation zone in COAD. **a**, The gene expression log ratio of cancer to normal for each gene within the zone in each patient. **b**, The somatic copy number log ratio of cancer to normal for each gene within the zone in each patient. See the full legend on page 3.

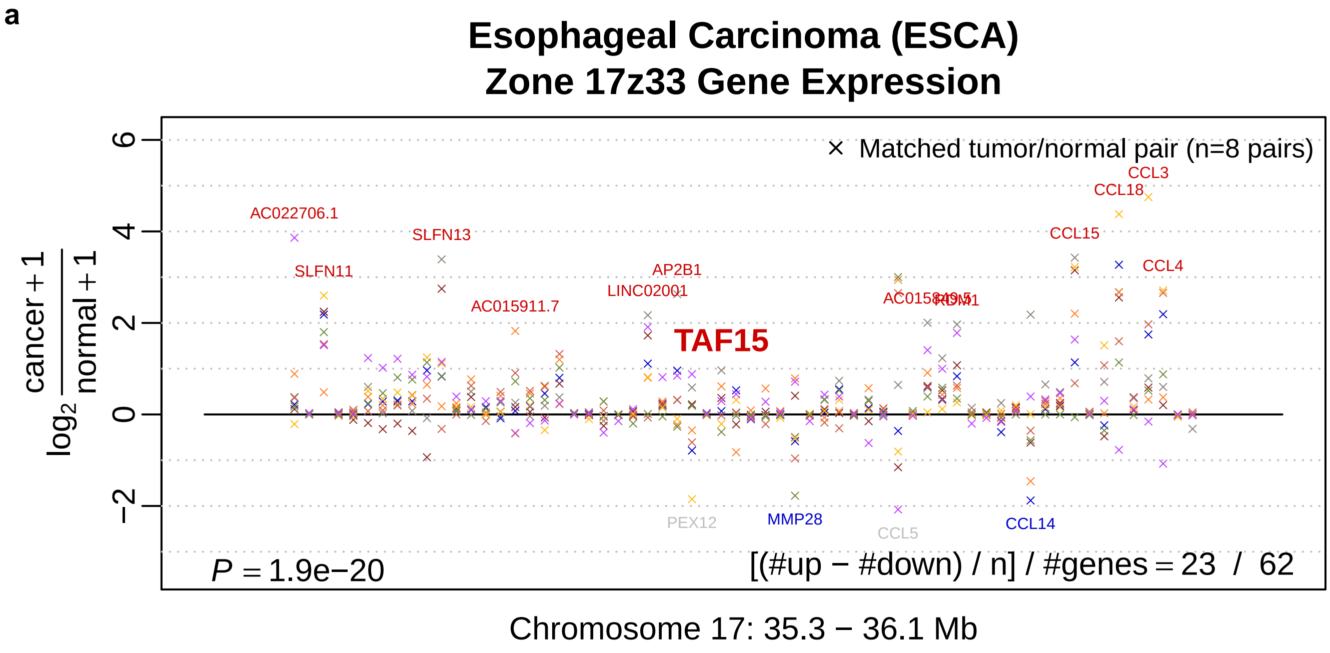

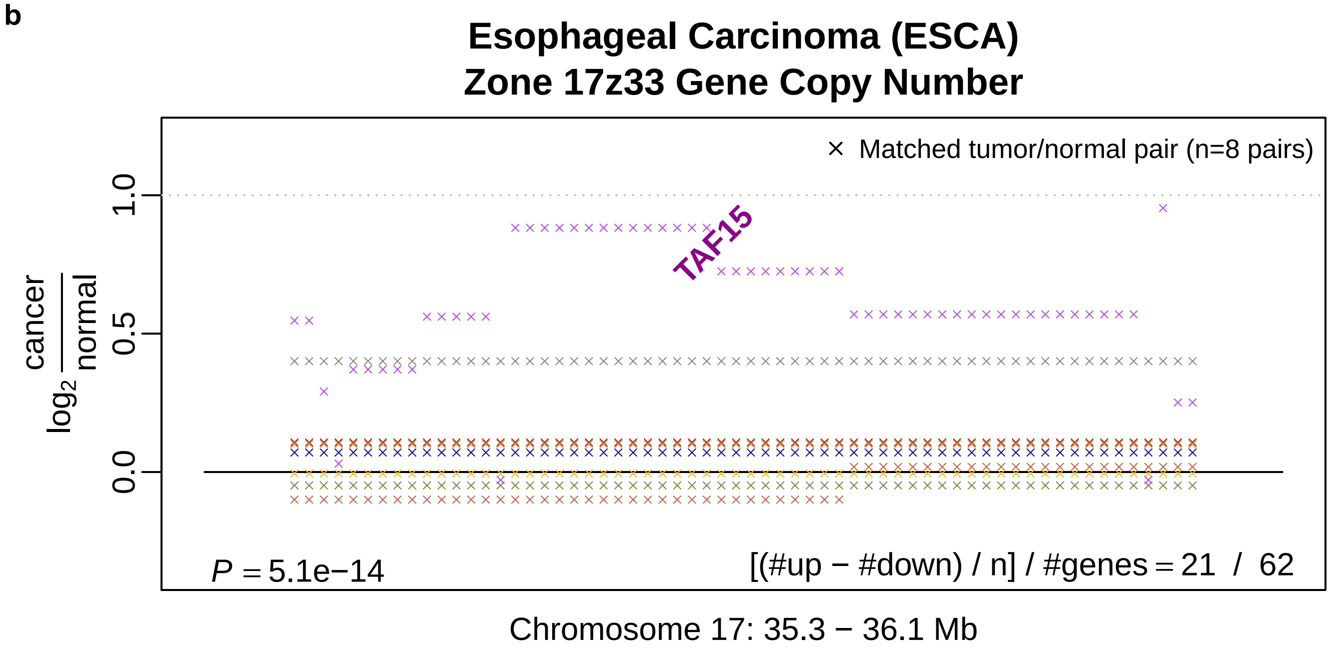

Supplementary Figure S3.5: The most statistically significant polarized regulation zone in ESCA. **a**, The gene expression log ratio of cancer to normal for each gene within the zone in each patient. **b**, The somatic copy number log ratio of cancer to normal for each gene within the zone in each patient. See the full legend on page 3.

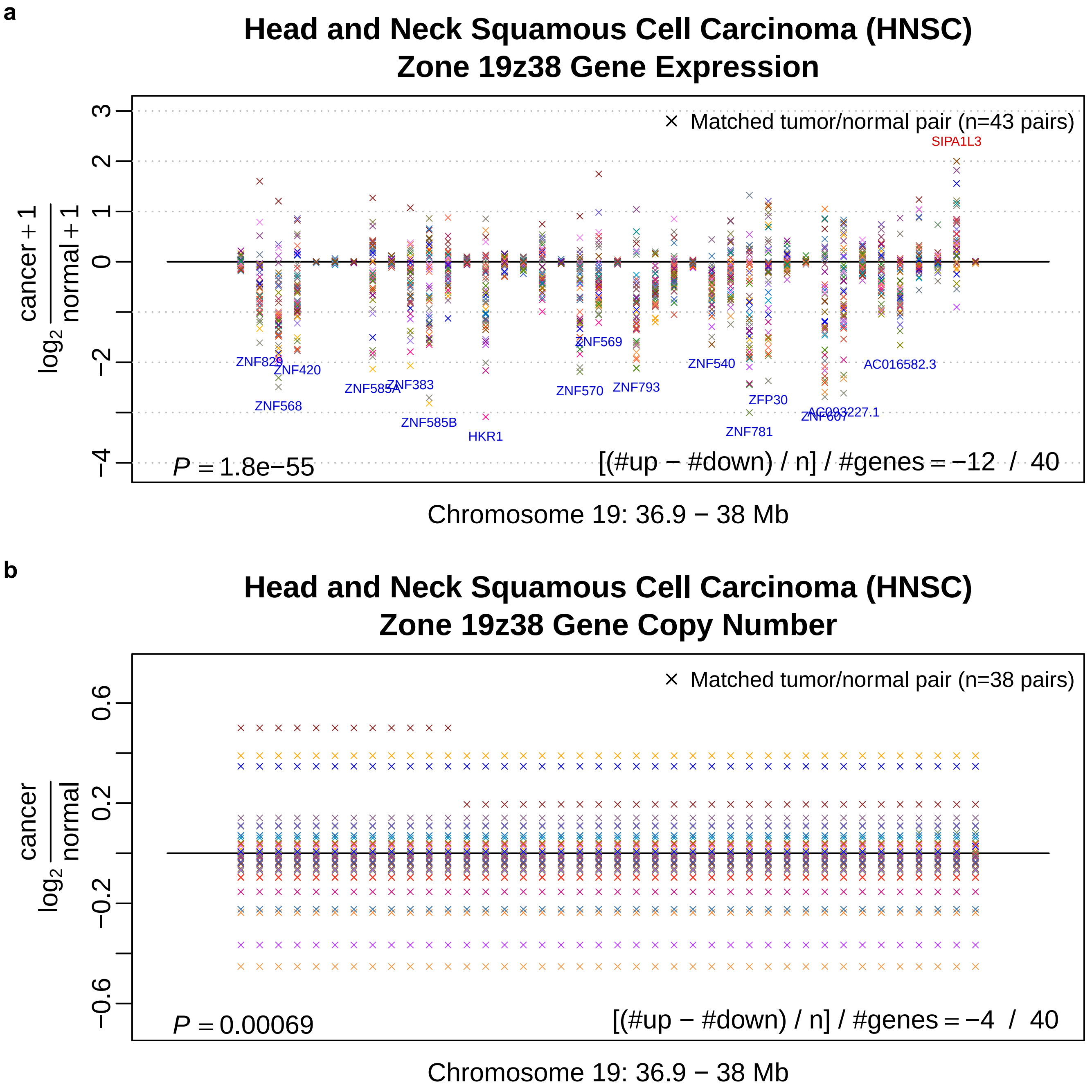

Supplementary Figure S3.6: The most statistically significant polarized regulation zone in HNSC. **a**, The gene expression log ratio of cancer to normal for each gene within the zone in each patient. **b**, The somatic copy number log ratio of cancer to normal for each gene within the zone in each patient. See the full legend on page 3.

a

Kidney Chromophobe (KICH) Zone 19z63 Gene Expression

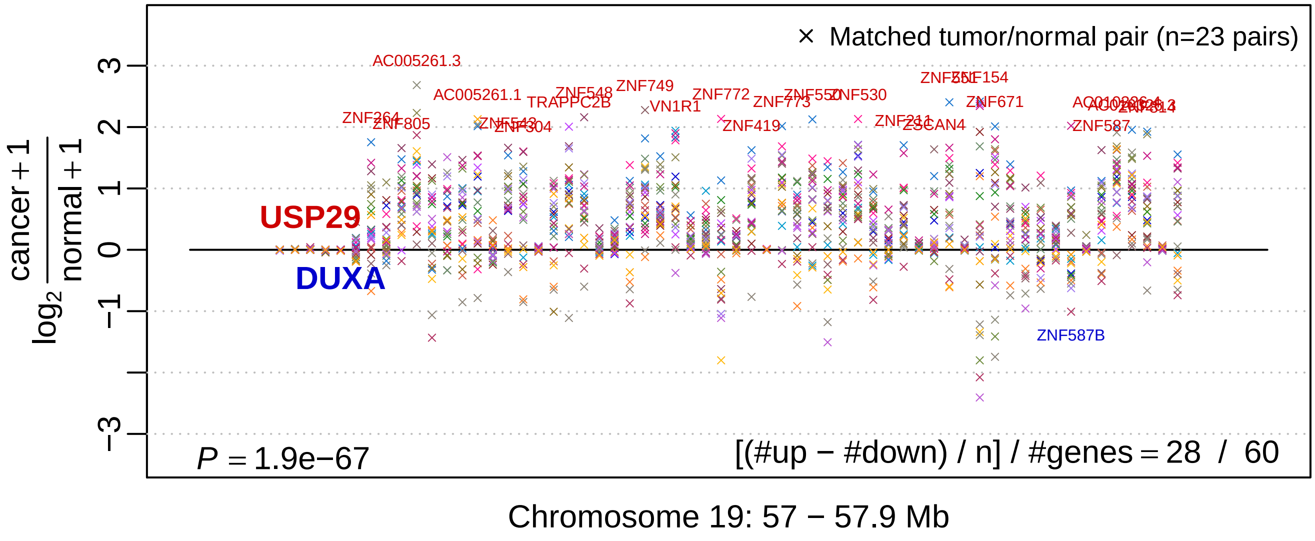

b

Kidney Chromophobe (KICH) Zone 19z63 Gene Copy Number

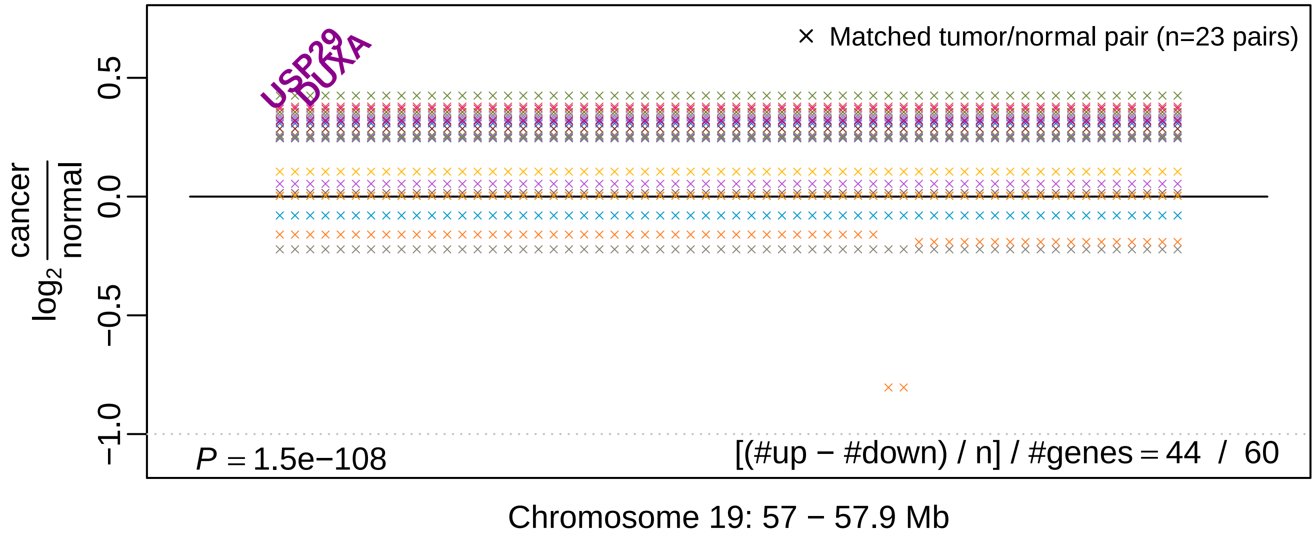

Supplementary Figure S3.7: The most statistically significant polarized regulation zone in KICH. a, The gene expression log ratio of cancer to normal for each gene within the zone in each patient. **b**, The somatic copy number log ratio of cancer to normal for each gene within the zone in each patient. See the full legend on page 3.

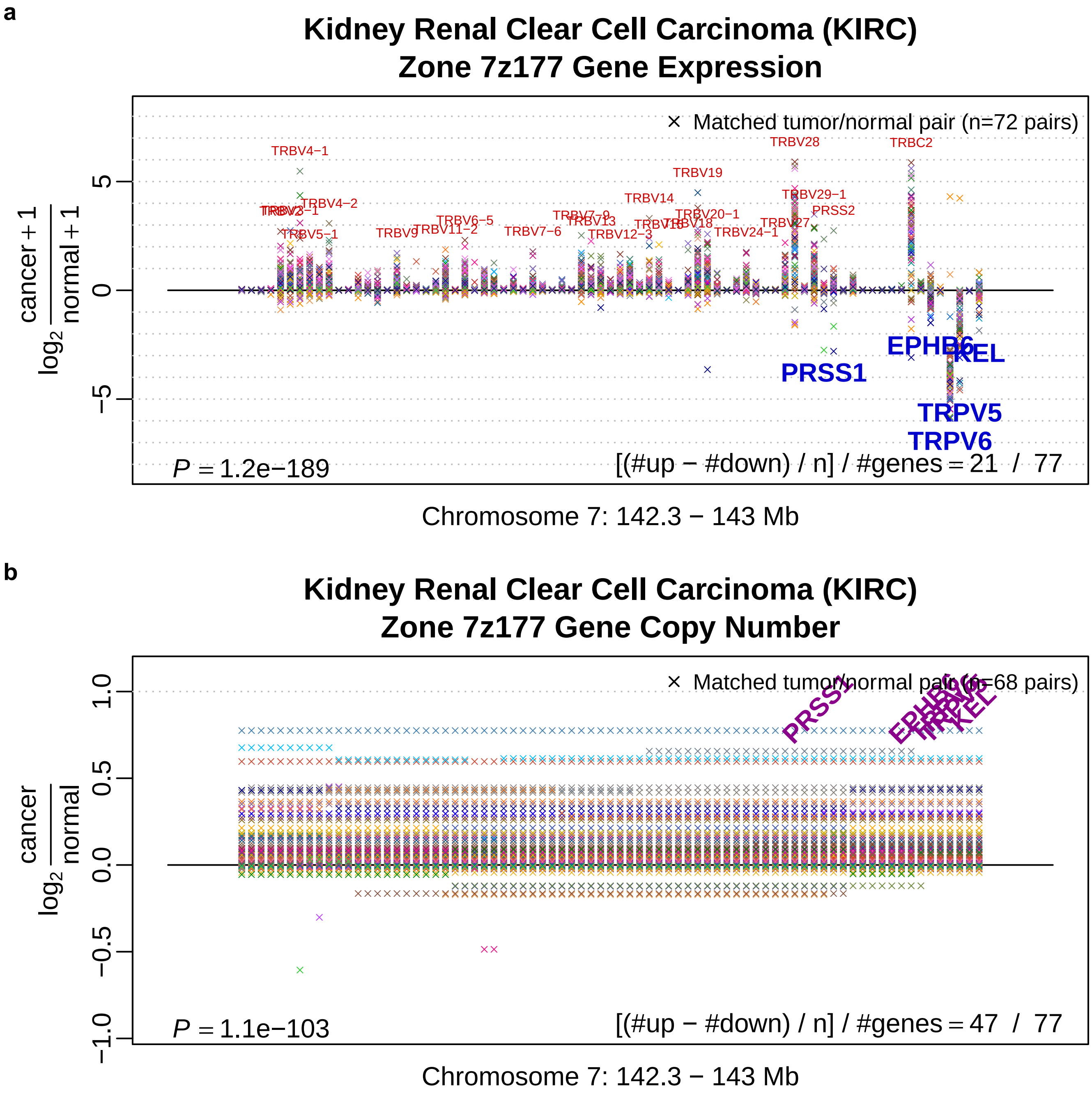

Supplementary Figure S3.8: The most statistically significant polarized regulation zone in KIRC. a, The gene expression log ratio of cancer to normal for each gene within the zone in each patient. **b**, The somatic copy number log ratio of cancer to normal for each gene within the zone in each patient. See the full legend on page 3.

a

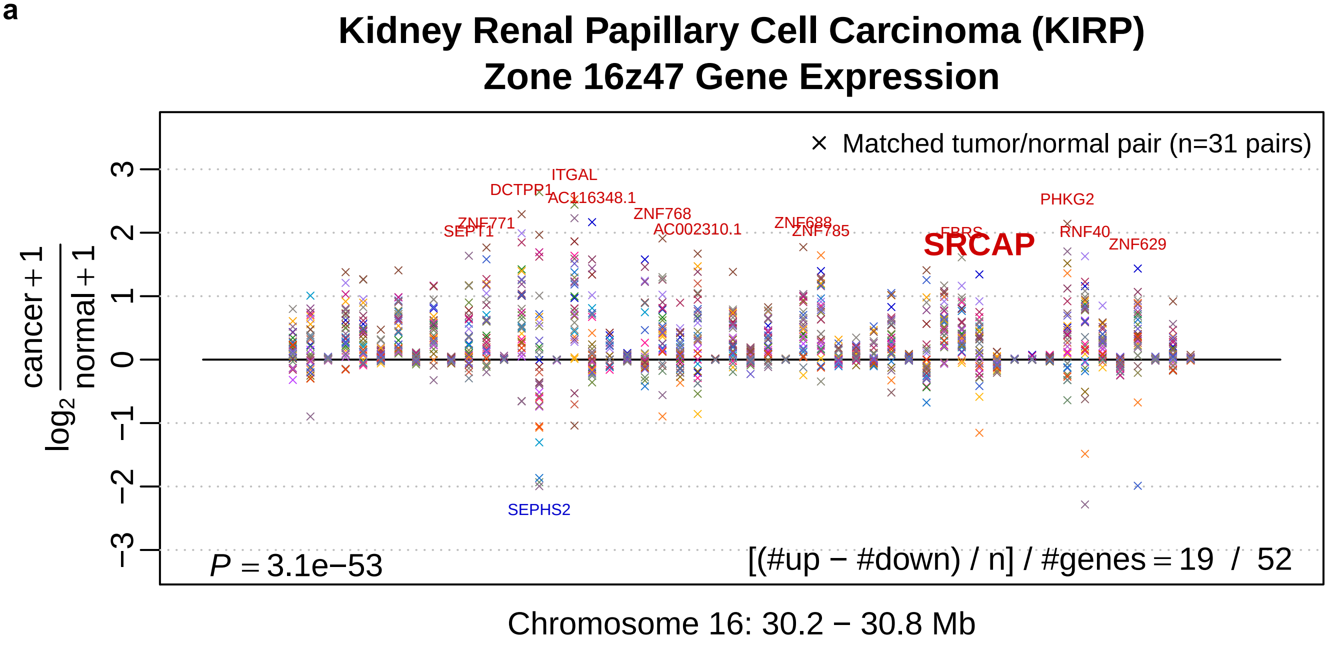

b

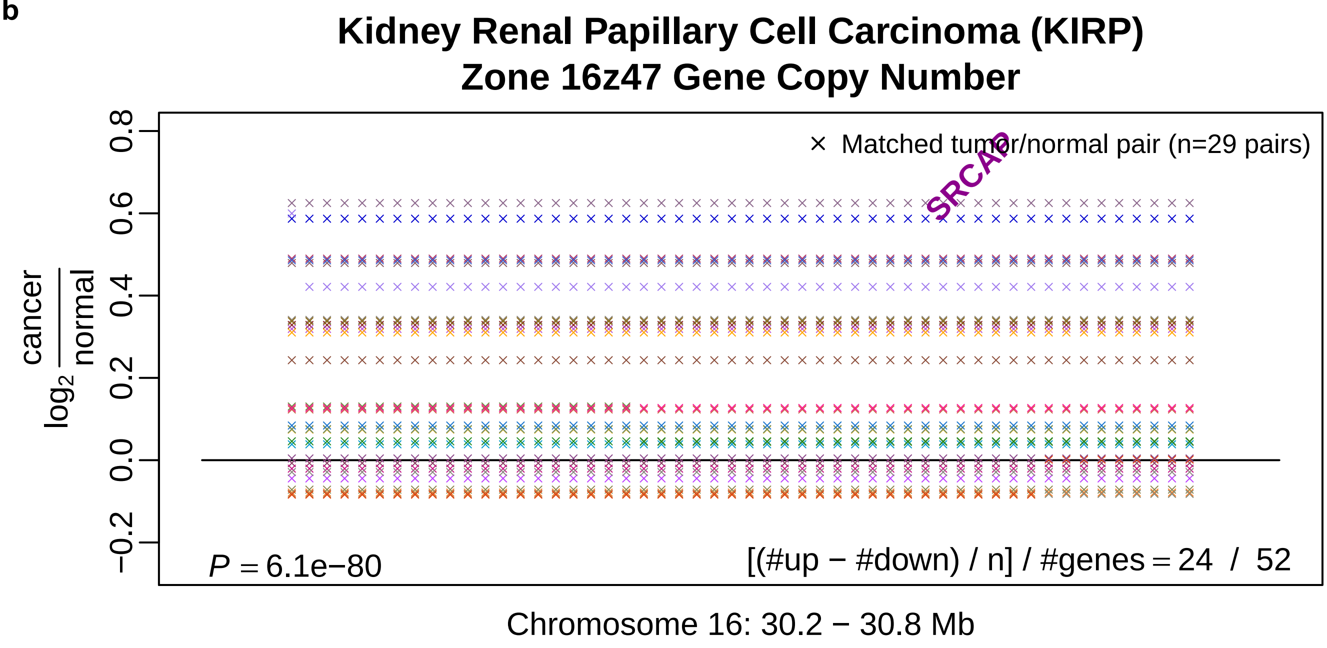

Supplementary Figure S3.9: The most statistically significant polarized regulation zone in KIRP. a, The gene expression log ratio of cancer to normal for each gene within the zone in each patient. **b**, The somatic copy number log ratio of cancer to normal for each gene within the zone in each patient. See the full legend on page 3.

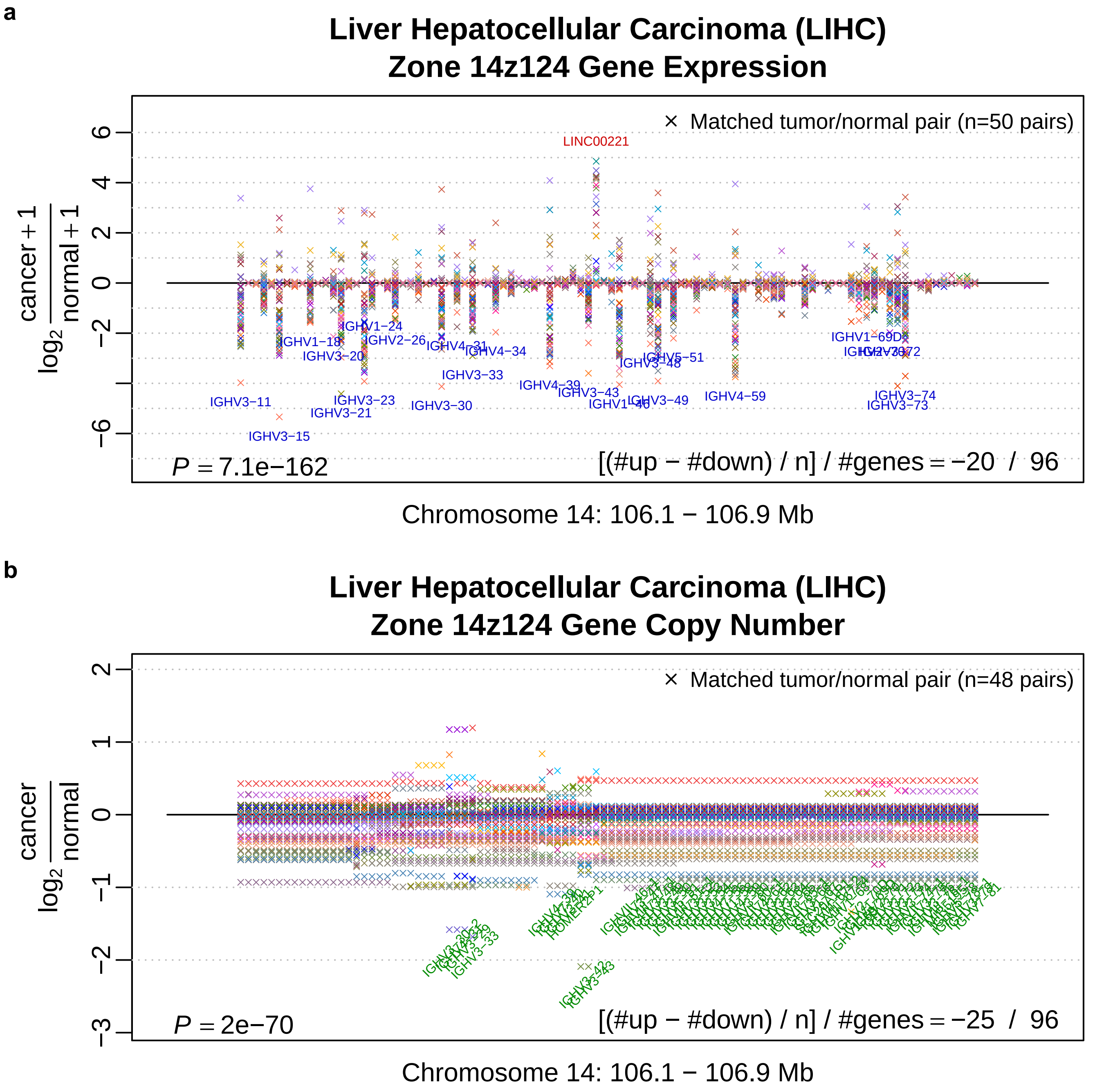

Supplementary Figure S3.10: The most statistically significant polarized regulation zone in LIHC. **a**, The gene expression log ratio of cancer to normal for each gene within the zone in each patient. **b**, The somatic copy number log ratio of cancer to normal for each gene within the zone in each patient. See the full legend on page 3.

a

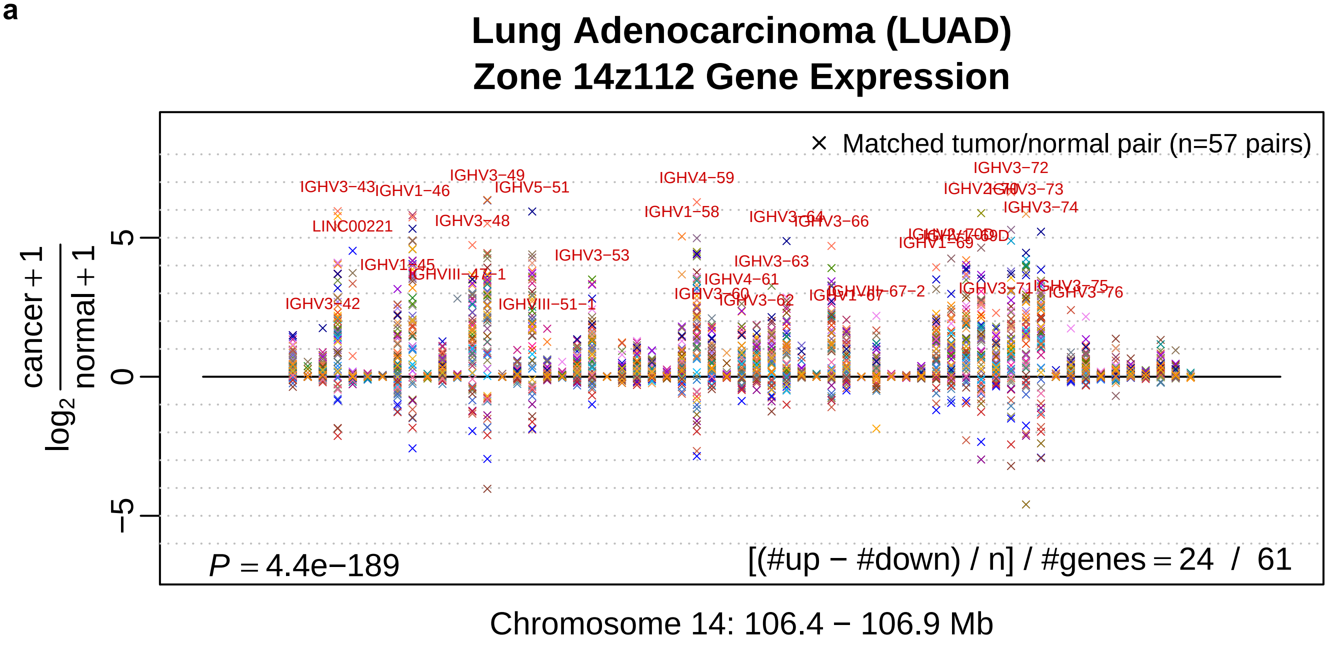

b

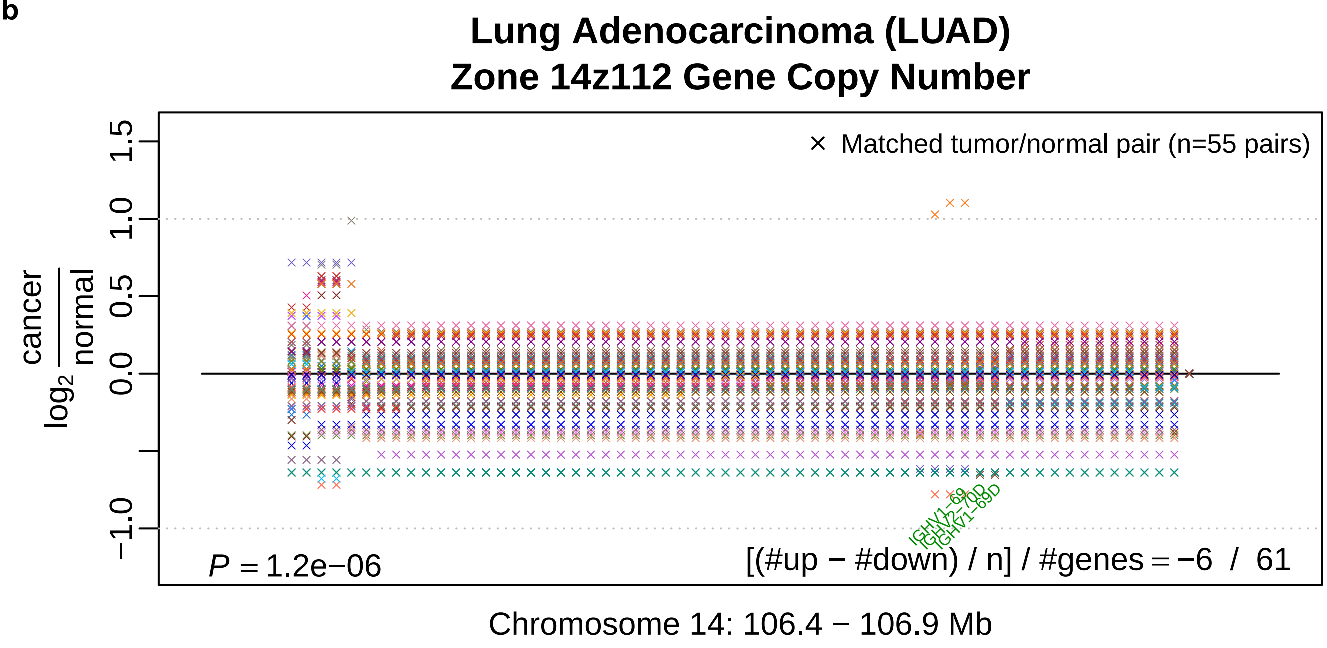

Supplementary Figure S3.11: The most statistically significant polarized regulation zone in LUAD. **a**, The gene expression log ratio of cancer to normal for each gene within the zone in each patient. **b**, The somatic copy number log ratio of cancer to normal for each gene within the zone in each patient. See the full legend on page 3.

a

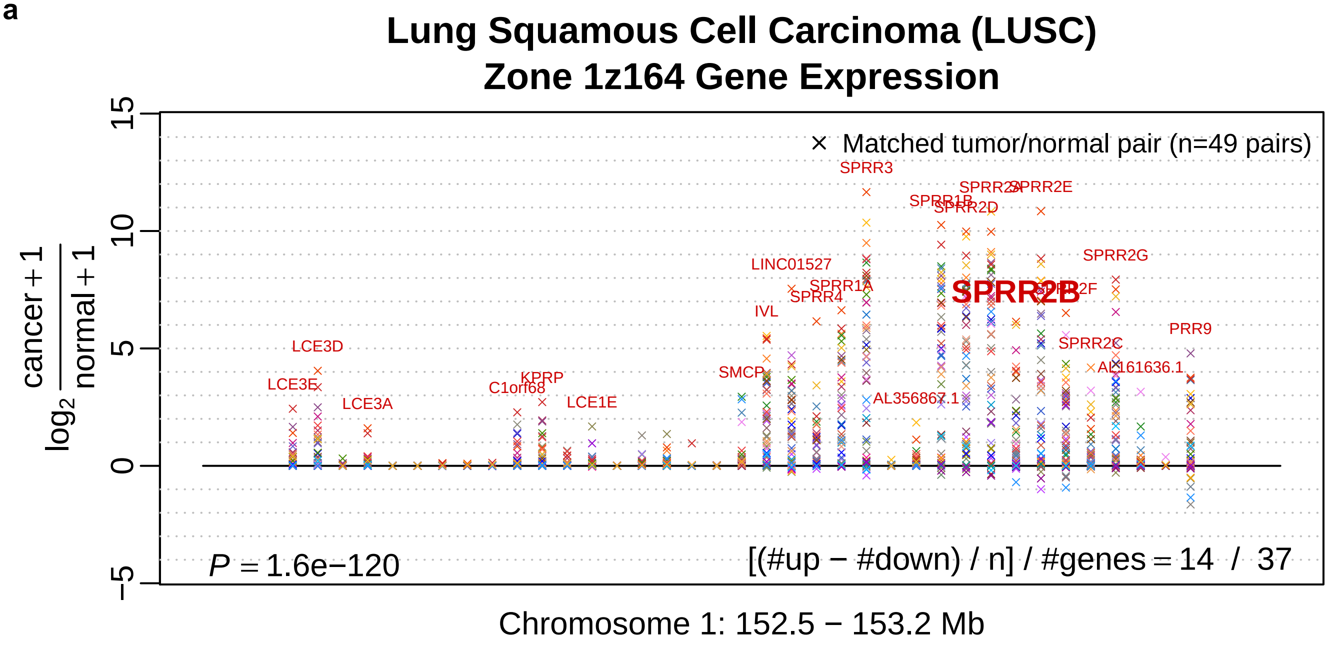

b

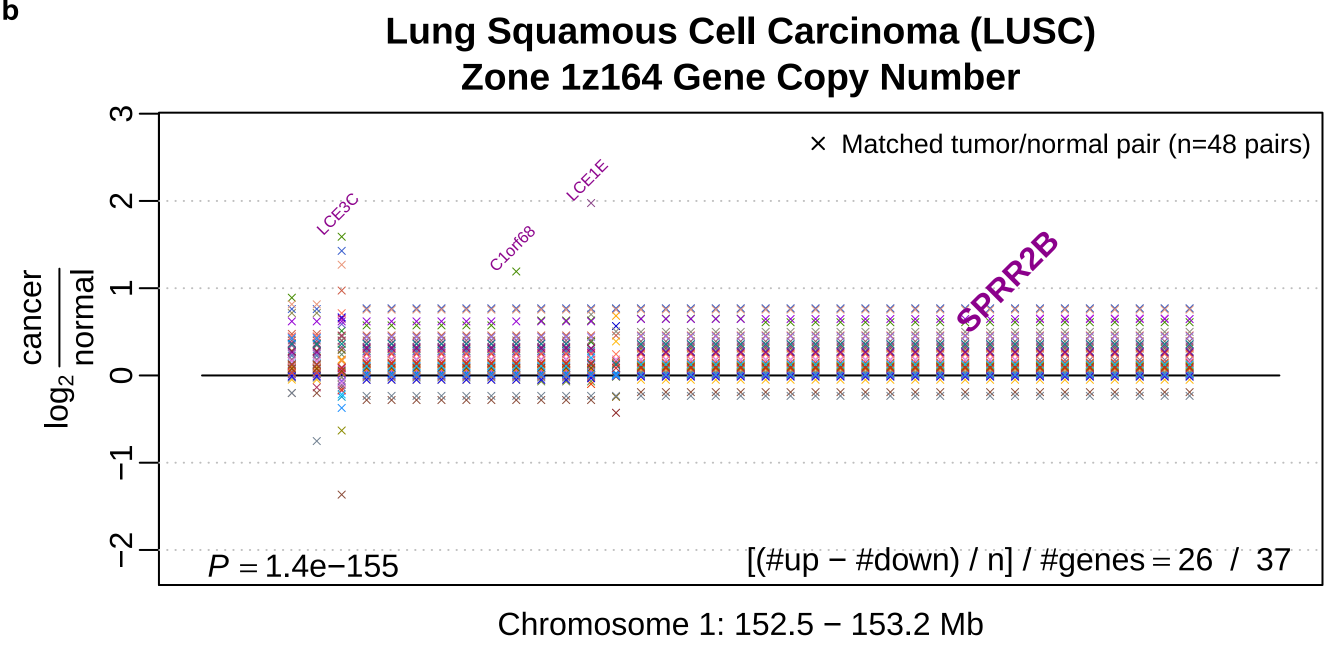

Supplementary Figure S3.12: The most statistically significant polarized regulation zone in LUSC. **a**, The gene expression log ratio of cancer to normal for each gene within the zone in each patient. **b**, The somatic copy number log ratio of cancer to normal for each gene within the zone in each patient. See the full legend on page 3.

a

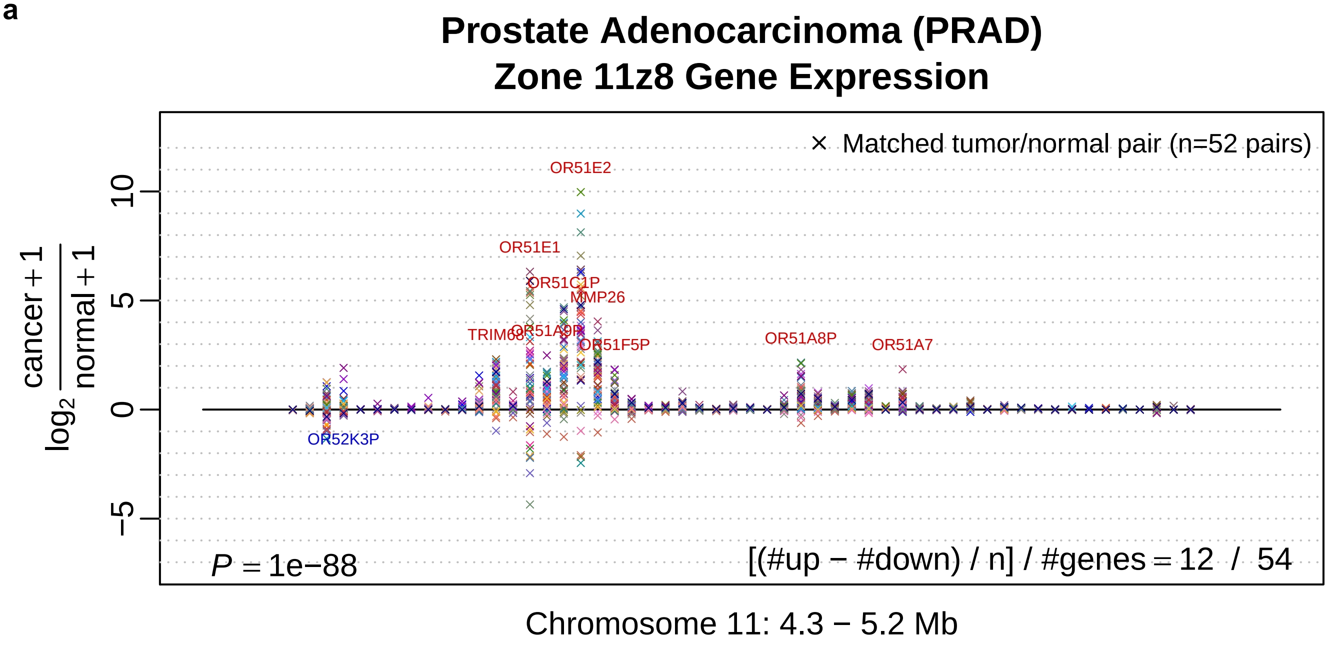

b

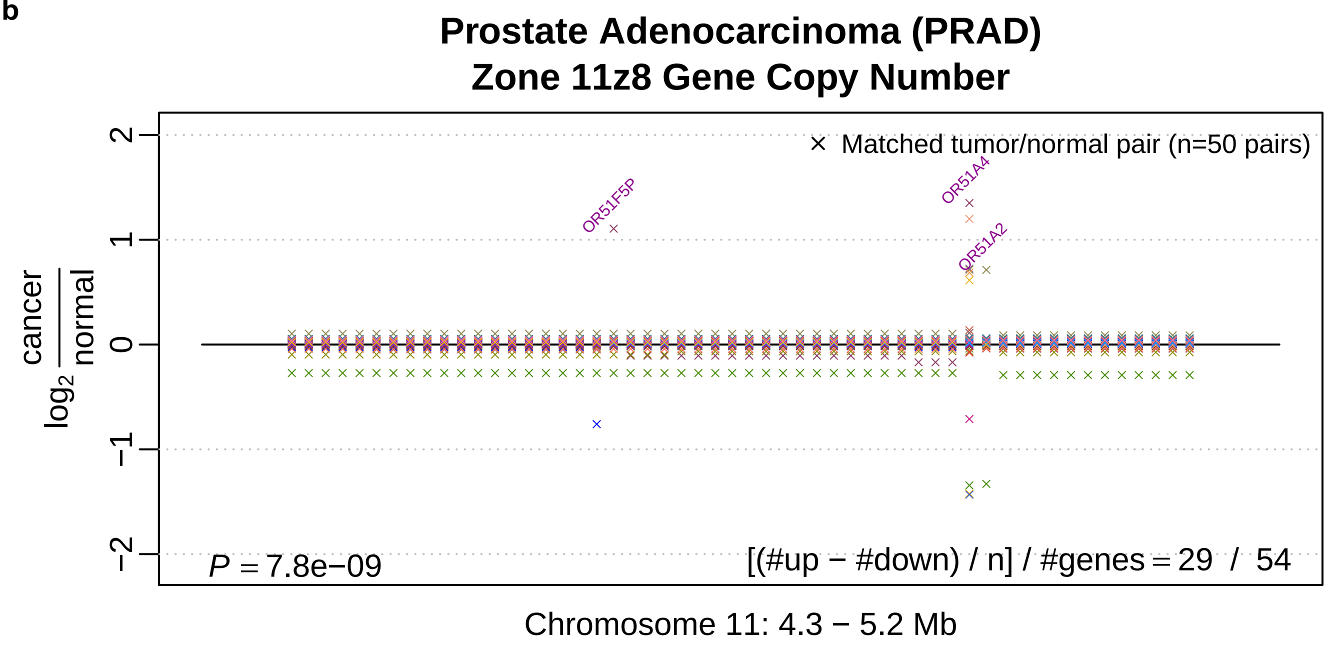

Supplementary Figure S3.13: The most statistically significant polarized regulation zone in PRAD. **a**, The gene expression log ratio of cancer to normal for each gene within the zone in each patient. **b**, The somatic copy number log ratio of cancer to normal for each gene within the zone in each patient. See the full legend on page 3.

a

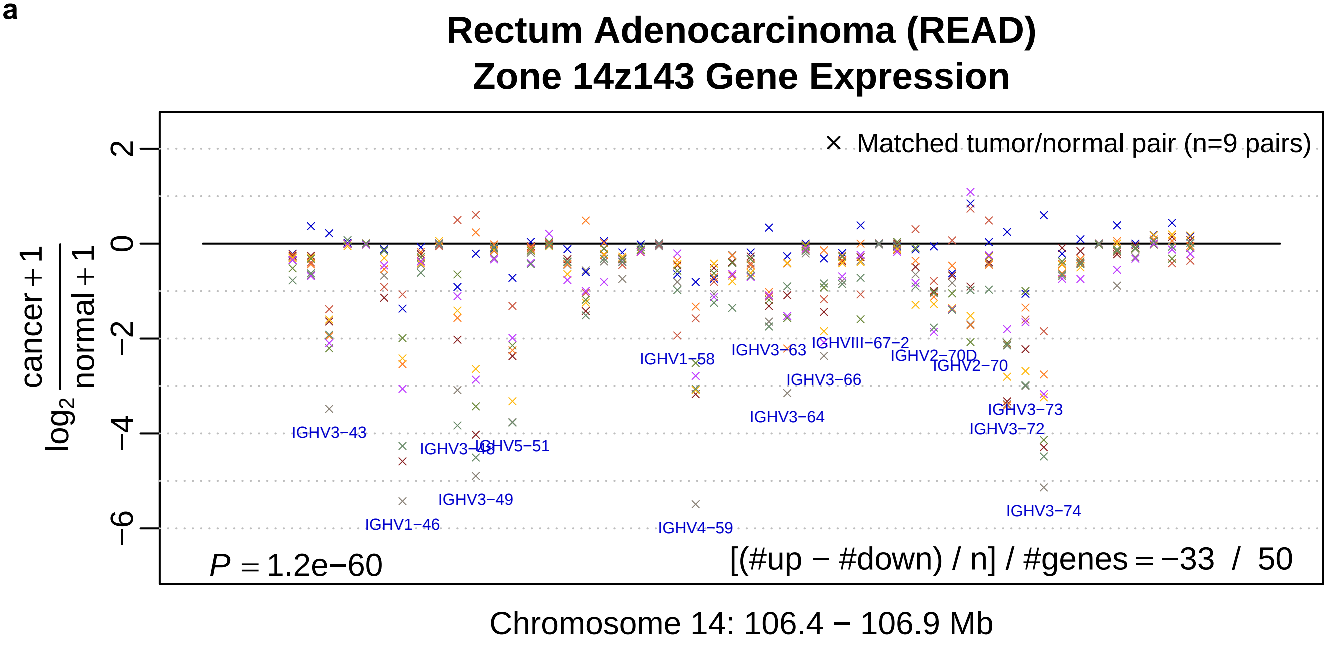

b

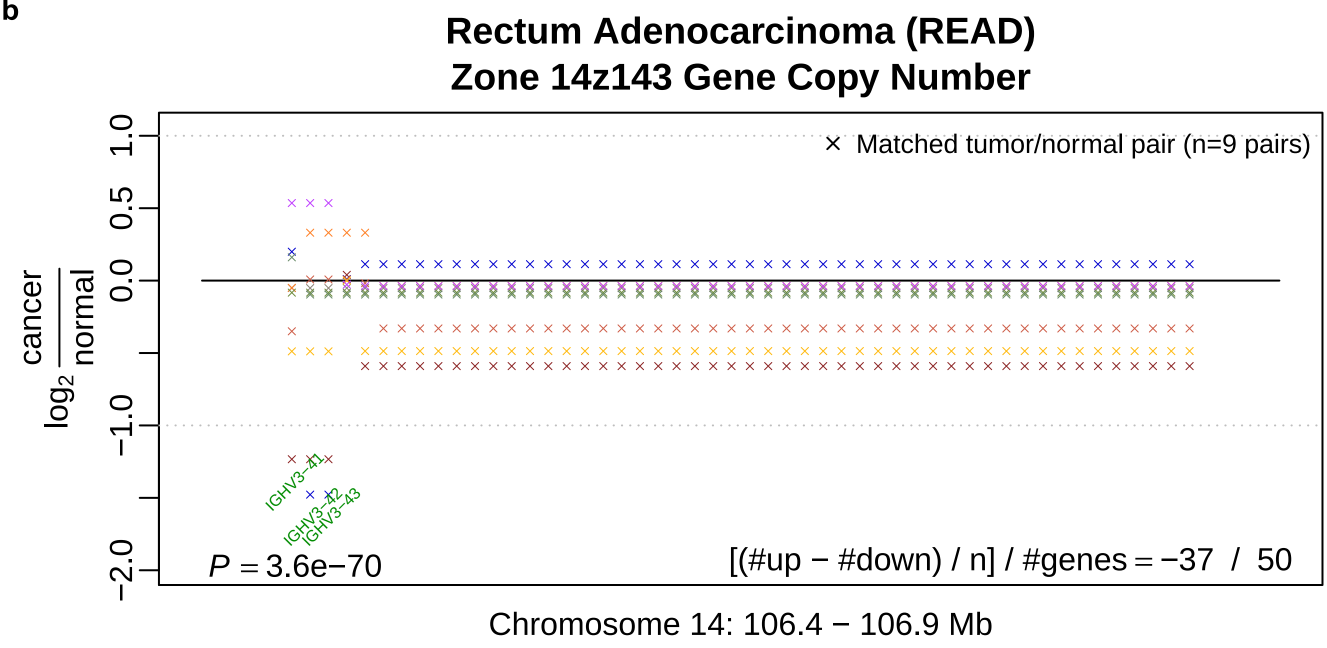

Supplementary Figure S3.14: The most statistically significant polarized regulation zone in READ. **a**, The gene expression log ratio of cancer to normal for each gene within the zone in each patient. **b**, The somatic copy number log ratio of cancer to normal for each gene within the zone in each patient. See the full legend on page 3.

a

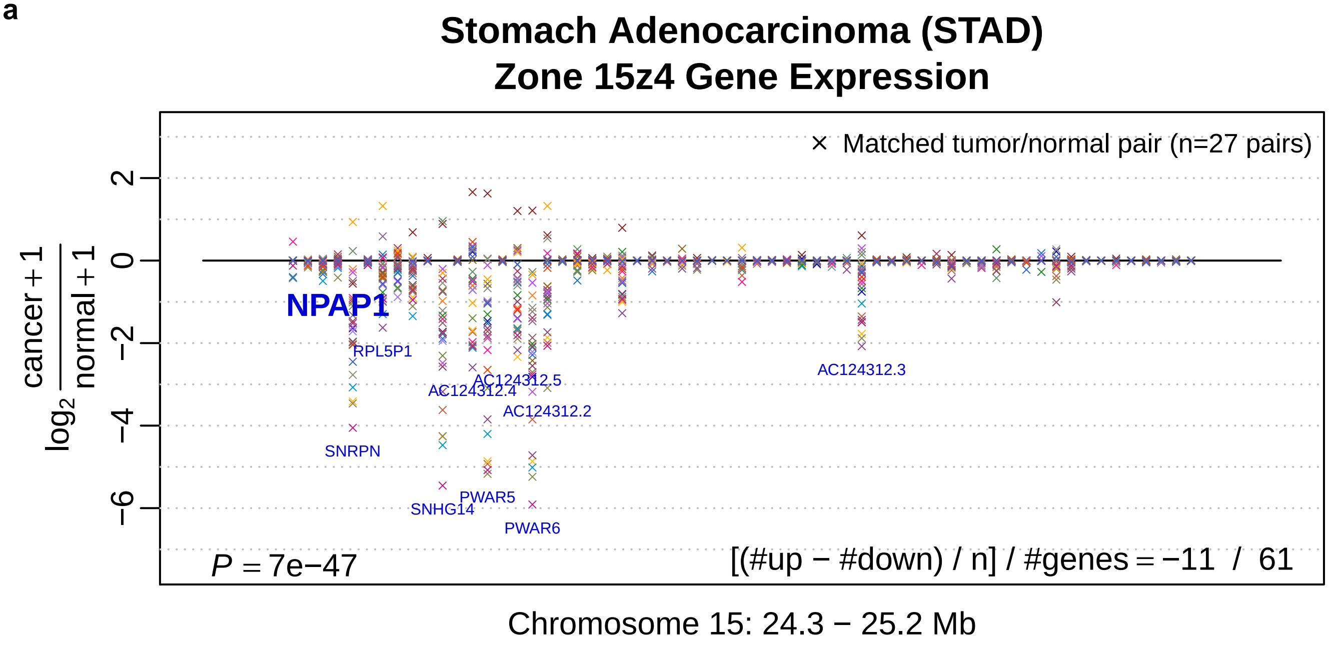

b

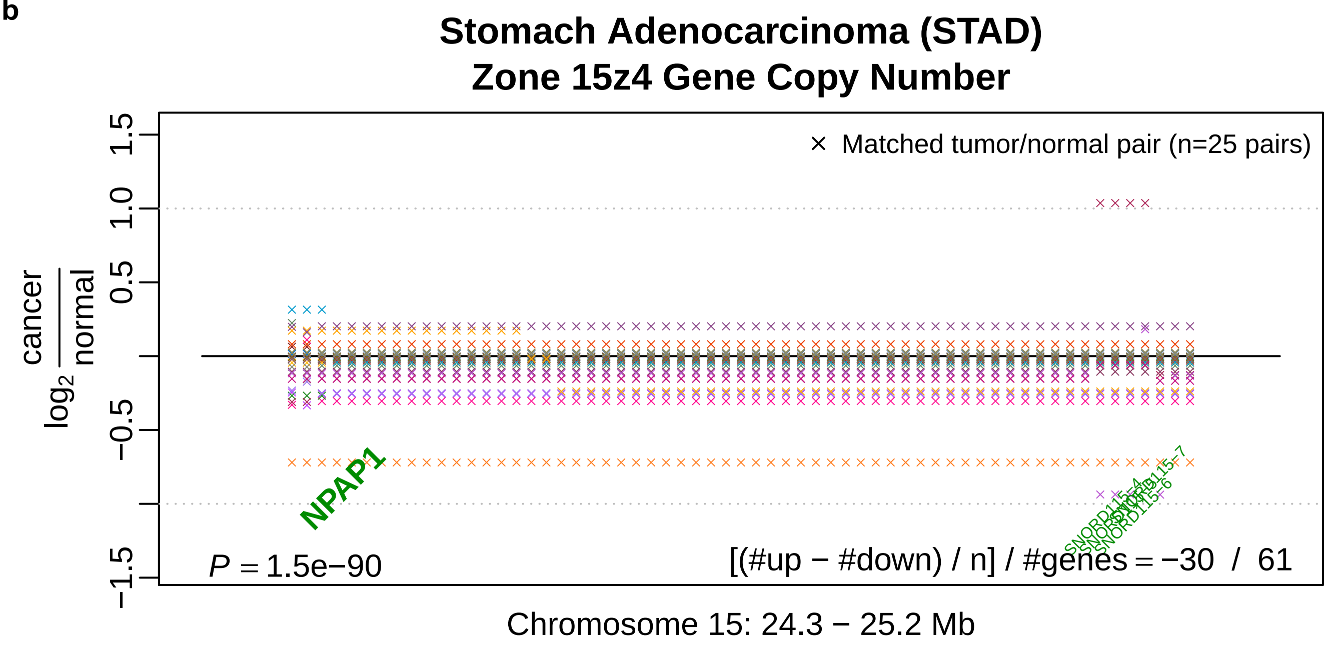

Supplementary Figure S3.15: The most statistically significant polarized regulation zone in STAD. **a**, The gene expression log ratio of cancer to normal for each gene within the zone in each patient. **b**, The somatic copy number log ratio of cancer to normal for each gene within the zone in each patient. See the full legend on page 3.

a

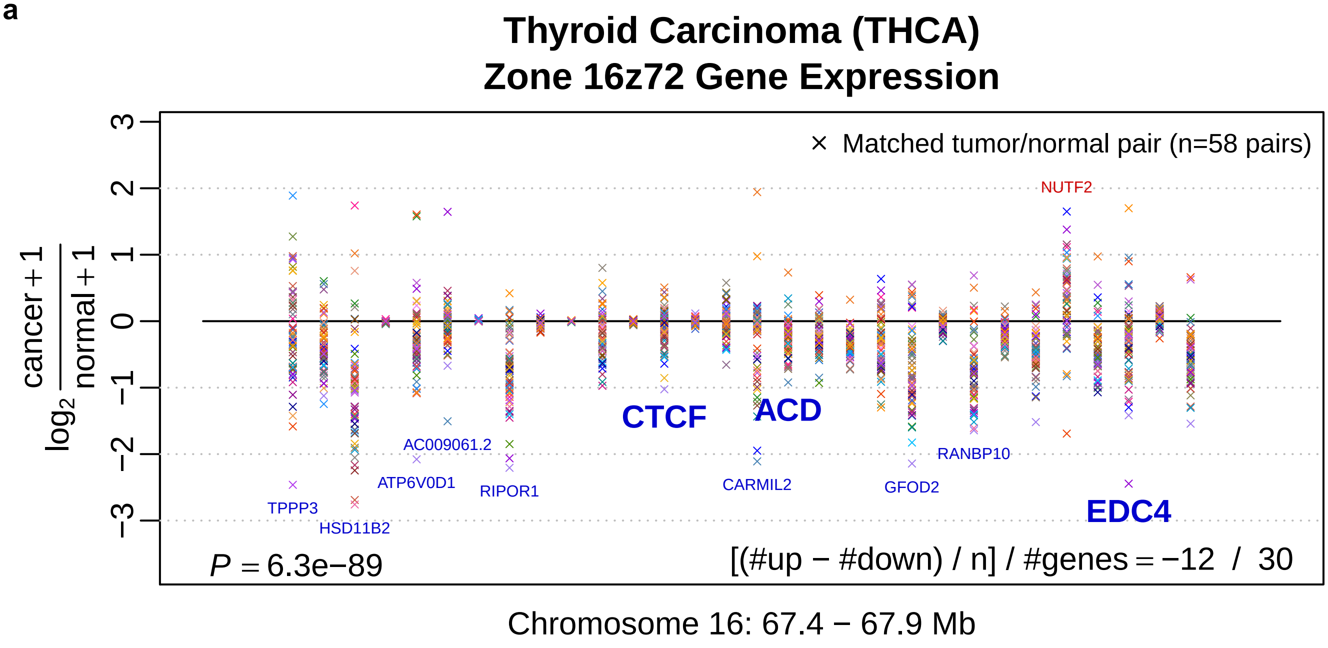

b

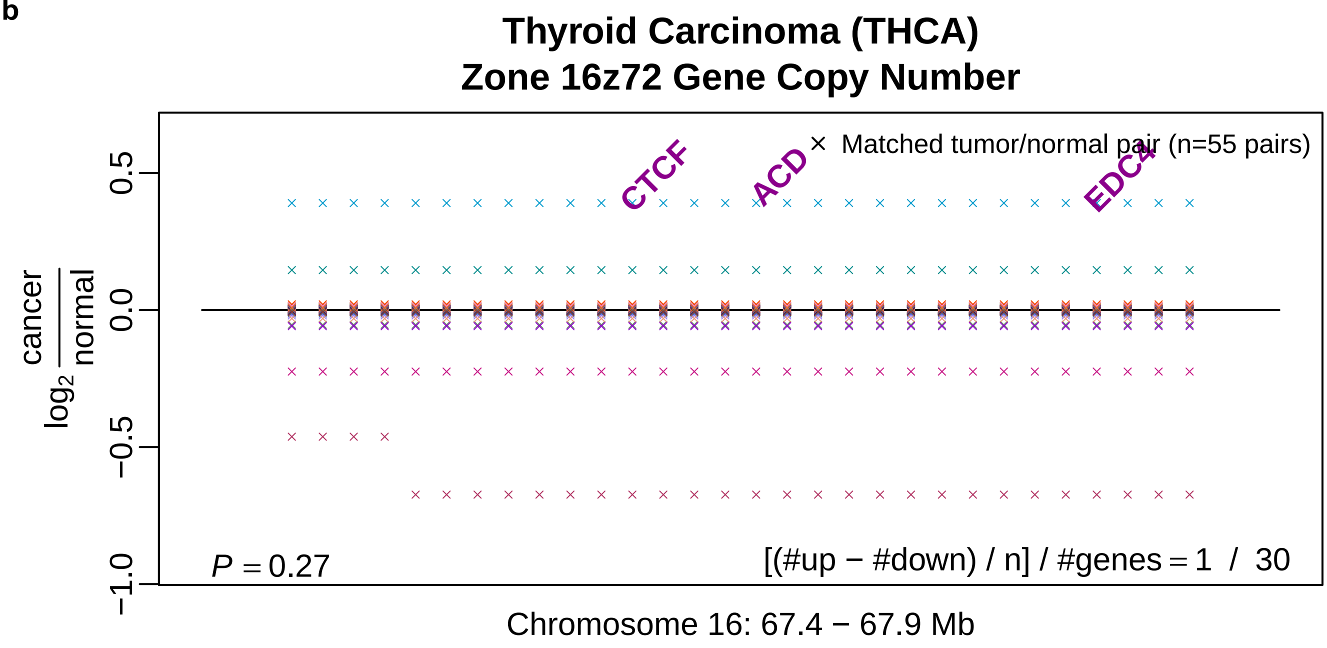

Supplementary Figure S3.16: The most statistically significant polarized regulation zone in THCA. a, The gene expression log ratio of cancer to normal for each gene within the zone in each patient. **b**, The somatic copy number log ratio of cancer to normal for each gene within the zone in each patient. See the full legend on page 3.

a

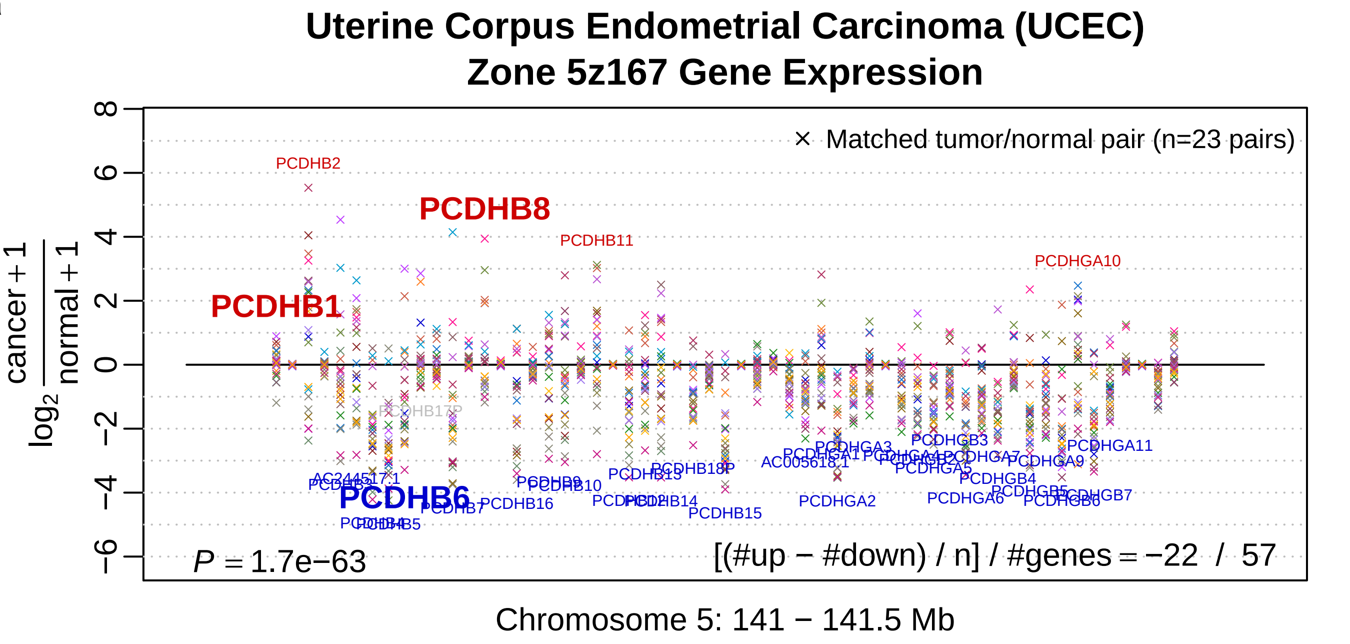

b

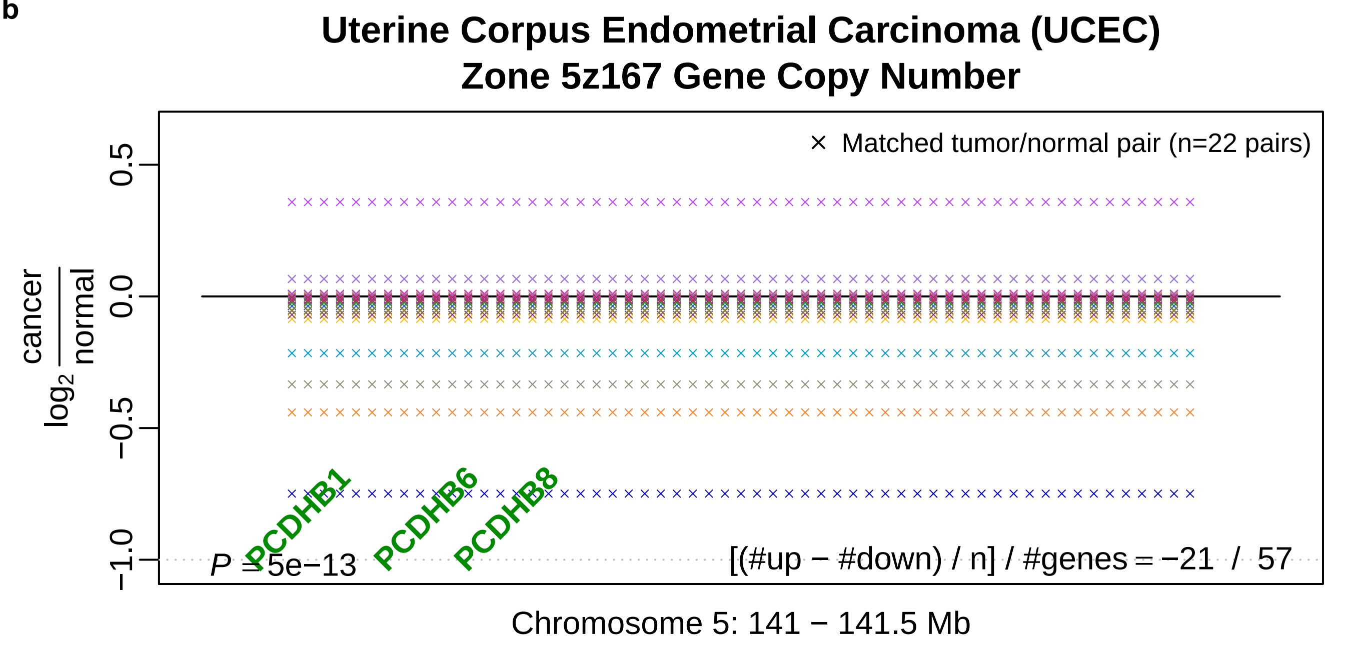

Supplementary Figure S3.17: The most statistically significant polarized regulation zone in UCEC. **a**, The gene expression log ratio of cancer to normal for each gene within the zone in each patient. **b**, The somatic copy number log ratio of cancer to normal for each gene within the zone in each patient. See the full legend on page 3.

1 Second most polarized regulation zones of 17 cancer types

a

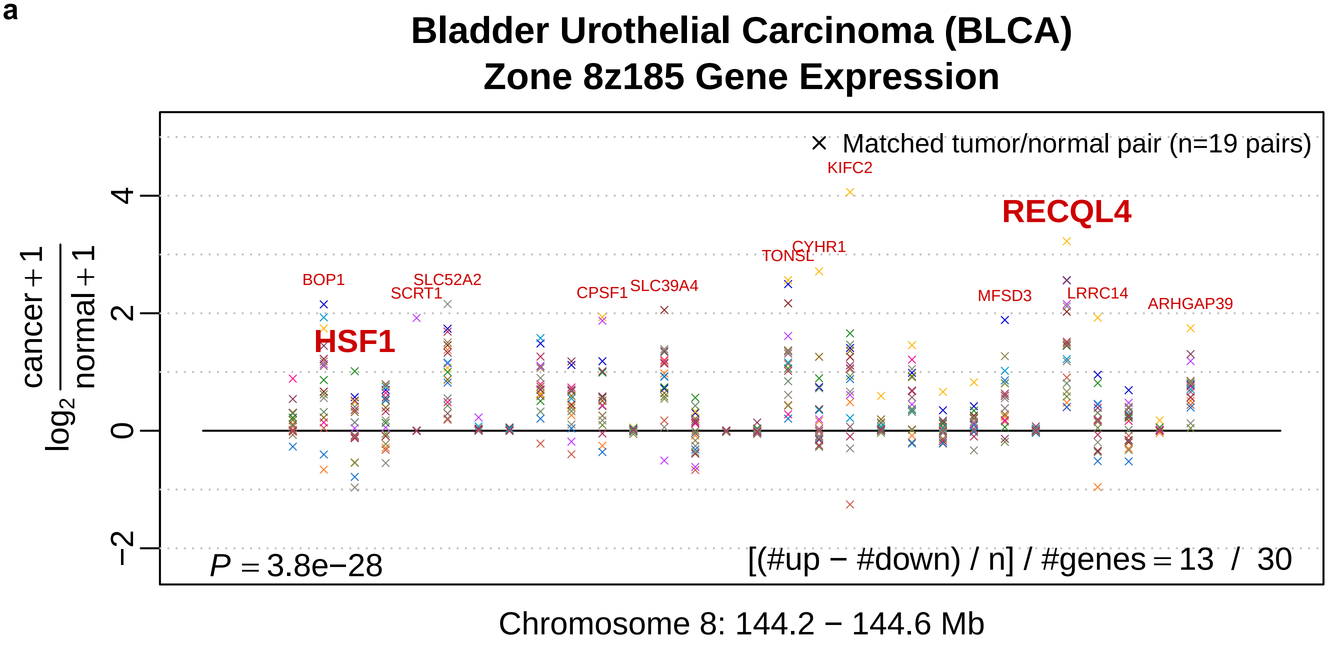

b

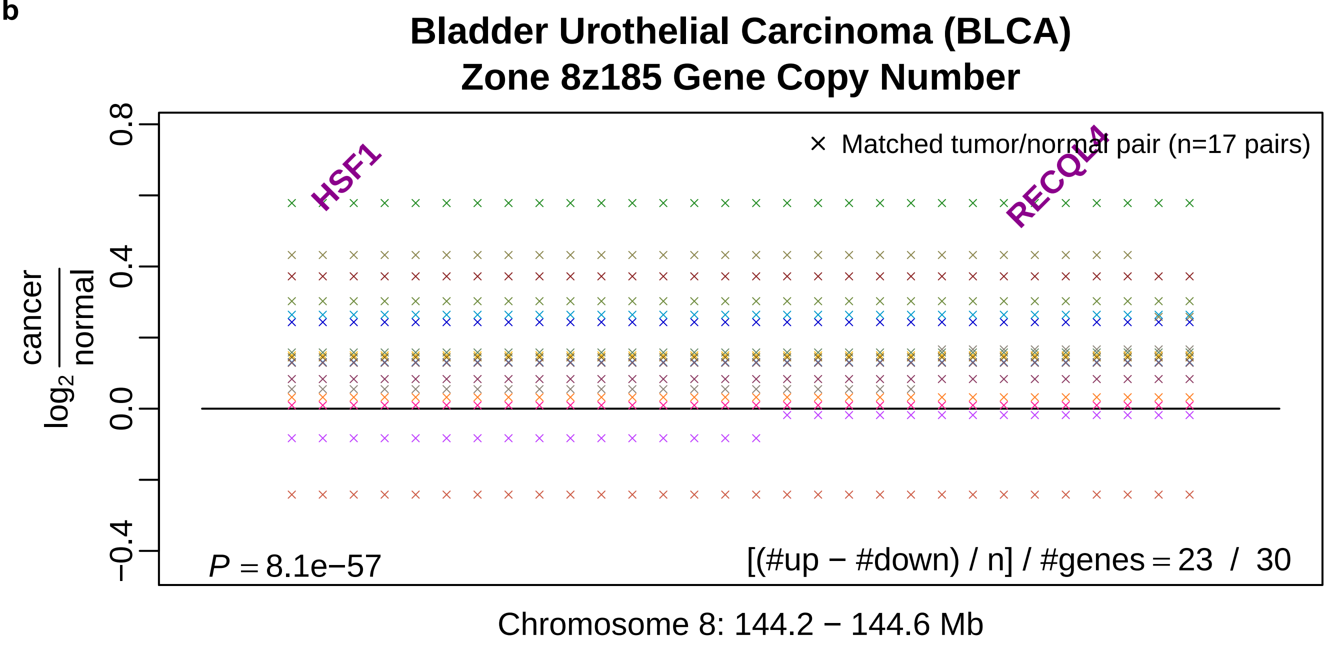

Supplementary Figure S3.18: The second most statistically significant polarized regulation zone in BLCA. a, The gene expression log ratio of cancer to normal for each gene within the zone in each patient. **b**, The somatic copy number log ratio of cancer to normal for each gene within the zone in each patient. See the full legend on page 3.

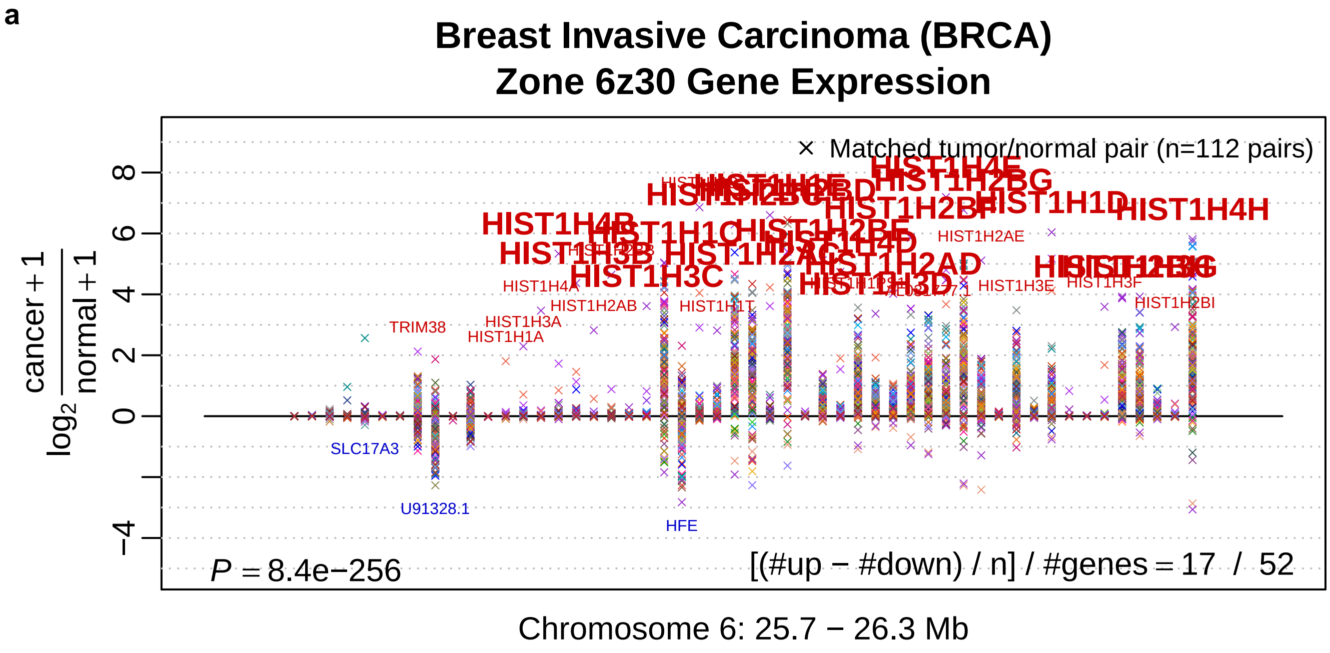

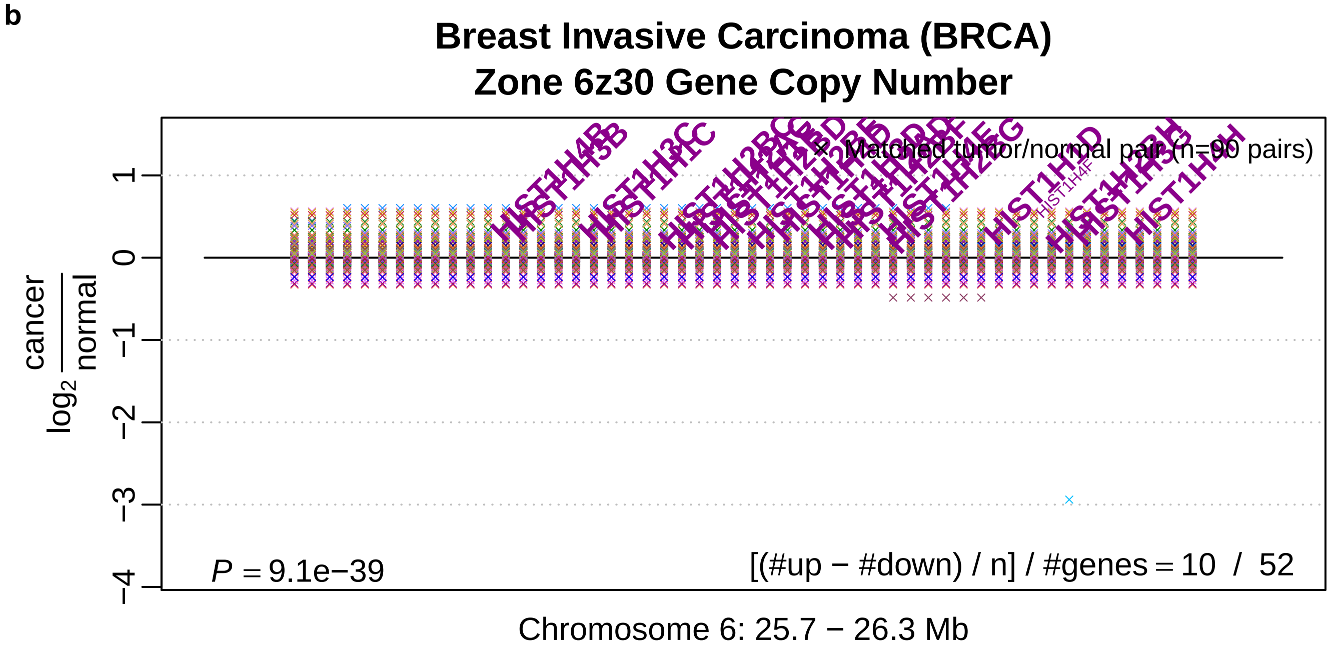

Supplementary Figure S3.19: The second most statistically significant polarized regulation zone in BRCA. a, The gene expression log ratio of cancer to normal for each gene within the zone in each patient. **b**, The somatic copy number log ratio of cancer to normal for each gene within the zone in each patient. See the full legend on page 3.

a

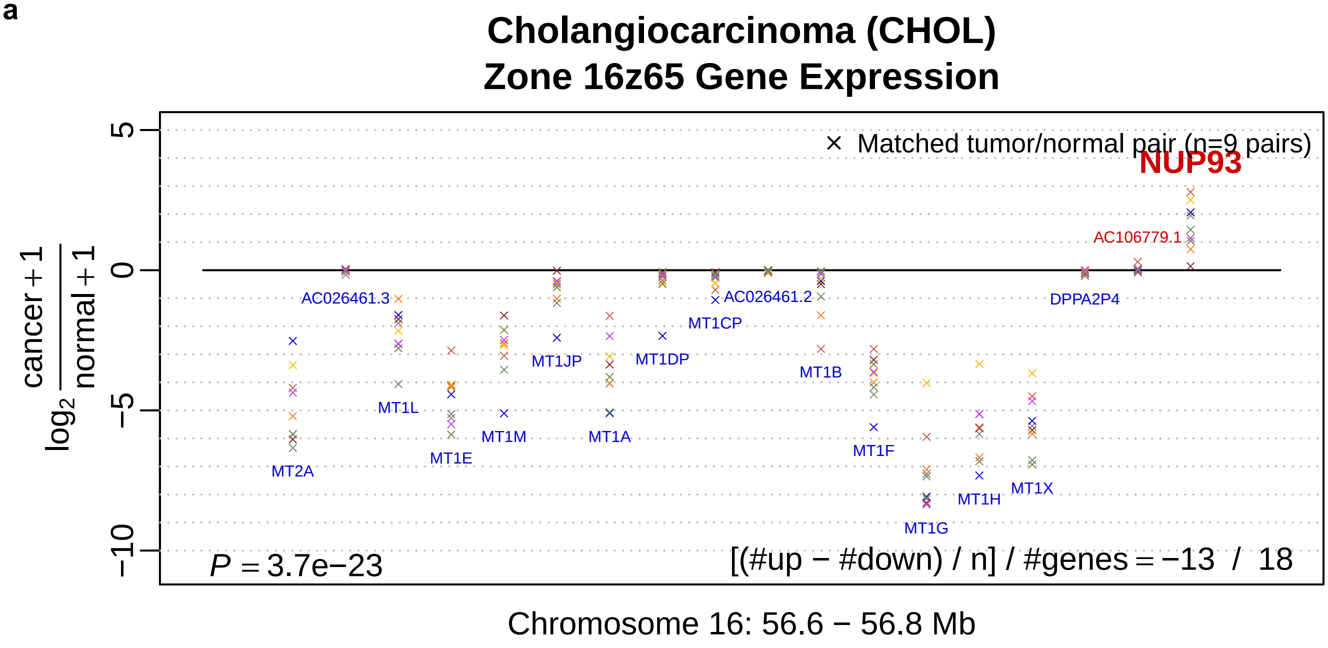

b

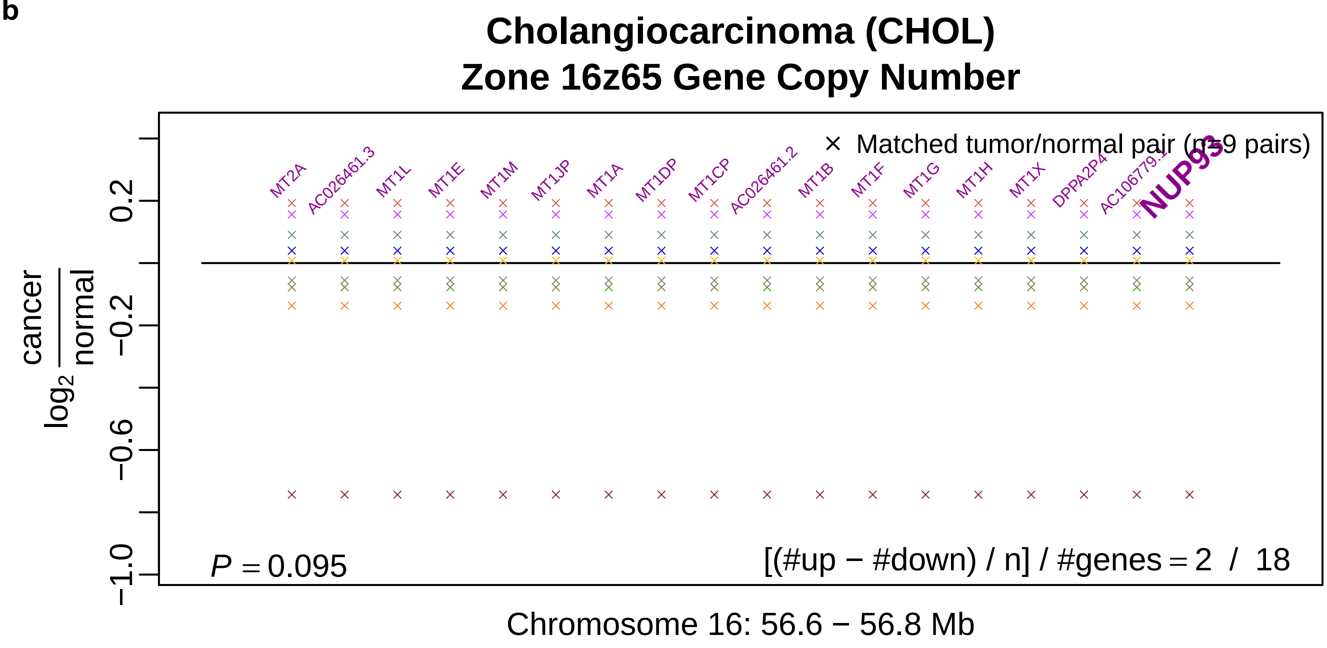

Supplementary Figure S3.20: The second most statistically significant polarized regulation zone in CHOL. a, The gene expression log ratio of cancer to normal for each gene within the zone in each patient. **b**, The somatic copy number log ratio of cancer to normal for each gene within the zone in each patient. See the full legend on page 3.

a

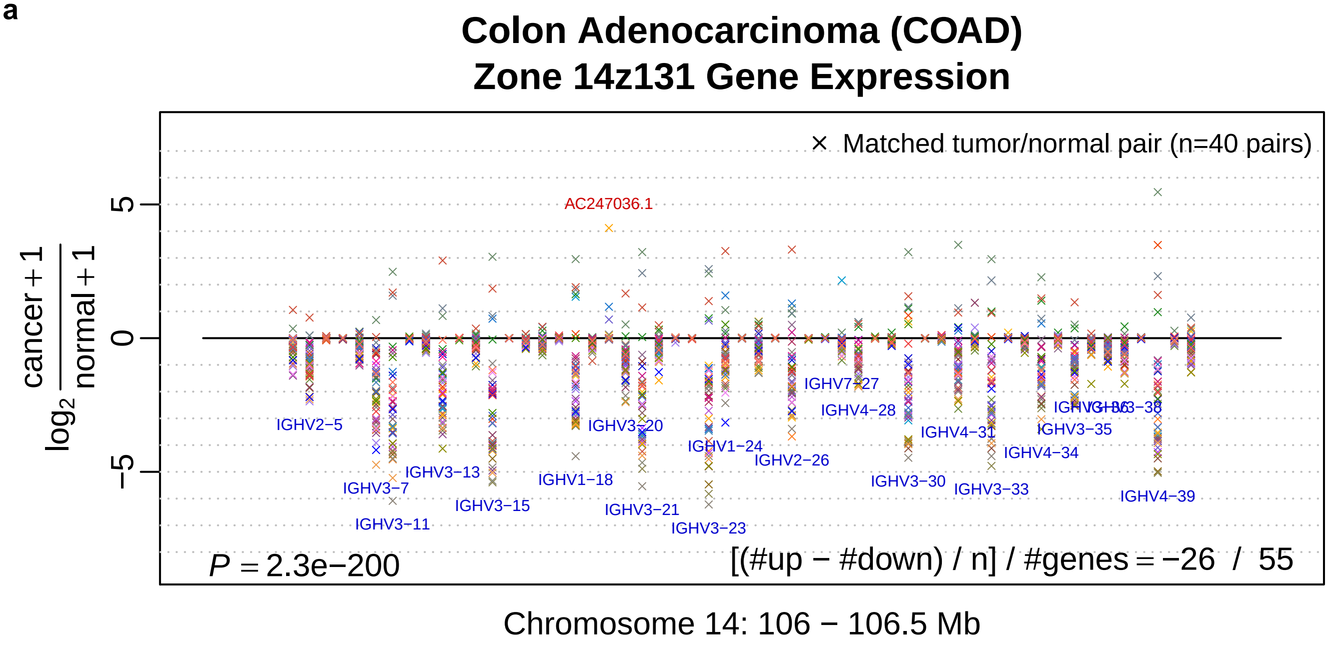

b

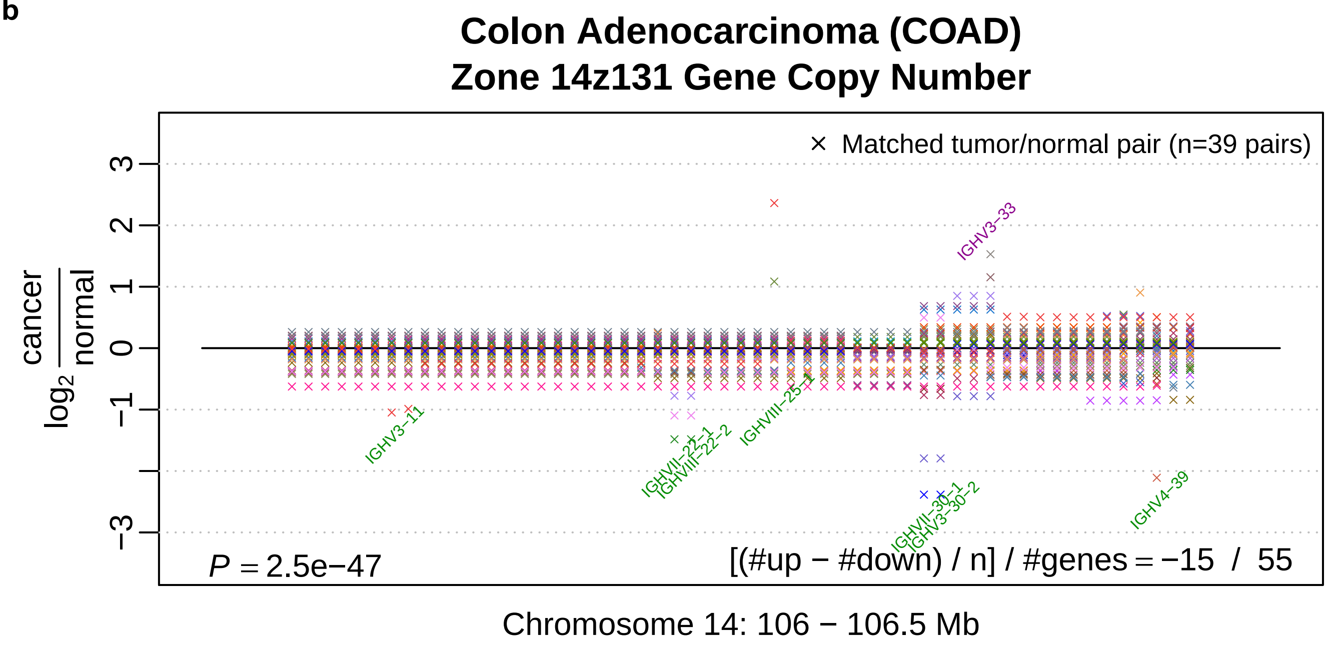

Supplementary Figure S3.21: The second most statistically significant polarized regulation zone in COAD. a, The gene expression log ratio of cancer to normal for each gene within the zone in each patient. **b**, The somatic copy number log ratio of cancer to normal for each gene within the zone in each patient. See the full legend on page 3.

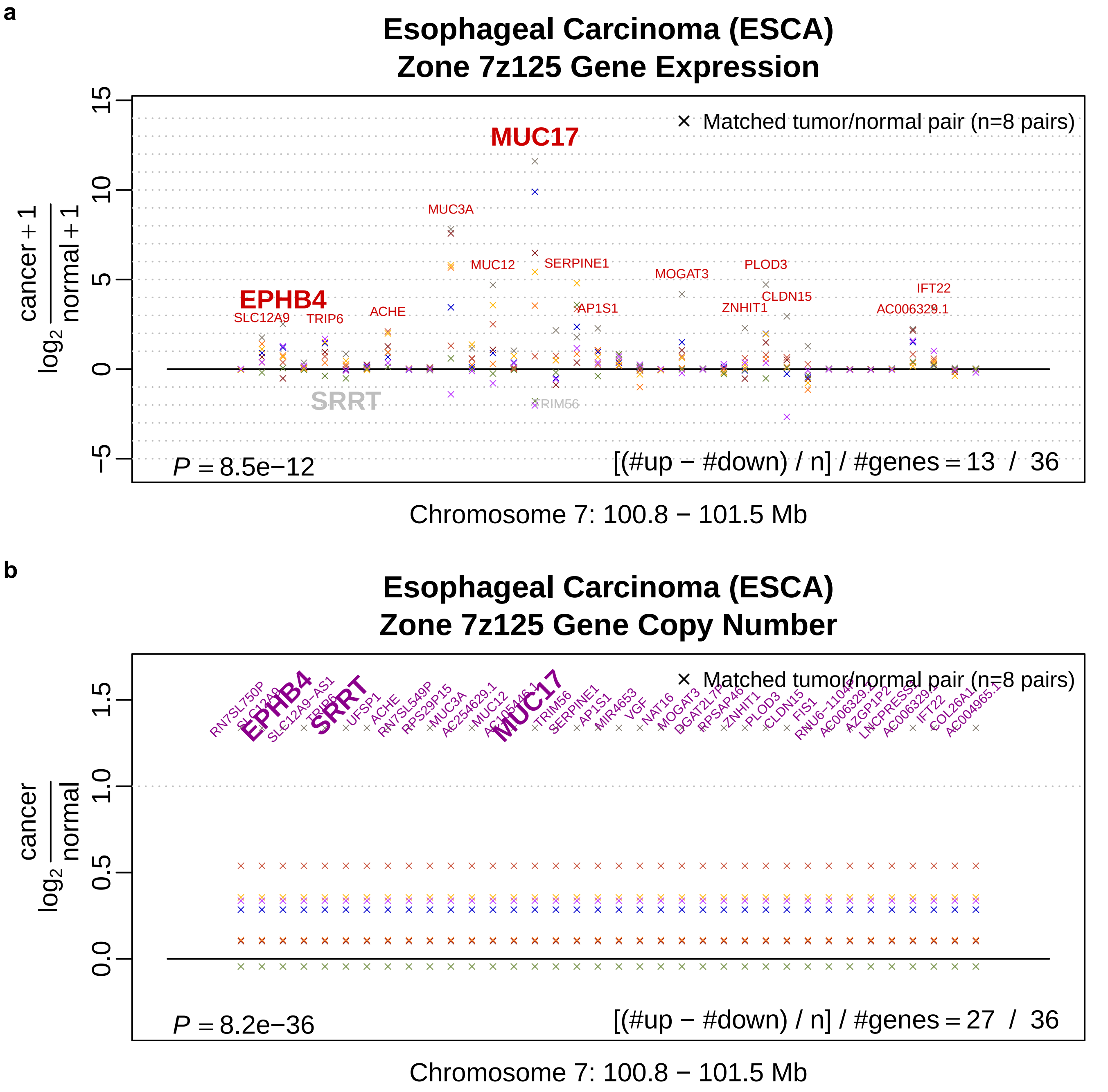

Supplementary Figure S3.22: The second most statistically significant polarized regulation zone in ESCA. a, The gene expression log ratio of cancer to normal for each gene within the zone in each patient. **b**, The somatic copy number log ratio of cancer to normal for each gene within the zone in each patient. See the full legend on page 3.

a

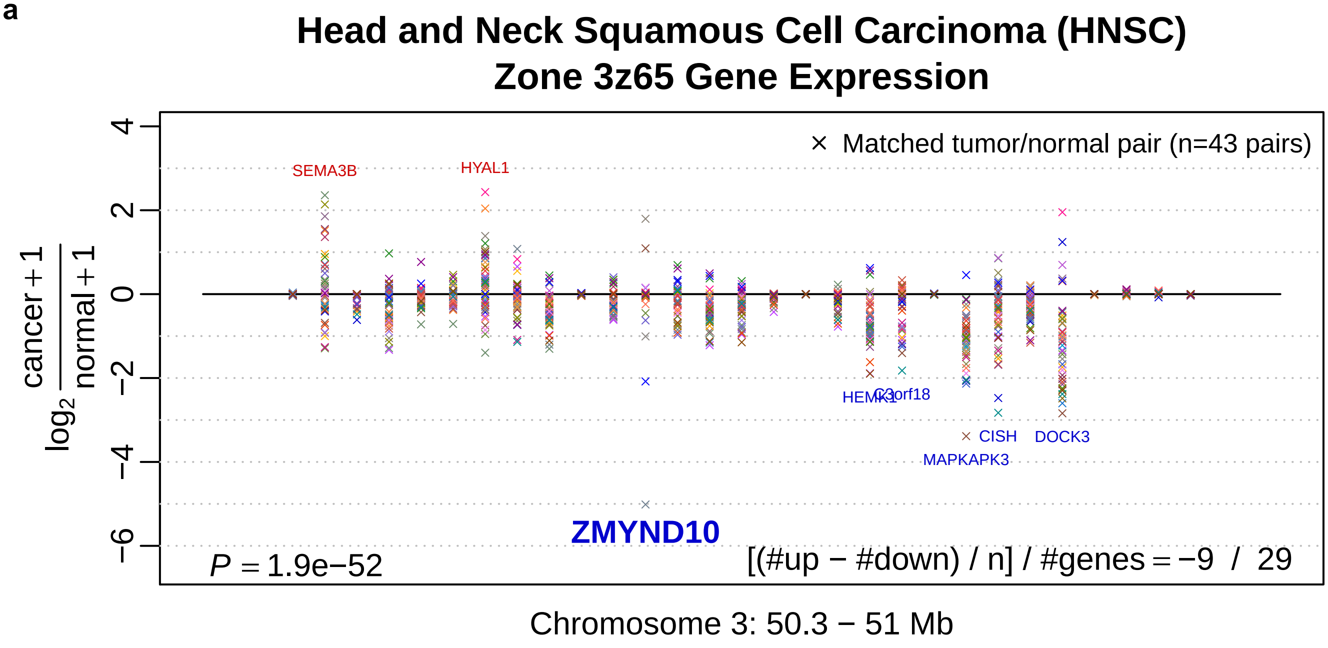

b

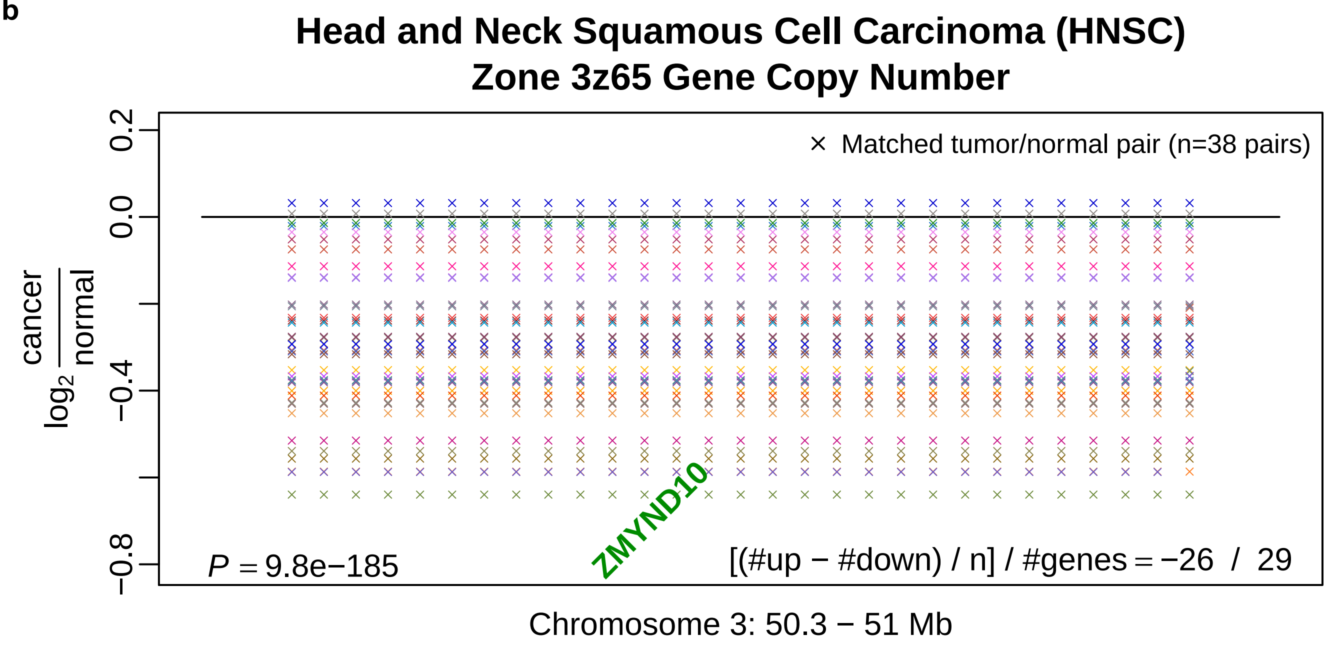

Supplementary Figure S3.23: The second most statistically significant polarized regulation zone in HNSC. a, The gene expression log ratio of cancer to normal for each gene within the zone in each patient. **b**, The somatic copy number log ratio of cancer to normal for each gene within the zone in each patient. See the full legend on page 3.

a

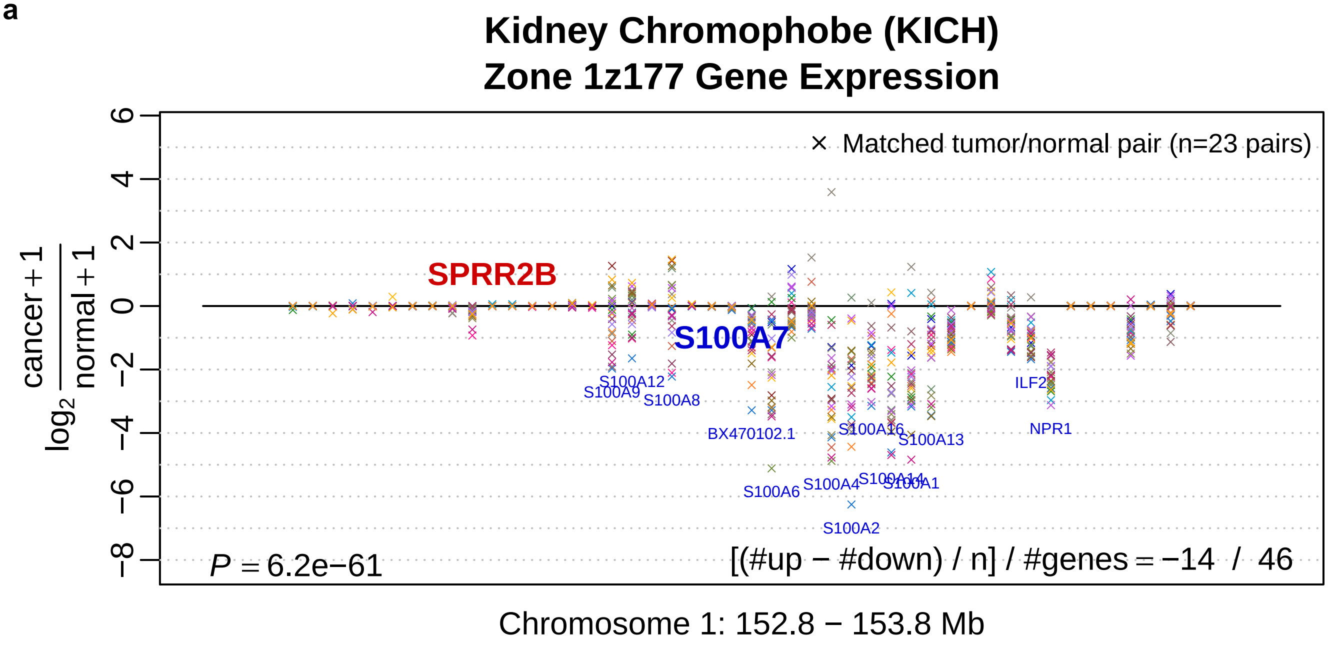

b

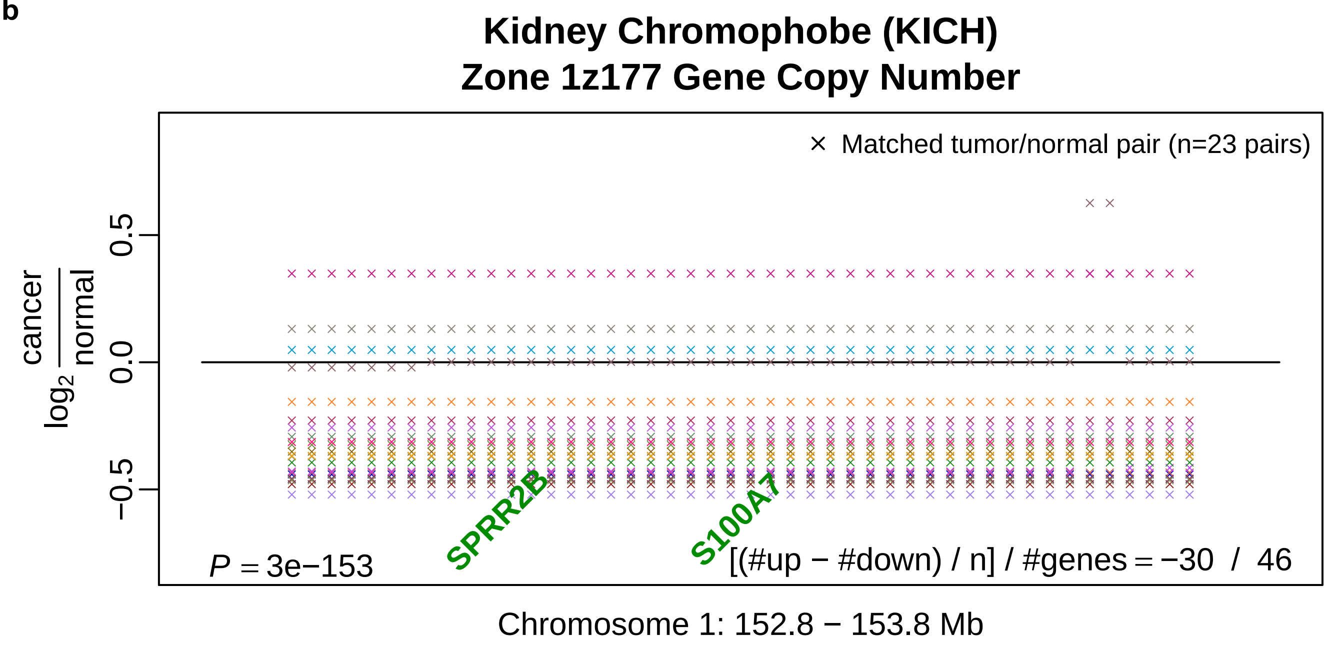

Supplementary Figure S3.24: The second most statistically significant polarized regulation zone in KICH. a, The gene expression log ratio of cancer to normal for each gene within the zone in each patient. **b**, The somatic copy number log ratio of cancer to normal for each gene within the zone in each patient. See the full legend on page 3.

a

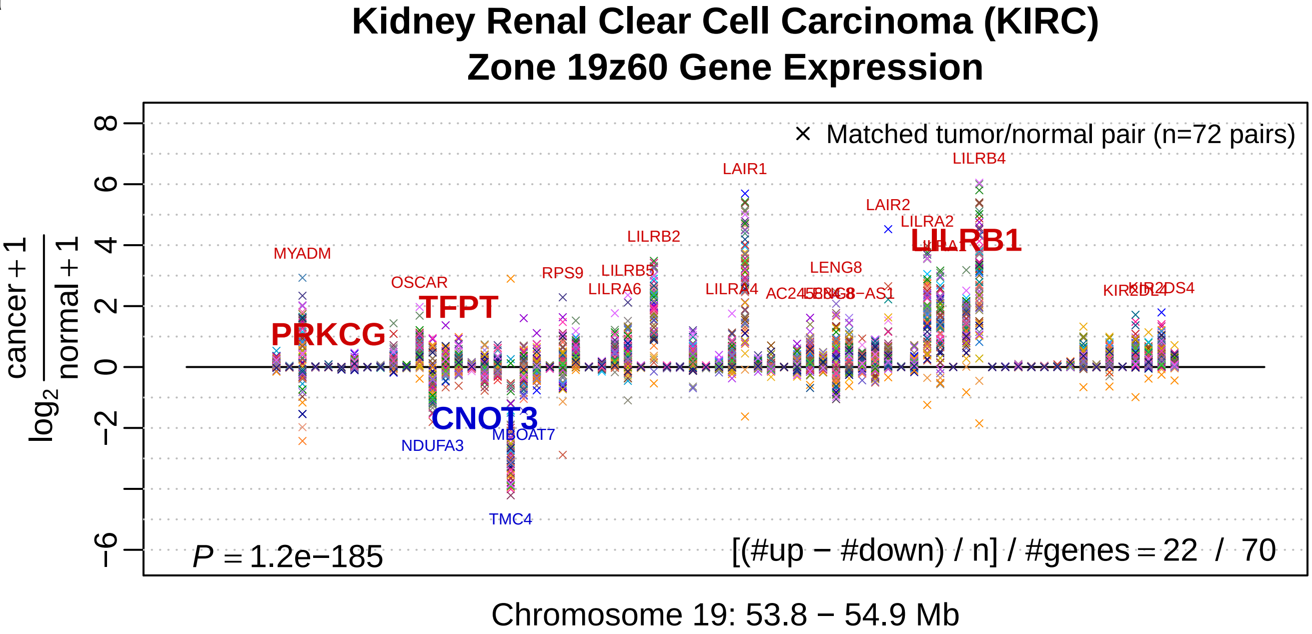

b

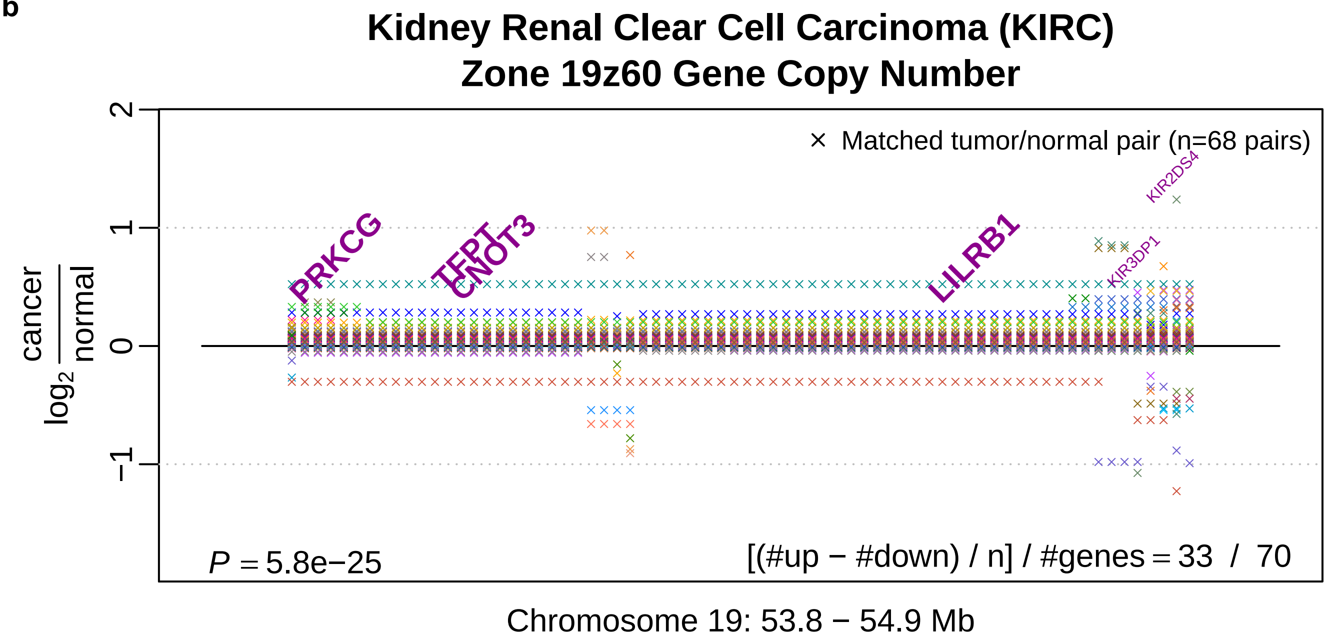

Supplementary Figure S3.25: The second most statistically significant polarized regulation zone in KIRC. a, The gene expression log ratio of cancer to normal for each gene within the zone in each patient. **b**, The somatic copy number log ratio of cancer to normal for each gene within the zone in each patient. See the full legend on page 3.

a

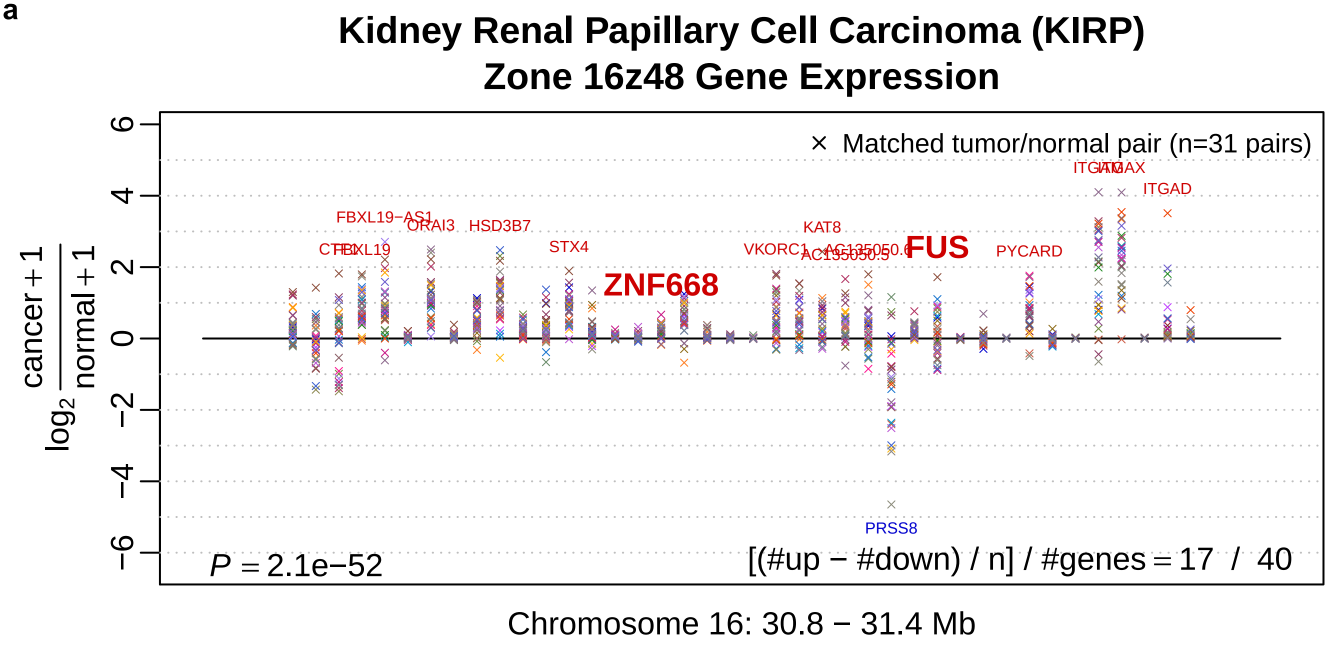

b

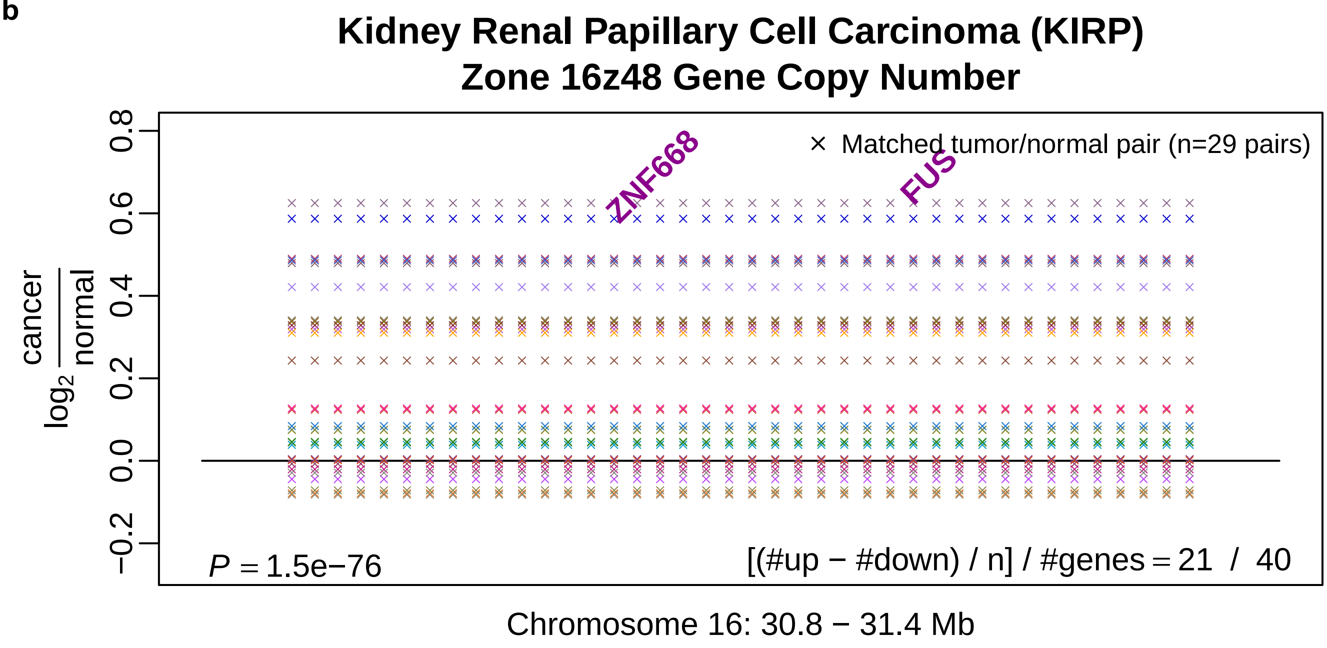

Supplementary Figure S3.26: The second most statistically significant polarized regulation zone in KIRP. a, The gene expression log ratio of cancer to normal for each gene within the zone in each patient. **b**, The somatic copy number log ratio of cancer to normal for each gene within the zone in each patient. See the full legend on page 3.

a

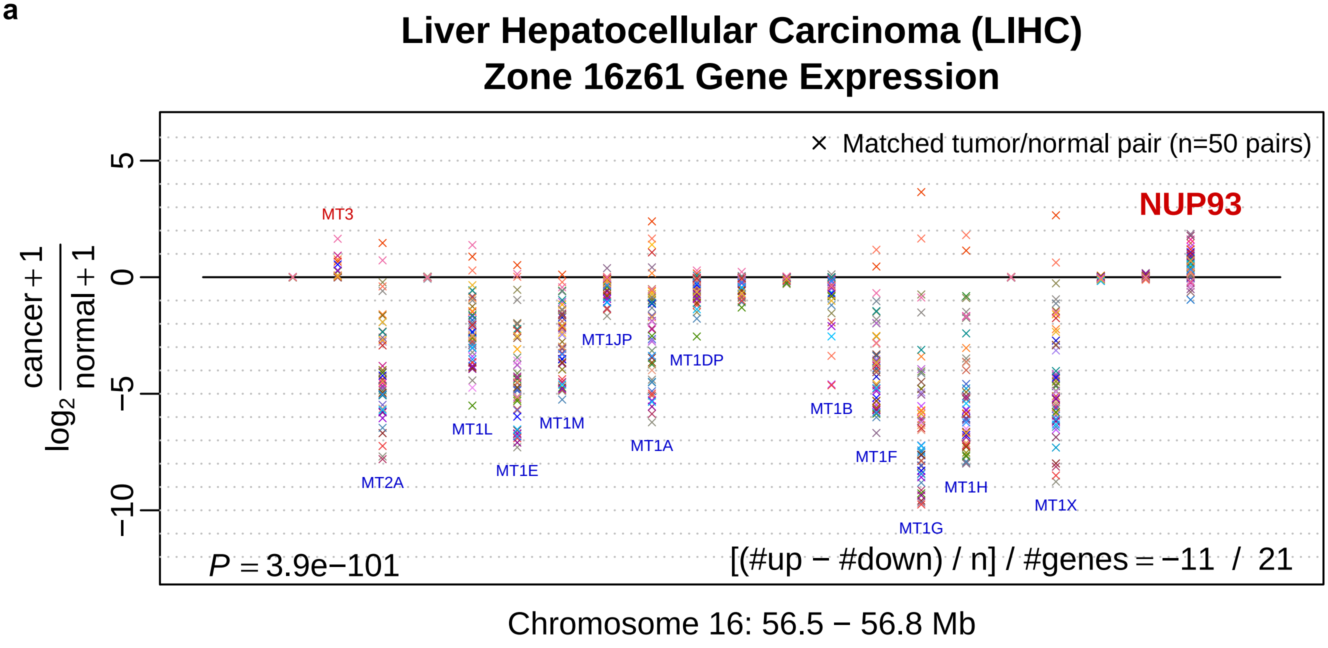

b

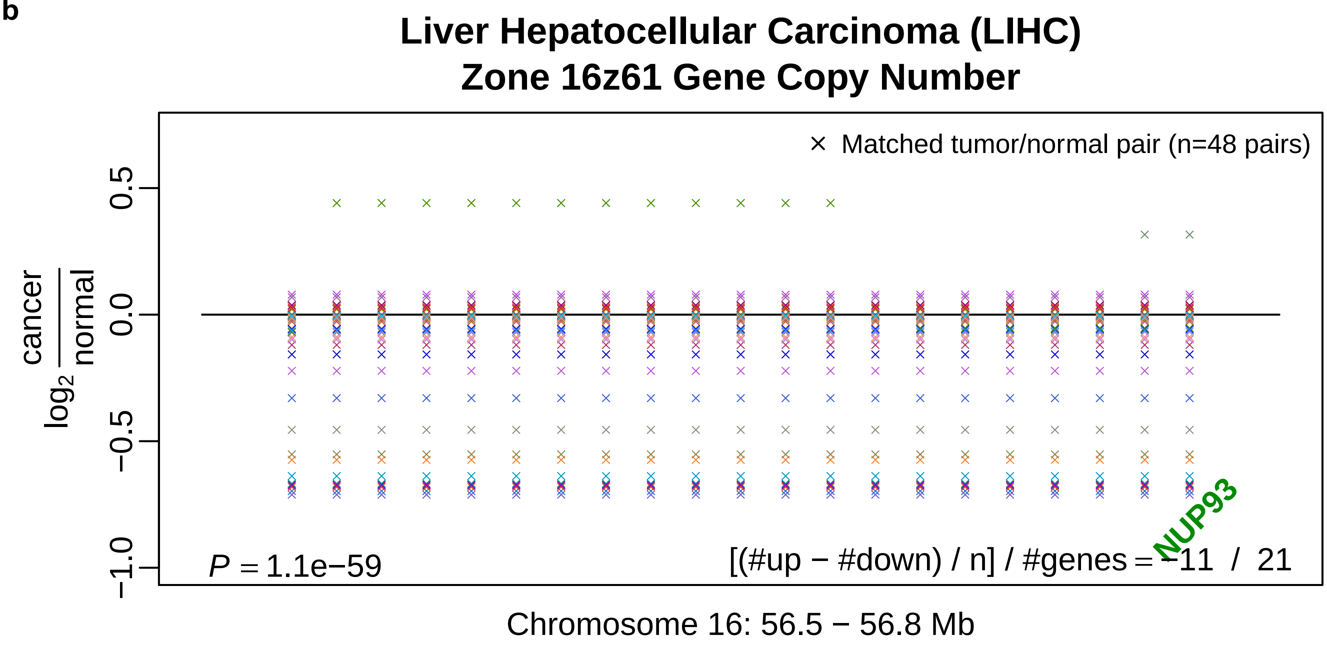

Supplementary Figure S3.27: The second most statistically significant polarized regulation zone in LIHC. a, The gene expression log ratio of cancer to normal for each gene within the zone in each patient. **b**, The somatic copy number log ratio of cancer to normal for each gene within the zone in each patient. See the full legend on page 3.

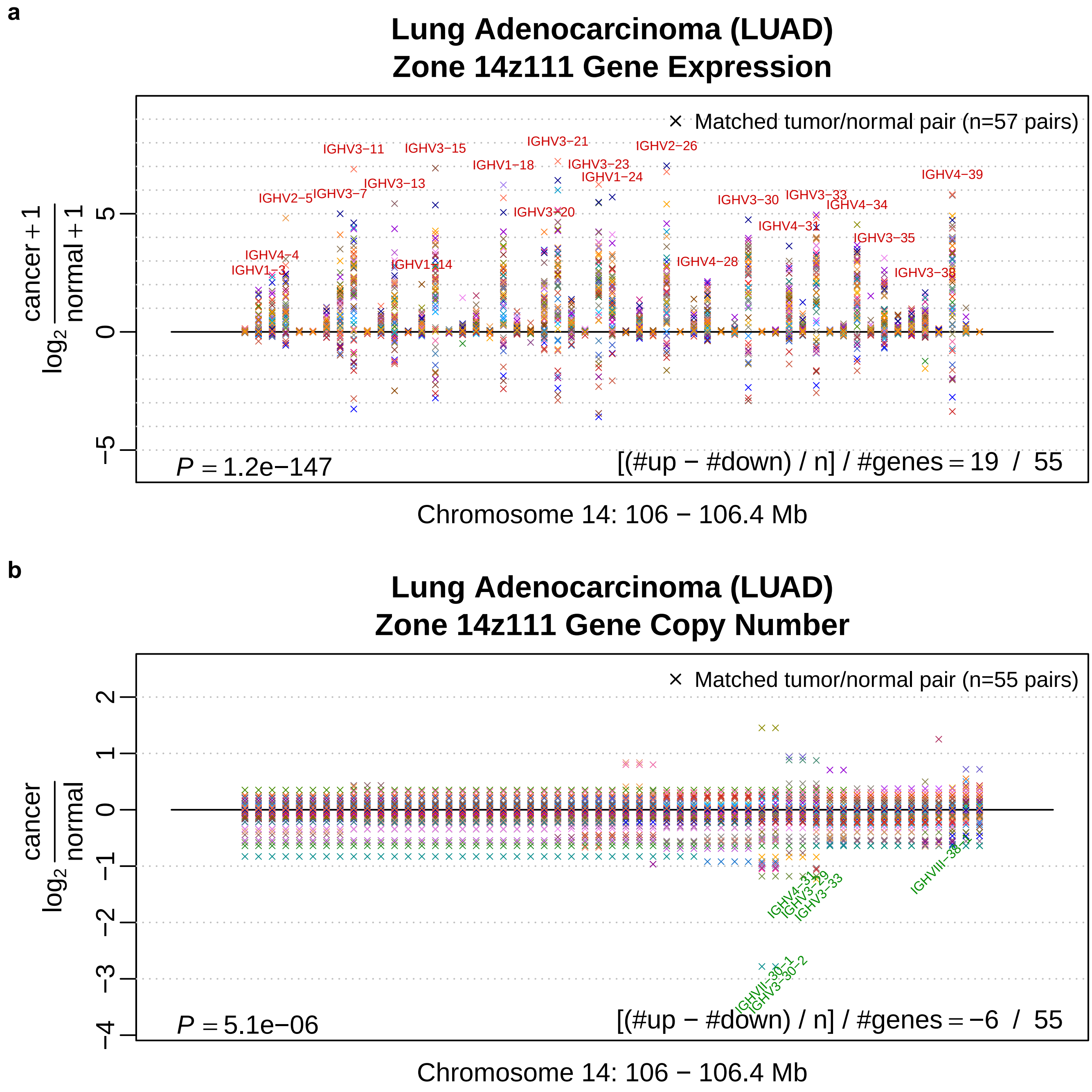

Supplementary Figure S3.28: The second most statistically significant polarized regulation zone in LUAD. a, The gene expression log ratio of cancer to normal for each gene within the zone in each patient. **b**, The somatic copy number log ratio of cancer to normal for each gene within the zone in each patient. See the full legend on page 3.

a

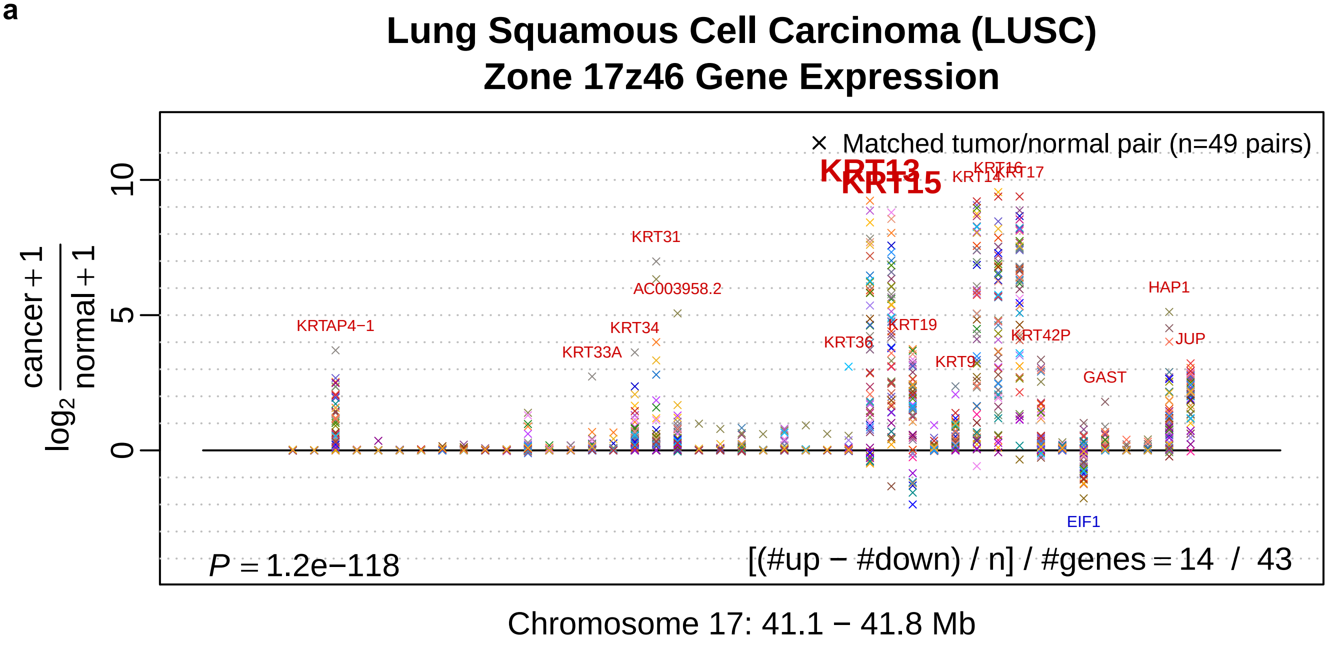

b

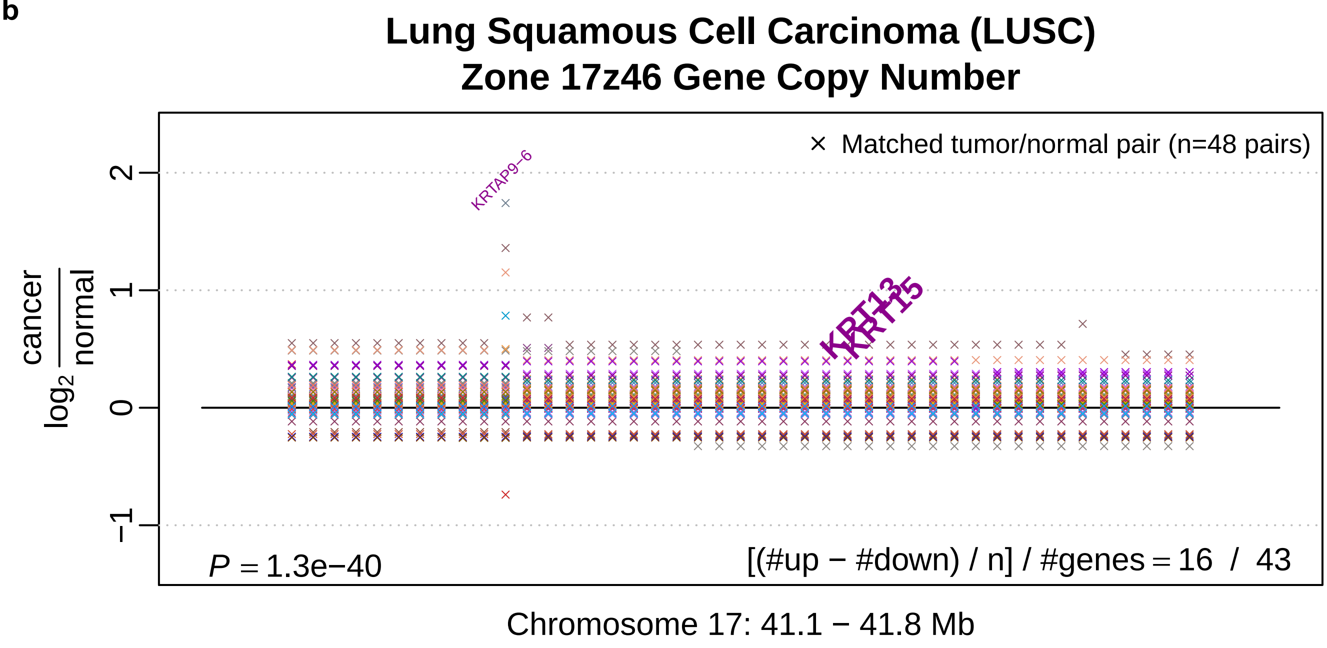

Supplementary Figure S3.29: The second most statistically significant polarized regulation zone in LUSC. a, The gene expression log ratio of cancer to normal for each gene within the zone in each patient. **b**, The somatic copy number log ratio of cancer to normal for each gene within the zone in each patient. See the full legend on page 3.

a

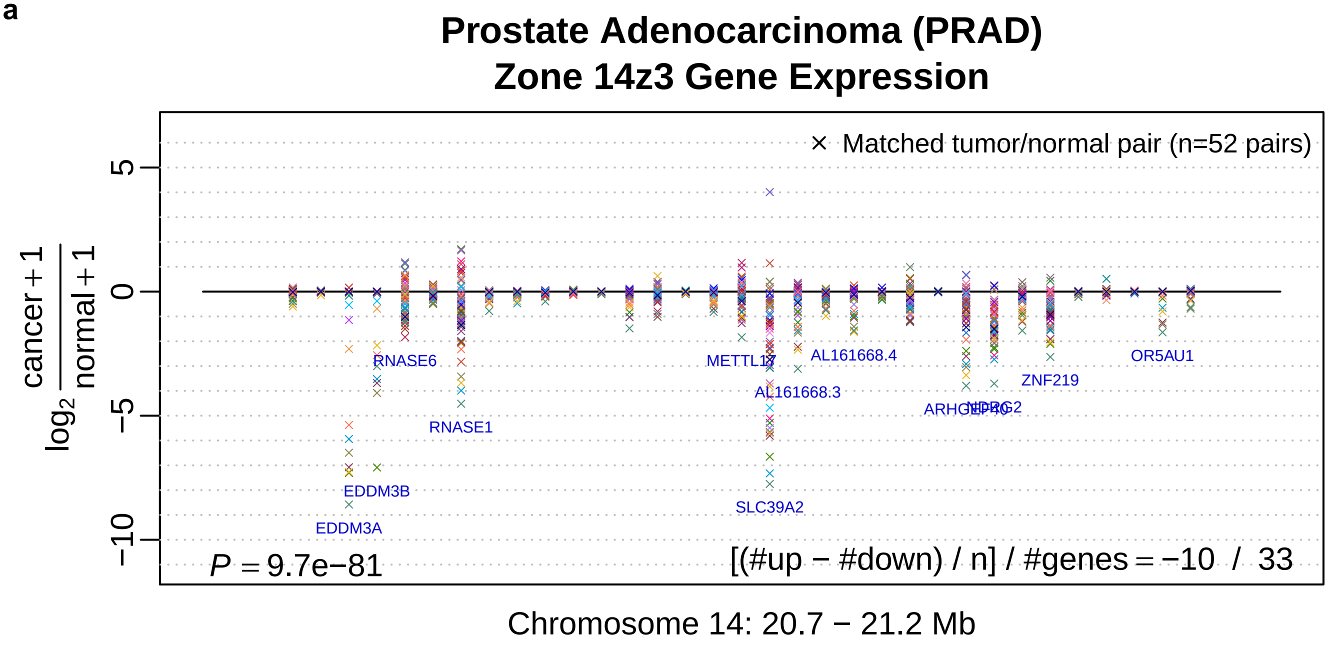

b

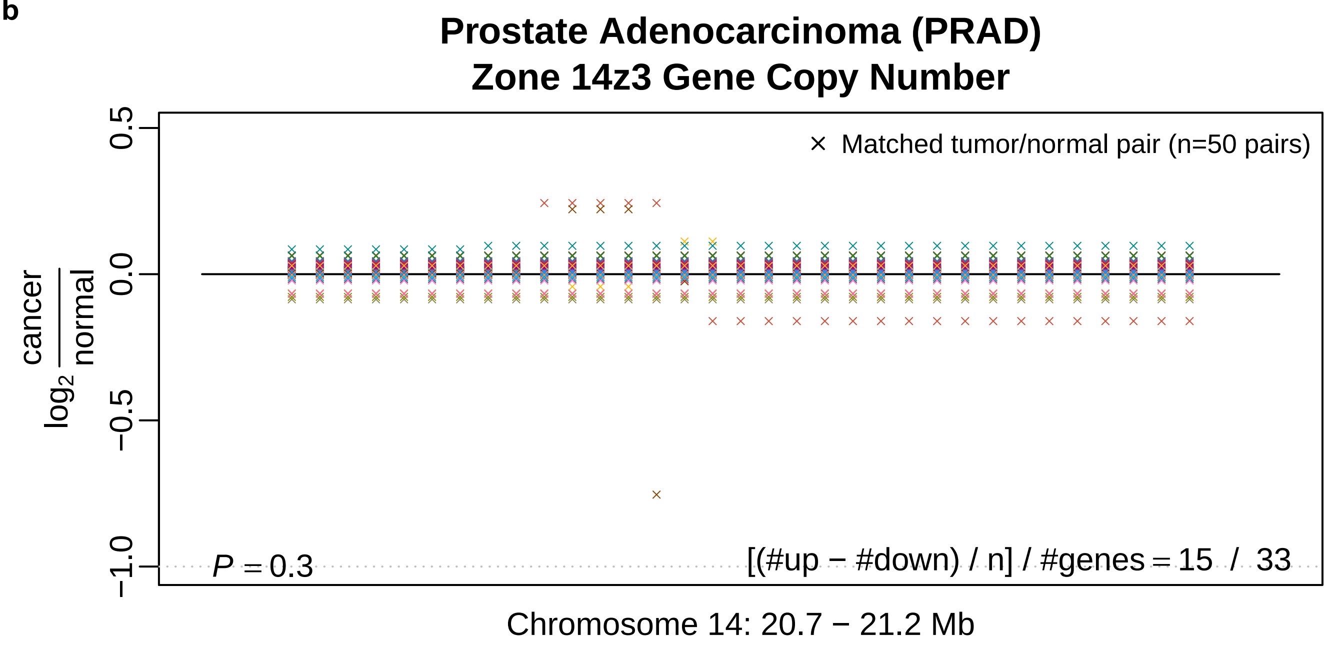

Supplementary Figure S3.30: The second most statistically significant polarized regulation zone in PRAD. a, The gene expression log ratio of cancer to normal for each gene within the zone in each patient. **b**, The somatic copy number log ratio of cancer to normal for each gene within the zone in each patient. See the full legend on page 3.

a

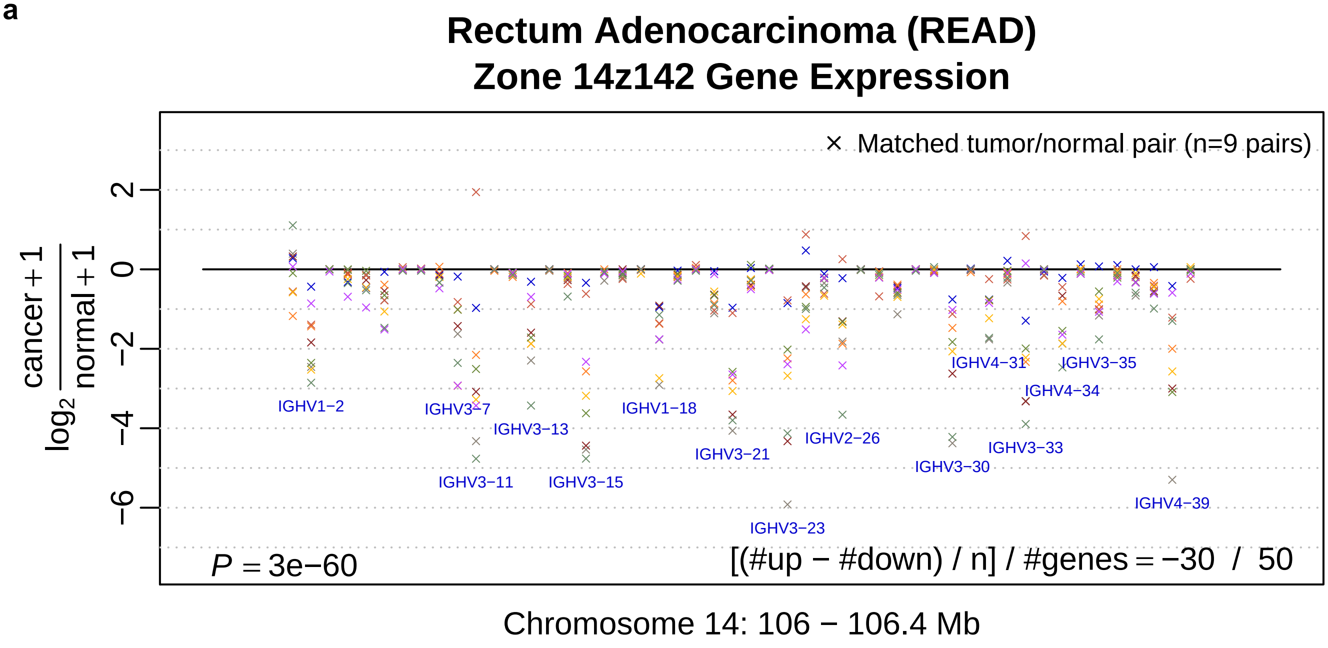

b

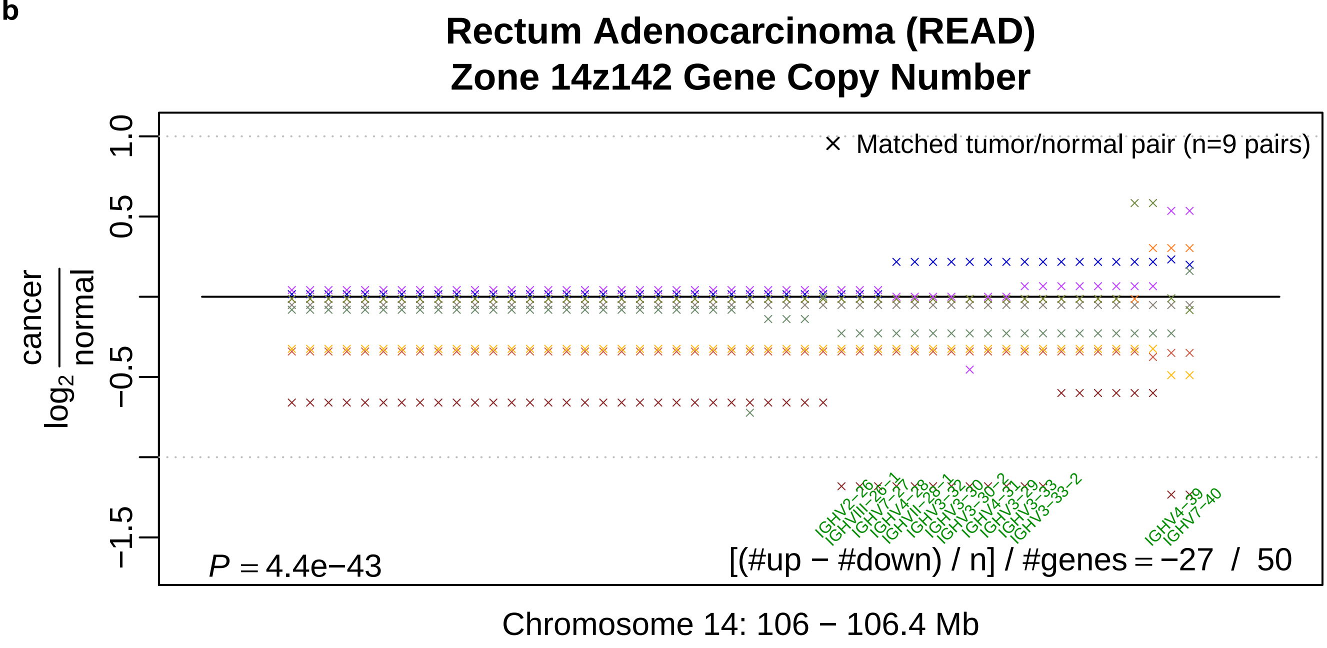

Supplementary Figure S3.31: The second most statistically significant polarized regulation zone in READ. a, The gene expression log ratio of cancer to normal for each gene within the zone in each patient. **b**, The somatic copy number log ratio of cancer to normal for each gene within the zone in each patient. See the full legend on page 3.

a

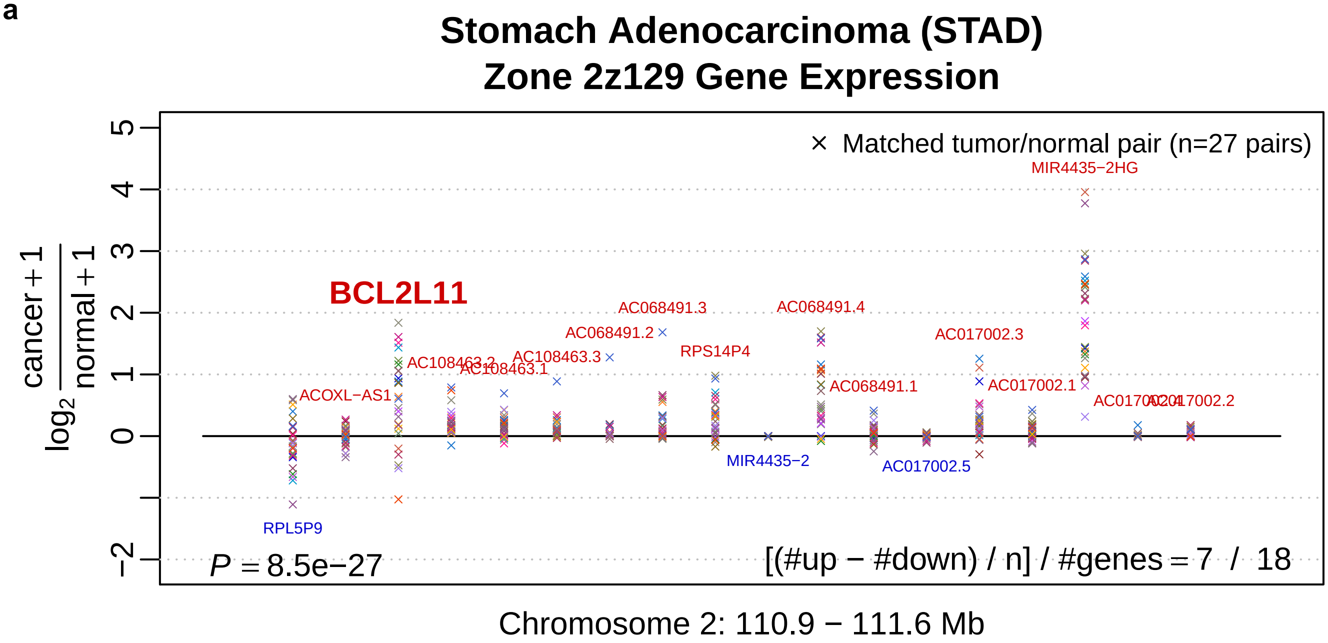

b

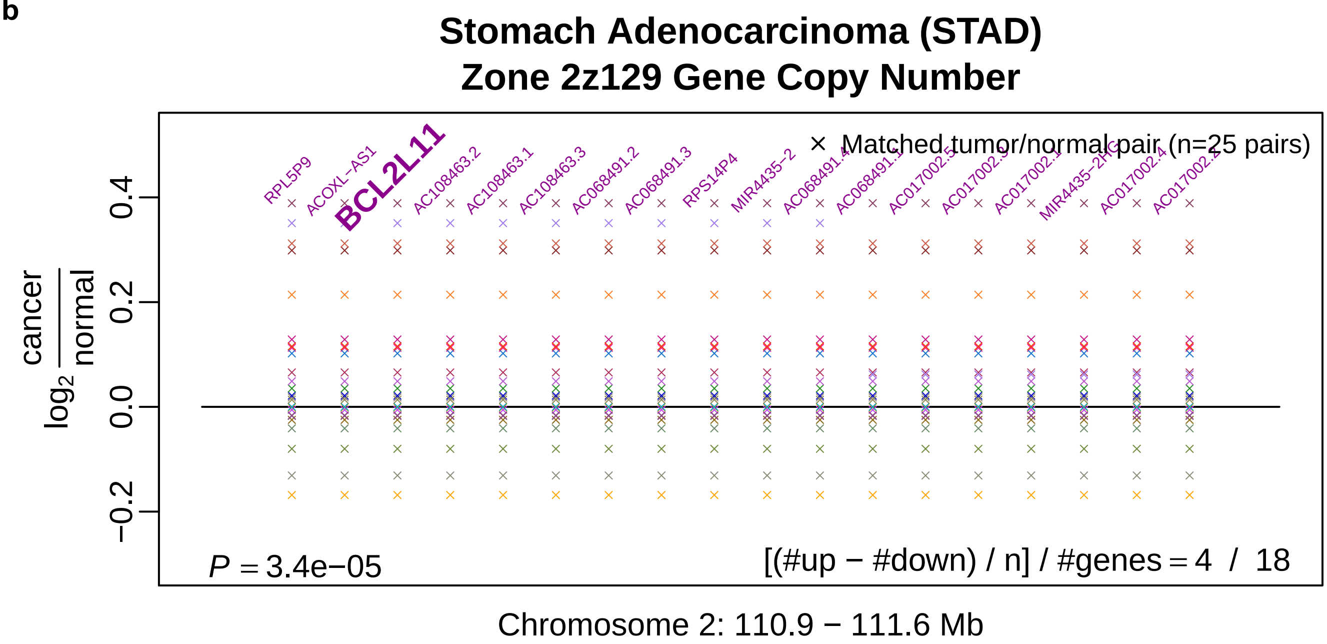

Supplementary Figure S3.32: The second most statistically significant polarized regulation zone in STAD. a, The gene expression log ratio of cancer to normal for each gene within the zone in each patient. **b**, The somatic copy number log ratio of cancer to normal for each gene within the zone in each patient. See the full legend on page 3.

a

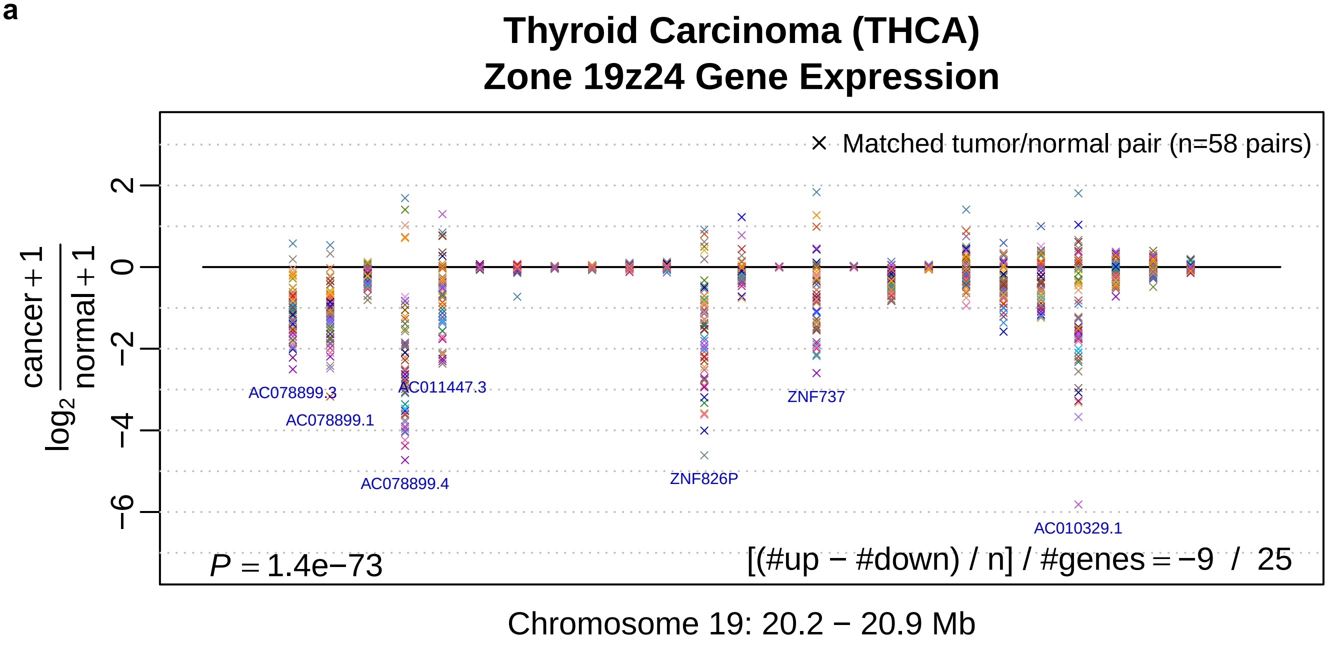

b

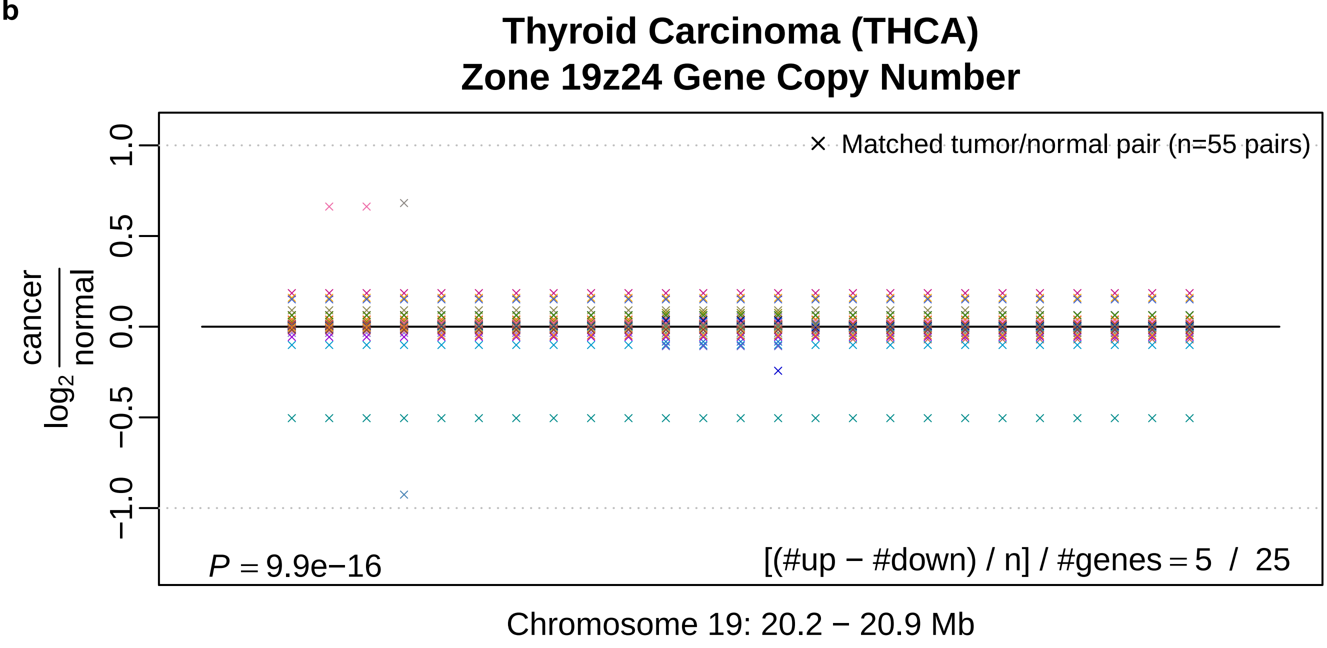

Supplementary Figure S3.33: The second most statistically significant polarized regulation zone in THCA. a, The gene expression log ratio of cancer to normal for each gene within the zone in each patient. **b**, The somatic copy number log ratio of cancer to normal for each gene within the zone in each patient. See the full legend on page 3.

a

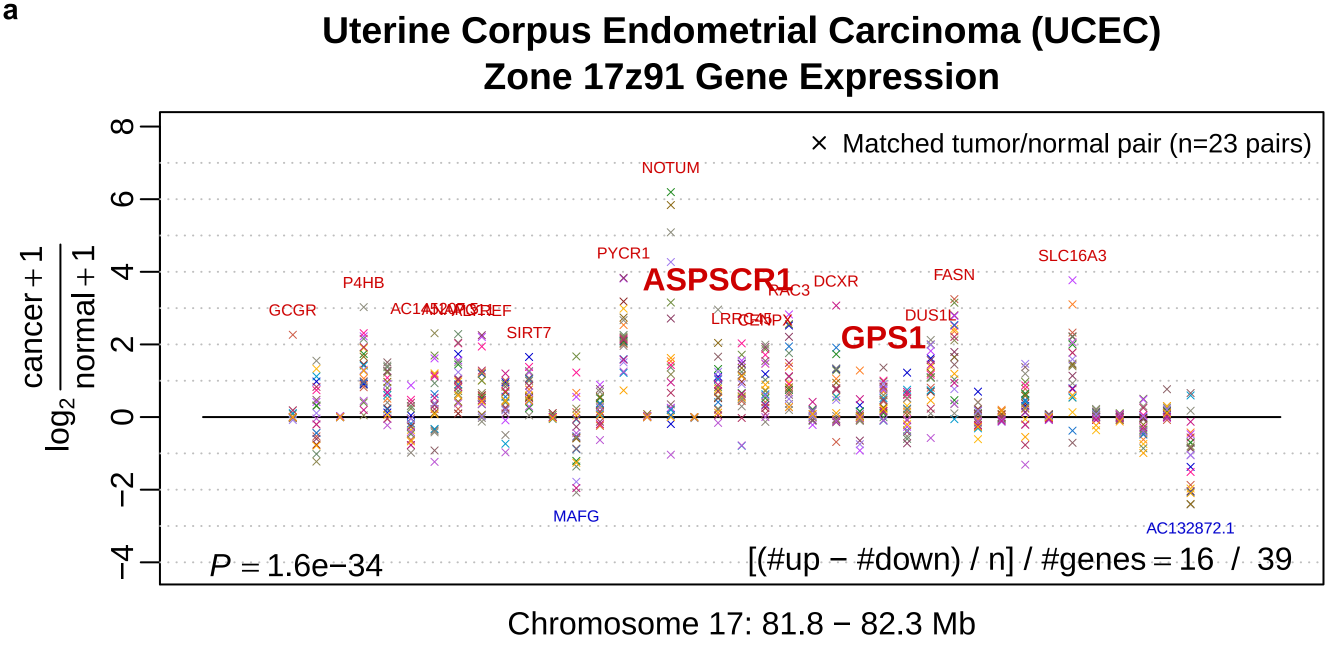

b

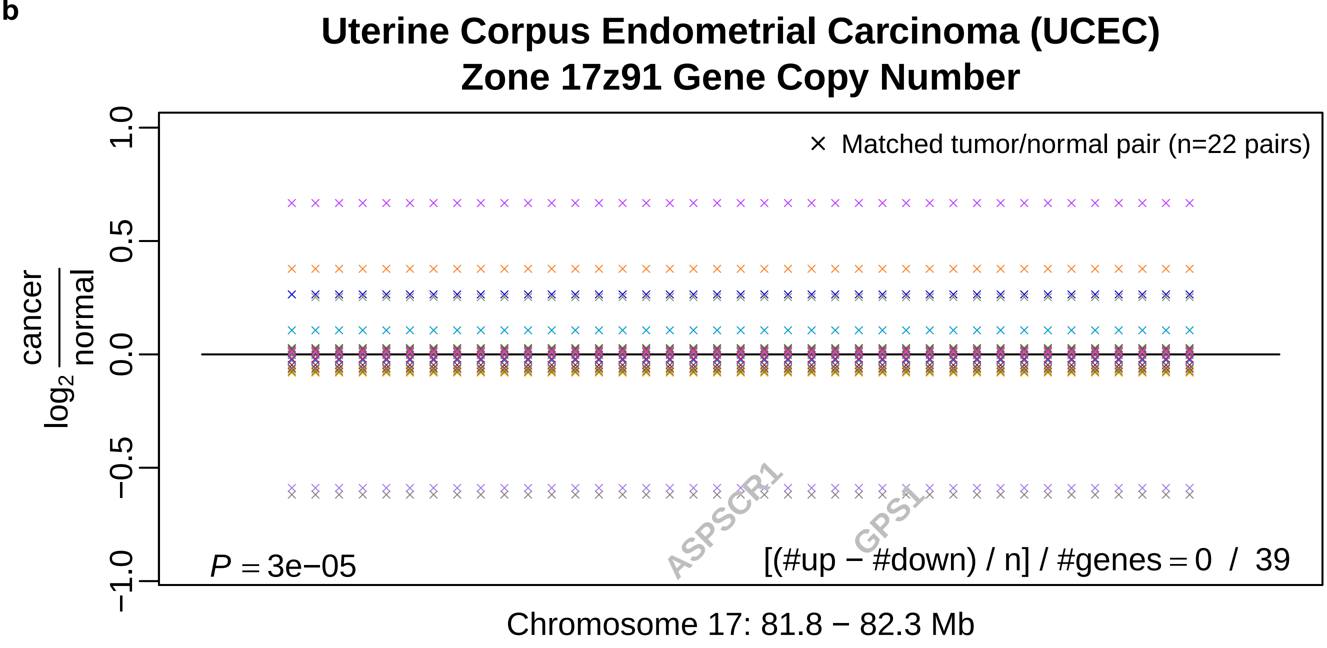

Supplementary Figure S3.34: The second most statistically significant polarized regulation zone in UCEC. a, The gene expression log ratio of cancer to normal for each gene within the zone in each patient. **b**, The somatic copy number log ratio of cancer to normal for each gene within the zone in each patient. See the full legend on page 3.

2 Third most polarized regulation zones of 17 cancer types

a

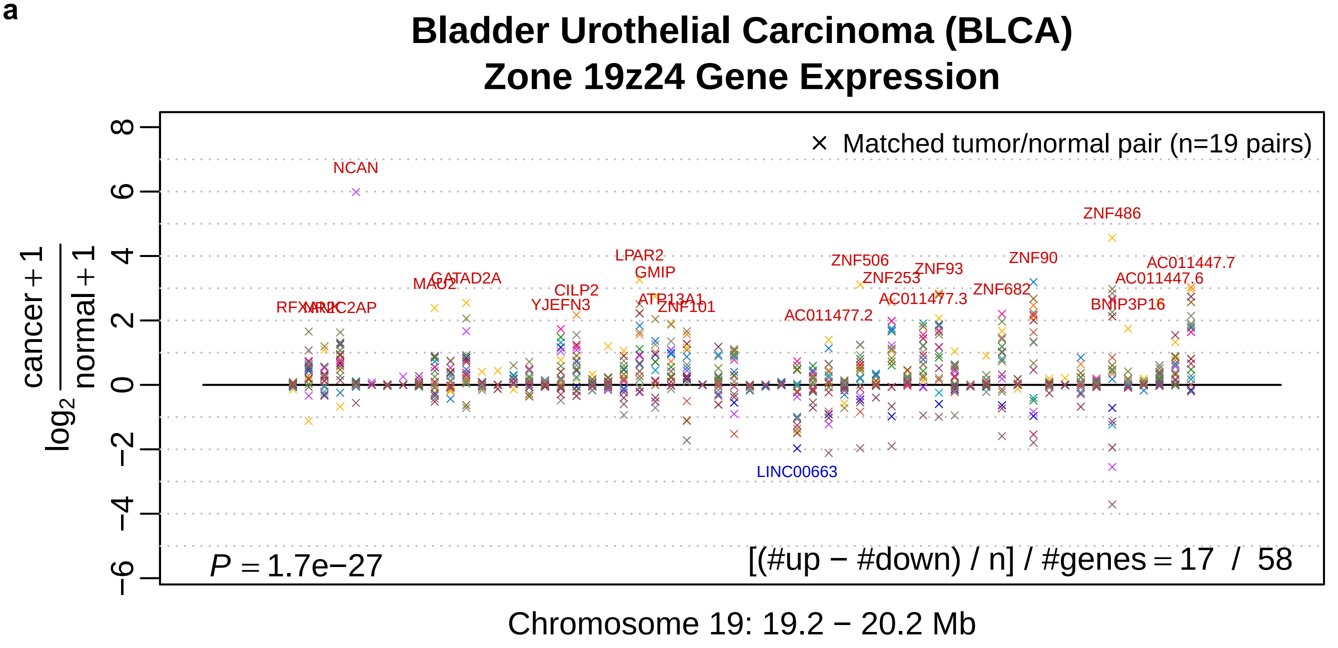

b

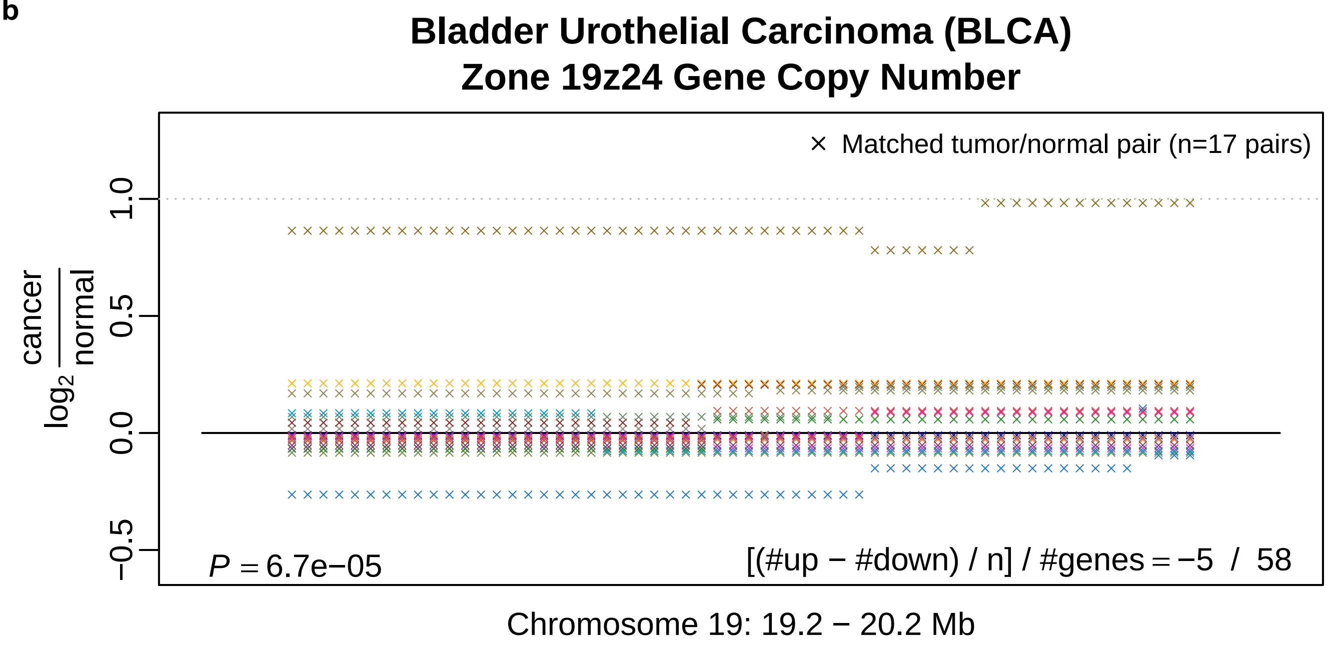

Supplementary Figure S3.35: The third most statistically significant polarized regulation zone in BLCA. a, The gene expression log ratio of cancer to normal for each gene within the zone in each patient. **b**, The somatic copy number log ratio of cancer to normal for each gene within the zone in each patient. See the full legend on page 3.

a

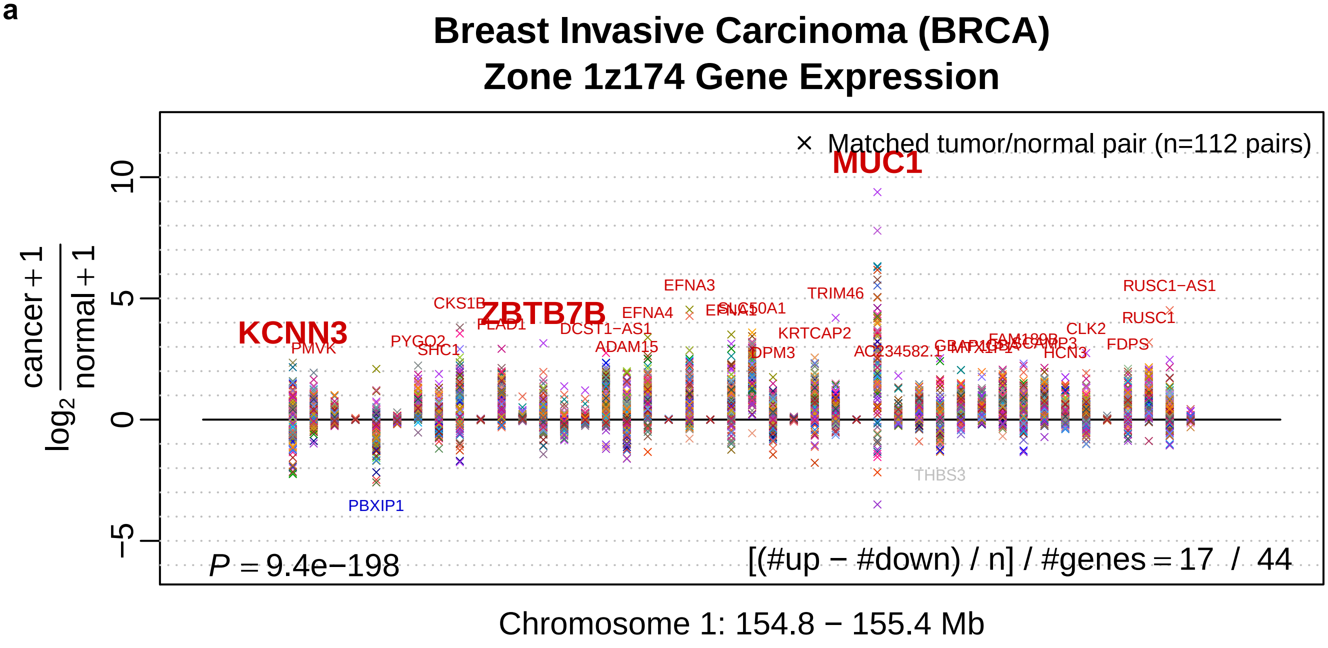

b

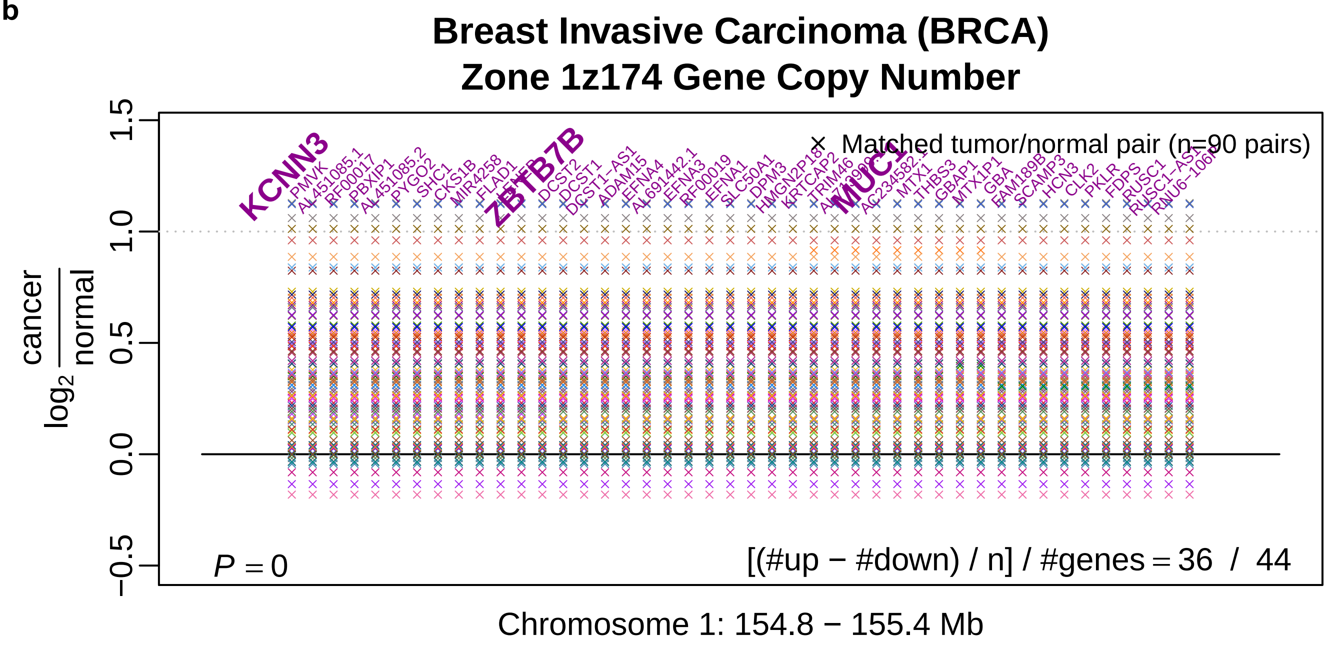

Supplementary Figure S3.36: The third most statistically significant polarized regulation zone in BRCA. a, The gene expression log ratio of cancer to normal for each gene within the zone in each patient. **b**, The somatic copy number log ratio of cancer to normal for each gene within the zone in each patient. See the full legend on page 3.

a

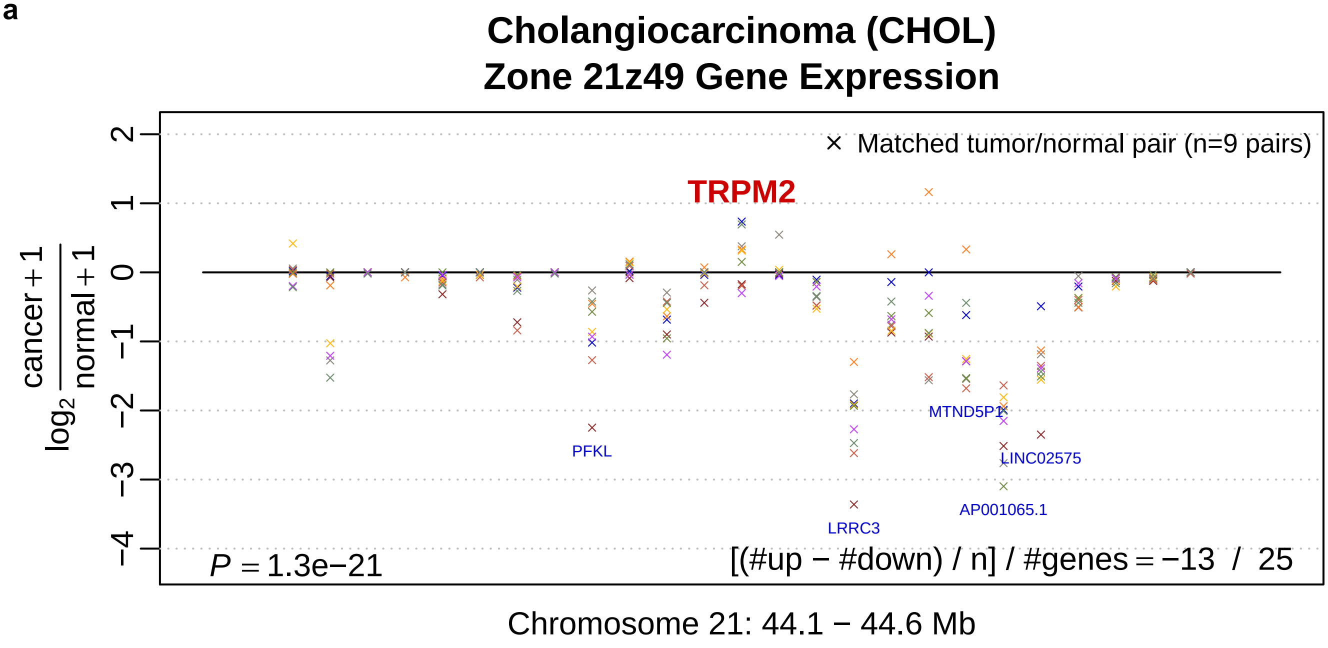

b

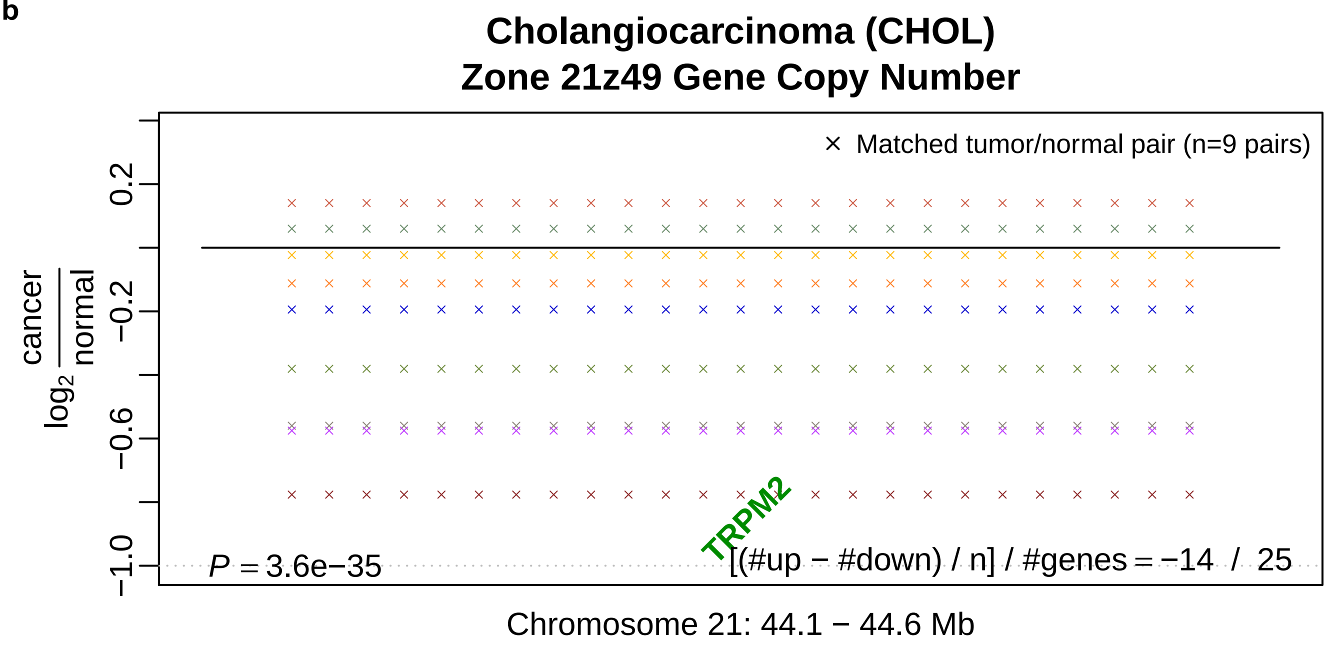

Supplementary Figure S3.37: The third most statistically significant polarized regulation zone in CHOL. a, The gene expression log ratio of cancer to normal for each gene within the zone in each patient. **b**, The somatic copy number log ratio of cancer to normal for each gene within the zone in each patient. See the full legend on page 3.

a

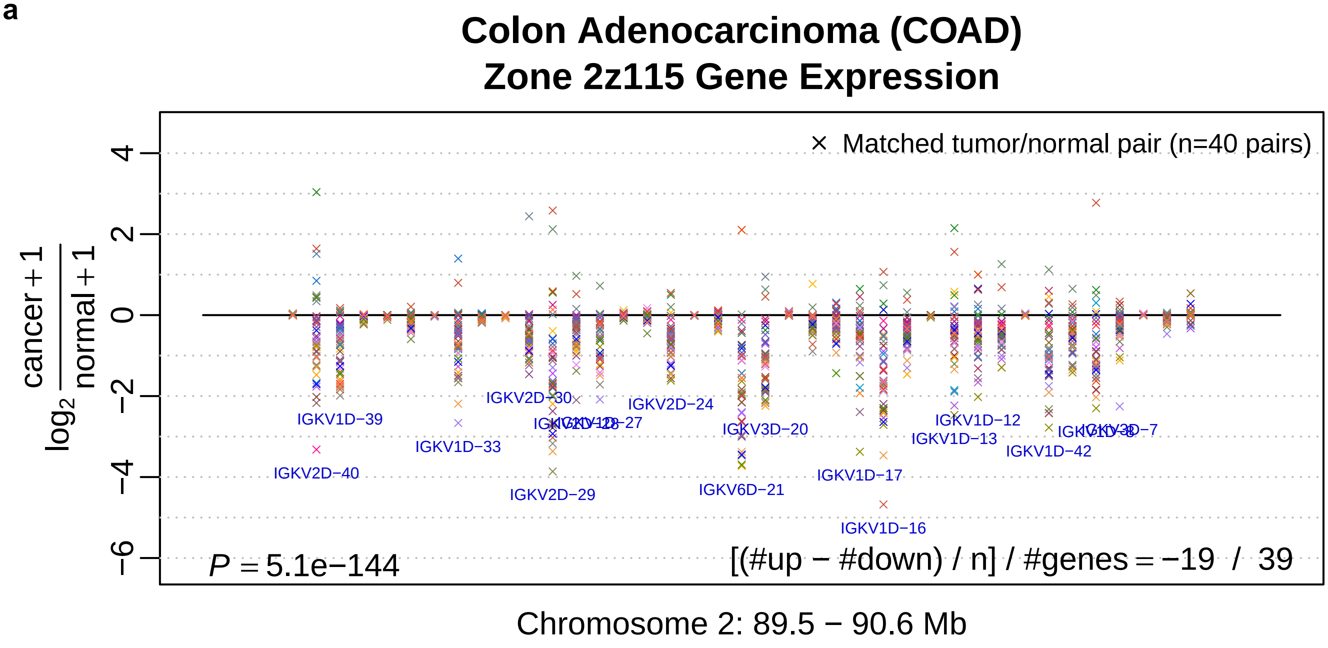

b

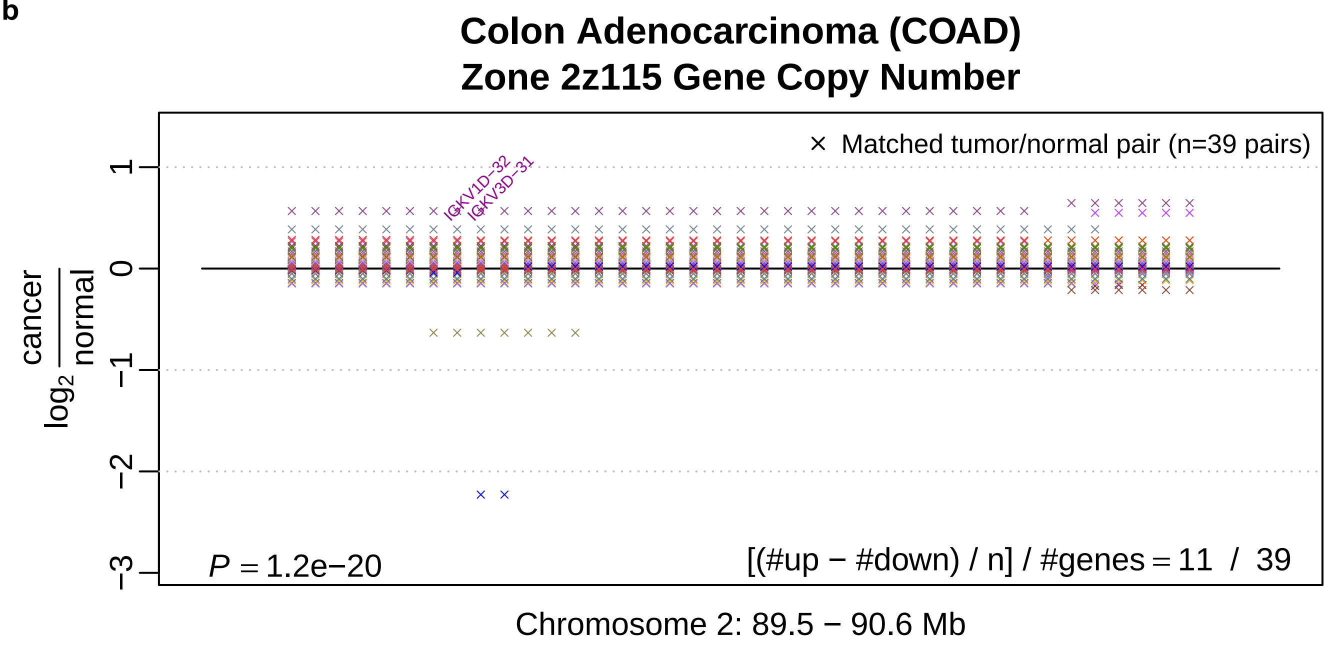

Supplementary Figure S3.38: The third most statistically significant polarized regulation zone in COAD. a, The gene expression log ratio of cancer to normal for each gene within the zone in each patient. **b**, The somatic copy number log ratio of cancer to normal for each gene within the zone in each patient. See the full legend on page 3.

a

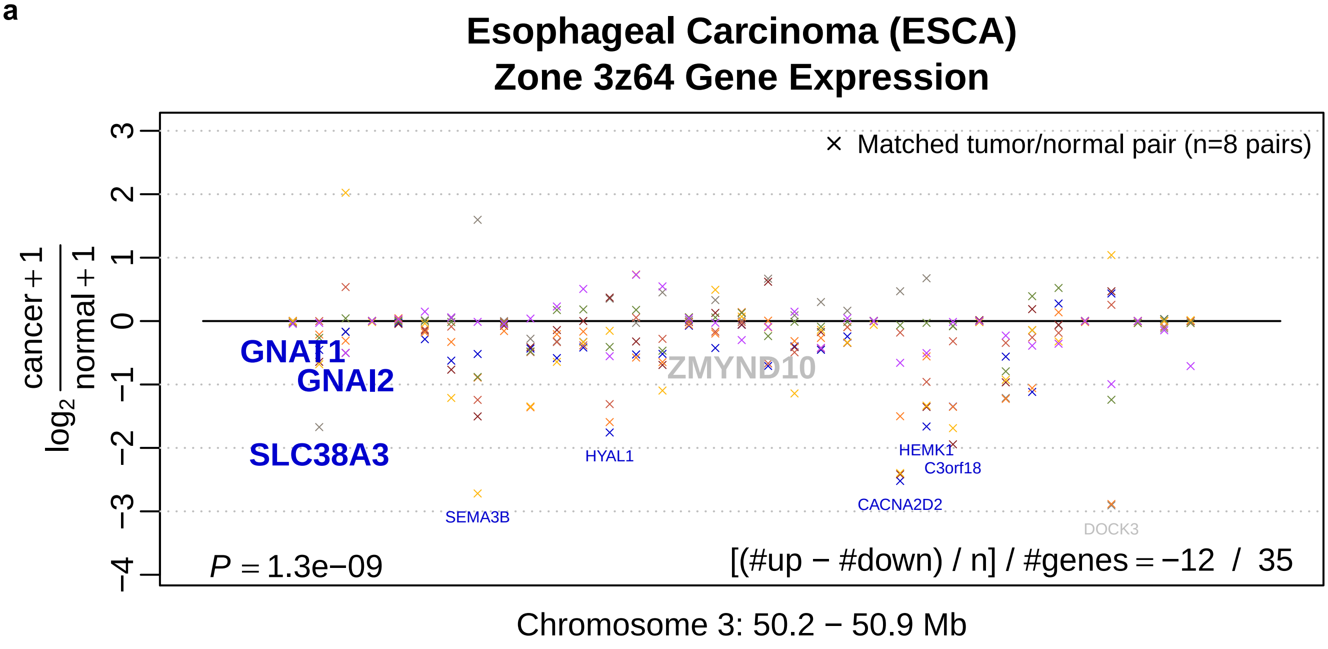

b

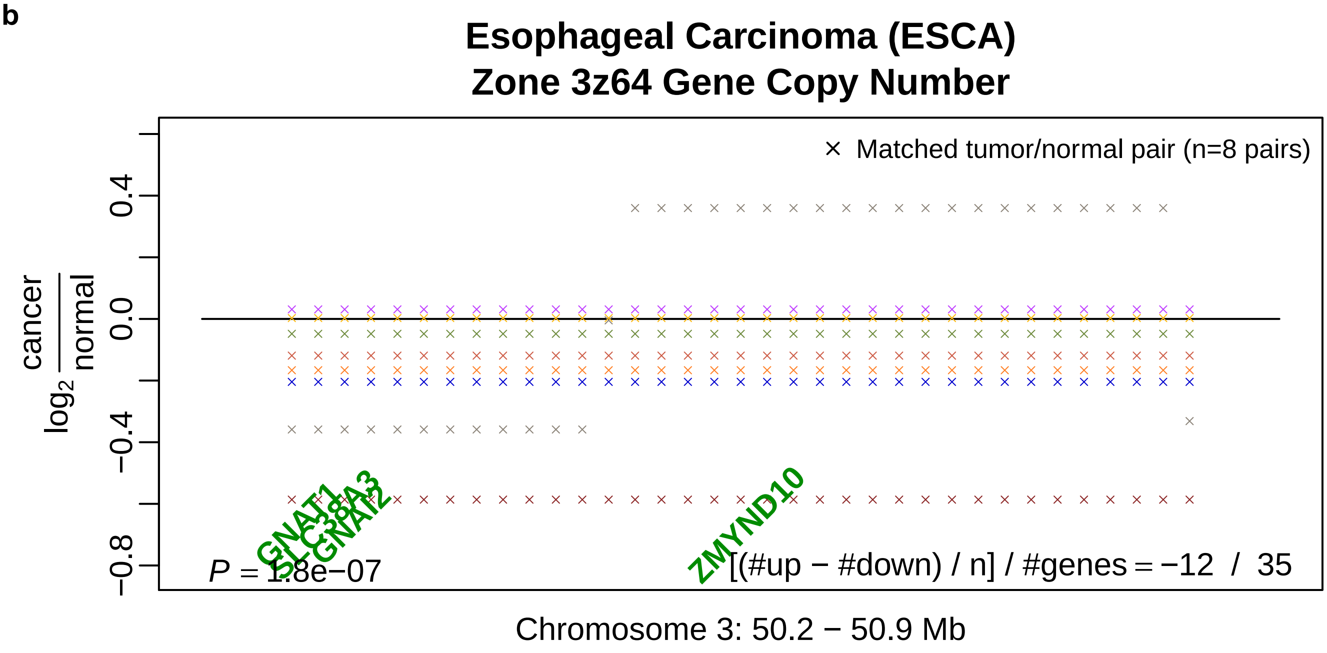

Supplementary Figure S3.39: The third most statistically significant polarized regulation zone in ESCA. a, The gene expression log ratio of cancer to normal for each gene within the zone in each patient. **b**, The somatic copy number log ratio of cancer to normal for each gene within the zone in each patient. See the full legend on page 3.

a

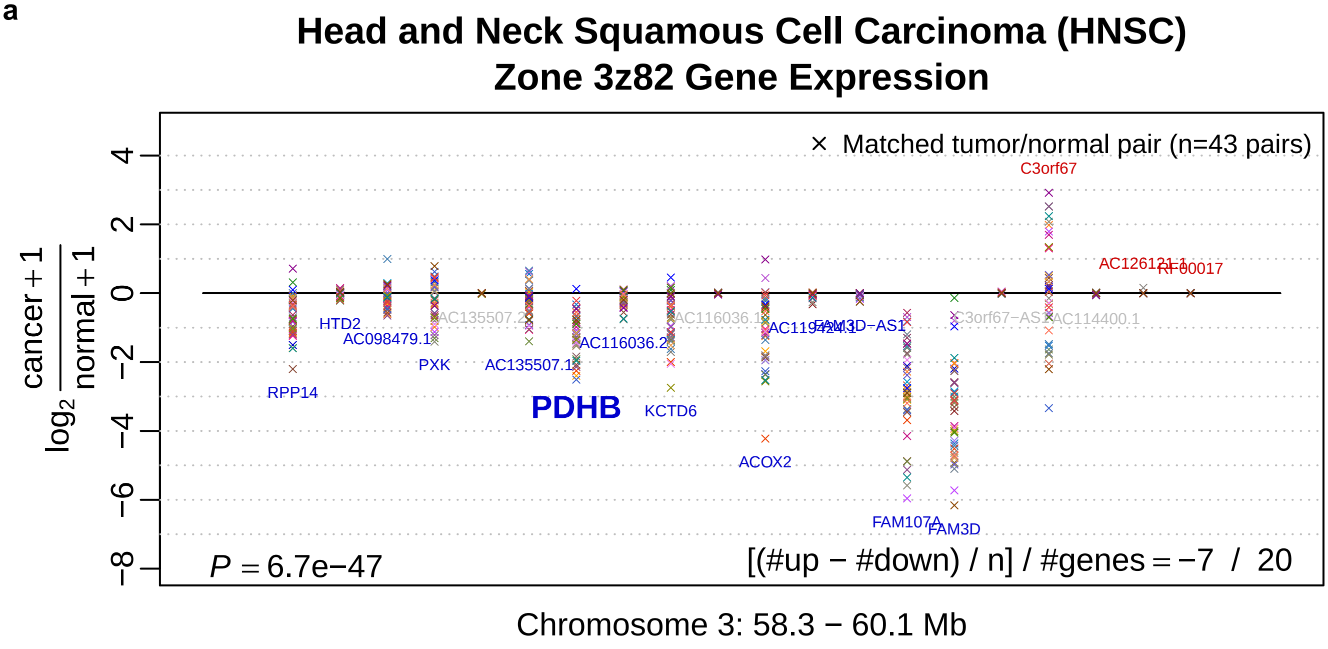

b

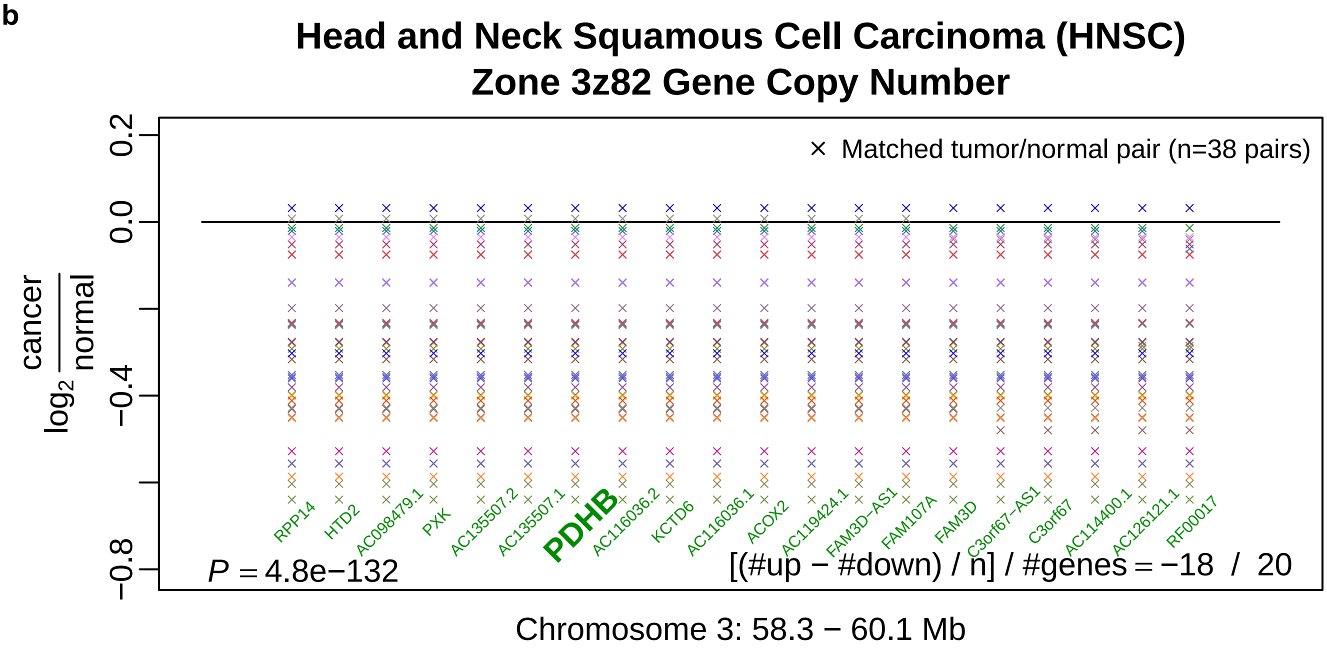

Supplementary Figure S3.40: The third most statistically significant polarized regulation zone in HNSC. a, The gene expression log ratio of cancer to normal for each gene within the zone in each patient. **b**, The somatic copy number log ratio of cancer to normal for each gene within the zone in each patient. See the full legend on page 3.

a

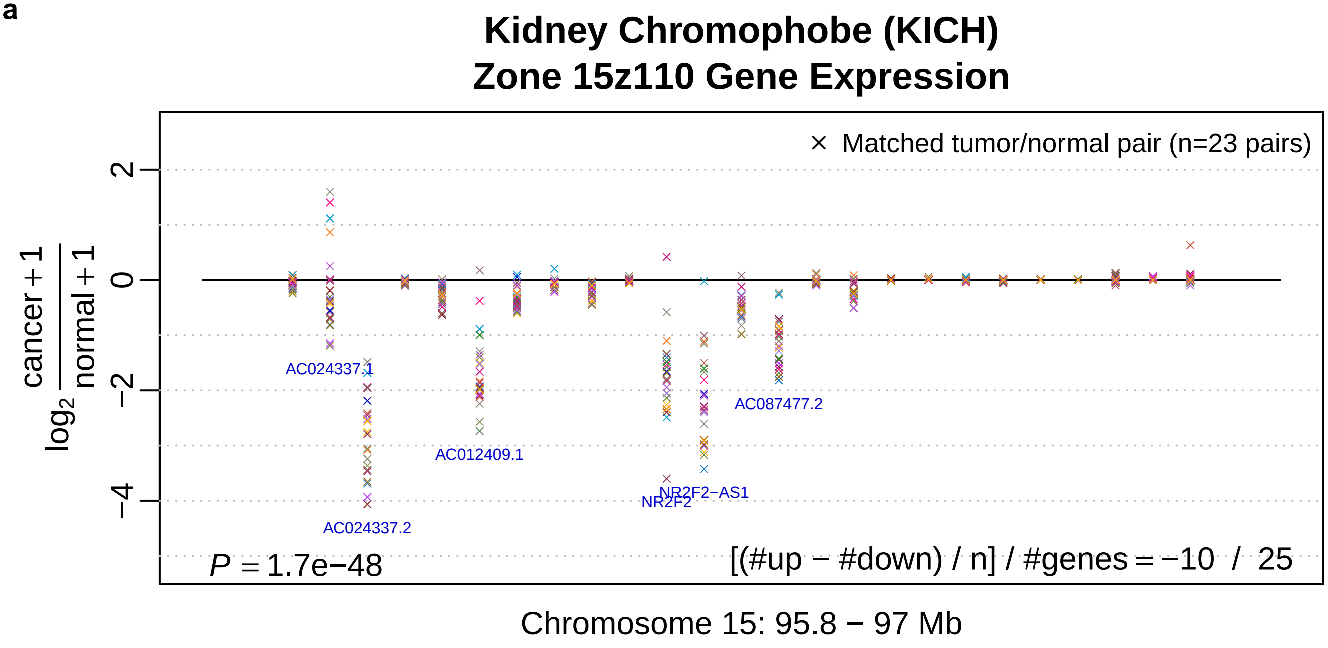

b

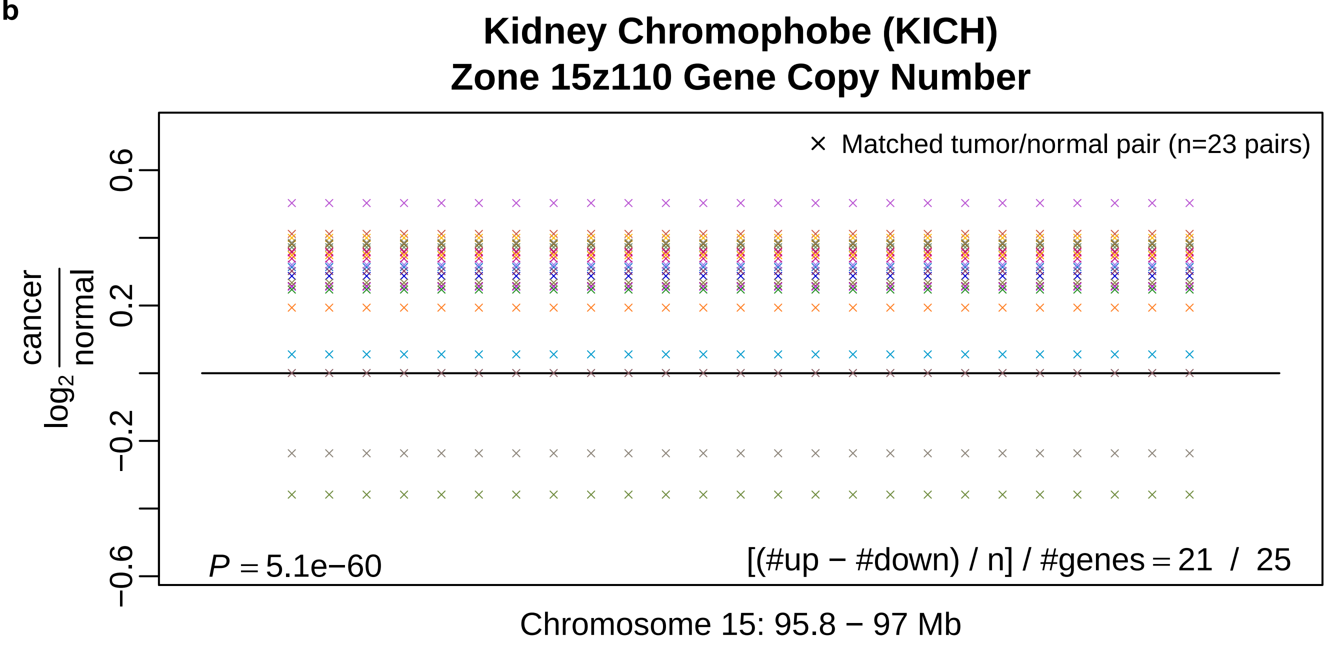

Supplementary Figure S3.41: The third most statistically significant polarized regulation zone in KICH. a, The gene expression log ratio of cancer to normal for each gene within the zone in each patient. **b**, The somatic copy number log ratio of cancer to normal for each gene within the zone in each patient. See the full legend on page 3.

a

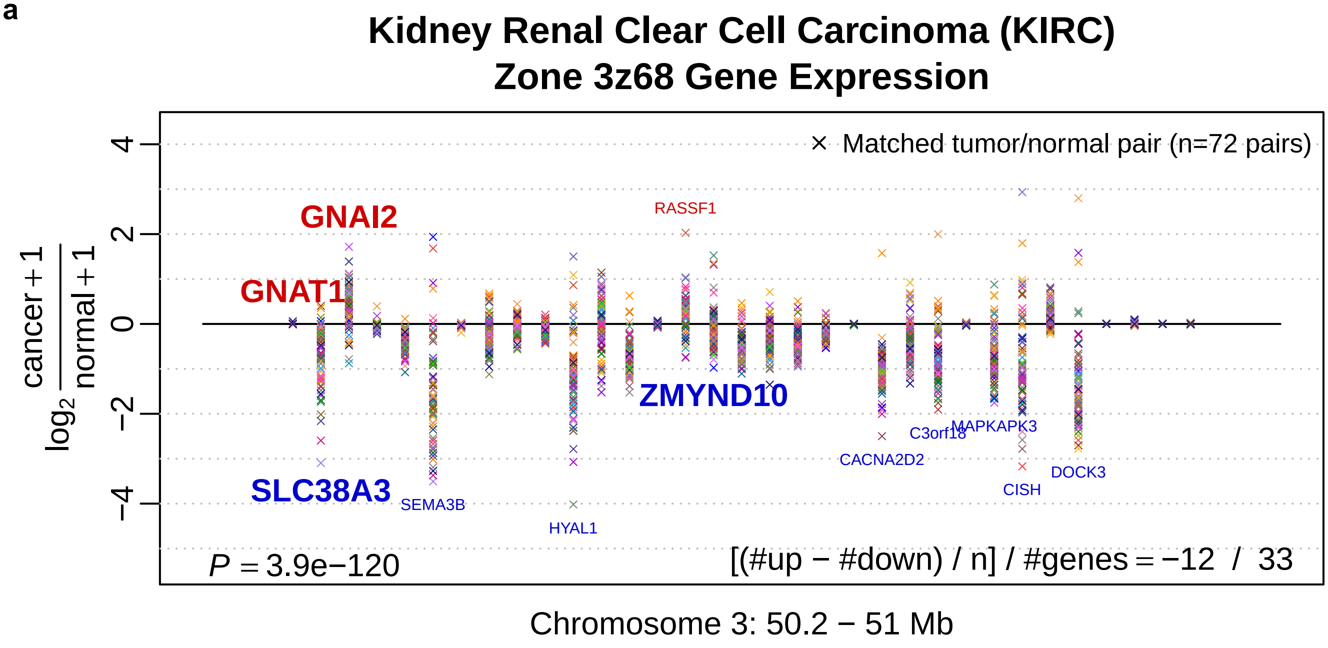

b

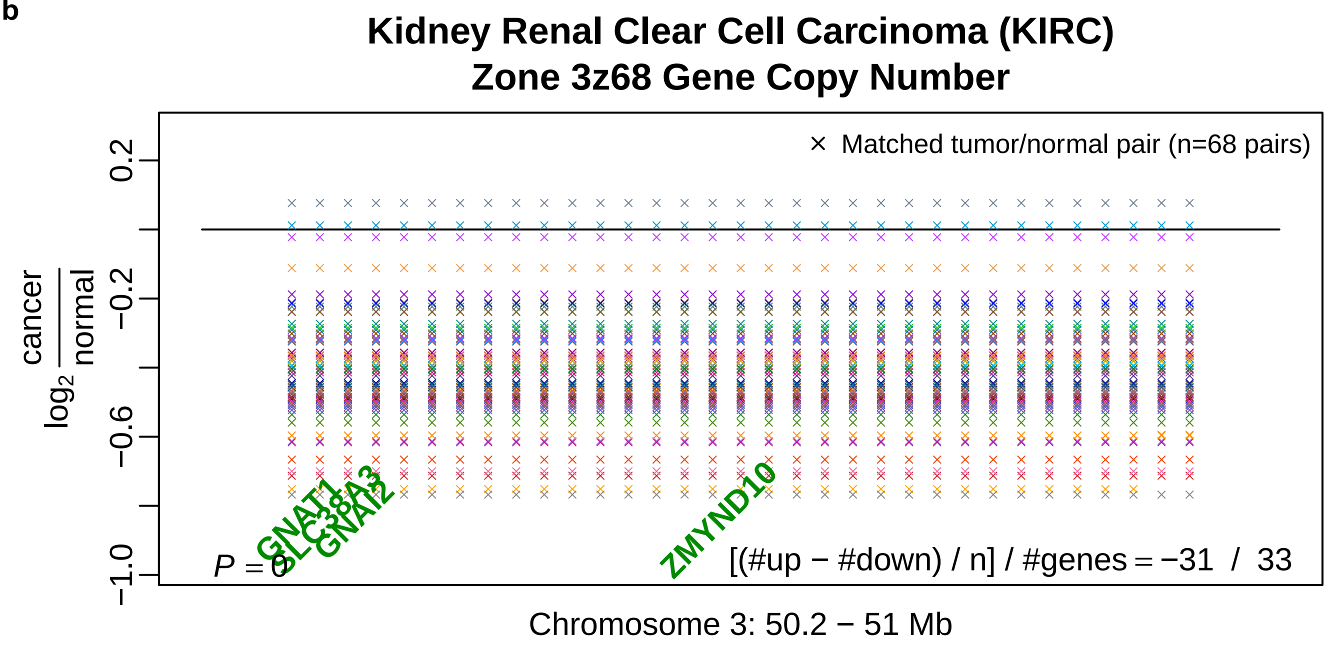

Supplementary Figure S3.42: The third most statistically significant polarized regulation zone in KIRC. a, The gene expression log ratio of cancer to normal for each gene within the zone in each patient. **b**, The somatic copy number log ratio of cancer to normal for each gene within the zone in each patient. See the full legend on page 3.

a

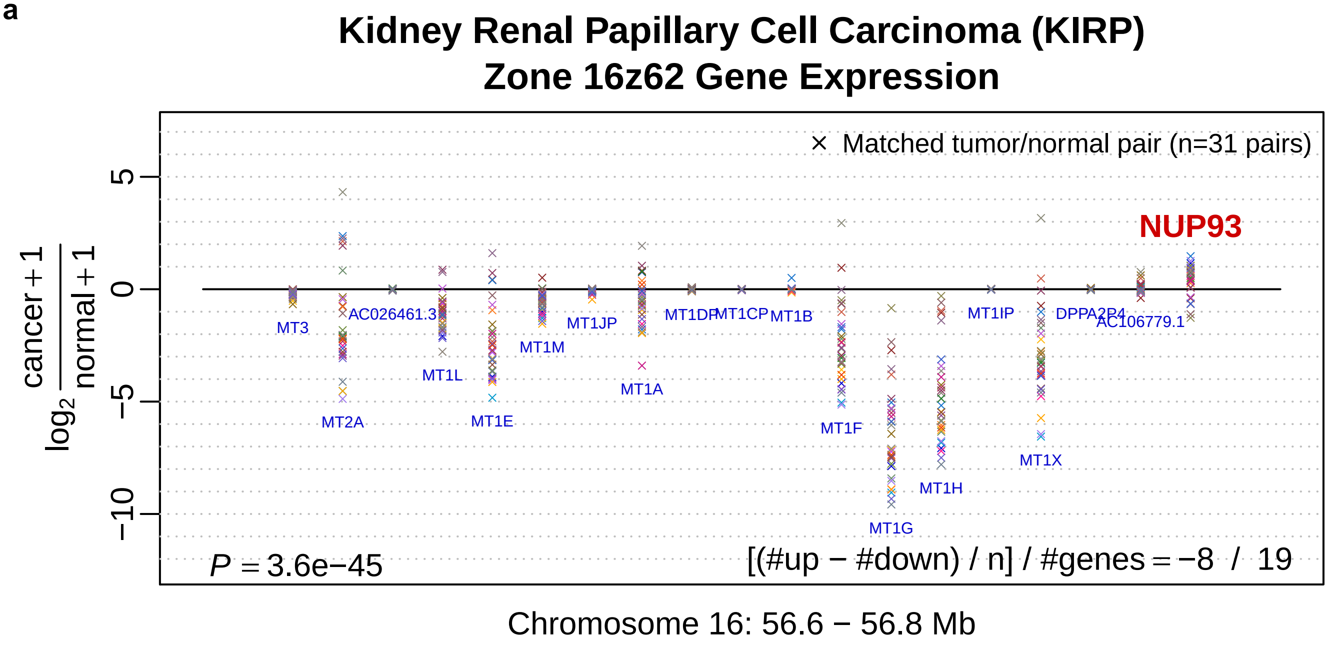

b

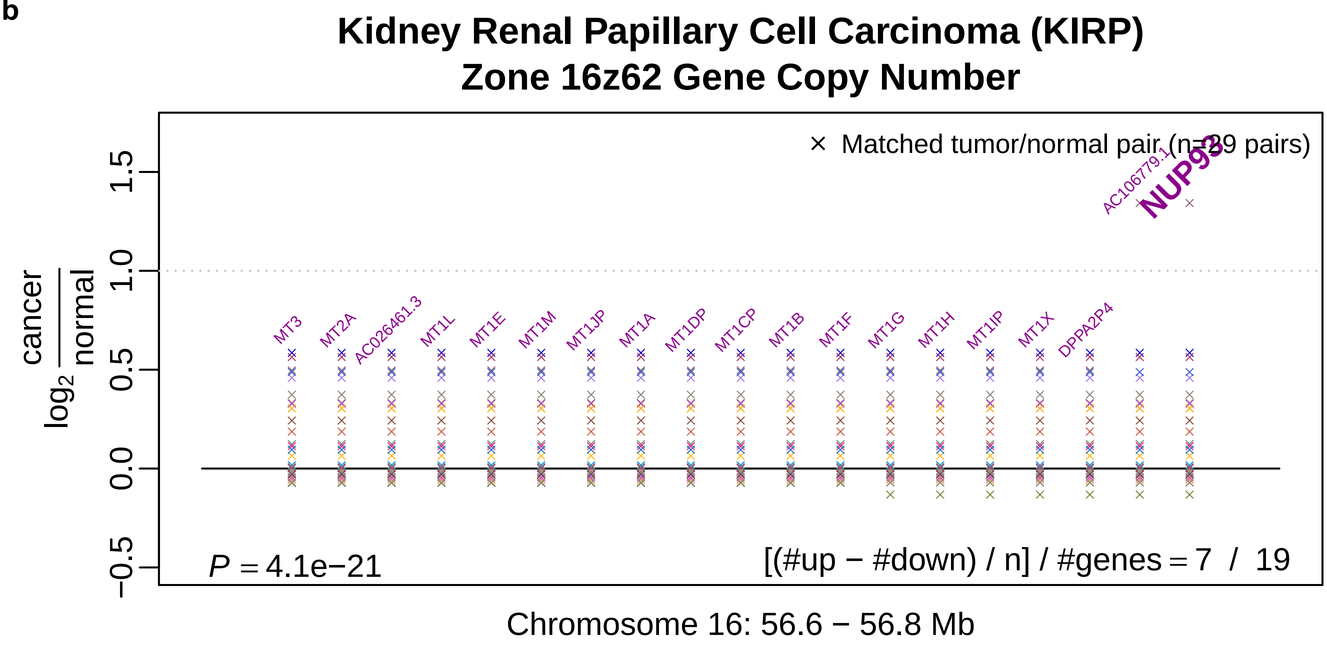

Supplementary Figure S3.43: The third most statistically significant polarized regulation zone in KIRP. a, The gene expression log ratio of cancer to normal for each gene within the zone in each patient. **b**, The somatic copy number log ratio of cancer to normal for each gene within the zone in each patient. See the full legend on page 3.

a

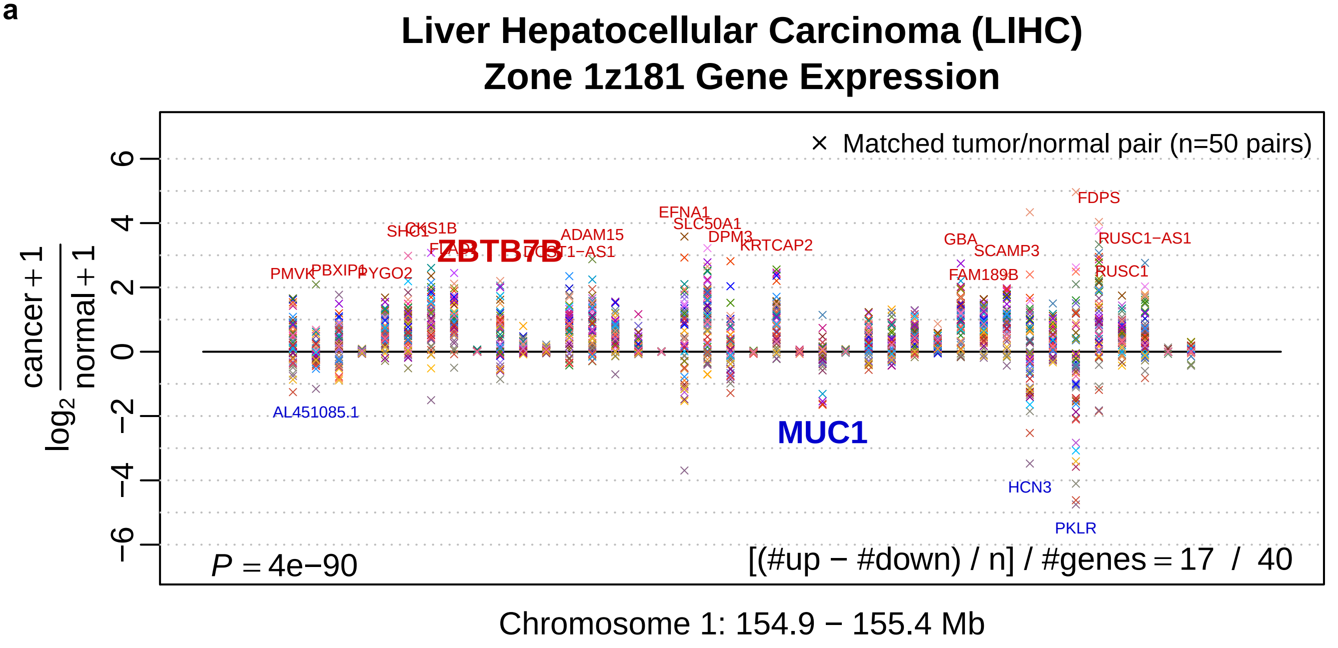

b

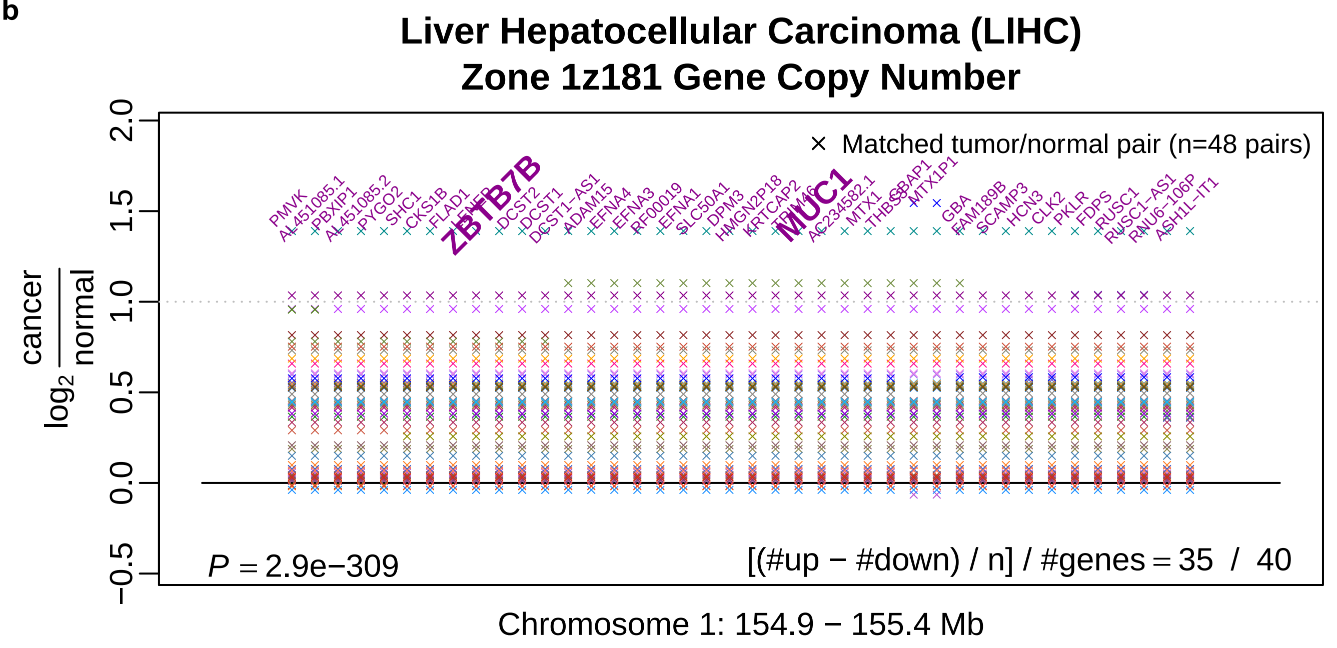

Supplementary Figure S3.44: The third most statistically significant polarized regulation zone in LIHC. a, The gene expression log ratio of cancer to normal for each gene within the zone in each patient. **b**, The somatic copy number log ratio of cancer to normal for each gene within the zone in each patient. See the full legend on page 3.

a

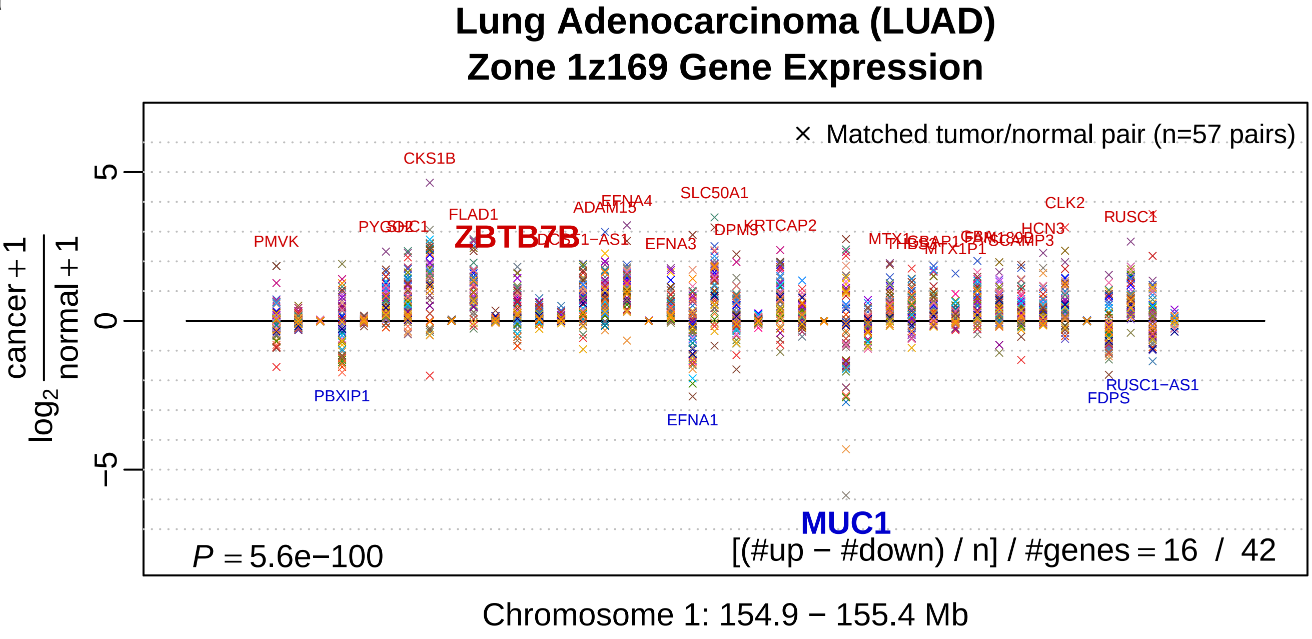

b

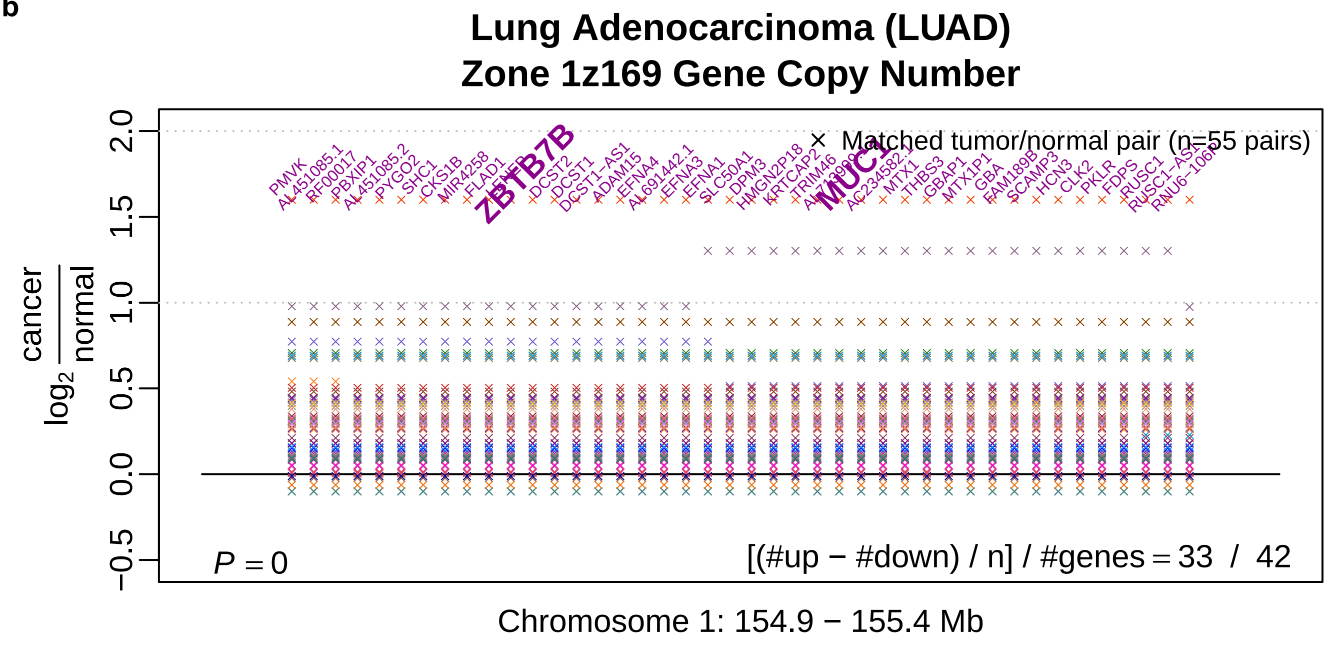

Supplementary Figure S3.45: The third most statistically significant polarized regulation zone in LUAD. a, The gene expression log ratio of cancer to normal for each gene within the zone in each patient. **b**, The somatic copy number log ratio of cancer to normal for each gene within the zone in each patient. See the full legend on page 3.

a

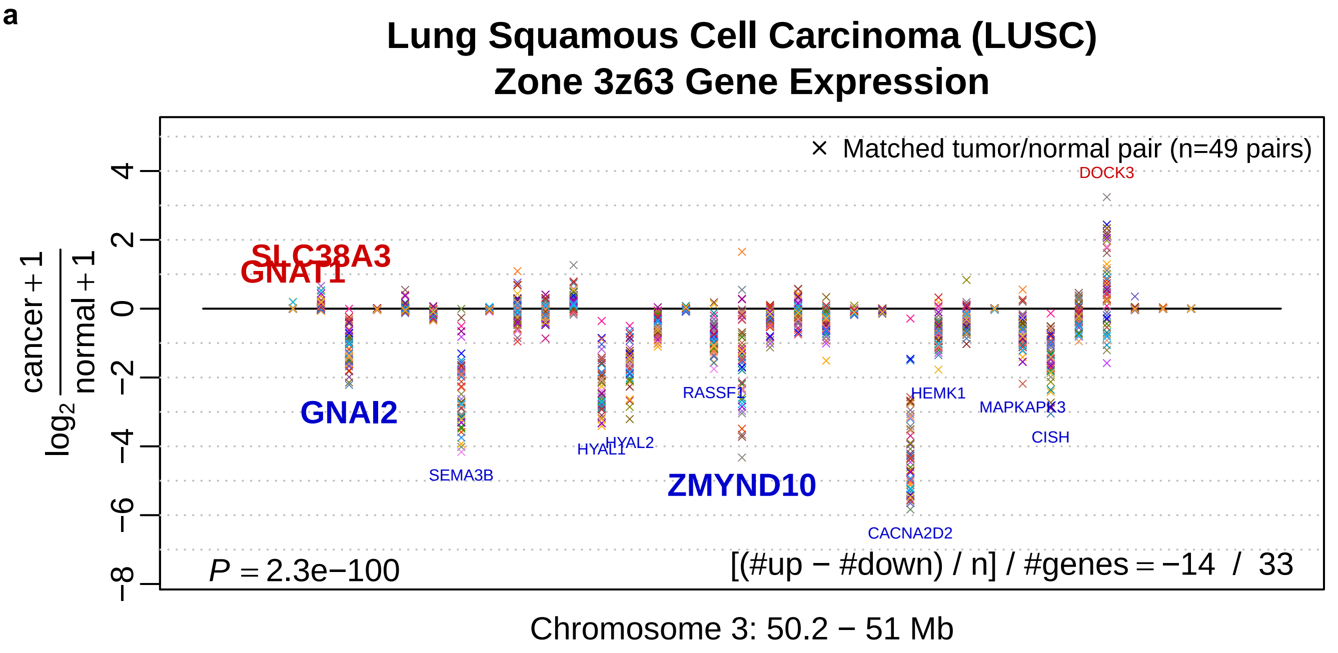

b

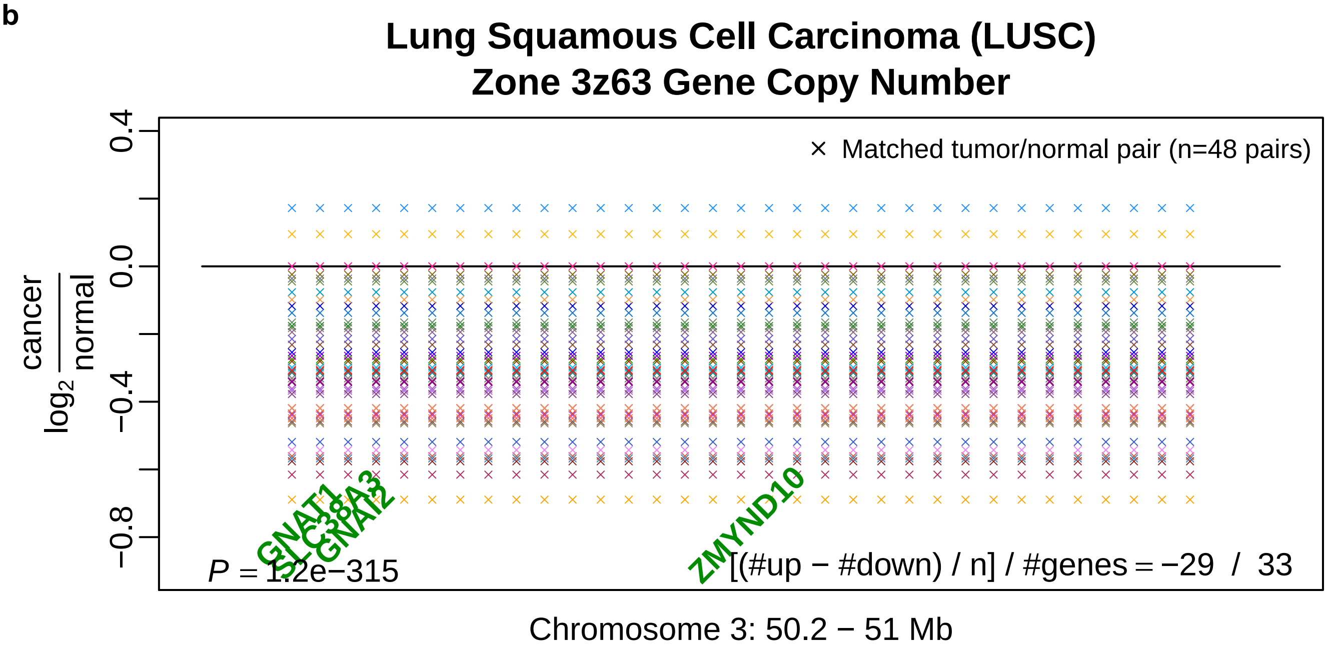

Supplementary Figure S3.46: The third most statistically significant polarized regulation zone in LUSC. a, The gene expression log ratio of cancer to normal for each gene within the zone in each patient. **b**, The somatic copy number log ratio of cancer to normal for each gene within the zone in each patient. See the full legend on page 3.

a

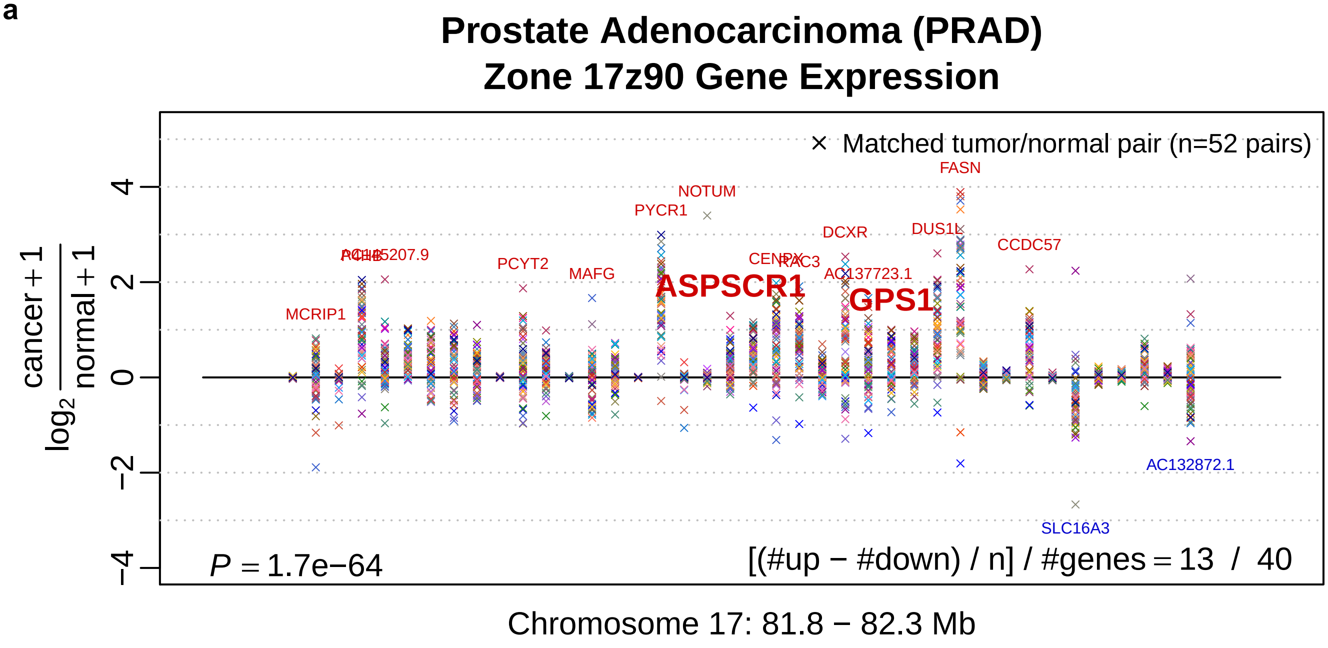

b

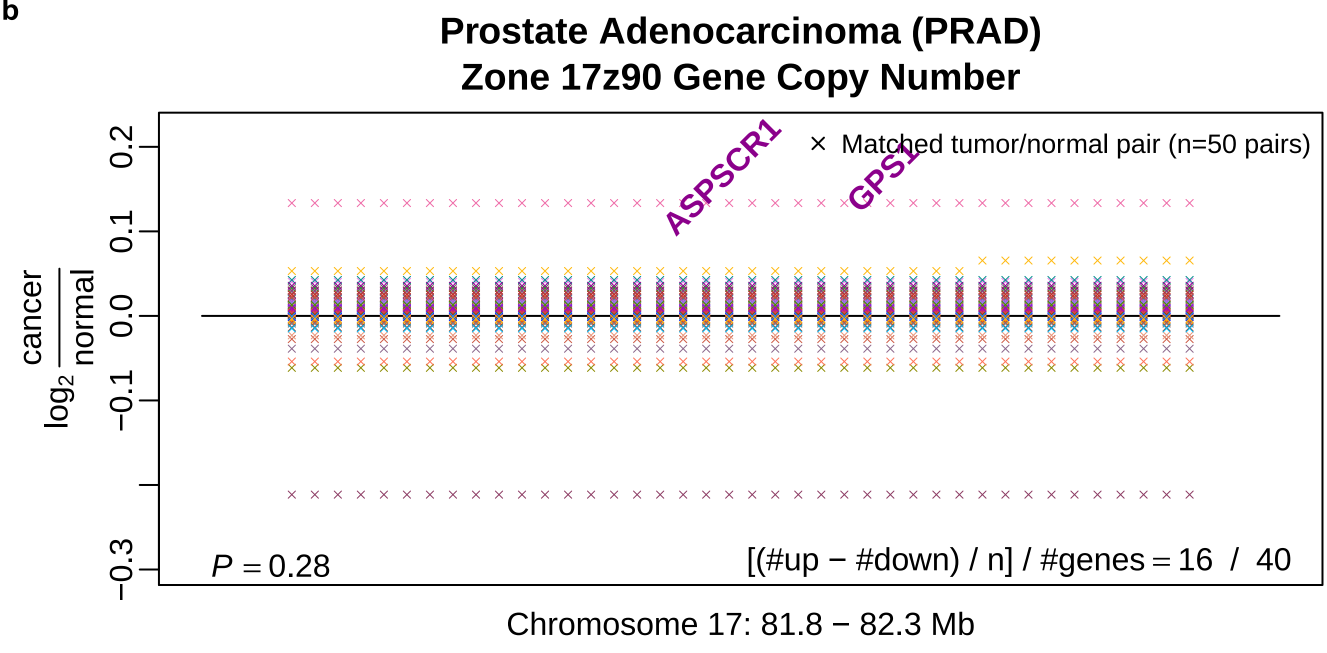

Supplementary Figure S3.47: The third most statistically significant polarized regulation zone in PRAD. a, The gene expression log ratio of cancer to normal for each gene within the zone in each patient. **b**, The somatic copy number log ratio of cancer to normal for each gene within the zone in each patient. See the full legend on page 3.

a

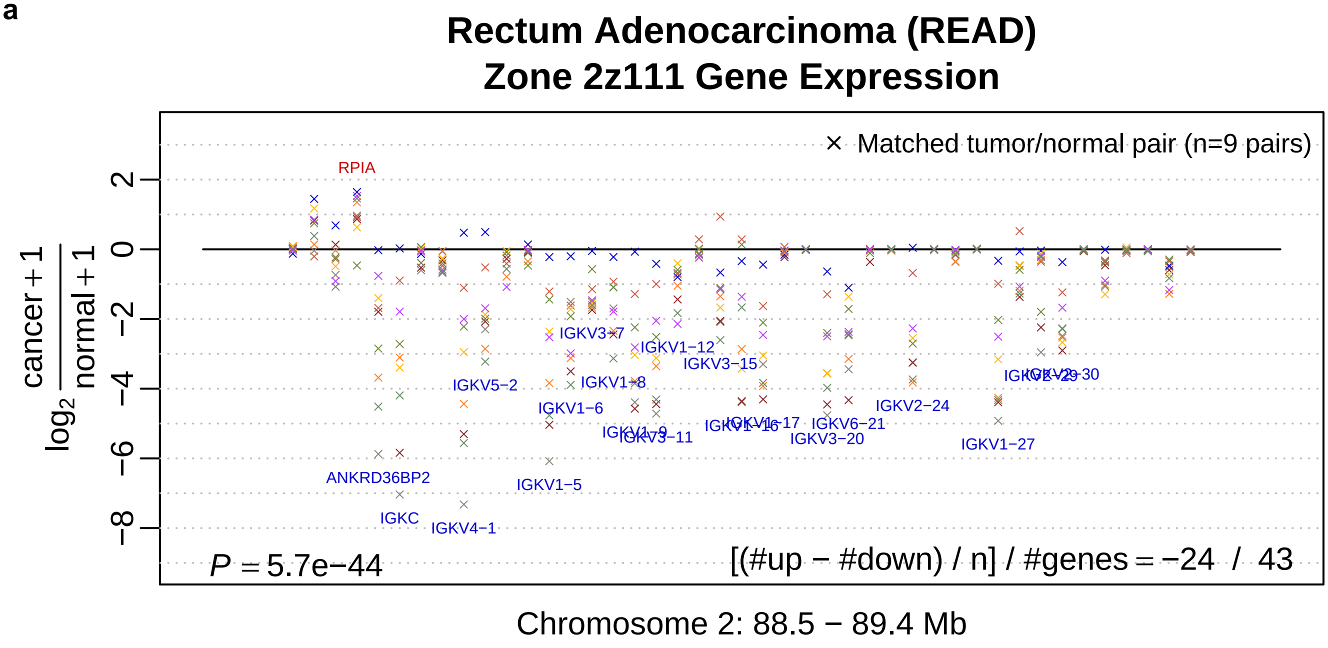

b

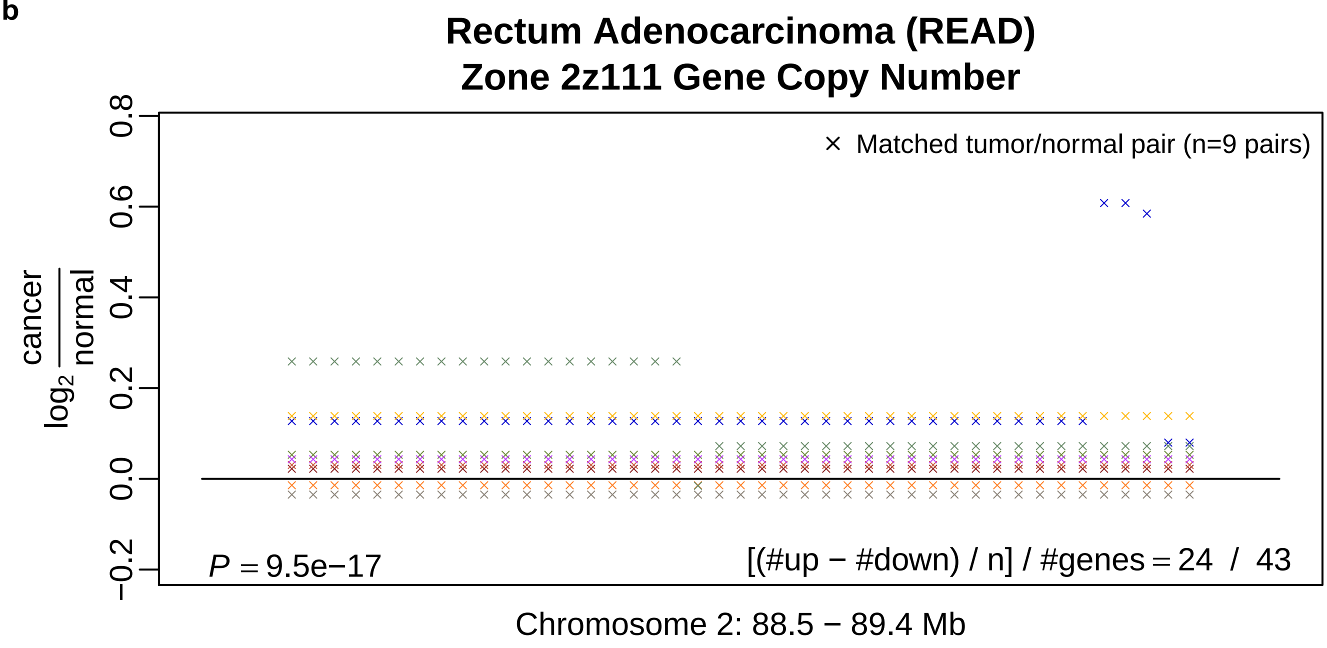

Supplementary Figure S3.48: The third most statistically significant polarized regulation zone in READ. a, The gene expression log ratio of cancer to normal for each gene within the zone in each patient. **b**, The somatic copy number log ratio of cancer to normal for each gene within the zone in each patient. See the full legend on page 3.

a

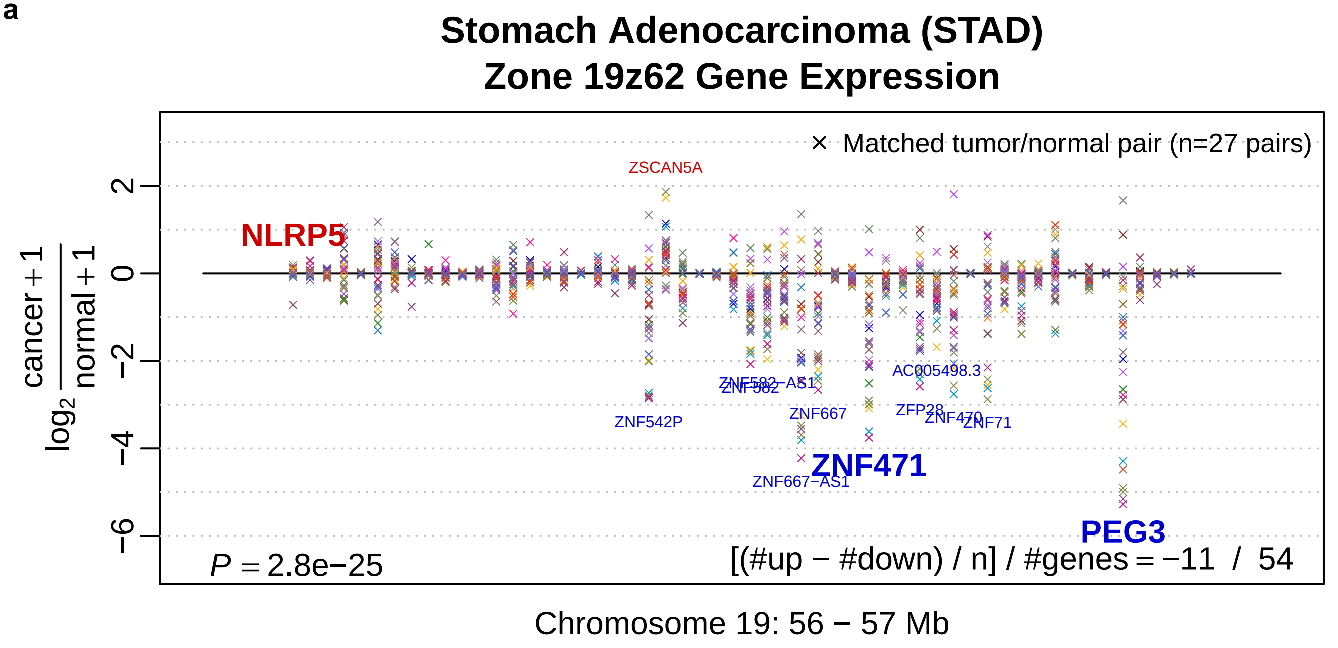

b

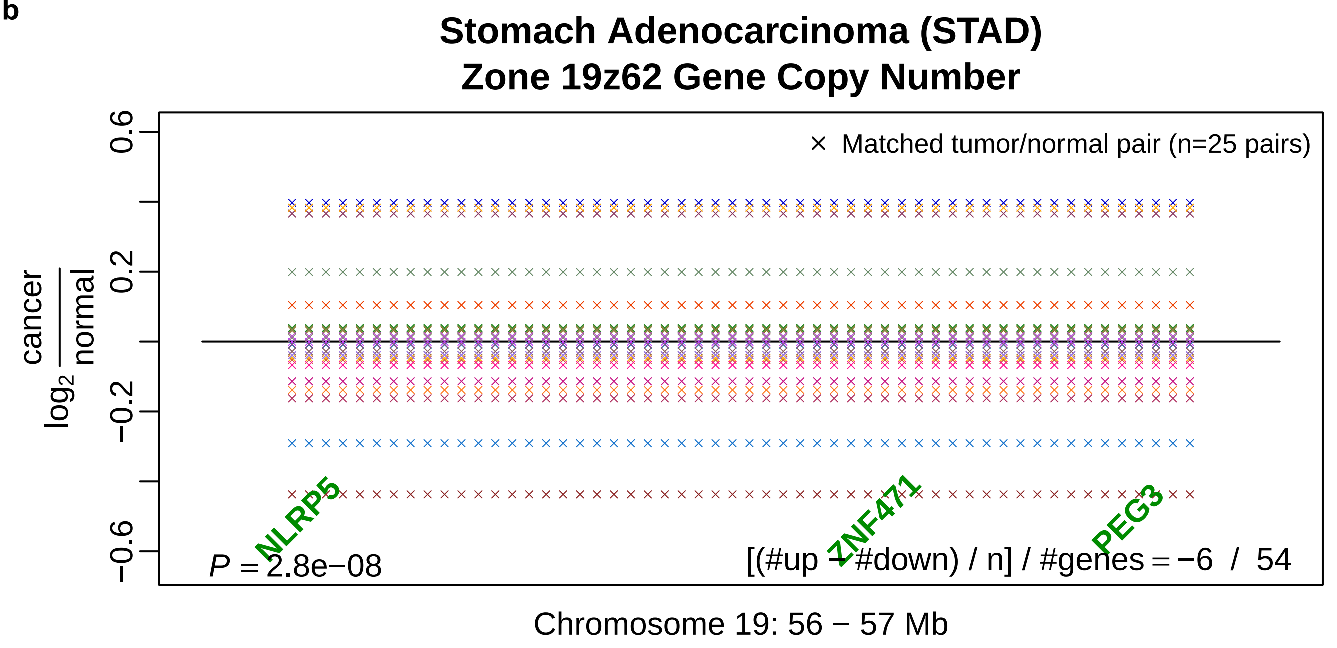

Supplementary Figure S3.49: The third most statistically significant polarized regulation zone in STAD. a, The gene expression log ratio of cancer to normal for each gene within the zone in each patient. **b**, The somatic copy number log ratio of cancer to normal for each gene within the zone in each patient. See the full legend on page 3.

a

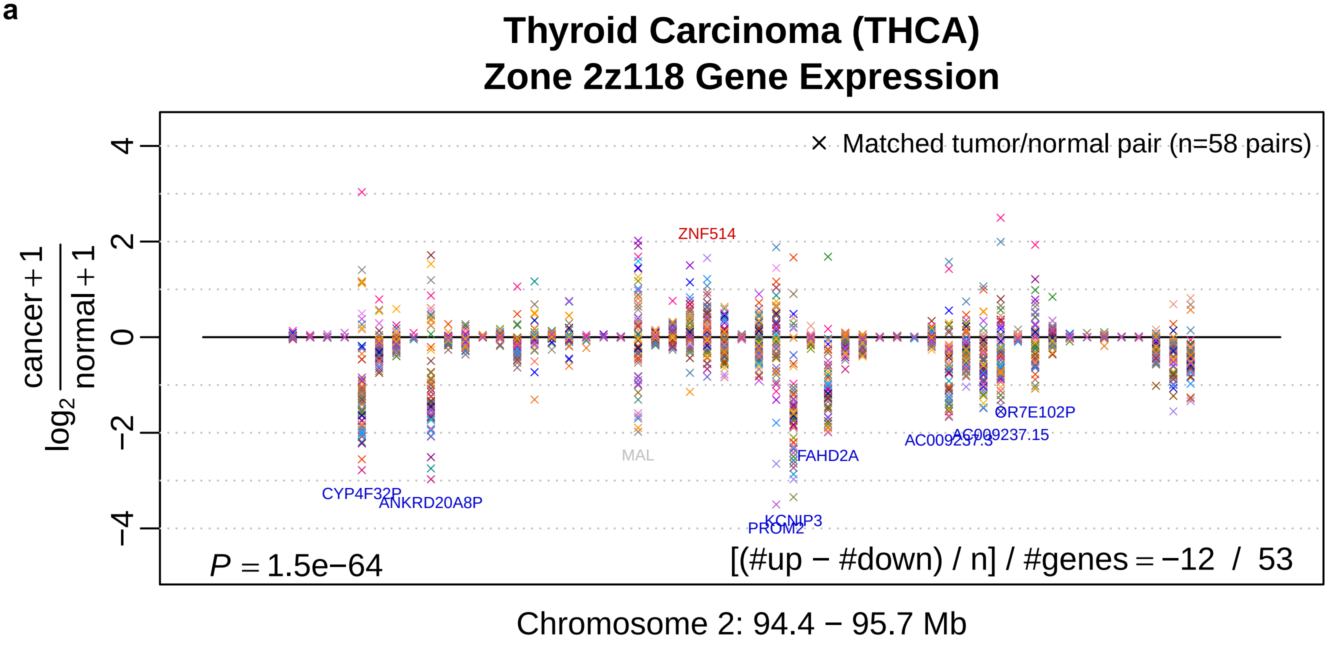

b

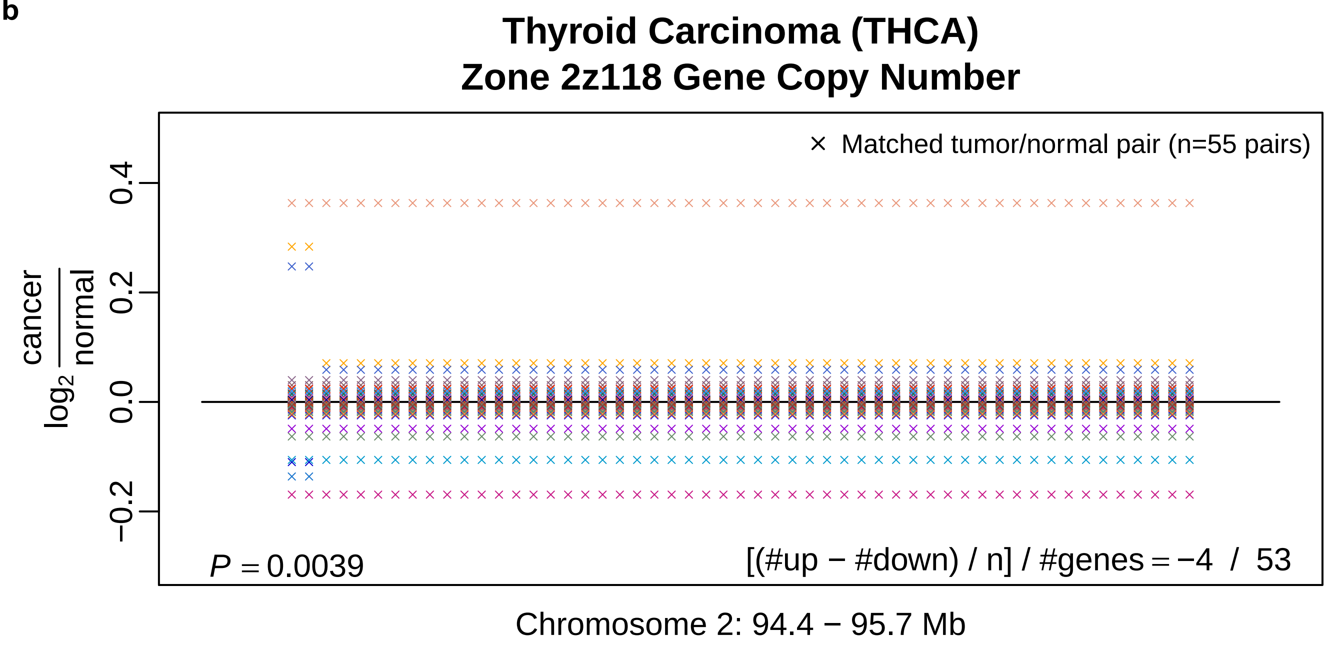

Supplementary Figure S3.50: The third most statistically significant polarized regulation zone in THCA. a, The gene expression log ratio of cancer to normal for each gene within the zone in each patient. **b**, The somatic copy number log ratio of cancer to normal for each gene within the zone in each patient. See the full legend on page 3.

a

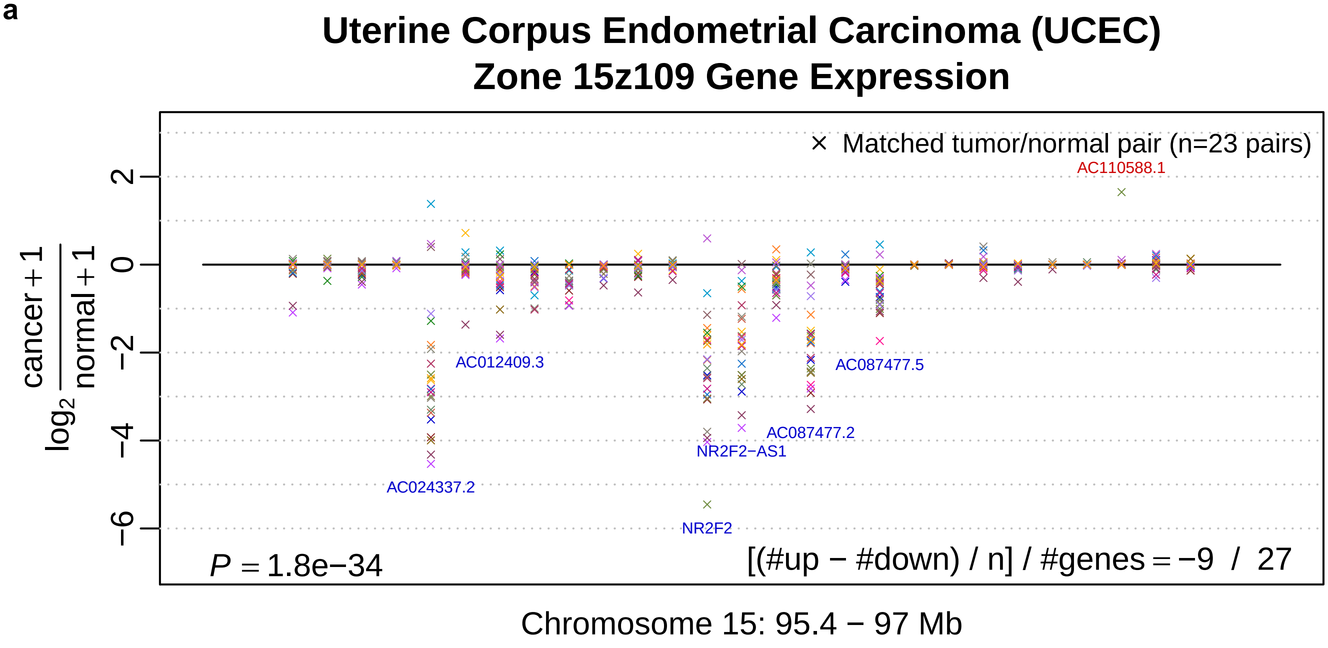

b

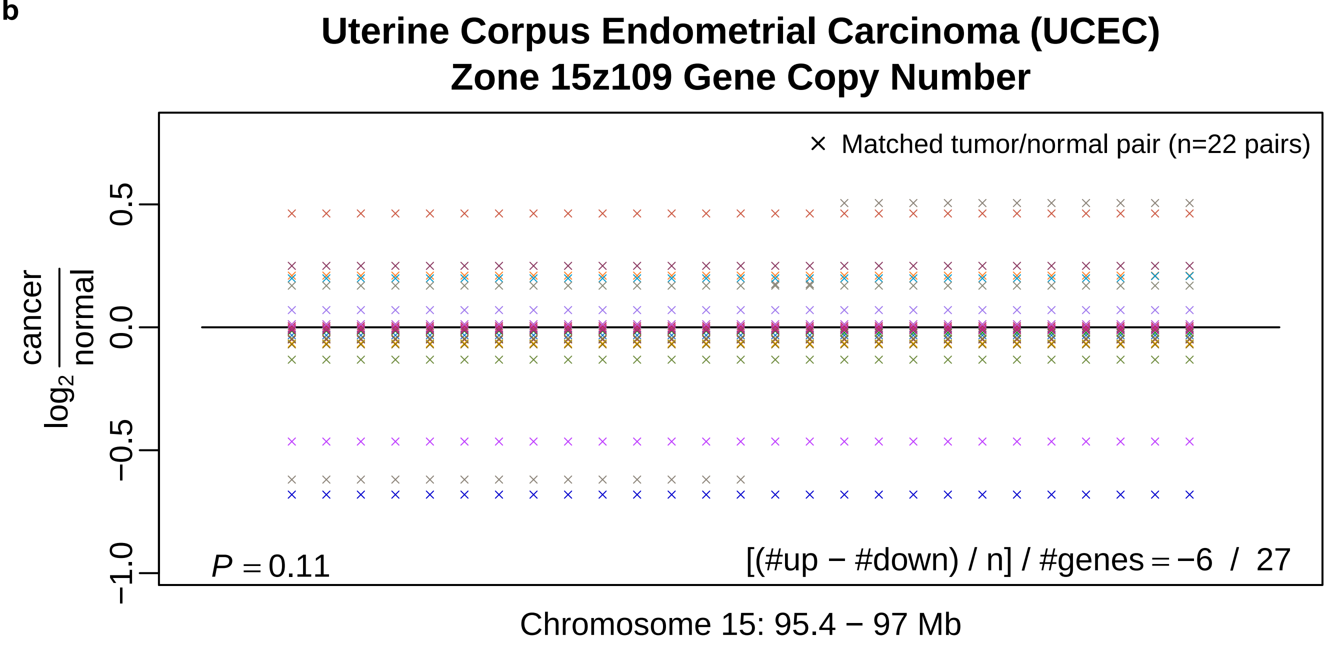

Supplementary Figure S3.51: The third most statistically significant polarized regulation zone in UCEC. a, The gene expression log ratio of cancer to normal for each gene within the zone in each patient. **b**, The somatic copy number log ratio of cancer to normal for each gene within the zone in each patient. See the full legend on page 3.

3 Fourth most polarized regulation zones of 17 cancer types

a

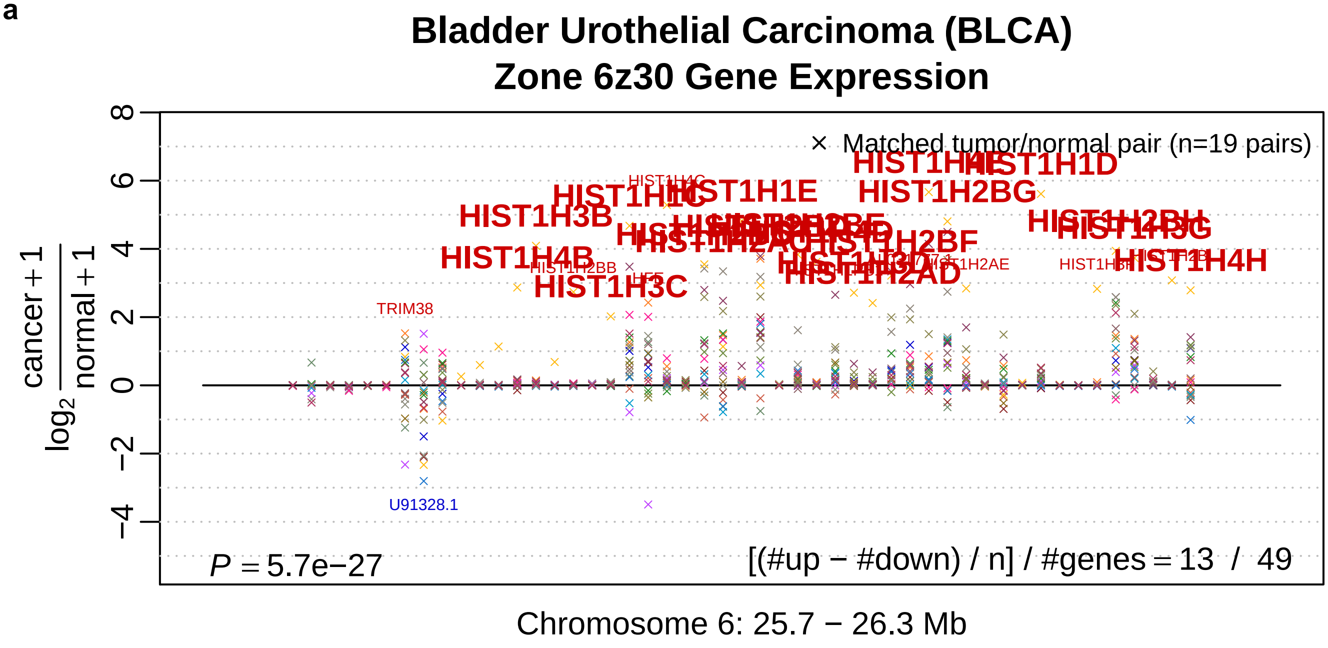

b

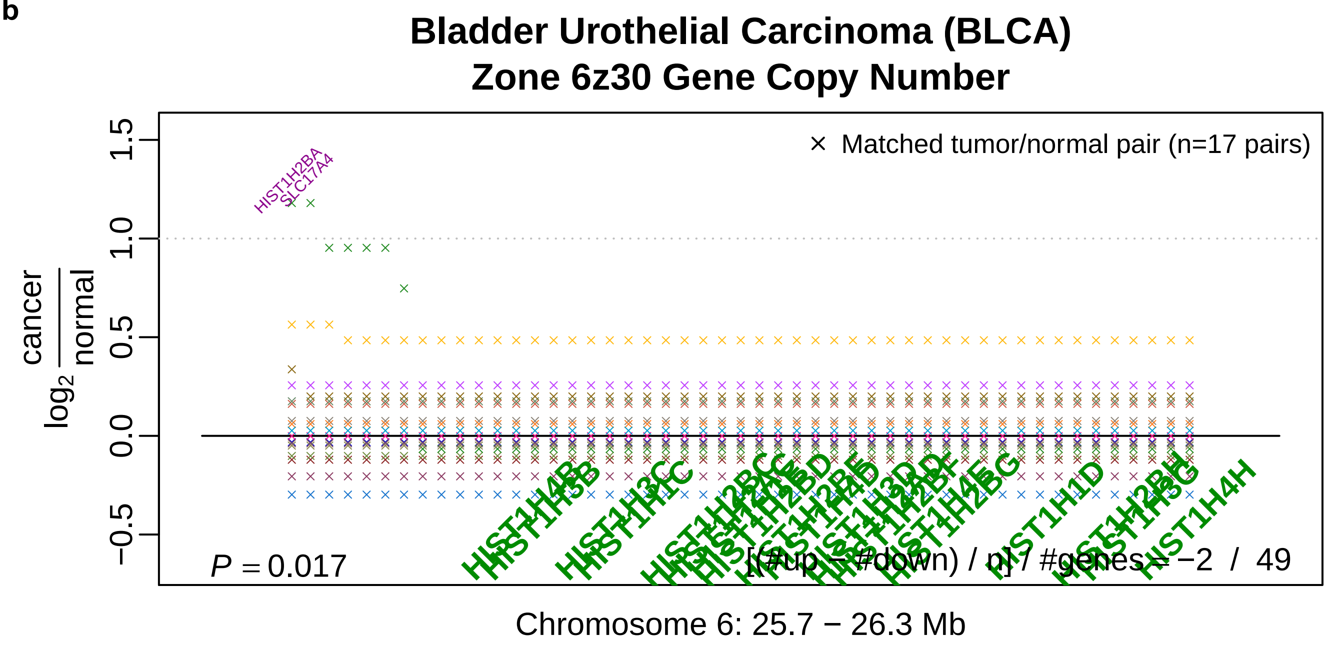

Supplementary Figure S3.52: The fourth most statistically significant polarized regulation zone in BLCA. a, The gene expression log ratio of cancer to normal for each gene within the zone in each patient. **b**, The somatic copy number log ratio of cancer to normal for each gene within the zone in each patient. See the full legend on page 3.

a

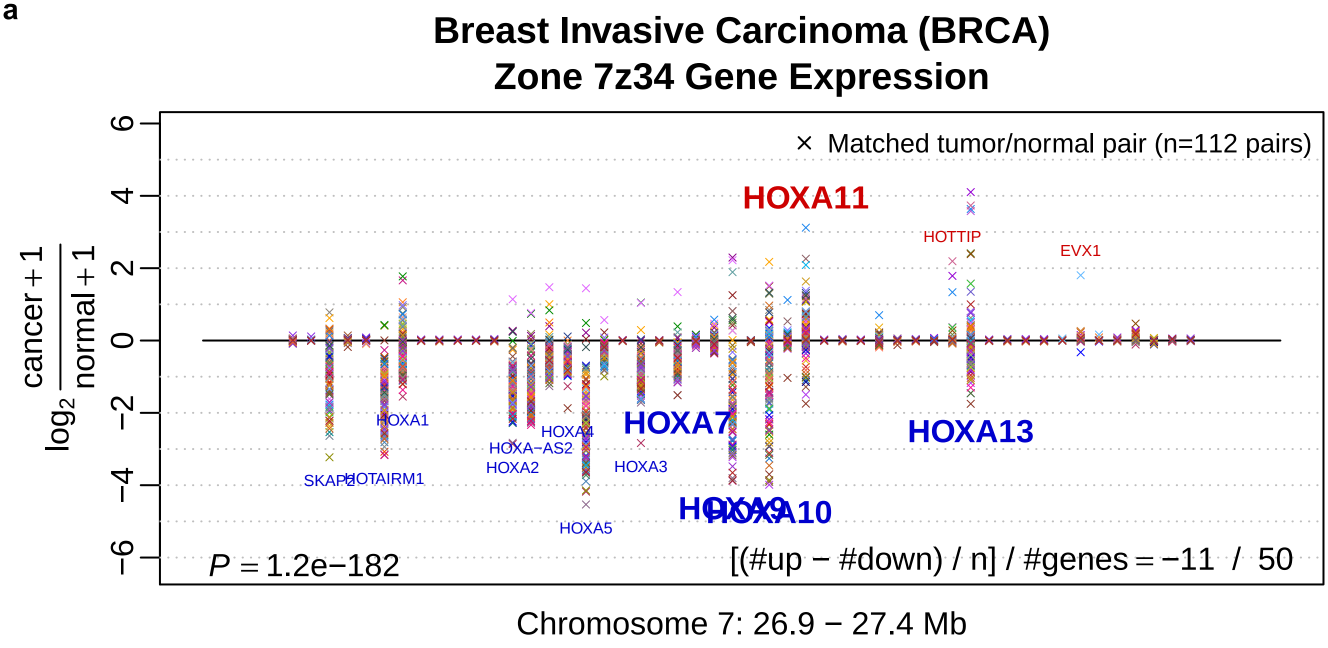

b

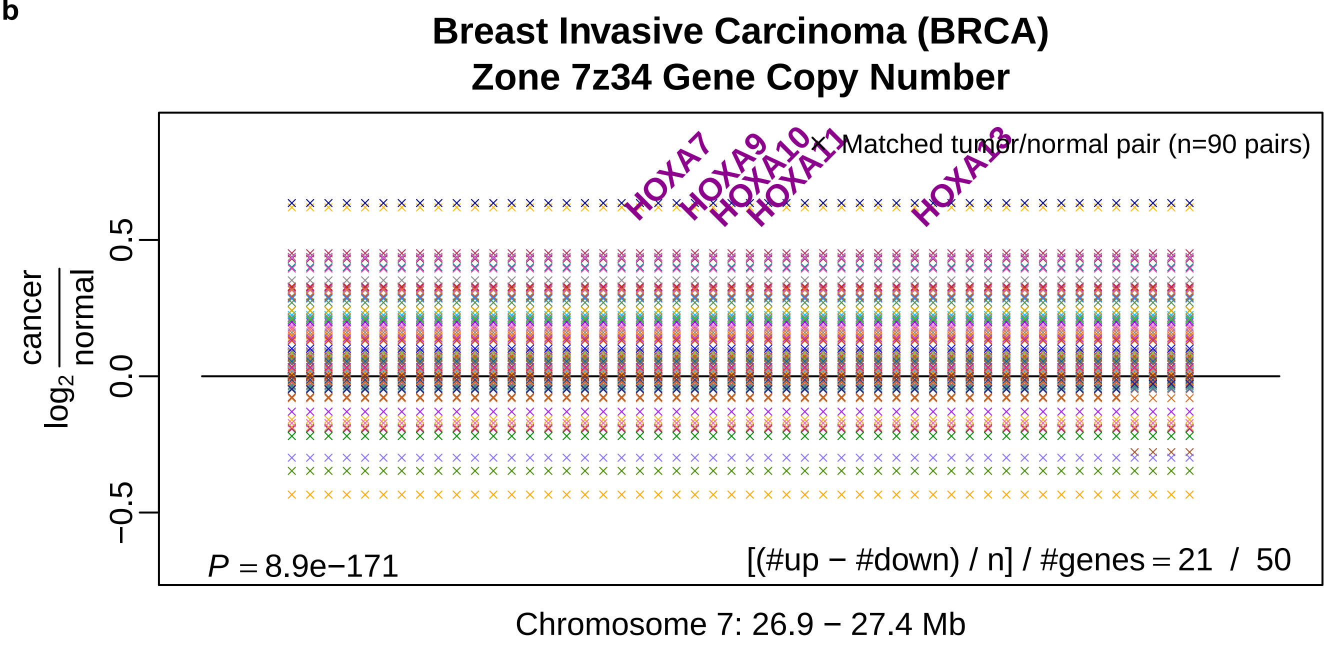

Supplementary Figure S3.53: The fourth most statistically significant polarized regulation zone in BRCA. a, The gene expression log ratio of cancer to normal for each gene within the zone in each patient. **b**, The somatic copy number log ratio of cancer to normal for each gene within the zone in each patient. See the full legend on page 3.

a

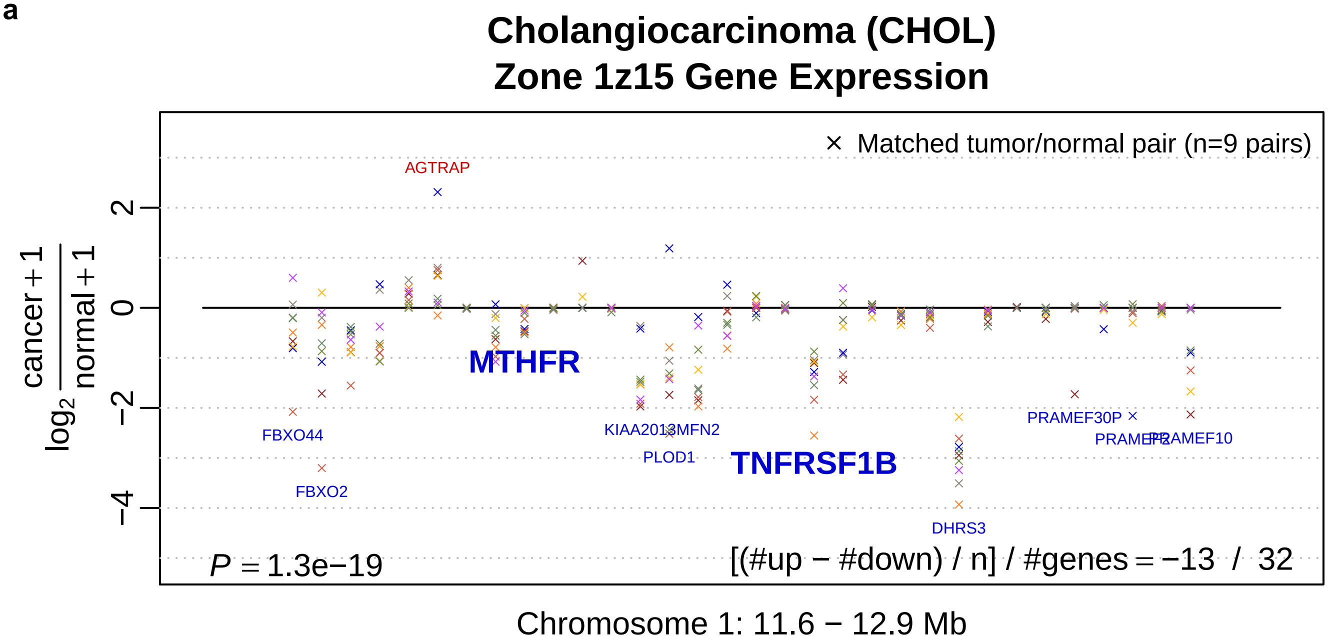

b

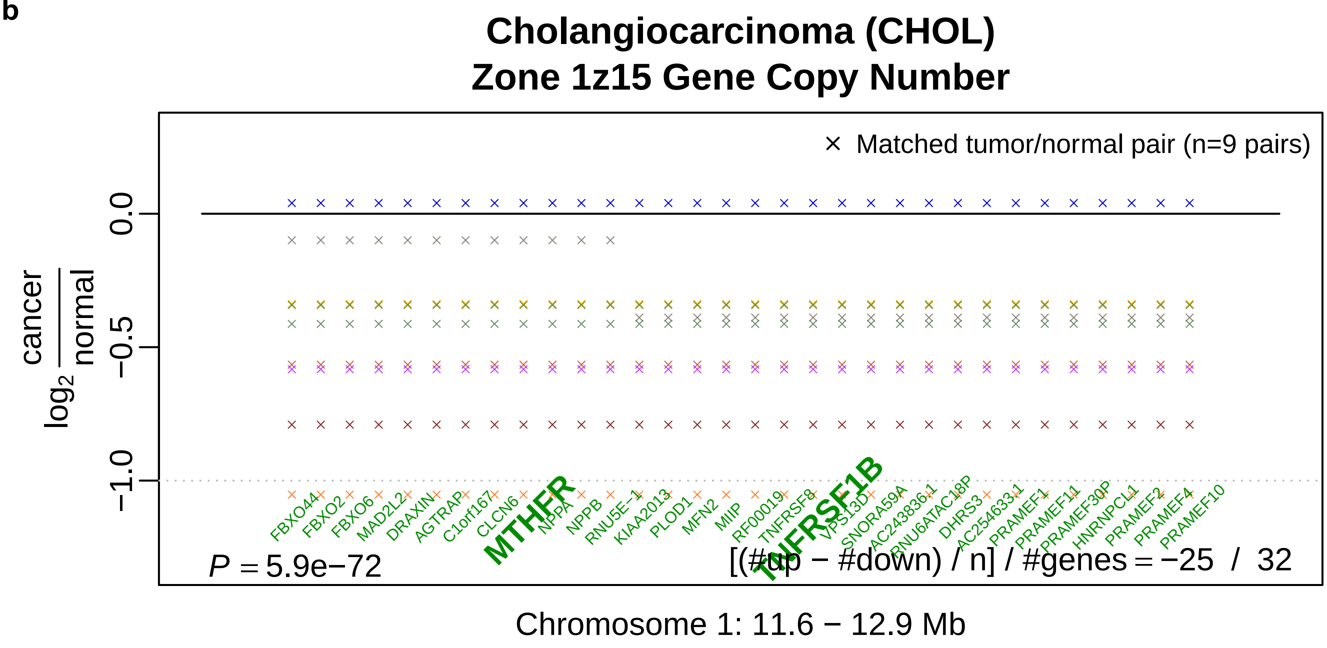

Supplementary Figure S3.54: The fourth most statistically significant polarized regulation zone in CHOL. a, The gene expression log ratio of cancer to normal for each gene within the zone in each patient. **b**, The somatic copy number log ratio of cancer to normal for each gene within the zone in each patient. See the full legend on page 3.

a

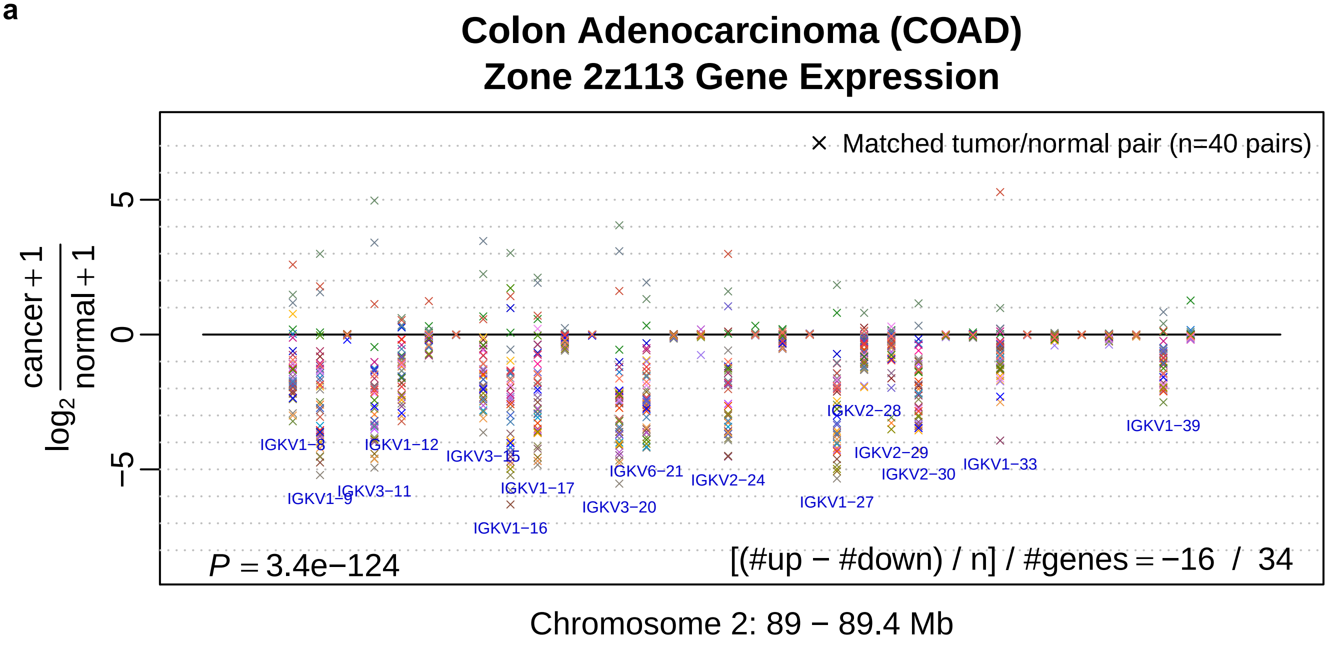

b

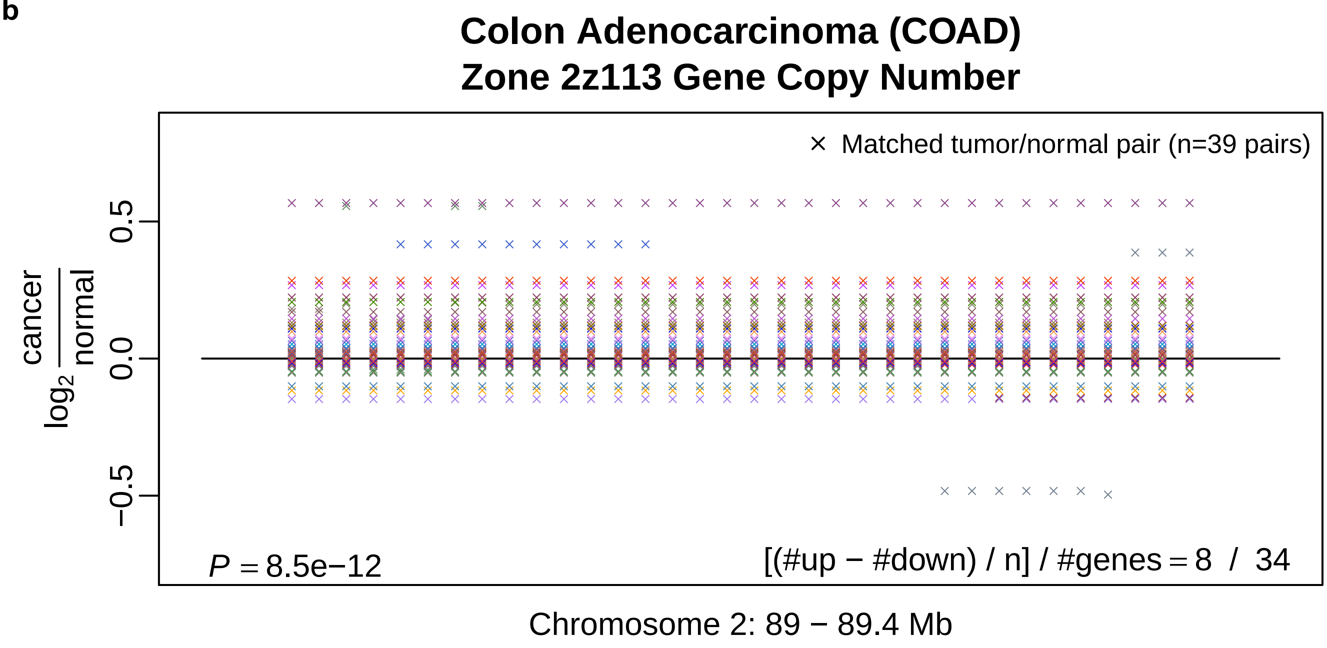

Supplementary Figure S3.55: The fourth most statistically significant polarized regulation zone in COAD. a, The gene expression log ratio of cancer to normal for each gene within the zone in each patient. **b**, The somatic copy number log ratio of cancer to normal for each gene within the zone in each patient. See the full legend on page 3.

a

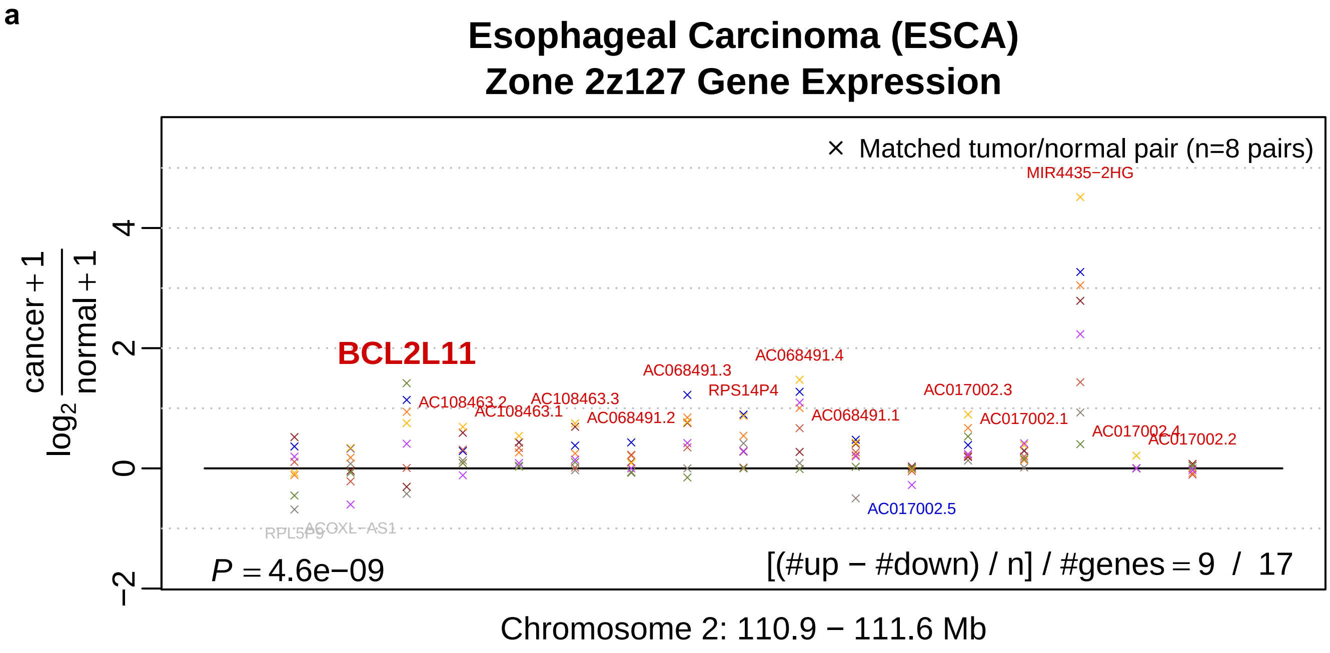

b

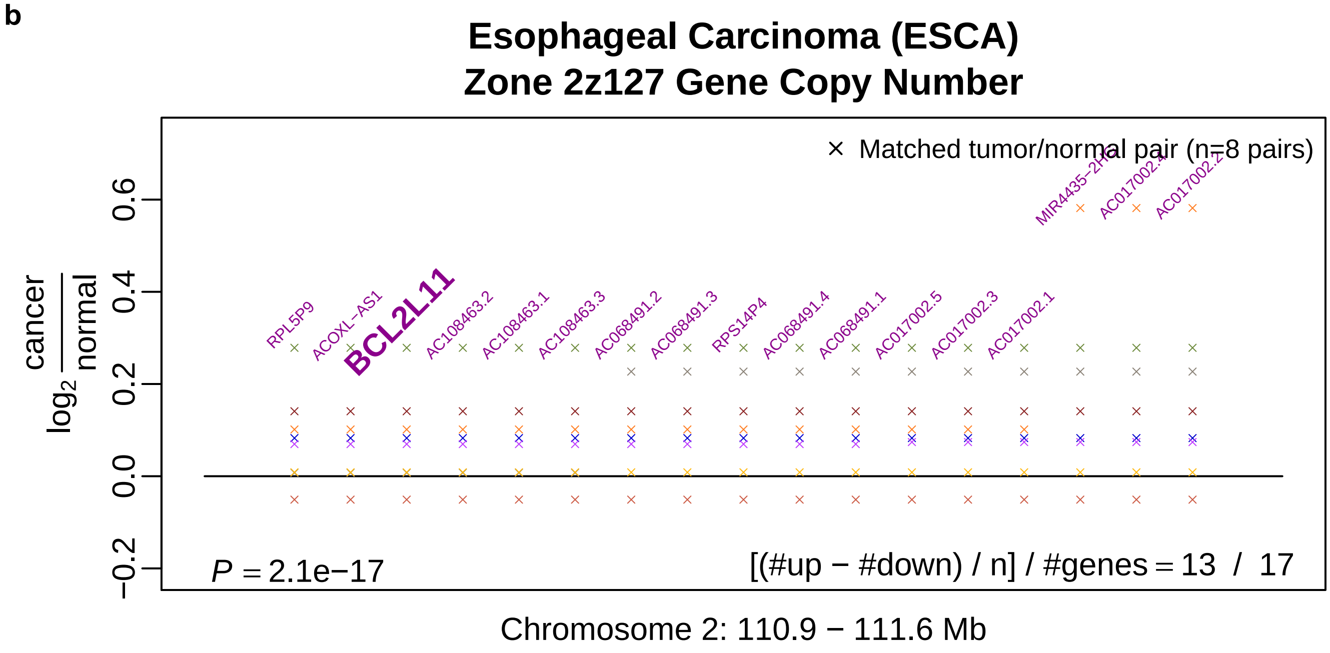

Supplementary Figure S3.56: The fourth most statistically significant polarized regulation zone in ESCA. a, The gene expression log ratio of cancer to normal for each gene within the zone in each patient. **b**, The somatic copy number log ratio of cancer to normal for each gene within the zone in each patient. See the full legend on page 3.

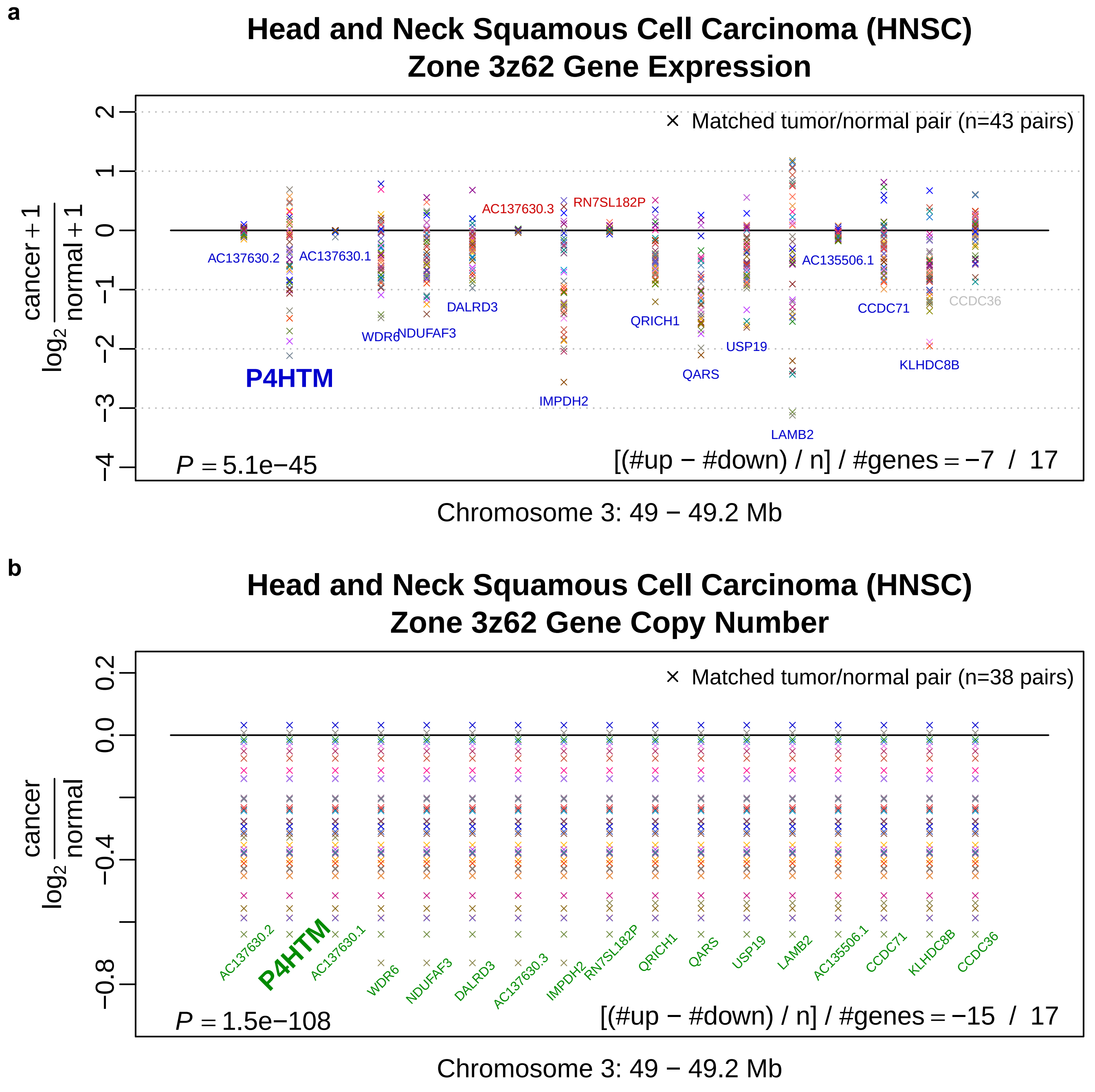

Supplementary Figure S3.57: The fourth most statistically significant polarized regulation zone in HNSC. a, The gene expression log ratio of cancer to normal for each gene within the zone in each patient. **b**, The somatic copy number log ratio of cancer to normal for each gene within the zone in each patient. See the full legend on page 3.

a

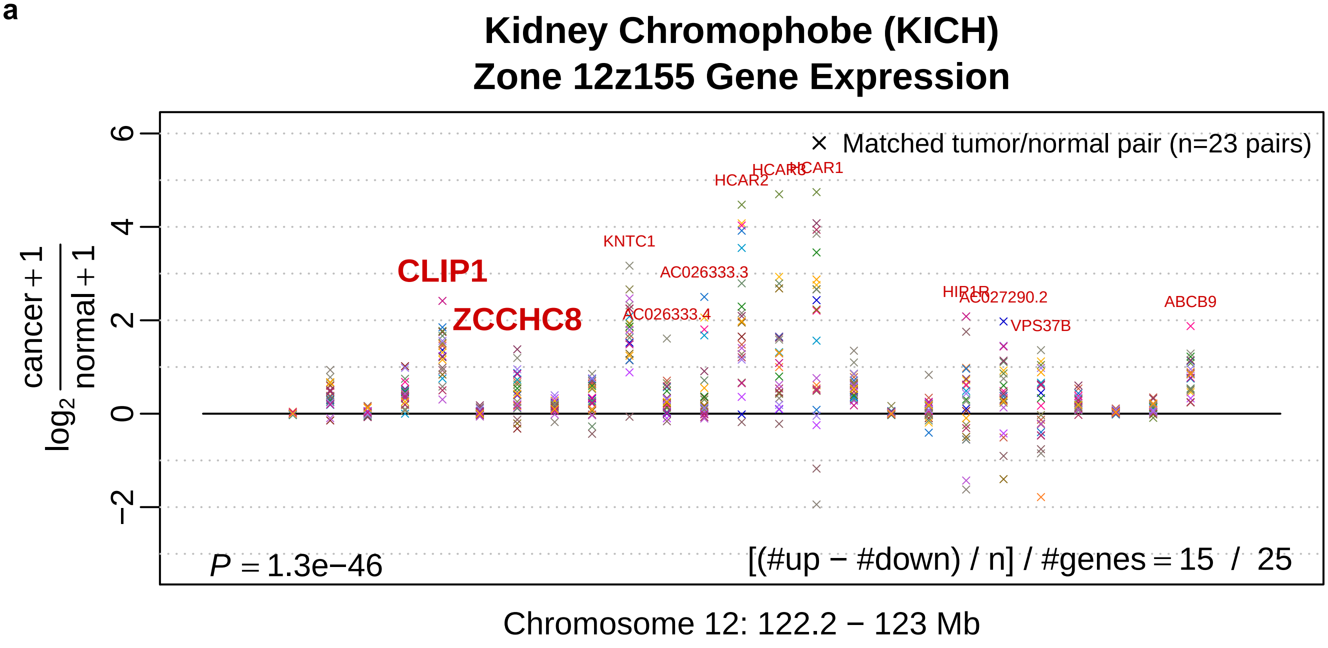

b

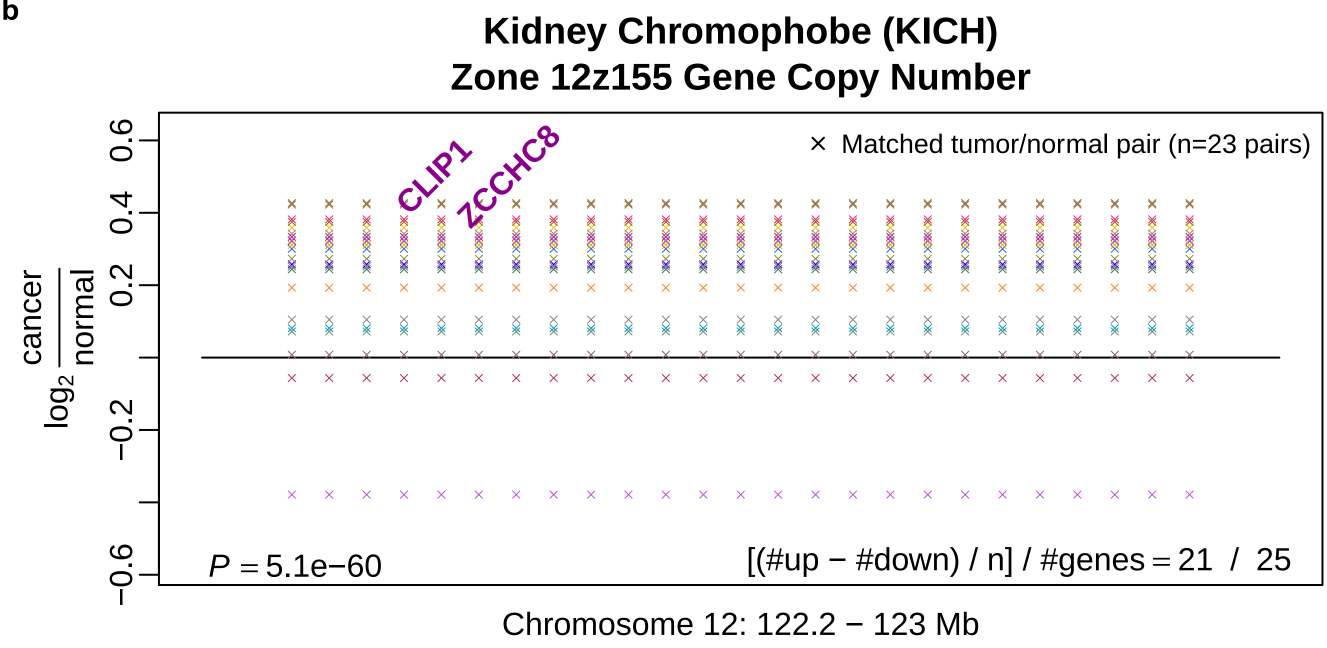

Supplementary Figure S3.58: The fourth most statistically significant polarized regulation zone in KICH. a, The gene expression log ratio of cancer to normal for each gene within the zone in each patient. **b**, The somatic copy number log ratio of cancer to normal for each gene within the zone in each patient. See the full legend on page 3.

a

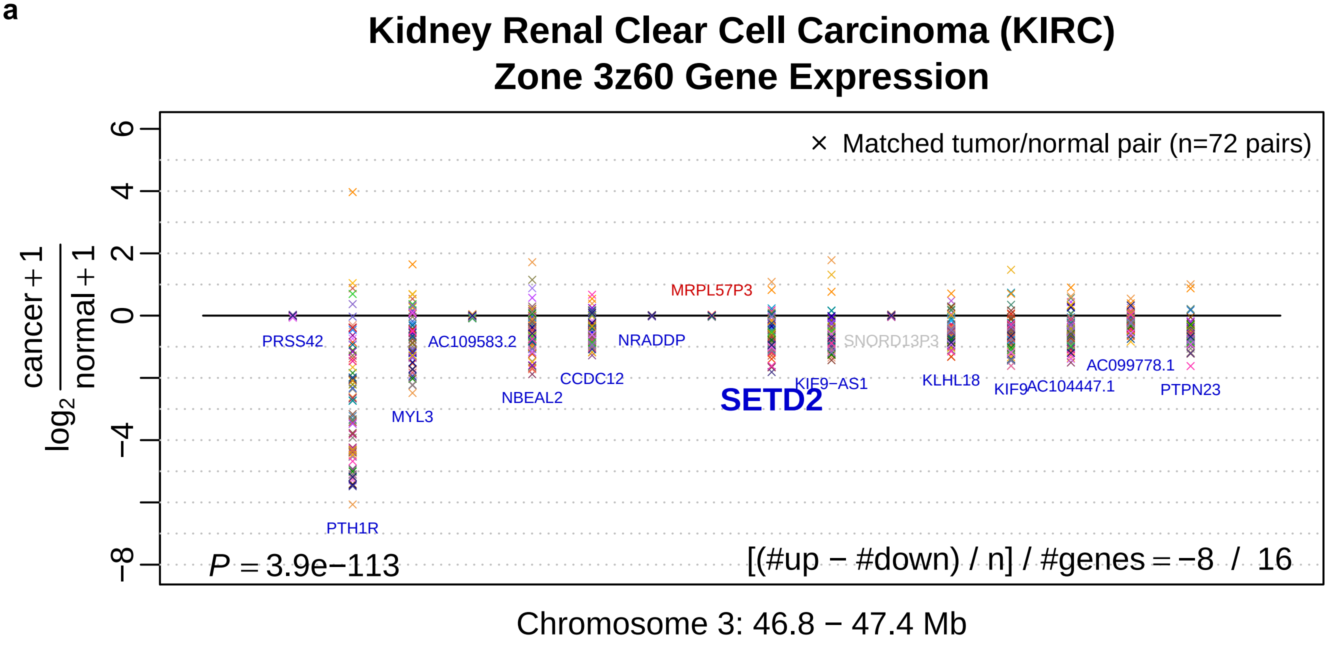

b

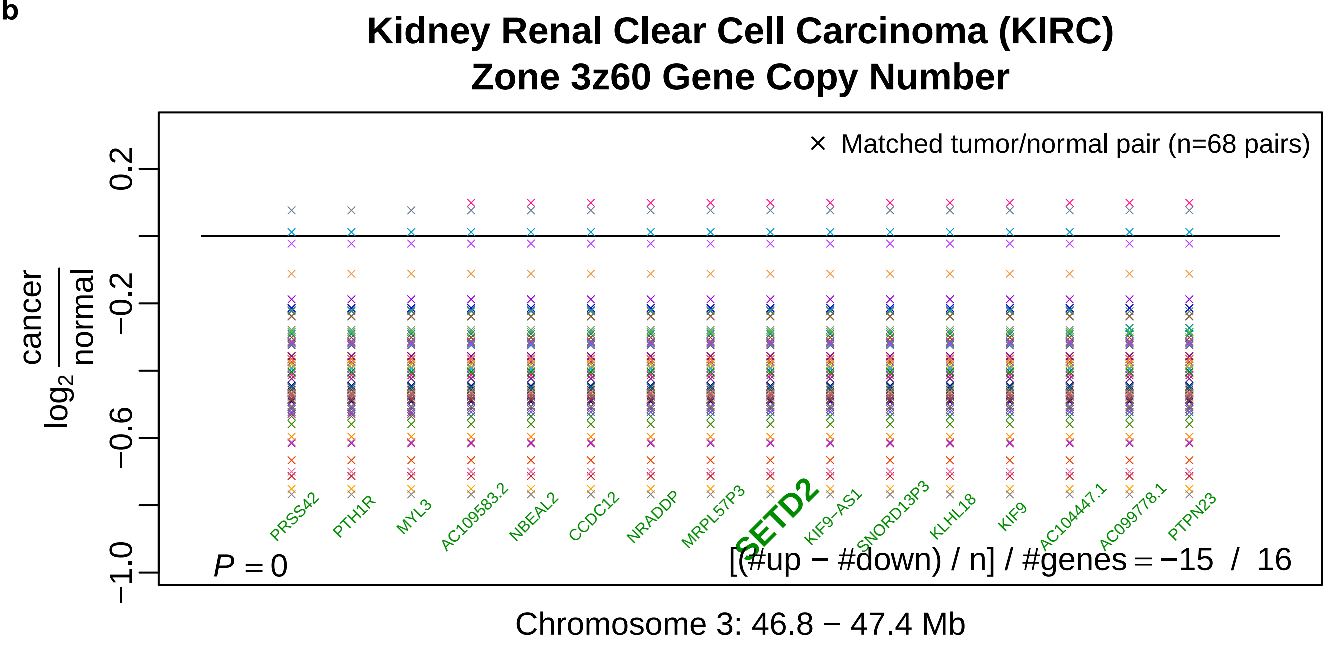

Supplementary Figure S3.59: The fourth most statistically significant polarized regulation zone in KIRC. a, The gene expression log ratio of cancer to normal for each gene within the zone in each patient. **b**, The somatic copy number log ratio of cancer to normal for each gene within the zone in each patient. See the full legend on page 3.

a

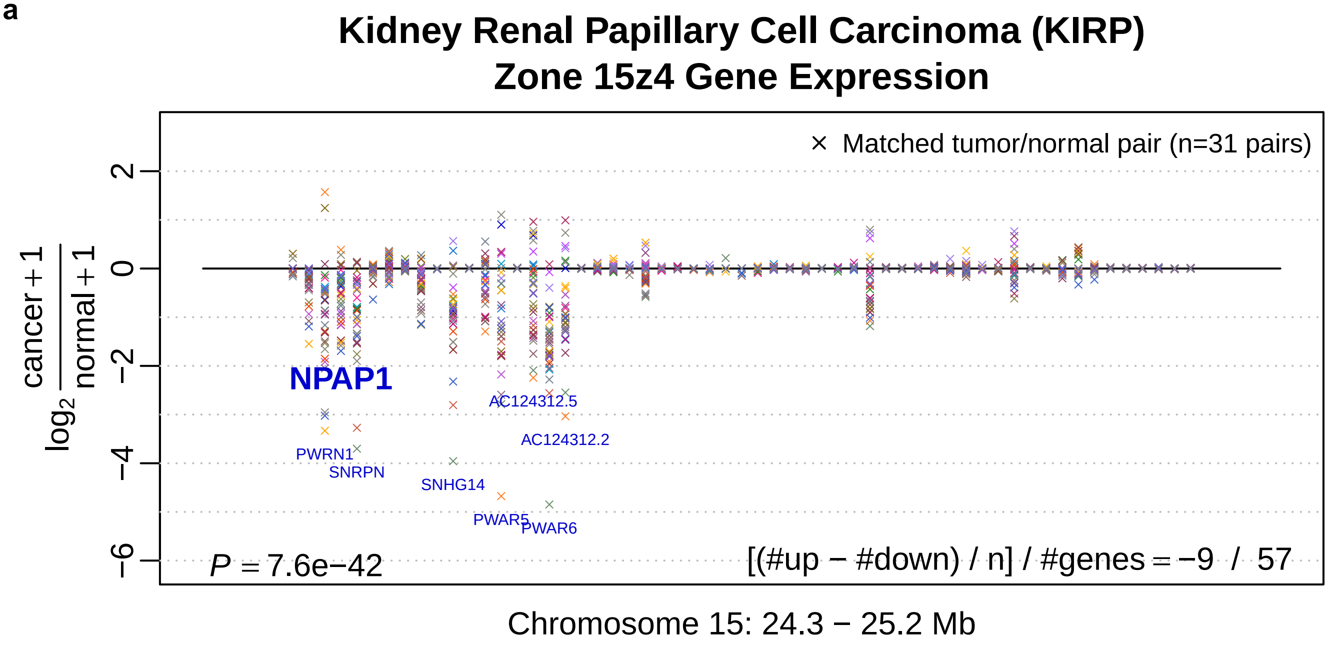

b

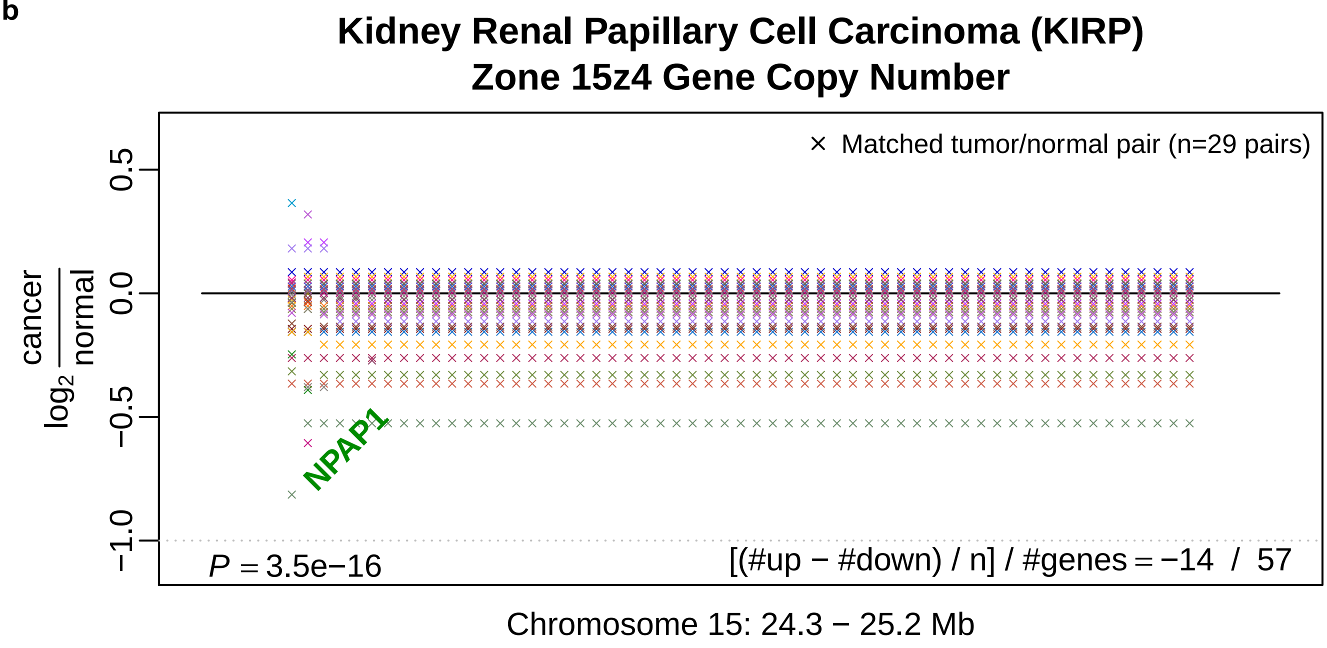

Supplementary Figure S3.60: The fourth most statistically significant polarized regulation zone in KIRP. a, The gene expression log ratio of cancer to normal for each gene within the zone in each patient. **b**, The somatic copy number log ratio of cancer to normal for each gene within the zone in each patient. See the full legend on page 3.

a

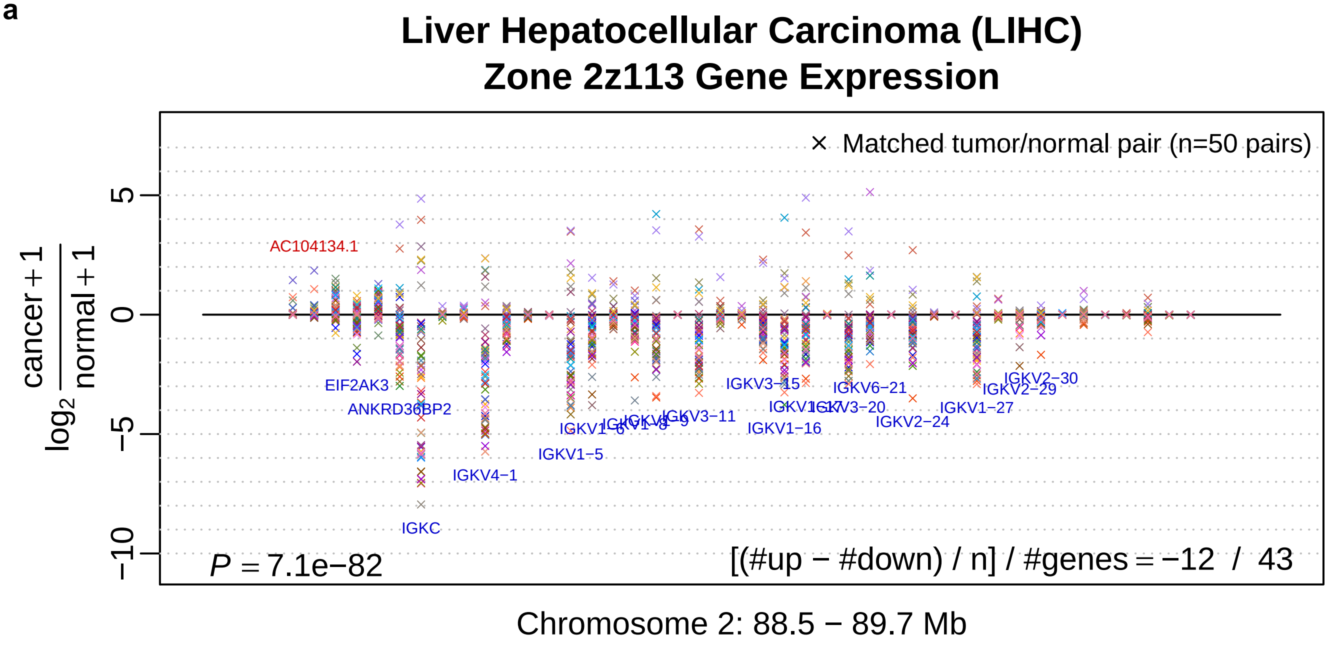

b

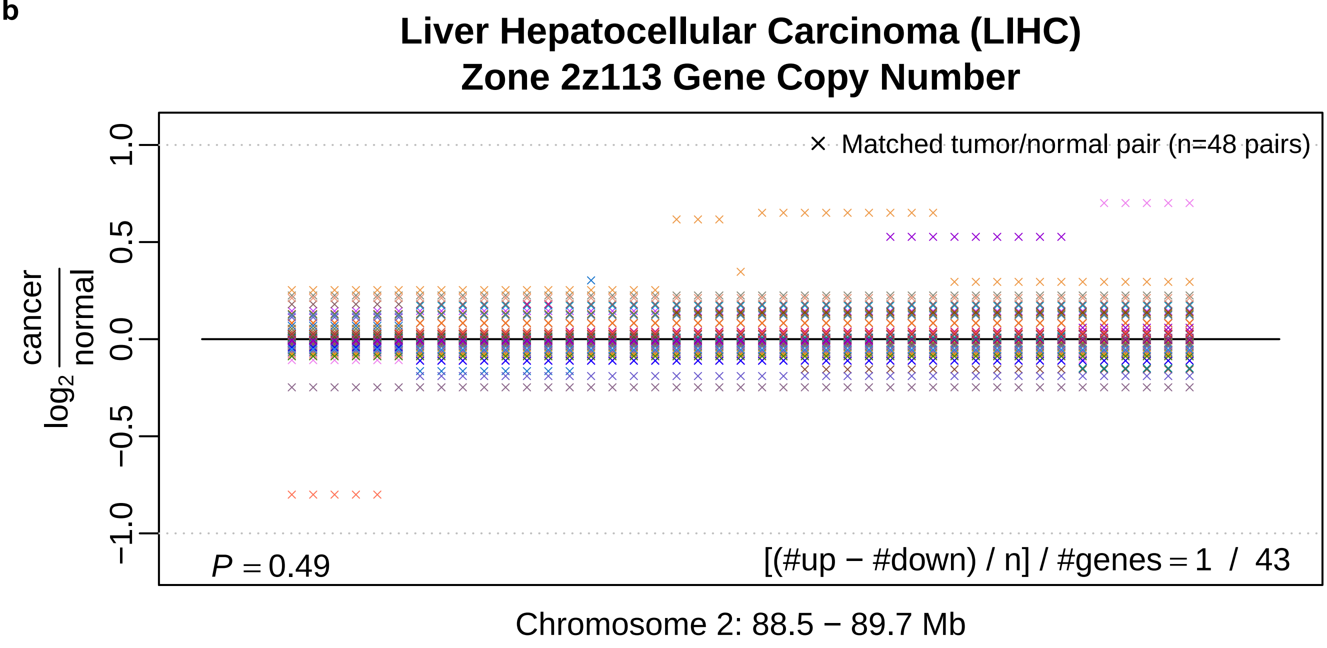

Supplementary Figure S3.61: The fourth most statistically significant polarized regulation zone in LIHC. a, The gene expression log ratio of cancer to normal for each gene within the zone in each patient. **b**, The somatic copy number log ratio of cancer to normal for each gene within the zone in each patient. See the full legend on page 3.

a

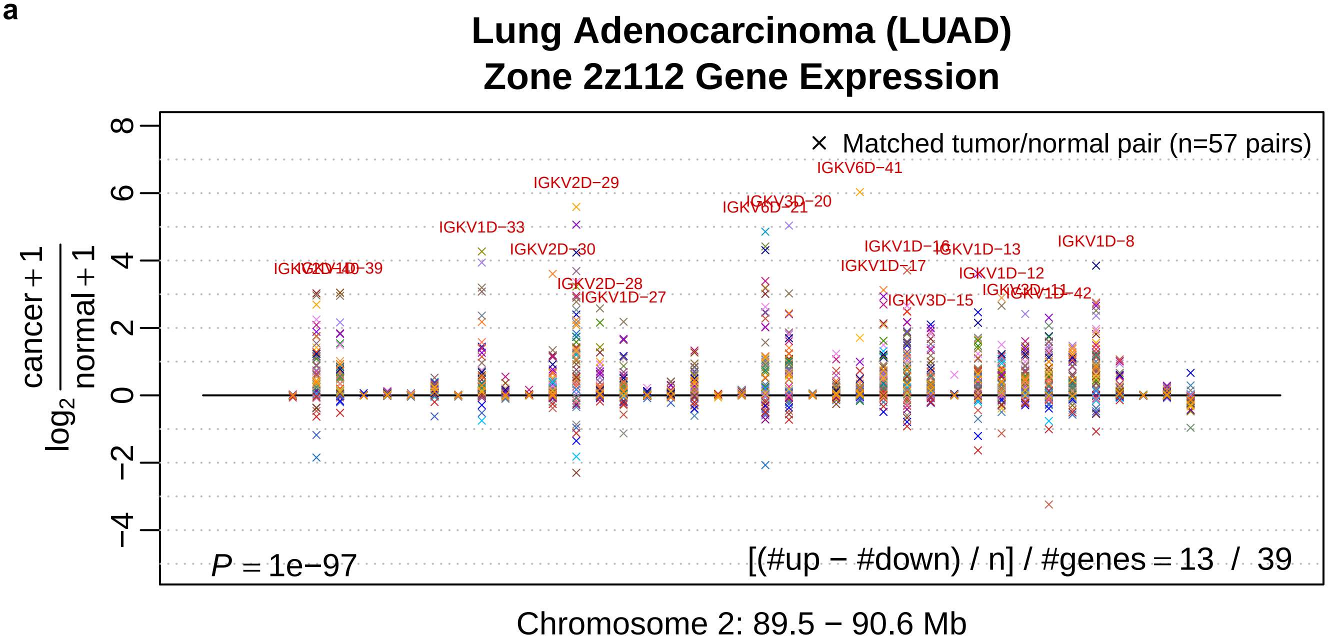

b

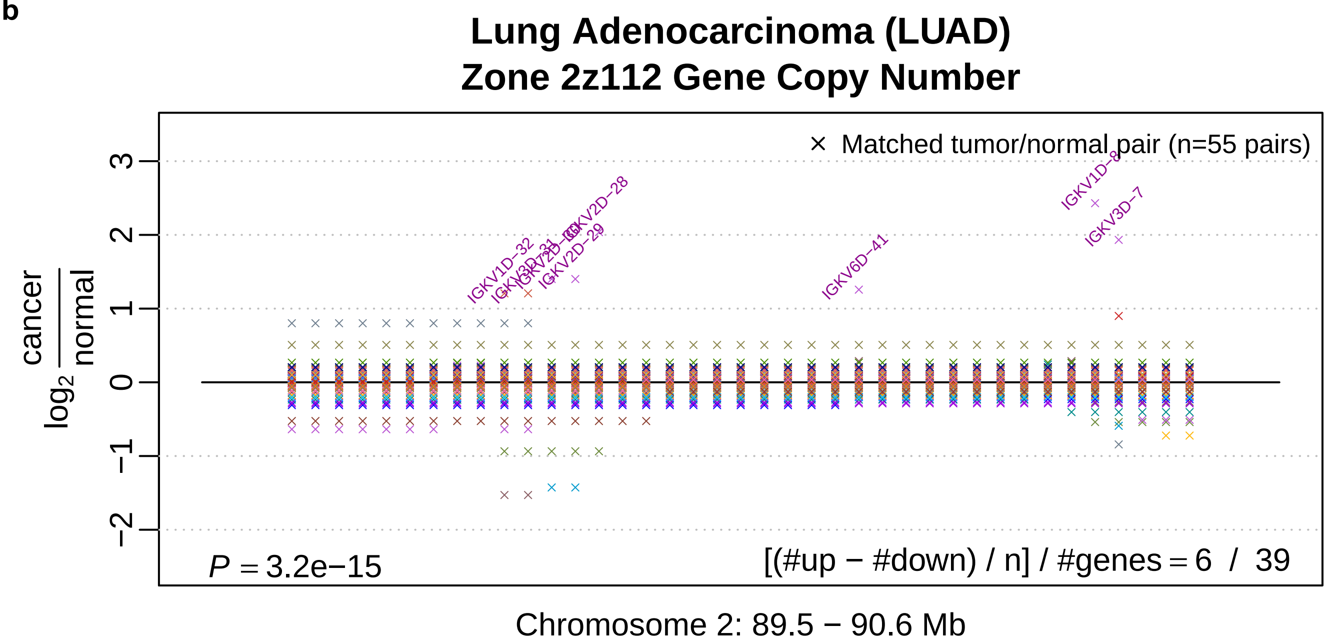

Supplementary Figure S3.62: The fourth most statistically significant polarized regulation zone in LUAD. a, The gene expression log ratio of cancer to normal for each gene within the zone in each patient. **b**, The somatic copy number log ratio of cancer to normal for each gene within the zone in each patient. See the full legend on page 3.

a

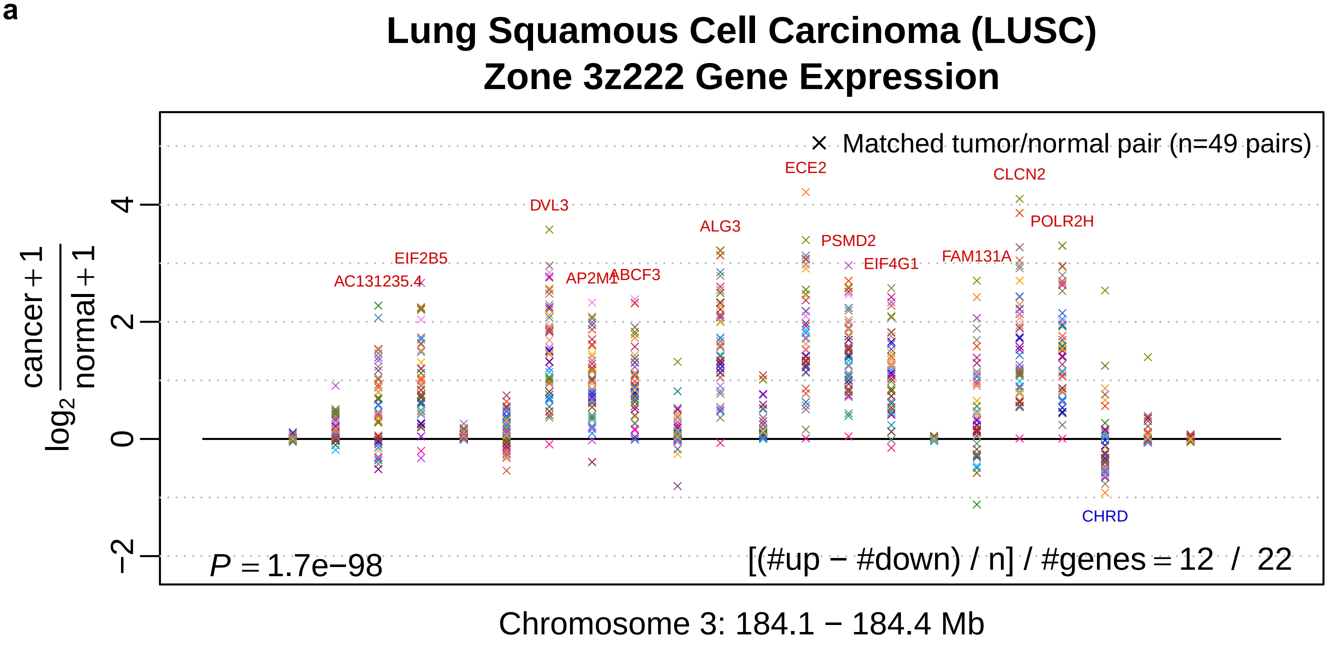

b

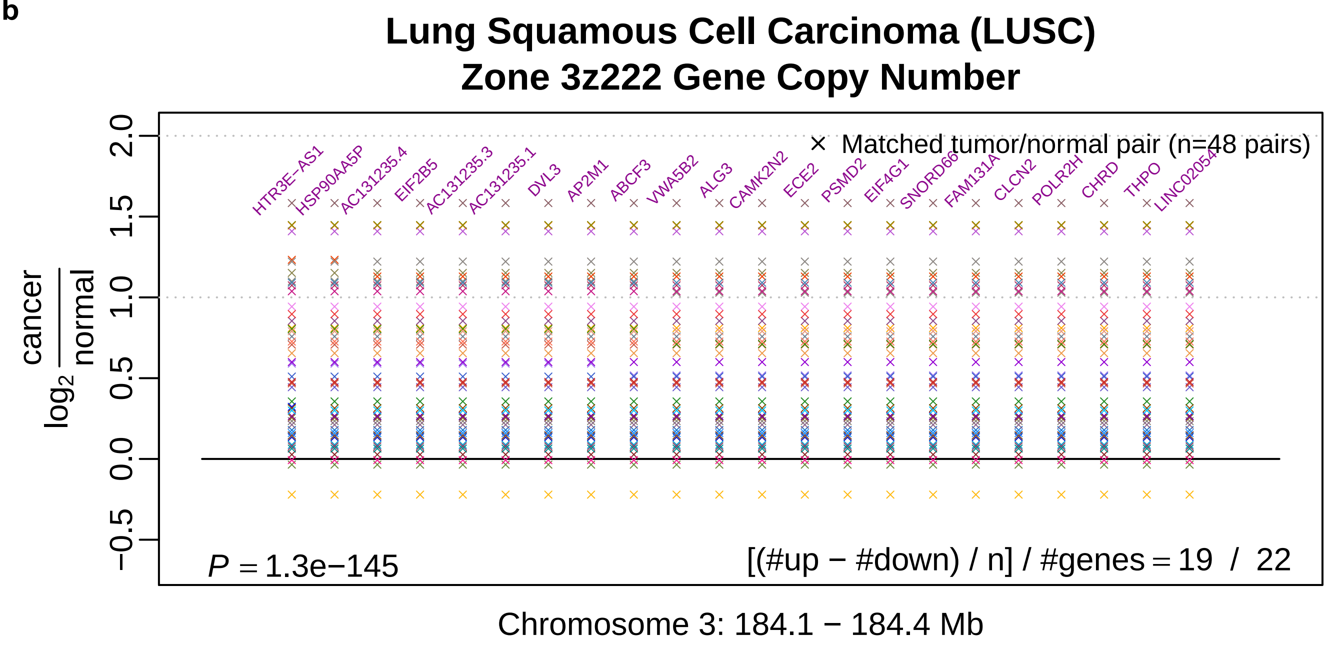

Supplementary Figure S3.63: The fourth most statistically significant polarized regulation zone in LUSC. a, The gene expression log ratio of cancer to normal for each gene within the zone in each patient. **b**, The somatic copy number log ratio of cancer to normal for each gene within the zone in each patient. See the full legend on page 3.

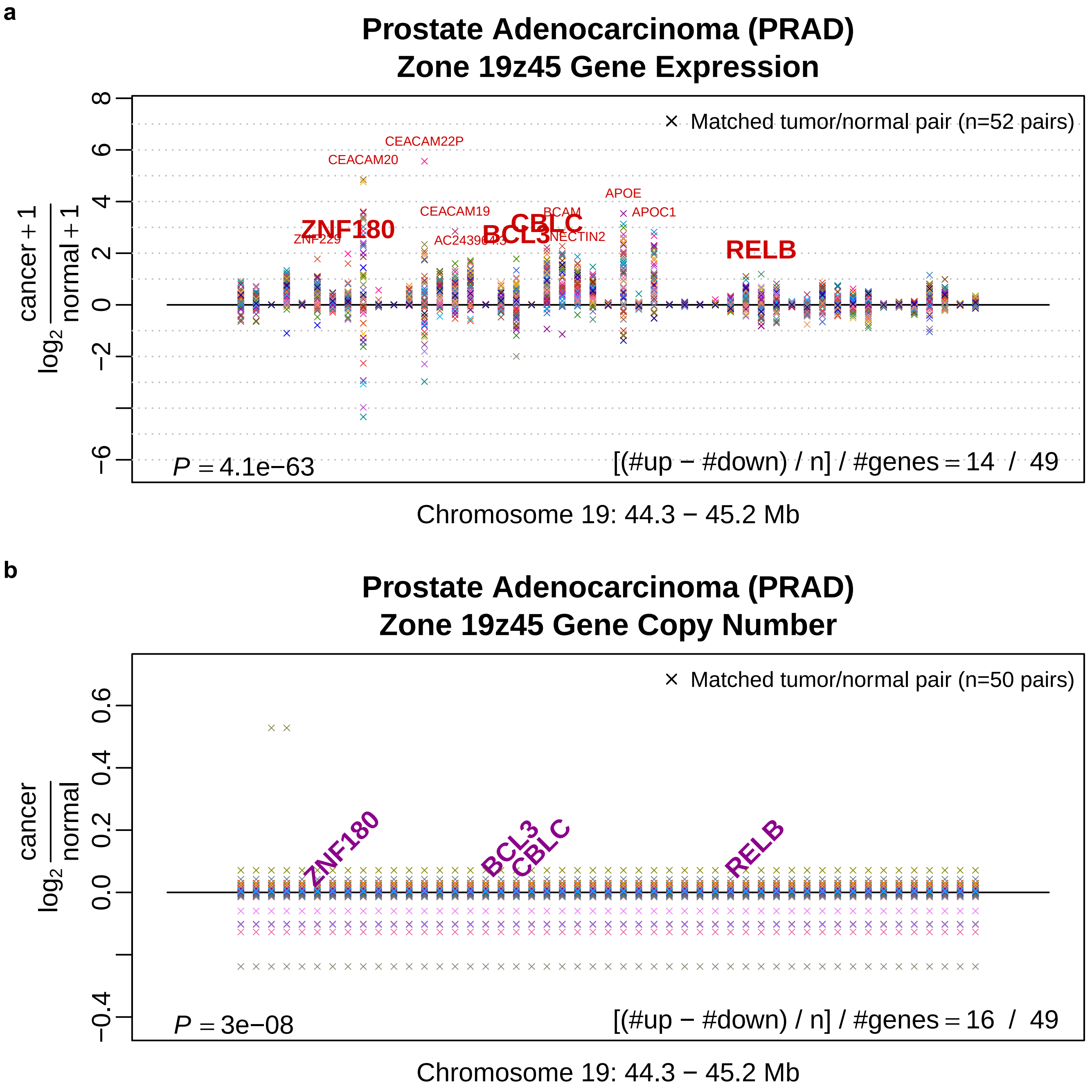

Supplementary Figure S3.64: The fourth most statistically significant polarized regulation zone in PRAD. a, The gene expression log ratio of cancer to normal for each gene within the zone in each patient. **b**, The somatic copy number log ratio of cancer to normal for each gene within the zone in each patient. See the full legend on page 3.

a

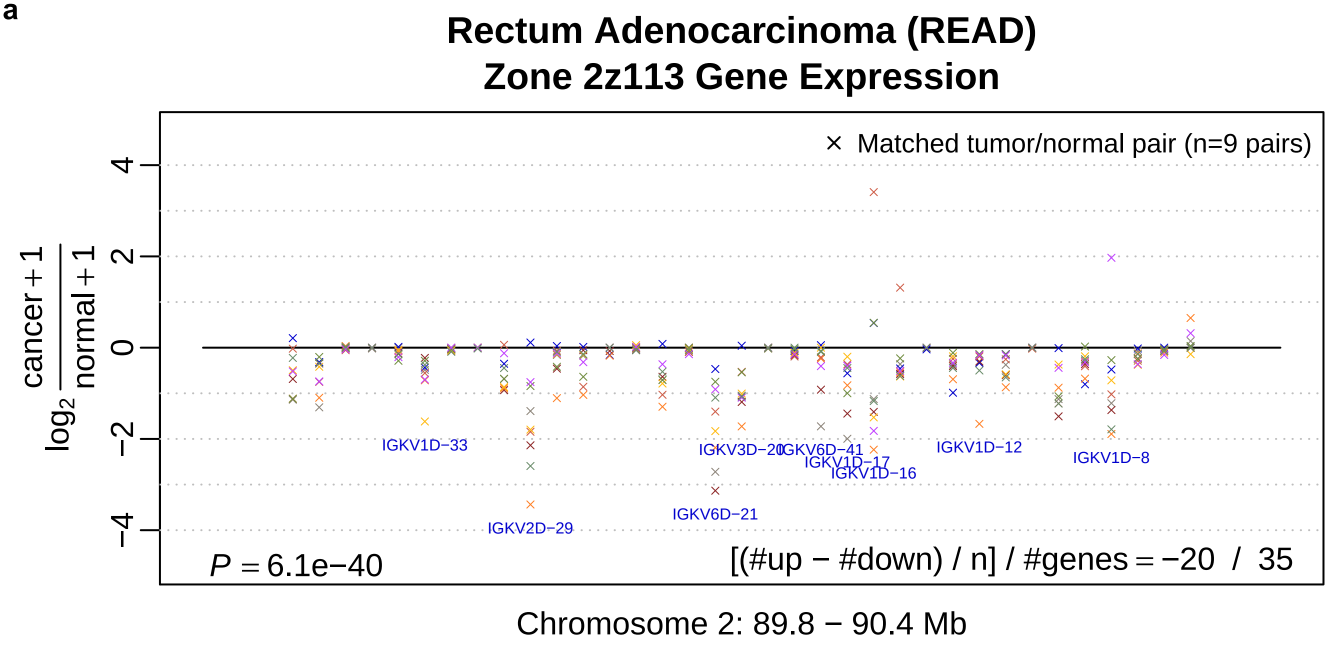

b

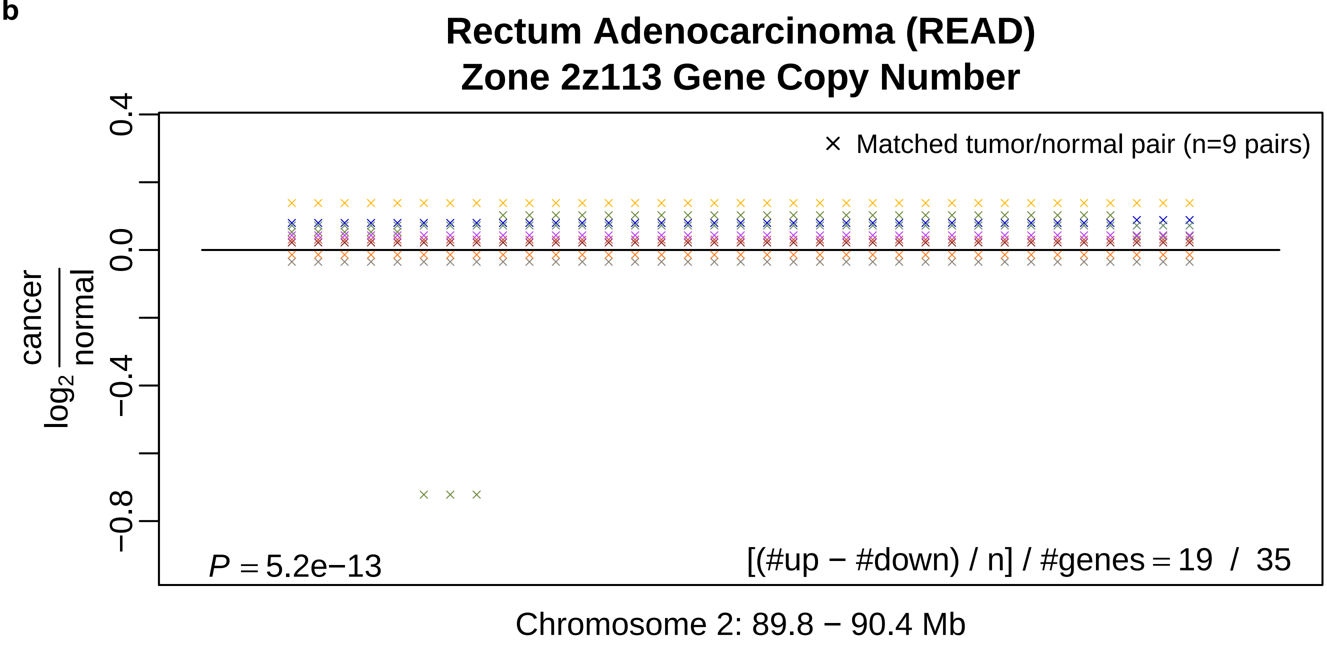

Supplementary Figure S3.65: The fourth most statistically significant polarized regulation zone in READ. a, The gene expression log ratio of cancer to normal for each gene within the zone in each patient. **b**, The somatic copy number log ratio of cancer to normal for each gene within the zone in each patient. See the full legend on page 3.

a

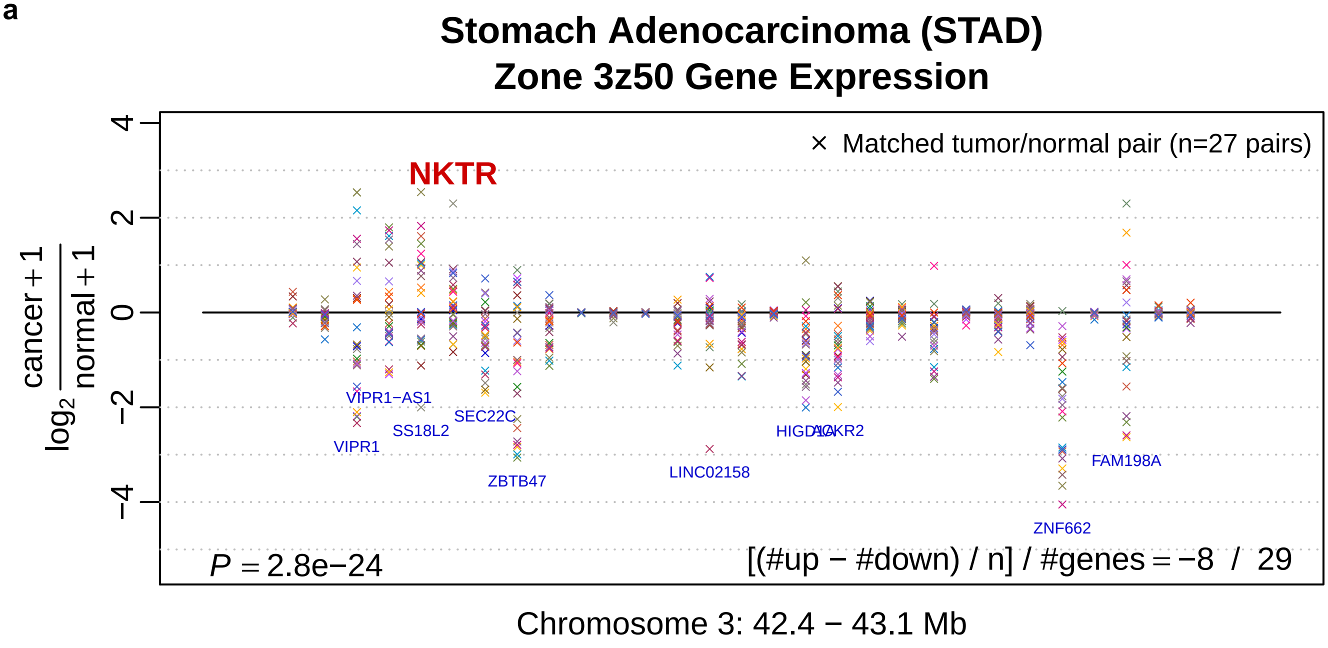

b

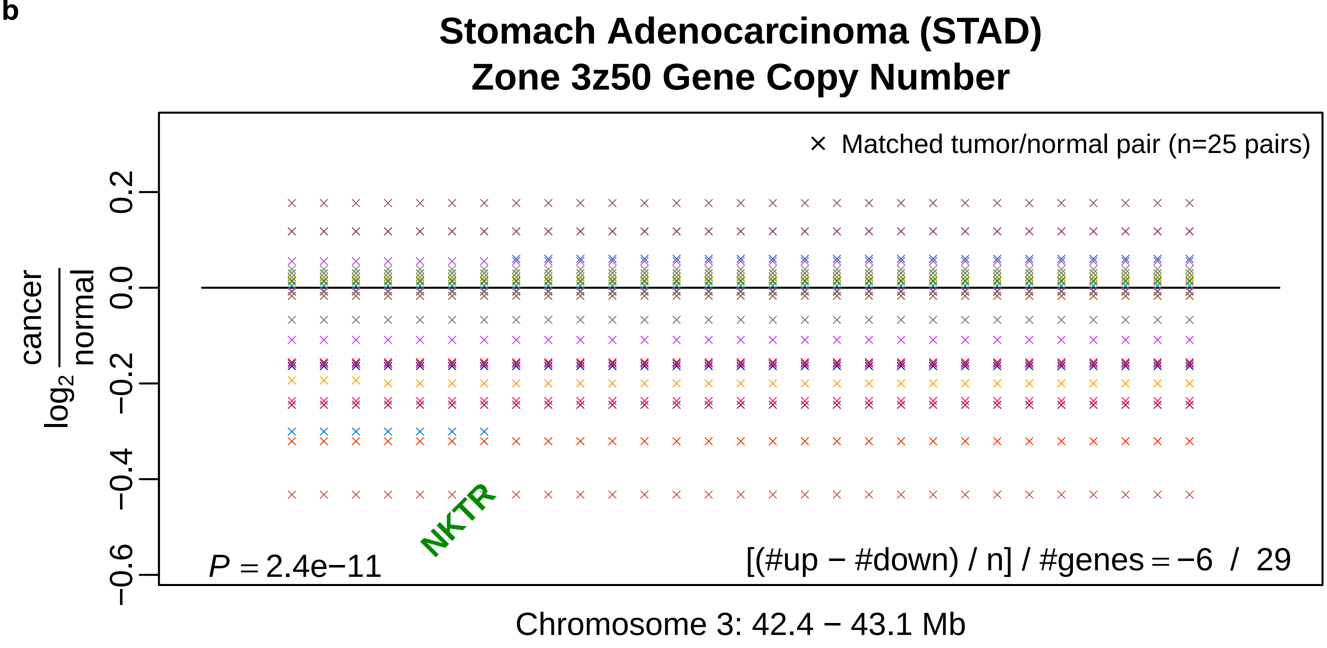

Supplementary Figure S3.66: The fourth most statistically significant polarized regulation zone in STAD. a, The gene expression log ratio of cancer to normal for each gene within the zone in each patient. **b**, The somatic copy number log ratio of cancer to normal for each gene within the zone in each patient. See the full legend on page 3.

a

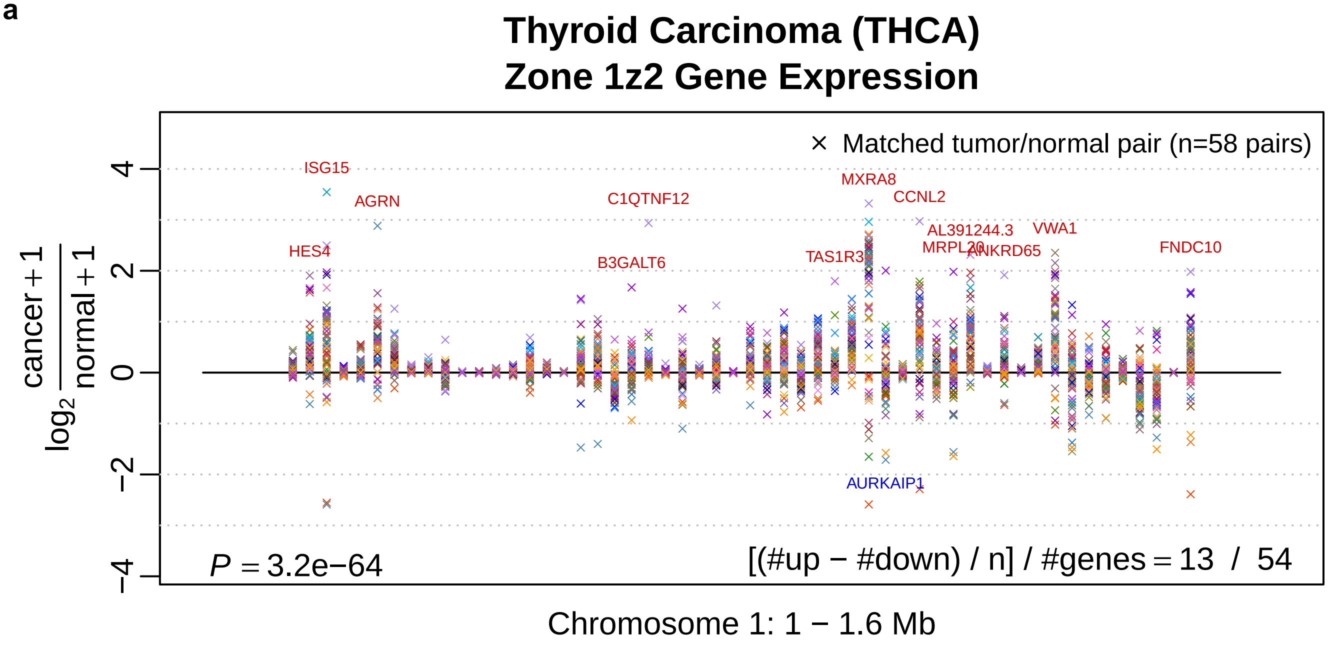

b

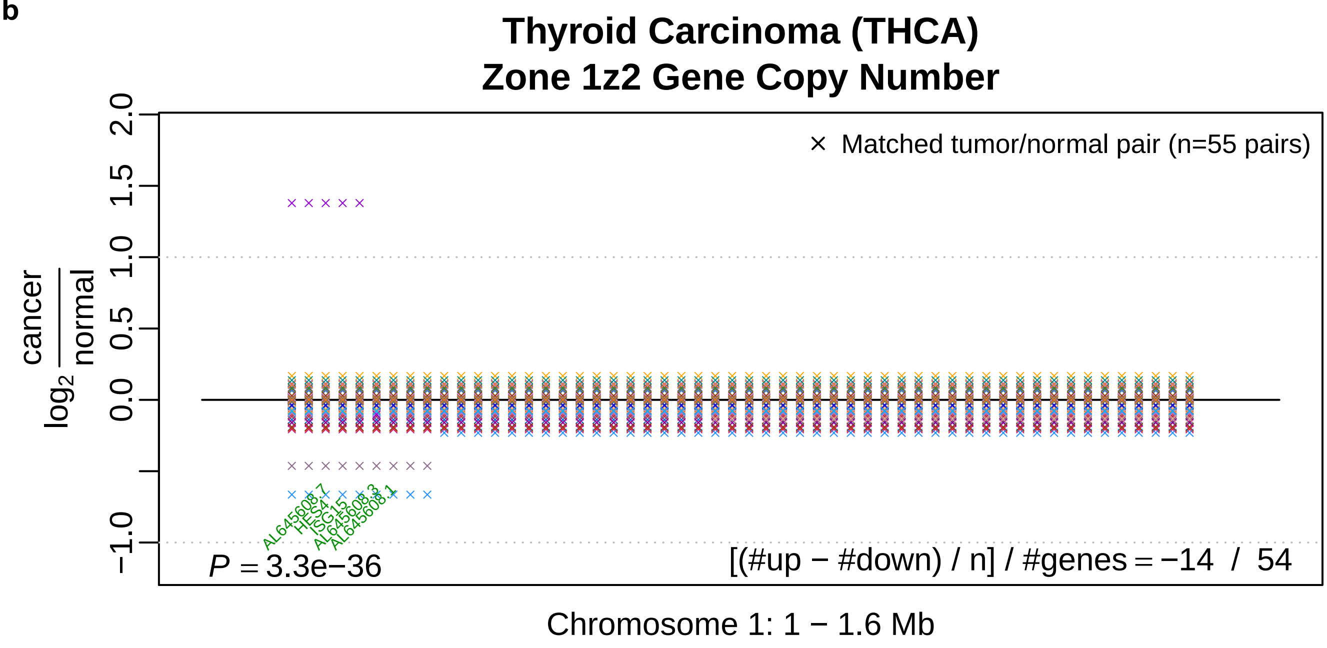

Supplementary Figure S3.67: The fourth most statistically significant polarized regulation zone in THCA. a, The gene expression log ratio of cancer to normal for each gene within the zone in each patient. **b**, The somatic copy number log ratio of cancer to normal for each gene within the zone in each patient. See the full legend on page 3.

a

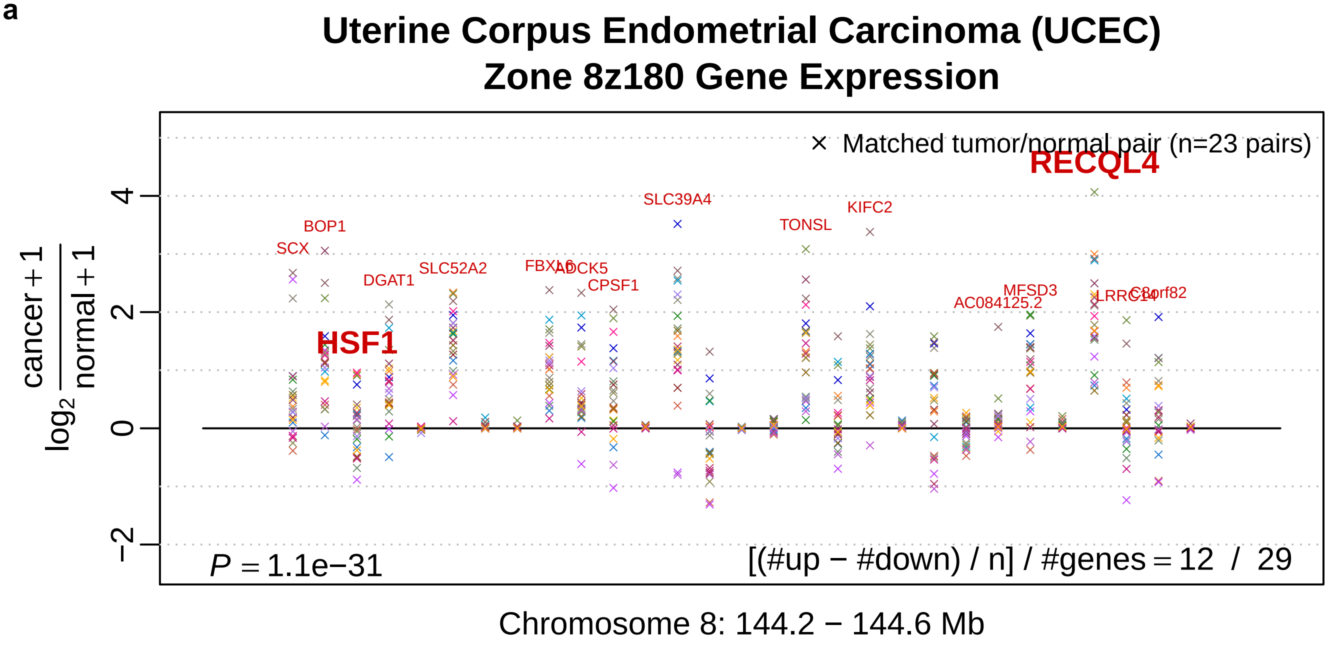

b

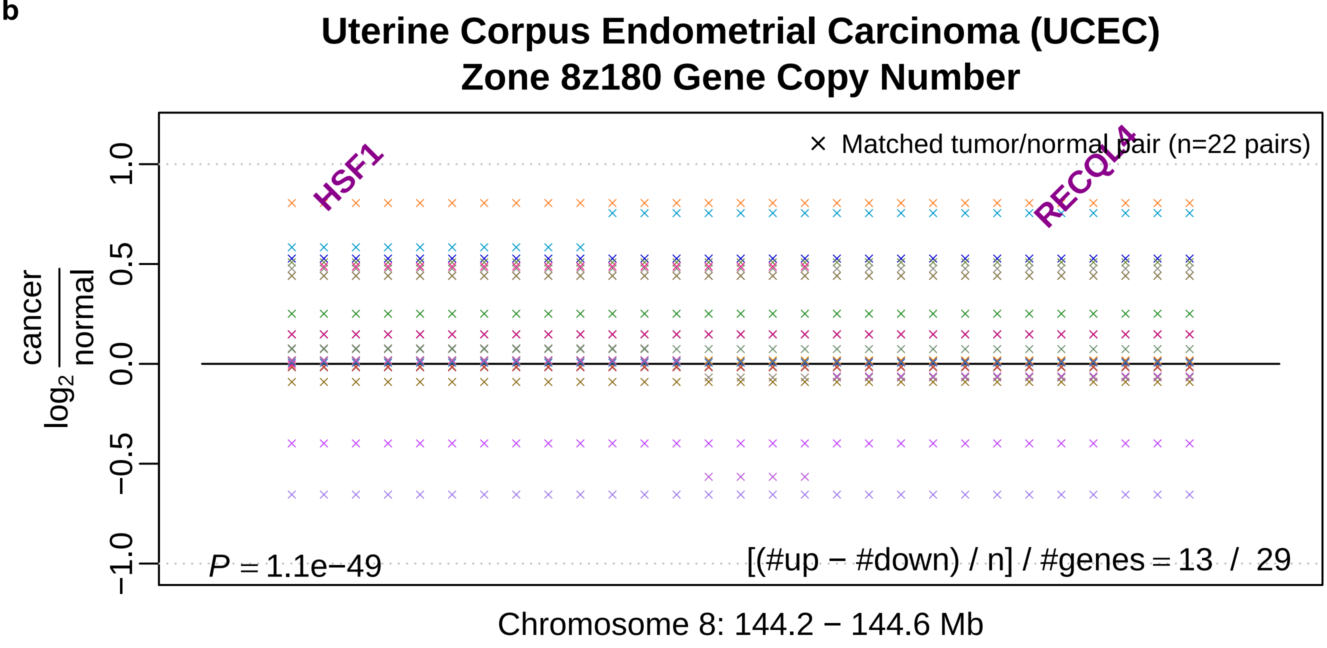

Supplementary Figure S3.68: The fourth most statistically significant polarized regulation zone in UCEC. a, The gene expression log ratio of cancer to normal for each gene within the zone in each patient. **b**, The somatic copy number log ratio of cancer to normal for each gene within the zone in each patient. See the full legend on page 3.

4 Fifth most polarized regulation zones of 17 cancer types

a

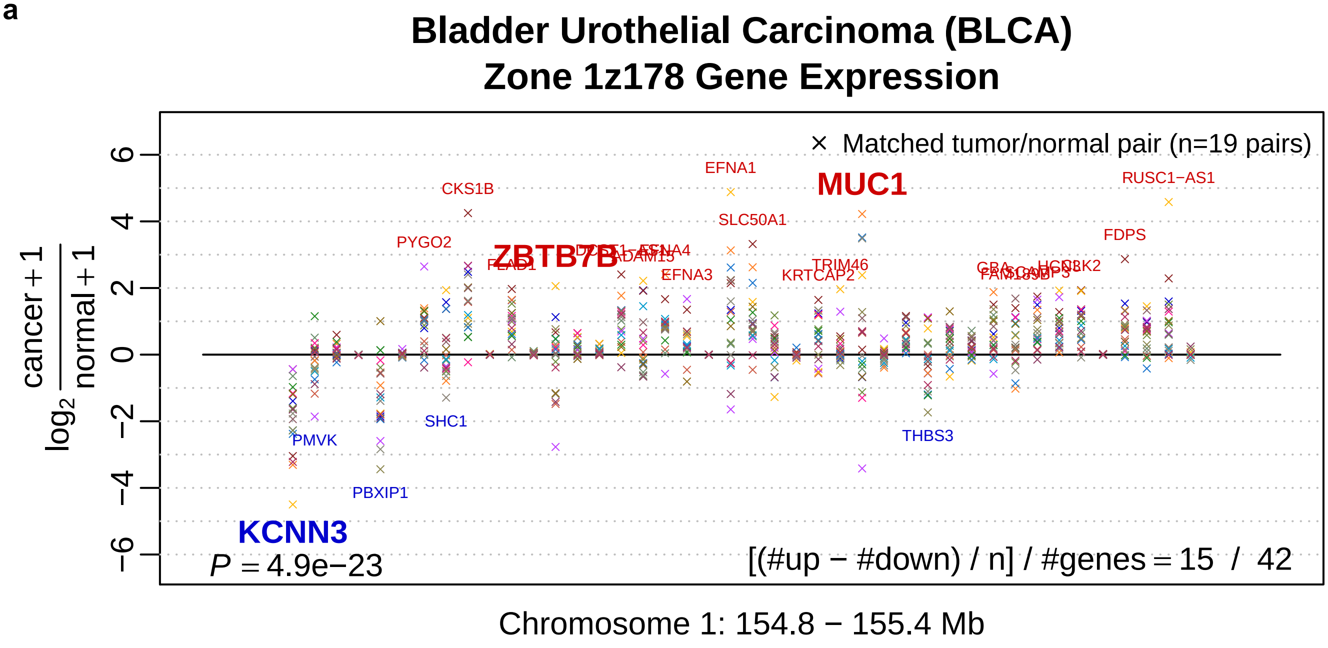

b

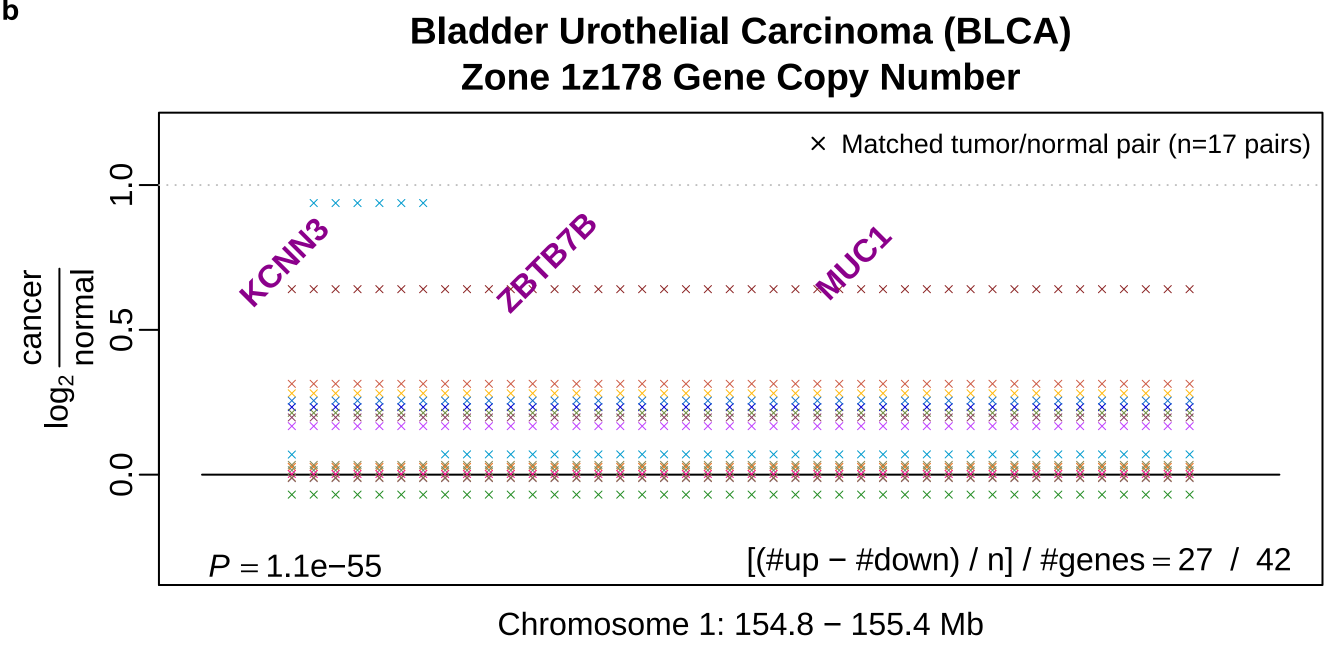

Supplementary Figure S3.69: The fifth most statistically significant polarized regulation zone in BLCA. a, The gene expression log ratio of cancer to normal for each gene within the zone in each patient. **b**, The somatic copy number log ratio of cancer to normal for each gene within the zone in each patient. See the full legend on page 3.

a

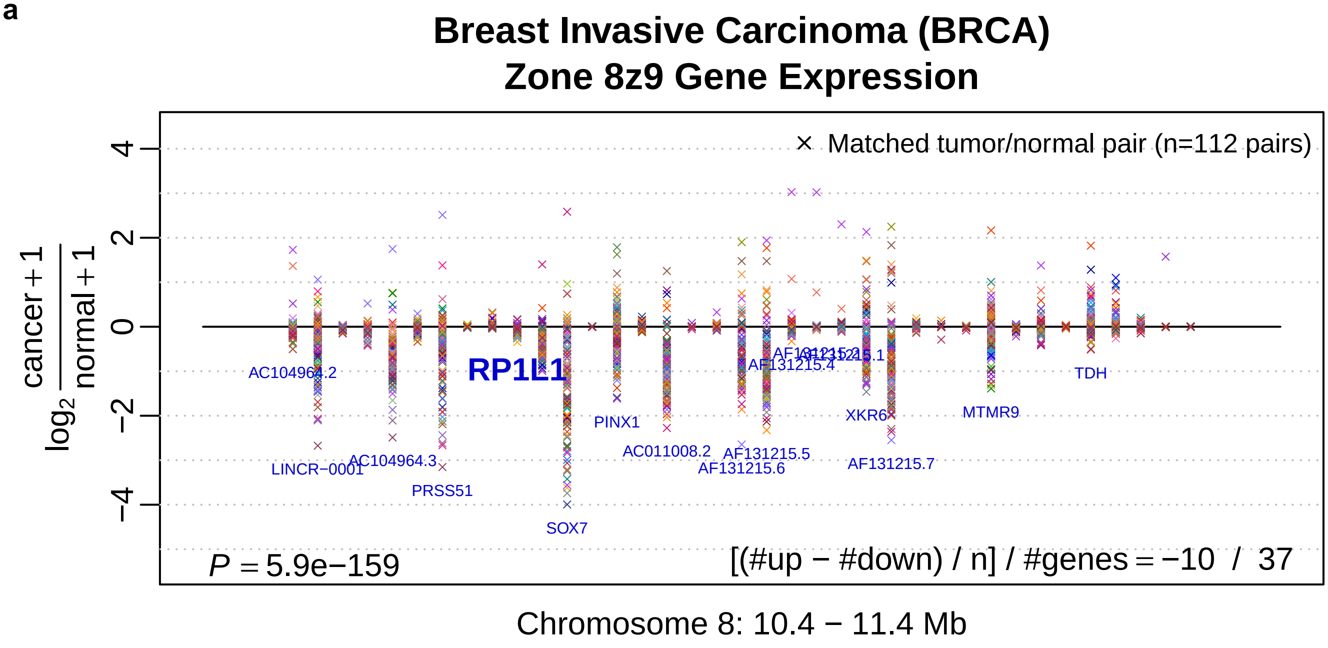

b

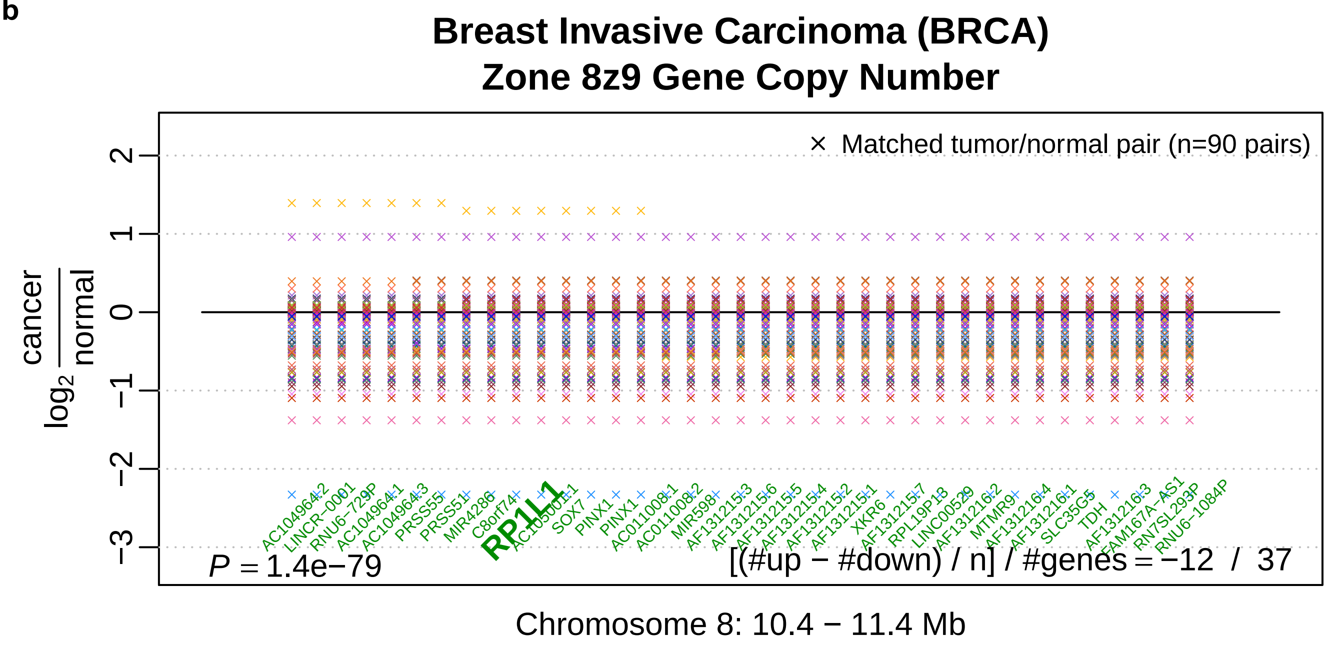

Supplementary Figure S3.70: The fifth most statistically significant polarized regulation zone in BRCA. a, The gene expression log ratio of cancer to normal for each gene within the zone in each patient. **b**, The somatic copy number log ratio of cancer to normal for each gene within the zone in each patient. See the full legend on page 3.

a

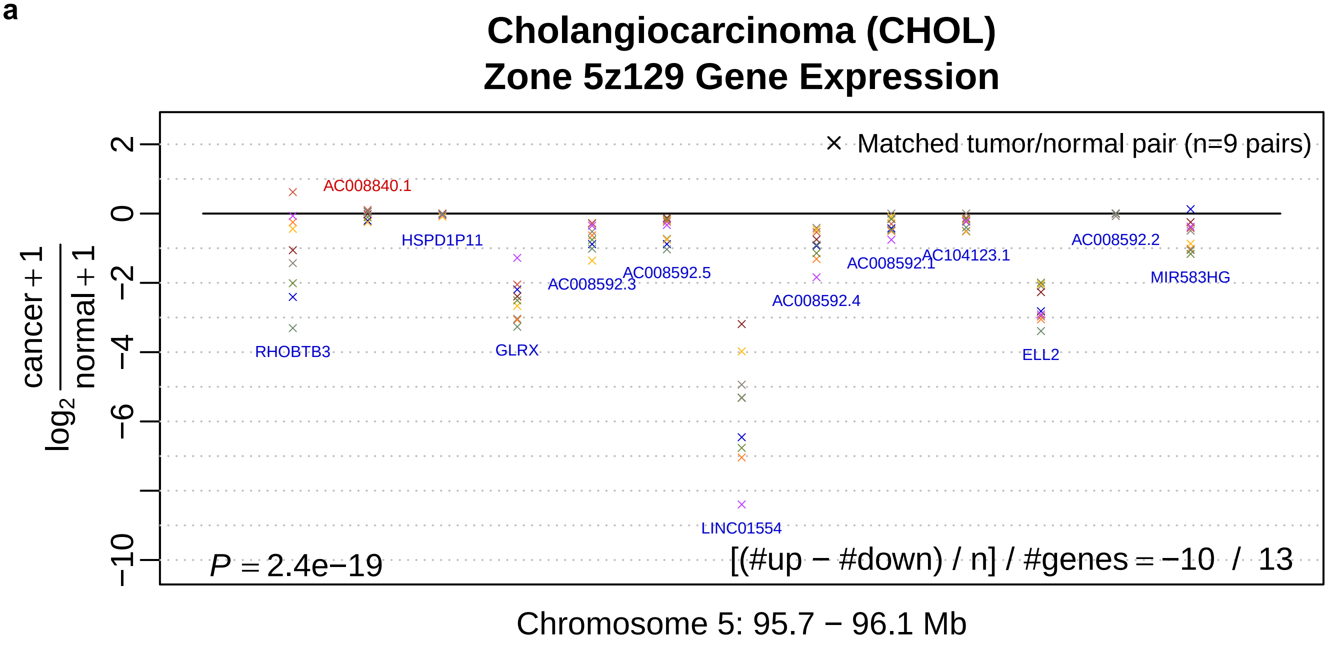

b

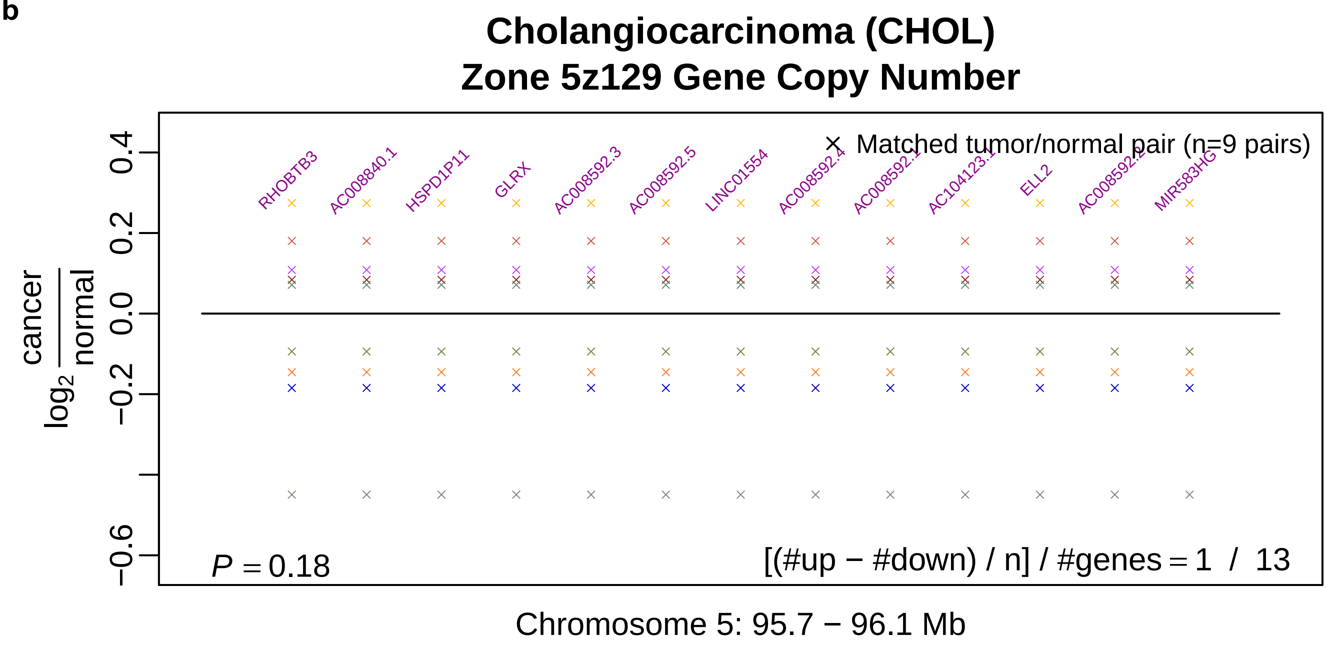

Supplementary Figure S3.71: The fifth most statistically significant polarized regulation zone in CHOL. a, The gene expression log ratio of cancer to normal for each gene within the zone in each patient. **b**, The somatic copy number log ratio of cancer to normal for each gene within the zone in each patient. See the full legend on page 3.

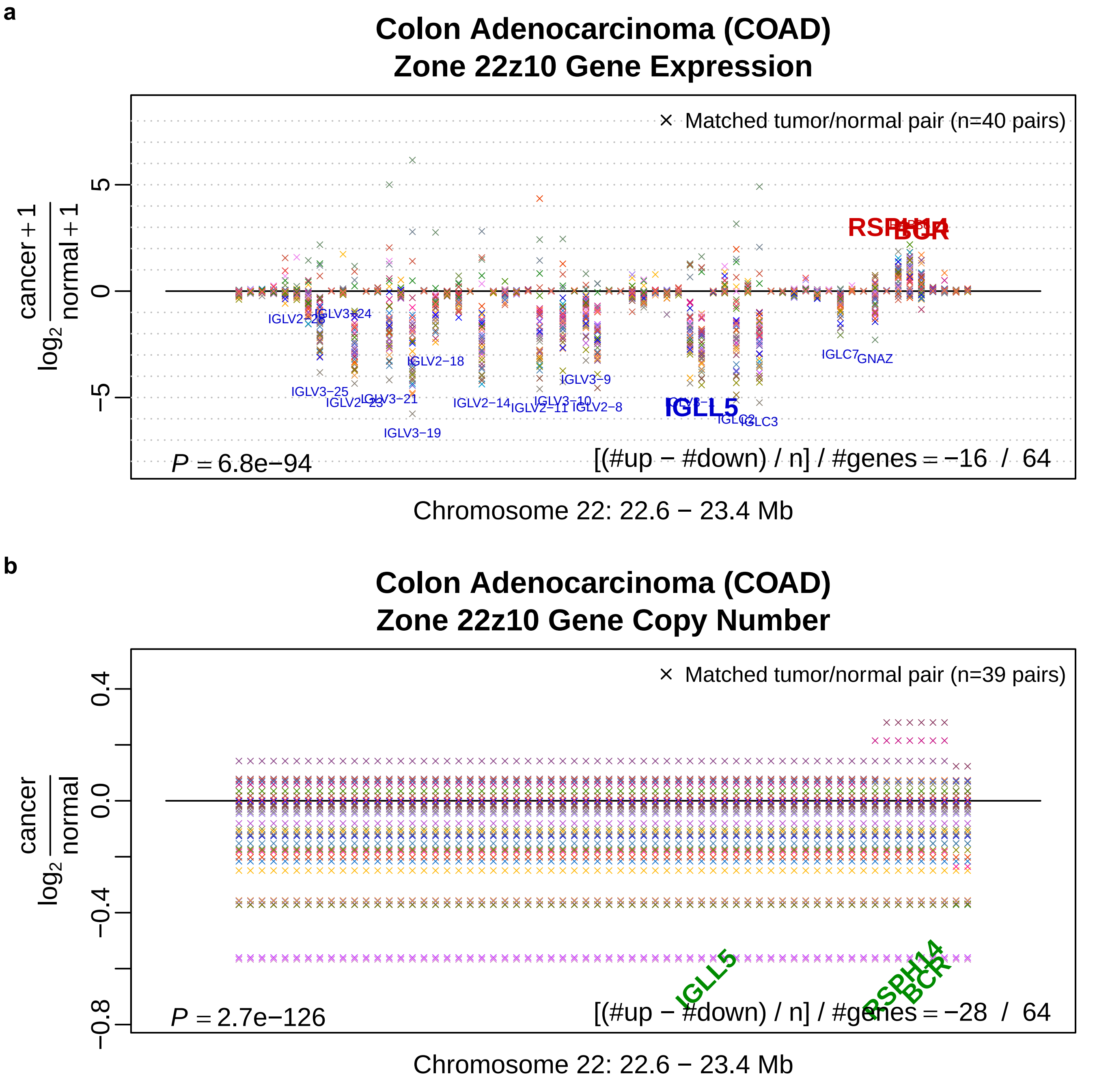

Supplementary Figure S3.72: The fifth most statistically significant polarized regulation zone in COAD. a, The gene expression log ratio of cancer to normal for each gene within the zone in each patient. **b**, The somatic copy number log ratio of cancer to normal for each gene within the zone in each patient. See the full legend on page 3.

a

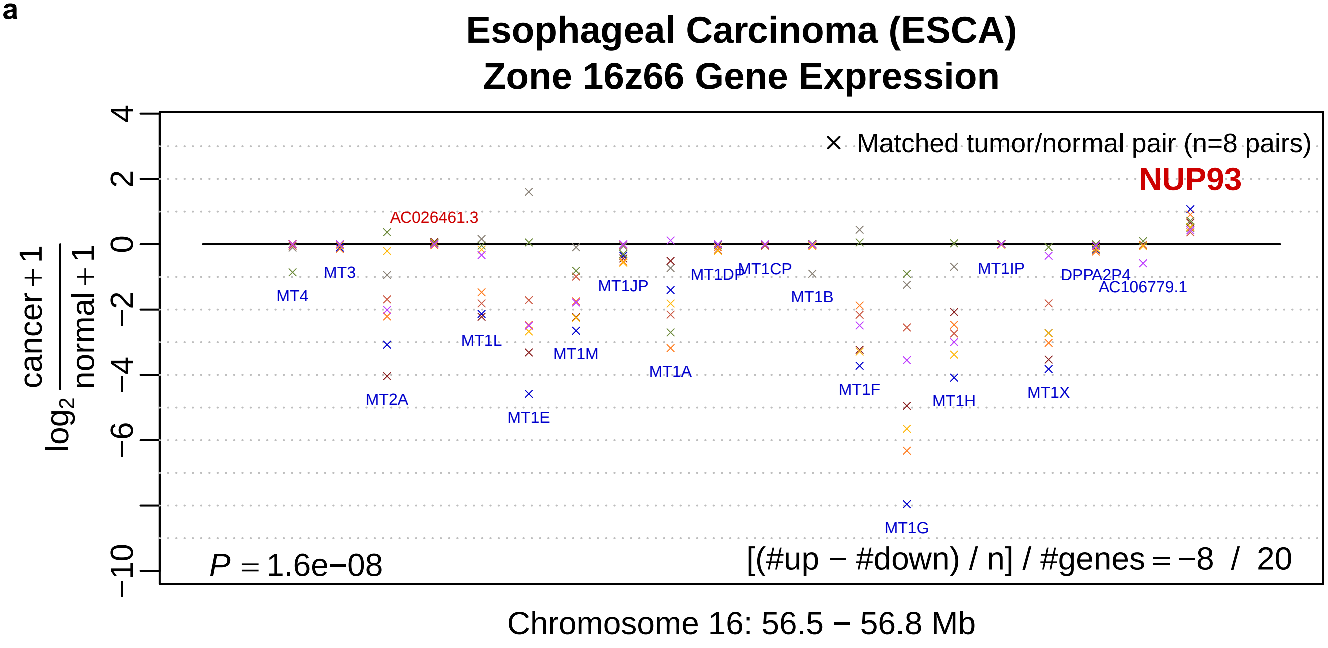

b

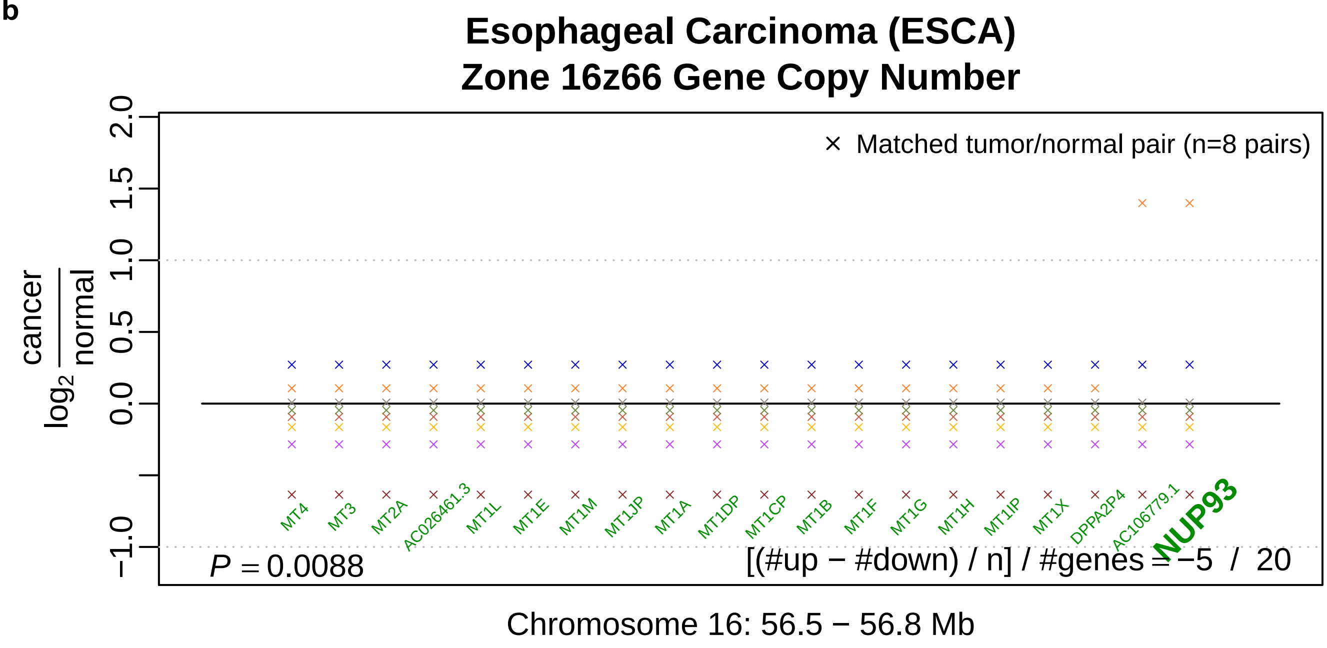

Supplementary Figure S3.73: The fifth most statistically significant polarized regulation zone in ESCA. a, The gene expression log ratio of cancer to normal for each gene within the zone in each patient. **b**, The somatic copy number log ratio of cancer to normal for each gene within the zone in each patient. See the full legend on page 3.

a

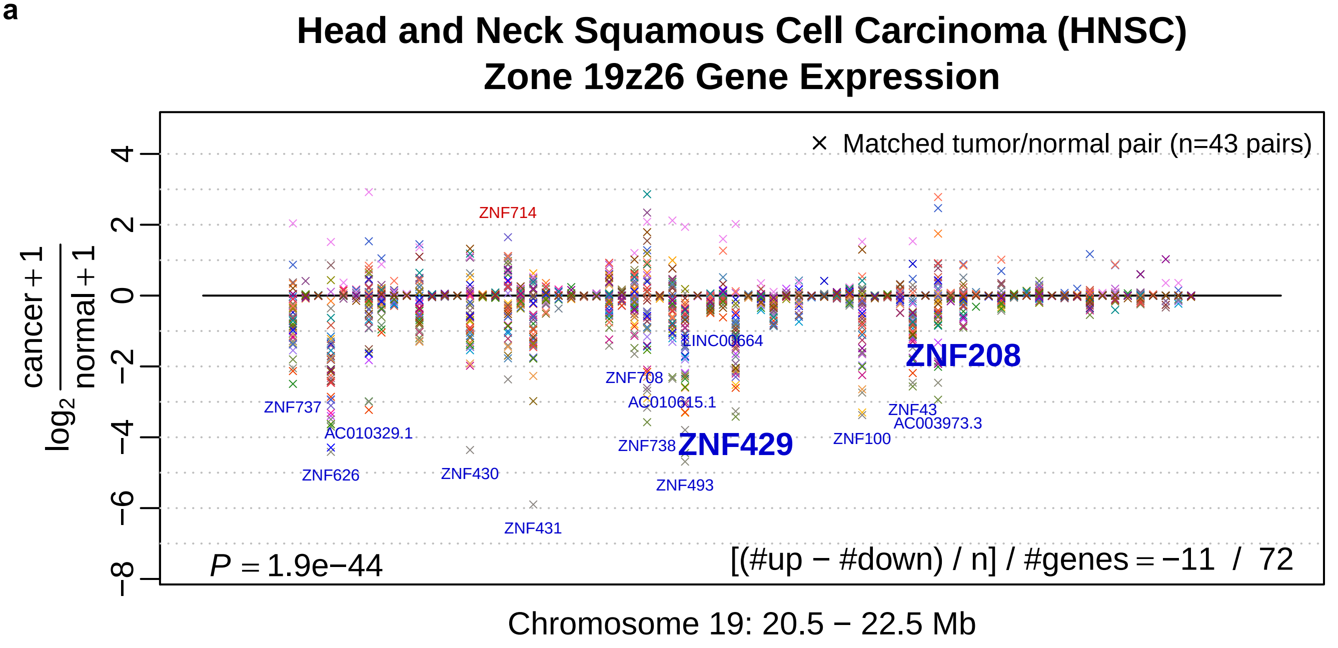

b

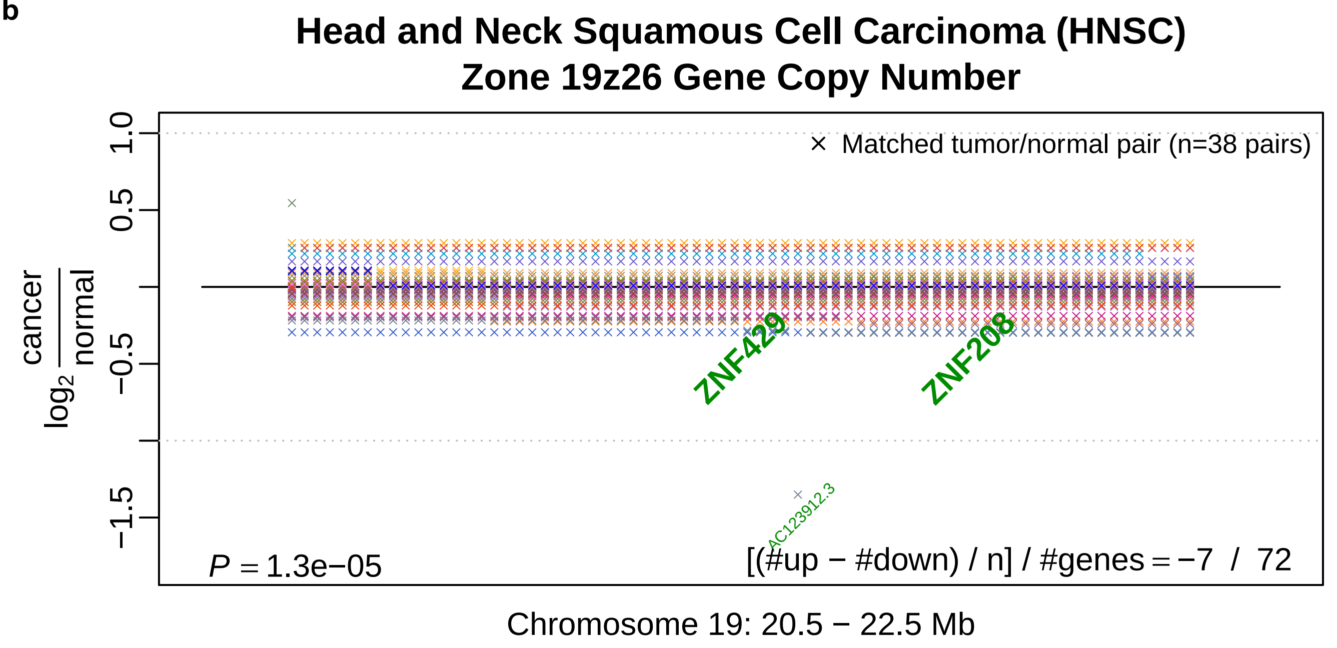

Supplementary Figure S3.74: The fifth most statistically significant polarized regulation zone in HNSC. a, The gene expression log ratio of cancer to normal for each gene within the zone in each patient. **b**, The somatic copy number log ratio of cancer to normal for each gene within the zone in each patient. See the full legend on page 3.

a

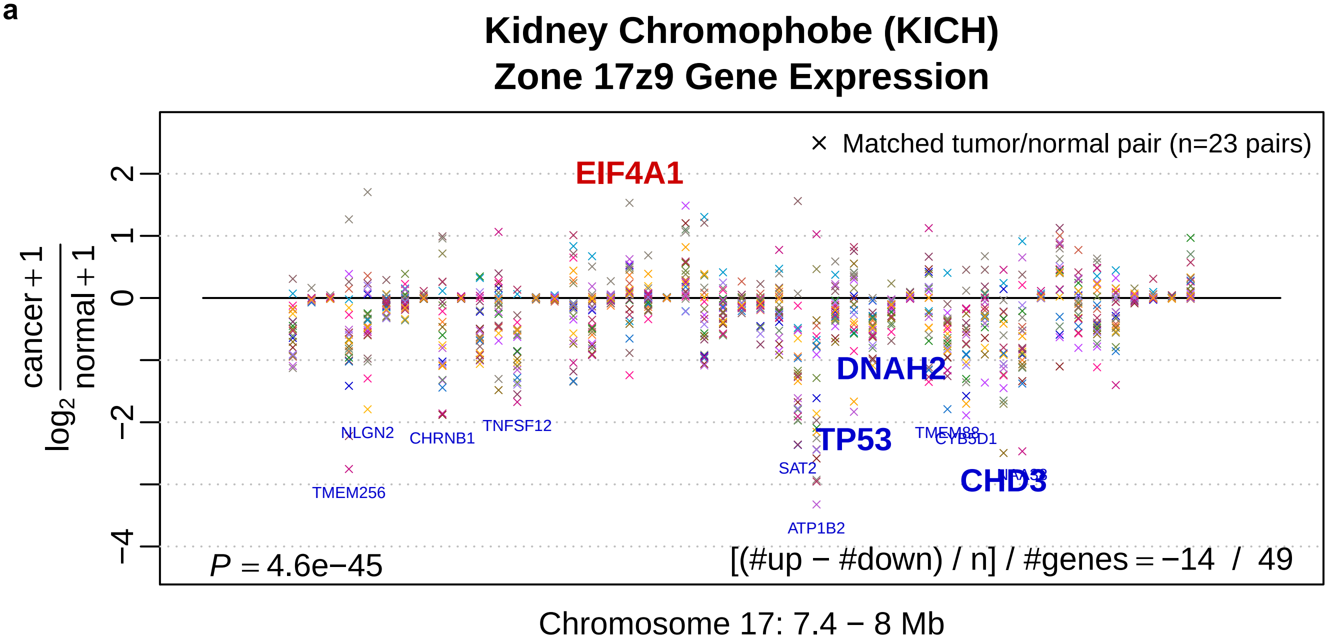

b

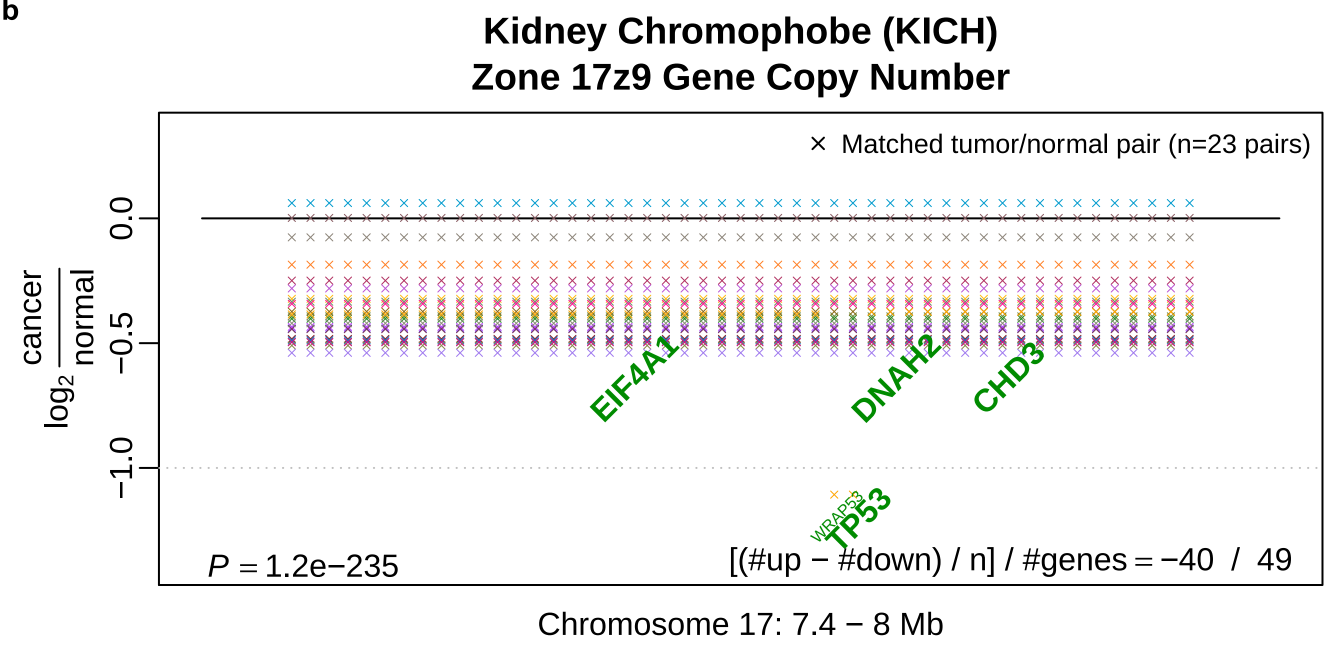

Supplementary Figure S3.75: The fifth most statistically significant polarized regulation zone in KICH. a, The gene expression log ratio of cancer to normal for each gene within the zone in each patient. **b**, The somatic copy number log ratio of cancer to normal for each gene within the zone in each patient. See the full legend on page 3.

a

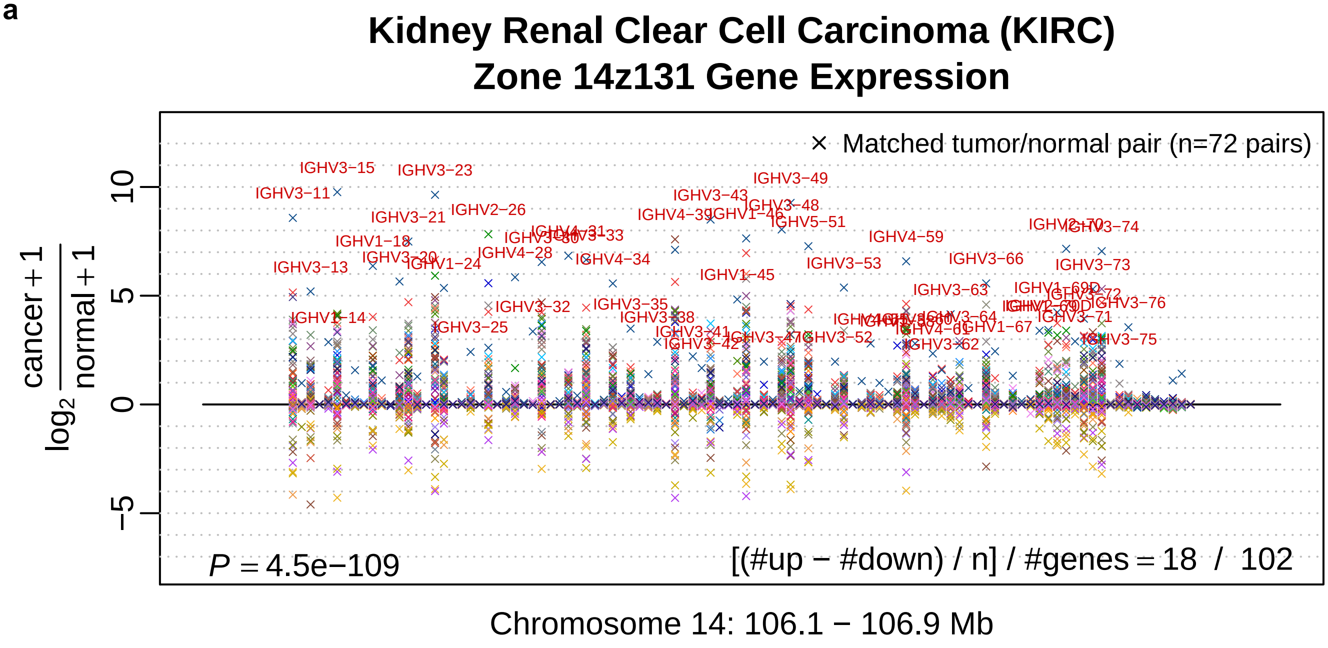

b

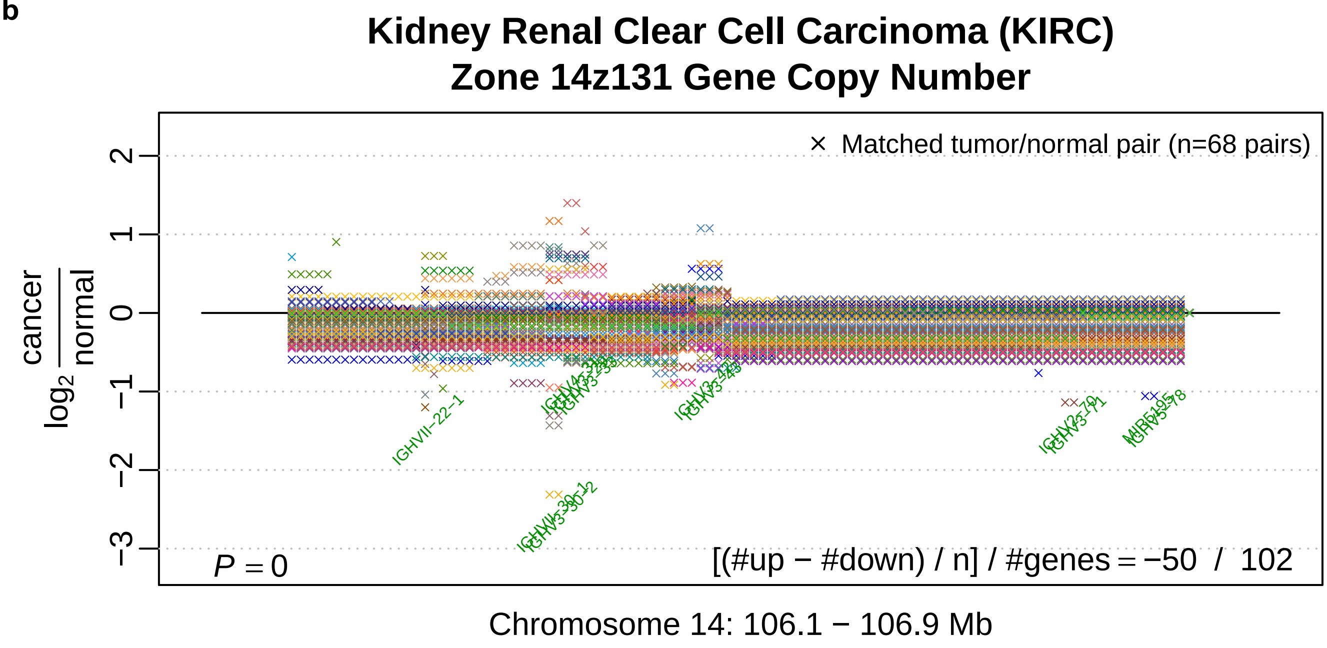

Supplementary Figure S3.76: The fifth most statistically significant polarized regulation zone in KIRC. a, The gene expression log ratio of cancer to normal for each gene within the zone in each patient. **b**, The somatic copy number log ratio of cancer to normal for each gene within the zone in each patient. See the full legend on page 3.

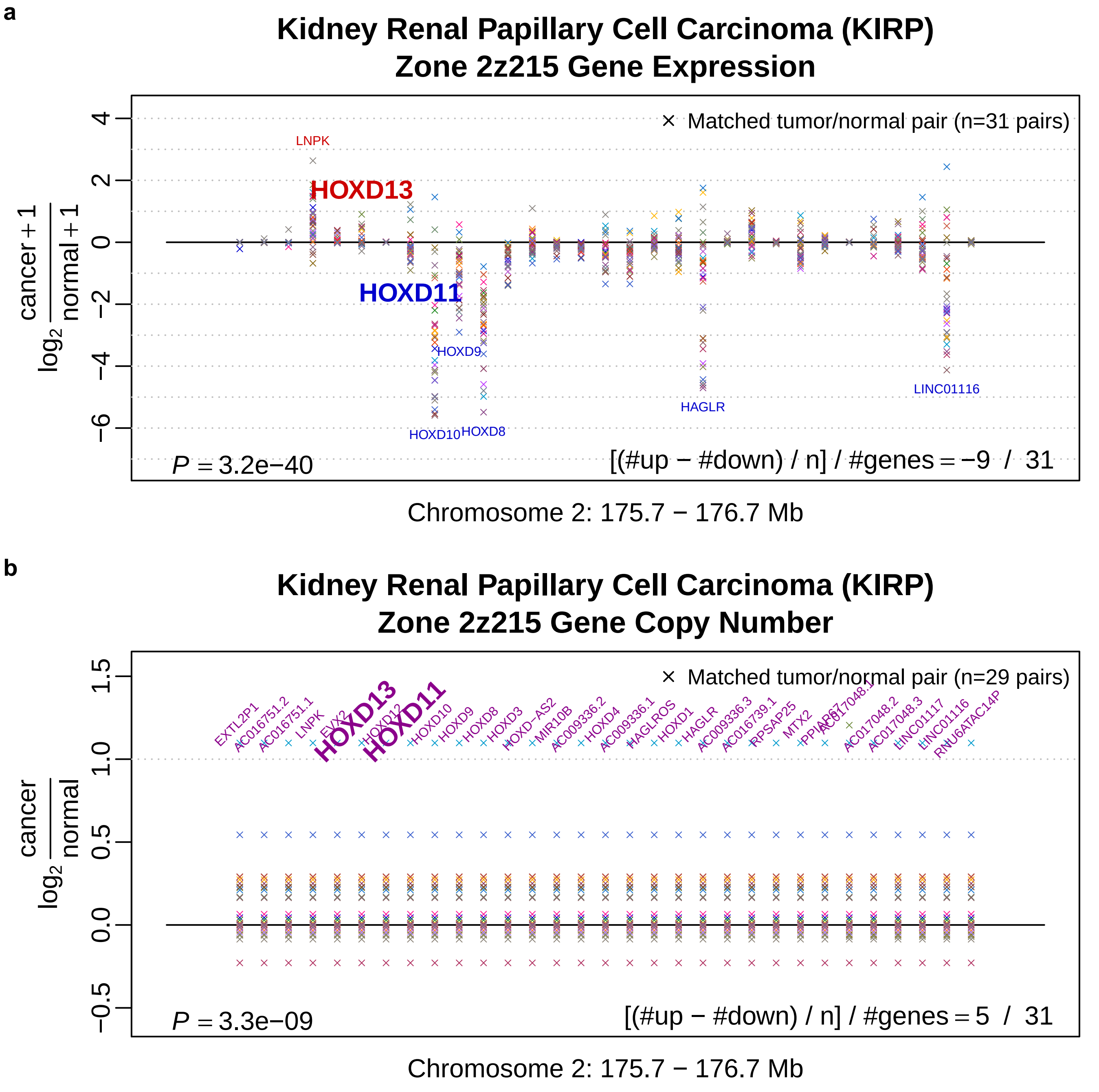

Supplementary Figure S3.77: The fifth most statistically significant polarized regulation zone in KIRP. a, The gene expression log ratio of cancer to normal for each gene within the zone in each patient. **b**, The somatic copy number log ratio of cancer to normal for each gene within the zone in each patient. See the full legend on page 3.

a

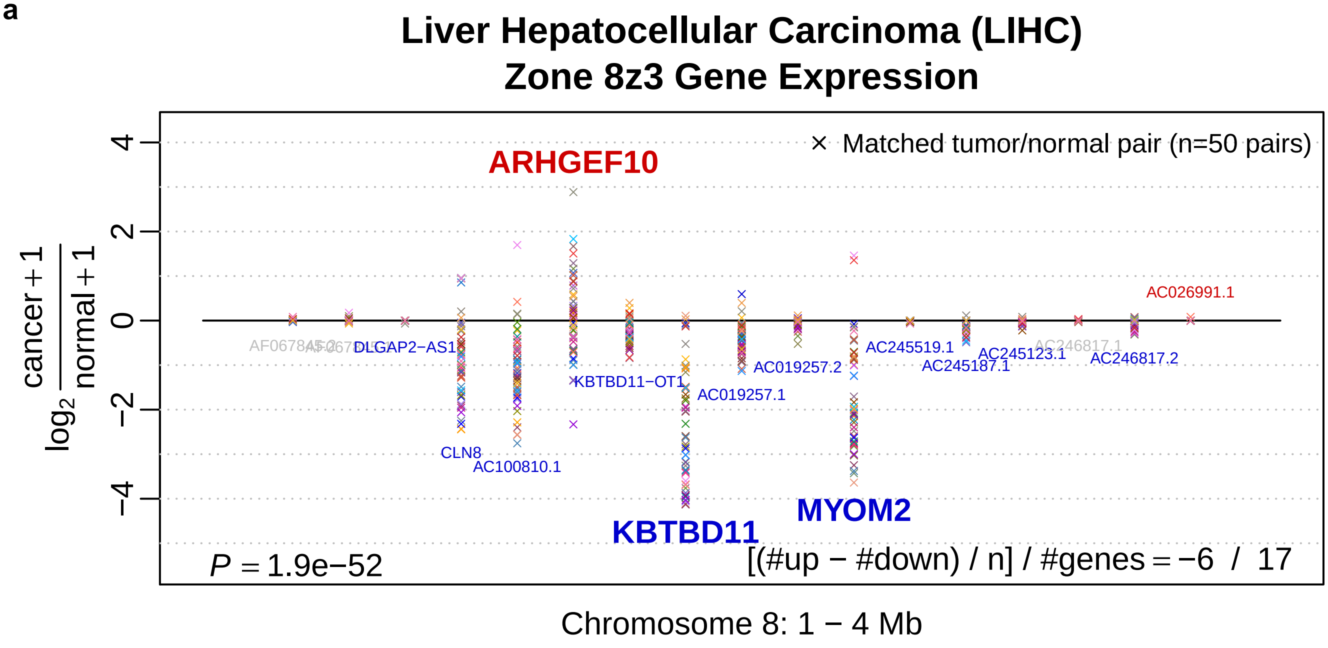

b

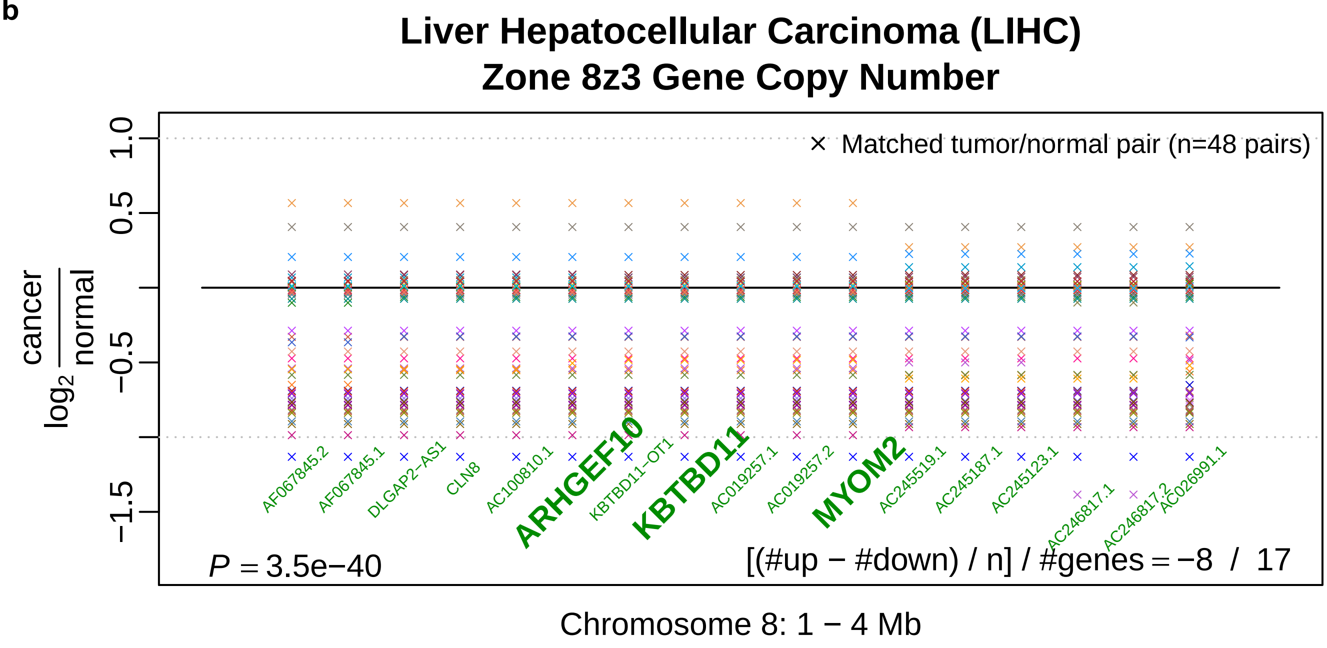

Supplementary Figure S3.78: The fifth most statistically significant polarized regulation zone in LIHC. a, The gene expression log ratio of cancer to normal for each gene within the zone in each patient. **b**, The somatic copy number log ratio of cancer to normal for each gene within the zone in each patient. See the full legend on page 3.

a

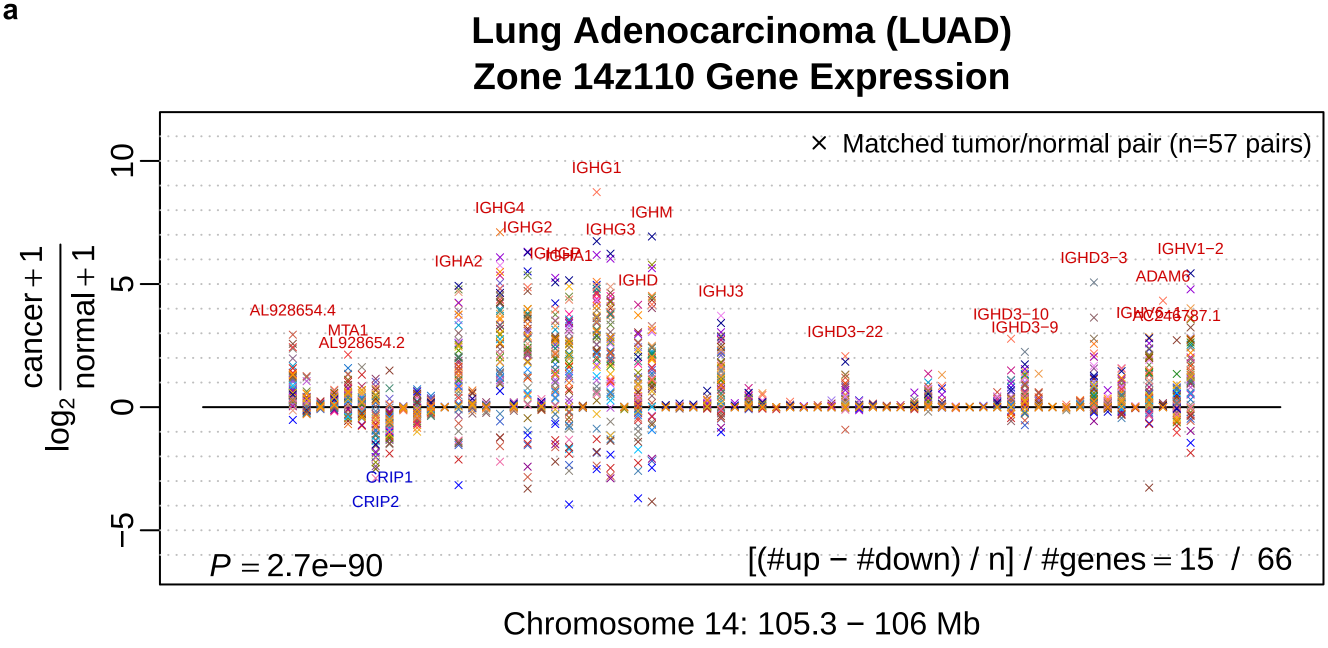

b

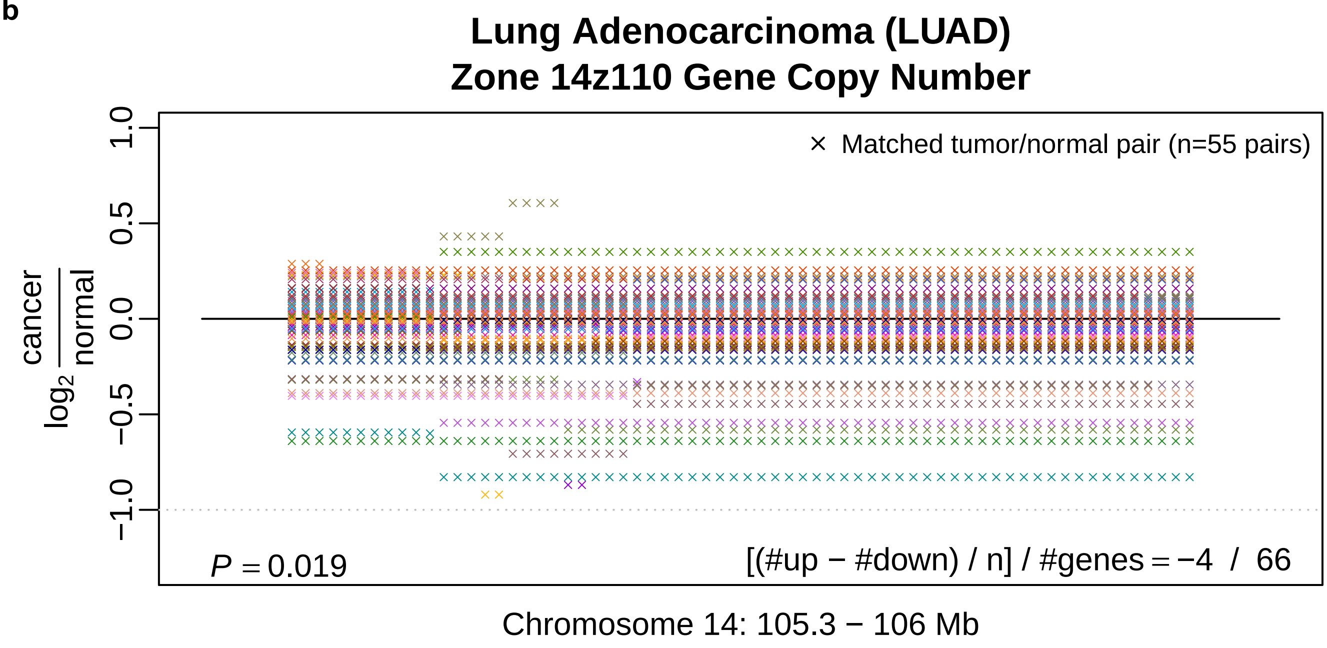

Supplementary Figure S3.79: The fifth most statistically significant polarized regulation zone in LUAD. a, The gene expression log ratio of cancer to normal for each gene within the zone in each patient. **b**, The somatic copy number log ratio of cancer to normal for each gene within the zone in each patient. See the full legend on page 3.

a

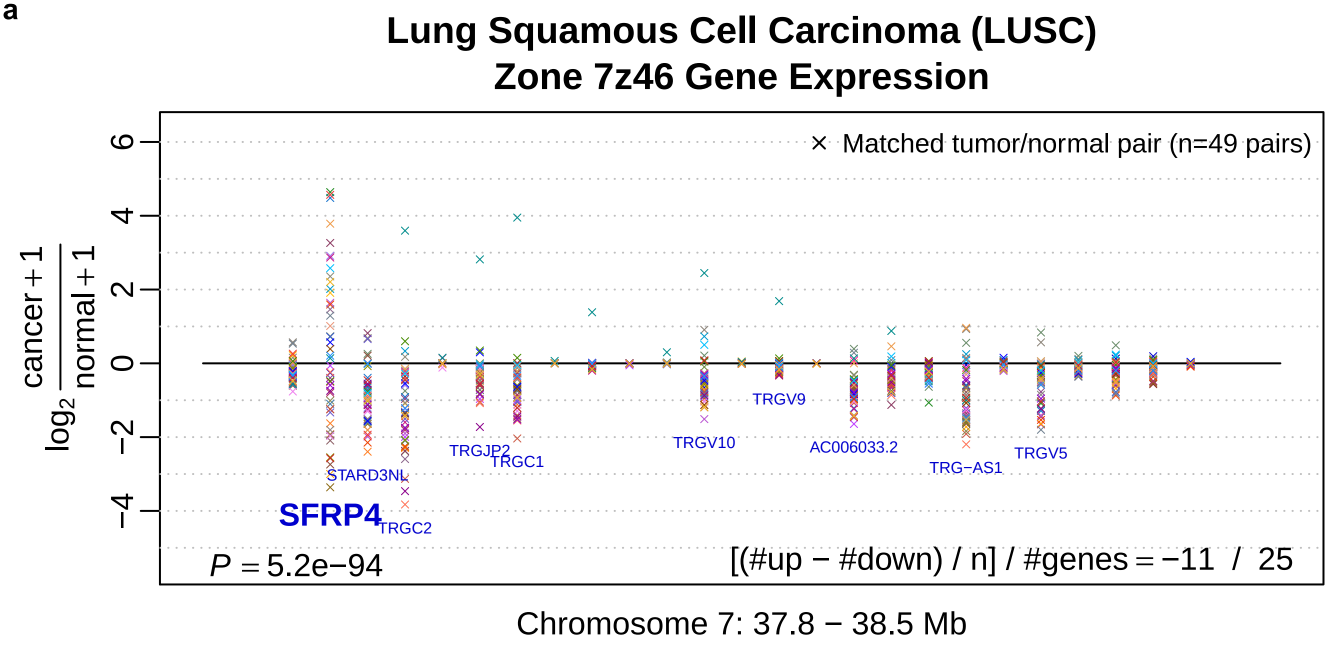

b

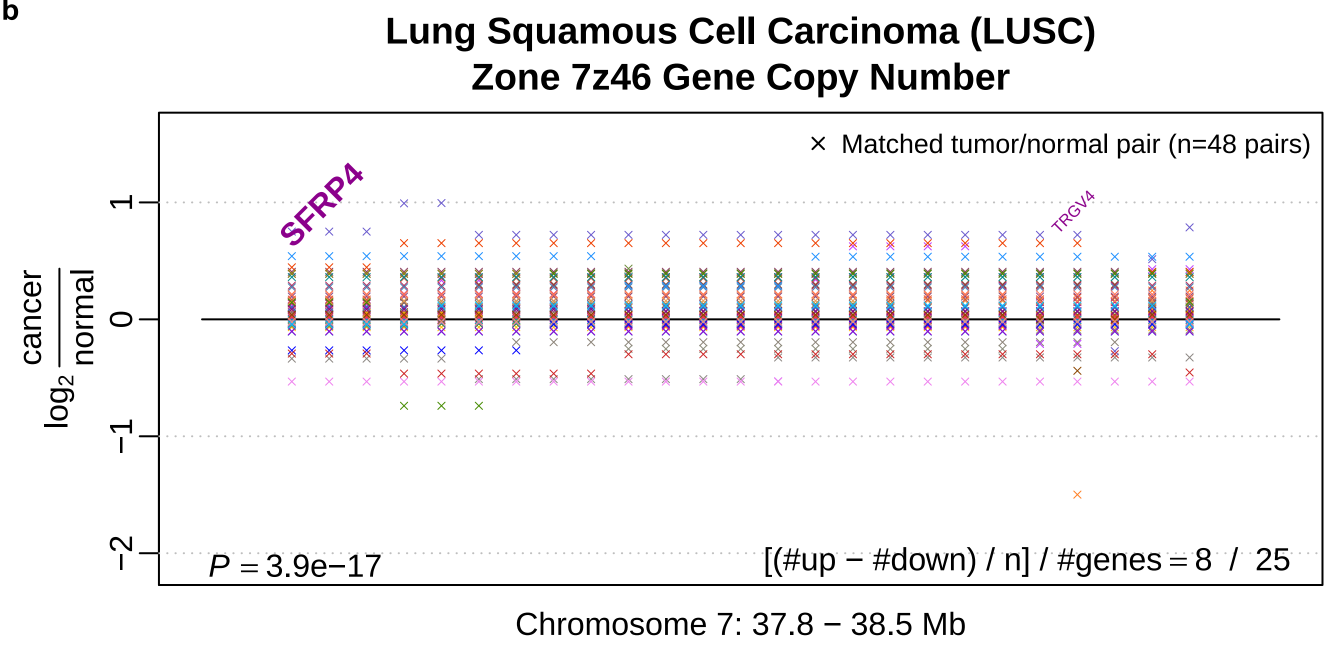

Supplementary Figure S3.80: The fifth most statistically significant polarized regulation zone in LUSC. a, The gene expression log ratio of cancer to normal for each gene within the zone in each patient. **b**, The somatic copy number log ratio of cancer to normal for each gene within the zone in each patient. See the full legend on page 3.

a

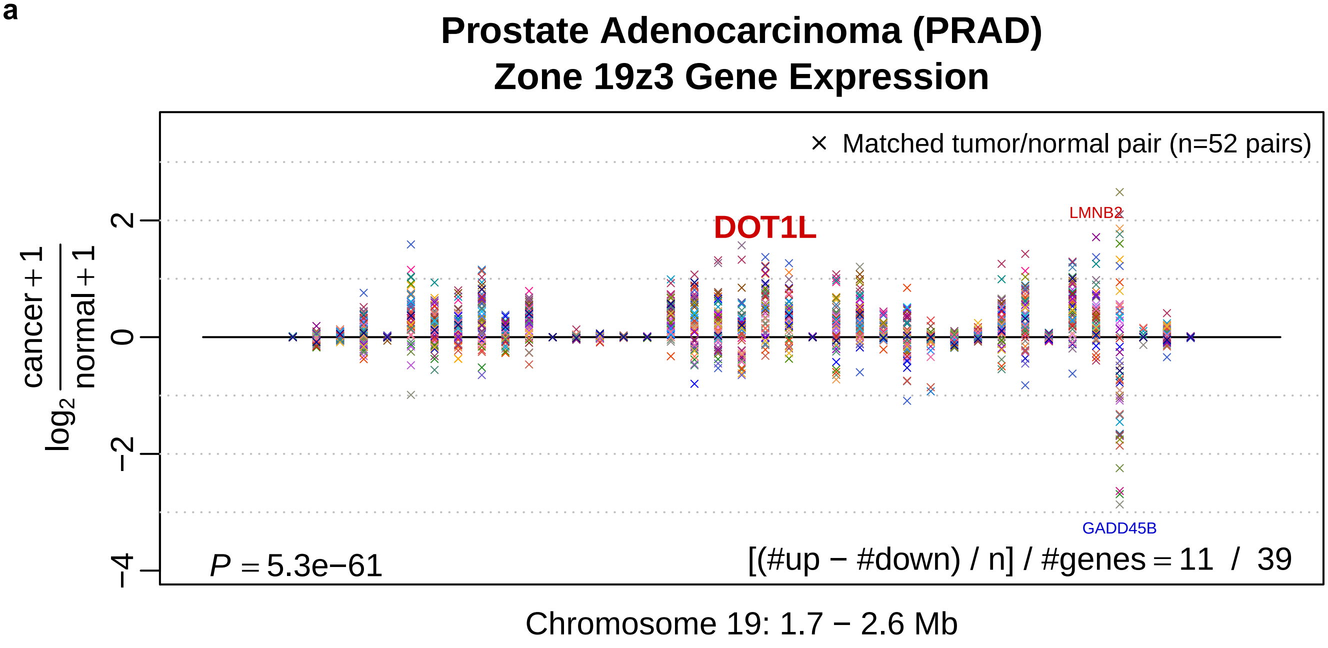

b

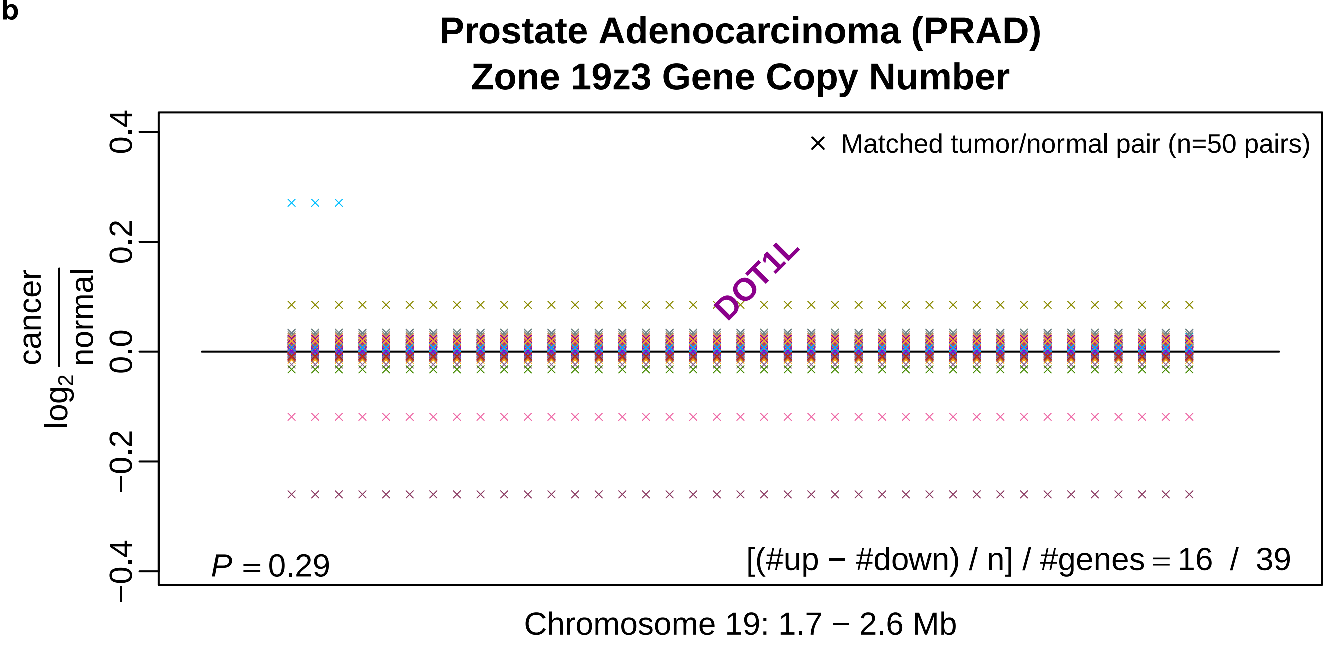

Supplementary Figure S3.81: The fifth most statistically significant polarized regulation zone in PRAD. a, The gene expression log ratio of cancer to normal for each gene within the zone in each patient. **b**, The somatic copy number log ratio of cancer to normal for each gene within the zone in each patient. See the full legend on page 3.

a

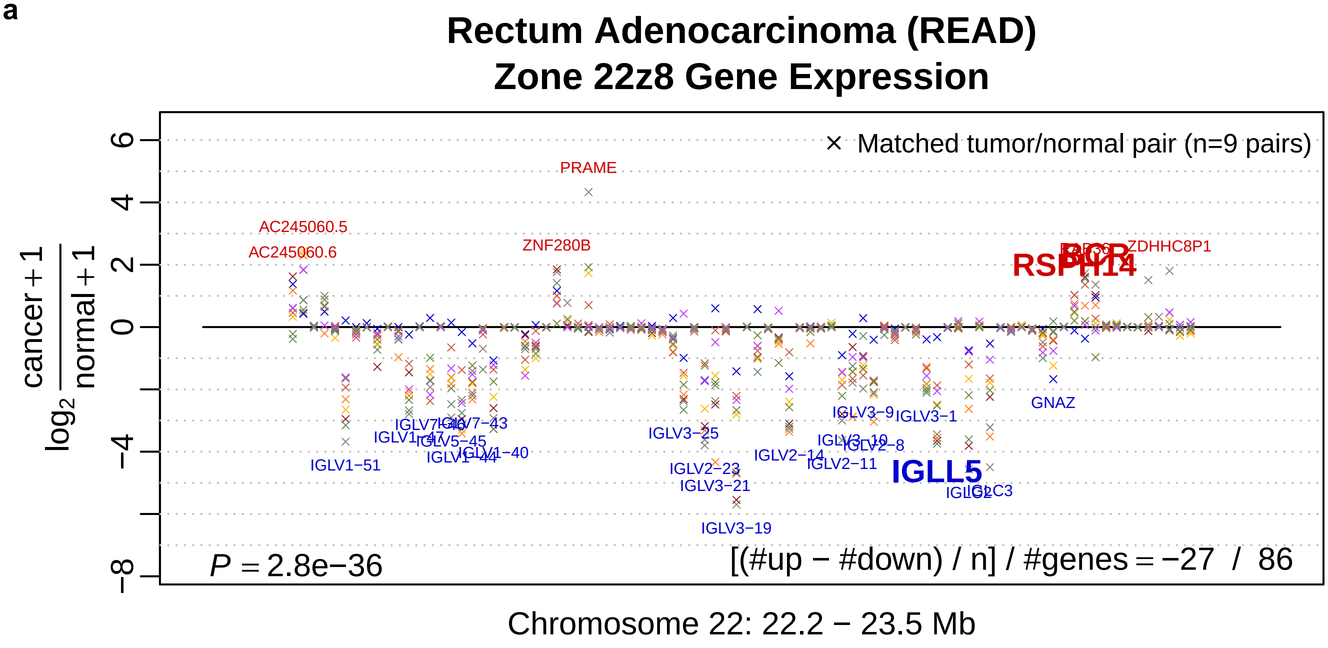

b

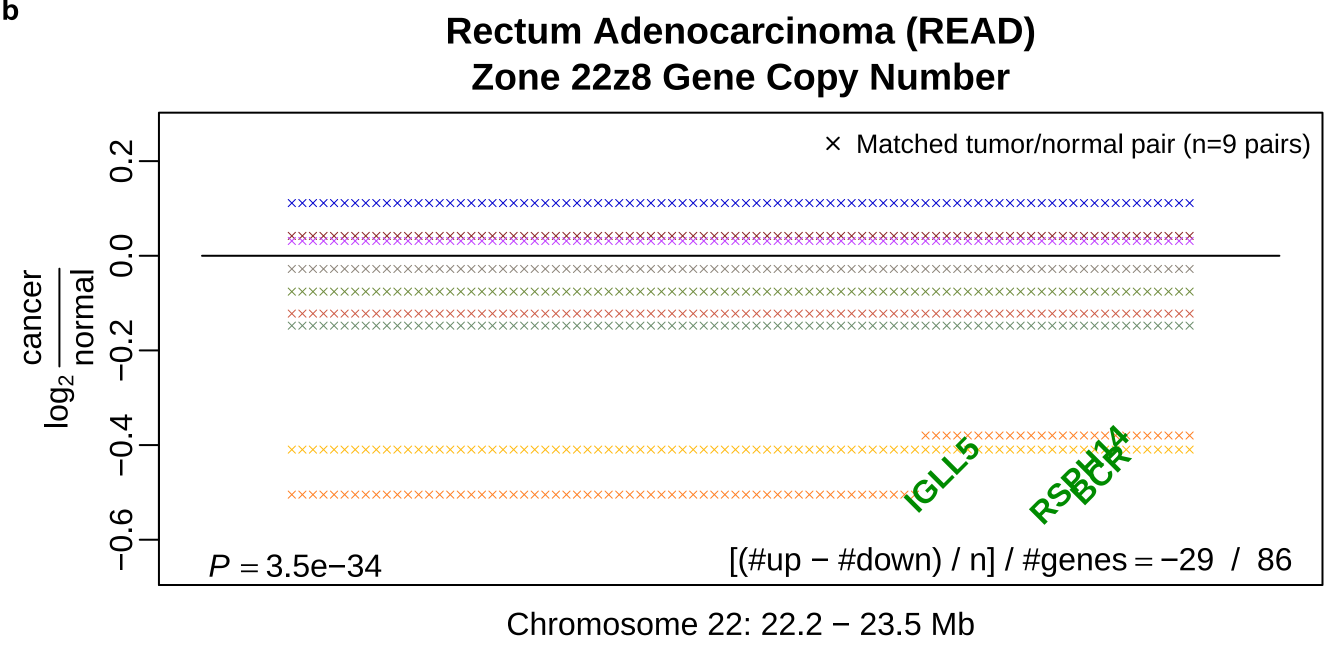

Supplementary Figure S3.82: The fifth most statistically significant polarized regulation zone in READ. a, The gene expression log ratio of cancer to normal for each gene within the zone in each patient. **b**, The somatic copy number log ratio of cancer to normal for each gene within the zone in each patient. See the full legend on page 3.

a

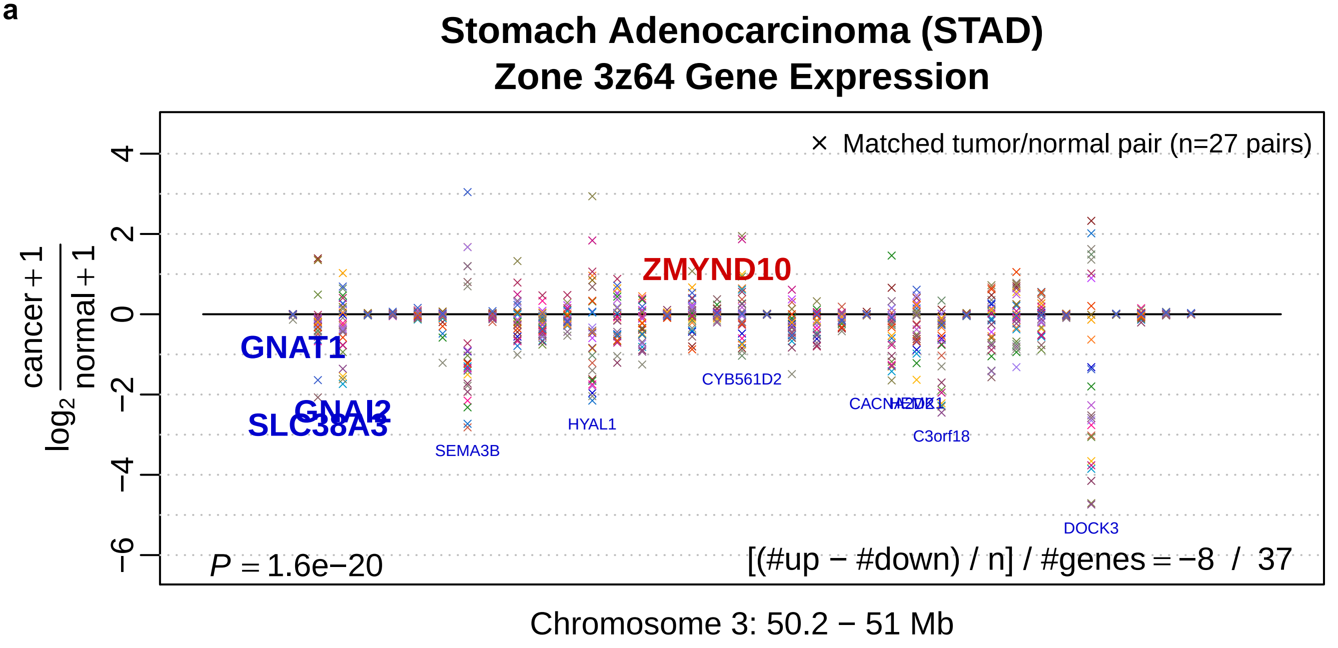

b

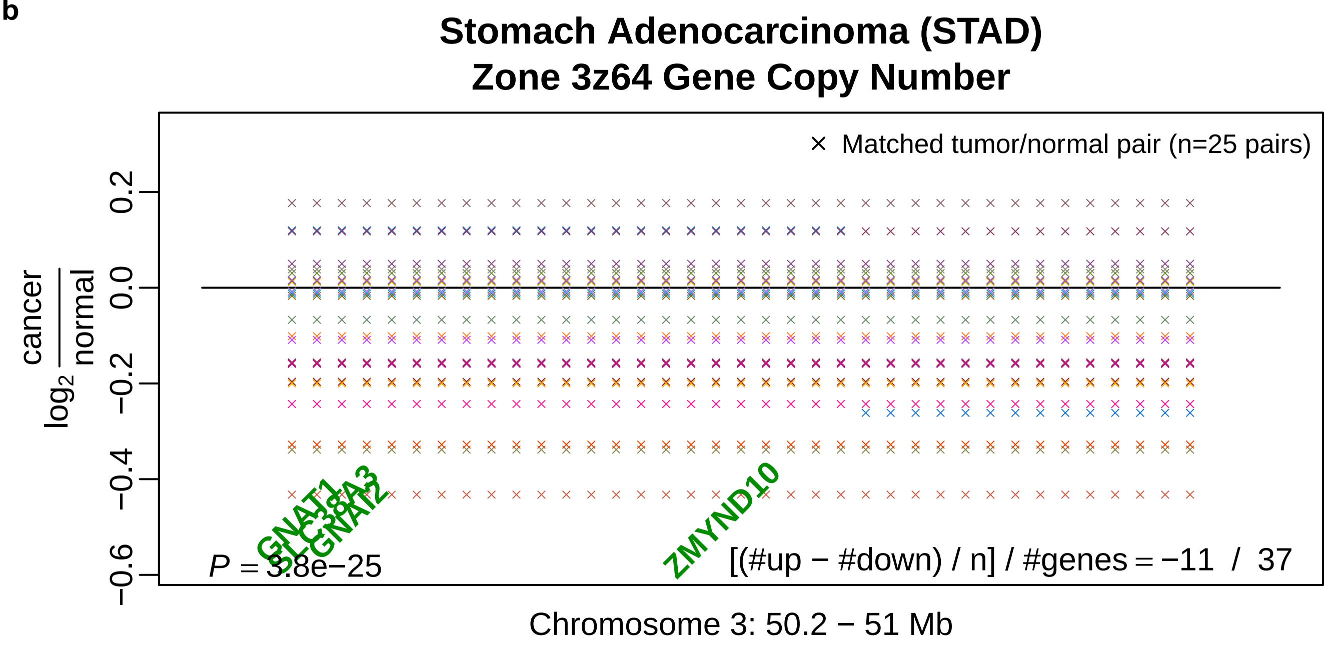

Supplementary Figure S3.83: The fifth most statistically significant polarized regulation zone in STAD. a, The gene expression log ratio of cancer to normal for each gene within the zone in each patient. **b**, The somatic copy number log ratio of cancer to normal for each gene within the zone in each patient. See the full legend on page 3.

a

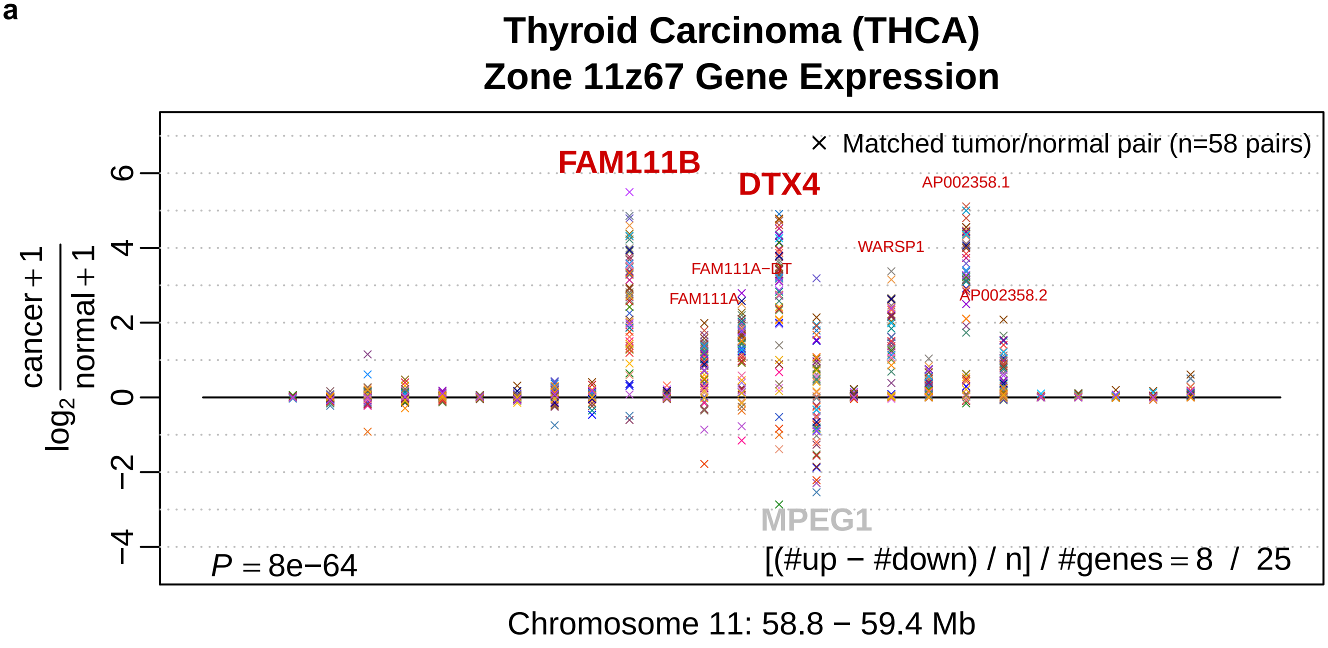

b

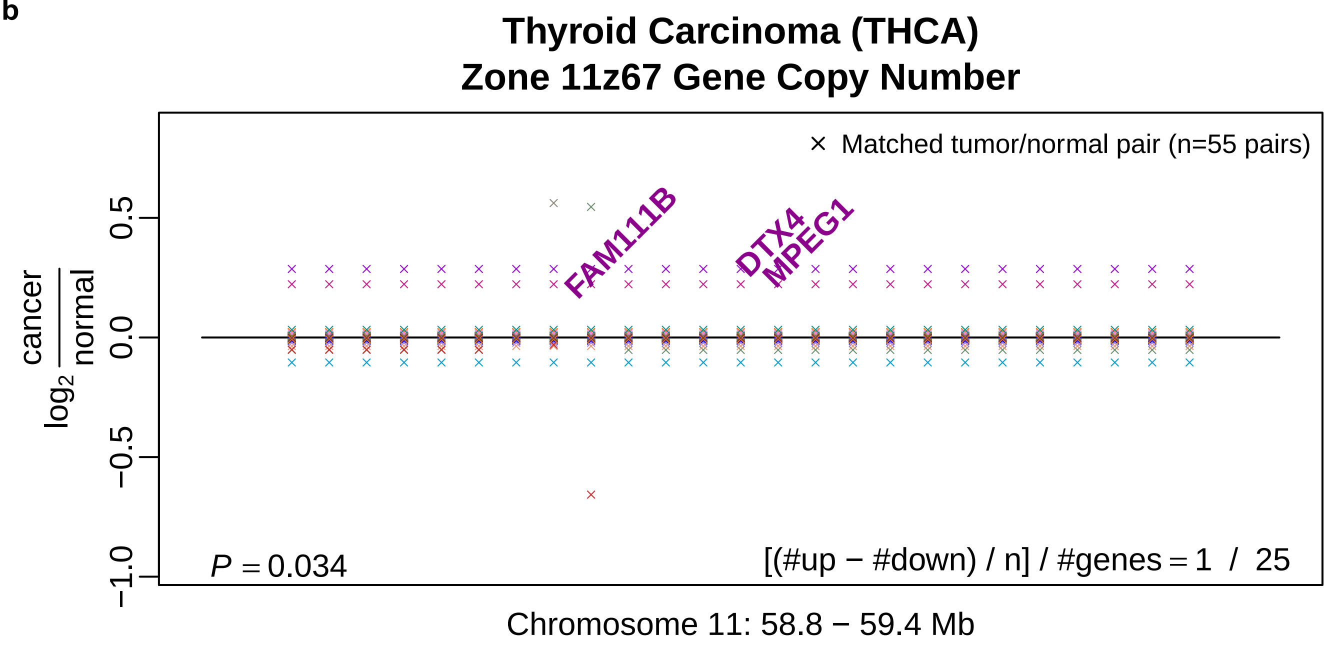

Supplementary Figure S3.84: The fifth most statistically significant polarized regulation zone in THCA. a, The gene expression log ratio of cancer to normal for each gene within the zone in each patient. **b**, The somatic copy number log ratio of cancer to normal for each gene within the zone in each patient. See the full legend on page 3.

a

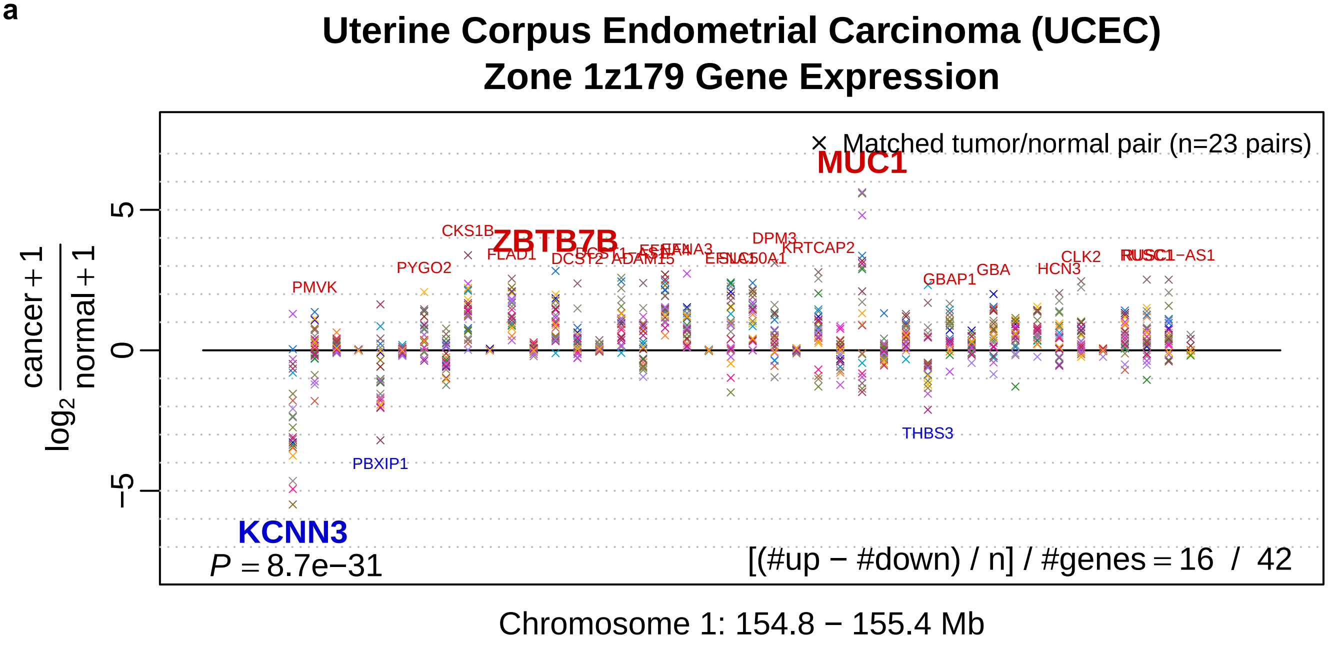

b

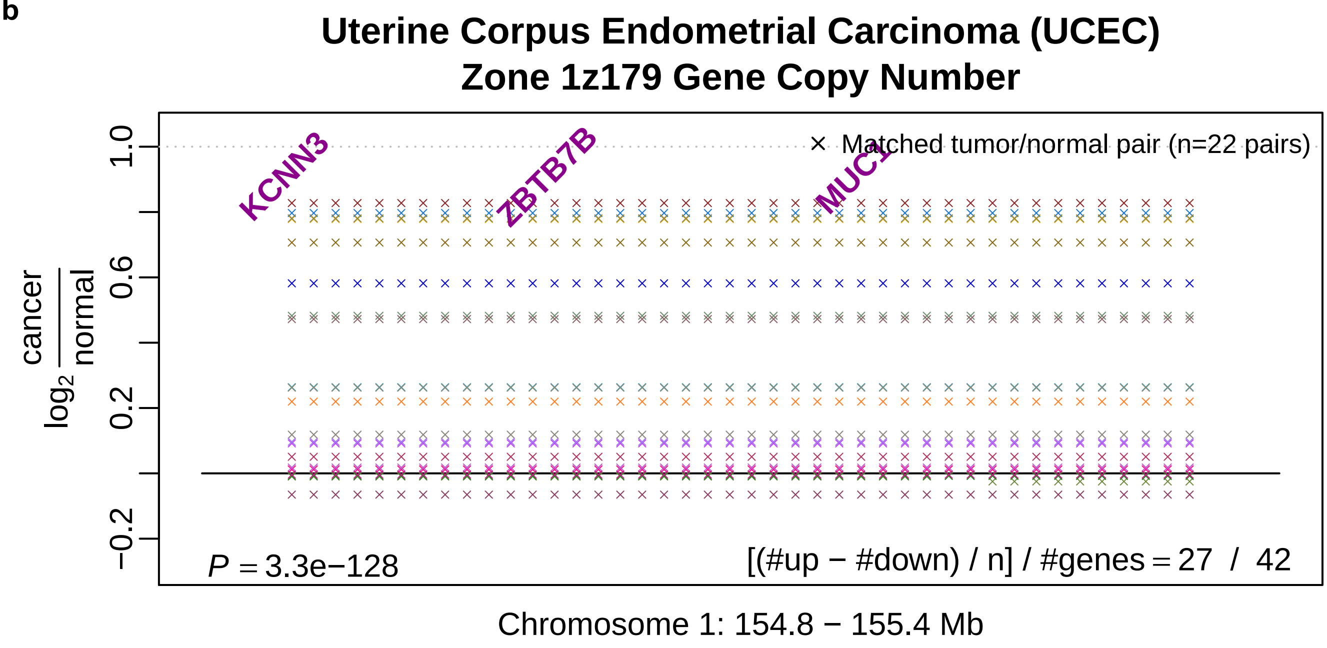

Supplementary Figure S3.85: The fifth most statistically significant polarized regulation zone in UCEC. a, The gene expression log ratio of cancer to normal for each gene within the zone in each patient. **b**, The somatic copy number log ratio of cancer to normal for each gene within the zone in each patient. See the full legend on page 3.
