# Supplementary material for: Efficient weighted univariate clustering maps outstanding dysregulated genomic zones in human cancers
Source: Bioinformatics. 2020 Jul 3;36(20):5027–36. doi: 10.1093/bioinformatics/btaa613 (PMC7755420; doi:10.1093/bioinformatics/btaa613)
Supplement: btaa613_Supplementary_Data [file btaa613_supplementary_data.zip › SuppNote-N1.pdf]

Supplementary Note N1: Optimal weighted univariate clustering is reproducible, fast, and scalable

The reproducibility of WUC and heuristic k-means is compared on clustering CpG sites for the MT genome in Figure N1.1. WUC returned identical results in all four runs, while heuristic k-means clusters visibly deviated from the optimal solution in different ways. For example, the locus at 5000 belongs to heuristic cluster 8, 11, 6, 7 in run 1, 2, 3, 4, respectively, while the optimal cluster of this locus is always 7. Here the leftmost cluster is indexed 1. Run 2 gave the worst clustering with a relative error of 90.9%. Although run 4 returned the correct cluster number at the locus, its overall clustering is still different from the optimal solution. Despite the MT genome containing only 435 CpG sites—a small data set, differences among the four heuristic runs are evident. Randomization in heuristic clustering to improve global optimality sacrificed reproducibility, while the deterministic WUC algorithm guarantees to reproduce.

Here we demonstrate on real data sets that it is now practical to solve large WUC problems. We compare WUC with the Hartigan-Wong algorithm, the default option of `kmeans()`. It uses a greedy strategy to repeatedly update the cluster assignment of each point. The large data set used is CpG sites along 25 human chromosomes (1–22, X, Y, and MT). A CpG site is a genomic coordinate on a chromosome where a cytosine (C) is followed immediately by a phosphate (p) and a guanine (G). Their clusters are called CpG islands. On human reference genome version GRCh38, we performed four runs on each chromosome at k=20: two runs with one restart and two runs with 20 restarts for heuristic k-means. Runtime statistics were obtained on an iMac (Mid 2010 model) with a 2.93GHz Intel Core i7 processor and 16GB 1333 MHz DDR3 memory. Figure N1.2 summarizes the runtime and relative errors for all 25 chromosomes for each of the four runs. The relative error is defined by the ratio of heuristic to minimal within-cluster SSQ minus 1. Heuristic k-means can finish faster than WUC but with high relative errors greater than 100%; when `nstart` was set to 20, relative errors were greatly reduced but it is ten times slower than WUC on an input of 2,500,000 points.

Next, we compare the runtime of the methods as functions of sample size n and number of clusters k. We include the quadratic (Wang and Song, 2011), log-linear, and linear time solutions for WUC, all implemented in package ‘Ckmeans.1d.dp’ and the Hartigan-Wong’s k-means algorithm. The relative error of heuristic k-means grows as k increases (Fig N1.3). With only one restart, the relative error of heuristic k-means can go beyond 100%; with 20 restarts, the relative error can still reach 10% and deteriorates as k increases. The runtime of heuristic k-means grows roughly exponentially to achieve a relative error of less than 5% due to a large number of restarts (Fig N1.4). Log-linear or linear WUC dramatically sped up the quadratic-time WUC (Fig N1.5). Figure N1.6 shows the runtime of log-linear, linear, and heuristic methods as a function of k averaged over multiple runs. The heuristic method with one restart converged to some solution quickly at a large k, but with no guarantee of globally optimality. The runtime advantage of the linear over log-linear solution is evidently announced at $k \geq 5$. Figure N1.7 compares the runtime of the methods as a function of n averaged over multiple runs at fixed k=5. At $n<300$, the log-linear solution is fastest due to its low overhead. At $500<n<3000$, all three methods used comparable time As n increases beyond 5000, the linear solution becomes the fastest and ran stably faster than log-linear or heuristic solutions.

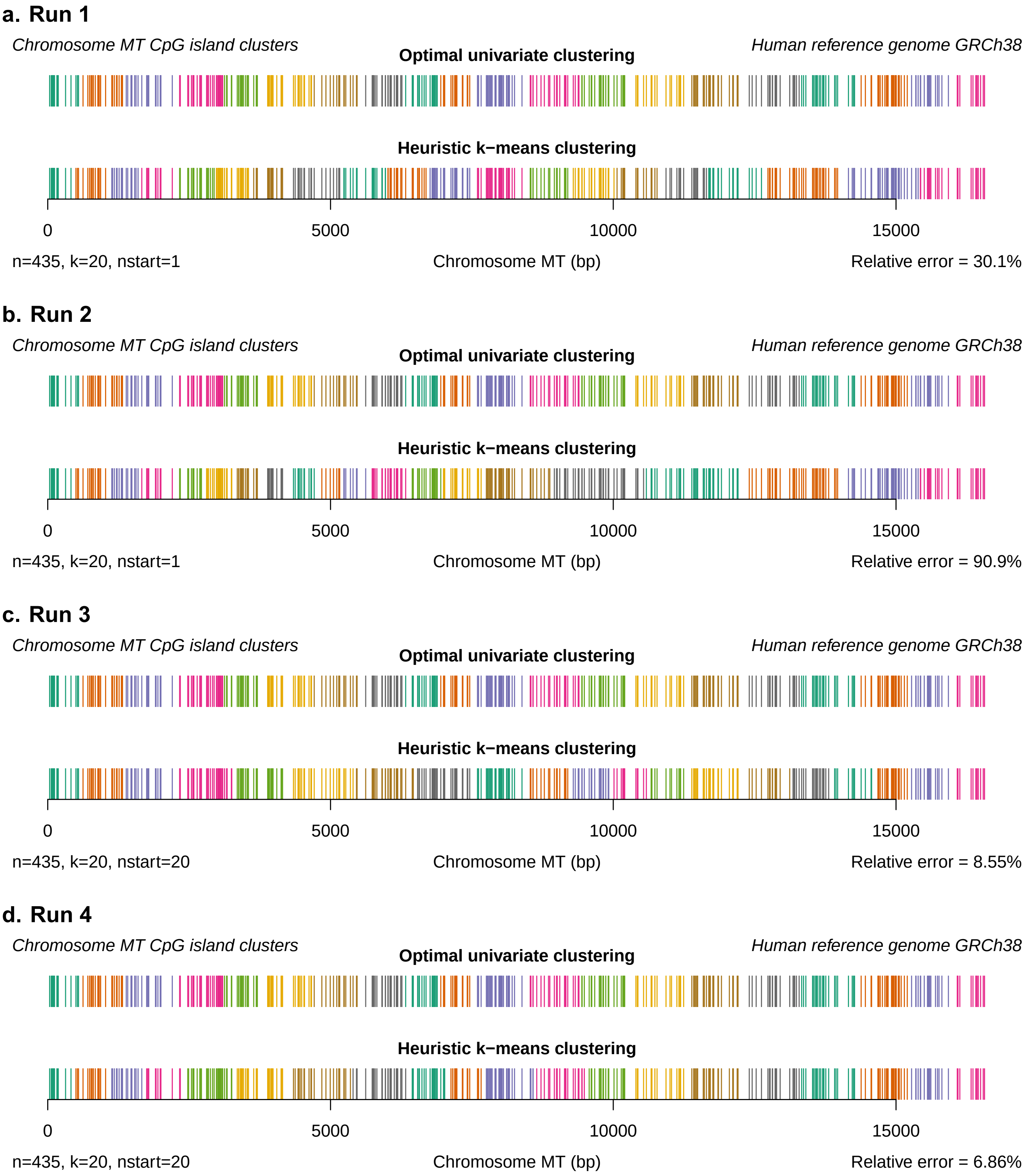

Figure N1.1: Reproducibility of optimal versus heuristic clustering of CpG sites along the human mitochondrial (MT) genome. The number of CpG clusters was set to $k = 20$. Clusters are shown using different colors and a color may be reused for nonconsecutive clusters. The optimal method produced identical clusterings in all four runs. Heuristic *k*-means using different numbers of restart generated visually distinct clusterings with a wide range of relative errors as marked in the plots. **a,** Run 1. nstart=1. **b,** Run 2. nstart=1. **c,** Run 3. nstart=20. **d,** Run 4. nstart=20.

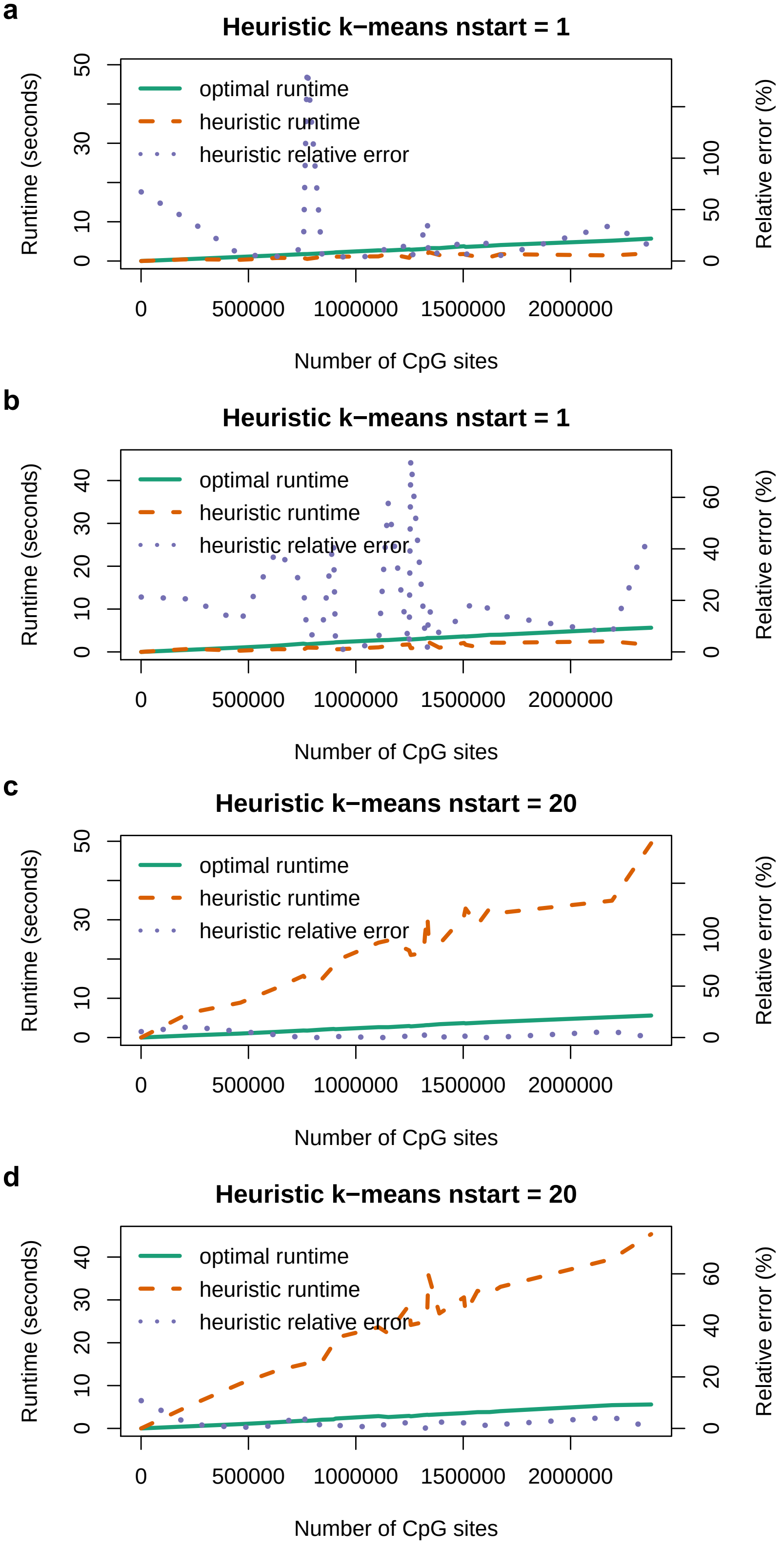

Figure N1.2: Runtime and relative errors for CpG site clustering along all human chromosomes. DNA sequences of human chromosome 1–22, X, Y, and mitochondrion were obtained from human reference genome version GRCh38. Runtime was measured for cluster analysis on each chromosome. The number of clusters was set to $k = 20$. **a,** Run 1. nstart=1. **b,** Run 2. nstart=1. **c,** Run 3. nstart=20. **d,** Run 4. nstart=20.

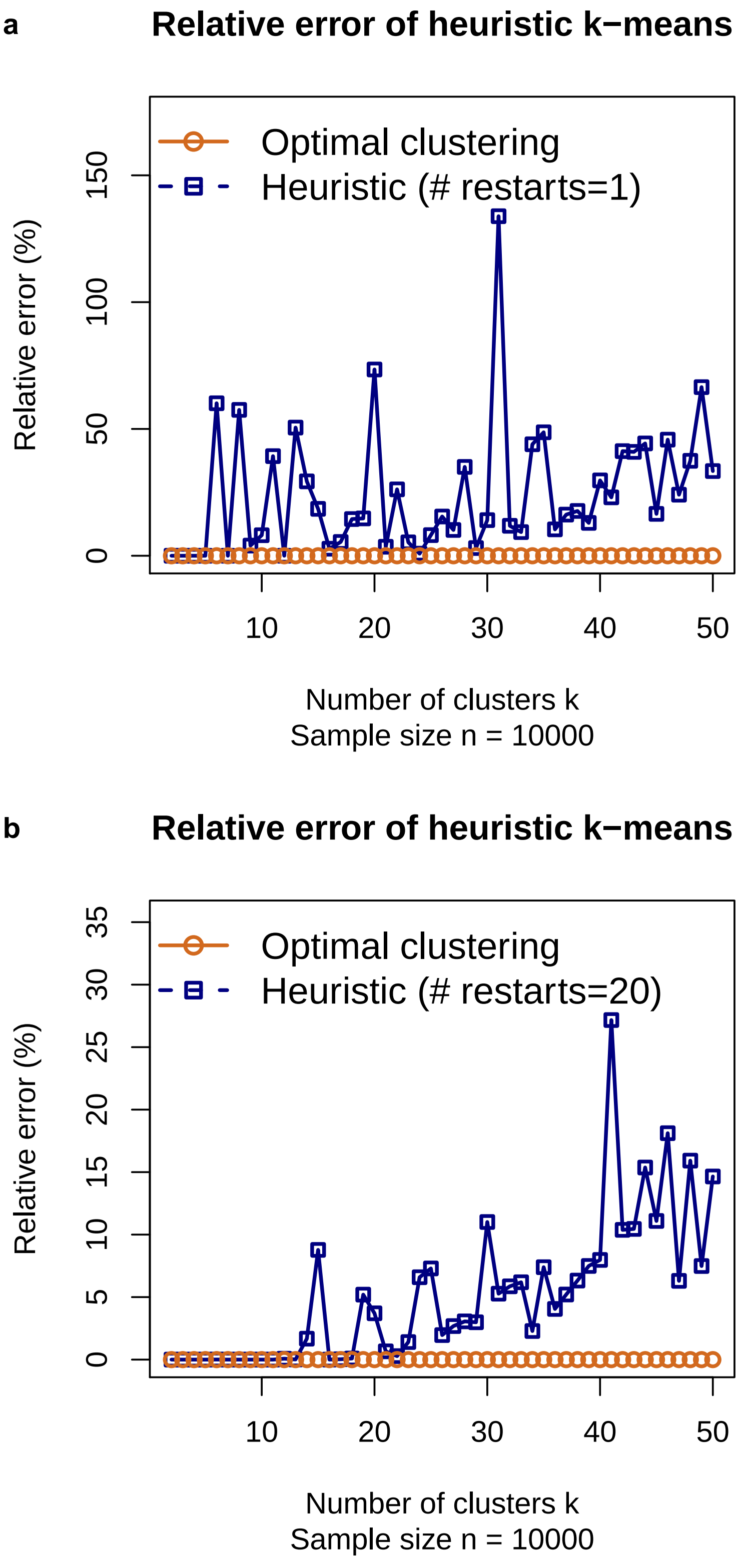

Figure N1.3: Relative errors of heuristic *k*-means solutions grow as the number of clusters increases. Optimal clustering solutions always achieve zero relative errors as the global minimum is guaranteed. **a,** The number of restart for heuristic *k*-means is 1. At $k = 6$, the relative error is about 60%; the maximum relative error of 140% occurs at $k = 31$. **b,** The number of restart for heuristic *k*-means is 20. The relative errors are less than one restart, but the increasing trend of errors is stronger. The maximum relative error can still be as high as 28% at $k = 41$.

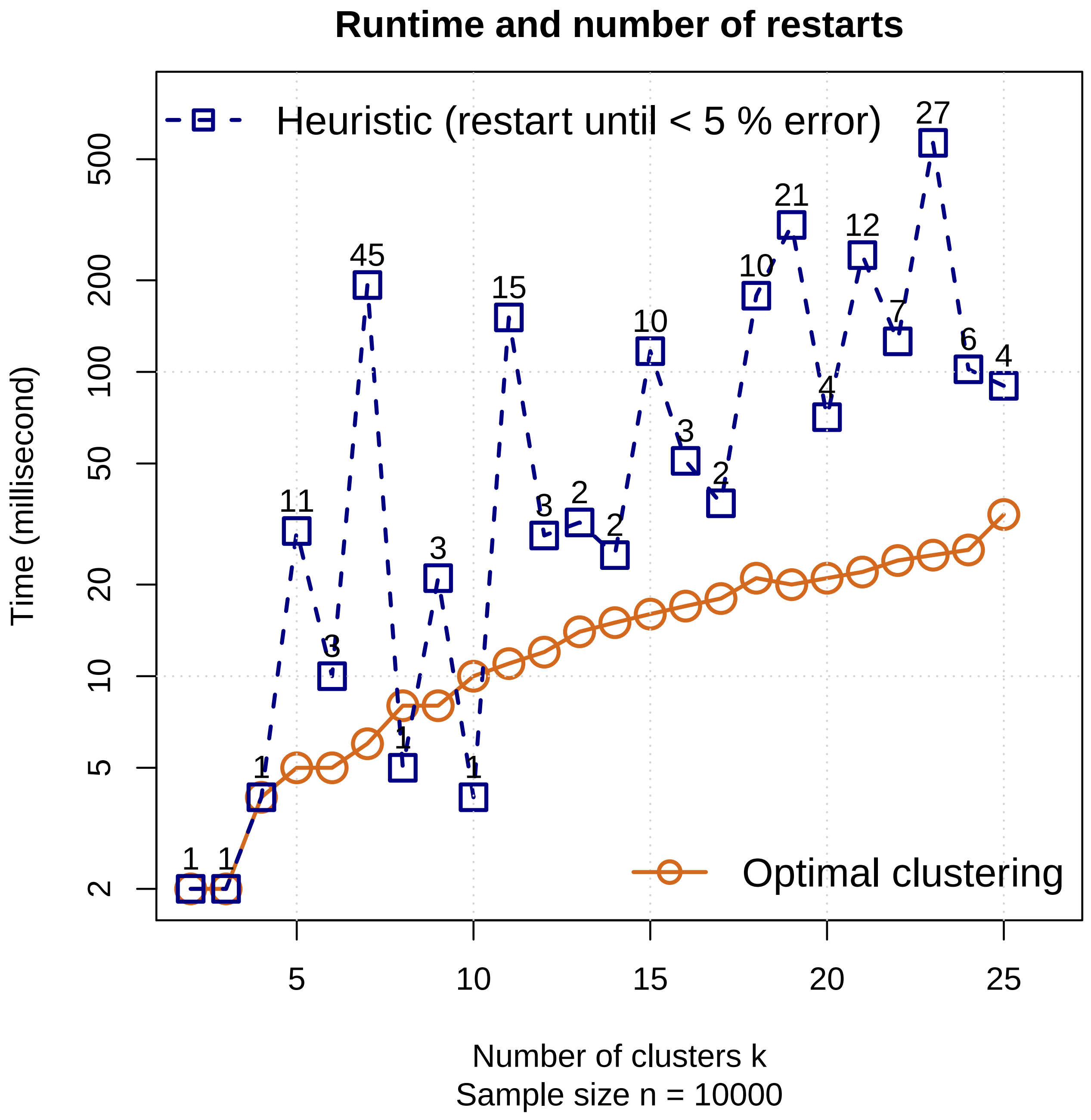

Figure N1.4: Empirical runtime for heuristic *k*-means clustering to achieve $<$5% relative error grows exponentially. The numbers of restarts for the heuristic *k*-means are marked on top of each runtime point. The runtime of the linear-time optimal clustering method increases about 1 millisecond when *k* increases by 1. The data were generated by sampling from Gaussian mixture models with increasing number of Gaussian components. The sample size is fixed to 10,000.

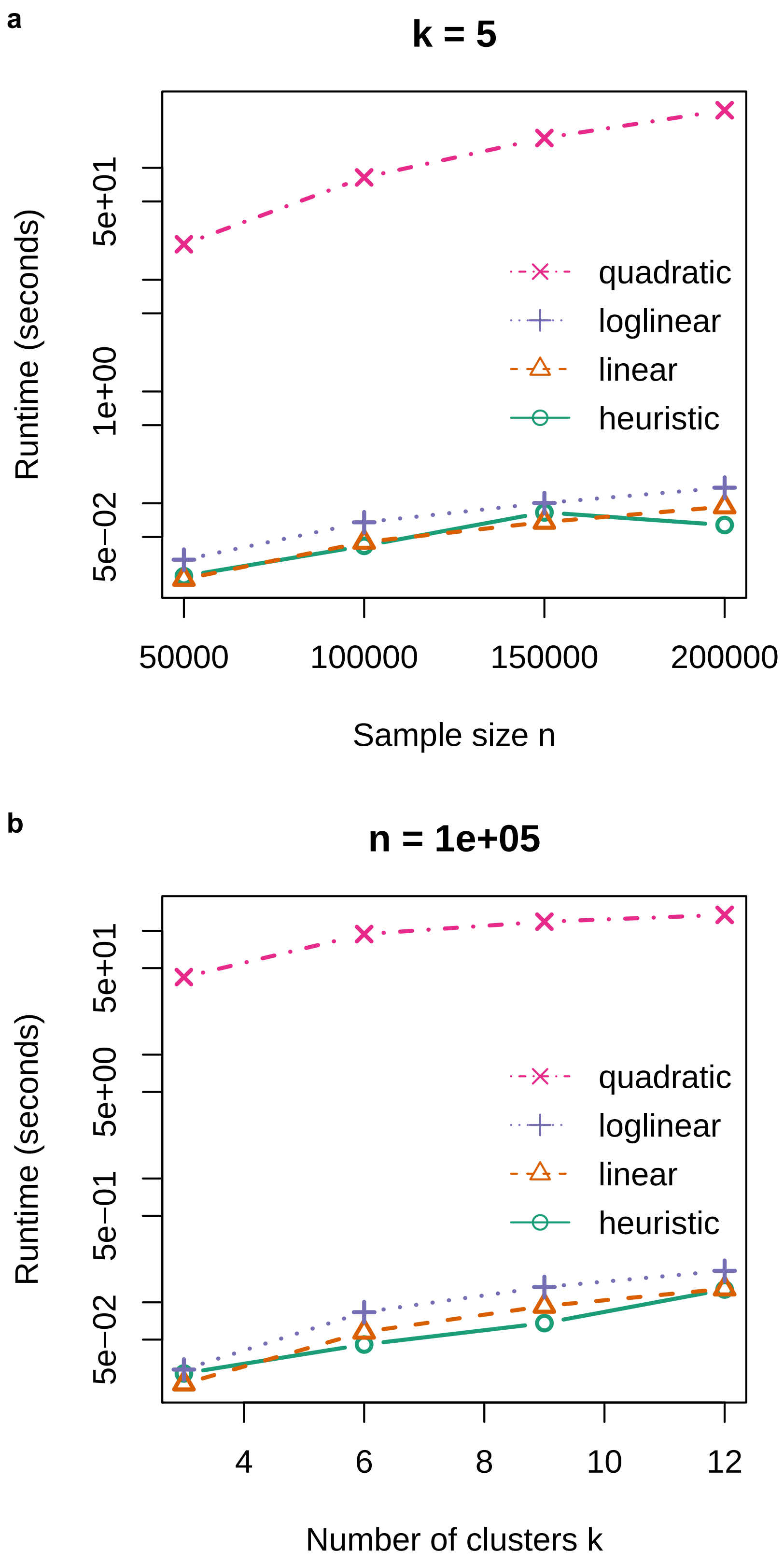

Figure N1.5: Runtime reduction over the optimal quadratic dynamic programming algorithm for univariate clustering. Each runtime value is an average of three runs. Three implementations (quadratic, loglinear, and linear) of optimal univariate clustering and heuristic *k*-mean are included. Heuristic *k*-means with one restart was fast but produces low quality clustering. **a,** Runtime as a function of sample size *n*. The number of clusters is fixed to $k = 5$. **b,** Runtime as a function of number of clusters *k*. The sample size is fixed to $n = 100,000$.

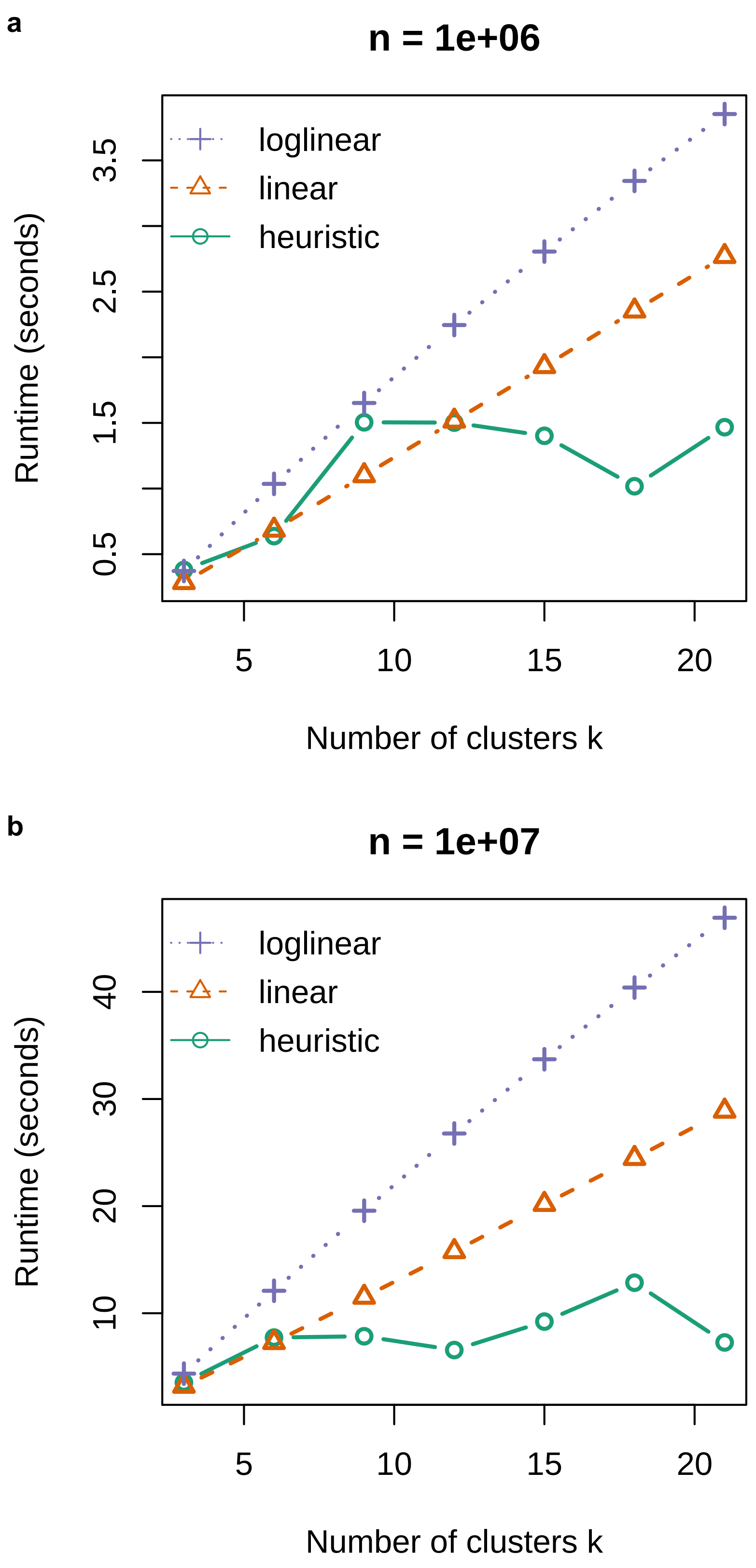

Figure N1.6: Runtime as a function of the number of clusters for three methods. Two implementations (loglinear and linear) of optimal univariate clustering are included. Each runtime value is an average of three runs. Heuristic k-means restart was set to 1. For large k values, heuristic k-means is fast with one restart but the clustering result can be of very low quality as k increases. The sample size was fixed at **a,** 1,000,000 and **b,** 10,000,000.

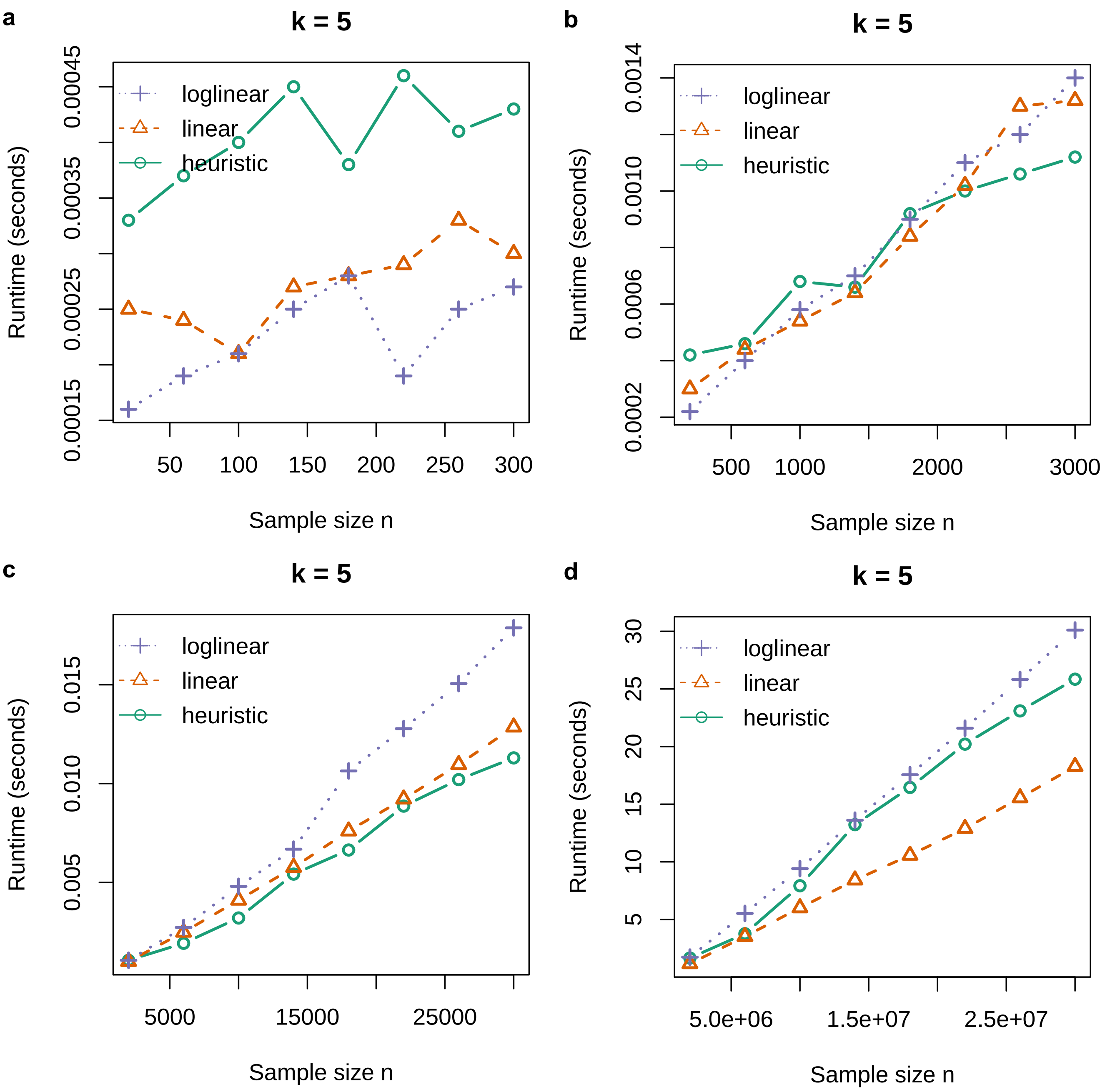

Figure N1.7: The runtime advantage of linear-time optimal clustering method becomes pronounced as the sample size *n* increases. The runtime as a function of input size is shown for three methods. Each runtime value is an average of three runs. The number of clusters is fixed to $k = 5$. Heuristic *k*-means restart was set to 1. The ranges of sample size grow geometrically in **a,** 50–300, **b,** 500–3,000, **c,** 5,000–30,000, and **d,** 5,000,000–30,000,000. In all cases, the linear-time solution is nearly the fastest for smaller sample sizes and the fastest for very large sample size.

References

Wang, H. and Song, M. (2011). Ckmeans.1d.dp: optimal k-means clustering in one dimension by dynamic programming. *The R Journal*, 3(2):29–33.
