# Supplementary material for: Efficient weighted univariate clustering maps outstanding dysregulated genomic zones in human cancers
Source: Bioinformatics. 2020 Jul 3;36(20):5027–36. doi: 10.1093/bioinformatics/btaa613 (PMC7755420; doi:10.1093/bioinformatics/btaa613)
Supplement: btaa613_Supplementary_Data [file btaa613_supplementary_data.zip › SuppNote-N2.pdf]

Supplementary Note N2: Statistical and biological relevance of detected dysregulated genomic maps in human cancers

Contents

N2.1 Numbers of outstanding genomic zones in each cancer type are higher than those expected by chance N2-1

N2.2 Boundaries of genomic zones are robust to sample variation in the TCGA collection N2-4

N2.3 Outstanding genomic zones in cancer are enriched with genetic and epigenetic signals in human genome N2-5

N2.4 The number of polarity conserved loci across cancer types is statistically significantly higher than what is expected by chance N2-8

For each cancer type, a genomic zone is a chromosomal interval with a start and an end genomic coordinate determined by expression-level weighted clustering of gene locations. Some genomic zones are further declared outstanding by the Pearson's chi-squared test based on the numbers of positively and negatively expressed genes. The polarity of a zone is positive/negative if the number of positively/negatively expressed genes dominates. Details were described in the Methods section of the main text.

Here, we perform four additional analyses to establish the statistical and biological relevance of the dysregulated genomic maps we have detected for all 17 cancer types. On the human cancer genomic maps defined by chromosomal start and end coordinates of each zone and their polarity signs, the four analyses provide the statistical significance of the total number of genomic zones for each cancer type, the robustness of the zone boundaries, the enrichment of genomic and epigenomic signals in outstanding zones, the statistical significance of the number of dysregulated loci conserved in all cancer types.

N2.1 Numbers of outstanding genomic zones in each cancer type are higher than those expected by chance

As genomic zone boundaries are determined by univariate clustering of gene positions weighted by their expression levels in pooled cancer and normal samples, it is not obvious whether the zone boundaries are effective in locking in clusters of genes of same polarity in cancer versus normal samples. An outstanding genomic zone is either positively or negatively polarized in cancer samples over matched normal samples at a statistical significance level of $P \leq 0.05$. Specifically, we test for each cancer type whether the number of outstanding genomic zones is different from what is expected by chance if the original genomic zones are randomly distributed along a chromosome.

For each cancer type, the input is the genomic zone boundaries obtained by weighted clustering of genes along each chromosome and the gene expression levels in all matched pair samples. We calculate the total number of zones—the observed test statistic—that are declared outstanding on the original zone boundaries using the gene expression data. The P-value that declares an outstanding zone is determined by Pearson's chi-squared test on the number of either positively or negatively expressed genes within the zone versus outside the zone, as described in the Methods section of the main text. The output is the test statistic—the number of outstanding genomic zone, its null distribution, and P-value. We use permutation test to obtain the null distribution and the P-value associated with the test statistic.

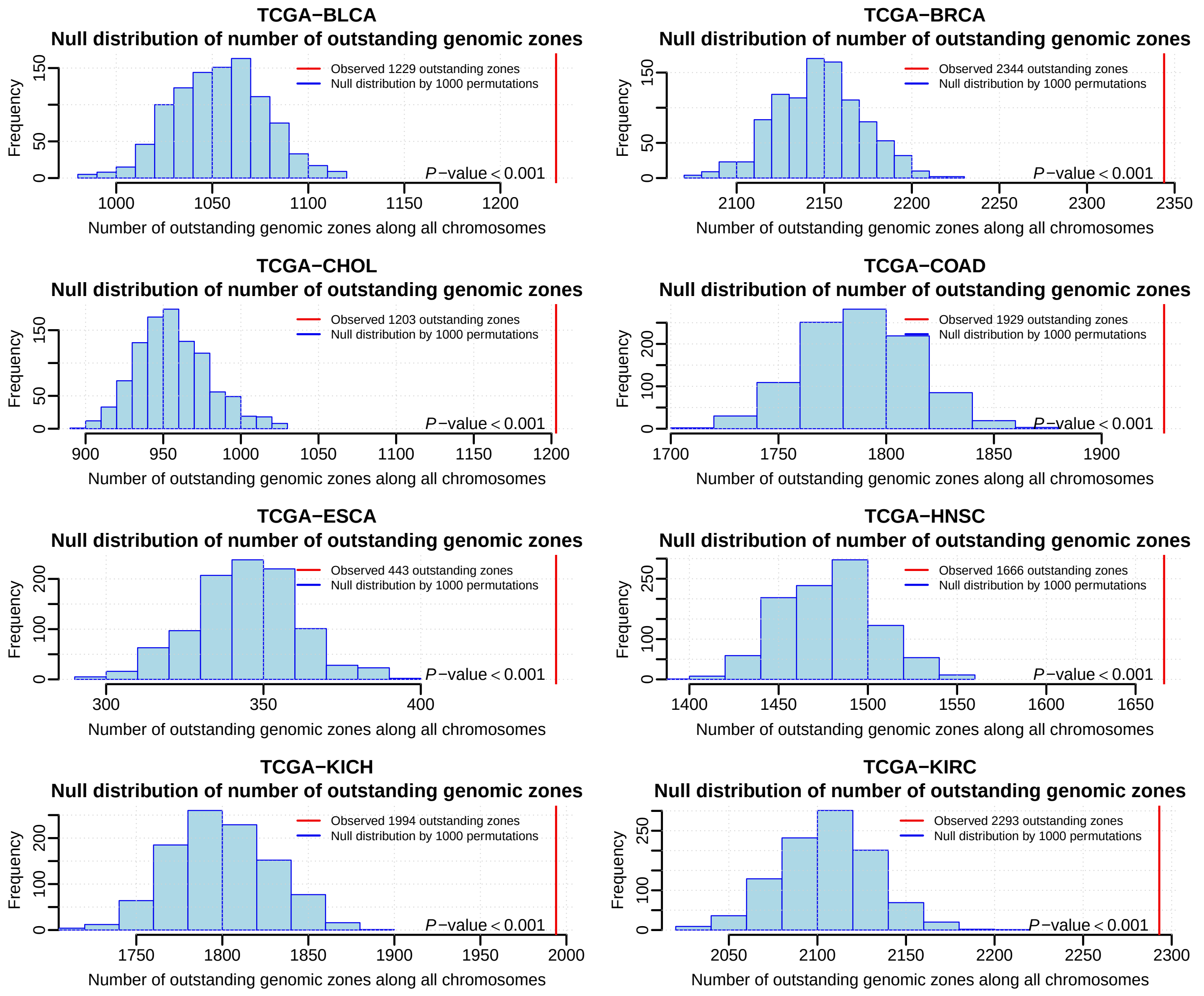

Figure N2.1: Statistical significance and null distributions of numbers of outstanding genomic zones for the cancer types of BLCA, BRCA, CHOL, COAD, ESCA, HNSC, KICH, and KIRC. Each null distribution is obtained after 1000 permutations of the original zones within each chromosome.

For each cancer type, we created from the original zone boundaries 1000 sets of permuted zones along a chromosome. Each set contains the same number of zones with the same zone widths, but the zones are arranged in a random order along the chromosome. Gene expression data are not permuted. For each set of zones, we compute a test statistic. The collection of test statistic constitutes the null distribution. The P-value is finally computed by the number of null test statistics greater than or equal to the observed test statistic.

For each of the 17 cancer types, the null distributions and statistical significance of the number of outstanding zones are shown in Figures N2.1 and N2.2. For each and every cancer type, its number of outstanding zones is statistically significantly (P-value < 0.01) higher than values in the null distributions. These results thus suggest that the genomic zones obtained by weighted univariate clustering indeed adapted to differential gene expression in cancer versus the matched normal samples.

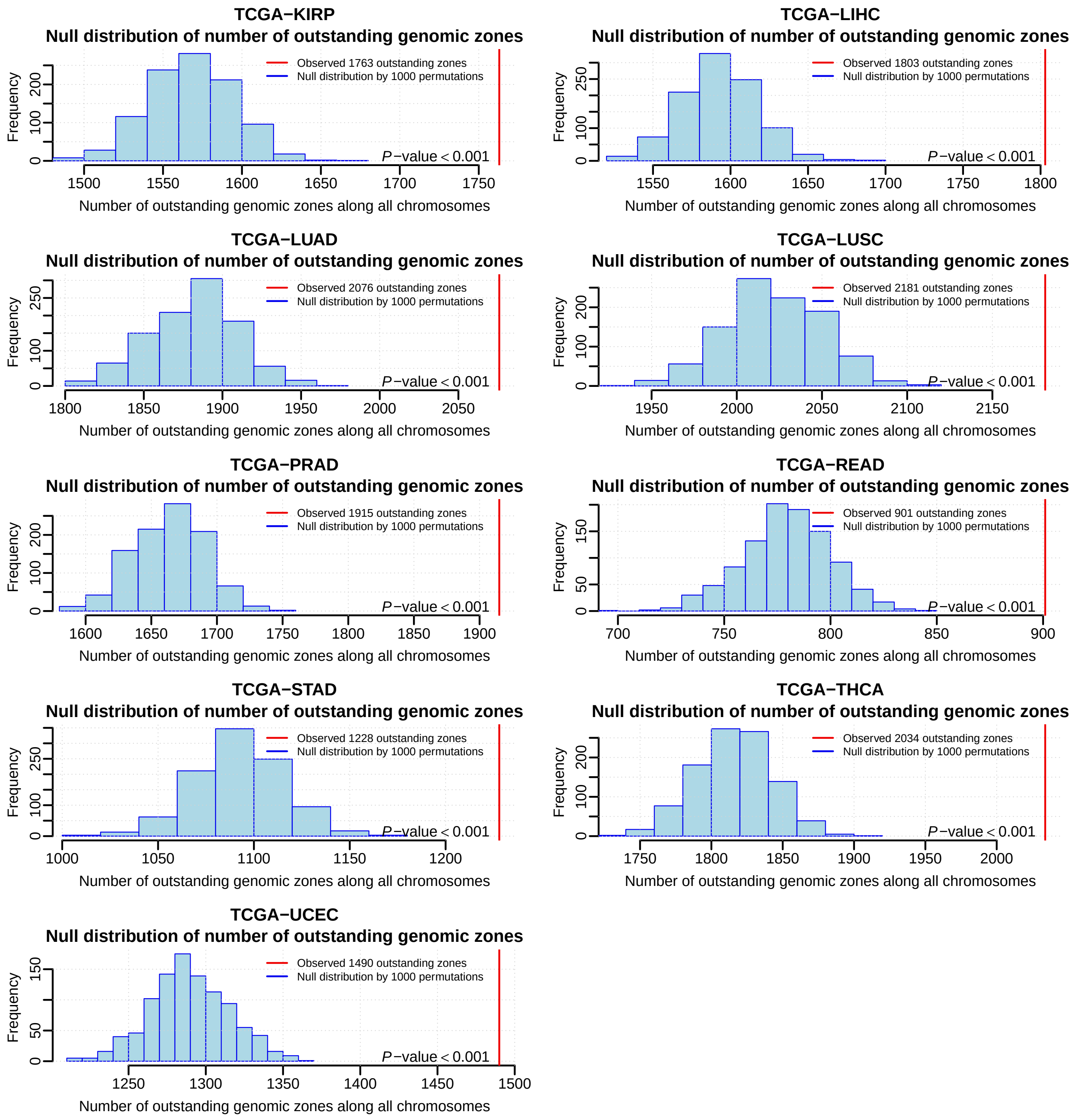

Figure N2.2: Statistical significance and null distributions of numbers of outstanding genomic zones for the cancer types of KIRP, LIHC, LUAD, LUSC, PRAD, READ, STAD, THCA, and UCEC. Each null distribution is obtained after 1000 permutations of the original zones within each chromosome.

N2.2 Boundaries of genomic zones are robust to sample variation in the TCGA collection

To examine the robustness of zones obtained by weighted univariate clustering and cancer transcriptomic data, we performed bootstrapping to study the influence of sample variation on zone boundaries. We selected three cancer types—BRCA, COAD, ESCA—to represent studies of the maximum, median, and minimum sample sizes, respectively. We chose chromosome 12 to represent the average chromosomal length. Clustering of gene positions was weighted by the combined normal and cancer patient gene expression data along chromosome 12.

Figure N2.3 shows evidence to support the robustness of the approach on the cancer transcriptomic data. We performed five bootstrapping for each data set. The total numbers of zones estimated varied only slightly 153–154 for BRCA, 150–154 for COAD, and 155–156 for ESCA. Bootstrapped zone boundaries show greater coincidence with the original when the sample size is larger. Most identical boundaries (135) between bootstrapped and original sample are found for BRCA with the largest sample size; least identical boundaries (109) are returned for ESCA with the smallest sample size.

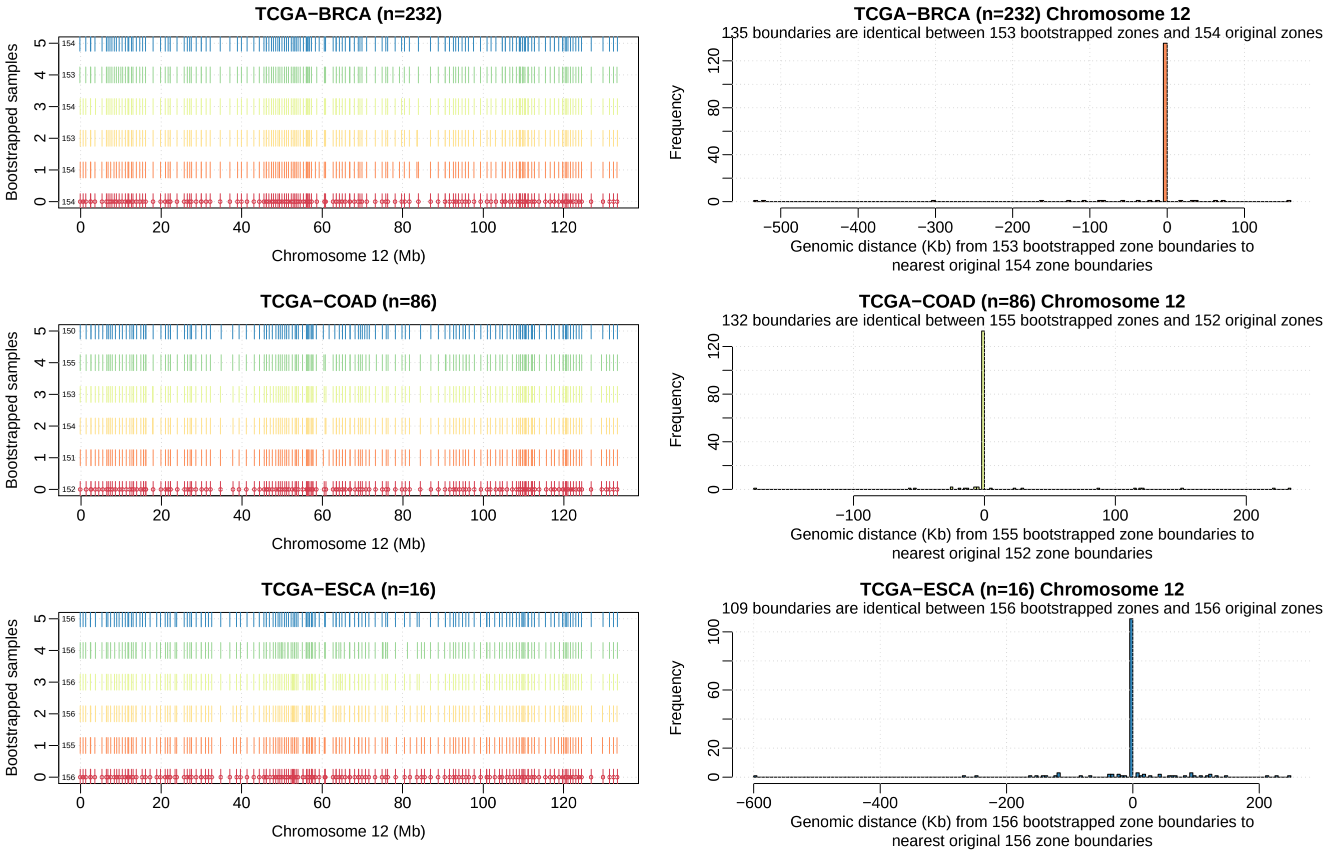

Figure N2.3: Zone boundaries and their bootstrapped estimation in three cancer types. Sample 0 is the original without bootstrapping. Sample 1 to 5 are bootstrapped. Zones estimated from the five bootstrapped samples and the original sample in three cancer types are shown on the left. Numbers marked to the left are total numbers of zone boundaries estimated from each sample. Histograms of distances between sample 5 and 0 are shown for each cancer type on the right.

N2.3 Outstanding genomic zones in cancer are enriched with genetic and epigenetic signals in human genome

To learn how the outstanding genomic zone maps in human cancers are associated with regulatory signals in the genome, we examined the enrichment of both genetic and epigenetic events in the detected genomic zones using LOLA: Genomic Locus Overlap Enrichment Analysis (Sheffield and Bock, 2016).

Different from gene set enrichment analyses that are independent of genomic loci, LOLA performs hypothesis testing on how genomic regions of known regulatory activity overlap with those from a new experiment. The core human (hg38) region database (`LOLACore_180412.tgz`) used by LOLA covers binding sites of thousands of both genetic and epigenetic factors from several sources including Cistrome (Zheng et al., 2019), CODEX (Sánchez-Castillo et al., 2015), ENCODE (Sloan et al., 2016), DNase-hypersensitivity profiles (Sheffield et al., 2013), and UCSC genomic features (Haeussler et al., 2019). Binding sites were profiled using chromatin immunoprecipitation sequencing (ChIP-seq), except UCSC genomic features which cover static properties of genome such as CpG island distribution.

For each cancer type, we separate the outstanding zones into two user sets: one containing all positively polarized outstanding zones and the other all negatively polarized. Here we postulate that different genetic/epigenetic signals may be involved in over- versus under-expressed zones. Each user set is intersected with each profile in the human genome region database to declare statistical enrichment.

Top ten genetic and epigenetic events that most overlap the outstanding genomic maps of each cancer type are summarized in Supplementary Table N2.1. Five are for positively polarized zones and five for the negatively polarized. Among the outstanding zones of all cancer types, most significantly enriched (2.7E-100) is Pol2 binding sites of breast cancer cell line MCF-7 in BRCA positively polarized zones. This suggests that positively polarized zones capture general transcription in breast cancer cells. The epigenetic modification of H3K4me3 is greatly enriched in negatively polarized zones of all cancer types and also enriched in positively polarized zones of several cancer types. Although H3K4me3 is most known to activate the transcription of nearby genes, here its association with negatively polarized zone expression may suggest an unconventional role in cancer. Binding sites of KDM4A in embryonic stem cell overlap with negatively polarized outstanding zones in all 17 cancer types except two (ESCA and HNSC). KDM4A encodes the lysine-specific demethylase, a transcriptional repressor, consistent with the negatively expressed zones in cancer. Previously, KDM4A is implicated in site-specific copy number gain in cancer (Black et al., 2013), but its association with down-regulation of many genomic zones seem unreported before.

Table N2.1: Top genetic and epigenetic events that are enriched in maps of outstanding genomic zones in 17 cancer types. The results are generated by LOLA genomic locus overlap enrichment analysis.

	cancerType	userSet	antibody	cellType	qValue	collection	dataSource	description
1	TCGA-BLCA	up	SAP30	Erythrocytic leukaemia cells	8.1E-62	codex	GSE32509	K562 erythrocytic leukaemia cells (ATCC CCL-243)
2		up	PLU1	K562	8.5E-62	encode_tfbs		ChIP K562 PLU1
3		up	Pol2	HepG2	1.1E-61	encode_tfbs		ChIP HepG2 Pol2
4		up	HDAC1	Erythrocytic leukaemia cells	1.1E-61	codex	GSE32509	K562 erythrocytic leukaemia cells (ATCC CCL-243)
5		up	Pol2	K562	1.1E-61	encode_tfbs		ChIP K562 Pol2
6		dn	KDM4A	Embryonic Stem Cell	2.6E-48	codex	GSE32509	K562 erythrocytic leukaemia cells (ATCC CCL-243)
7		dn			6.8E-47	ucsc_features		UCSC CpG islands
8		dn	H3K4me3	metastatic prostate cancer tissue	1.1E-44	cistrome_epigenome		cistrome_epigenome metastatic prostate cancer tissue H3K4me3
9		dn	H3K9ac	MCF-7	2.4E-42	cistrome_epigenome		cistrome_epigenome MCF-7 H3K9ac
10		dn	H3K4me3	LnCaP	2.4E-42	cistrome_epigenome		cistrome_epigenome LnCaP H3K4me3
11	TCGA-BRCA	up	Pol2	MCF-7	2.7E-100	encode_tfbs		ChIP MCF-7 Pol2
12		up	Pol2	GM12891	2.1E-90	encode_tfbs		ChIP GM12891 Pol2
13		up	Pol2	MCF-7	2.7E-90	encode_tfbs		ChIP MCF-7 Pol2
14		up	Pol2	GM12878	2.7E-90	encode_tfbs		ChIP GM12878 Pol2
15		up	Pol2-4H8	GM12891	1.5E-89	encode_tfbs		ChIP GM12891 Pol2-4H8

cancerType is the 17 cancer types that were studied. userSet indicates whether the positively (up) or negatively (dn) polarized outstanding zones are used for the corresponding enrichment analysis. For each cancer type, the top five enriched region sets are shown for the positively polarized outstanding zones and also top five enriched region sets are given for the negatively polarized outstanding zones.

Continued on next page

Table N2.1—continued from previous page

	cancerType	userSet	antibody	cellType	qValue	collection	dataSource	description
16		dn			7.1E-81	ucsc_features		UCSC CpG islands
17		dn	KDM4A	Embryonic Stem Cell	4.3E-79	codex	GSE32509	K562 erythrocytic leukaemia cells (ATCC CCL-243)
18		dn	H3K4me3	LnCaP	1.7E-77	cistrome_epigenome		cistrome_epigenome LnCaP H3K4me3
19		dn	H3K9ac	MCF-7	5.6E-76	cistrome_epigenome		cistrome_epigenome MCF-7 H3K9ac
20		dn	H3K4me3	metastatic prostate cancer tissue	9.1E-76	cistrome_epigenome		cistrome_epigenome metastatic prostate cancer tissue H3K4me3
21	TCGA-CHOL	up	Pol2	A549	7.3E-75	encode_tfbs		ChIP A549 Pol2
22		up	PHF8	Embryonic Stem Cell	1.1E-73	codex	GSE32509	K562 erythrocytic leukaemia cells (ATCC CCL-243)
23		up	POLR2A	Embryonic Stem Cell	2.0E-73	codex	GSE36578	codex Embryonic Stem Cell POLR2A
24		up	Pol2	A549	3.2E-73	encode_tfbs		ChIP A549 Pol2
25		up	KDM4A	Embryonic Stem Cell	9.4E-73	codex	GSE32509	K562 erythrocytic leukaemia cells (ATCC CCL-243)
26		dn			3.1E-41	ucsc_features		UCSC CpG islands
27		dn	KDM4A	Embryonic Stem Cell	3.0E-40	codex	GSE32509	K562 erythrocytic leukaemia cells (ATCC CCL-243)
28		dn	H3K9ac	MCF-7	1.6E-39	cistrome_epigenome		cistrome_epigenome MCF-7 H3K9ac
29		dn	H3K4me3	LnCaP	6.0E-39	cistrome_epigenome		cistrome_epigenome LnCaP H3K4me3
30		dn	H3K9ac	MCF-7	3.2E-38	cistrome_epigenome		cistrome_epigenome MCF-7 H3K9ac
31	TCGA-COAD	up	Pol2	HeLa-S3	2.0E-98	encode_tfbs		ChIP HeLa-S3 Pol2
32		up	Pol2	Gliobla	3.5E-98	encode_tfbs		ChIP Gliobla Pol2
33		up	Pol2	HeLa-S3	2.2E-95	encode_tfbs		ChIP HeLa-S3 Pol2
34		up	Pol2	HeLa-S3	7.2E-94	encode_tfbs		ChIP HeLa-S3 Pol2
35		up	Pol2	HepG2	4.2E-93	encode_tfbs		ChIP HepG2 Pol2
36		dn	H3K4me3	LnCaP	5.6E-70	cistrome_epigenome		cistrome_epigenome LnCaP H3K4me3
37		dn	KDM4A	Embryonic Stem Cell	9.9E-70	codex	GSE32509	K562 erythrocytic leukaemia cells (ATCC CCL-243)
38		dn	H3K4me3	LnCaP	4.1E-68	cistrome_epigenome		cistrome_epigenome LnCaP H3K4me3
39		dn	H3K4me3	metastatic prostate cancer tissue	1.8E-67	cistrome_epigenome		cistrome_epigenome metastatic prostate cancer tissue H3K4me3
40		dn	H3K4me3	CS58.LnCaP	1.1E-63	cistrome_epigenome		cistrome_epigenome CS58.LnCaP H3K4me3
41	TCGA-ESCA	up	PolII	MCF-7	3.6E-16	cistrome_cistrome		cistrome_cistrome MCF-7 PolII
42		up	VDR	lymphoblastoid(GM10861)	3.6E-16	cistrome_cistrome		cistrome_cistrome lymphoblastoid(GM10861) VDR
43		up	ERG	Leukaemia cell	3.6E-16	codex	GSE46044	Acute myelomonocytic leukaemia cell line carrying inv(16) translocation (CBF-beta
44		up	NFKB	GM12892	5.9E-16	encode_tfbs		ChIP GM12892 NFKB
45		up	H3K4me3	LnCaP	8.4E-16	cistrome_epigenome		cistrome_epigenome LnCaP H3K4me3
46		dn	H3K9ac	MCF-7	1.0E-14	cistrome_epigenome		cistrome_epigenome MCF-7 H3K9ac
47		dn	PolII	VCaP	1.2E-14	cistrome_cistrome		cistrome_cistrome VCaP PolII
48		dn	RARA	NB4 cells	1.7E-14	cistrome_cistrome		cistrome_cistrome NB4 cells RARA
49		dn	H3K4me3	metastatic prostate cancer tissue	1.7E-14	cistrome_epigenome		cistrome_epigenome metastatic prostate cancer tissue H3K4me3
50		dn	H3K4me3	CS57.LnCaP	2.5E-14	cistrome_epigenome		cistrome_epigenome CS57.LnCaP H3K4me3
51	TCGA-HNSC	up	KDM4A	Embryonic Stem Cell	3.9E-71	codex	GSE32509	K562 erythrocytic leukaemia cells (ATCC CCL-243)
52		up			7.0E-67	ucsc_features		UCSC CpG islands
53		up	Pol2	HeLa-S3	2.7E-60	encode_tfbs		ChIP HeLa-S3 Pol2
54		up	Pol2	ProgFib	2.1E-59	encode_tfbs		ChIP ProgFib Pol2
55		up	TAF1	H1-hESC	8.3E-59	encode_tfbs		ChIP H1-hESC TAF1
56		dn	H3K4me3	CS57.LnCaP	1.1E-57	cistrome_epigenome		cistrome_epigenome CS57.LnCaP H3K4me3
57		dn	H3K4me3	metastatic prostate cancer tissue	2.9E-55	cistrome_epigenome		cistrome_epigenome metastatic prostate cancer tissue H3K4me3
58		dn	H3K4me3	LnCaP	3.9E-55	cistrome_epigenome		cistrome_epigenome LnCaP H3K4me3
59		dn	H3K4me3	VCaP	1.4E-54	cistrome_epigenome		cistrome_epigenome VCaP H3K4me3
60		dn	FOXA2	Endoderm	9.7E-54	codex	GSE46130	Day 5 of in vitro differentiation with FGF2; BMP4 and B27
61	TCGA-KICH	up	H3K4me3	LnCaP	2.7E-82	cistrome_epigenome		cistrome_epigenome LnCaP H3K4me3
62		up			2.7E-82	ucsc_features		UCSC CpG islands
63		up	H3K4me3	CS57.LnCaP	4.9E-82	cistrome_epigenome		cistrome_epigenome CS57.LnCaP H3K4me3
64		up	H3K4me3	CS58.LnCaP	1.4E-81	cistrome_epigenome		cistrome_epigenome CS58.LnCaP H3K4me3
65		up	H3K4me3	LnCaP	7.6E-81	cistrome_epigenome		cistrome_epigenome LnCaP H3K4me3
66		dn	KDM4A	Embryonic Stem Cell	7.9E-86	codex	GSE32509	K562 erythrocytic leukaemia cells (ATCC CCL-243)
67		dn	H3K4me3	metastatic prostate cancer tissue	1.5E-79	cistrome_epigenome		cistrome_epigenome metastatic prostate cancer tissue H3K4me3
68		dn			1.6E-79	ucsc_features		UCSC CpG islands
69		dn	H3K9ac	MCF-7	2.7E-79	cistrome_epigenome		cistrome_epigenome MCF-7 H3K9ac
70		dn	H3K9ac	MCF-7	1.6E-75	cistrome_epigenome		cistrome_epigenome MCF-7 H3K9ac
71	TCGA-KIRC	up	H3K4me3	metastatic prostate cancer tissue	2.8E-73	cistrome_epigenome		cistrome_epigenome metastatic prostate cancer tissue H3K4me3
72		up	TAF1	H1-hESC	1.6E-72	encode_tfbs		ChIP H1-hESC TAF1
73		up	H3K4me3	CS58.LnCaP	2.4E-71	cistrome_epigenome		cistrome_epigenome CS58.LnCaP H3K4me3
74		up	KDM4A	Embryonic Stem Cell	4.8E-71	codex	GSE32509	K562 erythrocytic leukaemia cells (ATCC CCL-243)
75		up	H3K4me3	CS57.LnCaP	2.0E-70	cistrome_epigenome		cistrome_epigenome CS57.LnCaP H3K4me3
76		dn			3.3E-89	ucsc_features		UCSC CpG islands
77		dn	H3K4me3	metastatic prostate cancer tissue	8.5E-87	cistrome_epigenome		cistrome_epigenome metastatic prostate cancer tissue H3K4me3
78		dn	KDM4A	Embryonic Stem Cell	4.5E-86	codex	GSE32509	K562 erythrocytic leukaemia cells (ATCC CCL-243)

cancerType is the 17 cancer types that were studied. userSet indicates whether the positively (up) or negatively (dn) polarized outstanding zones are used for the corresponding enrichment analysis. For each cancer type, the top five enriched region sets are shown for the positively polarized outstanding zones and also top five enriched region sets are given for the negatively polarized outstanding zones.

Continued on next page

Table N2.1—continued from previous page

	cancerType	userSet	antibody	cellType	qValue	collection	dataSource	description
79		dn	H3K9ac	MCF-7	6.6E-80	cistrome_epigenome		cistrome_epigenome MCF-7 H3K9ac
80		dn	H3K4me3	CS58.LnCaP	1.2E-79	cistrome_epigenome		cistrome_epigenome CS58.LnCaP H3K4me3
81	TCGA-KIRP	up	KDM4A	Embryonic Stem Cell	2.6E-64	codex	GSE32509	K562 erythrocytic leukaemia cells (ATCC CCL-243)
82		up	Max	A549	2.6E-64	encode_tfbs		ChIP A549 Max
83		up			2.6E-64	ucsc_features		UCSC CpG islands
84		up	POLR2A	Embryonic Stem Cell	3.5E-64	codex	GSE36578	codex Embryonic Stem Cell POLR2A
85		up	TAF1	H1-hESC	9.4E-64	encode_tfbs		ChIP H1-hESC TAF1
86		dn			2.3E-66	ucsc_features		UCSC CpG islands
87		dn	H3K4me3	metastatic prostate cancer tissue	1.2E-65	cistrome_epigenome		cistrome_epigenome metastatic prostate cancer tissue H3K4me3
88		dn	KDM4A	Embryonic Stem Cell	3.6E-65	codex	GSE32509	K562 erythrocytic leukaemia cells (ATCC CCL-243)
89		dn	FOXA2	Endoderm	2.8E-62	codex	GSE46130	Day 5 of in vitro differentiation with FGF2; BMP4 and B27
90		dn	H3K9ac	MCF-7	8.0E-62	cistrome_epigenome		cistrome_epigenome MCF-7 H3K9ac
91	TCGA-LIHC	up	POLR2A	Leukemia cell	7.7E-92	codex	GSE50625	Jurkat T-ALL cell line treated with flavopiridol for 6 hours.
92		up	Pol2	K562	1.6E-91	encode_tfbs		ChIP K562 Pol2
93		up	SAP30	Erythrocytic leukaemia cells	3.2E-91	codex	GSE32509	K562 erythrocytic leukaemia cells (ATCC CCL-243)
94		up	POLR2A	Embryonic Stem Cell	3.9E-90	codex	GSE36578	codex Embryonic Stem Cell POLR2A
95		up	Pol2(phosphoS2)	HeLa-S3	3.9E-90	encode_tfbs		ChIP HeLa-S3 Pol2(phosphoS2)
96		dn			8.5E-66	ucsc_features		UCSC CpG islands
97		dn	CTCF	Erythroid Progenitor	1.4E-63	codex	GSE26501	CD36+ erythroid progenitor cells were derived from mobilised CD34+ cells isolate
98		dn	KDM4A	Embryonic Stem Cell	1.1E-62	codex	GSE32509	K562 erythrocytic leukaemia cells (ATCC CCL-243)
99		dn	H3K4me3	metastatic prostate cancer tissue	1.1E-62	cistrome_epigenome		cistrome_epigenome metastatic prostate cancer tissue H3K4me3
100		dn	CTCF	Hematopoietic Stem and Progenitor Cells	4.3E-60	codex	GSE26501	Mobilised CD34+ cells isolated from the healthy peripheral blood.
101	TCGA-LUAD	up	Pol2	A549	3.6E-85	encode_tfbs		ChIP A549 Pol2
102		up	H3K4me3	LnCaP	3.6E-85	cistrome_epigenome		cistrome_epigenome LnCaP H3K4me3
103		up	Pol2	A549	6.1E-85	encode_tfbs		ChIP A549 Pol2
104		up	Pol2	A549	5.3E-82	encode_tfbs		ChIP A549 Pol2
105		up	Pol2	HeLa-S3	2.1E-81	encode_tfbs		ChIP HeLa-S3 Pol2
106		dn	KDM4A	Embryonic Stem Cell	4.2E-83	codex	GSE32509	K562 erythrocytic leukaemia cells (ATCC CCL-243)
107		dn			9.6E-76	ucsc_features		UCSC CpG islands
108		dn	FOXA2	Endoderm	2.6E-72	codex	GSE46130	Day 5 of in vitro differentiation with FGF2; BMP4 and B27
109		dn	H3K4me3	metastatic prostate cancer tissue	2.2E-70	cistrome_epigenome		cistrome_epigenome metastatic prostate cancer tissue H3K4me3
110		dn	H3K4me3	VCaP	4.8E-68	cistrome_epigenome		cistrome_epigenome VCaP H3K4me3
111	TCGA-LUSC	up	H3K4me3	CS57.LnCaP	1.4E-87	cistrome_epigenome		cistrome_epigenome CS57.LnCaP H3K4me3
112		up	PHF8	Erythrocytic leukaemia cells	1.6E-87	codex	GSE32509	K562 erythrocytic leukaemia cells (ATCC CCL-243)
113		up	H3K4me3	LnCaP	4.2E-86	cistrome_epigenome		cistrome_epigenome LnCaP H3K4me3
114		up	PHF8	Erythrocytic leukaemia cells	5.9E-86	codex	GSE32509	K562 erythrocytic leukaemia cells (ATCC CCL-243)
115		up			8.1E-86	ucsc_features		UCSC CpG islands
116		dn	KDM4A	Embryonic Stem Cell	2.3E-83	codex	GSE32509	K562 erythrocytic leukaemia cells (ATCC CCL-243)
117		dn	H3K4me3	metastatic prostate cancer tissue	5.3E-82	cistrome_epigenome		cistrome_epigenome metastatic prostate cancer tissue H3K4me3
118		dn	H3K9ac	MCF-7	3.6E-79	cistrome_epigenome		cistrome_epigenome MCF-7 H3K9ac
119		dn			3.4E-77	ucsc_features		UCSC CpG islands
120		dn	FOXA2	Endoderm	2.1E-76	codex	GSE46130	Day 5 of in vitro differentiation with FGF2; BMP4 and B27
121	TCGA-PRAD	up	H3K4me3	LnCaP	9.6E-84	cistrome_epigenome		cistrome_epigenome LnCaP H3K4me3
122		up	H3K36me3	LnCaP	1.1E-79	cistrome_epigenome		cistrome_epigenome LnCaP H3K36me3
123		up	PolII	VCaP	1.8E-79	cistrome_cistrome		cistrome_cistrome VCaP PolII
124		up	H3K4me3	LnCaP	1.8E-79	cistrome_epigenome		cistrome_epigenome LnCaP H3K4me3
125		up	H3K4me3	VCaP	6.5E-78	cistrome_epigenome		cistrome_epigenome VCaP H3K4me3
126		dn	KDM4A	Embryonic Stem Cell	8.6E-75	codex	GSE32509	K562 erythrocytic leukaemia cells (ATCC CCL-243)
127		dn			2.9E-72	ucsc_features		UCSC CpG islands
128		dn	FOXA2	Endoderm	1.0E-66	codex	GSE46130	Day 5 of in vitro differentiation with FGF2; BMP4 and B27
129		dn	H3K4me3	CS58.LnCaP	2.6E-66	cistrome_epigenome		cistrome_epigenome CS58.LnCaP H3K4me3
130		dn	H3K4me3	metastatic prostate cancer tissue	3.3E-65	cistrome_epigenome		cistrome_epigenome metastatic prostate cancer tissue H3K4me3
131	TCGA-READ	up	Pol2	HepG2	7.4E-55	encode_tfbs		ChIP HepG2 Pol2
132		up	SAP30	Erythrocytic leukaemia cells	9.0E-54	codex	GSE32509	K562 erythrocytic leukaemia cells (ATCC CCL-243)
133		up	Pol2	HeLa-S3	5.1E-53	encode_tfbs		ChIP HeLa-S3 Pol2
134		up	TAF1	HeLa-S3	5.4E-53	encode_tfbs		ChIP HeLa-S3 TAF1
135		up	Pol2	GM18951	8.1E-53	encode_tfbs		ChIP GM18951 Pol2
136		dn	KDM4A	Embryonic Stem Cell	6.7E-40	codex	GSE32509	K562 erythrocytic leukaemia cells (ATCC CCL-243)
137		dn			2.1E-37	ucsc_features		UCSC CpG islands
138		dn	H3K4me3	LnCaP	1.7E-36	cistrome_epigenome		cistrome_epigenome LnCaP H3K4me3
139		dn	H3K4me3	metastatic prostate cancer tissue	4.3E-36	cistrome_epigenome		cistrome_epigenome metastatic prostate cancer tissue H3K4me3

cancerType is the 17 cancer types that were studied. userSet indicates whether the positively (up) or negatively (dn) polarized outstanding zones are used for the corresponding enrichment analysis. For each cancer type, the top five enriched region sets are shown for the positively polarized outstanding zones and also top five enriched region sets are given for the negatively polarized outstanding zones.

Continued on next page

Table N2.1—continued from previous page

	cancerType	userSet	antibody	cellType	qValue	collection	dataSource	description
140		dn	H3K4me3	CS58.LnCaP	4.4E-36	cistrome_epigenome		cistrome_epigenome CS58.LnCaP H3K4me3
141	TCGA-STAD	up	MAZ_(ab85725)	GM12878	3.4E-48	encode_tfbs		ChIP GM12878 MAZ_(ab85725)
142		up	SAP30	Erythrocytic leukaemia cells	1.9E-46	codex	GSE32509	K562 erythrocytic leukaemia cells (ATCC CCL-243)
143		up	Pol2	A549	5.4E-46	encode_tfbs		ChIP A549 Pol2
144		up	Pol2	HeLa-S3	8.3E-46	encode_tfbs		ChIP HeLa-S3 Pol2
145		up	HDAC1	Erythrocytic leukaemia cells	1.2E-45	codex	GSE32509	K562 erythrocytic leukaemia cells (ATCC CCL-243)
146		dn	H3K4me3	CS58.LnCaP	9.8E-36	cistrome_epigenome		cistrome_epigenome CS58.LnCaP H3K4me3
147		dn	H3K4me3	MCF-7	1.4E-35	cistrome_epigenome		cistrome_epigenome MCF-7 H3K4me3
148		dn	KDM4A	Embryonic Stem Cell	1.8E-34	codex	GSE32509	K562 erythrocytic leukaemia cells (ATCC CCL-243)
149		dn			2.1E-34	ucsc_features		UCSC CpG islands
150		dn	H3K4me3	LnCaP	2.3E-34	cistrome_epigenome		cistrome_epigenome LnCaP H3K4me3
151	TCGA-THCA	up	POLR2A	Embryonic Stem Cell	4.1E-75	codex	GSE36578	codex Embryonic Stem Cell POLR2A
152		up			6.7E-75	ucsc_features		UCSC CpG islands
153		up	PolII	MCF-7	1.1E-71	cistrome_cistrome		cistrome_cistrome MCF-7 PolII
154		up	KDM4A	Embryonic Stem Cell	2.6E-71	codex	GSE32509	K562 erythrocytic leukaemia cells (ATCC CCL-243)
155		up	TAF1	H1-hESC	9.0E-71	encode_tfbs		ChIP H1-hESC TAF1
156		dn	KDM4A	Embryonic Stem Cell	1.5E-80	codex	GSE32509	K562 erythrocytic leukaemia cells (ATCC CCL-243)
157		dn			3.7E-79	ucsc_features		UCSC CpG islands
158		dn	H3K4me3	VCaP	1.2E-76	cistrome_epigenome		cistrome_epigenome VCaP H3K4me3
159		dn	H3K9me3	VCaP	1.2E-76	cistrome_epigenome		cistrome_epigenome VCaP H3K9me3
160		dn	H3K4me3	metastatic prostate cancer tissue	4.5E-76	cistrome_epigenome		cistrome_epigenome metastatic prostate cancer tissue H3K4me3
161	TCGA-UCEC	up	PLU1	K562	1.0E-63	encode_tfbs		ChIP K562 PLU1
162		up	KDM5B	Erythrocytic leukaemia cells	8.2E-63	codex	GSE32509	K562 erythrocytic leukaemia cells (ATCC CCL-243)
163		up	PHF8_(A301-772A)	K562	8.5E-62	encode_tfbs		ChIP K562 PHF8_(A301-772A)
164		up	Pol2	HeLa-S3	2.4E-61	encode_tfbs		ChIP HeLa-S3 Pol2
165		up	HDAC1	Erythrocytic leukaemia cells	3.7E-61	codex	GSE32509	K562 erythrocytic leukaemia cells (ATCC CCL-243)
166		dn	KDM4A	Embryonic Stem Cell	6.6E-54	codex	GSE32509	K562 erythrocytic leukaemia cells (ATCC CCL-243)
167		dn			5.0E-51	ucsc_features		UCSC CpG islands
168		dn	H3K4me3	metastatic prostate cancer tissue	3.7E-50	cistrome_epigenome		cistrome_epigenome metastatic prostate cancer tissue H3K4me3
169		dn	FOXA2	Endoderm	9.8E-49	codex	GSE46130	Day 5 of in vitro differentiation with FGF2; BMP4 and B27
170		dn	H3K4me3	LnCaP	1.7E-48	cistrome_epigenome		cistrome_epigenome LnCaP H3K4me3

cancerType is the 17 cancer types that were studied. userSet indicates whether the positively (up) or negatively (dn) polarized outstanding zones are used for the corresponding enrichment analysis. For each cancer type, the top five enriched region sets are shown for the positively polarized outstanding zones and also top five enriched region sets are given for the negatively polarized outstanding zones.

N2.4 The number of polarity conserved loci across cancer types is statistically significantly higher than what is expected by chance

Here, we examine whether the 109 polarity conserved loci across cancer types could arise by chance. On each chromosome, genomic zones of all cancer types are intersected to obtain fine zones on that chromosome. A fine zone is conserved in polarity if that fine zone comes from an outstanding zone and have the same polarity in over 80% ($\geq$14) cancer types. We thus obtained 304 conserved fine zones from input zone maps of all cancer types. Fine zones that are neighbors along a chromosome are merged to give the 109 conserved loci reported in the main text.

We developed a permutation test to determine if the number of conserved fine zones (304) is statistically significant. Using the input set of zone maps, we generated 1000 sets of permuted maps to represent the null distribution. For a given chromosome of a given cancer type, the order of the zones are randomly shuffled from the input zones of that chromosome and cancer type. A permuted zone has the same width and polarity with an original zone but its chromosomal coordinates are randomly arranged. For each set of permuted maps for all cancer types, the number of conserved fine zones is recorded. The collection of numbers from all permutations constitutes the null distribution. The proportion with equal or more than 304 conserved fine zones is reported to be the P-value.

Our simulation generated the null distribution of the number of conserved fine zones as shown in Fig N2.4. No permuted zone map returned more than the number of observed conserved fine maps. As the smallest non-zero P-value of 1000 permutation is 0.001, we thus report the P-value of the observed fine zones as

<0.001. Therefore, we conclude that the zone polarity conservation across cancer types is statistically significantly unexpected by chance.

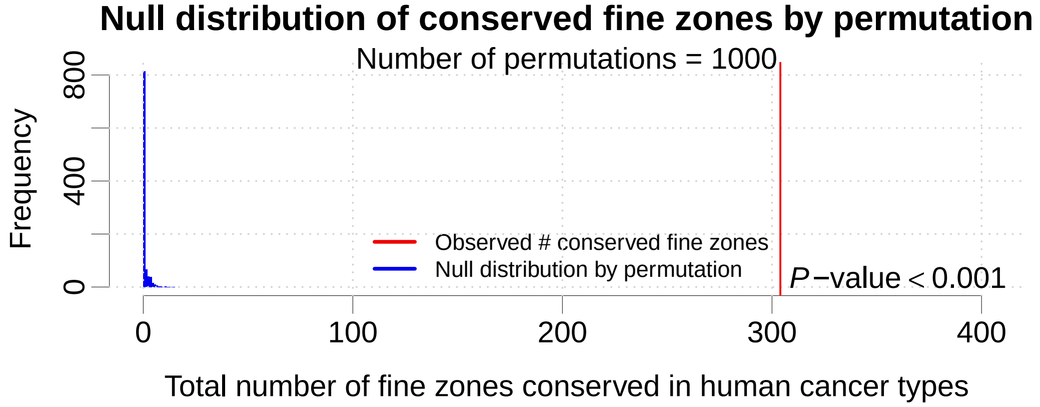

Figure N2.4: The null distribution and statistical significance of the number of conserved fine zones. The null distribution is obtained by permuting 1000 times the orders of zones along each chromosome in each cancer type while the zones do not change in their width or polarity. The horizontal location of the red line is the observed number (304) of conserved fine zones across cancer types.

References

Black, J. C., Manning, A. L., Van Rechem, C., Kim, J., Ladd, B., Cho, J., Pineda, C. M., Murphy, N., Daniels, D. L., Montagna, C., Lewis, P. W., Glass, K., Allis, C. D., Dyson, N. J., Getz, G., and Whetstine, J. R. (2013). KDM4A lysine demethylase induces site-specific copy gain and rereplication of regions amplified in tumors. *Cell*, 154(3):541–555.

Haeussler, M., Zweig, A. S., Tyner, C., Speir, M. L., Rosenbloom, K. R., Raney, B. J., Lee, C. M., Lee, B. T., Hinrichs, A. S., Gonzalez, J. N., Gibson, D., Diekhans, M., Clawson, H., Casper, J., Barber, G. P., Haussler, D., Kuhn, R. M., and Kent, W. J. (2019). The UCSC Genome Browser database: 2019 update. *Nucleic acids research*, 47(D1):D853–D858.

Sánchez-Castillo, M., Ruau, D., Wilkinson, A. C., Ng, F. S. L., Hannah, R., Diamanti, E., Lombard, P., Wilson, N. K., and Gottgens, B. (2015). CODEX: a next-generation sequencing experiment database for the haematopoietic and embryonic stem cell communities. *Nucleic Acids Res*, 43(Database issue):D1117–23.

Sheffield, N. C. and Bock, C. (2016). LOLA: Enrichment analysis for genomic region sets and regulatory elements in R and Bioconductor. *Bioinformatics*, 32(4):587–589.

Sheffield, N. C., Thurman, R. E., Song, L., Safi, A., Stamatoyannopoulos, J. A., Lenhard, B., Crawford, G. E., and Furey, T. S. (2013). Patterns of regulatory activity across diverse human cell types predict tissue identity, transcription factor binding, and long-range interactions. *Genome Research*, 23(5):777–788.

Sloan, C. A., Chan, E. T., Davidson, J. M., Malladi, V. S., Strattan, J. S., Hitz, B. C., Gabdank, I., Narayanan, A. K., Ho, M., Lee, B. T., Rowe, L. D., Dreszer, T. R., Roe, G., Podduturi, N. R., Tanaka, F., Hong, E. L., and Cherry, J. M. (2016). ENCODE data at the ENCODE portal. *Nucleic Acids Res*, 44(D1):D726–32.

Zheng, R., Wan, C., Mei, S., Qin, Q., Wu, Q., Sun, H., Chen, C.-H., Brown, M., Zhang, X., Meyer, C. A., and Liu, X. S. (2019). Cistrome Data Browser: expanded datasets and new tools for gene regulatory analysis. *Nucleic Acids Res*, 47(D1):D729–D735.
