# Supplementary material for: Efficient weighted univariate clustering maps outstanding dysregulated genomic zones in human cancers
Source: Bioinformatics. 2020 Jul 3;36(20):5027–36. doi: 10.1093/bioinformatics/btaa613 (PMC7755420; doi:10.1093/bioinformatics/btaa613)
Supplement: btaa613_Supplementary_Data [file btaa613_supplementary_data.zip › SuppNote-N3.pdf]

Supplementary Note N3: Summary of top dysregulated genomic zones in human cancers

Legend used in zone visualization

Each ‘x’ marks one patient with a pair of matched tumor-normal samples. Colors of the ‘x’ marks indicate different patients. The horizontal location of ‘x’ represents a unique gene.

Genes within a zone are ordered by their start genomic coordinates. They are equally spaced in the visualization within the zone, not linearly proportional to their genomic distance.

Known cancer genes from COSMIC Cancer Gene Census (CGC) version 87 and Network of Cancer Genes 6 are marked along the chromosomes. Those highly differentially regulated are marked in bold font. Six cancer loci from CGC—*IGH@*, *IGK@*, *IGL@*, *TRA@*, *TRB@*, and *TRD@*—are not marked, as they are not genes and thus no expression data is available.

a, The name of a gene is marked at its relative position only if its expression changed substantially between tumor and matched normal tissues. The color of a gene name text is red/blue for up-/down-regulation in cancer versus normal.

b, The name of a gene is marked at its relative position only if its copy number changed substantially between tumor and matched normal tissues. The color of a gene name text is purple/green for amplification/deletion in somatic copy number in cancer versus normal.

1 Top dysregulated genomic zones consistent with SCNA polarization

Though varying greatly over cancer types, many top polarized regulation zones are consistent with SCNA polarization. These zones frequently hit known cancer genes in Fig N3.1 and Supplementary Figure S3. Known cancer genes are obtained by combining CGC v87 and Network of Cancer Genes (NCG) 6 (Repana et al., 2018).

The 3rd most polarized regulation zone (chr1:154.8-155.4 Mb; Fig N3.1a,b) in BRCA contains the *MUC1* gene, encoding an oncoprotein highly expressed in malignant breast tumors in contrast to non-malignant tumors or healthy tissues (Zaretsky et al., 1990; Kufe, 2013). The number of clinical trials on MUC1-targeted cancer immunotherapy has reached a historical high in 2017 (Taylor-Papadimitriou et al., 2018). The selection of the *MUC1* neighborhood as a top zone is less due to the high expression level of *MUC1* than the consistent up-regulation of genes in this zone in BRCA. This is partially supported by previous studies that the *MUC1* promoter is comprised of binding sites for diverse transcription factors activated in BRCA (Abe and Kufe, 1993).

In COAD and READ, the top six regulation polarization zones are identical: each same ranked pair of zones in both cancers occupies almost the same genomic regions and shares the same polarity. The cancer locus of immunoglobulin lambda (*IGL@*) from CGC v87, chr22:22.6-23.4 Mb in COAD (Fig N3.1c) and chr22:22.2-23.5 Mb in READ (Fig N3.1i), is the fifth most polarized regulation zones for both cancer types. The zone is dominated by down-regulated and deleted immunoglobulin lambda variable genes. *BCR*, a known cancer fusion gene (CGC v87), is up-regulated in most tumor samples despite its somatic copy number loss (Fig N3.1d,j). The phosphorylation of BCR maintains β-catenin transcriptional activity implicated in tumor cell invasion in colorectal cancer (Jeitany et al., 2018). Independently from Human Protein Altas (Uhlen et al., 2015), all 12 patients of colorectal cancer showed high or medium BCR protein expression.

The most polarized zone of ESCA, chr17:35.3-36.1 Mb, is up-regulated (Fig N3.1e) and amplified (Fig N3.1f). This zone includes a known cancer gene *TAF15*, a player in the general transcription machinery. The TAF15 proto-oncoprotein is overexpressed in esophageal squamous cell carcinoma (Pawar et al., 2011).

The second most polarized zone of KICH chr1:152.8-153.8 Mb is down-regulated (Fig N3.1g), also significantly reduced in somatic copy number (Fig N3.1h). It includes a known cancer gene *S100A7* and several other genes encoding members in the S100 protein family such as *S100A1*, *S100A2*, *S100A4*, and *S100A6*. They are all remarkably down-regulated, consistent with the immunonegativity of S100A1 protein expression in KICH, known to be a powerful marker of KICH against other kidney cancer types (Li et al., 2007).

2 Top dysregulated genomic zones with polarity opposite to SCNA

On the other hand, many other top zones exhibit opposite polarity between regulation and SCNA (Fig N3.2). The most significant polarized regulation zone in BRCA located at chr17:68.9-70.7 Mb (Fig N3.2a,b) is down-regulated, consistent among $\geq$ 14 (80%) cancer types. Here several ABC transporter genes are found, among which *ABCA10* is involved in gastric adenocarcinoma (NCG6). It is under-expressed in nearly all 112 tumor-normal matched pairs. Despite a general understanding of the connection between ABC transporters and cancer drug resistance, other roles for *ABCA10* in cancer including BRCA is emerging but unclear (Fletcher et al., 2010).

In spite of a gain in somatic copy number, the immunoglobulin kappa locus (*IGK@*) is heavily down-regulated in both COAD (Top 3 and 4 of COAD: Fig S3.38 and S3.55) and READ (Top 3 and 4 of READ: Fig S3.48 and S3.65), which contradicts results of high expression levels of *IGK@* in human colorectal patients (Yang et al., 2009). Although *IGK@* is a known locus of cancer driver genes for lymphoma (CGC v87), it is not yet established for roles in colorectal cancers.

The most polarized regulation zone in KIRC is located at chr7:142.3-143 Mb (Fig N3.2c,d). Although this zone is amplified in somatic copy number, five know cancer genes (CGC v87 + NCG6) are under-expressed. *TRPV5* and *TRPV6* are lowly expressed in nearly all 72 tumor-normal pairs, supported by independent findings of both genes being significantly under-expressed in renal cell carcinoma over benign tissues (Wu et al., 2011).

The fifth polarized zone of LUSC, chr7:37.8-38.5 Mb, is down-regulated, but has a significantly increased somatic copy number (Fig S3.80). This zone contains known tumor suppressor gene *SFRP4* encoding secreted frizzled-related protein 4. Its promoter is often hypermethylated in cancers to compromise its role of apoptosis inhibitor (Pohl et al., 2015), though such a mechanism has not been reported for lung cancers.

In LUAD and KIRC, the immunoglobulin heavy locus (*IGH@*) is highly expressed (Fig N3.2e) but at a somatic copy number loss (Fig N3.2f). This is shown in three zones of LUAD (Top 1, 2 and 5 of LUAD: Fig S3.11, S3.28, and S3.79) and the top 5 zone of KIRC at chr14:106.1-106.9 Mb (Fig S3.76). Such aberrant genome rearrangement is unknown for either cancer type, though it is observed in lymphoma driven by long established MYC-IGH translocation (Taub et al., 1982). This raises a question as to what caused *IGH@* to over-express despite its lost somatic copies in LUAD and KIRC.

In THCA, six out of top ten polarized regulation zones have highly divergent polarity from SCNA (Figure N3.3 to Figure N3.12).

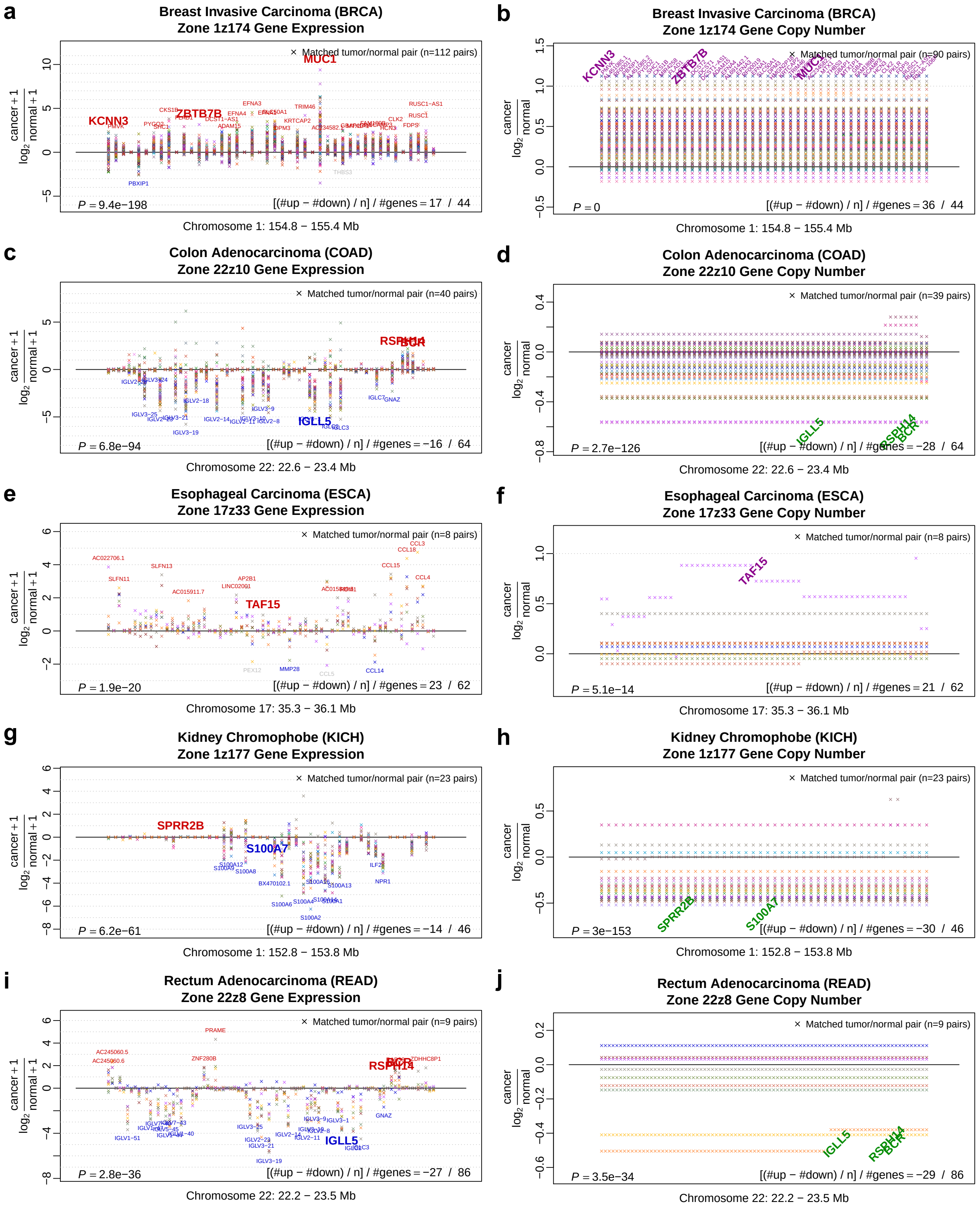

Figure N3.1: Top polarized regulation zones that are also consistent with SCNA polarization. The 3rd, 5th, 1st, 2rd, and 5th most significantly polarized regulation zones of BRCA, COAD, ESCA, KICH, and READ contain important known cancer genes from CGC v87 or NCG6, as marked in large bold font.

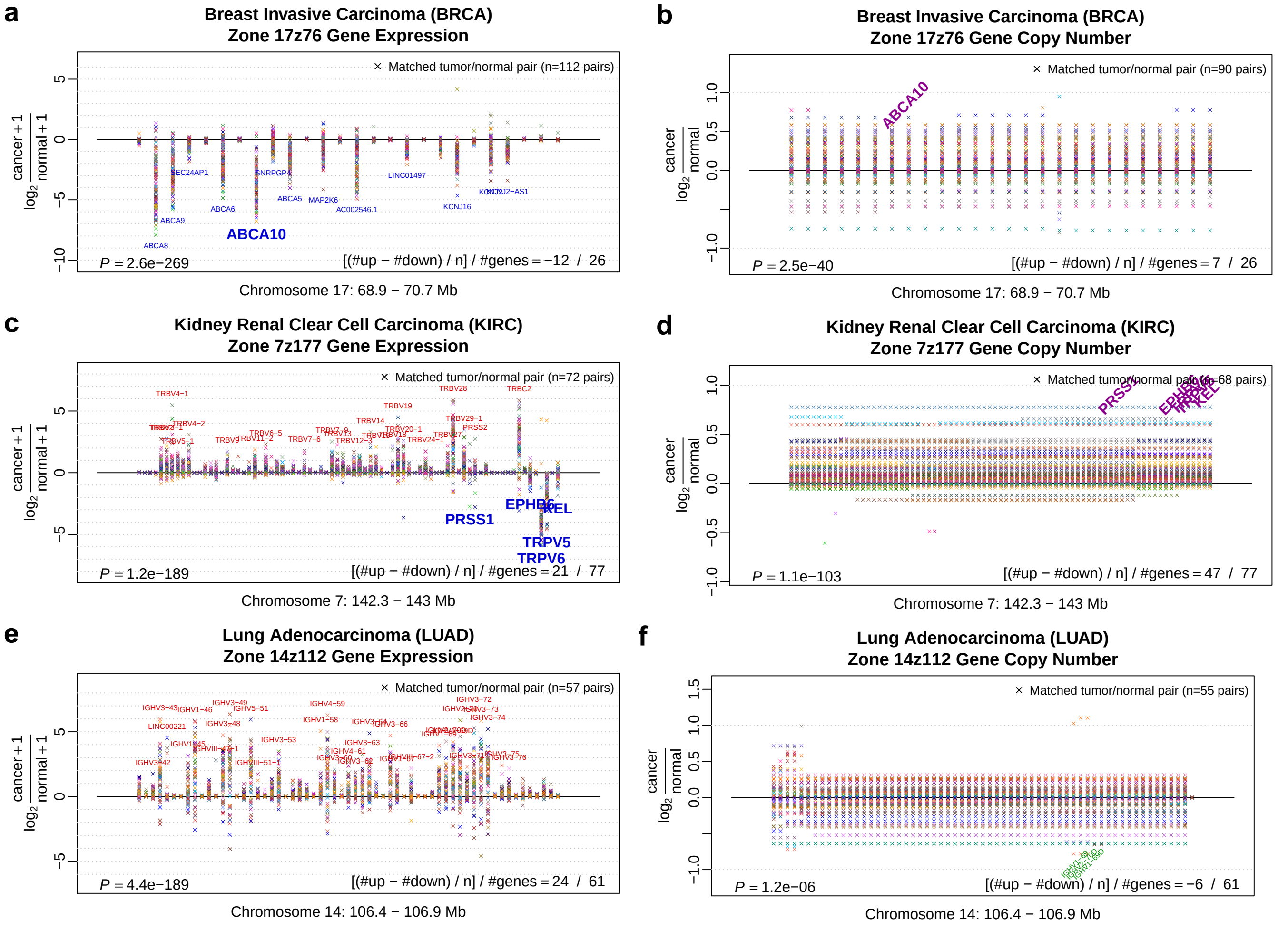

Figure N3.2: Top polarized zones of regulation in several cancer types are either reversely or insignificantly polarized in somatic copy number alteration. The most significant polarized regulation zones of BRCA, KIRC, and LUAD and the corresponding SCNA polarization are shown. Known cancer genes from CGC v87 or NCG6 are marked in large bold font.

a

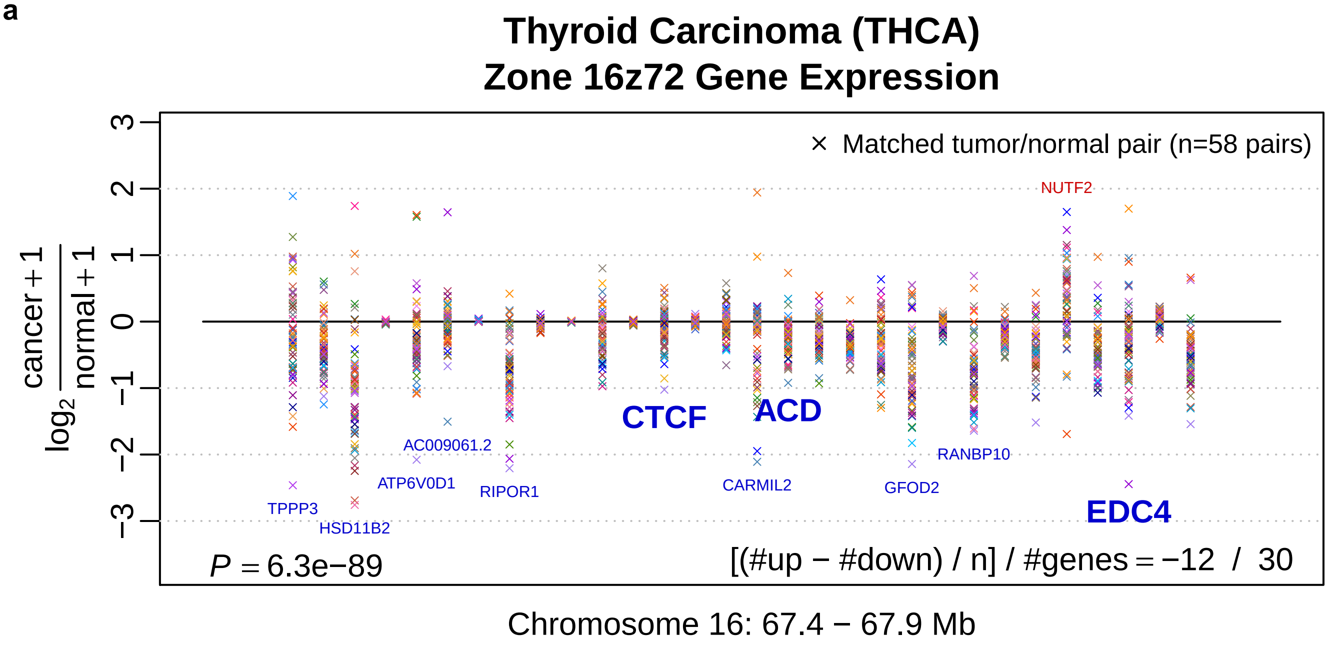

b

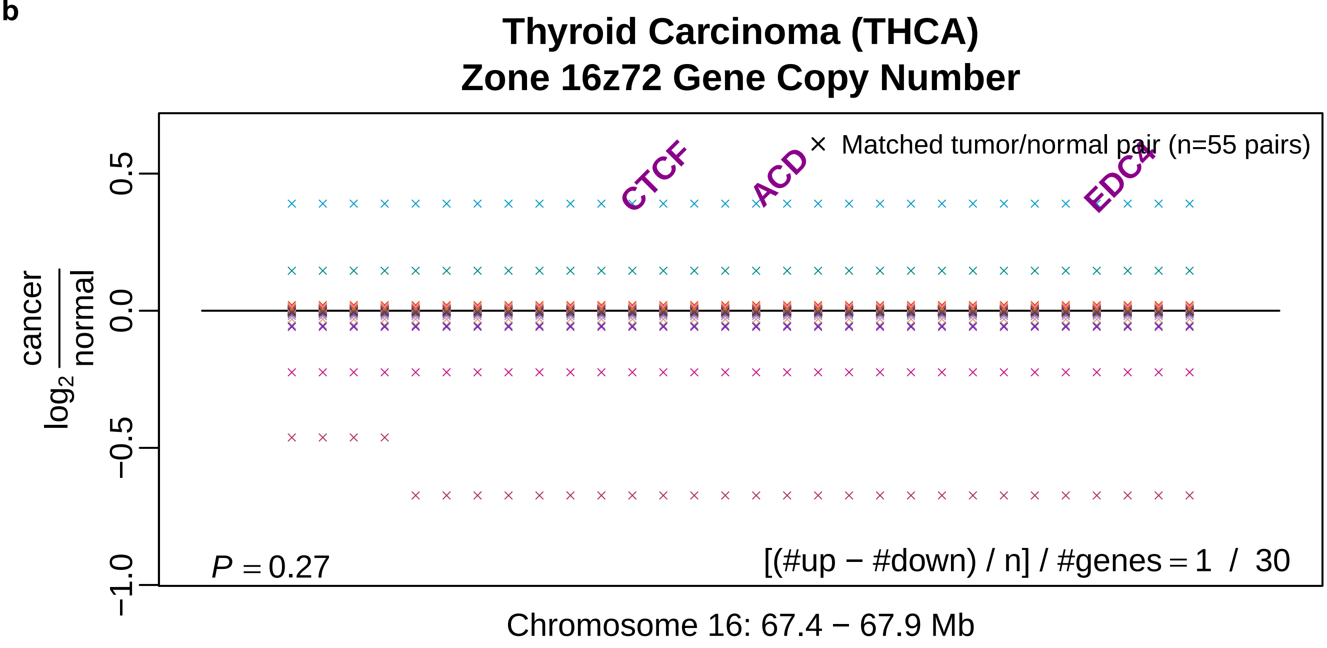

Figure N3.3: The most statistically significant polarized regulation zone in thyroid carcinoma (THCA). Significant zone regulation polarization is not associated with insignificant polarization in zone somatic copy number alteration. **a**, The zone is significantly negatively polarized in regulation in cancer over matched normal. **b**, The same zone is insignificantly polarized in somatic copy number alteration in cancer over matched normal.

a

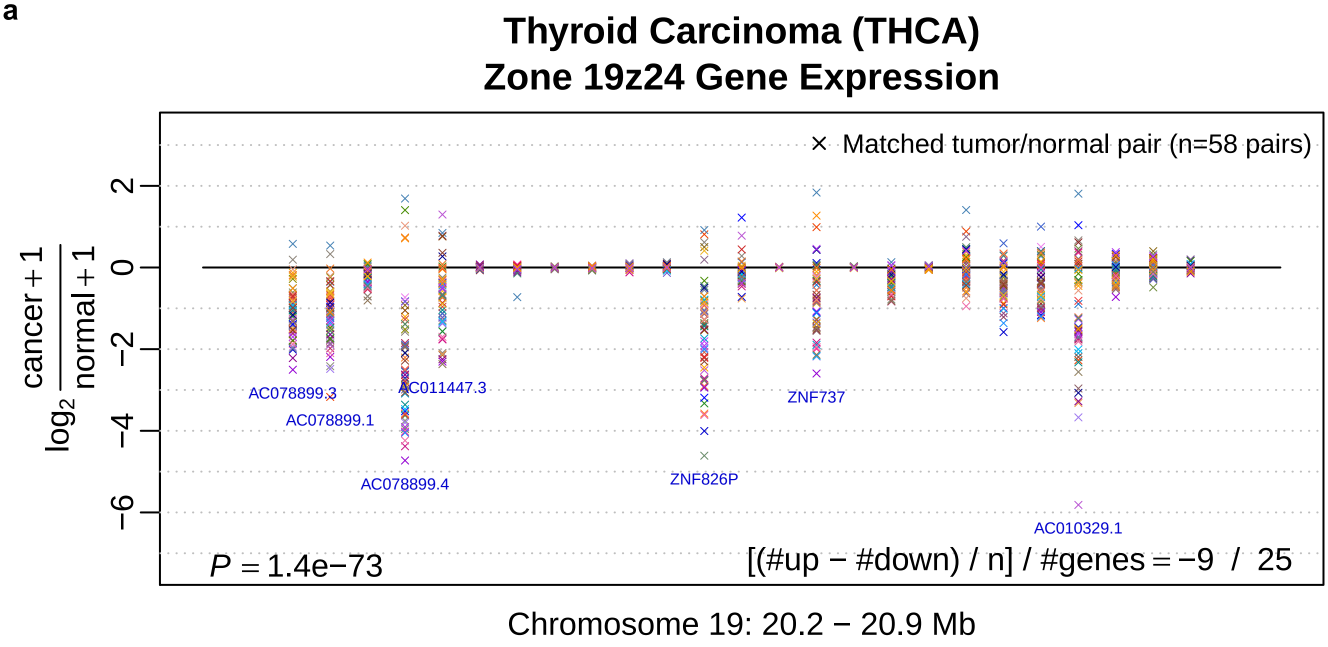

b

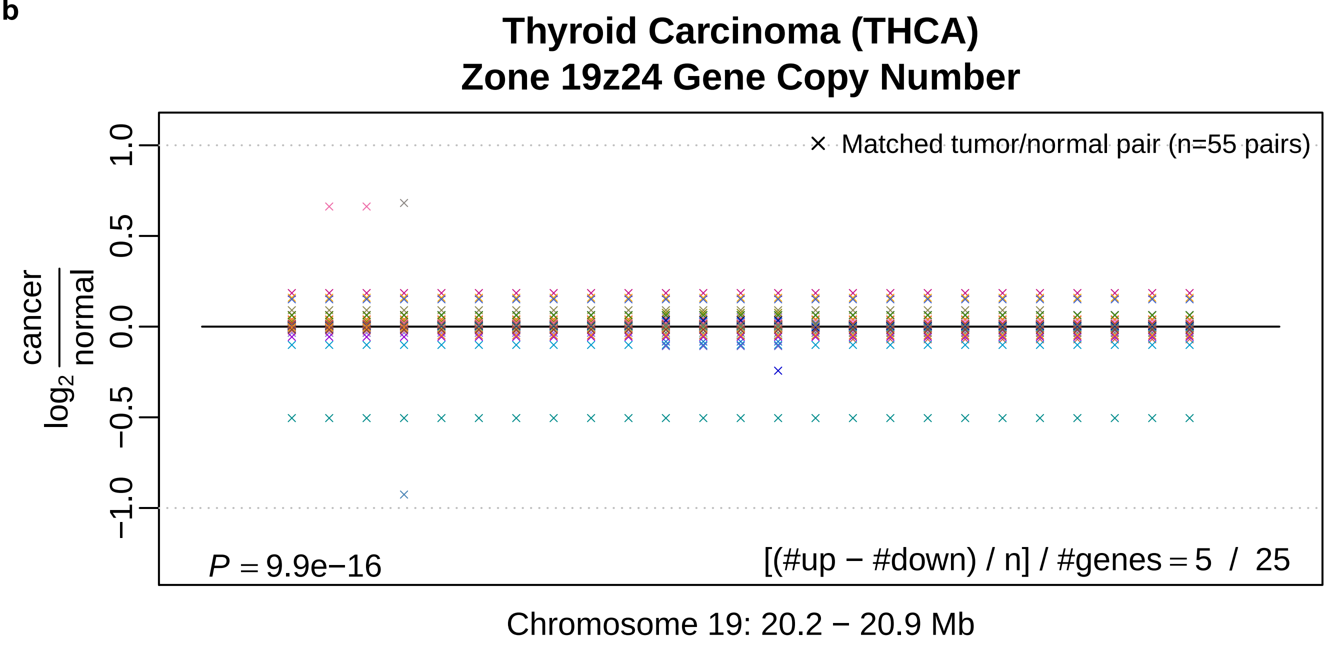

Figure N3.4: The second most statistically significant polarized regulation zone in thyroid carcinoma (THCA). Negative zone regulation polarization is inconsistent with positive polarization in zone somatic copy number alteration. **a**, The zone is significantly negatively (-) polarized in regulation in cancer over matched normal. **b**, The same zone is significantly positively (+) polarized in somatic copy number alteration in cancer over matched normal.

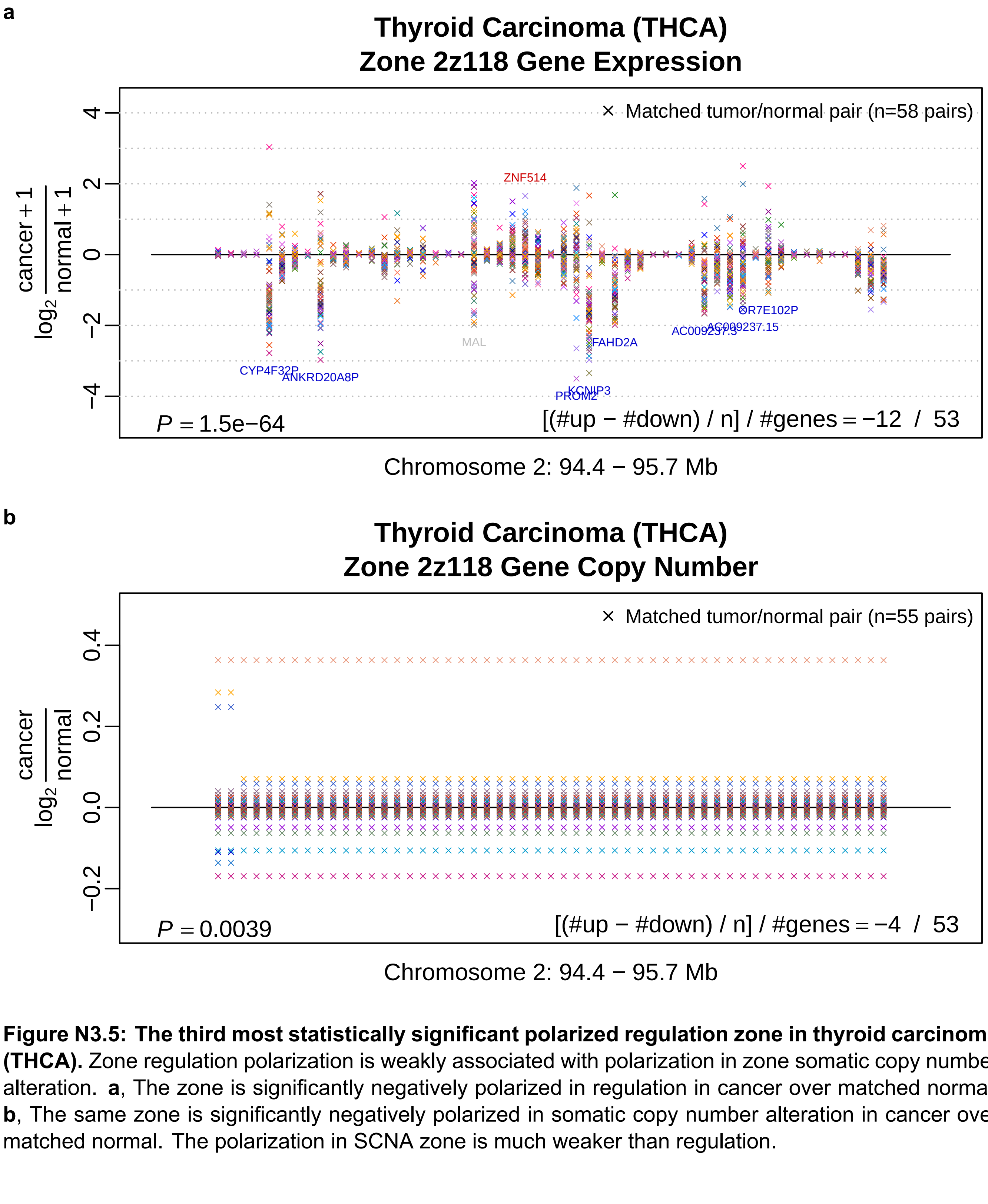

Figure N3.5: The third most statistically significant polarized regulation zone in thyroid carcinoma (THCA). Zone regulation polarization is weakly associated with polarization in zone somatic copy number alteration. **a**, The zone is significantly negatively polarized in regulation in cancer over matched normal. **b**, The same zone is significantly negatively polarized in somatic copy number alteration in cancer over matched normal. The polarization in SCNA zone is much weaker than regulation.

a

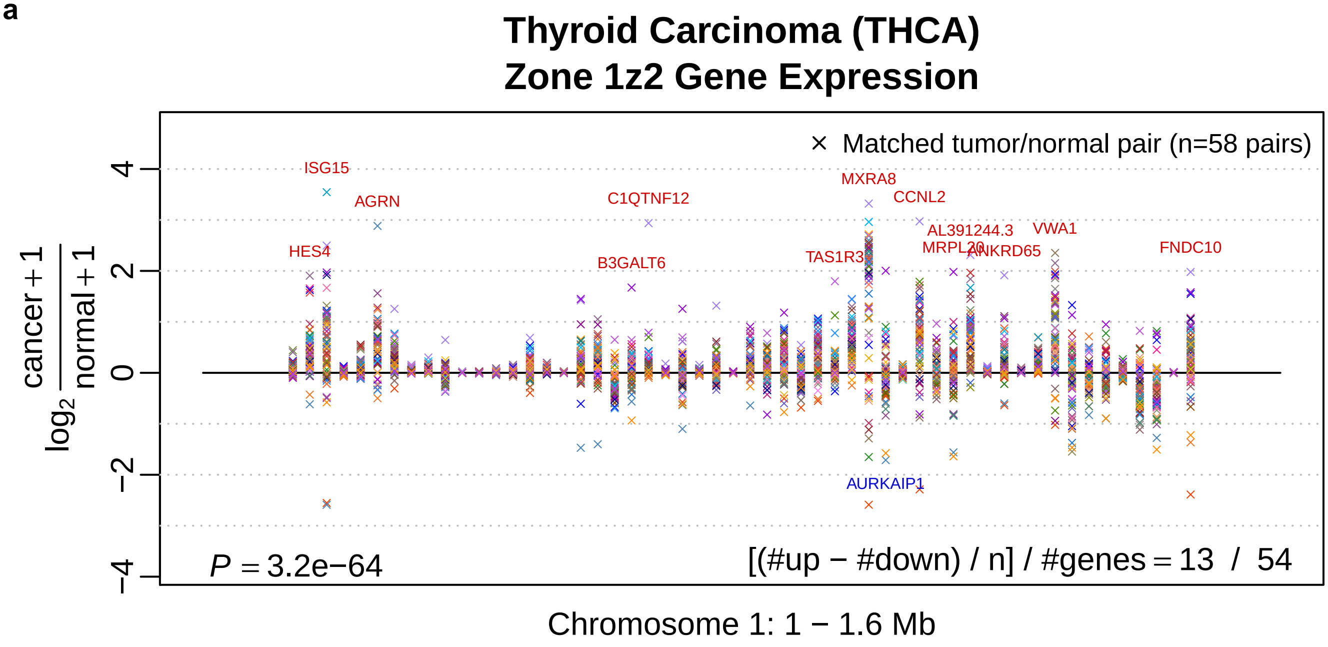

b

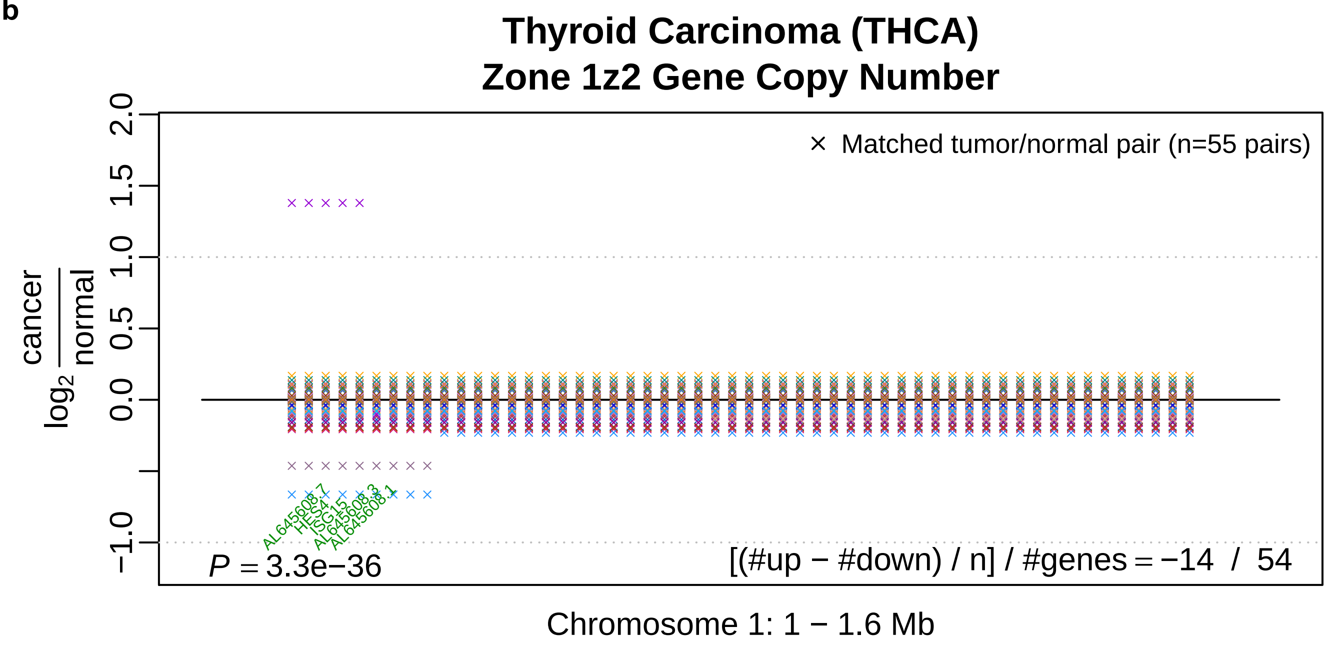

Figure N3.6: The fourth most statistically significant polarized regulation zone in thyroid carcinoma (THCA). Positive zone regulation polarization is not explainable by negative polarization in zone somatic copy number alteration. **a**, The zone is significantly positively (+) polarized in regulation in cancer over matched normal. **b**, The same zone is significantly negatively (-) polarized in somatic copy number alteration in cancer over matched normal.

a

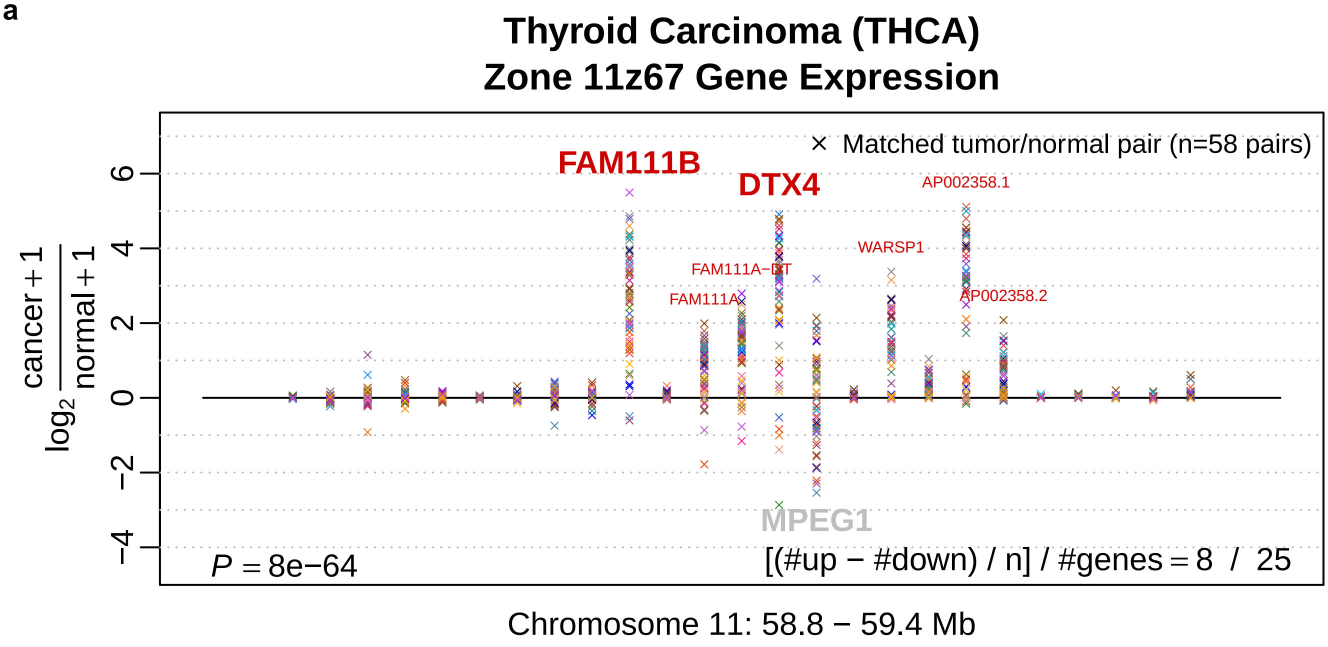

b

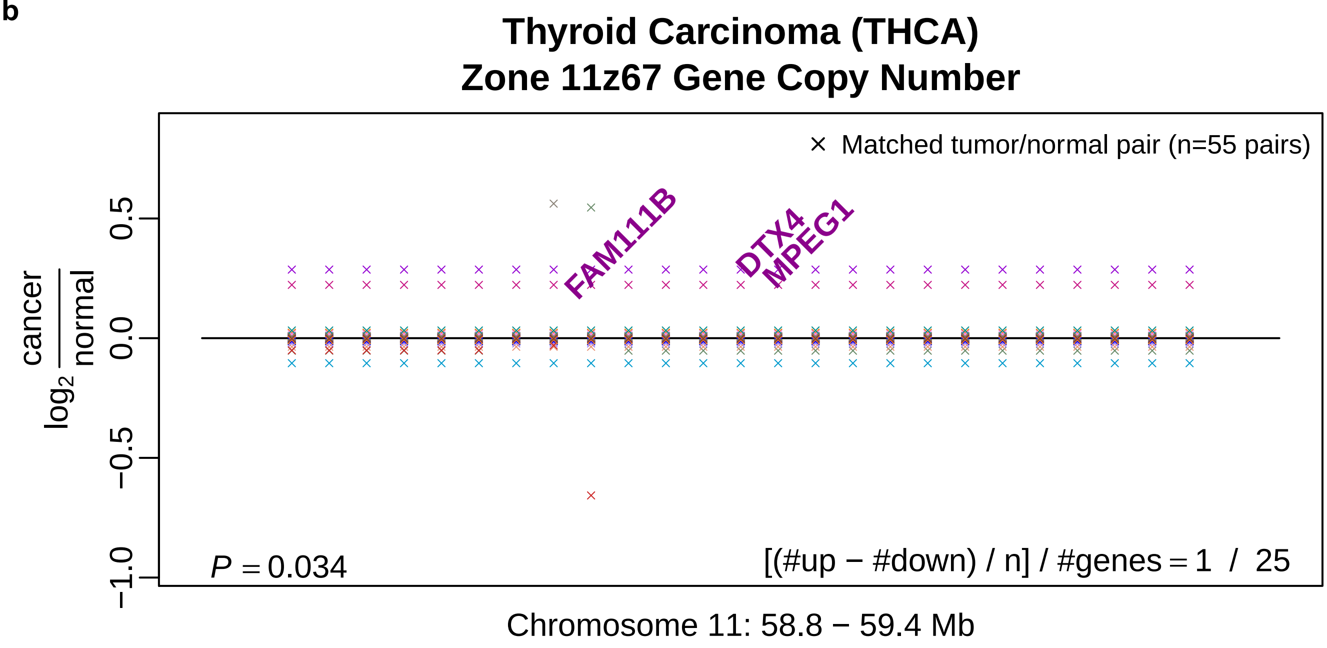

Figure N3.7: The fifth most statistically significant polarized regulation zone in thyroid carcinoma (THCA). Significant zone regulation polarization is barely explained by polarization in zone somatic copy number alteration. **a**, The zone is significantly positively polarized in regulation in cancer over matched normal. **b**, The same zone is weakly positively polarized in somatic copy number alteration in cancer over matched normal.

a

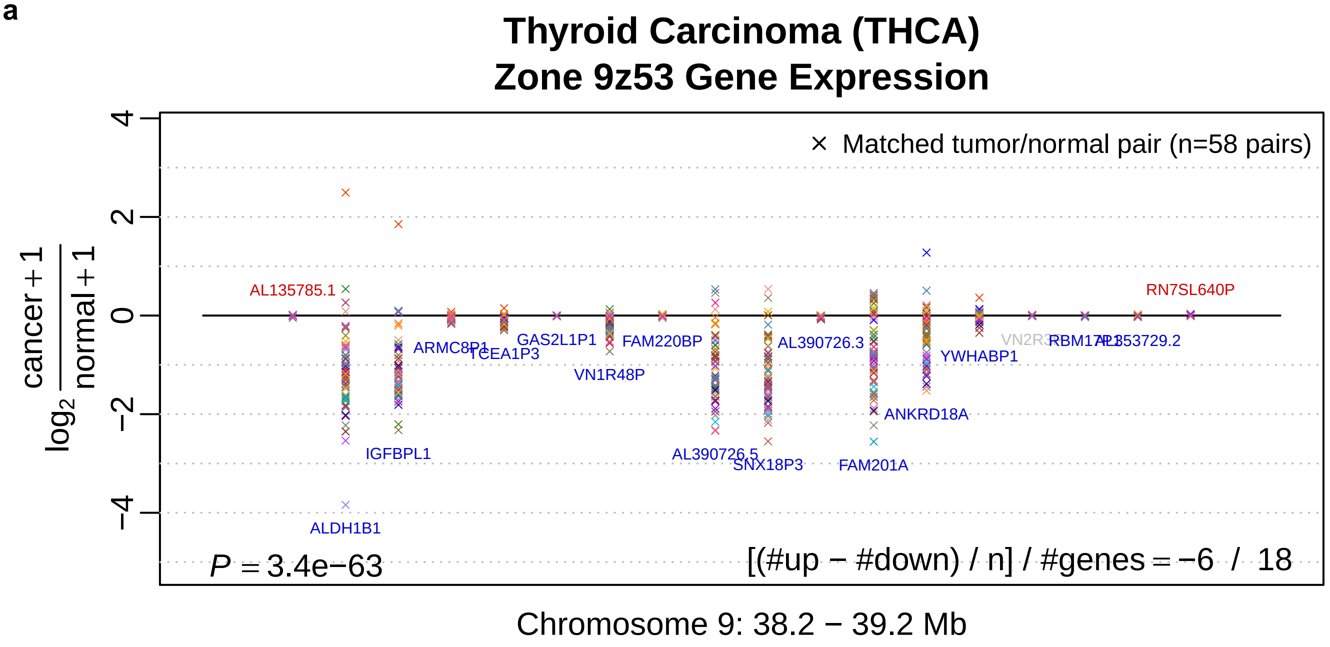

b

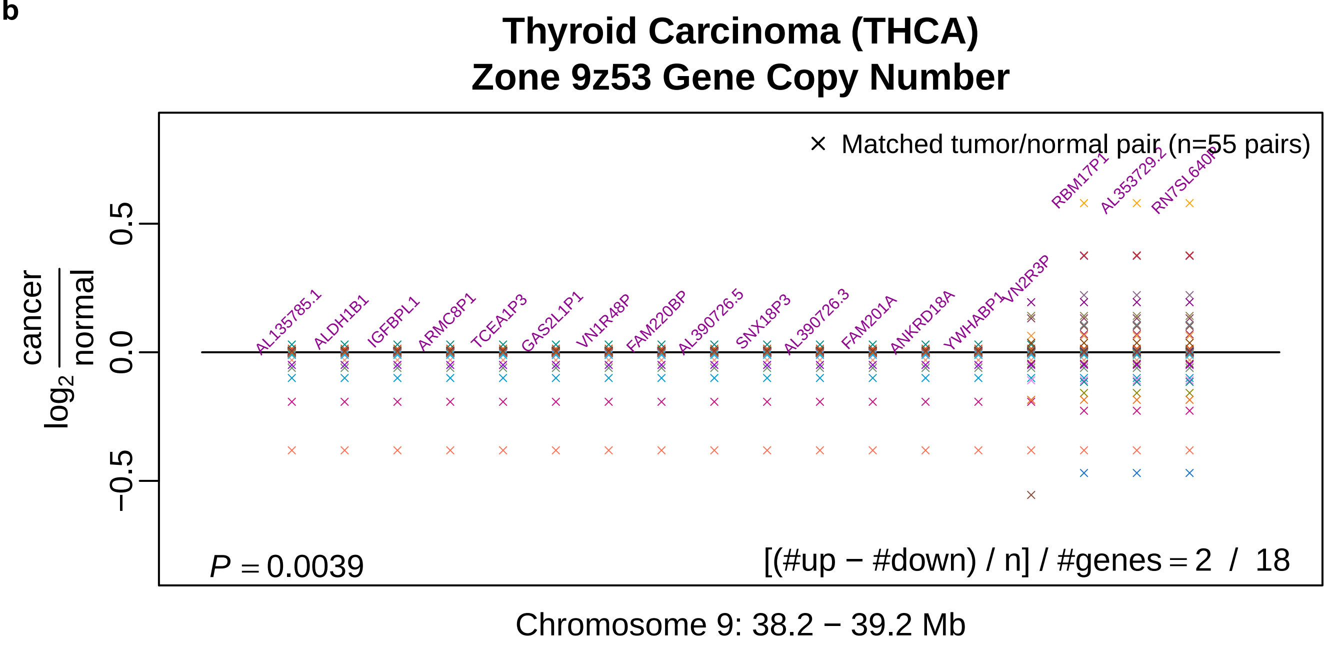

Figure N3.8: The sixth most statistically significant polarized regulation zone in thyroid carcinoma (THCA). Negative zone regulation polarization cannot be explained by positive polarization in zone somatic copy number alteration. **a**, The zone is significantly negatively polarized in regulation in cancer over matched normal. **b**, The same zone is significantly positively polarized in somatic copy number alteration in cancer over matched normal.

a

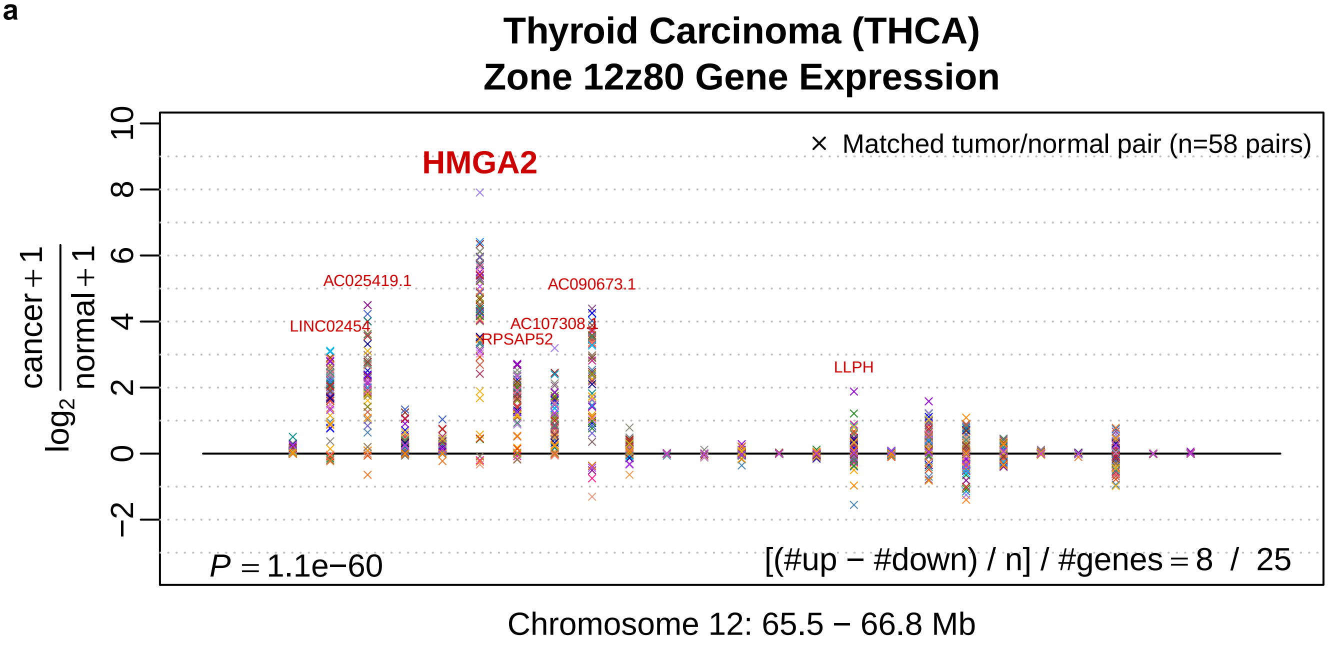

b

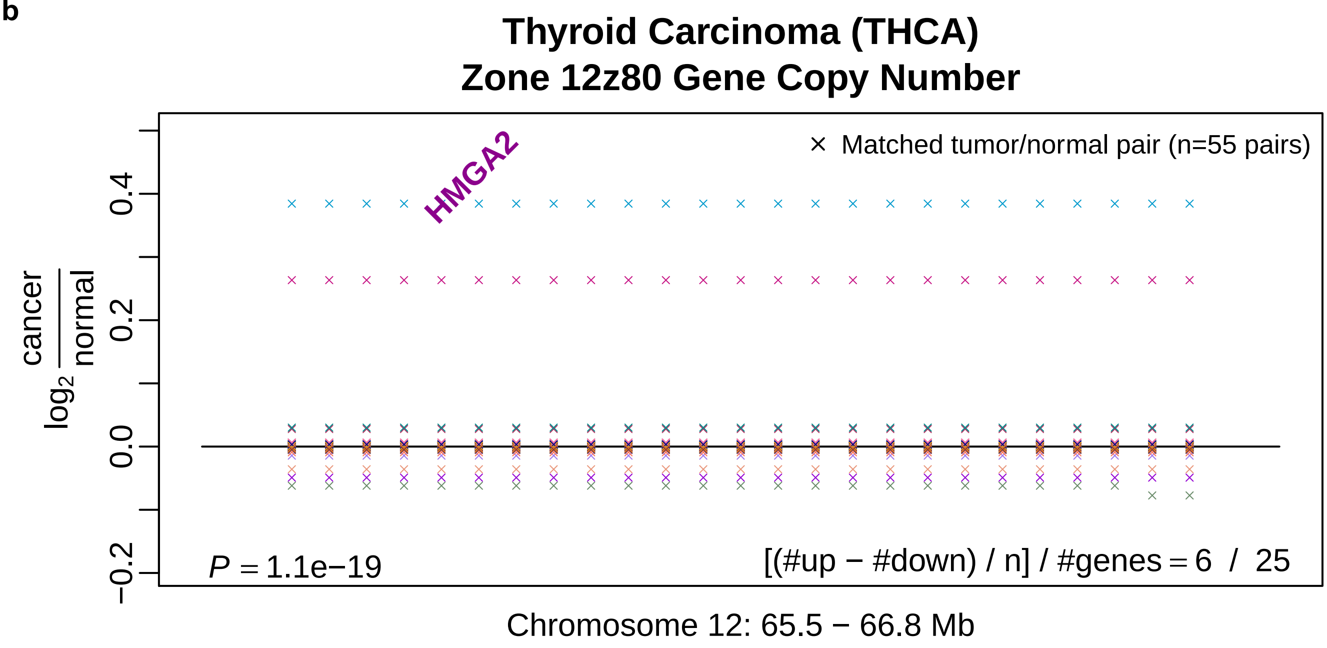

Figure N3.9: The seventh most statistically significant polarized regulation zone in thyroid carcinoma (THCA). Zone regulation polarization is consistent with polarization in zone somatic copy number alteration. **a**, The zone is significantly positively polarized in regulation in cancer over matched normal. **b**, The same zone is also significantly positively polarized in somatic copy number alteration in cancer over matched normal.

a

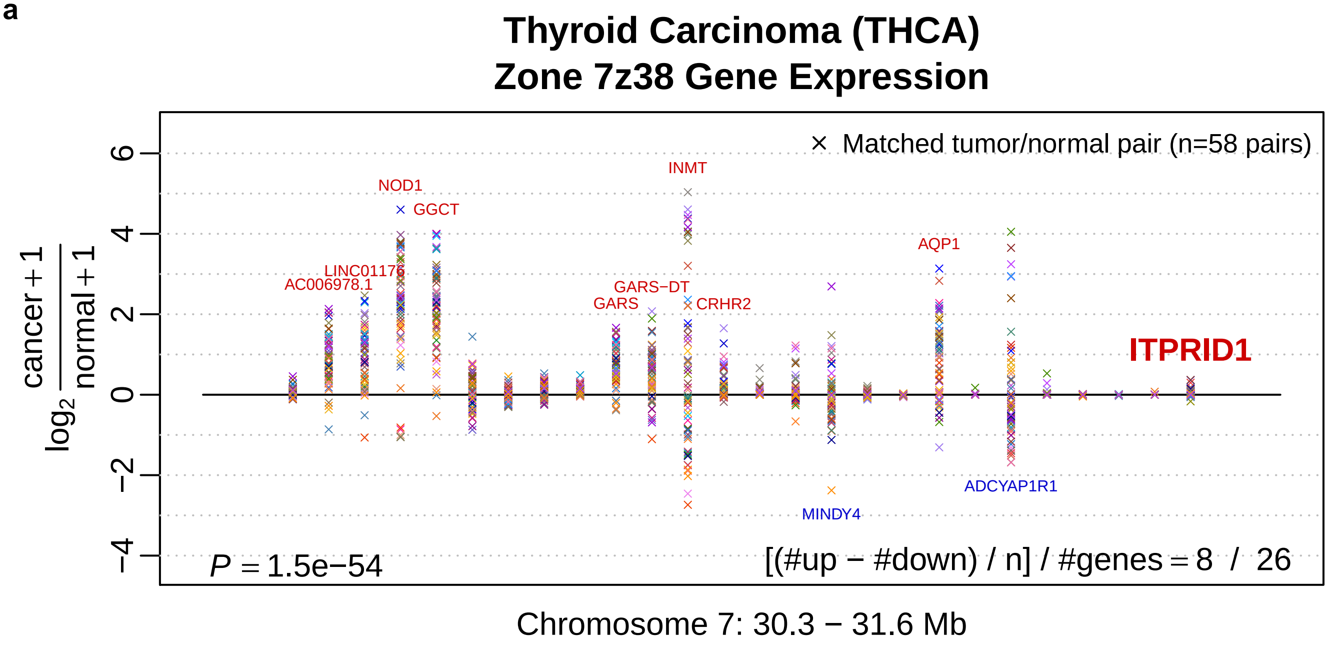

b

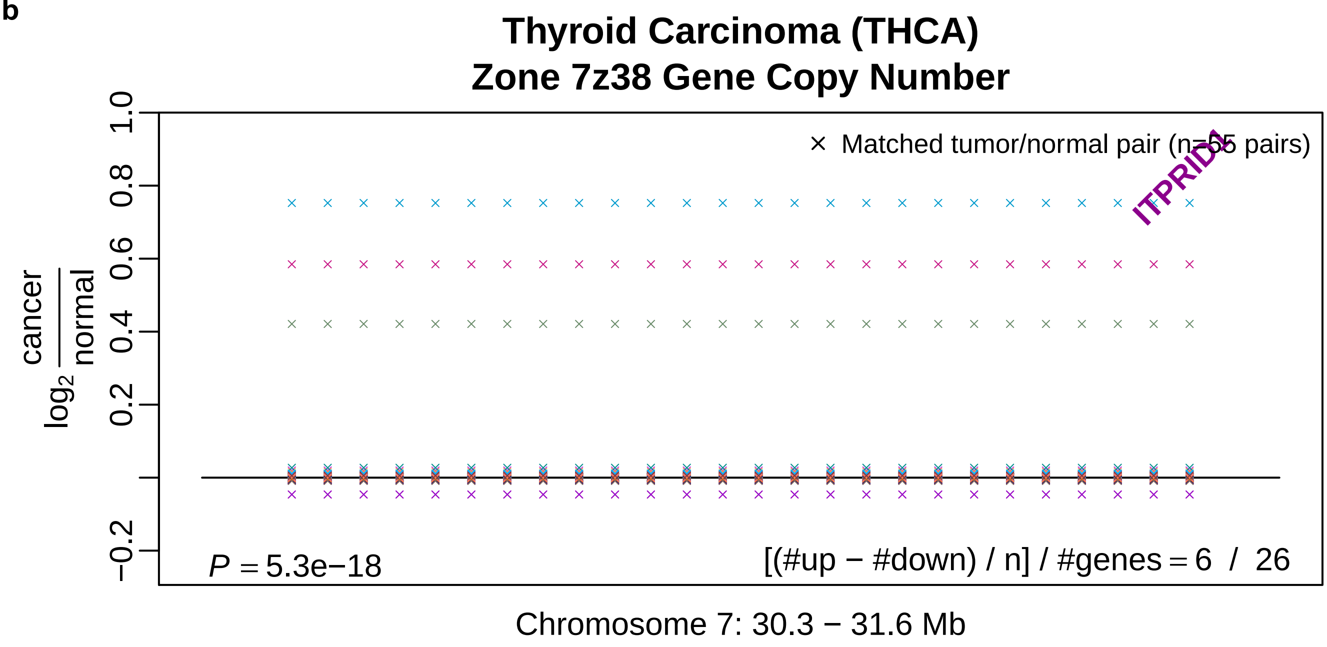

Figure N3.10: The eighth most statistically significant polarized regulation zone in thyroid carcinoma (THCA). Zone regulation polarization is consistent with polarization in zone somatic copy number alteration. **a**, The zone is significantly positively polarized in regulation in cancer over matched normal. **b**, The same zone is also significantly polarized in somatic copy number alteration in cancer over matched normal.

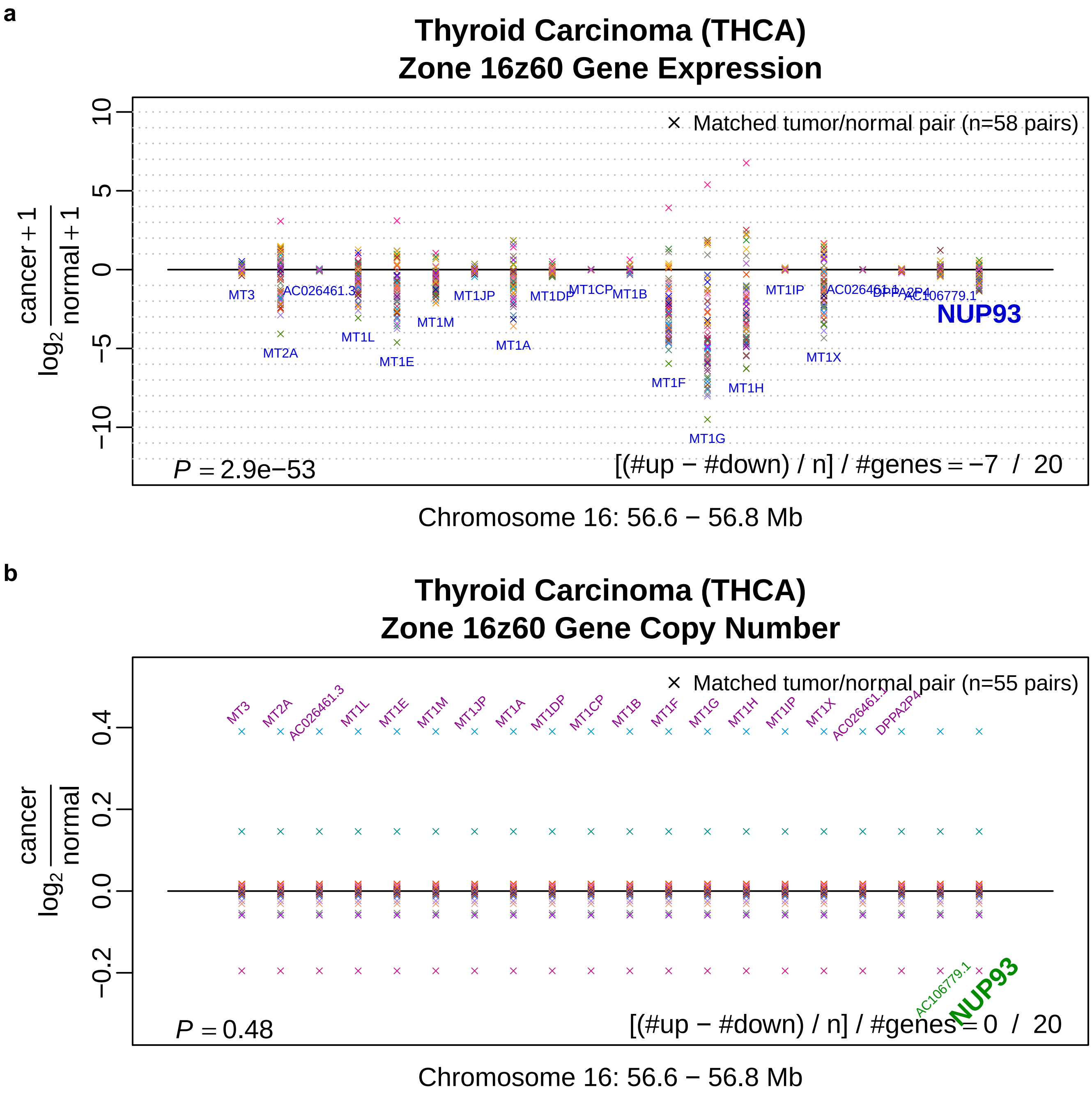

Figure N3.11: The ninth most statistically significant polarized regulation zone in thyroid carcinoma (THCA). Significant zone regulation polarization cannot be explained by insignificant polarization in zone somatic copy number alteration. **a**, The zone is significantly negatively polarized in regulation in cancer over matched normal. **b**, The same zone is insignificantly polarized in somatic copy number alteration in cancer over matched normal.

a

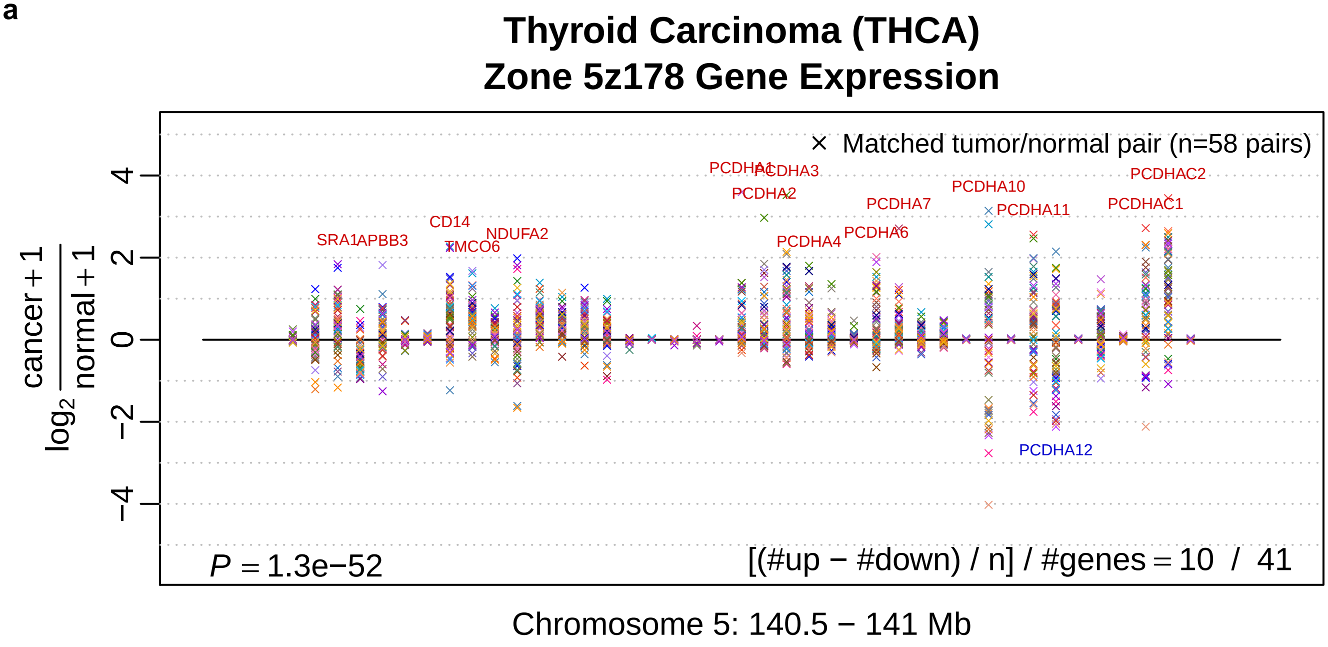

b

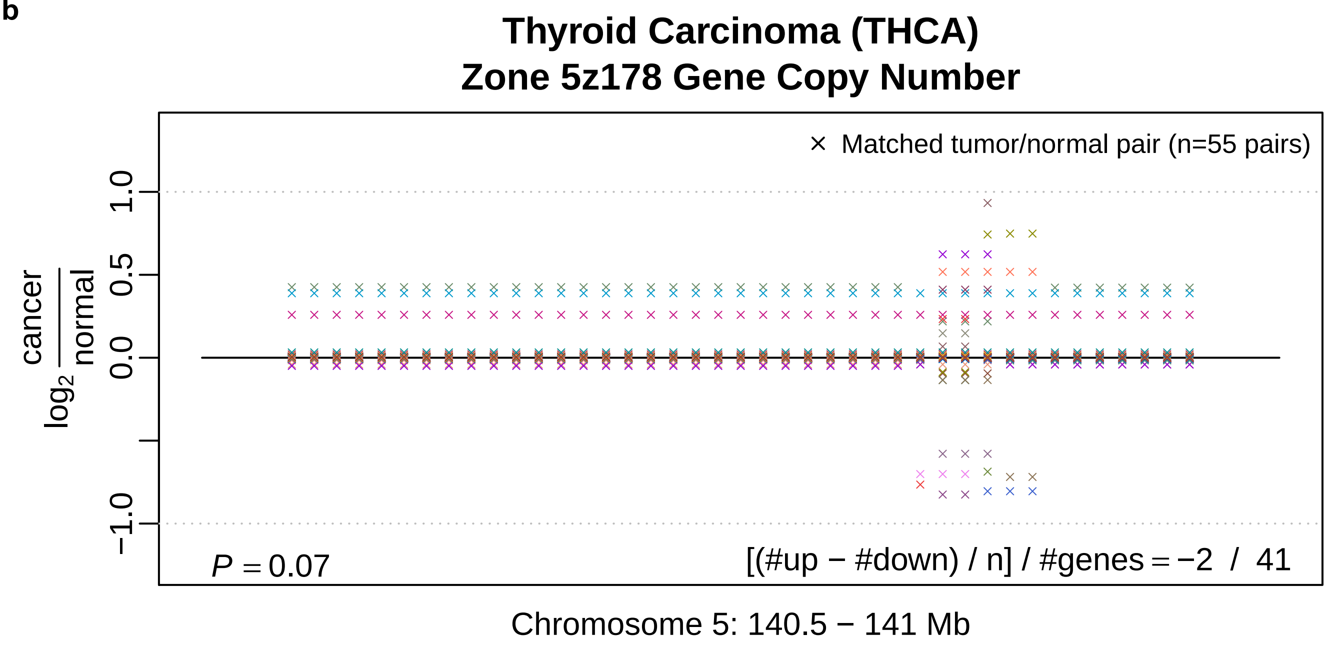

Figure N3.12: The tenth most statistically significant polarized regulation zone in thyroid carcinoma (THCA). Significant zone regulation polarization cannot be explained by insignificant polarization in zone somatic copy number alteration. **a**, The zone is significantly positively polarized in regulation in cancer over matched normal. **b**, The same zone is insignificantly polarized in somatic copy number alteration in cancer over matched normal.

References

Abe, M. and Kufe, D. W. (1993). Characterization of cis-acting elements regulating transcription of the human DF3 breast carcinoma-associated antigen (MUC1) gene. *Proc Natl Acad Sci U S A*, 90(1):282–286.

Fletcher, J. I., Haber, M., Henderson, M. J., and Norris, M. D. (2010). ABC transporters in cancer: more than just drug efflux pumps. *Nat Rev Cancer*, 10(2):147–156.

Jeitany, M., Leroy, C., Tosti, P., Lafitte, M., Le Guet, J., Simon, V., Bonenfant, D., Robert, B., Grillet, F., Mollevi, C., El Messaoudi, S., Otandault, A., Canterel-Thouennon, L., Busson, M., Thierry, A. R., Martineau, P., Pannequin, J., Roche, S., and Sirvent, A. (2018). Inhibition of DDR1-BCR signalling by nilotinib as a new therapeutic strategy for metastatic colorectal cancer. *EMBO Mol Med*, 10(4).

Kufe, D. W. (2013). MUC1-C oncoprotein as a target in breast cancer: activation of signaling pathways and therapeutic approaches. *Oncogene*, 32(9):1073–1081.

Li, G., Barthelemy, A., Feng, G., Gentil-Perret, A., Peoc'h, M., Genin, C., and Tostain, J. (2007). S100A1: a powerful marker to differentiate chromophobe renal cell carcinoma from renal oncocytoma. *Histopathology*, 50(5):642–647.

Pawar, H., Kashyap, M. K., Sahasrabuddhe, N. A., Renuse, S., Harsha, H. C., Kumar, P., Sharma, J., Kandasamy, K., Marimuthu, A., Nair, B., Rajagopalan, S., Maharudraiah, J., Premalatha, C. S., Kumar, K. V. V., Vijayakumar, M., Chaerkady, R., Prasad, T. S. K., Kumar, R. V., Kumar, R. V., and Pandey, A. (2011). Quantitative tissue proteomics of esophageal squamous cell carcinoma for novel biomarker discovery. *Cancer Biol Ther*, 12(6):510–522.

Pohl, S., Scott, R., Arfuso, F., Perumal, V., and Dharmarajan, A. (2015). Secreted frizzled-related protein 4 and its implications in cancer and apoptosis. *Tumour Biol*, 36(1):143–152.

Repana, D., Nulsen, J., Dressler, L., Bortolomeazzi, M., Kuppili Venkata, S., Tourna, A., Yakovleva, A., Palmieri, T., and Ciccarelli, F. D. (2018). The network of cancer genes (NCG): a comprehensive catalogue of known and candidate cancer genes from cancer sequencing screens. *bioRxiv*.

Taub, R., Kirsch, I., Morton, C., Lenoir, G., Swan, D., Tronick, S., Aaronson, S., and Leder, P. (1982). Translocation of the c-myc gene into the immunoglobulin heavy chain locus in human Burkitt lymphoma and murine plasmacytoma cells. *Proc Natl Acad Sci U S A*, 79(24):7837–7841.

Taylor-Papadimitriou, J., Burchell, J. M., Graham, R., and Beatson, R. (2018). Latest developments in MUC1 immunotherapy. *Biochem Soc Trans*, 46(3):659–668.

Uhlen, M., Fagerberg, L., Hallstrom, B. M., Lindskog, C., Oksvold, P., Mardinoglu, A., Sivertsson, A., Kampf, C., Sjostedt, E., Asplund, A., Olsson, I., Edlund, K., Lundberg, E., Navani, S., Szigyarto, C. A.-K., Odeberg, J., Djureinovic, D., Takanen, J. O., Hober, S., Alm, T., Edqvist, P.-H., Berling, H., Tegel, H., Mulder, J., Rockberg, J., Nilsson, P., Schwenk, J. M., Hamsten, M., von Feilitzen, K., Forsberg, M., Persson, L., Johansson, F., Zwahlen, M., von Heijne, G., Nielsen, J., and Ponten, F. (2015). Proteomics. Tissue-based map of the human proteome. *Science*, 347(6220):1260419.

Wu, Y., Miyamoto, T., Li, K., Nakagomi, H., Sawada, N., Kira, S., Kobayashi, H., Zakohji, H., Tsuchida, T., Fukazawa, M., Araki, I., and Takeda, M. (2011). Decreased expression of the epithelial Ca2+ channel TRPV5 and TRPV6 in human renal cell carcinoma associated with vitamin D receptor. *J Urol*, 186(6):2419–2425.

Yang, S.-B., Chen, X., Wu, B.-Y., Wang, M.-W., Cai, C.-H., Cho, D.-B., Chong, J., Li, P., Tang, S.-G., and Yang, P.-C. (2009). Immunoglobulin kappa and immunoglobulin lambda are required for expression of the anti-apoptotic molecule Bcl-xL in human colorectal cancer tissue. *Scand J Gastroenterol*, 44(12):1443–1451.

Zaretsky, J. Z., Weiss, M., Tsarfaty, I., Hareuveni, M., Wreschner, D. H., and Keydar, I. (1990). Expression of genes coding for pS2, c-erbB2, estrogen receptor and the H23 breast tumor-associated antigen. A comparative analysis in breast cancer. *FEBS Lett*, 265(1-2):46–50.
