# Supplementary material for: Efficient weighted univariate clustering maps outstanding dysregulated genomic zones in human cancers
Source: Bioinformatics. 2020 Jul 3;36(20):5027–36. doi: 10.1093/bioinformatics/btaa613 (PMC7755420; doi:10.1093/bioinformatics/btaa613)
Supplement: btaa613_Supplementary_Data [file btaa613_supplementary_data.zip › SuppNote-N4.pdf]

Supplementary Note N4: Top somatic copy number alteration zones match known cancer aneuploidy

List of Figures

N4.1 BLCA top polarized copy number zone N4-6
N4.2 BRCA top polarized copy number zone N4-7
N4.3 CHOL top polarized copy number zone N4-8
N4.4 COAD top polarized copy number zone N4-9
N4.5 ESCA top polarized copy number zone N4-10
N4.6 HNSC top polarized copy number zone N4-11
N4.7 KICH top polarized copy number zone N4-12
N4.8 KIRC top polarized copy number zone N4-13
N4.9 KIRP top polarized copy number zone N4-14
N4.10 LIHC top polarized copy number zone N4-15
N4.11 LUAD top polarized copy number zone N4-16
N4.12 LUSC top polarized copy number zone N4-17
N4.13 PRAD top polarized copy number zone N4-18
N4.14 READ top polarized copy number zone N4-19
N4.15 STAD top polarized copy number zone N4-20
N4.16 THCA top polarized copy number zone N4-21
N4.17 UCEC top polarized copy number zone N4-22

The top SCNA zone of bladder urothelial carcinoma (BLCA) chr5:68.7-70.4 Mb (Fig N4.1) overlaps cytogenetic bands 5q13.1 and 5q13.2. The zone is negatively polarized, consistent with regions along arm 5q being frequently deleted in invasive bladder cancer (Kram et al., 2001).

In breast invasive carcinoma (BRCA), the top zone chr1:160.1-160.7 Mb (Fig N4.2) in 1q23 is positively polarized, which is supported by previous results that copy number gains of arm 1q were the most frequent aberration in 67% of 55 primary unselected primary breast carcinomas (Tirkkonen et al., 1998). That study reported the next most frequent gains occurred in 8q (49%); other recurrent gains were found at 16p, 5p12-14, 19q, 11q13-14, 17q12, 17q22-24, 19p, and 20q13 (> 18%); losses were found in greater than 18% at 8p, 16q, 13q, 17p, 9p, Xq, 6q, 11q, and 18q (Tirkkonen et al., 1998). All these abnormalities are reproduced by the SCNA zone polarity map of BRCA (Fig S2.2). In uterine corpus endometrial carcinoma (UCEC), the top zone chr1:152.8-153.8 Mb (Fig N4.17) is positively polarized in 1q21.3. Endometrial carcinoma in 109 Black or African American (BOAA) patients showed significant 1q amplification, but not observed in 374 Caucasian women from a recent study (Guttery et al., 2018). It used a UCEC sample collection from TCGA much larger in sample size than this study, but unmatched normal samples were used. In contrast, our study included 22 pairs of matched tumor-normal samples, in which only two are from BOAA, 11 are from Caucasian women, and the race of the remaining nine pairs is unknown. As our study uses matched pairs, it is not subject to the influence of germline copy number alteration. Therefore, it is possible that 1q amplification is indeed a strong marker of SCNA in UCEC regardless of race.

In cholangiocarcinoma (CHOL), the top zone chr14:106.1-106.9 Mb (Fig N4.3) in 14q, negatively polarized, coincides with one of the most exclusively deleted cytogenetic bands 14q24.1-14q32.33 in 52.6% out of 53 patients with intrahepatic cholangiocarcinomas (Dalmasso et al., 2015). The same study marked 1p, 3p and 14q as exclusive deletion and 1q, 7p, 7q and 8q as exclusive amplification. All these arms see consistent polarization except 45-125 Mb being positively polarized in 1p (Fig S2.3).

Among colon adenocarcinoma (COAD) genomic zones, the negatively polarized zone 15:24.3-25.2 Mb (Fig N4.4), residing in 15q11.2, is the most significant. The loss of 15q11-q21 is a strong marker of the progression of benign adenoma to malignant carcinoma in 194 colorectal tumor samples (Hermsen et al.,

2002). Numerous studies of colorectal cancers have observed the chromosomal copy number gain of 7, 8q, 13q, and 20 and loss of 4, 8p, 17p, 18, and 20p (Sheffer et al., 2009). All these changes are reproduced in our analysis (Fig S2.4) In rectum adenocarcinoma (READ), chr17:4.5-5.2 Mb (Fig N4.14) is negatively polarized in 17p13.2, belonging to 17p which is subject to most common partial losses (Bardi et al., 1995). This region contains a known oncogene *USP6*, which is implicated in colon cancer invasion and metastasis (Zeng et al., 2018). Both zones (15:24.3-25.2 Mb and chr17:4.5-5.2 Mb) are statistically significantly negatively polarized in COAD and READ.

In esophageal carcinoma (ESCA), chr2:130.7-132.6 Mb (Fig N4.5) in 2q21 is the most significant SCNA zone, positively polarized and containing 62 genes. It is the weakest top zone among all cancer types, overrepresented at about 1.3 times in eight pairs of matched tumor-normal samples. This zone is in a larger context where almost the entire chromosome 2 is positively polarized. This finding largely disagrees with earlier studies that did not use matched tumor-normal pairs. In a study of 17 human esophageal tumor samples (Pack et al., 1999), it documented frequent chromosomal losses in 2q (100%), 3p (100%), 13q (100%), Xq (94%), 4 (82%), 5q (82%) weak, 18q (76%), 9p (76%), 6q (70%), 12q (70%), 14q (65%), 11q (59%), and 1p (53%); interstitial deletions on 1p, 3p, 5q, 6q, 11q (near telomere), and 12q; chromosomal gains in 19 (100%), 20q (94%), 22 (94%), 16p (65%), 17 (59%), 12q (59%), 8q (53%), 9q (53%), and 3q (50%). Our results indicate opposite polarity in 2q, 13q, 12q, 14q, 12q, 22, and 16p.

In head and neck squamous cell carcinoma (HNSC), chr5:141-141.5 Mb (Fig N4.6) in 5q31.3 is the most polarized zone, negatively polarized. This is in a context where the entire long arm 5q is negatively polarized. 5q deletion was indeed observed independently in 50% of 50 primary HNSCs (Bockmuhl et al., 1998).

In kidney chromophobe (KICH), the top zone chr1:1-1.6 Mb (Fig N4.7) in 1p36.33 is negatively polarized. It concurs with chromosome 1 being one of the most common losses to KICH genome (Speicher et al., 1994). Most common losses are chromosome 1, 2, 6, 10, 13, 17, and 21 (Speicher et al., 1994), and most common gains are chromosome 4, 7, 15, 19, and 20 (Sperga et al., 2013). They are fully represented by polarized zones in KICH. In kidney renal clear cell carcinoma (KIRC), the top zone chr3:26.4-27.9 Mb (Fig N4.8) in 3p24.1 is negatively polarized, in agreement with 3p deletion being the most common anomaly in KIRC (Kovacs et al., 1988). In kidney renal papillary cell carcinoma (KIRP), the top zone chr7:62.8–64.9 Mb (Fig N4.9), overlapping 7q11.1 and 7q11.21 and next to the centromere on the long arm, is positively polarized in SCNA. Second only to 7p (56%), 7q (44%) copy number gain is frequently observed in KIRP (Jiang et al., 1998).

In liver hepatocellular carcinoma (LIHC), the top zone chr1:152.8-153.8 Mb (Fig N4.10) in 1q21.3 is positively polarized. Indeed, 1q21 is the most commonly amplified region in LIHC (Ma et al., 2008). This region contains a known cancer gene *S100A7*, though its involvement in liver cancer is not yet entirely understood.

In lung adenocarcinoma (LUAD), the positive polarization of the top zone chr1:154.9- 155.4 Mb (Fig N4.11), crossing 1q21.3 and 1q22, is supported by 1q overrepresentation—the most common alteration in LUAD. The top zone overlaps 1q22-q23, where the peak incidence occurred in 73% of 83 primary LUADs (Goeze et al., 2002). This zone includes the oncogene *MUC1*.

In lung squamous cell carcinoma (LUSC), the top zone chr5:141-141.5 Mb (Fig N4.12) in 5q31.3 is negatively polarized. 5q in non-small cell lung carcinoma frequently displayed loss of heterozygosity, thus consistent with our result. The nearby 5q31.1 is a region subject to highly frequent deletion (Mendes-da Silva et al., 2000).

In prostate adenocarcinoma (PRAD), the top zone chr8:10.4-11.5 Mb (Fig N4.13) is found negatively polarized in 8p23.1, belonging to the 8p23 band at which 29% of 60 cases of human prostate cancer showed loss of heterozygosity (Perinchery et al., 1999).

In stomach adenocarcinoma (STAD), the most polarized zone chrY:16.5-87.1 Mb (Fig N4.15) covers almost the entire long arm of Yq, overlapping Yq11.22, Yq11.23, and Yq12. The zone is among the most heavily negatively polarized, consistent with previous findings that deletion on the Y-chromosome is the most prominent cytogenetic band abnormality in a gastric cancer study (Ochi et al., 1986).

In thyroid carcinoma (THCA), the top zone chr22:26.1-27.3 Mb (Fig N4.16) in 22q12.1 is negatively polarized. Indeed, 22q contains the most frequent (41%) allelic losses in follicular thyroid carcinoma (Kitamura et al., 2001; Chai et al., 2016).

References

Bardi, G., Sukhikh, T., Pandis, N., Fenger, C., Kronborg, O., and Heim, S. (1995). Karyotypic characterization of colorectal adenocarcinomas. *Genes Chromosomes Cancer*, 12(2):97–109.

Bockmuhl, U., Wolf, G., Schmidt, S., Schwendel, A., Jahnke, V., Dietel, M., and Petersen, I. (1998). Genomic alterations associated with malignancy in head and neck cancer. *Head Neck*, 20(2):145–151.

Chai, L., Li, J., and Lv, Z. (2016). An integrated analysis of cancer genes in thyroid cancer. *Oncol Rep*, 35(2):962–970.

Dalmasso, C., Carpentier, W., Guettier, C., Camilleri-Broet, S., Borelli, W. V., Campos Dos Santos, C. R., Castaing, D., Duclos-Vallee, J.-C., and Broet, P. (2015). Patterns of chromosomal copy-number alterations in intrahepatic cholangiocarcinoma. *BMC Cancer*, 15:126.

Goeze, A., Schluns, K., Wolf, G., Thasler, Z., Petersen, S., and Petersen, I. (2002). Chromosomal imbalances of primary and metastatic lung adenocarcinomas. *J Pathol*, 196(1):8–16.

Guttery, D. S., Blighe, K., Polymeros, K., Symonds, R. P., Macip, S., and Moss, E. L. (2018). Racial differences in endometrial cancer molecular portraits in The Cancer Genome Atlas. *Oncotarget*, 9(24):17093–17103.

Hermsen, M., Postma, C., Baak, J., Weiss, M., Rapallo, A., Sciutto, A., Roemen, G., Arends, J.-W., Williams, R., Giaretti, W., De Goeij, A., and Meijer, G. (2002). Colorectal adenoma to carcinoma progression follows multiple pathways of chromosomal instability. *Gastroenterology*, 123(4):1109–1119.

Jiang, F., Richter, J., Schraml, P., Bubendorf, L., Gasser, T., Sauter, G., Mihatsch, M. J., and Moch, H. (1998). Chromosomal imbalances in papillary renal cell carcinoma: genetic differences between histological subtypes. *Am J Pathol*, 153(5):1467–1473.

Kitamura, Y., Shimizu, K., Ito, K., Tanaka, S., and Emi, M. (2001). Allelotyping of follicular thyroid carcinoma: frequent allelic losses in chromosome arms 7q, 11p, and 22q. *J Clin Endocrinol Metab*, 86(9):4268–4272.

Kovacs, G., Erlandsson, R., Boldog, F., Ingvarsson, S., Muller-Brechlin, R., Klein, G., and Sumegi, J. (1988). Consistent chromosome 3p deletion and loss of heterozygosity in renal cell carcinoma. *Proc Natl Acad Sci U S A*, 85(5):1571–1575.

Kram, A., Li, L., Zhang, R. D., Yoon, D. S., Ro, J. Y., Johnston, D., Grossman, H. B., Scherer, S., and Czerniak, B. (2001). Mapping and genome sequence analysis of chromosome 5 regions involved in bladder cancer progression. *Lab Invest*, 81(7):1039–1048.

Ma, N.-F., Hu, L., Fung, J. M., Xie, D., Zheng, B.-J., Chen, L., Tang, D.-J., Fu, L., Wu, Z., Chen, M., Fang, Y., and Guan, X.-Y. (2008). Isolation and characterization of a novel oncogene, amplified in liver cancer 1, within a commonly amplified region at 1q21 in hepatocellular carcinoma. *Hepatology*, 47(2):503–510.

Mendes-da Silva, P., Moreira, A., Duro-da Costa, J., Matias, D., and Monteiro, C. (2000). Frequent loss of heterozygosity on chromosome 5 in non-small cell lung carcinoma. *Mol Pathol*, 53(4):184–187.

Ochi, H., Douglass, H. O. J., and Sandberg, A. A. (1986). Cytogenetic studies in primary gastric cancer. *Cancer Genet Cytogenet*, 22(4):295–307.

Pack, S. D., Karkera, J. D., Zhuang, Z., Pak, E. D., Balan, K. V., Hwu, P., Park, W. S., Pham, T., Ault, D. O., Glaser, M., Liotta, L., Detera-Wadleigh, S. D., and Wadleigh, R. G. (1999). Molecular cytogenetic fingerprinting of esophageal squamous cell carcinoma by comparative genomic hybridization reveals a consistent pattern of chromosomal alterations. *Genes Chromosomes Cancer*, 25(2):160–168.

Perinchery, G., Bukurov, N., Nakajima, K., Chang, J., Hooda, M., Oh, B. R., and Dahiya, R. (1999). Loss of two new loci on chromosome 8 (8p23 and 8q12-13) in human prostate cancer. *Int J Oncol*, 14(3):495–500.

Sheffer, M., Bacolod, M. D., Zuk, O., Giardina, S. F., Pincas, H., Barany, F., Paty, P. B., Gerald, W. L., Notterman, D. A., and Domany, E. (2009). Association of survival and disease progression with chromosomal instability: a genomic exploration of colorectal cancer. *Proc Natl Acad Sci U S A*, 106(17):7131–7136.

Speicher, M. R., Schoell, B., du Manoir, S., Schrock, E., Ried, T., Cremer, T., Storkel, S., Kovacs, A., and Kovacs, G. (1994). Specific loss of chromosomes 1, 2, 6, 10, 13, 17, and 21 in chromophobe renal cell carcinomas revealed by comparative genomic hybridization. *Am J Pathol*, 145(2):356–364.

Sperga, M., Martinek, P., Vanecek, T., Grossmann, P., Bauleth, K., Perez-Montiel, D., Alvarado-Cabrero, I., Nevidovska, K., Lietuvietis, V., Hora, M., Michal, M., Petersson, F., Kuroda, N., Suster, S., Branzovsky, J., and Hes, O. (2013). Chromophobe renal cell carcinoma–chromosomal aberration variability and its relation to paner grading system: an array CGH and FISH analysis of 37 cases. *Virchows Arch*, 463(4):563–573.

Tirkkonen, M., Tanner, M., Karhu, R., Kallioniemi, A., Isola, J., and Kallioniemi, O. P. (1998). Molecular cytogenetics of primary breast cancer by CGH. *Genes Chromosomes Cancer*, 21(3):177–184.

Zeng, H., Yuan, F., Mi, Y., Xian, G., Qin, C., and Zhang, D. (2018). As an independent prognostic factor, usp6 promotes the invasion and metastasis of colon cancer. *Biochem Biophys Res Commun*, 505(3):816–822.

Legend used in the top genomic zones

Each ‘x’ marks one patient with a pair of matched tumor-normal samples. Colors of the ‘x’ marks indicate different patients. The horizontal location of ‘x’ represents a unique gene.

Genes within a zone are ordered by their start genomic coordinates. They are equally spaced in the visualization within the zone, not linearly proportional to their genomic distance.

Known cancer genes from COSMIC Cancer Gene Census (CGC) version 87 and Network of Cancer Genes 6 are marked along the chromosomes. Six cancer loci from CGC—*IGH@*, *IGK@*, *IGL@*, *TRA@*, *TRB@*, and *TRD@*—are not marked, as they are not genes and thus no expression data is available. Those are highly differential are marked in bold font.

a, The name of a gene is marked at its relative position only if its copy number changed substantially between tumor and matched normal tissues. The color of a gene name text is purple/green for amplification/deletion in somatic copy number in cancer versus normal.

b, The name of a gene is marked at its relative position only if its expression changed substantially between tumor and matched normal tissues. The color of a gene name text is red/blue for up-/down-regulation in cancer versus normal.

a

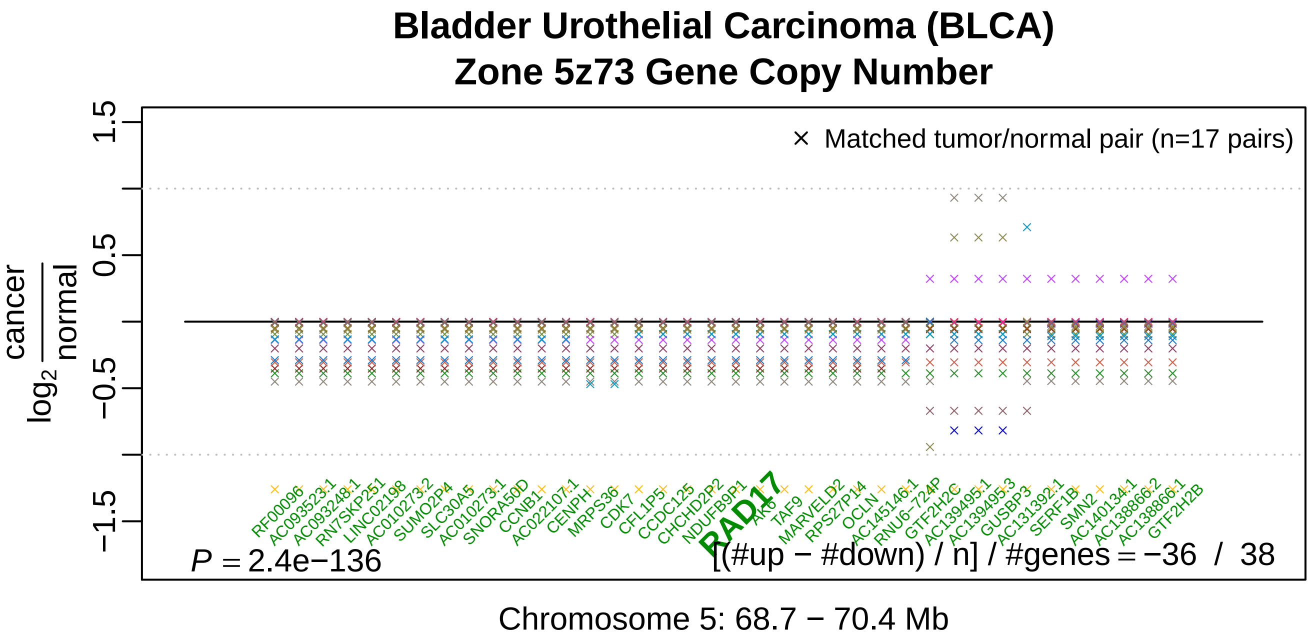

b

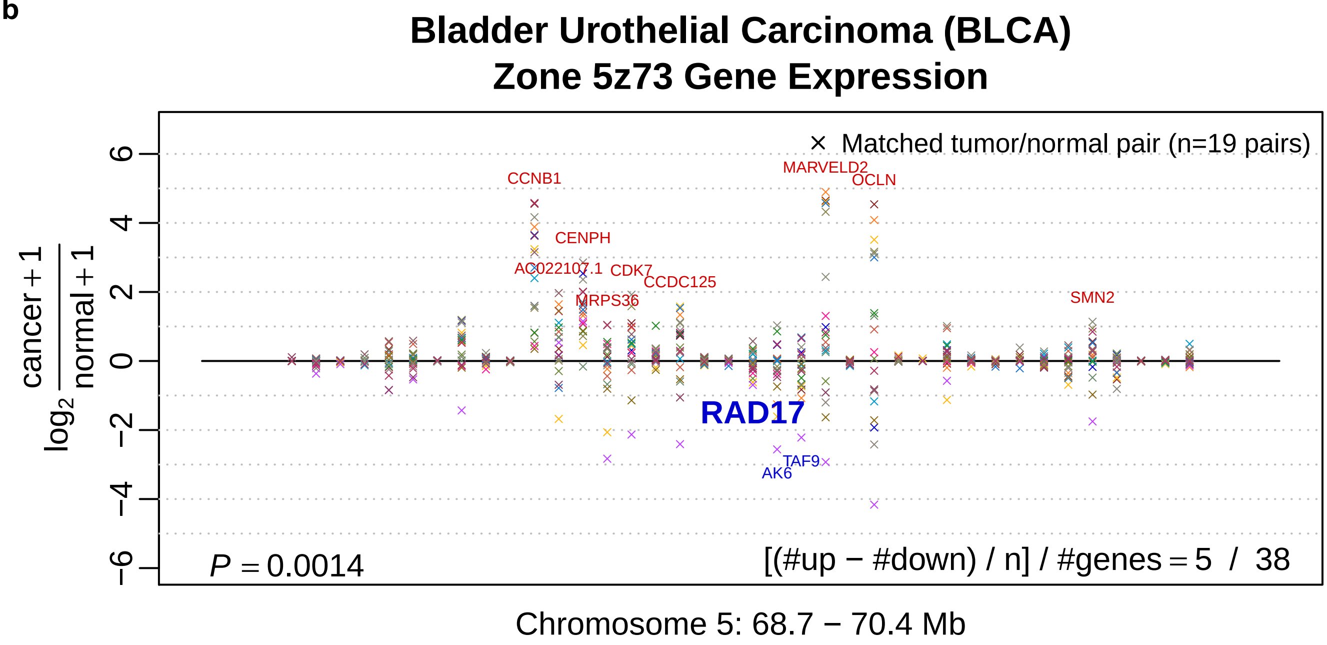

Figure N4.1: The most statistically significant polarized somatic copy number zone in BLCA. a, The somatic copy number log ratio of cancer to normal for each gene within the zone in each patient. **b**, The gene expression log ratio of cancer to normal for each gene within the zone in each patient. See the full legend on page 5.

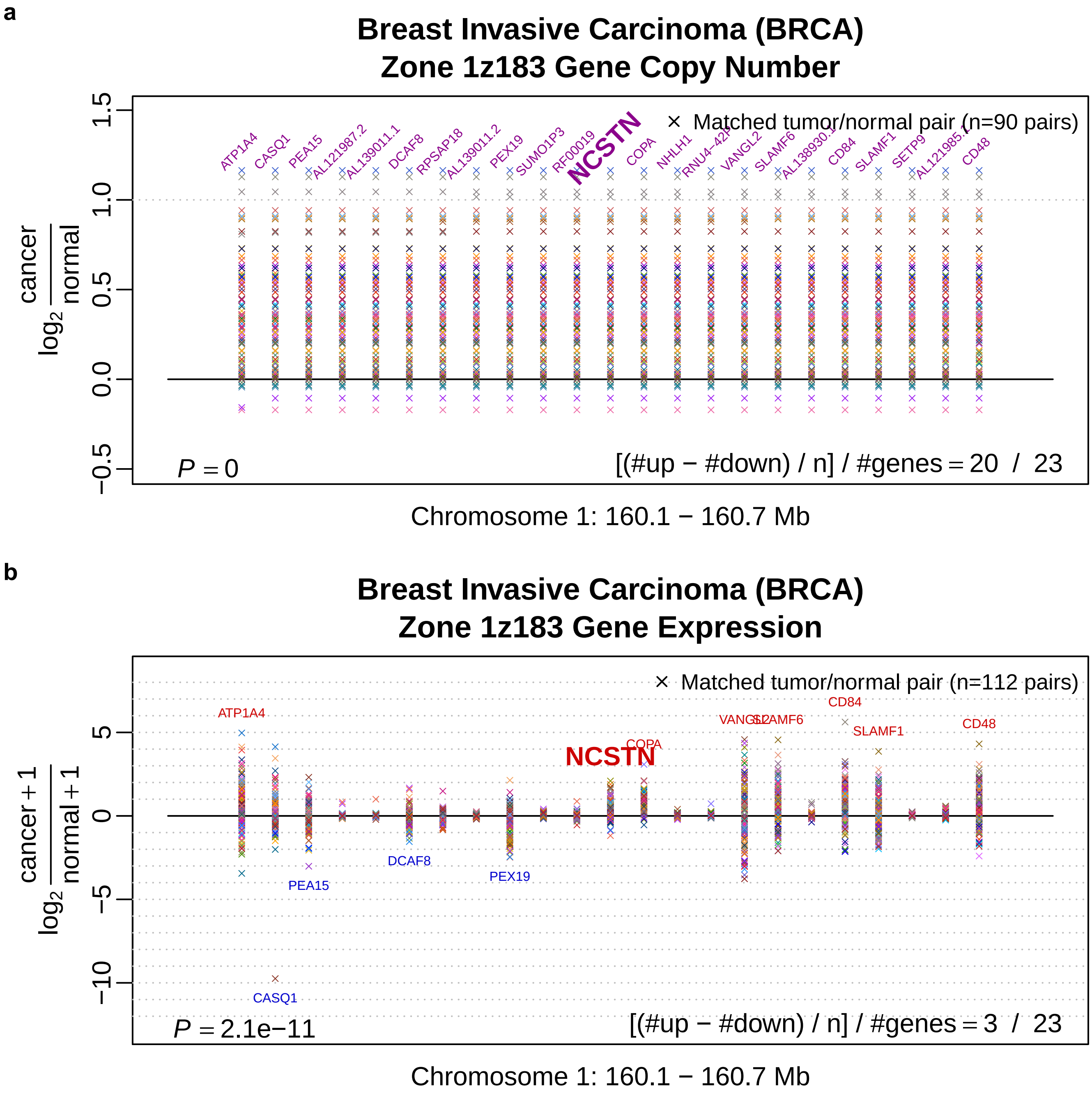

Figure N4.2: The most statistically significant polarized somatic copy number zone in BRCA. a, The somatic copy number log ratio of cancer to normal for each gene within the zone in each patient. **b**, The gene expression log ratio of cancer to normal for each gene within the zone in each patient. See the full legend on page 5.

a

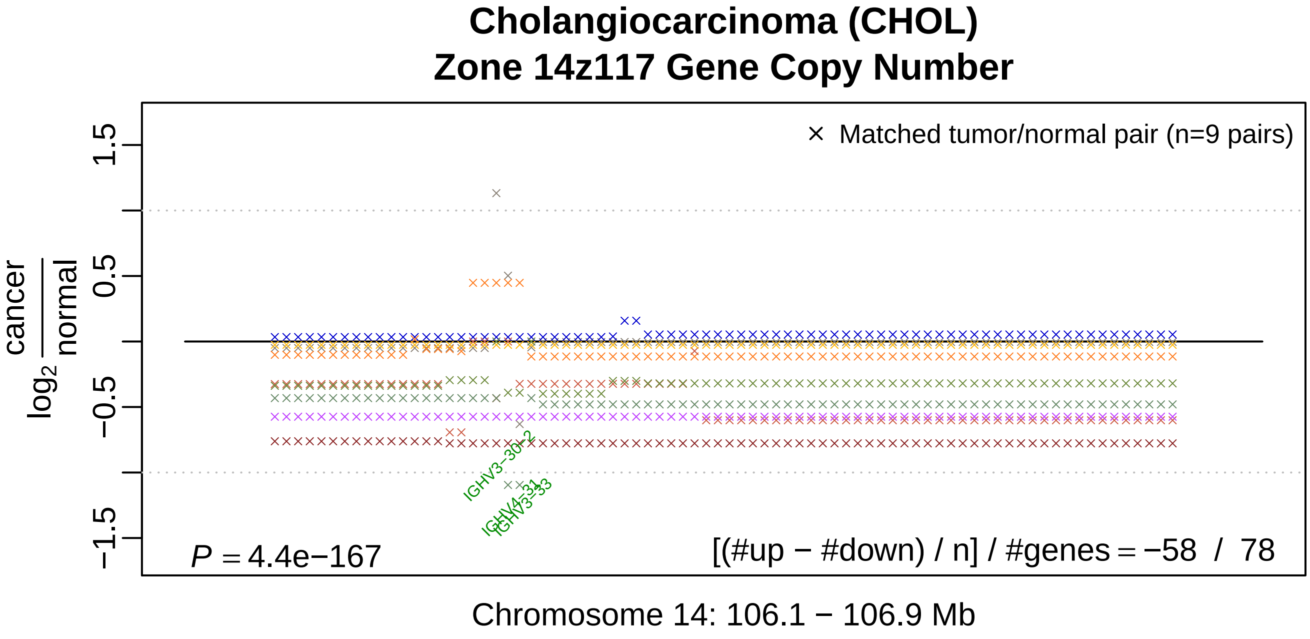

b

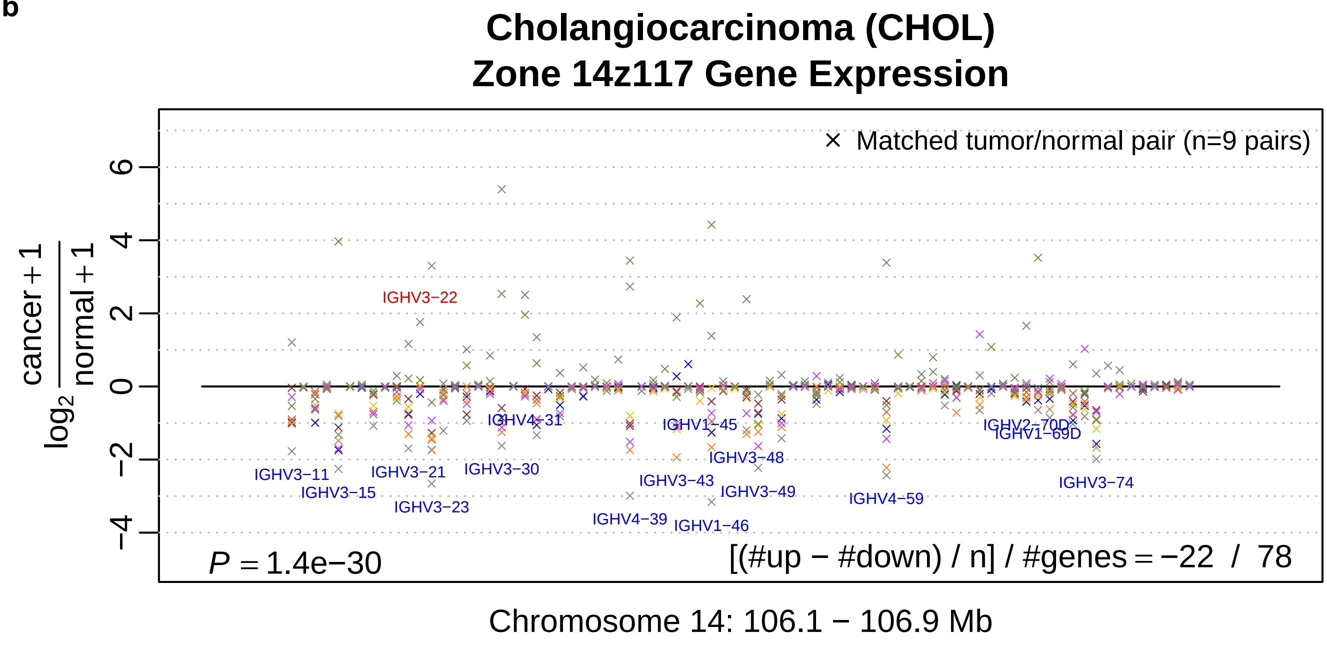

Figure N4.3: The most statistically significant polarized somatic copy number zone in CHOL. a, The somatic copy number log ratio of cancer to normal for each gene within the zone in each patient. **b**, The gene expression log ratio of cancer to normal for each gene within the zone in each patient. See the full legend on page 5.

a

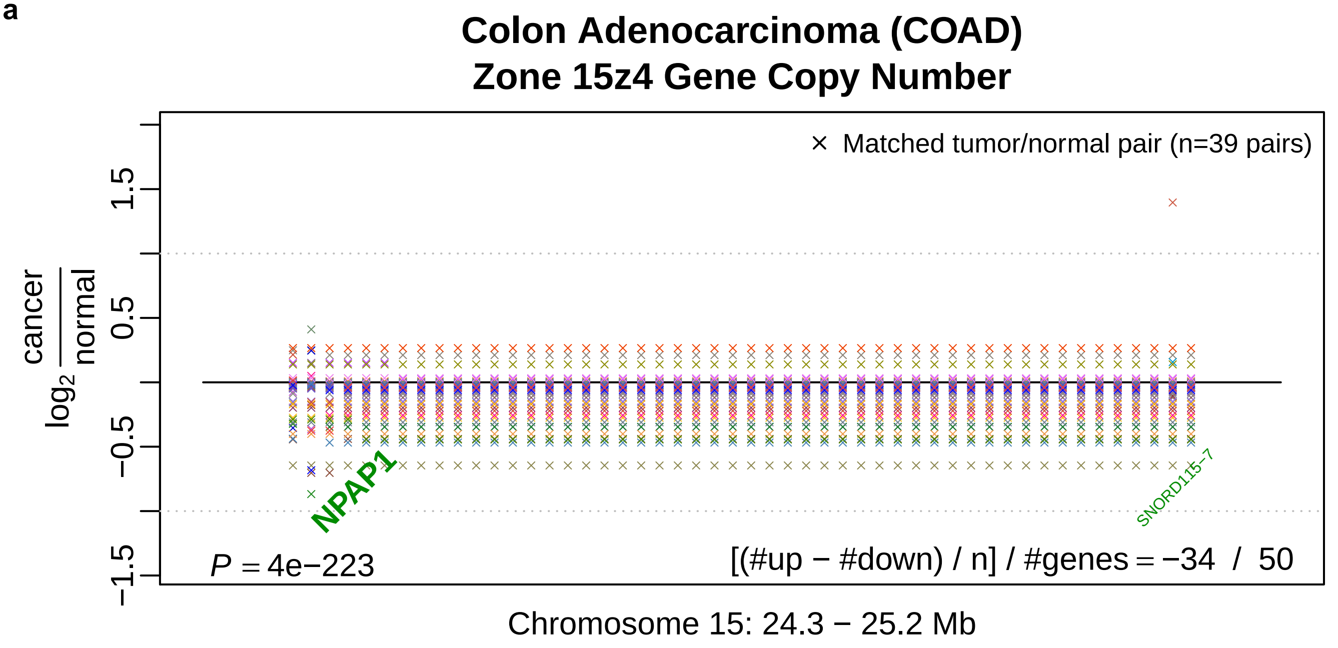

b

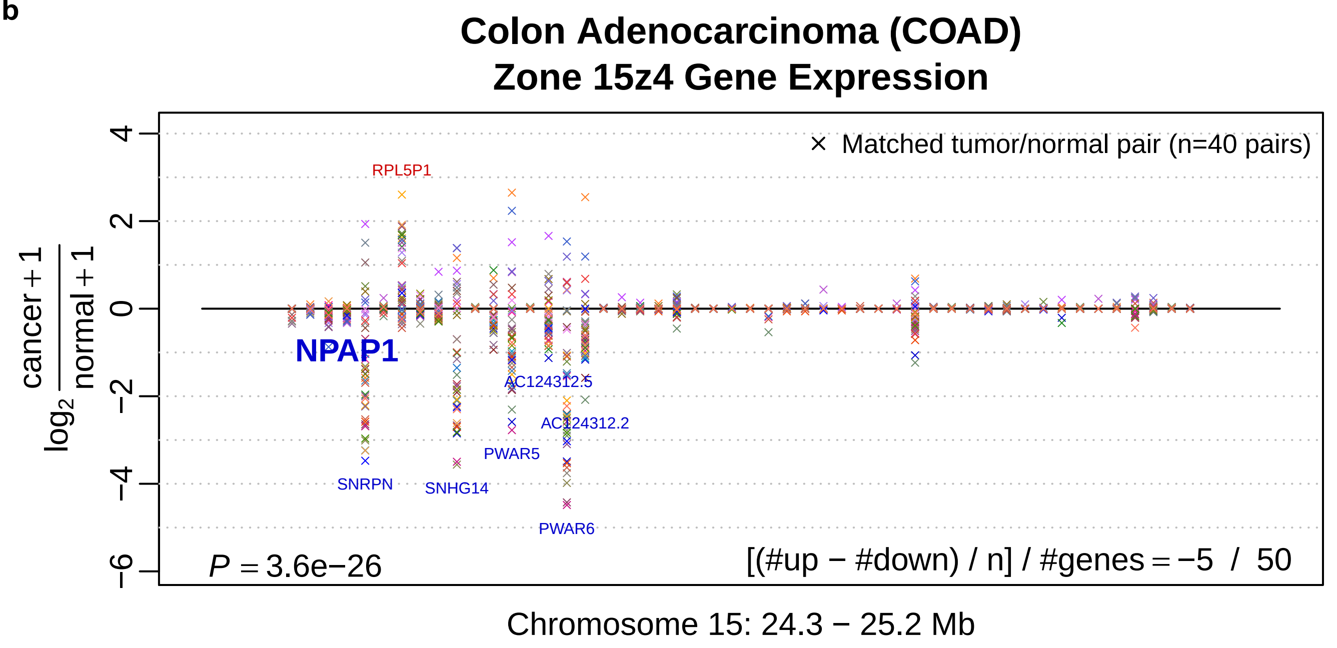

Figure N4.4: The most statistically significant polarized somatic copy number zone in COAD. a, The somatic copy number log ratio of cancer to normal for each gene within the zone in each patient. **b**, The gene expression log ratio of cancer to normal for each gene within the zone in each patient. See the full legend on page 5.

a

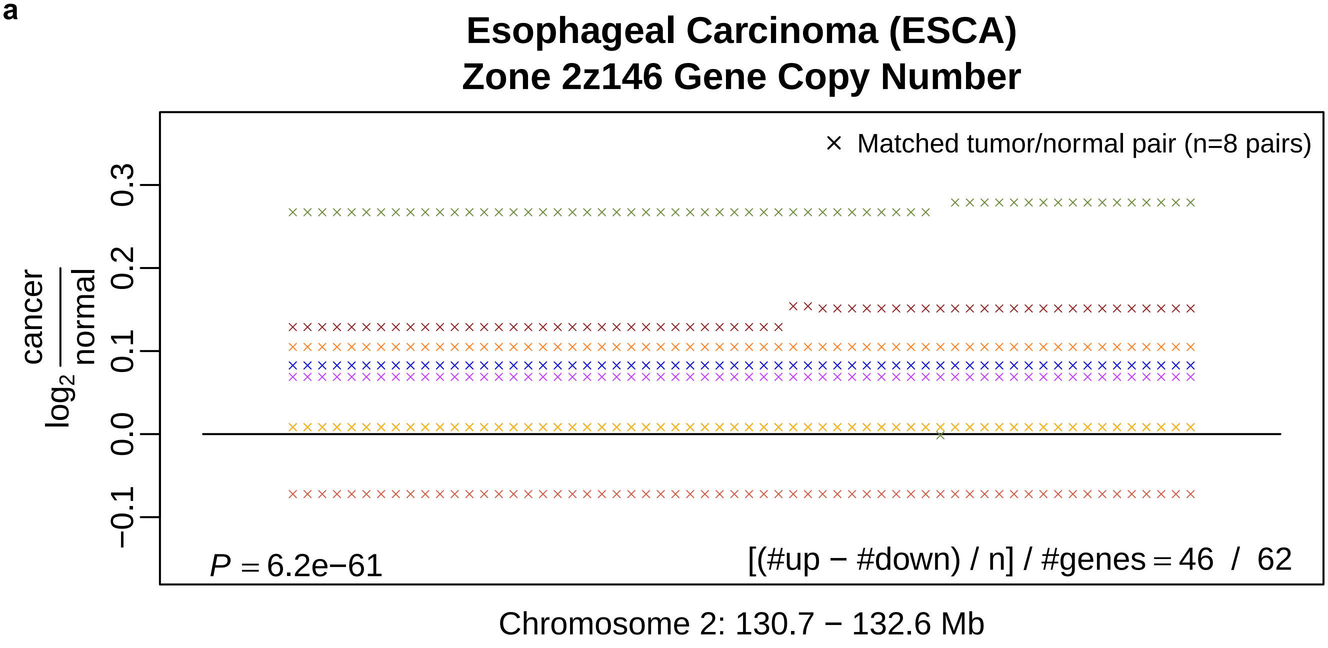

b

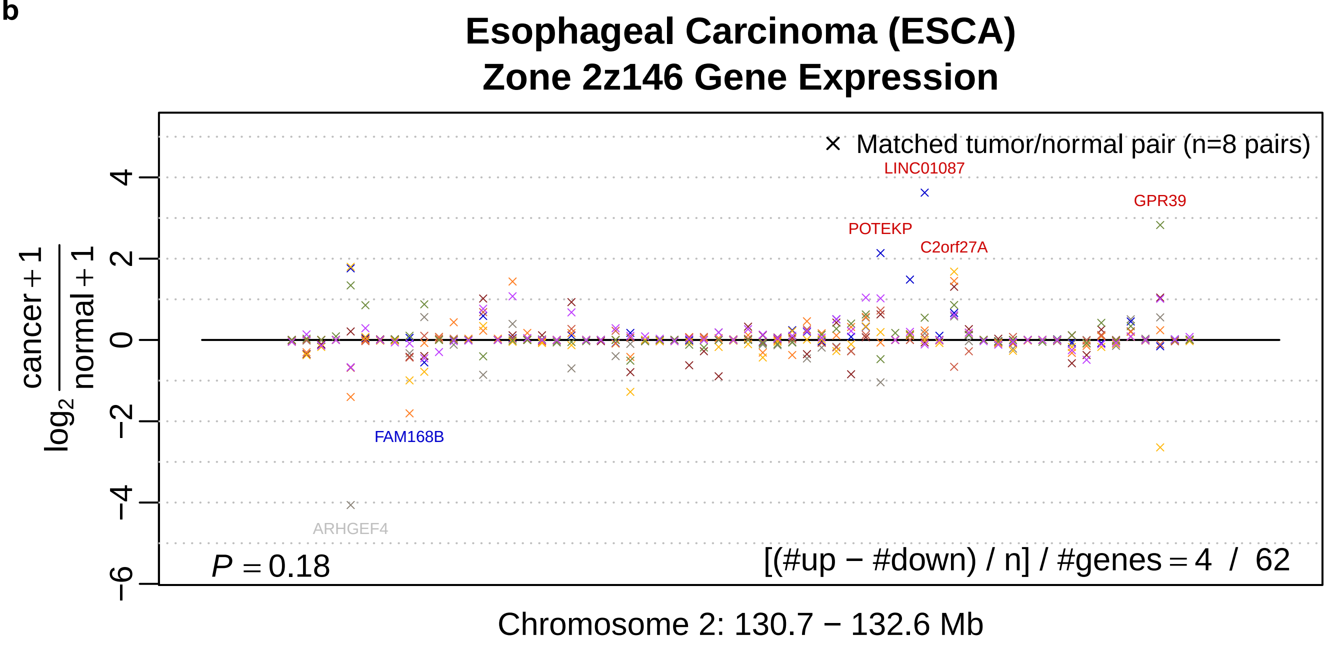

Figure N4.5: The most statistically significant polarized somatic copy number zone in ESCA. a, The somatic copy number log ratio of cancer to normal for each gene within the zone in each patient. **b**, The gene expression log ratio of cancer to normal for each gene within the zone in each patient. See the full legend on page 5.

a

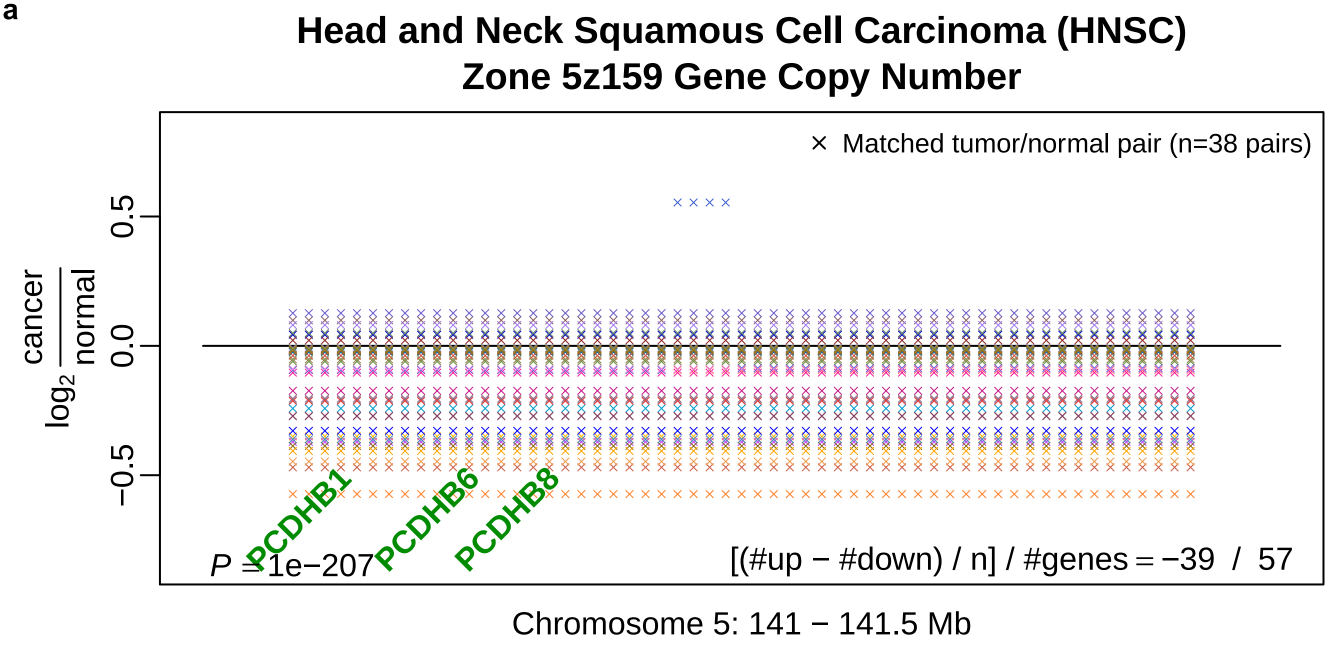

b

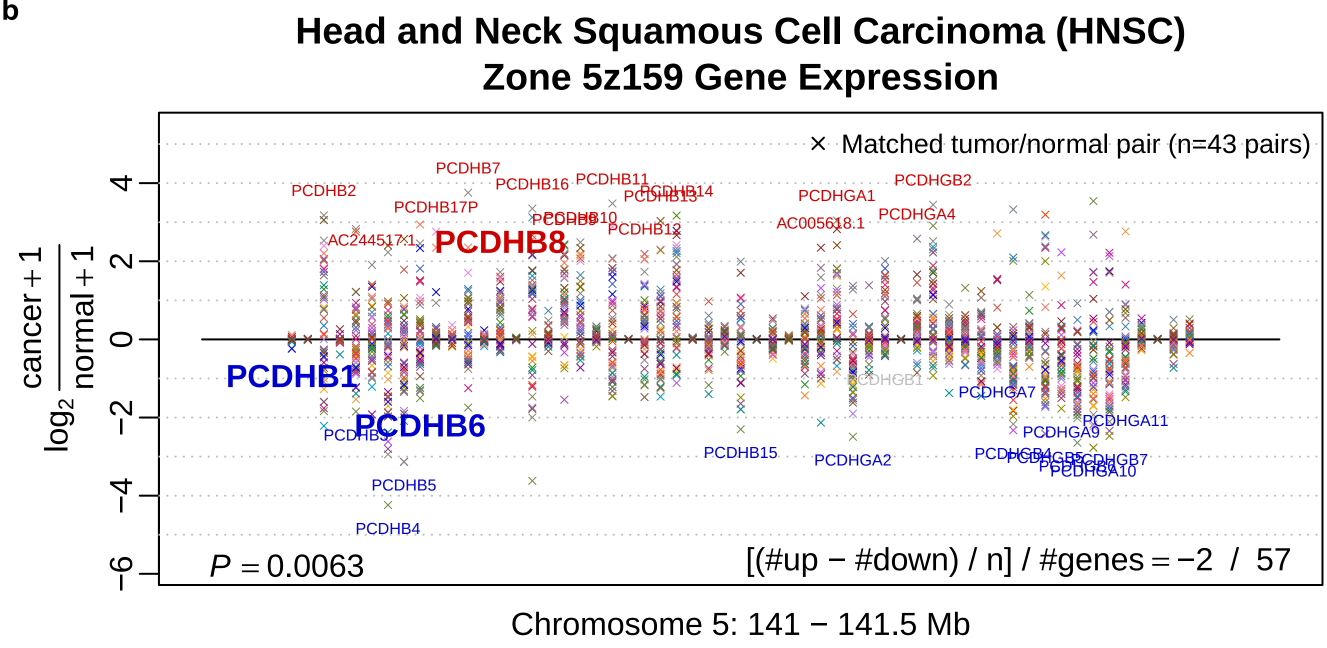

Figure N4.6: The most statistically significant polarized somatic copy number zone in HNSC. a, The somatic copy number log ratio of cancer to normal for each gene within the zone in each patient. **b**, The gene expression log ratio of cancer to normal for each gene within the zone in each patient. See the full legend on page 5.

a

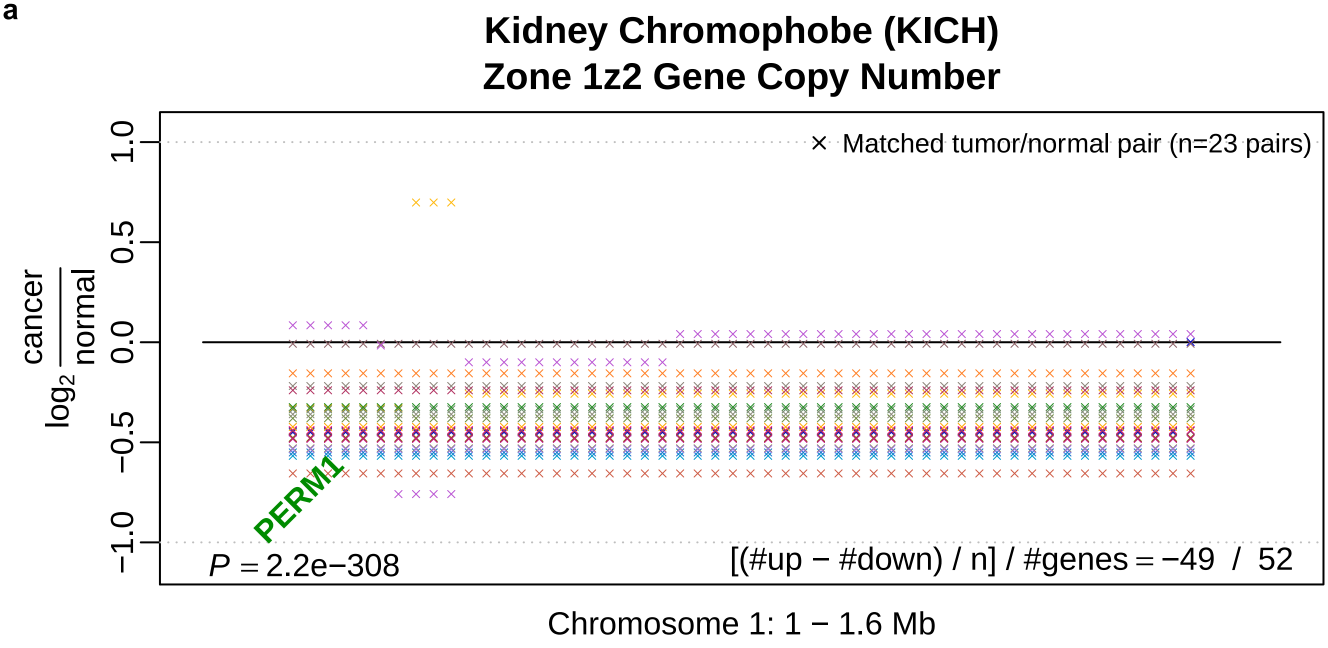

b

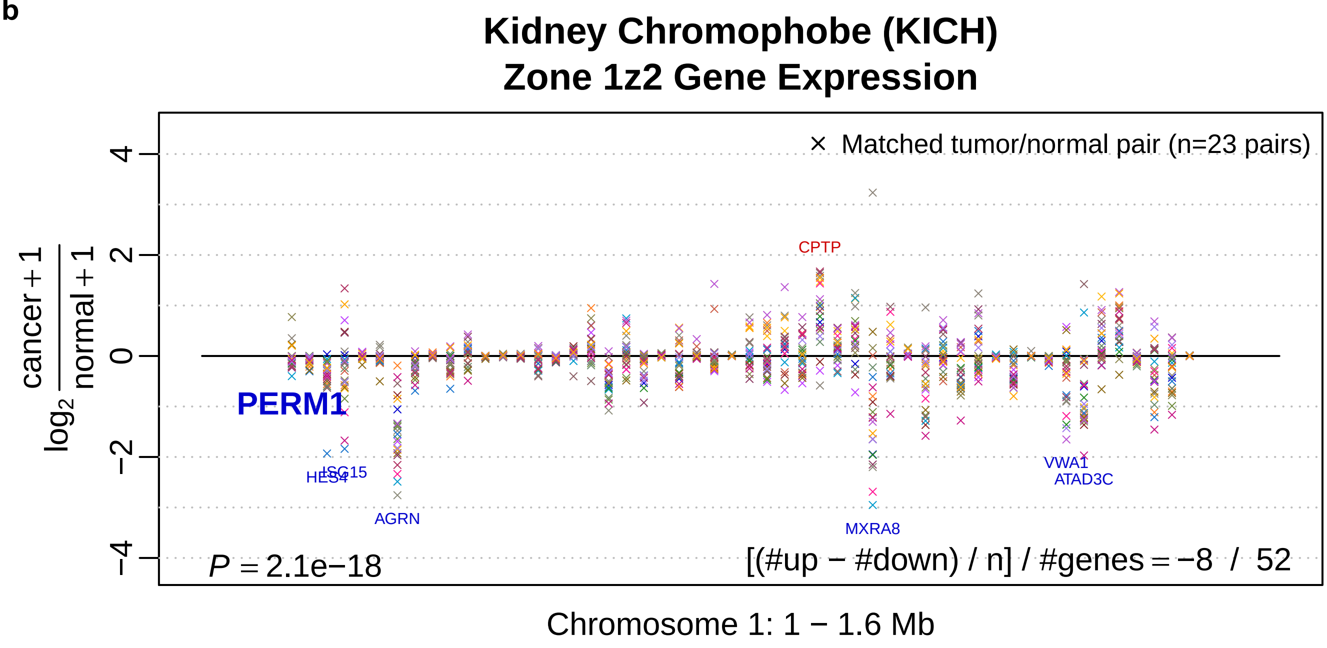

Figure N4.7: The most statistically significant polarized somatic copy number zone in KICH. a, The somatic copy number log ratio of cancer to normal for each gene within the zone in each patient. **b**, The gene expression log ratio of cancer to normal for each gene within the zone in each patient. See the full legend on page 5.

a

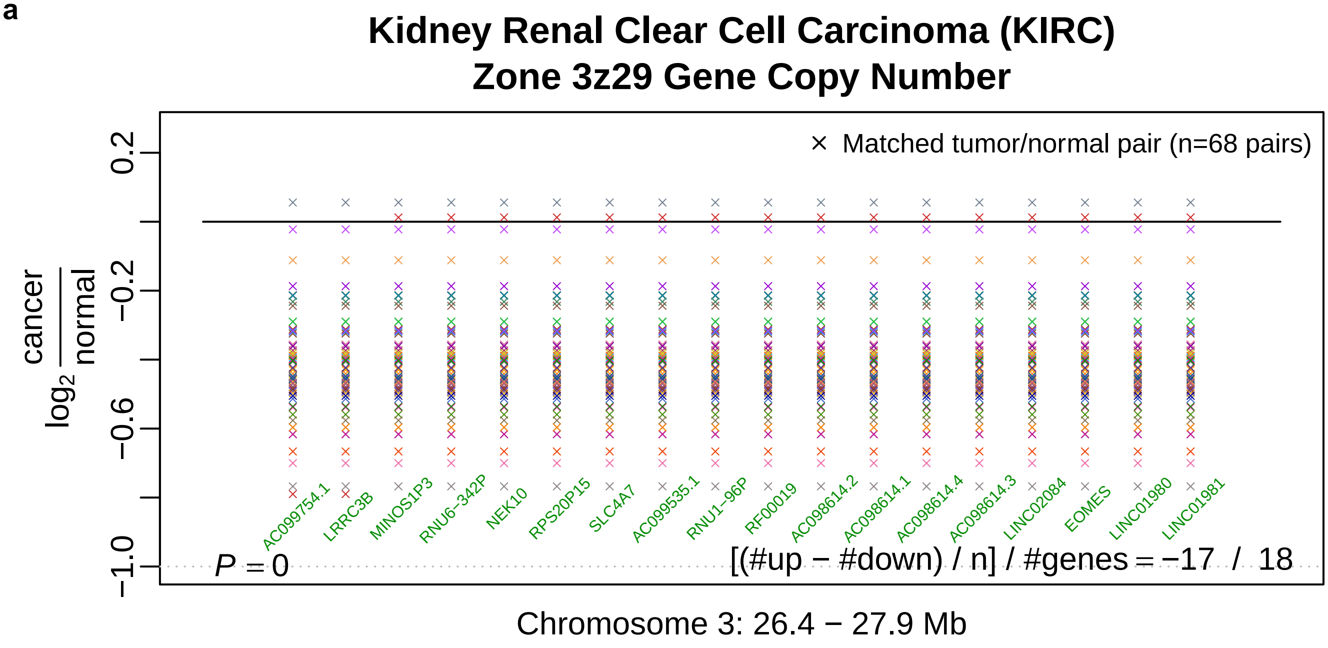

b

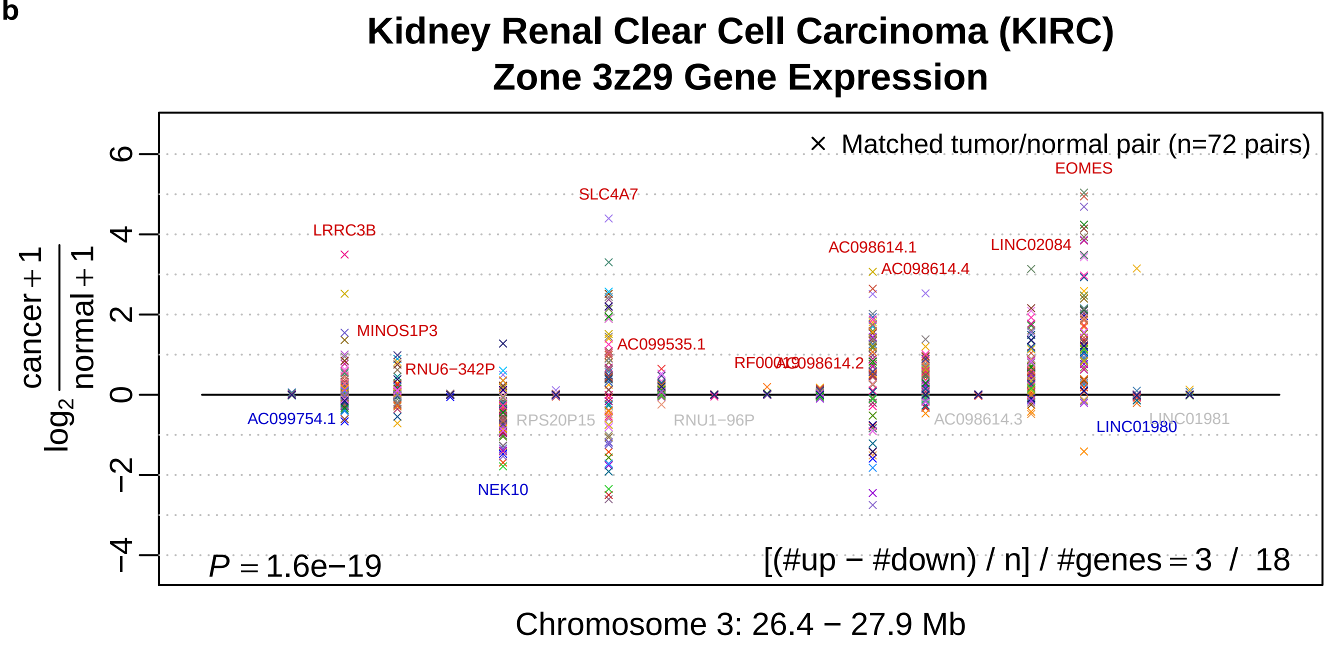

Figure N4.8: The most statistically significant polarized somatic copy number zone in KIRC. a, The somatic copy number log ratio of cancer to normal for each gene within the zone in each patient. **b**, The gene expression log ratio of cancer to normal for each gene within the zone in each patient. See the full legend on page 5.

a

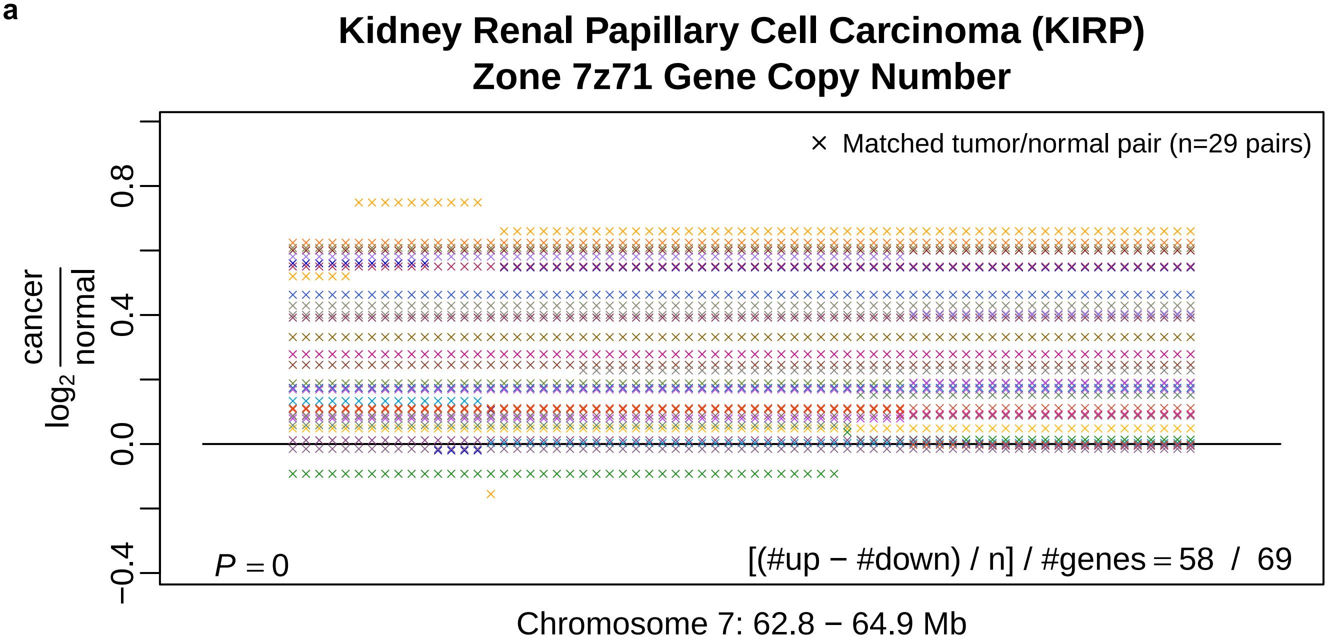

b

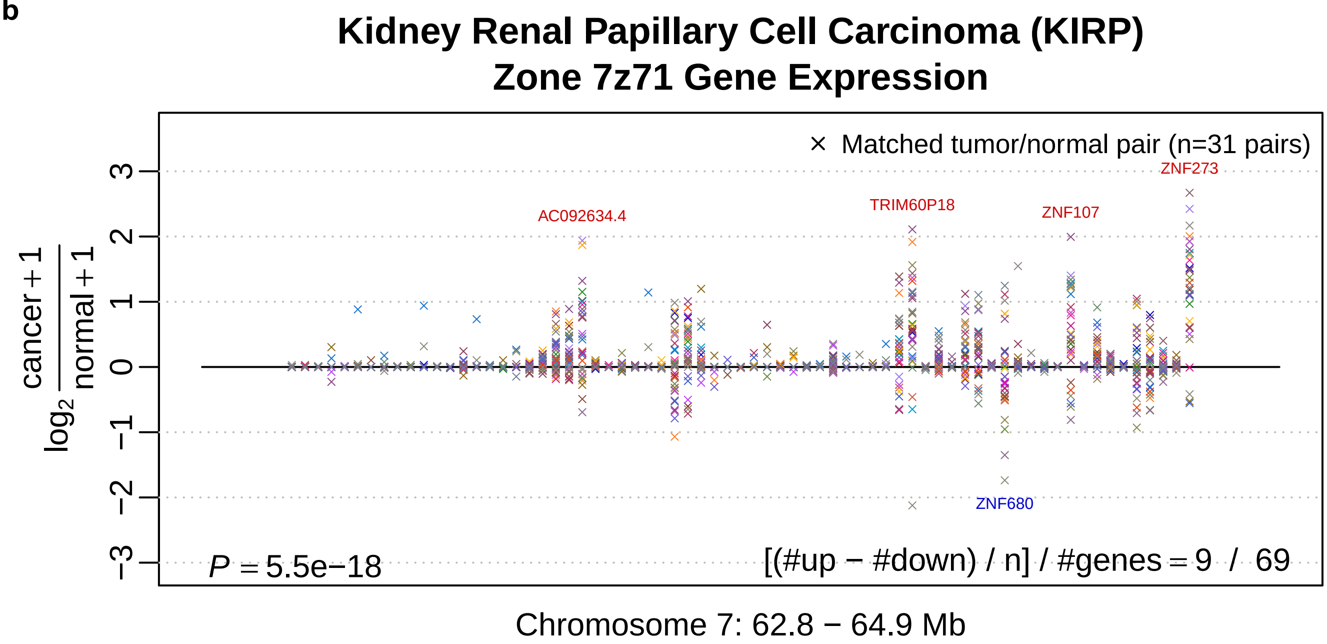

Figure N4.9: The most statistically significant polarized somatic copy number zone in KIRP. a, The somatic copy number log ratio of cancer to normal for each gene within the zone in each patient. **b**, The gene expression log ratio of cancer to normal for each gene within the zone in each patient. See the full legend on page 5.

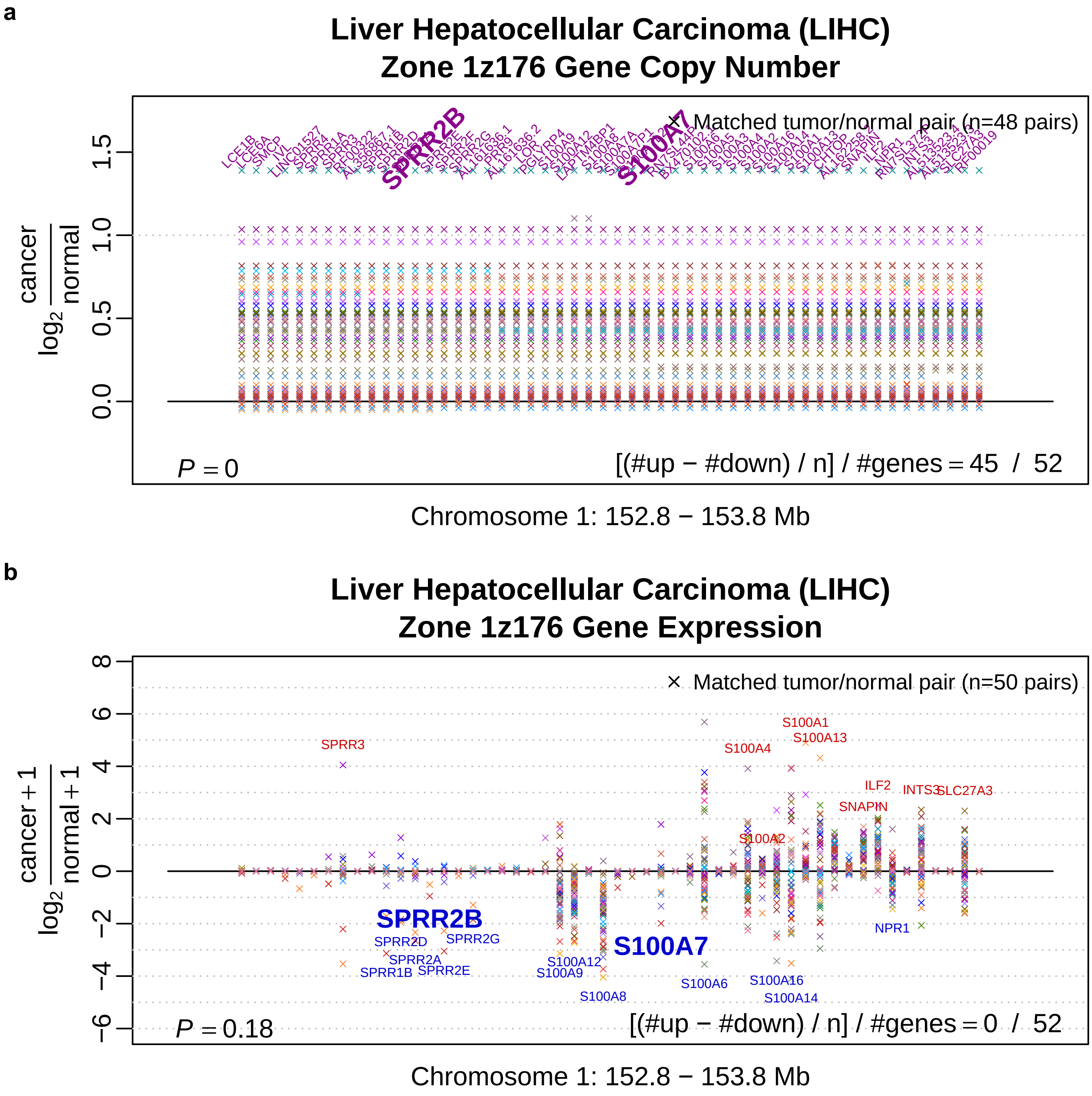

Figure N4.10: The most statistically significant polarized somatic copy number zone in LIHC. a, The somatic copy number log ratio of cancer to normal for each gene within the zone in each patient. **b**, The gene expression log ratio of cancer to normal for each gene within the zone in each patient. See the full legend on page 5.

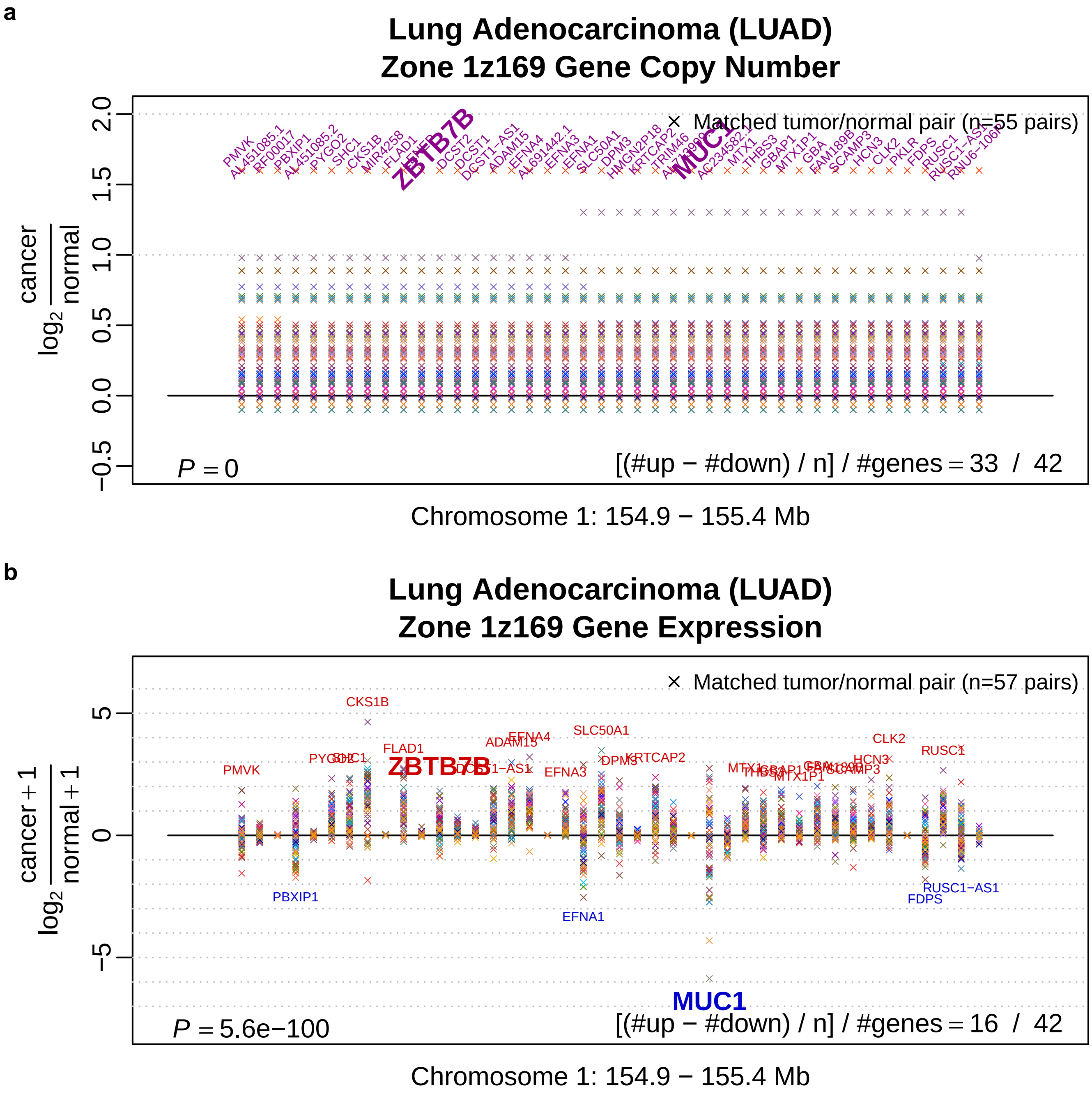

Figure N4.11: The most statistically significant polarized somatic copy number zone in LUAD. a, The somatic copy number log ratio of cancer to normal for each gene within the zone in each patient. **b**, The gene expression log ratio of cancer to normal for each gene within the zone in each patient. See the full legend on page 5.

a

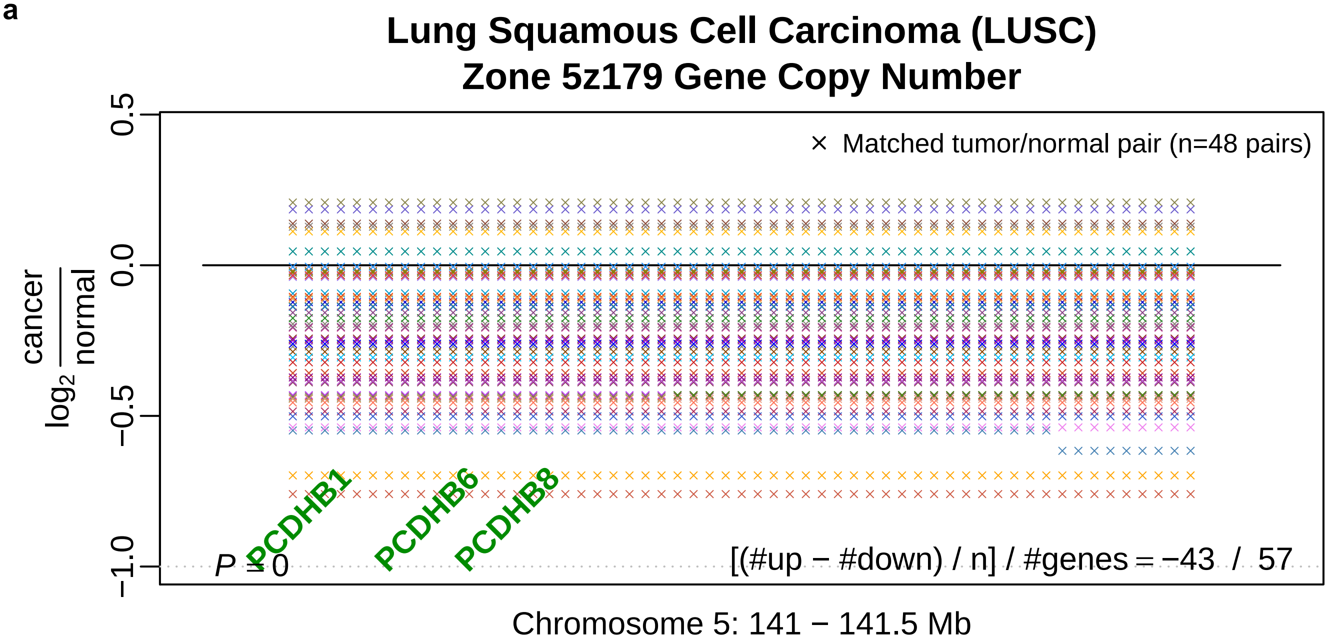

b

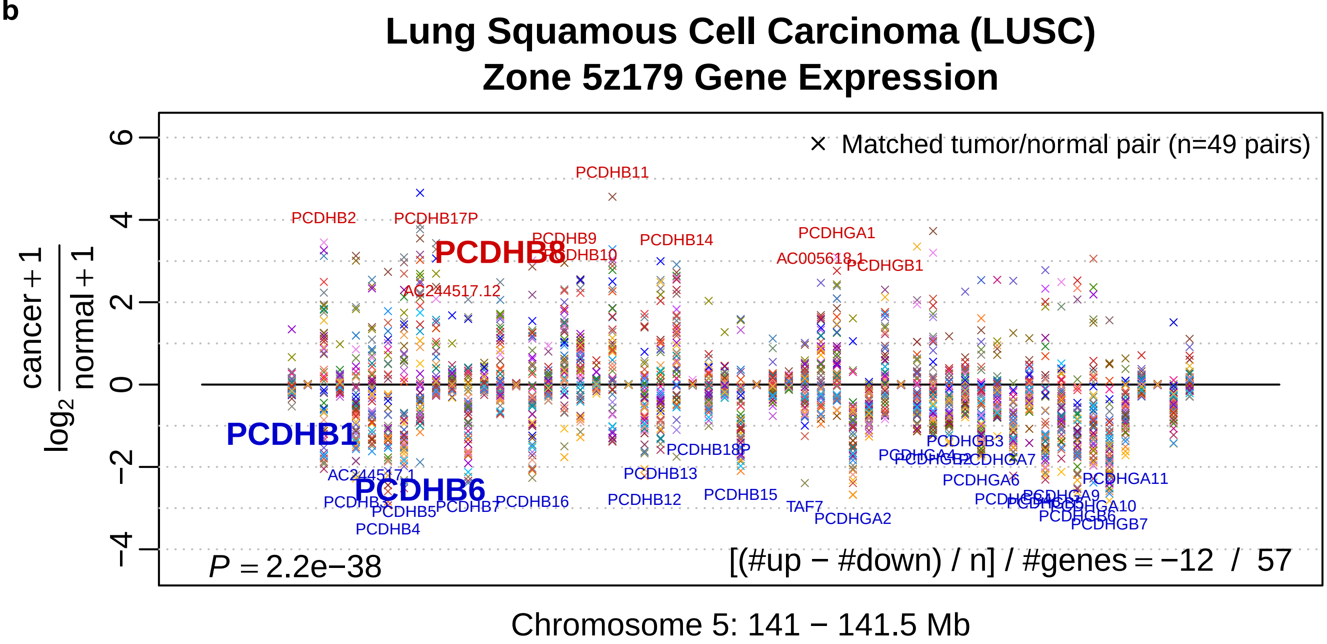

Figure N4.12: The most statistically significant polarized somatic copy number zone in LUSC. a, The somatic copy number log ratio of cancer to normal for each gene within the zone in each patient. **b**, The gene expression log ratio of cancer to normal for each gene within the zone in each patient. See the full legend on page 5.

a

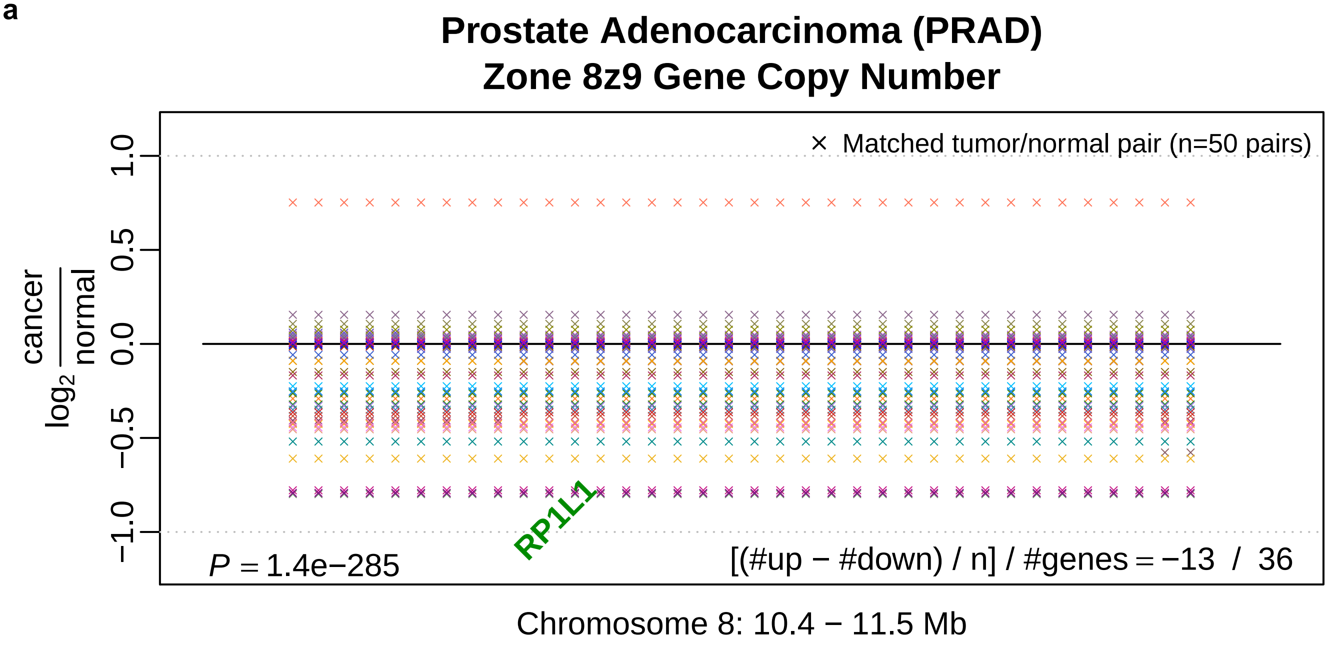

b

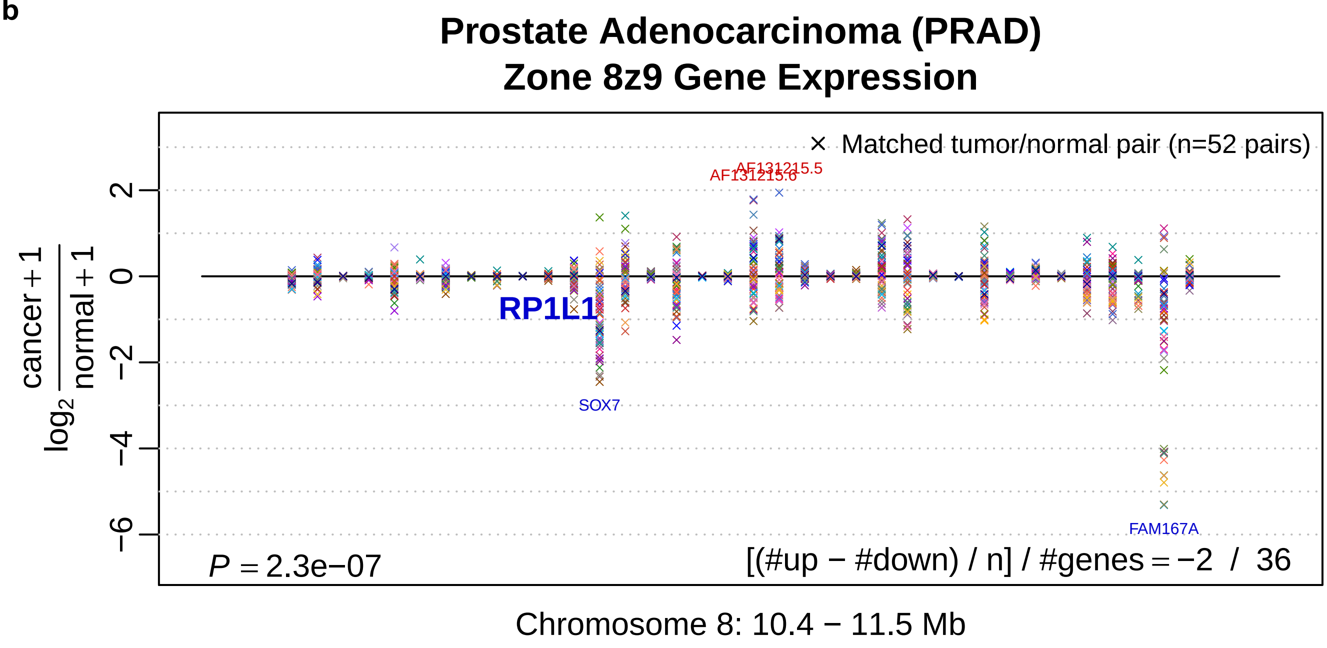

Figure N4.13: The most statistically significant polarized somatic copy number zone in PRAD. a, The somatic copy number log ratio of cancer to normal for each gene within the zone in each patient. **b**, The gene expression log ratio of cancer to normal for each gene within the zone in each patient. See the full legend on page 5.

a

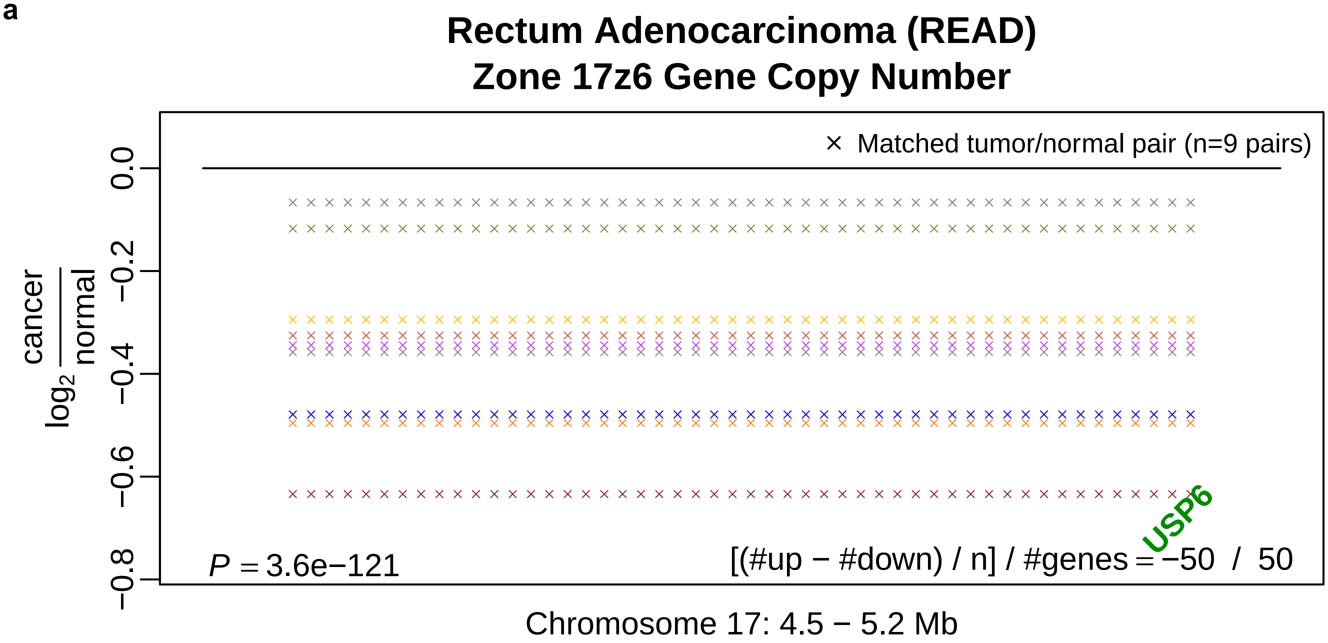

b

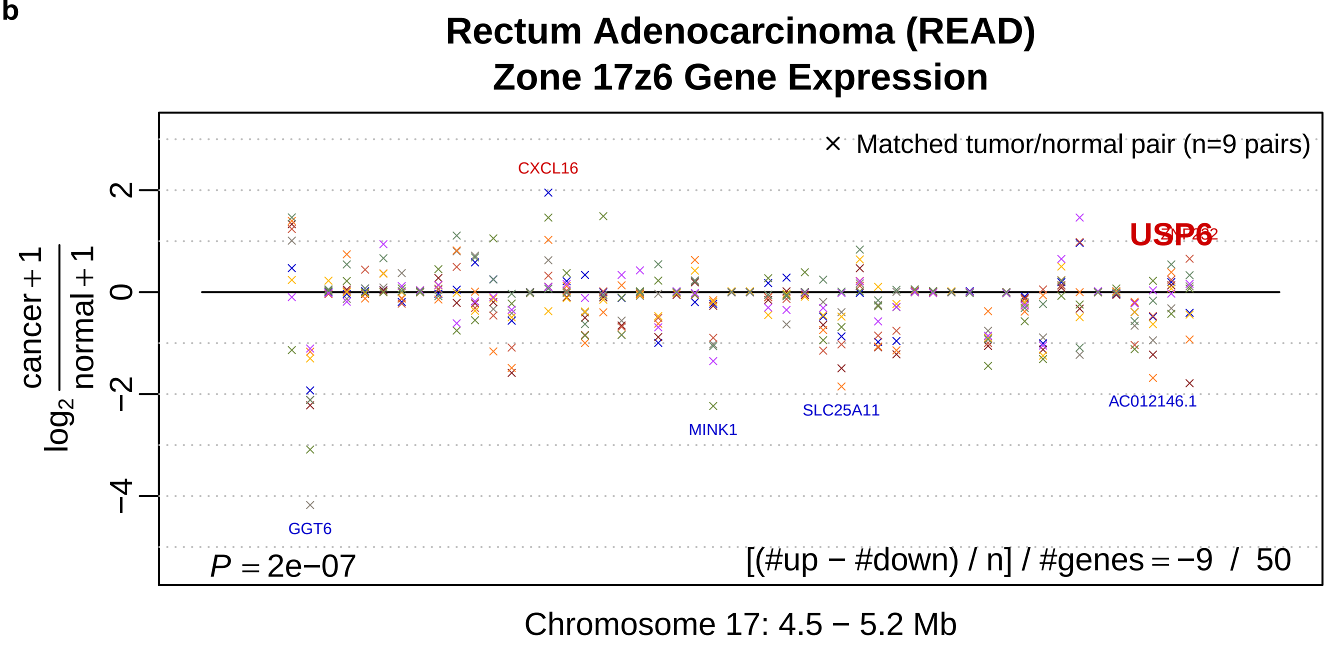

Figure N4.14: The most statistically significant polarized somatic copy number zone in READ. a, The somatic copy number log ratio of cancer to normal for each gene within the zone in each patient. **b**, The gene expression log ratio of cancer to normal for each gene within the zone in each patient. See the full legend on page 5.

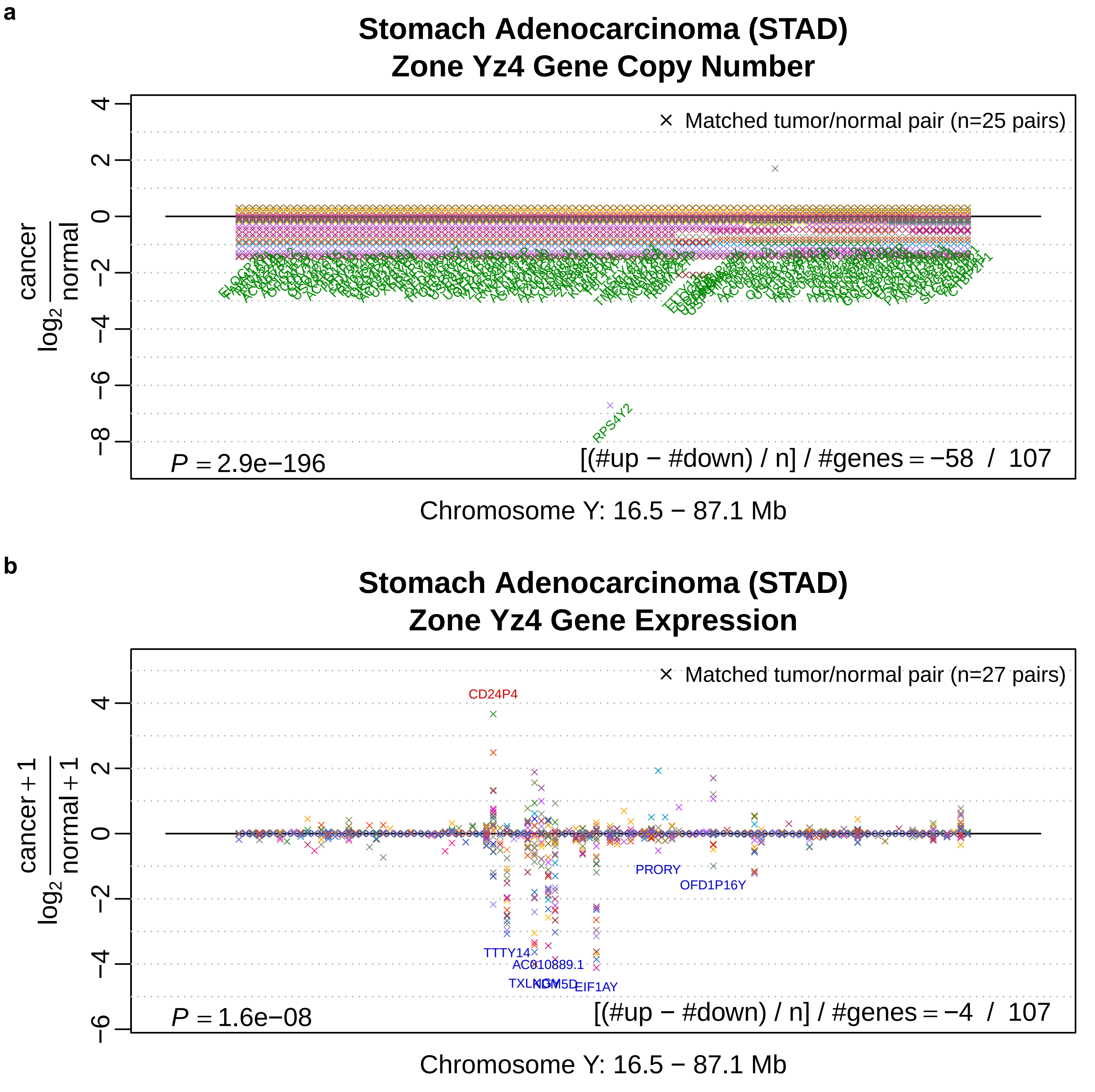

Figure N4.15: The most statistically significant polarized somatic copy number zone in STAD. a, The somatic copy number log ratio of cancer to normal for each gene within the zone in each patient. **b**, The gene expression log ratio of cancer to normal for each gene within the zone in each patient. See the full legend on page 5.

a

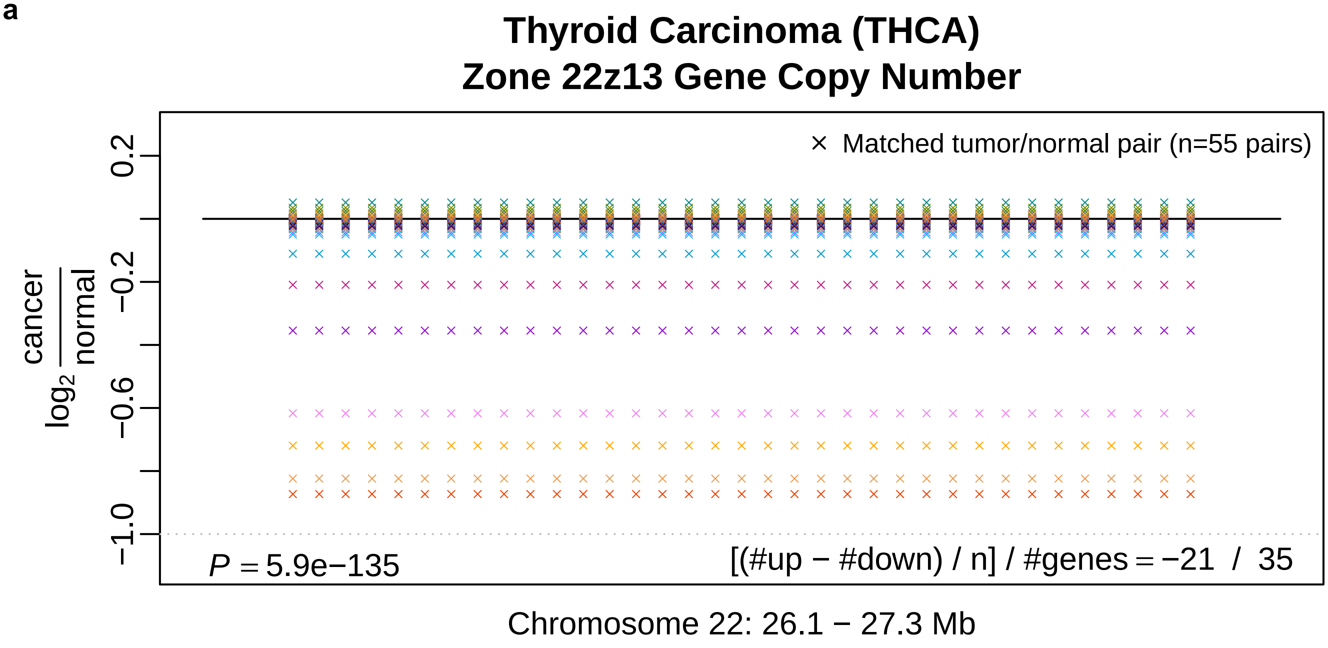

b

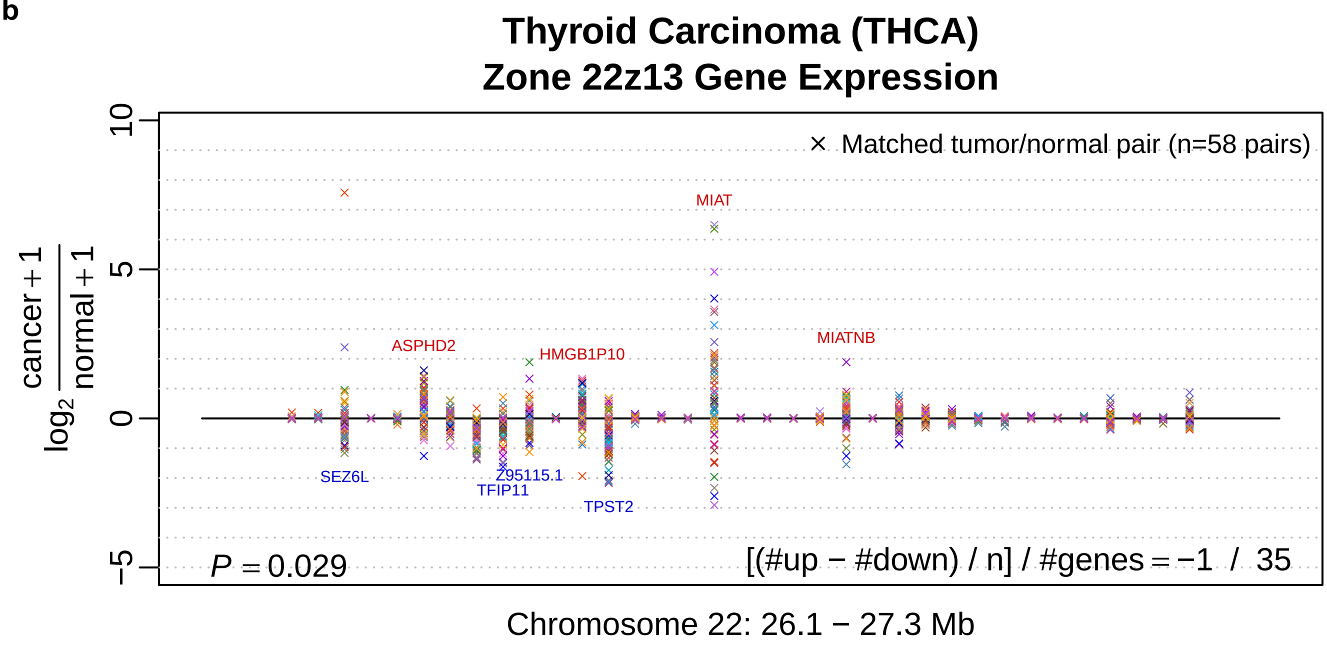

Figure N4.16: The most statistically significant polarized somatic copy number zone in THCA. a, The somatic copy number log ratio of cancer to normal for each gene within the zone in each patient. **b**, The gene expression log ratio of cancer to normal for each gene within the zone in each patient. See the full legend on page 5.

a

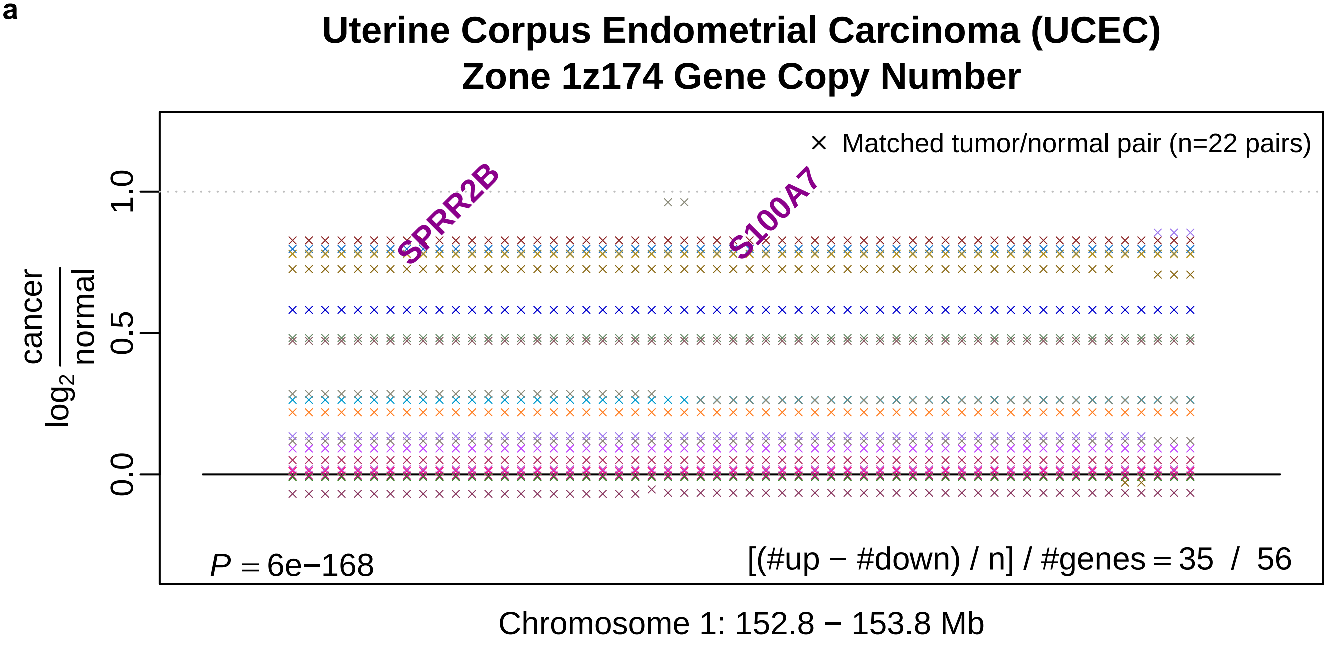

b

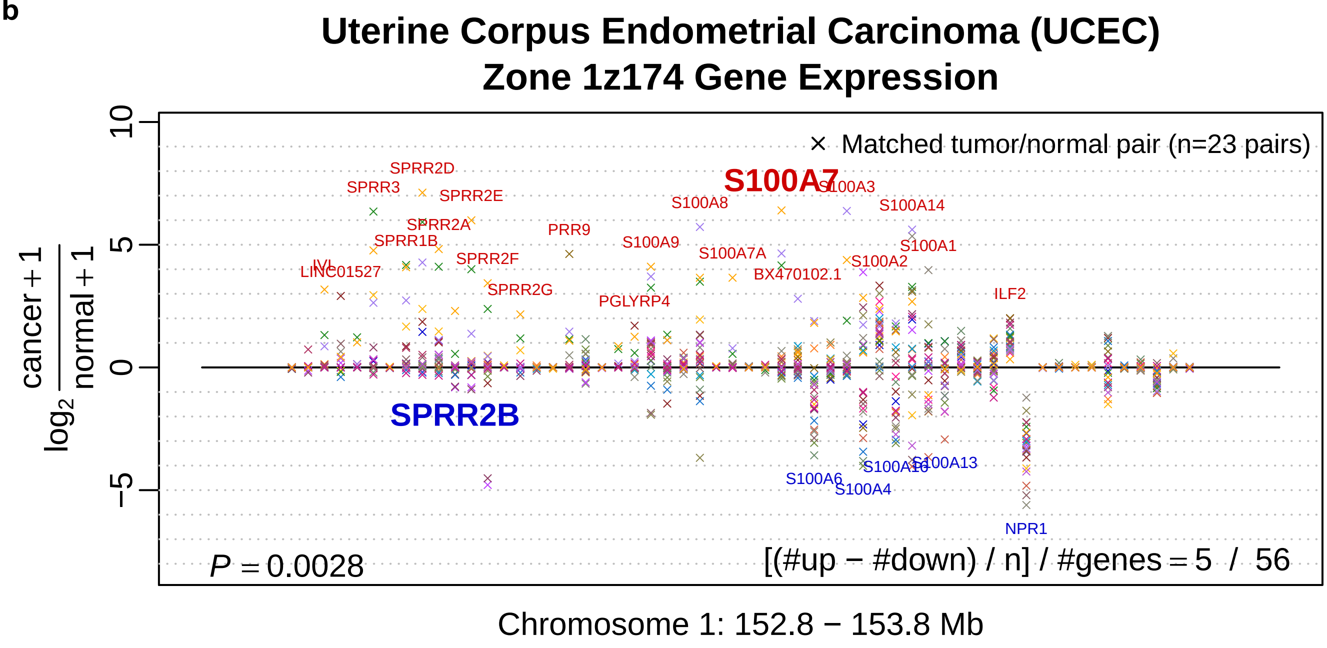

Figure N4.17: The most statistically significant polarized somatic copy number zone in UCEC. a, The somatic copy number log ratio of cancer to normal for each gene within the zone in each patient. **b**, The gene expression log ratio of cancer to normal for each gene within the zone in each patient. See the full legend on page 5.
