# Supplementary material for: Efficient weighted univariate clustering maps outstanding dysregulated genomic zones in human cancers
Source: Bioinformatics. 2020 Jul 3;36(20):5027–36. doi: 10.1093/bioinformatics/btaa613 (PMC7755420; doi:10.1093/bioinformatics/btaa613)
Supplement: btaa613_Supplementary_Data [file btaa613_supplementary_data.zip › SuppNote-N5.pdf]

Supplementary Note N5: Optimal weighted univariate clustering algorithms and proofs

Contents

N5.1 Overview of weighted univariate clustering algorithms **N5-1**

N5.2 A dynamic programming solution to weighted univariate clustering **N5-2**

N5.3 Quadratic-time dynamic programming **N5-3**

N5.4 Speeding up dynamic programming to log-linear time **N5-4**

N5.5 A linear time solution to weighted univariate clustering **N5-9**
N5.5.1 The univariate clustering problem is totally monotonic N5-10
N5.5.2 Linear-time search space reduction N5-11
N5.5.3 Divide-and-conquer N5-14

N5.6 Estimating the optimal number of clusters **N5-15**

This supplementary file overlaps with the Methods section in the main text. Here we provide a self-contained description of methods including all proofs.

N5.1 Overview of weighted univariate clustering algorithms

We define the weighted univariate clustering problem first and give its exact dynamic programming solution based on extension from the solution to the unweighted univariate clustering problem by (Bellman, 1973).

Then we discuss three options to speed up the dynamic programming to attain quadratic, log-linear, and linear runtime, with increasingly heavy use of the mathematical properties of the weighted univariate clustering problem.

For a given k, two slower algorithms in quadratic $O(kn^2)$ and log-linear time $O(kn \log n)$, respectively, are first presented to setup the background for the linear time algorithm. The quadratic time algorithm is derived from the unweighted univariate clustering algorithm introduced by Wang and Song (2011). The log-linear time algorithm is based on a general solution to the monotonic matrix search problem (Aggarwal et al., 1987). The linear-time $O(kn)$ algorithm takes advantage of a *total monotone* property of the weighted univariate clustering problem. Stronger than the monotone property, the total monotone property states that even if we constrain cluster boundaries to fall on a subset of data points, the constrained optimal boundaries will not decrease if additional points greater than the current points in the subproblem are inserted. With this property, the divide-and-conquer strategy to fill a row of the dynamic programming matrices reduces the problem into one subproblem of half the size—instead of two in the log-linear time solution—removing the $\lg n$-factor from the log-linear time solution. Aggarwal et al. (1987) designed the SMAWK algorithm to search row maxima in totally monotonic matrices. After establishing a concave quadrangle inequality for the sum of squared distance function, we prove that subproblems of weighted univariate clustering can be formulated as problems to search row minima of totally monotonic matrices. The core of the linear-time algorithm is a novel algorithm to perform matrix column reduction in-place to minimize runtime overhead. We prove the algorithm is correct and derive its asymptotic runtime.

When the number of clusters is given by a range $[k_{\text{min}}, k_{\text{max}}]$, the runtime of each algorithm replaces k by k_{max}, as solving for k_{max} would automatically generate results for smaller k values. To choose an optimal

number of clusters from the range for k, we use the Bayesian information criterion that promotes likelihood based on a Gaussian mixture model and penalizes the number of components in the model.

N5.2 A dynamic programming solution to weighted univariate clustering

Given an array of n sorted numbers $x_0 \le x_1 \le \cdots \le x_{n-1}$ with non-negative weights $y_0, y_1, \cdots, y_{n-1}$, we define a k-clustering $C(k, n)$ as k disjoint intervals covering all numbers in $0, \ldots, n-1$ using the following notation:

$$C(k,n) = \left\{ \underbrace{[j_0+1,\, j_1]}_{\text{cluster } 0}, \underbrace{[j_1+1, j_2]}_{\text{cluster } 1}, \cdots, \underbrace{[j_{k-2}+1, j_{k-1}]}_{\text{cluster } k-2}, \underbrace{[j_{k-1}+1, j_k]}_{\text{cluster } k-1} \right\}$$

where $j_0 = -1 < j_1 < \cdots < j_k = n-1$. We call $j_{k-1}+1$ the lower decision boundary of cluster $k-1$ and j_k the upper decision boundary of cluster $k-1$.

Now we define the distance measure used in univariate clustering. Let $\mu(j,i)$ be the weighted mean function defined on x_j to x_i $(j \le i)$, a subarray of x, as follows:

$$\mu(j,i) = \frac{1}{Y_i - Y_{j-1}} \sum_{l=j}^{i} x_l y_l \tag{N5.1}$$

where

$$Y_i = \begin{cases} \sum_{l=0}^{i} y_l & i \ge 0 \\ 0 & i = -1 \end{cases} \tag{N5.2}$$

We define the sum of squared distances function $s(j,i)$ from each point in x_j to x_i to the mean $\mu(j,i)$ as

$$s(j,i) = \begin{cases} \sum\limits_{l=j}^{i} y_l \left[x_l - \mu(j,i)\right]^2 & j \le i \\ \infty & j > i \end{cases} \tag{N5.3}$$

For a clustering $C(k,n)$, the total sum of squared distances is

$$SSQ(C(k,n)) = \sum_{q=0}^{k-1} s(j_q+1, j_{q+1}) \tag{N5.4}$$

mathematically equivalent to

$$SSQ(C(k,n)) = \sum_{i=0}^{n-1} y_i x_i^2 - \sum_{q=0}^{k-1} (Y_{j_{q+1}} - Y_{j_q}) \mu^2(j_q+1, j_{q+1}) \tag{N5.5}$$

A clustering $C^*(k,n)$ is optimal if and only if it minimizes $SSQ(C(k,n))$.

Bellman (1973) first described a dynamic programming solution to the unweighted univariate clustering problem based on additive distance measures. Let S be a $k \times n$ matrix defined by

$$S[q,i] = \text{min}\{SSQ(C(q+1, i+1))\} \quad q = 0, \ldots, k-1;\ i = 0, \ldots, n-1 \tag{N5.6}$$

which is the minimum SSQ value when x_0 to x_i are grouped into $q+1$ clusters in an optimal clustering $C^*(q+1, i+1)$. Let $J[q,i]$ in another $k \times n$ matrix J be the smallest index to the points in cluster q of

$C^*(q+1, i+1)$. The recurrence equations for dynamic programming are given as (Bellman, 1973)

$$S[q, i] = \min_{q \le j \le i} S[q-1, j-1] + s(j, i) \quad 0 < q \le i \tag{N5.7}$$

$$J[q, i] = \max \left\{ j \mid \operatorname*{argmin}_{q \le j \le i} S[q-1, j-1] + s(j, i) \right\} \quad 0 < q \le i \tag{N5.8}$$

If there are multiple optimal clustering solutions, we assign the maximum of all optimal indices to $J[q][i]$. Matrices S and J are initialized by

$$S[0, i] = s(0, i),\; J[0, i] = 0 \quad 0 \le i \le n-1 \tag{N5.9}$$

To ensure each cluster contains at least one point, we set $S[q, i] = \infty$ and $J[q, i]$ undefined when $i < q$.

Definition 1. *The optimal index function $j_q(i)$ maps i in given cluster q to the largest index that achieves the minimum distance measure $S[q, i]$ by*

$$j_q(i) = J[q, i] \tag{N5.10}$$

This definition will be used in deriving the log-linear and linear time dynamic programming algorithms.

To retrieve an optimal solution, the Backtrack algorithm returns the upper boundaries of each cluster in linear time.

```
Backtrack(J, n, k)
b = an empty array to hold k upper boundaries of each cluster
j = n − 1
q = k − 1
b[q] = j
while q > 0
      j = J[q,j] − 1
      q = q − 1
      b[q] = j
return b
```

Solving the recurrence equations in Eqs (N5.7, N5.8) must search for an optimal j among $O(n)$ candidates and evaluate each candidate using $O(n)$ time to compute $s(j, i)$, for each of the $(k-2) \times n$ entries in S and J. For the last row in S and J ($q = k-1$), it is only necessary to compute the last element $S[q, n-1]$ and $J[q, n-1]$ in time $O(n^2)$. It takes an additional time of $O(n \lg n)$ when x is unsorted. The backtrack algorithm takes $O(k)$ time. Therefore, the total runtime to solve the recurrence equation is $O(n \lg n + (k-2)n^3 + n^2)$. The space complexity is $O(kn)$ when an optimal clustering must be returned by backtracking. Unfortunately, the cubic runtime makes this original dynamic programming solution unrealistic for large data sets despite the guaranteed clustering optimality.

N5.3 Quadratic-time dynamic programming

The quadratic speedup uses the same recurrence equations as defined in Eqs (N5.7) and (N5.8) but computes $s(j, i)$ in constant time. In the early versions (Wang and Song, 2011) of R package `Ckmeans.1d.dp`, $s(j, i)$ was computed backward from $s(j+1, i)$ in constant time.

In the current quadratic implementation, $s(j, i)$ is indirectly computed in constant runtime from stored running sums of elements in x and their squares. Specifically, we precompute running sums of x and their

squares

$$Z[i] = \sum_{l=0}^{i} x_l y_l, \quad Y[i] = \sum_{l=0}^{i} y_l, \quad \text{and} \quad Q[i] = \sum_{l=0}^{i} y_l x_l^2 \quad i = 0, \dots, n-1$$

and store them in two arrays before filling the dynamic programming matrices. Using the precomputed running sums is also applicable to the fast log-linear-time and linear-time solutions to be described in the following subsections.

To curb numerical round-off errors in computing sums $Z[i]$ and sums of squares $Q[i]$, which can be much larger than x_l, we shift the original values in x by subtracting its median. A shift of x will not change the optimal solution to the univariate clustering problem. This measure broadens the range of input arrays the program can handle without numerical instability.

During dynamic programming for each entry in the matrices, instead of using Eq (N5.1) which takes linear time, we equivalently compute in constant time the mean of x_j to x_i by

$$\mu(j,i) = \begin{cases} \frac{1}{Y[i]} \sum_{l=0}^{i} x_l y_l = \frac{Z[i]}{Y[i]} & j = 0,\, i \ge 0 \\ \frac{1}{Y[i]-Y[j-1]} \left(\sum_{l=0}^{i} x_l y_l - \sum_{l=0}^{j-1} x_l y_l \right) = \frac{Z[i]-Z[j-1]}{Y[i]-Y[j-1]} & 0 < j \le i \end{cases} \tag{N5.11}$$

Instead of using Eq (N5.3) in linear time, we calculate $s(j,i)$ in constant time using the running sums by

$$s(j,i) = \begin{cases} \sum_{l=0}^{i} y_l x_l^2 - \frac{1}{Y[i]} \left(\sum_{l=0}^{i} x_l y_l \right)^2 & j = 0,\, i \ge 0 \\ \sum_{l=0}^{i} y_l x_l^2 - \sum_{l=0}^{j-1} y_l x_l^2 - (Y[i] - Y[j-1])\mu^2(j,i) & 0 < j \le i \end{cases} \tag{N5.12}$$

or equivalently

$$s(j,i) = \begin{cases} Q[i] - \frac{1}{Y[i]} Z^2[i] & j = 0,\, i \ge 0 \\ Q[i] - Q[j-1] - (Y[i] - Y[j-1])\mu^2(j,i) & 0 < j \le i \end{cases} \tag{N5.13}$$

With a constant time $O(1)$ needed for computing $s(j,i)$ down from $O(n)$, the total runtime of clustering n points becomes $O(n \lg n + (k-2)n^2 + n)$ when x is unsorted. Although storing the running sums requires additional $O(n)$ space, the total space complexity remains $O(kn)$ when backtrack must be conducted.

N5.4 Speeding up dynamic programming to log-linear time

The subproblems in dynamic programming for the weighted univariate clustering problem satisfy a monotone property that $j_q(i-1) \le j_q(i)$ $(0 < i < n, 0 \le q < k)$. The interpretation is that the lower decision boundary of cluster q in an optimal $(q+1)$-clustering of the first $i-1$ points of x is no greater than that for the first i points. We prove this monotone property, which leads to a log-linear time algorithm. The log-linear solution is not new and a C++ implementation is first available on the Internet (Hilferink, 2015), before being aware of which, we had independently developed the log-linear time implementation in `Ckmeans.1d.dp` version 3.4.6. Although the two implementations are both based on this monotone property, the mathematical derivation and coding differ greatly. Some of the mathematical preliminaries presented hereafter will also be used in arriving at the linear-time dynamic programming solution in the next subsection.

Lemma N5.4.1. *Let μ_n be the weighted mean of a sequence of n real numbers from x_1 to x_n with non-negative weights y_1 to y_n, respectively. After a new real number x with weight $y \ge 0$ is introduced to the*

sequence, the weighted mean of the $n+1$ numbers, μ_{n+1}, satisfies

$$(Y_n + y)\mu_{n+1}^2 - Y_n\mu_n^2 = yx^2 - \frac{Y_n y(\mu_n - x)^2}{Y_n + y} \tag{N5.14}$$

where $Y_n = \sum_{i=1}^n y_i$.

Proof. By algebraic manipulation, we have

$$\begin{aligned}
(Y_n + y)\mu_{n+1}^2 - Y_n\mu_n^2 &= (Y_n + y)\left(\frac{Y_n\mu_n + xy}{Y_n + y}\right)^2 - Y_n\mu_n^2 && \text{(N5.15)}\\
&= \frac{Y_n^2\mu_n^2 + x^2y^2 + 2Y_n yx\mu_n}{Y_n + y} - \frac{Y_n^2\mu_n^2 + Y_n y\mu_n^2}{Y_n + y} && \text{(N5.16)}\\
&= \frac{x^2y^2 + 2Y_n xy\mu_n - Y_n y\mu_n^2}{Y_n + y} && \text{(N5.17)}\\
&= \frac{x^2y^2 + Y_n x^2 y - Y_n x^2 y + 2Y_n xy\mu_n - Y_n y\mu_n^2}{Y_n + y} && \text{(N5.18)}\\
&= x^2 y - \frac{Y_n y(\mu_n - x)^2}{Y_n + y} && \text{(N5.19)}
\end{aligned}$$

which proves the lemma. □

Lemma N5.4.2. *Let $x_1 \leq \ldots \leq x_n$ represent a sequence of n real numbers sorted in non-decreasing order with nonnegative weights y_1 to y_n, respectively. Let μ_n be the weighted mean of the sequence. Let μ_m be the weighted mean of x_1 to x_m. If we introduce $x_0 \leq x_1$ with weight $y_0 \geq 0$, the weighted mean μ_{n+1} of x_0 to x_n and the weighted mean μ_{m+1} of x_0 to x_m must satisfy*

$$Y_m\mu_m^2 - (Y_m + y_0)\mu_{m+1}^2 \leq Y_n\mu_n^2 - (Y_n + y_0)\mu_{n+1}^2 \tag{N5.20}$$

where $Y_m = \sum_{i=1}^m y_i$ and $Y_n = \sum_{i=1}^n y_i$.

Proof. Subtracting the left by right hand sides of the inequality, we have

$$\begin{aligned}
&\left[Y_m\mu_m^2 - (Y_m + y_0)\mu_{m+1}^2\right] - \left[n\mu_n^2 - (Y_n + y_0)\mu_{n+1}^2\right] && \text{(N5.21)}\\
&= -\left[x_0^2 y_0 - \frac{Y_m y_0(\mu_m - x_0)^2}{Y_m + y_0}\right] + \left[x_0^2 y_0 - \frac{Y_n y_0(\mu_n - x_0)^2}{Y_n + y_0}\right] \quad (\because \text{Lemma N5.4.1}) && \text{(N5.22)}\\
&= \frac{y_0(\mu_m - x_0)^2}{1 + \frac{y_0}{Y_m}} - \frac{y_0(\mu_n - x_0)^2}{1 + \frac{y_0}{Y_n}} && \text{(N5.23)}\\
&\leq 0 \quad (\because \mu_n \geq \mu_m \geq x_0,\ Y_n \geq Y_m,\ y_0 > 0) && \text{(N5.24)}
\end{aligned}$$

Therefore, the original inequality is true. □

Lemma N5.4.3 (Convex quadrangle inequality)**.** *We define the weighted squared mean function*

$$w(j, i) = (Y_i - Y_{j-1})\mu^2(j, i) \tag{N5.25}$$

where $\mu(j, i)$ is the weighted mean function for x_i to x_j defined in Eq (N5.1). For any four increasing indices $0 \leq i_1 \leq i_2 \leq i_3 \leq i_4 \leq n-1$ to sequence $x_0, \ldots, x_{n-1}$ already sorted in ascending order, $w(j, i)$ satisfies the convex quadrangle inequality:

$$w(i_2, i_3) + w(i_1, i_4) \leq w(i_2, i_4) + w(i_1, i_3) \tag{N5.26}$$

Proof. We prove the theorem by repeatedly applying Lemma N5.4.2:

$$
\begin{aligned}
& w(i_2, i_3) - w(i_2, i_4) && \text{(N5.27)}\\
= & (Y_{i_3} - Y_{i_2-1})\mu^2(i_2, i_3) - (Y_{i_4} - Y_{i_2-1})\mu^2(i_2, i_4) && \text{(N5.28)}\\
\le & (Y_{i_3} - Y_{i_2-2})\mu^2(i_2-1, i_3) - (Y_{i_4} - Y_{i_2-2})\mu^2(i_2-1, i_4) \quad (\because \text{Lemma N5.4.2}) && \text{(N5.29)}\\
\le & (Y_{i_3} - Y_{i_2-3})\mu^2(i_2-2, i_3) - (Y_{i_4} - Y_{i_2-3})\mu^2(i_2-2, i_4) \quad (\because \text{Lemma N5.4.2}) && \text{(N5.30)}\\
& \cdots (\text{keep extending the index from } i_2 \text{ down to } i_1 \text{ by Lemma N5.4.2}) &&\\
\le & (Y_{i_3} - Y_{i_2-(i_2-i_1)-1})\mu^2(i_2-(i_2-i_1), i_3) && \text{(N5.31)}\\
& \quad - (Y_{i_4} - Y_{i_2-(i_2-i_1)-1})\mu^2(i_2-(i_2-i_1), i_4) && \text{(N5.32)}\\
= & (Y_{i_3} - Y_{i_1-1})\mu^2(i_1, i_3) - (Y_{i_4} - Y_{i_1-1})\mu^2(i_1, i_4) && \text{(N5.33)}\\
= & w(i_1, i_3) - w(i_1, i_4) && \text{(N5.34)}
\end{aligned}
$$

which is equivalent to the inequality in Eq (N5.26). □

Theorem N5.4.4 (Monotonic increasing of cluster boundary). *Given that $j_q(i-1)$ is the largest index in cluster q of an optimal $(q+1)$-clustering $C^*(q+1, i)$ on the first i points, the largest index $j_q(i)$ in cluster q of some optimal $(q+1)$-clustering $C(q+1, i+1)$ on the first $i+1$ points must increase and satisfy*

$$j_q(i) \ge j_q(i-1) \quad (q < i < n) \tag{N5.35}$$

Proof. We write the optimal $(q+1)$-clustering for the first i points $x_0, \ldots, x_{i-1}$ as

$$C^*(q+1, i) = \left\{ \underbrace{[0,\, j_1^*]}_{\text{cluster } 0 \text{ of size } n_1^*},\ \underbrace{[j_1^*+1, j_2^*]}_{\text{cluster } 1 \text{ of size } n_2^*},\ \cdots,\ \underbrace{[j_{q-1}^*+1, j_q^*]}_{\text{cluster } q-1 \text{ of size } n_{q-1}^*},\ \underbrace{[j_q^*+1, i-1]}_{\text{cluster } q \text{ of size } n_q^*} \right\}$$

with weighted cluster means $\mu_0^*, \ldots, \mu_q^*$, and weighted cluster sizes n_1^*, …, n_q^*. By definition, we have $j_q^* + 1 = j_q(i-1)$.

Let $C(q+1, i)$ be a $(q+1)$-clustering of $x_0, \ldots, x_{i-1}$, not necessarily optimal:

$$C(q+1, i) = \left\{ \underbrace{[0,\, j_1]}_{\text{cluster } 0 \text{ of size } n_0},\ \underbrace{[j_1+1, j_2]}_{\text{cluster } 1 \text{ of size } n_1},\ \cdots,\ \underbrace{[j_{q-1}+1, j_q]}_{\text{cluster } q-1 \text{ of size } n_{q-1}},\ \underbrace{[j_q+1, i-1]}_{\text{cluster } q \text{ of size } n_q} \right\}$$

satisfying the condition that j_q, the upper boundary of cluster $q-1$ of $C(q+1, i)$, is no greater than j_q^*, the upper boundary of cluster $q-1$ of $C^*(q+1, i)$:

$$j_q \le j_q^* \tag{N5.36}$$

We use $\mu_0, \ldots, \mu_q$ to denote the means of cluster 0 to q of $C(q+1, i)$. Thus, for the two clusters $(j_q, i-1]$ of weighted size n_q with weighted mean μ_q and $(j_q^*, i-1]$ of weighted size n_q^* with weighted mean μ_q^*, we have

$$
\begin{aligned}
n_q &\ge n_q^* && \text{(N5.37)}\\
\mu_q &\le \mu_q^* \le x_i && \text{(N5.38)}
\end{aligned}
$$

Now we define $C(q+1, i+1)$ as a $(q+1)$-clustering of $x_0, \ldots, x_i$ after inserting x_i to cluster q of $C(q+1, i)$:

$$C(q+1, i+1) = \left\{ \underbrace{[0, j_1]}_{\text{cluster } 0 \text{ of size } n_0}, \underbrace{[j_1+1, j_2]}_{\text{cluster } 1 \text{ of size } n_1}, \cdots, \underbrace{[j_{q-1}+1, j_{q-1}]}_{\text{cluster } q-1 \text{ of size } n_{q-1}}, \underbrace{[j_q+1, i]}_{\text{cluster } q \text{ of size } n_q + y_i} \right\}$$

The first q clusters of $C(q+1, i+1)$ have the same means with $C(q+1, i)$. We use μ_q^+ to denote the mean of cluster q of $C(q+1, i+1)$.

Let $C'(q+1, i+1)$ be a $(q+1)$-clustering of $x_0, \ldots, x_i$ obtained by inserting x_i to cluster q of $C^*(q+1, i)$. $C'(q+1, i+1)$ differs from $C^*(q+1, i)$ only in cluster q. The size of cluster q of $C'(q+1, i+1)$ is $n_q^* + y_i$ and we use μ_q' to denote the new cluster mean. We can transform $SSQ(C'(q+1, i+1))$ as follows:

$$SSQ(C'(q+1, i+1)) \tag{N5.39}$$

$$= \sum_{l=0}^{i} y_l x_l^2 - \left[\left(\sum_{p=0}^{q} n_p^* \mu_p^{*2} \right) + (n_q^* + y_i)\mu_q'^2 - n_q^* \mu_q^{*2} \right] \tag{N5.40}$$

$$= y_i x_i^2 + SSQ(C^*(q+1, i)) - (n_q^* + y_i)\mu_q'^2 + n_q^* \mu_q^{*2} \tag{N5.41}$$

$$\leq y_i x_i^2 + SSQ(C(q+1, i)) - (n_q^* + y_i)\mu_q'^2 + n_q^* \mu_q^{*2} \quad (\because \text{Optimality of } C^*) \tag{N5.42}$$

$$= \sum_{l=0}^{i} y_l x_l^2 - \left(\sum_{p=0}^{q} n_p \mu_p^2 \right) - (n_q^* + y_i)\mu_q'^2 + n_q^* \mu_q^{*2} \tag{N5.43}$$

$$= \left[\sum_{l=0}^{i} y_l x_l^2 - \sum_{p=0}^{q-1} n_p \mu_p^2 - (n_q + y_i)\mu_q^{+2} \right] + (n_q + y_i)\mu_q^{+2} - n_q \mu_q^2 - (n_q^* + y_i)\mu_q'^2 + n_q^* \mu_q^{*2} \tag{N5.44}$$

$$= SSQ(C(q+1, i+1)) + [n_q^* \mu_q^{*2} + (n_q + y_i)\mu_q^{+2}] - [n_q \mu_q^2 + (n_q^* + y_i)\mu_q'^2]$$
$$(\because \text{Definition of } SSQ(C(q+1, i+1))) \tag{N5.45}$$

$$\leq SSQ(C(q+1, i+1)) \quad (\because \text{Applying conditions in Eq (N5.38) and Lemma N5.4.3}) \tag{N5.46}$$

implying that as long as $j_q \leq j_q^*$, $C(q+1, i+1)$ is never better than $C'(q+1, i+1)$. Therefore the optimal k-clustering $C^*(q+1, i+1)$ that always uses the largest optimal index must satisfy $j_q(i) - 1 \geq j_q^*$, equivalent to

$$j_q(i) \geq j_q^* + 1 = j_q(i-1) \tag{N5.47}$$

which is exactly the inequality in Eq (N5.35). □

Corollary N5.4.5. *The lower decision boundaries of cluster* q *of some optimal clusterings* $C^*(q+1, q+1), \ldots, C^*(q+1, n)$ *increase monotonically:*

$$j_q(q) \leq j_q(q+1) \leq \cdots \leq j_q(n-1)$$

Proof. The corollary follows immediately from Theorem N5.4.4. □

Corollary N5.4.5 leads to a recursive algorithm Fill-Row() to fill row q of S and J. Algorithm Weighted-Univariate-Clustering-(WUC)-Log-Linear(*x*,*k*) is the entry point. In computing each row in matrices S and J, Fill-Row() calls Algorithm Find-Minimum() to search the largest lower decision boundary of cluster q between *jmin* and *jmax* that minimizes the distance measure for the center element at column i. Then Fill-Row() recursively solves entries in row q before and after column i, respectively.

Find-Minimum(*q*, *i*, *jmin*, *jmax*, *S*, *J*, *s*())

```
if q > i
    S[q, i] = ∞
elseif q ≡ 0 or i ≡ 0
    S[q, i] = s(0, i)
    J[q, i] = 0
else
    S[q, i] = S[q − 1, i − 1]
    J[q, i] = i
    jmin = max(jmin, q)
    jmax = min(jmax, i)
    for j = jmin to jmax
        if S[q − 1, j − 1] + s(j, i) ≤ S[q, i]
            S[q, i] = S[q − 1, j − 1] + s(j, i)
            J[q, i] = j
return
```

Fill-Row(*q*, *l*, *h*, *jmin*, *jmax*, *S*, *J*, *s*())

```
if l ≤ h
    i = (l + h)/2 // integer division
    Find-Minimum(q, i, jmin, jmax, S, J, s())
    Fill-Row(q, l, i − 1, jmin, J[q, i], S, J, s())
    Fill-Row(q, i + 1, h, J[q, i], jmax, S, J, s())
return
```

Weighted-Univariate-Clustering-(WUC)-Log-Linear(*x*,*y*,*k*)

```
n = length(x)
Compute running sums Z on x
Compute running sums of squares Q on x
Compute running sums Y on y
Define function s() by Eq (N5.13) using Z, Y, and Q
for q = 0 to k-2
    Fill-Row(q, q, n − 1, q, n − 1, S, J, s())
Fill-Row(k − 1, n − 1, n − 1, k − 1, n − 1, S, J, s())
return S and J
```

Theorem N5.4.6 (Log-linear runtime). *Let* $n = h - l + 1$ *be the number of elements to fill in row* q *of matrices* S *and* J*. Let* $m = jmax - jmin + 1$ *be the number of positions to search for* $j_q(i)$ *for* $i \in [l, h]$*. The runtime of* Fill-Row() *on the subproblem defined by* m *and* n *is*

$$T(n, m) = T(n/2, am) + T(n/2, (1 - a)m) + m \quad \text{(N5.48)}$$

where a*, a fractional number in* $[0, 1]$*, can change from iteration to iteration depending on the input data. Then* $T(n, m) = O(m \lg n)$*.*

Proof. We prove the runtime using the substitution method. We first establish the base cases for $n = 2, 3$:

$$T(2, m) = 2m \le c \cdot m \cdot \lg 2 = cm \quad \text{(N5.49)}$$

$$T(3, m) = 3m \le c \cdot m \cdot \lg 3 = cm \lg 3 \quad \text{(N5.50)}$$

which are true for $c \ge 2$.

With the base cases, we can assume the following induction hypotheses are true for problems whose size parameters are smaller than n and m:

$$T(n/2, am) \le cam \lg(n/2) \text{ and } T(n/2, (1-a)m) \le c(1-a)m \lg(n/2) \tag{N5.51}$$

By induction, we can derive an upper bound for $T(n, m)$ as follows:

$$\begin{aligned} T(n,m) &= T(n/2, am) + T(n/2, (1-a)m) + m && \text{(N5.52)} \\ &\le cam \lg(n/2) + c(1-a)m \lg(n/2) + m && \text{(N5.53)} \\ &= cm \lg n + (1-c)m && \text{(N5.54)} \\ &\le cm \lg n \quad (c \ge 1) && \text{(N5.55)} \end{aligned}$$

The last inequality is true as long as $c \ge 1$. After integrating the requirement of $c \ge 2$ in the base cases, we have

$$T(n,m) \le cm \lg n \quad (c \ge 2) \tag{N5.56}$$

It is therefore true by the definition of the big-O notation that $T(n, m) = O(m \lg n)$ and we have just proved the theorem. □

Corollary N5.4.7. *Algorithm* Fill-Row *takes* $O(n \lg n)$ *to compute the entire row* q *of* n *elements in* S *and* J.

Proof. After replacing m by n in $T(n, m)$ in Theorem N5.4.6, we immediately have $T(n, n) = O(n \lg n)$. $T(n, n)$ is the time to fill n entries in row q, each with a search range of 0 to $n-1$, in $k \times n$ matrix S and J. □

In the initial call to Fill-Row(), m and n are equal to the total number of input points. During the recurrence, m can be greater than n and can be split very unevenly between the two recursive calls. However, the total cost at one level of the recursion tree is always equal to the total number of input points. The height of the recursion tree is at most $1 + \lg n$. Therefore, the runtime for dynamic programming is $O(n \lg n)$, better than $O(n^2)$. Counting the time to sort x and the linear time to compute the last rows in S and J, we arrive at a total runtime of $O(kn \lg n)$. The space complexity remains $O(kn)$.

N5.5 A linear time solution to weighted univariate clustering

We sped up the dynamic programming further to linear time by taking advantage of a *total monotone* property of the univariate clustering problem. Stronger than the monotone property, the total monotone property states that even if we constrain the cluster boundaries to fall on a subset of the data points, the constrained optimal boundaries will not decrease if additional points greater than the current points in the subproblem are inserted. With this property, the divide-and-conquer strategy to fill a row of the dynamic programming matrices reduces the problem into one subproblem of half the size—instead of two in the log-linear time solution—removing the $\lg n$-factor from the $O(n \lg n)$ time solution. Aggarwal et al. (1987) provided the centerpiece known as the SMAWK algorithm to search row maxima in a totally monotonic matrix. After establishing a concave quadrangle inequality for the sum of squared distance function $s(j, i)$, we will prove that the subproblems of weighted univariate clustering can be formulated as problems to search row minima of totally monotonic matrices. We further design an in-place algorithm to perform the matrix column reduction equivalently in an array, instead of deleting matrix columns. We prove the algorithm is correct and derive its asymptotic runtime.

N5.5.1 The univariate clustering problem is totally monotonic

Theorem 1 (Concave quadrangle inequality). *For any four increasing indices $0 \le i_1 \le i_2 \le i_3 \le i_4 \le n-1$ to sequence $x_0, \ldots, x_{n-1}$ already sorted in ascending order, $s(j,i)$ satisfies the concave quadrangle inequality:*

$$s(i_2, i_3) + s(i_1, i_4) \ge s(i_2, i_4) + s(i_1, i_3) \tag{N5.57}$$

Proof. We prove the above inequality based on the convex quadrangle inequality regarding $w(j,i)$ from Lemma N5.4.3. Rewriting $s(j,i)$, we have

$$s(j,i) = \left[\sum_{l=j}^{i} y_l x_l^2\right] - (Y_i - Y_{j-1})\mu^2(j,i) = \left[\sum_{l=j}^{i} y_l x_l^2\right] - w(j,i) \tag{N5.58}$$

It then follows that

$$\begin{aligned} s(i_2, i_3) + s(i_1, i_4) &= \left[\sum_{l=i_2}^{i_3} y_l x_l^2\right] - w(i_2, i_3) + \left[\sum_{l=i_1}^{i_4} y_l x_l^2\right] - w(i_1, i_4) && \text{(N5.59)} \\ &= \left[\sum_{l=i_2}^{i_4} y_l x_l^2\right] - w(i_2, i_3) + \left[\sum_{l=i_1}^{i_3} y_l x_l^2\right] - w(i_1, i_4) && \text{(N5.60)} \\ &\ge \left[\sum_{l=i_2}^{i_4} y_l x_l^2\right] - w(i_2, i_4) + \left[\sum_{l=i_1}^{i_3} y_l x_l^2\right] - w(i_1, i_3) \quad (\because \text{Lemma N5.4.3}) && \text{(N5.61)} \\ &= s(i_2, i_4) + s(i_1, i_3) && \text{(N5.62)} \end{aligned}$$

which is exactly the inequality in Eq (N5.57). □

Now we transform the weighted univariate clustering problem to $k-1$ matrix search problems. For each q ($0 < q < k$), we define an $n \times n$ matrix $A(q)$ by

$$A(q)_{i,j} = \begin{cases} S[q-1,\, j-1] + s(j,i) & 1 \le q \le j \le i < n \\ +\infty & 0 \le j < q \text{ or } i < j < n \end{cases} \tag{N5.63}$$

which we call clustering matrix. Although $A(q)$ depends on q, our derivation next will be for a fixed q and we thus drop q to simplify $A(q)$ to A. We also simplify $j_q(i)$ defined in Eq (N5.10) to $j(i)$—the largest index to the minimum element in row i of A.

Definition 2. *Matrix A is monotonic if and only if $j(i_1) \le j(i_2)$ is true for any row numbers $i_1 < i_2$.*

Definition 3. *Matrix A is totally monotonic if and only if every sub-matrix of A is monotonic.*

Lemma 2 (2×2 matrix monotonicity). *Let A' be a 2×2 sub-matrix of A defined by*

$$A' = \begin{bmatrix} A_{i_1, j_1} & A_{i_1, j_2} \\ A_{i_2, j_1} & A_{i_2, j_2} \end{bmatrix} \tag{N5.64}$$

where $i_1 < i_2$ and $j_1 < j_2$. Let $j'(i') \in [0,1]$ be the largest column index of the minimum element in row $i' \in [0,1]$ in A'. Then we must have $j'(0) \le j'(1)$.

Proof. If $A'_{0,0} < A'_{0,1}$, then $j'(0) = 0$. As $j'(1)$ can only take the value of 0 or 1, it follows immediately that $j'(0) \le j'(1)$.

Now we examine the situation when $A'_{0,0} \ge A'_{0,1}$. If $j_1 < j_2 \le i_1 < i_2$, we have

$$
\begin{aligned}
0 &\le A'_{0,0} - A'_{0,1} && \text{(N5.65)}\\
&= A_{i_1,j_1} - A_{i_1,j_2} && \text{(N5.66)}\\
&= \{S[q-1,\, j_1-1] + s(j_1,i_1)\} - \{S[q-1,\, j_2-1] + s(j_2,i_1)\} && \text{(N5.67)}\\
&= S[q-1,\, j_1-1] - S[q-1,\, j_2-1] + s(j_1,i_1) - s(j_2,i_1) && \text{(N5.68)}\\
&\le S[q-1,\, j_1-1] - S[q-1,\, j_2-1] + s(j_1,i_2) - s(j_2,i_2) \quad (\because \text{Theorem 1}) && \text{(N5.69)}\\
&= \{S[q-1,\, j_1-1] + s(j_1,i_2)\} - \{S[q-1,\, j_2-1] + s(j_2,i_2)\} && \text{(N5.70)}\\
&= A_{i_2,j_1} - A_{i_2,j_2} && \text{(N5.71)}\\
&= A'_{1,0} - A'_{1,1} && \text{(N5.72)}
\end{aligned}
$$

which implies that either $A'_{1,0} > A'_{1,1}$ giving rise to $j'(1) = 1$; or $A'_{1,0} = A'_{1,1}$ where by the definition of $j'(i)$ on A', the tie is broken by taking the larger index value $j'(1) = 1$, not 0. Therefore $j'(0) = j'(1) = 1$, still satisfying $j'(0) \le j'(1)$. If $j_1 < i_1 \le j_2 < i_2$, then $A'_{0,0} < A'_{0,1} = \infty$ leading to $j'(0) = 0$. Regardless of the value of $j'(1)$, either 0 or 1, it must follows $j'(0) \le j'(1)$. ☐

Theorem 3 (Total monotonicity). *Clustering matrix A is totally monotonic.*

Proof. This theorem follows by applying mathematical induction from monotonic 2×2 sub-matrices (Lemma 2) to derive monotonic $2 \times m'$ ($m' \le n$) and then any sized $n' \times m'$ ($n' \le n$) monotonic sub-matrices of A. ☐

N5.5.2 Linear-time search space reduction

The linear-time solution to totally monotonic matrix search problems relies on an efficient search space reduction algorithm called Reduce(*A*) (Aggarwal et al., 1987). It iteratively trims down columns in totally monotonic matrix A until A has no more columns than rows. The central idea is that *N* rows in matrix A requires at most *N* columns in A for optimal solutions. If A is totally monotonic, it is always possible to remove the extra columns within linear time $O(N + M)$. If a matrix has $M \le N$ columns, then the algorithm does not perform column reduction.

We adapt the original Reduce algorithm (Aggarwal et al., 1987) to Reduce-Min with three changes. Reduce-Min preserves potential row minima instead of maxima; the matrix is 0-based instead of 1-based; and a tie is broken by choosing the larger column index, to be consistent with previous versions of `Ckmeans.1d.dp` packages (Wang and Song, 2011).

Definition 4. *We call an entry $A_{i,j'}$ infeasible if $j' \ne j(i)$. Column j' of A is infeasible if $A_{i,j'}$ is infeasible for every i. When there are multiple optimal solutions, the one with the largest indices is feasible and all others infeasible. The infeasible entries are referred to as dead in (Aggarwal et al., 1987).*

As maintaining a copy of matrix A would require at least $\Omega(NM)$ time, the algorithm must compute only needed entries in A in constant time without storing the entire matrix A. For weighted univariate clustering, this is possible by maintaining only indices of feasible columns in A using a stack (Eppstein, 2005) or a pre-allocated linked list of input size (Luessi et al., 2009). Still, these implementations require either dynamic memory allocation or pointer maintenance within the while-loop, thus carrying considerable runtime overhead. To reduce this overhead, we accomplish column reduction in an array of length M in place as given in the Reduce-Min-In-Place algorithm.

This strategy moves feasible column indices towards the beginning of the input column index array without deleting memory used by infeasible column indices. Inside the while-loop, the column index array is used in place without additional memory allocation or deallocation. It realizes the original Reduce algorithm using the simplest data structure with minimum runtime and memory overhead.

Theorem 4. *The algorithm* Reduce-Min-In-Place *always terminates.*

Proof. We suppose the algorithm would run into an infinite while-loop. The number of iterations when $m = (l+1) + (M - r)$ is decremented is at most $M - N$ because the algorithm does not increase the value of m. As m can be decreased only in the second and third if-conditions, both conditions can be run only a finite number of times. Therefore, after the last decrement of m, the algorithm must repeatedly satisfy the first if-condition and l increases by 1 until $l + 1 = p = N - 1$. In the iteration right after p first becomes $N - 1$, only the second and the third if-conditions are possible, which will result in another decrement of m. This contradicts the fact that the last decrement of m has already completed. Therefore, the algorithm must terminate. □

Theorem 5. *The algorithm* Reduce-Min-In-Place *correctly removes only infeasible candidate columns from the input matrix. The output matrix has no more columns than rows. Additionally, the output matrix is still totally monotonic.*

Proof. We use loop invariant to prove that Reduce-Min-In-Place will return a matrix that contain all feasible columns for each row i in A, based on the correctness argument for the Reduce algorithm (Aggarwal et al., 1987). Let B be an $N \times (l+2)$ sub-matrix containing columns $cols[0..l]$ and column $cols[r]$ of A before each **while** iteration:

$$B = \begin{bmatrix} \text{column } 0 & \cdots & \text{column } l & \text{column } l+1 \\ A_{0,cols[0]} & \cdots & A_{0,cols[l]} & A_{0,cols[r]} \\ \vdots & \ddots & \vdots & \vdots \\ A_{l+1,cols[0]} & \cdots & A_{l+1,cols[l]} & A_{l+1,cols[r]} \\ \vdots & \ddots & \vdots & \vdots \\ A_{N-1,cols[0]} & \cdots & A_{N-1,cols[l]} & A_{N-1,cols[r]} \end{bmatrix} \quad (l \geq 0)$$

When $l < 0$, B contains only column $cols[r]$ of A. Matrix B is useful for the purpose of proof but unnecessary in the program.

Loop invariants: Before each **while** iteration, (1) the elements in the upper triangular region above the diagonal line $(B_{0,0}, \ldots, B_{l+1,l+1})$ of matrix B are infeasible; (2) the columns that are detected as infeasible in A are covered by neither $cols[0..l]$ nor $cols[r..M-1]$; (3) $l < r$; (4) The array of $cols[0..l]$ concatenated by $cols[r..M-1]$ is sorted in increasing order;

Initialization: Before the first iteration, B is an $N \times 1$ matrix with an empty upper triangular region and thus elements in the region infeasible giving rise to loop invariant (1). The removed columns are empty and thus infeasible. Thus (2) is also true. (3) is true because $l = -1 < r = 0$. (4) As $cols[0..l]$ is empty and $cols[r..M-1]$ is sorted because it contains exactly $0, \ldots, M-1$, the concatenation of the two arrays are sorted in increasing order.

Maintenance: We will show that the loop invariants are maintained for each of the three conditions based on the total monotonicity of A established by Theorem 3.

The first condition $A_{p,j} < A_{p,j+}$ and $p < N - 1$ is true. The condition suggests that in the 2×2 matrix

$$A' = \begin{bmatrix} A_{i,j} & A_{i,j+} \\ A_{p,j} & A_{p,j+} \end{bmatrix} \quad 0 \leq i < p \qquad \text{(N5.73)}$$

and thus $j'(1) = 0$. By loop invariant (4), we have $j < j_+$ and A', a legitimate sub-matrix of A, is monotonic. Lemma 2 can thus be applied to A' so that $j'(0) \leq j'(1) = 0$, implying column 1 (the second column) of A' is infeasible. Collectively, $A_{0,j_+}, \ldots, A_{p,j_+}$ in column j_+ from row $i = 0$ to $i = p$ are infeasible. A_{p,j_+} is infeasible because it is worse than $A_{p,j}$. By loop invariant (1) before the current iteration, column $j = cols[r]$ of A was column p of B already satisfying loop invariant (1). Right before the next iteration, $cols[l]$ records j and r was incremented so that $cols[r]$ records j_+. Thus the dimension of the updated B is $N \times (l+2)$ with the last two columns from columns j and j_+ of A. The diagonal element of the last column of updated B is $B_{p+1,p+1}$. Above it are exactly $p+1$ infeasible elements $A_{0,j_+}, \ldots, A_{p,j_+}$. Therefore, in combination with the previous columns in B, loop invariant (1) is maintained.

Since there is no change to the infeasible columns, loop invariant (2) carries over and stays true. Loop invariant (3) $l < r$ maintains true as both l and r were incremented. (4) also stays true as the smallest element j from a sorted array was moved from $cols[r..M-1]$ to become the largest element in the sorted array $cols[0..l]$.

The second condition $A_{N-1,j} < A_{N-1,j_+}$ and $p = N-1$ is true. Matrix B remains unchanged as j was still recorded in $cols[r]$ after the iteration and no changed occurred to l or $cols[l]$. Thus loop invariant (1) is maintained after this condition.

Replacing p by $N-1$, we can deduce that A_{i,j_+} $(0 \leq i \leq N-1)$ are infeasible using the same argument for the first condition. This suggests that the entire column j_+ of A is infeasible. As $cols[r+1]$ is overwritten by j, the newly detected infeasible column j_+ does not belong to $cols[0..l]$ or $cols[r..M-1]$, suggesting loop invariant (2) is true. As neither the value of l nor $cols[0..l]$ was changed during the iteration, loop invariant (1) carries over and is still true. Loop invariant (3) $l < r$ maintains true as only r was incremented. Loop invariant (4) is true as the smallest element j from a sorted array replaced the second smallest element in $cols[r..M-1]$, while the sorted array $cols[0..l]$ stays unchanged.

The third condition $A_{p,j} \geq A_{p,j_+}$ is true. We define the 2×2 matrix

$$A' = \begin{bmatrix} A_{p,j} & A_{p,j_+} \\ A_{i,j} & A_{i,j_+} \end{bmatrix} \quad p < i \leq N-1 \tag{N5.74}$$

The inequality of $A_{p,j} \geq A_{p,j_+}$ suggests $j'(0) = 1$ (when $A_{p,j} = A_{p,j_+}$, the tie is broken by taking the larger index 1, not 0). By loop invariant (4) before the iteration, we have $j < j_+$ and thus A' is a legitimate sub-matrix of A. The total monotonicity of A indicates that A' is monotonic (Lemma 2) such that $1 = j'(0) \leq j'(1)$, making column 0 in A' infeasible. Collectively, $A_{p,j}, \ldots, A_{N-1,j}$ within column j from row $i = p$ to $i = N-1$ are infeasible. By definition, the last column p of B is exactly column $cols[r] = j$ of A, implying $A_{0,j}, \ldots, A_{p-1,j}$ are infeasible. Therefore, the entire column j of A is infeasible.

If $p > 0$, the last column of B is effectively removed by $cols[r] = cols[l]$, loop invariant (1) regarding B thus still maintains. As l was reduced by 1, the infeasible column is also removed from $cols[0..l]$. Therefore loop invariant (2) satisfies before the next iteration. Loop invariant (3) $l < r$ maintains true as only l was decremented. Loop invariant (4) is true as the largest element was removed from sorted $cols[0..l]$ and replaced the smallest element in $cols[r..M-1]$.

If $p = 0$, the infeasible column j of A, also the only column of B, is effectively replaced by column j_+ of A with the increment of r by 1, before the next iteration. Therefore loop invariants (1) and (2) maintain. Loop invariant (3) $l < r$ maintains true as only r was incremented. Loop invariant (4) is true as $cols[0..l]$ is empty and the smallest element in $cols[r..M-1]$ was removed.

It is impossible to have $p < 0$ as l can only be deceased when $p > 0$ and the decrement is at most 1.

Termination: Theorem 4 indicates that the algorithm will indeed terminate. If $M \leq N$, the output matrix has no more number of columns than rows; otherwise, the condition for the **while** loop suggests that A will be an $N \times N$ square matrix upon termination. Loop invariant (2) implies that only infeasible columns in the original input matrix A were removed thus all optimal solutions to $j(i)$ are contained the returned columns of A. Loop invariant (4) suggests that the feasible column numbers are maintained in an increasing order, rendering the output matrix as a valid sub-matrix of matrix A, therefore also totally monotonic.

□

N5.5.3 Divide-and-conquer

The SMAWK algorithm for matrix search developed by (Aggarwal et al., 1987) reduces columns from A first, recursively solves a sub-matrix containing the odd rows of A, and then calculates the solutions to the even rows in A. We designed the Fill-Row-SMAWK algorithm following the same strategy without explicitly maintaining matrix A, to calculate an entire row in the dynamic programming matrix S. Fill-Row-SMAWK is called by Algorithm Weighted-Univariate-Clustering-(WUC)-Linear to compute the entire dynamic programming matrix. Find-Minimum-From-Candidates is an auxiliary procedure to find the solution of a single entry in A.

The Reduce-Min-In-Place conducts $O(N+M)$ comparisons, the same with the Reduce algorithm (Aggarwal et al., 1987). Manipulating the array adds only a constant factor and thus the total runtime is still $O(N+M)$. Therefore the runtime of Fill-Row-SMAWK on input matrix of size $N \times M$ is

$$T(N, M) = T(N/2, N/2) + O(N + M) = O(N + M)$$

giving rise to the runtime for the entire row of n elements as

$$T(n) = O(n)$$

The total runtime of clustering on n input points is $O(kn)$ when x is sorted. Although the additional space linear to the input array size is needed to store the running sums and candidate indices during the recursion, the total space complexity remains $O(kn)$ when backtrack must be conducted.

Find-Minimum-From-Candidates(*q*, *i*, *js*, *S*, *J*, *s*())

```
S[q, i] = S[q − 1, i − 1]
J[q, i] = i
for each j in js
    if S[q − 1, j − 1] + s(j, i) ≤ S[q, i]
        S[q, i] = S[q − 1, j − 1] + s(j, i)
        J[q, i] = j
return
```

Fill-Even-Positions (*imin*, *imax*, *istep*, *q*, *js*, *S*, *J*, *s*())

```
istep2 = istep ∗ 2
imin0 = imin
imax0 = imin + ⌊(imax − imin)/istep2⌋ ∗ istep2
r = 0
for i = imin0 to imax0 by istep2
    S[q, i] = S[q − 1, i − 1]
    J[q, i] = i
    if i − istep < imin
        jmin = js[0]
    else
        jmin = J[q, i − istep]
    while js[r] < jmin
        r = r + 1
    if i + istep ≤ imax
        jmax = J[q, i + istep]
    else
        jmax = js[length(js) − 1]
    jmax = min(i, jmax)
    while js[r] ≤ jmax
        j = js[r]
        if S[q − 1, j − 1] + s(j, i) ≤ S[q, i]
            S[q, i] = S[q − 1, j − 1] + s(j, i)
            J[q, i] = j
        r = r + 1
    r = r − 1
```

Fill-Row-SMAWK(*imin*, *imax*, *istep*, *q*, *js*, *S*, *J*, *s*())

```
if imin ≡ imax
    Find-Minimum-From-Candidates(q, imin, js, S, J, s())
else
    N = ⌊(imax − imin)/istep⌋ + 1
    Create a matrix function A(p, j)
    (p = 0, . . . , N − 1 and j = 1, . . . , n − 1) as follows:
    if j = 0
        A(p, j) = s(j, imin + p ∗ istep)
    else
        A(p, j) = S[q − 1, j − 1] + s(j, imin + p ∗ istep)
    js′ = Reduce-Min-In-Place(js, N, A)
    istep2 = istep * 2
    imin1 = imin + istep
    imax1 = imin1 + floor((imax - imin1) / istep2) * istep2
    Fill-Row-SMAWK(imin1, imax1, istep2, q, js′, S, J, s())
    Fill-Even-Positions(imin, imax, istep, q, js, S, J, s())
return
```

Weighted-Univariate-Clustering-(WUC)-Linear(*x*, *y*, *k*)

```
n = length(x)
Compute running sums Z on x
Compute running sums of squares Q on x
Compute running sums Y on y
Define function s() by Eq (N5.13) using Z, Y, and Q
for i = 0 to n − 1
    S[0, i] = s(0, i)
    J[0, i] = 0
for q = 1 to k − 1
    if q < k − 1
        imin = q
    else
        imin = n − 1
    imax = n − 1
    for r = 0 to n − q − 1
        js[r] = r + q      // js: j-candidates
    Fill-Row-SMAWK(imin, imax, 1, q, js, S, s())
return S and J
```

N5.6 Estimating the optimal number of clusters

We assess optimality using the Bayesian information criterion (BIC) directly from the dynamic programming matrix. We define a Gaussian mixture model (GMM) with k components as follows

$$f(x \mid \lambda, \mu, \sigma^2) = \sum_{q=0}^{k-1} \frac{\lambda_q}{\sqrt{2\pi\sigma_q^2}} \exp\left[-\frac{(x-\mu_q)^2}{2\sigma_q^2}\right] \tag{N5.75}$$

where each cluster is treated as a component with three parameters $\lambda_q, \mu_q, \sigma_q^2$. Let $\lambda = (\lambda_0, \ldots, \lambda_{k-1})$ be the nonnegative weight vector of all components. Let $\mu = (\mu_0, \ldots, \mu_{k-1})$ be the mean vector of all

components. Let $\sigma = (\sigma_0, \ldots, \sigma_{k-1})$ be the variance vector of all components. Let the set of indices to x in cluster q be

$$I(q) = \{i \mid c(i) = q, i = 0, \ldots, n-1\} \tag{N5.76}$$

We estimate the GMM parameters for each cluster q $(0 \le q \le k-1)$ by

$$\hat{\lambda}_q = \frac{1}{n} |I(q)| \tag{N5.77}$$

$$\hat{\mu}_q = \frac{1}{n} \sum_{i \in I(q)} x_i \tag{N5.78}$$

$$\hat{\sigma}_q^2 = \begin{cases} \hat{\sigma}^2_{\text{max}} & |I(q)| = 1 \\ \hat{\sigma}^2_{\text{min}} & \text{all } |I(q)| > 1 \text{ points in cluster } q \text{ are equal} \\ \frac{1}{n-1} \sum\limits_{i \in I(q)} (x_i - \hat{\mu}_q)^2 & \text{otherwise} \end{cases} \tag{N5.79}$$

Let d_{min} be the minimum distance between a point in cluster q and a point in its surrounding clusters. We estimate the minimum and maximum variance of a cluster by

$$\hat{\sigma}^2_{\text{min}} = d^2_{\text{min}}(q)/36 \tag{N5.80}$$

$$\hat{\sigma}^2_{\text{max}} = d^2_{\text{min}}(q) \tag{N5.81}$$

With the chosen $\hat{\sigma}^2_{\text{min}}$, the midpoint between the two closest points falls at the 3σ position. $\hat{\sigma}^2_{\text{max}}$ corresponds to the tightest boundary on both sides before reaching a point in another cluster, used only when the cluster contains a single element.

Using the estimated parameters, we can compute the log-likelihood

$$\log L = \sum_{i=0}^{n-1} \log f(x_i \mid \hat{\lambda}, \hat{\mu}, \hat{\sigma}^2) \tag{N5.82}$$

and then we have

$$\text{BIC}(k) = 2 \log L - (3k-1) \log n \tag{N5.83}$$

where $3k-1$ is the total number of independent parameters, which is one less the total number of parameters because the constraint that $\lambda_0 + \cdots + \lambda_{k-1} = 1$. Then we find the optimal number of parameters k^* that maximizes BIC(k) on x.

The total runtime for selecting an optimal number of clusters is linear in sample size but quadratic in the number of clusters. The cluster boundaries (bins) can be backtracked in $O(k)$ time; estimating the GMM takes $O(n)$ time; and calculating the log-likelihood needs $O(kn)$ time. Thus the runtime to evaluate a single k is $O(kn)$. To evaluate all number of clusters up to k will thus take $O(k^2 n)$ time, shadowing the $O(n \lg n + kn)$ time to fill in the dynamic programming matrix.

We next evaluated the performance of the described method to estimate the number of clusters in given univariate data. We used 100 datasets of sample size ranging from 11 to 7980 from R package `datasets` (version 3.3.1). Their numerical columns are vectorized to arrays before being used. Then we applied optimal univariate clustering to estimate the best GMM models that maximizes BIC on the data. We also ran the expectation maximization (EM) algorithm implemented in R package `mclust` (Fraley and Raftery, 2002; Fraley et al., 2012, 2016) for general multivariate GMMs. The package also uses BIC to select the optimal number of components. Figure N5.1 shows sample-normalized BIC (BIC/n) and its improvement, the runtime, and the estimated number of clusters/components by the two methods. The figure suggests that the two methods achieved comparable BIC (Fig N5.1a,b) but the estimated numbers of optimal clusters/components can differ greatly (Fig N5.1c). Despite being quadratic in k, optimal univariate clustering runs faster than expectation maximization in orders of magnitude.

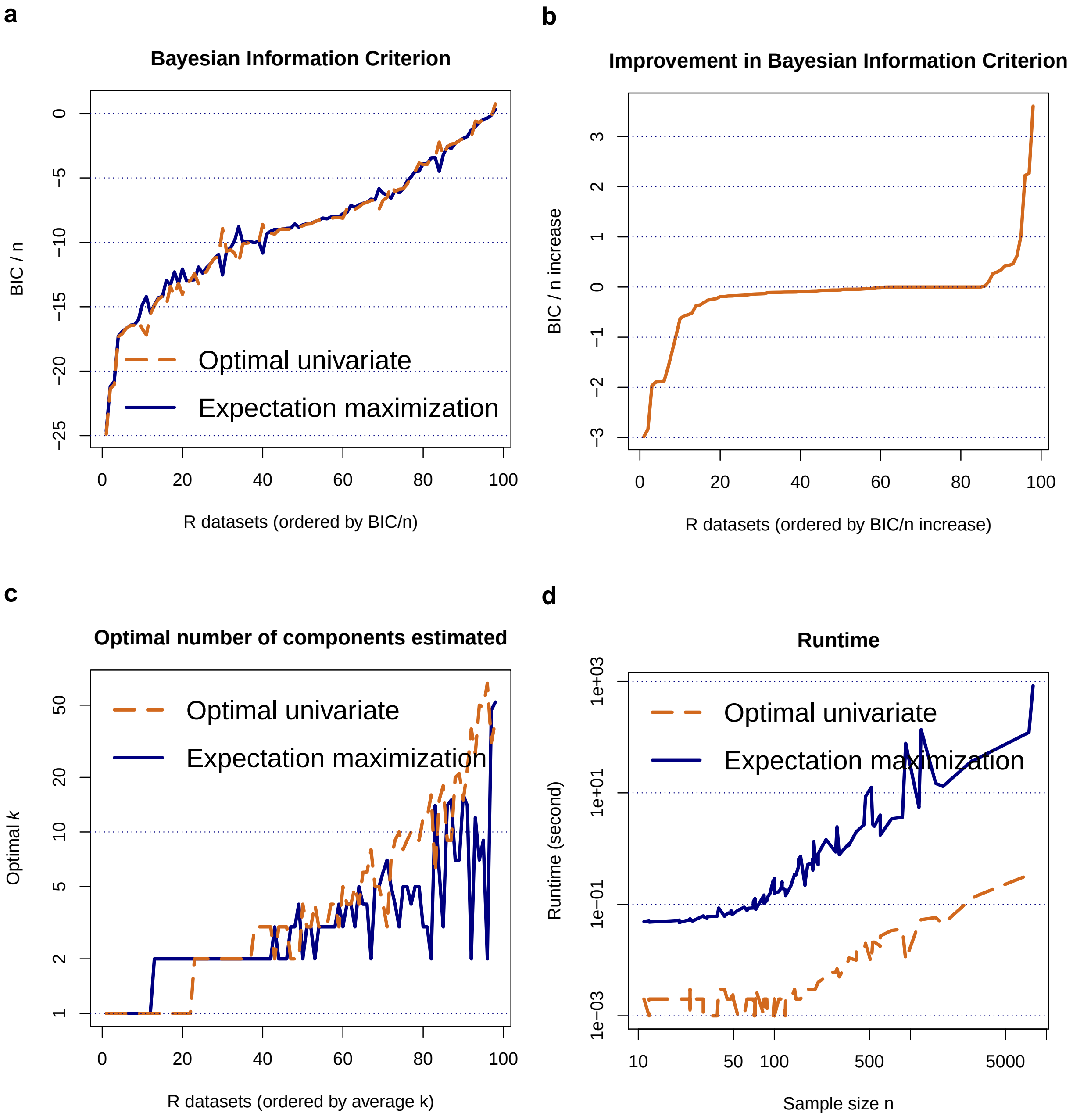

Figure N5.1: Performance of optimal univariate clustering on selecting the optimal number of clusters by Bayesian information criterion (BIC) in comparison with expectation maximization (EM). Exactly 100 datasets in R (version 3.3.1) were used for the evaluation. **a,** The sample size normalized BIC (BIC/n) is comparable between optimal univariate clustering and EM. **b,** On 14 datasets, optimal clustering improved the BIC/n by up to 3.61; on the remaining datasets, EM performed better by up to 2.98. **c,** The estimated optimal numbers of clusters by the two methods are comparable in some cases but vary greatly in others, despite that their BIC values can be similar. **d,** Optimal univariate clustering ran about 100 times faster than the EM algorithm in determining optimal k.

References

Aggarwal, A., Klawe, M. M., Moran, S., Shor, P., and Wilber, R. (1987). Geometric applications of a matrix-searching algorithm. *Algorithmica*, 2(1-4):195–208.

Bellman, R. (1973). A note on cluster analysis and dynamic programming. *Mathematical Biosciences*, 18(3):311–312.

Eppstein, D. (2005). Totally monotone matrix searching algorithms. `https://www.ics.uci.edu/~eppstein/PADS/SMAWK.py`. Accessed: Dec 11, 2016.

Fraley, C. and Raftery, A. E. (2002). Model-based clustering, discriminant analysis and density estimation. *Journal of the American Statistical Association*, 97:611–631.

Fraley, C., Raftery, A. E., Murphy, T. B., and Scrucca, L. (2012). *mclust Version 4 for R: Normal Mixture Modeling for Model-Based Clustering, Classification, and Density Estimation*.

Fraley, C., Raftery, A. E., Scrucca, L., Murphy, T. B., and Fop, M. (2016). *mclust: Gaussian Mixture Modelling for Model-Based Clustering, Classification, and Density Estimation*. R package version 5.2.

Hilferink, M. (2015). Fisher's natural breaks classification. `http://wiki.objectvision.nl/index.php/Fisher's_Natural_Breaks_Classification`. Accessed: Dec 10, 2016.

Luessi, M., Eichmann, M., Schuster, G. M., and Katsaggelos, A. K. (2009). Framework for efficient optimal multilevel image thresholding. *Journal of Electronic Imaging*, 18(1):013004.

Wang, H. and Song, M. (2011). Ckmeans.1d.dp: optimal k-means clustering in one dimension by dynamic programming. *The R Journal*, 3(2):29–33.
