# Supplementary material for: Efficient weighted univariate clustering maps outstanding dysregulated genomic zones in human cancers
Source: Bioinformatics. 2020 Jul 3;36(20):5027–36. doi: 10.1093/bioinformatics/btaa613 (PMC7755420; doi:10.1093/bioinformatics/btaa613)
Supplement: btaa613_Supplementary_Data [file btaa613_supplementary_data.zip › Supp-ReadMe.pdf]

SUPPLEMENTARY INFORMATION SUMMARY

Supplementary Tables

Supplementary Table S1: Zones and associated statistics for maps of genomic zone showing polarity in gene regulation for 17 cancer types, including

- **pid:** TCGA project ID indicating the cancer type
- **chr:** the chromosome where a zone is located
- **z.name:** the name of a zone
- **start:** the starting genomic coordinate of a zone
- **end:** the ending genomic coordinate of a zone
- **n.matched:** the number of matched tumor-normal sample pairs to obtain zones and statistics
- **n.genes:** the number of genes located in a zone
- **n.dn:** the number of sample pairs where genes in a zone are down-regulated in cancer
- **n.eq:** the number of sample pairs where genes in a zone are not differentially expressed in cancer
- **n.up:** the number of sample pairs where genes in a zone are up-regulated in cancer
- **chisq.p.adj:** the adjusted Pearson chi-squared test p-value
- **upordn.pct:** the percentage of up or down-regulated samples accumulated for all genes in a zone

Supplementary Table S2: Common loci consistently polarized in 14 (80%) or more out of 17 human cancer types. Loci are derived from intersecting maps of genomic zone across cancer types. A total of 109 common loci are given with their chromosomal locations and polarity.

Supplementary Table S3: Statistical significance of horizontal continuity along chromosomes in each cancer type. The columns are defined as follows:

- **pid:** TCGA project ID indicating the cancer type
- **chr:** Chromosome
- **non.empty.zones:** the number of non-empty zones along the chromosome
- **pos.zones:** the number of outstanding positive zones along the chromosome
- **neg.zones:** the number of outstanding negative zones along the chromosome
- **padj.pos:** the adjusted P-value for positive zone continuity
- **padj.neg:** the adjusted P-value for negative zone continuity
- **max.len.pos:** the maximum number of consecutive positive zones along the chromosome. '*' indicates the number is statistically significant.
- **max.len.neg:** the maximum number of consecutive negative zones along the chromosome. '*' indicates the number is statistically significant.

Supplementary Figures

Supplementary Figure S1: Maps of genomic zone showing polarity in gene regulation and somatic copy number alteration in 17 cancer types (organized by chromosome).

Supplementary Figure S2: Maps of genomic zone showing polarity in gene regulation and somatic copy number alteration in 17 cancer types (organized by cancer type).

Supplementary Figure S3: Top five most polarized regulation zones for each of 17 cancer types.

Supplementary Notes

Supplementary Note N1: Performance of fast, optimal, and weighted univariate clustering.

Supplementary Note N2: Statistical and biological relevance of detected dysregulated genomic maps in human cancers.

Supplementary Note N3: Summary of top dysregulated genomic zones in human cancers.

Supplementary Note N4: Top somatic copy number alteration zones match known cancer aneuploidy.

Supplementary Note N5: Optimal weighted univariate clustering algorithms and proofs.
