# Supplementary material for: Efficient weighted univariate clustering maps outstanding dysregulated genomic zones in human cancers
Source: Bioinformatics. 2020 Jul 3;36(20):5027–36. doi: 10.1093/bioinformatics/btaa613 (PMC7755420; doi:10.1093/bioinformatics/btaa613)
Supplement: btaa613_Supplementary_Data [file btaa613_supplementary_data.zip › Supp-Table-S2.pdf]

Supplementary Table S2: Common loci consistently polarized in 14 (80%) or more out of 17 human cancer types. Loci are derived from intersecting maps of genomic zone across cancer types. A total of 109 common loci are given with their chromosomal locations and polarity.

Chr	Start (Mb)	End (Mb)	Polarity
1	52.30	52.48	+ > 80%
1	70.60	71.65	- > 80%
1	109.70	109.82	- > 80%
1	180.47	180.71	+ > 80%
1	223.93	224.81	+ > 80%
1	228.40	228.41	+ > 80%
2	27.28	27.52	+ > 80%
2	37.67	38.00	- > 80%
2	74.49	74.63	+ > 80%
2	101.11	101.49	+ > 80%
2	110.85	111.58	+ > 80%
2	112.36	112.88	+ > 80%
2	166.05	167.46	- > 80%
3	27.95	29.52	- > 80%
3	38.02	38.03	- > 80%
3	42.38	43.06	- > 80%
3	58.27	59.91	- > 80%
3	65.03	65.27	- > 80%
3	151.55	152.01	- > 80%
3	196.23	197.11	+ > 80%
4	6.85	7.28	- > 80%
4	15.38	15.82	- > 80%
4	53.80	54.34	- > 80%
4	55.28	55.59	+ > 80%
4	84.75	85.92	- > 80%
4	99.19	99.64	- > 80%
4	100.56	101.00	- > 80%
4	148.03	149.45	- > 80%
4	173.77	176.48	- > 80%
4	185.85	186.40	- > 80%
5	40.49	41.39	- > 80%
5	150.97	151.39	- > 80%
6	26.21	26.28	+ > 80%
6	55.45	56.11	- > 80%
6	67.67	70.16	- > 80%
6	70.85	72.12	- > 80%
6	119.10	119.46	- > 80%
6	133.53	133.61	- > 80%
6	162.35	164.35	- > 80%
6	168.94	169.00	+ > 80%
7	2.16	2.60	+ > 80%
7	5.80	6.18	+ > 80%
7	32.14	32.61	+ > 80%
7	64.88	65.47	+ > 80%
7	65.54	65.60	+ > 80%
7	76.19	76.75	+ > 80%
7	78.92	79.55	- > 80%
7	102.17	102.59	+ > 80%
7	105.27	105.71	+ > 80%
7	130.16	130.24	+ > 80%
7	148.85	149.37	+ > 80%
8	1.05	3.20	- > 80%
8	48.47	50.11	- > 80%
8	93.61	94.03	+ > 80%
8	103.22	103.97	+ > 80%
8	123.22	123.26	+ > 80%
8	123.38	123.56	+ > 80%
8	129.40	130.30	+ > 80%
8	143.27	143.79	+ > 80%
9	17.29	18.13	- > 80%
9	34.39	34.78	- > 80%
9	67.76	68.89	- > 80%
9	69.96	70.11	- > 80%
9	91.05	91.58	- > 80%
10	15.58	16.00	- > 80%
10	44.17	44.58	- > 80%
10	102.62	102.97	- > 80%
11	7.12	8.26	- > 80%
11	29.22	30.84	- > 80%
11	34.00	34.16	+ > 80%
11	74.11	74.55	+ > 80%
11	93.92	94.21	+ > 80%
11	109.34	110.94	- > 80%
11	111.85	112.51	- > 80%
11	118.48	118.75	- > 80%
12	49.04	49.49	+ > 80%
12	66.92	67.84	+ > 80%
12	80.22	80.55	- > 80%
12	85.55	86.27	- > 80%
12	97.74	99.23	+ > 80%
12	99.31	99.37	+ > 80%
12	109.04	109.15	- > 80%
12	123.05	123.74	+ > 80%
13	26.93	27.20	- > 80%
13	72.43	72.96	+ > 80%
14	18.24	20.57	+ > 80%
15	24.27	25.19	- > 80%
15	95.26	97.03	- > 80%
16	2.97	3.03	+ > 80%
16	20.18	20.53	- > 80%
16	56.55	56.66	- > 80%
17	10.12	11.74	- > 80%
17	28.56	28.98	+ > 80%
17	31.69	32.58	+ > 80%
17	37.10	37.90	+ > 80%
17	59.01	59.44	+ > 80%
17	68.87	70.47	- > 80%
18	0	0.38	+ > 80%
18	4.93	5.42	- > 80%
18	33.30	33.72	- > 80%
18	44.08	45.20	- > 80%
18	66.52	66.69	- > 80%
19	53.15	53.25	+ > 80%
19	56.03	56.89	- > 80%
20	24.39	26.50	+ > 80%
20	33.57	33.78	+ > 80%
20	35.43	35.87	+ > 80%
20	48.42	49.38	+ > 80%
X	102.97	103.45	- > 80%
